# Supplementary material for: Addition of alkynes and osmium carbynes towards functionalized dπ–pπ conjugated systems
Source: Nat Commun. 2020 Sep 16;11:4651. doi: 10.1038/s41467-020-18498-2 (PMC7495419; doi:10.1038/s41467-020-18498-2)
Supplement: Supplementary file 1 — Supplementary Information [file 41467_2020_18498_MOESM1_ESM.pdf]

Addition of alkynes and osmium carbynes towards functionalized d_π-p_π conjugated systems

Chen et al.

Supplementary Figures and Tables

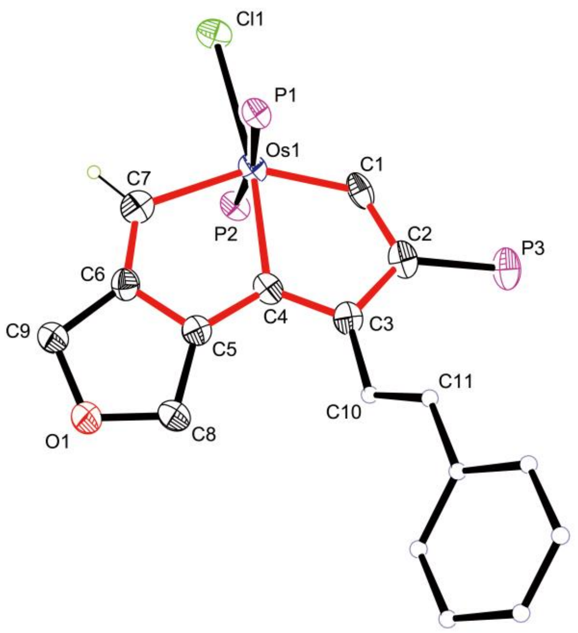

Supplementary Figure 1. X-ray molecular structure for the cation of complex **1a** drawn with 50% probability level. The phenyl groups in PPh_3 are omitted for clarity. Selected bond lengths [Å] and angles [°]: Os1–C1 1.840(5), C2–C1 1.390(8), C3–C2 1.431(7), C4–C3 1.417(7), C4–C5 1.396(7), C6–C5 1.404(7), C7–C6 1.349(7), Os1–C7 2.061(5), Os1–C4 2.111(5), C2–C1–Os1 130.9(4), C1–C2–C3 108.5(4), C4–C3–C2 109.4(5), C3–C4–Os1 118.2(4), C1–Os1–C4 72.5(2), C5–C4–Os1 115.2(4), C4–C5–C6 114.9(5), C7–C6–C5 115.1(5), C6–C7–Os1 118.8(4), C7–Os1–C4 75.8(2).

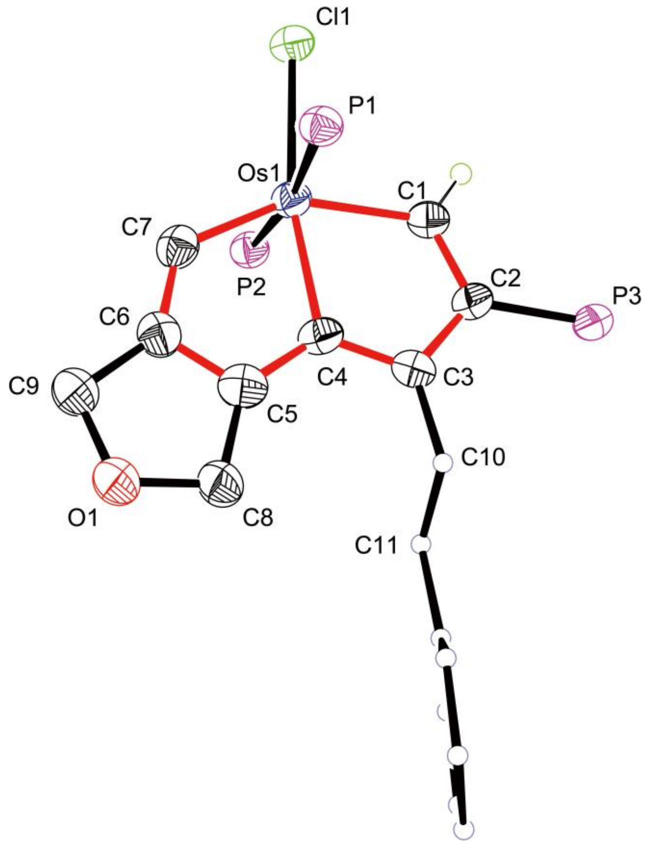

Supplementary Figure 2. X-ray molecular structure for the cation of complex **2a** drawn with 50% probability level. The phenyl groups in PPh_3 are omitted for clarity. Selected bond lengths [Å] and angles [°]: Os1–C1 2.029(6), C1–C2 1.380(8), C3–C2 1.431(8), C3–C4 1.402(8), C5–C4 1.408(9), C6–C5 1.379(9), C6–C7 1.375(9), Os1–C7 1.855(6), Os1–C4 2.107(6), C2–C1–Os1 120.7(4), C1– C2–C3 114.2(5), C4–C3–C2 111.9(5), C3–C4–Os1 118.2(4), C1–Os1–C4 75.0(2), C5– C4–Os1 115.7(4), C6–C5–C4 112.6(6), C7–C6– C5 109.5(5), C6–C7–Os1 128.6(5), C7–Os1– C4 73.5(3).

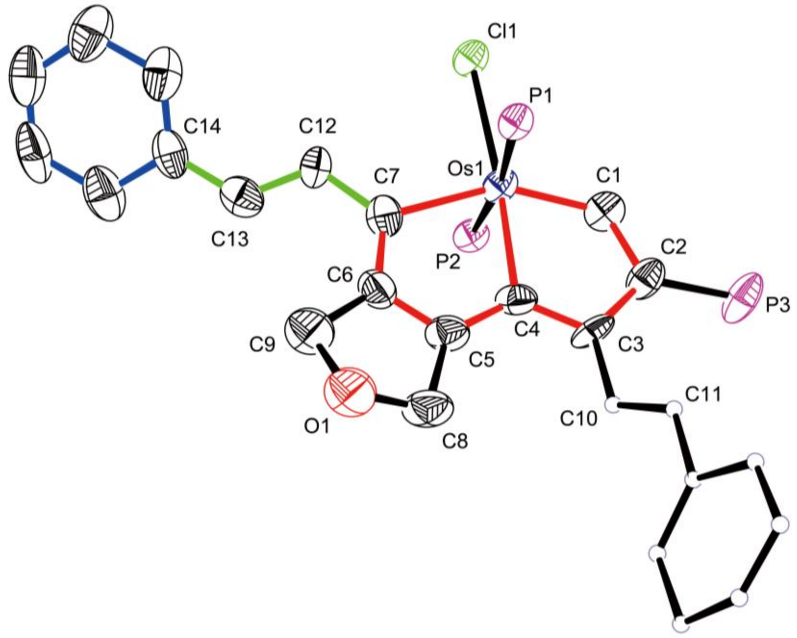

Supplementary Figure 3. X-ray molecular structure for the cation of complex **3** drawn with 50% probability level. The phenyl groups in PPh_3 are omitted for clarity. Selected bond lengths [Å] and angles [°]: Os1–C1 1.844(7), C1–C2 1.390(9), C2–C3 1.437(10), C4–C3 1.389(9), C4–C5 1.402(9), C5–C6 1.392(10), C7–C6 1.397(10), Os1–C7 2.109(6), Os1–C4 2.118(6), C12–C13 1.357(9), C2–C1–Os1 130.0(5), C1–C2–C3 108.9(6), C4–C3–C2 109.4(5), C3–C4–Os1 119.0(5), C1–Os1–C4 72.5(3), C5–C4–Os1 114.7(5), C6–C5–C4 116.2(6), C5–C6–C7 116.8(6), C6–C7–Os1 114.9(5), C7–Os1–C4 77.3(3).

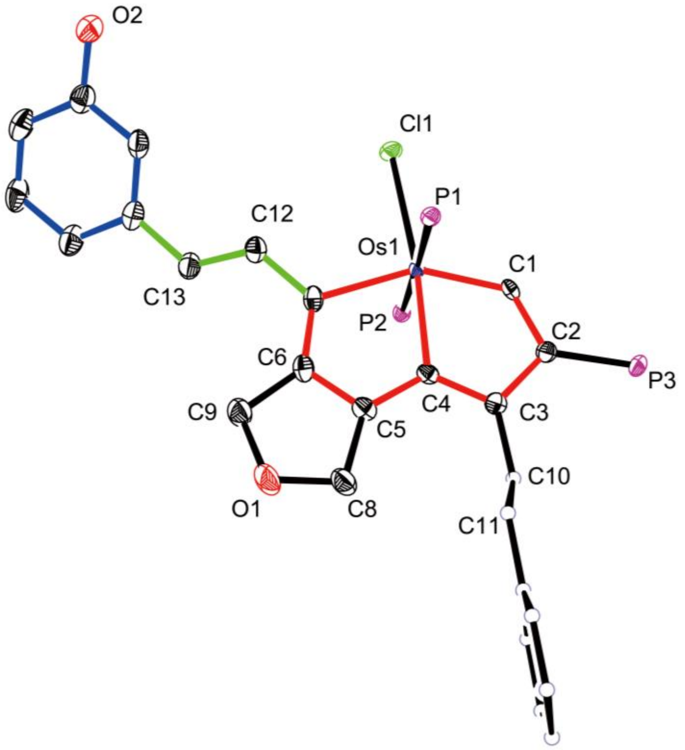

Supplementary Figure 4. X-ray molecular structure for the cation of complex **15** drawn with 50% probability level. The phenyl groups in PPh_3 are omitted for clarity. Selected bond lengths [Å] and angles [°]: Os1–C1 1.832(2), C2–C1 1.401(4), C2–C3 1.435(4), C4–C3 1.402(4), C4–C5 1.406(4), C6–C5 1.391(4), C7–C6 1.392(4), Os1–C7 2.091(3), Os1–C4 2.114(3), C13–C12 1.340(4), C2–C1–Os1 130.2(2), C1–C2–C3 108.3(2), C4–C3–C2 110.4(2), C3–C4–Os1 117.82(19), C1–Os1–C4 73.21(11), C5–C4–Os1 115.41(19), C6–C5–C4 115.2(2), C5–C6–C7 116.5(2), C6–C7–Os1 116.00(19), C7–Os1–C4 76.78(10).

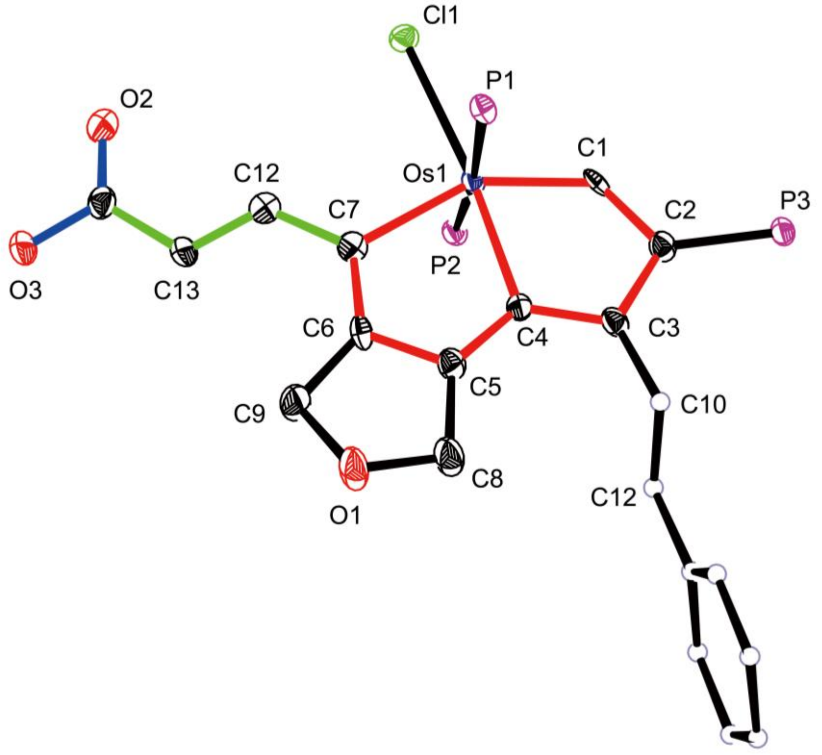

Supplementary Figure 5. X-ray molecular structure for the cation of complex **29** drawn with 50% probability level. The phenyl groups in PPh_3 are omitted for clarity. Selected bond lengths [Å] and angles [°]: Os1–C1 1.827(4), C2–C1 1.400(6), C2–C3 1.435(6), C4–C3 1.415(6), C4–C5 1.393(6), C6–C5 1.399(6), C7–C6 1.400(6), Os1–C7 2.086(4), Os1–C4 2.114(4), C12–C13 1.349(6), C2–C1–Os1 131.9(3), C1–C2–C3 107.1(4), C4–C3–C2110.6(4), C3–C4–Os1 117.7(3), C1–Os1–C4 72.62(16), C5–C4–Os1 115.9(3), C4–C5–C6 115.0(4), C5–C6–C7 116.1(4), C6–C7– Os1 116.0(3), C7–Os1–C4 76.66(15).

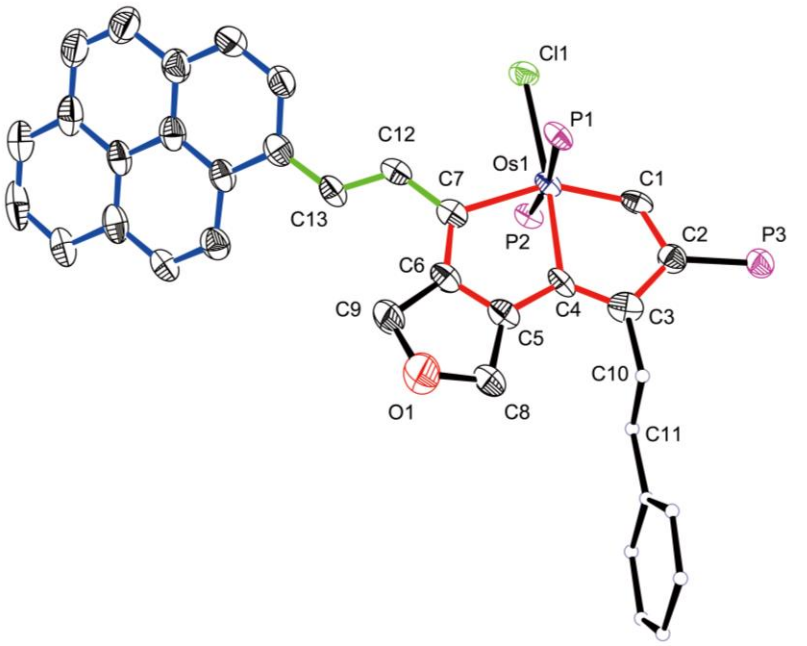

Supplementary Figure 6. X-ray molecular structure for the cation of complex **30'** drawn with 50% probability level. The phenyl groups in PPh_3 are omitted for clarity. Selected bond lengths [Å] and angles [°]: Os1–C1 1.835(6), C2–C1 1.390(8), C2–C3 1.432(8), C4–C3 1.379(8), C5–C4 1.389(8), C5–C6 1.376(8), C7–C6 1.411(8), Os1–C7 2.090(5), Os1–C4 2.124(6), C12–C13 1.353(8), C2–C1–Os1 131.2(4), C1–C2–C3 107.4(5), C4–C3–C2 111.1(5), C3–C4–Os1 118.1(4), C1– Os1–C4 72.2(2), C5– C4–Os1 114.5(4), C6–C5–C4 117.1(5), C5–C6–C7 115.8(5), C6–C7 –Os1 115.6(4), C7–Os1–C4 77.0(2).

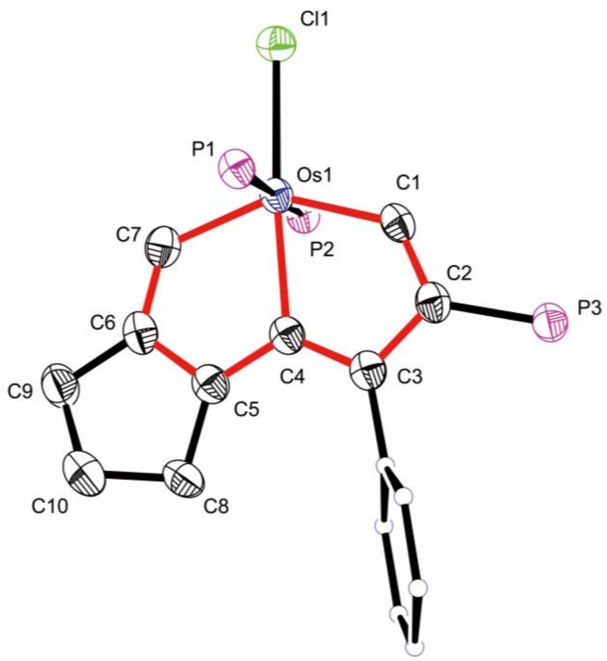

Supplementary Figure 7. X-ray molecular structure for the cation of complex **37** drawn with 50% probability level. The phenyl groups in PPh_3 are omitted for clarity. Selected bond lengths [Å] and angles [°]: Os1–C1 1.963(3), C2–C1 1.363(5), C3–C2 1.432(5), C3–C4 1.399(5), C5–C4 1.408(5), C6–C5 1.391(5), C6–C7 1.373(5), Os1–C7 1.950(4), Os1–C4 2.176(3), C2–C1–Os1 128.4(3), C1–C2–C3 111.2(3), C4–C3–C2 110.4(3), C3–C4–Os1 119.0(3), C1–Os1–C4 71.06(14), C5– C4–Os1 116.3(3), C6–C5–C4 112.9(3), C7–C6–C5 111.2(3), C6–C7–Os1 127.4(3), C7–Os1– C4 72.17(14).

Supplementary Table 1. Crystal data and structure refinement of 1a, 2a, 3, and 15.

	**1a**	**2a**	**3**	**15**
Empirical formula	$C_{71}H_{57}Cl_2OOsP_3$	$C_{71}H_{57}Cl_2OOsP_3$	$C_{79}H_{63}Cl_2OOsP_3$	$C_{79}H_{63}Cl_2O_2OsP_3$
Mol. weight	1280.17	1280.17	1382.3	1398.3
Temperature [K]	99.9(5)	100.0(2)	100.00(10)	100.0(3)
Crystal system	triclinic	triclinic	triclinic	triclinic
Space group	P-1	P-1	P-1	P-1
a [Å]	12.8490(2)	14.2651(4)	11.9760(3)	13.34580(10)
b [Å]	13.2616(2)	14.2916(4)	13.0064(4)	16.61390(10)
c [Å]	22.4264(3)	19.2757(4)	23.0977(6)	17.70430(10)
α [°]	81.5250(10)	91.372(2)	93.774(2)	78.9380(10)
β [°]	78.0430(10)	107.575(2)	100.212(2)	72.0740(10)
γ [°]	61.291(2)	112.085(3)	105.623(3)	71.6370(10)
V [Å^3]	3273.83(10)	3429.15(17)	3385.42(17)	3525.12(5)
Z	2	2	2	2
ρ_{calcd} [g cm^{-3}]	1.299	1.24	1.356	1.317
μ [mm^{-1}]	5.428	5.182	5.291	5.099
F(000)	1292	1292	1400	1416
Crystal size [mm^3]	0.1 × 0.1 × 0.1	0.15 × 0.15 × 0.13	0.2 × 0.2 × 0.1	0.15 × 0.14 × 0.13
Radiation	CuKα (λ = 1.54184)	CuKα (λ = 1.54184)	CuKα (λ = 1.54184)	CuKα (λ = 1.54184)
2θ range [°]	7.612 to 124.998	6.756 to 124.998	7.108 to 156.992	5.276 to 124.998
Coll. refl.	34495	34905	45846	75856
Indep. refl.	10435	10940	13978	11250
data/restraints/params	10435/72/703	10940/44/721	13978/36/745	11250/24/785
GOF on F^2	1.055	1.081	1.049	1.057
R_1/wR_2 [$I \geq 2\sigma(I)$]	0.0449/0.1156	0.0535/0.1571	0.0632/0.1591	0.0251/0.0639
R_1/wR_2 (all data)	0.0524/0.1196	0.0615/0.1634	0.0742/0.1730	0.0258/0.0657
Largest peak/hole [e Å-3]	3.62/-2.25	3.35/-1.11	2.39/-3.23	1.06/-1.40

Supplementary Table 2. Crystal data and structure refinement of 29, 30' and 37.

	**29**	**30'**	**37**
Empirical formula	$C_{74}H_{59}Cl_2O_3OsP_3$	$C_{89}H_{67}BClF_4OOsP_3$	$C_{70}H_{58}B_2ClF_8OsP_3$
Mol. weight	1350.22	1557.79	1391.34
Temperature [K]	100.0(3)	100.00(10)	100.0(2)
Crystal system	orthorhombic	triclinic	monoclinic
Space group	Pbca	P-1	I2/a
a [Å]	14.3183(2)	11.9441(2)	26.3388(5)
b [Å]	23.5216(4)	12.8665(2)	20.7254(3)
c [Å]	37.3136(7)	29.3443(3)	25.3639(4)
α [°]	90	98.4380(10)	90
β [°]	90	91.6950(10)	93.938(2)
γ [°]	90	113.7820(10)	90
V [Å^3]	12566.8(4)	4062.33(11)	13813.0(4)
Z	8	2	8
ρ_{calcd} [g cm^{-3}]	1.427	1.274	1.338
μ [mm^{-1}]	5.712	4.247	4.997
F(000)	5456	1576	5584
Crystal size [mm^3]	0.16 × 0.14 × 0.13	0.15 × 0.13 × 0.12	0.14 × 0.13 × 0.12
Radiation	CuKα (λ = 1.54184)	CuKα (λ = 1.54184)	CuKα (λ = 1.54184)
2θ range [°]	7.516 to 124.988	6.12 to 129.986	5.43 to 124.97
Coll. refl.	42888	98716	79161
Indep. refl.	10014	13724	11024
data/restraints/params	10014/0/758	13724/36/875	11024/0/766
GOF on F^2	1.032	1.078	1.07
R_1/wR_2 [$I \geq 2\sigma(I)$]	0.0365/0.0940	0.0566/0.1513	0.0355/0.1008
R_1/wR_2 (all data)	0.0442/0.0976	0.0594/0.1534	0.0413/0.1070
Largest peak/hole [e Å-3]	1.63/-0.52	1.82/-1.90	1.02/-0.81

Supplementary Table 3. The best performance of PSCs based on PM6:Y6 without and with neat ethanol as ETL under 100 mW cm^{-2} AM 1.5 G irradiation.

ETL	V_{oc} (V)	J_{sc} (mA/cm^2)	FF(%)	PCE(%)
w/o	0.76	23.94	57.82	10.47
ethanol	0.75	23.70	62.07	11.02

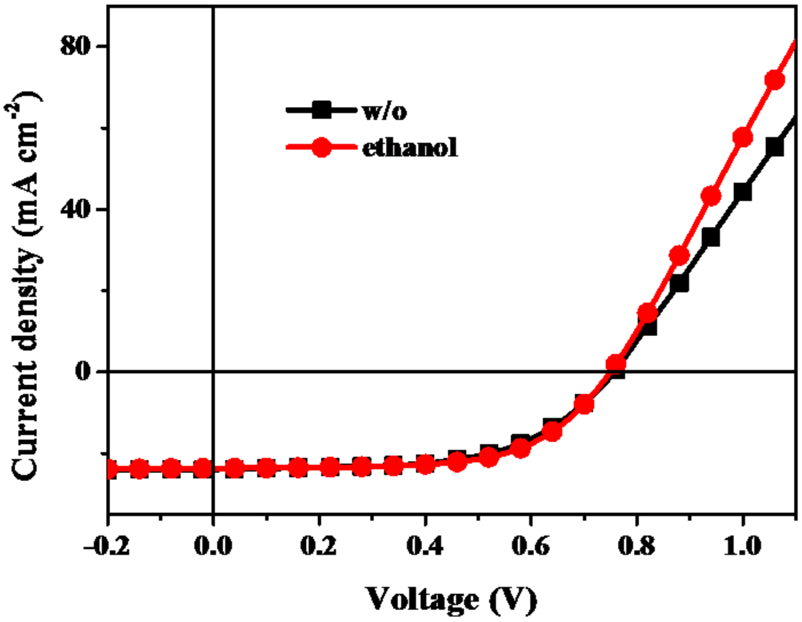

Supplementary Figure 8. The best *J-V* curves of OSCs based on PM6:Y6 without and with neat ethanol as ETL under 100 mW cm^{-2} AM 1.5 G irradiation.

Supplementary Table 4. Mobility of electron-only (μ_e) devices based on PM6:Y6 with PDINO or complex **30** in dark.

ETL	Thickness (nm)	Active layer	Thickness (nm)	μ_e (cm^2 V^{-1} S^{-1})
PDINO	7.519±0.062	PM6:Y6	110	1.7×10^{-4}
Complex **30**	6.972±0.328		110	5.7×10^{-4}

Supplementary Table 5. The performance based on PTB7-Th:$PC_{71}BM$ with PDINO/complex **30** as ETL respectively under 100 mW cm^{-2} AM 1.5 G irradiation.

ETL	V_{oc} (V)	J_{sc} (mA/cm^2)	FF (%)	PCE (%)	Jcal[a]
PDINO	0.80	15.69	68.30	8.54<br>(8.32 ±0.22)[b]	15.22
Complex **30**	0.80	16.02	71.04	9.15<br>(8.93 ±0.22)	15.74

[a]The calculated J_{sc} values from EQE curves;

[b]Average value ± standard deviation were calculated from the statistics of 20 different devices

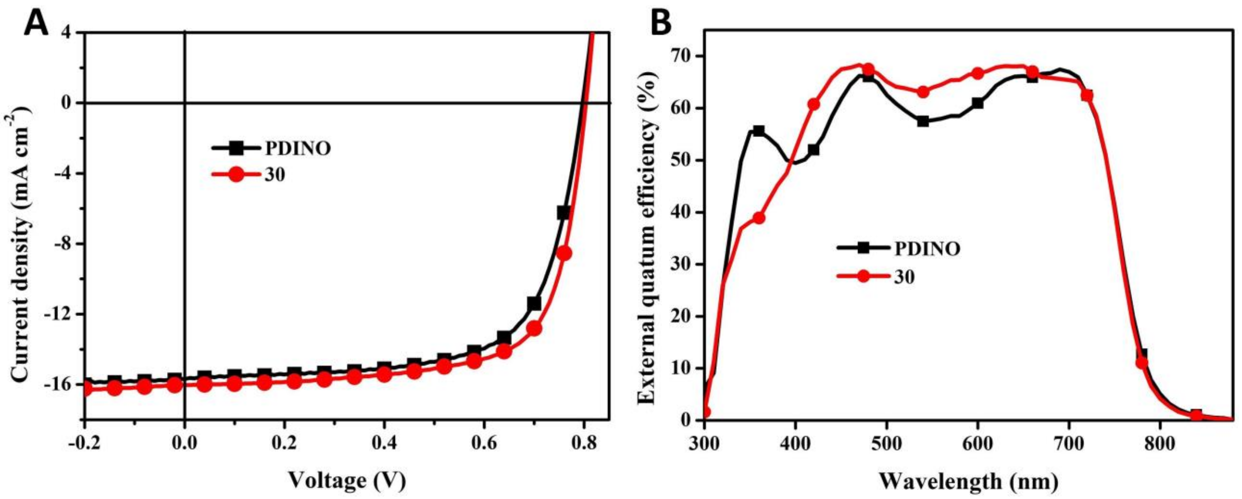

Supplementary Figure 9. The (A) *J-V* and (B) EQE curves of OSCs based on PTB7-Th:$PC_{71}BM$ with PDINO/complex **30** as ETL respectively under 100 mW cm^{-2} AM 1.5 G irradiation.

Supplementary Table 6. The performance based on PTB7-Th:IEICO-4F with PDINO/complex **30** as ETL respectively under 100 mW cm^{-2} AM 1.5 G irradiation.

ETL	V_{oc} (V)	J_{sc} (mA/cm^2)	FF (%)	PCE (%)	Jcal[a]
PDINO	0.72	20.40	62.49	9.17 (8.82±0.35)[b]	20.18
Complex **30**	0.74	21.72	65.22	10.42 (10.09±0.33)	21.35

[a]The calculated J_{sc} values from EQE curves;

[b]Average value ±standard deviation were calculated from the statistics of 20 different devices

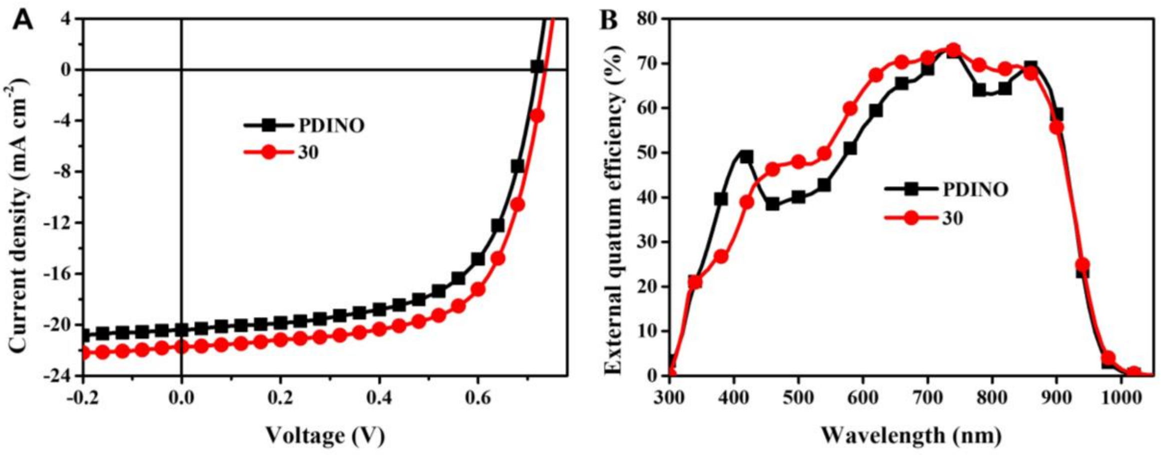

Supplementary Figure 10. The (A) *J-V* and (B) EQE curves of OSCs based on PTB7-Th:IEICO-4F with PDINO/complex **30** as ETL respectively under 100 mW cm^{-2} AM 1.5 G irradiation.

Supplementary Table 7. TD-DFT calculation results of absorption peaks of different compounds

Compound	λ_{abs}/nm Calc.(Exp.)	Excitation	f	Percentage
**3**	557 (572)	HOMO to LUMO	0.8250	96.7%
**30**	626 (634)	HOMO to LUMO	1.1179	98.5%

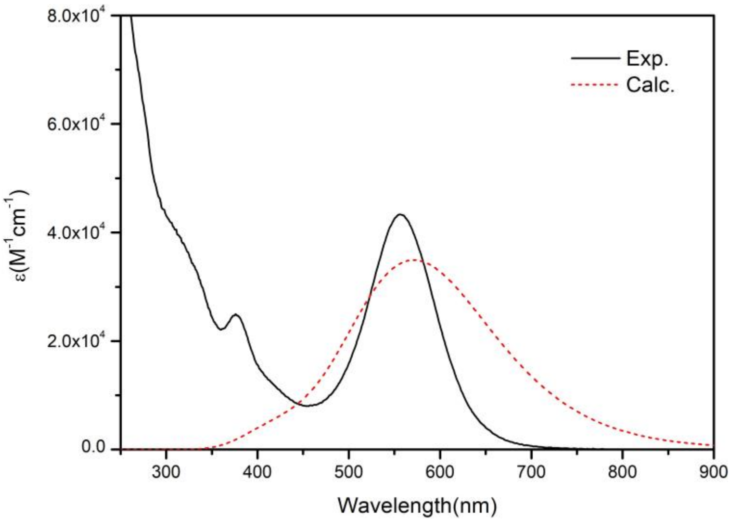

Supplementary Figure 11. UV-vis–NIR absorption spectra of carbolong complexes **3** and its modeling fitted one.

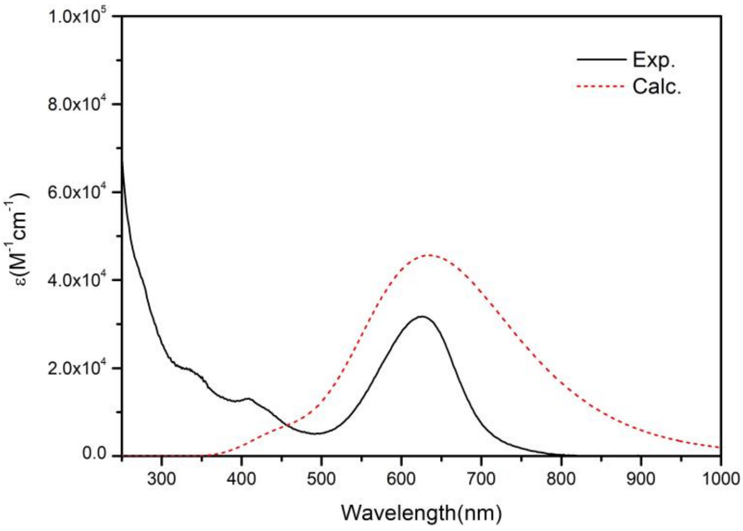

Supplementary Figure 12. UV-vis–NIR absorption spectra of carbolong complexes **30** and its modeling fitted one.

Supplementary Table 8. DFT calculation results of HOMO and LUMO

Compound	LUMO (eV)	HOMO (eV)	HOMO-LUMO gap (eV)
**1a**	-4.63	-7.56	2.93
**3**	-4.58	-7.02	2.44
**30**	-4.49	-6.63	2.14

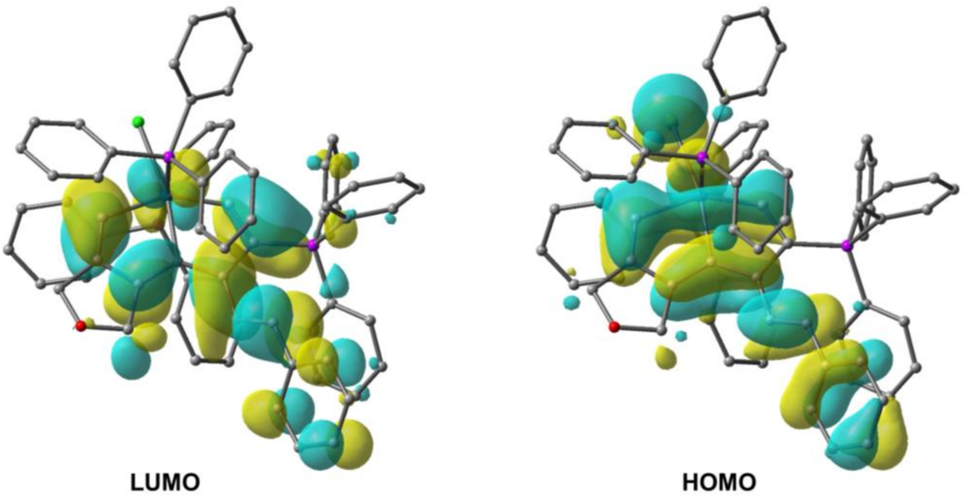

Supplementary Figure 13. Selected orbitals of complex **1a** showed above (isovalue = 0.02).

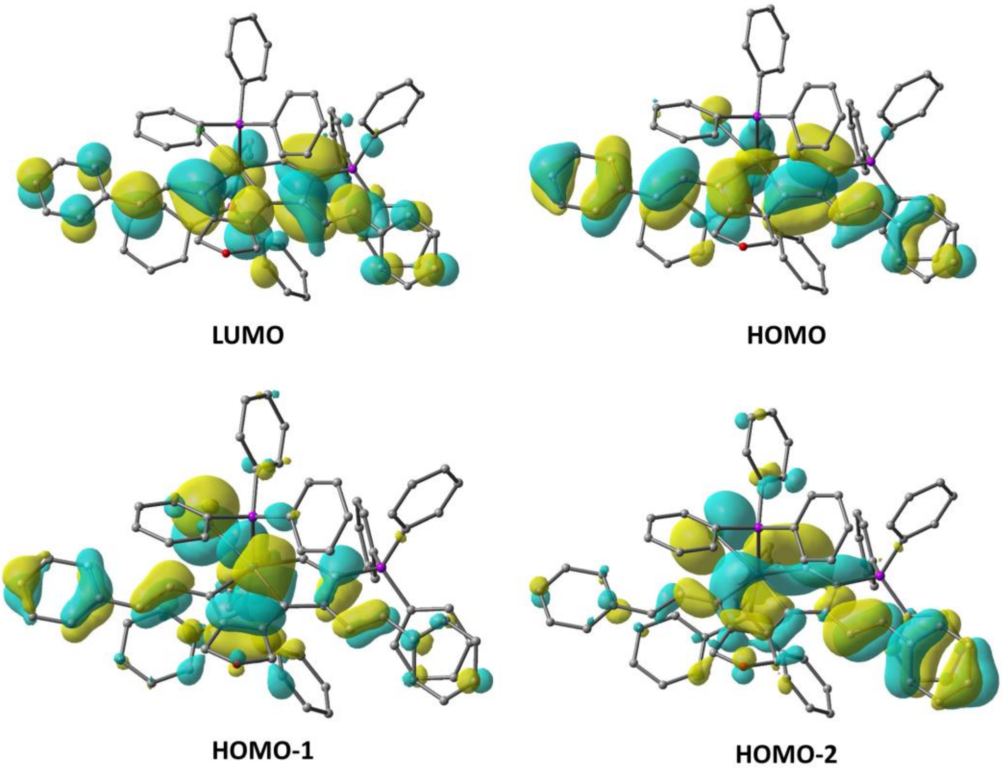

Supplementary Figure 14. Selected orbitals of complex **3** showed above (isovalue = 0.02).

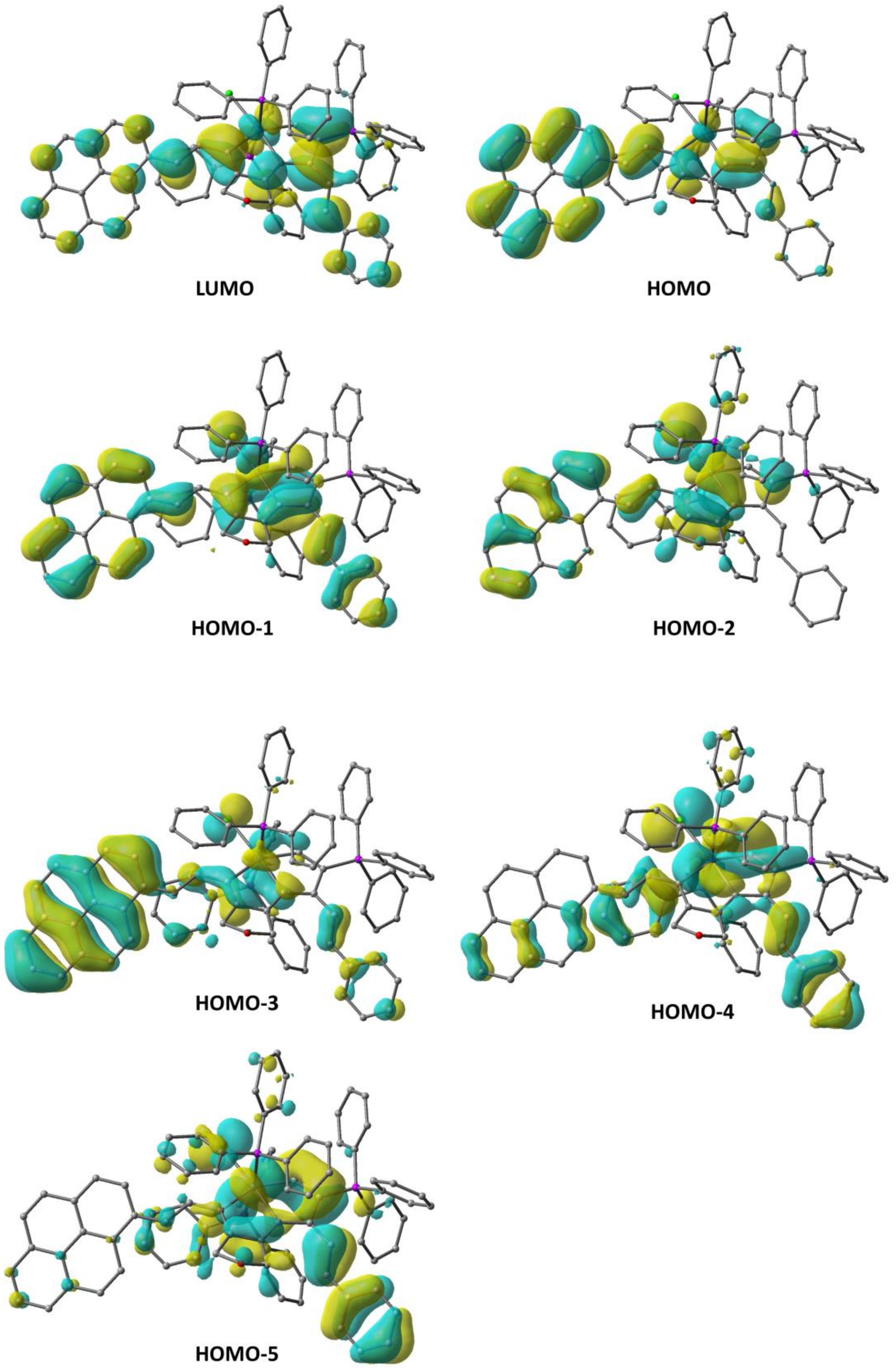

Supplementary Figure 15. Selected orbitals of complex **30** showed above (isovalue = 0.02).

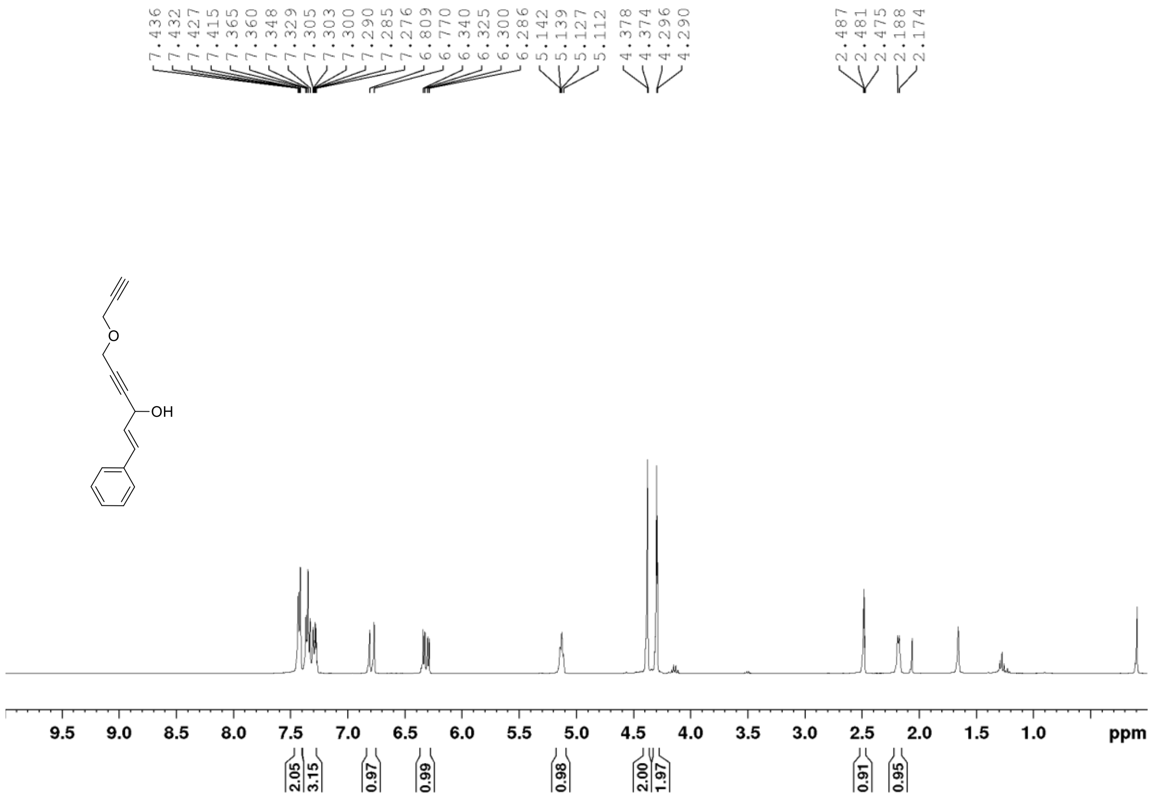

Supplementary Figure 16. The ^{1}H NMR (400.1 MHz, $CDCl_3$) spectrum of complex **S1-1** at room temperature.

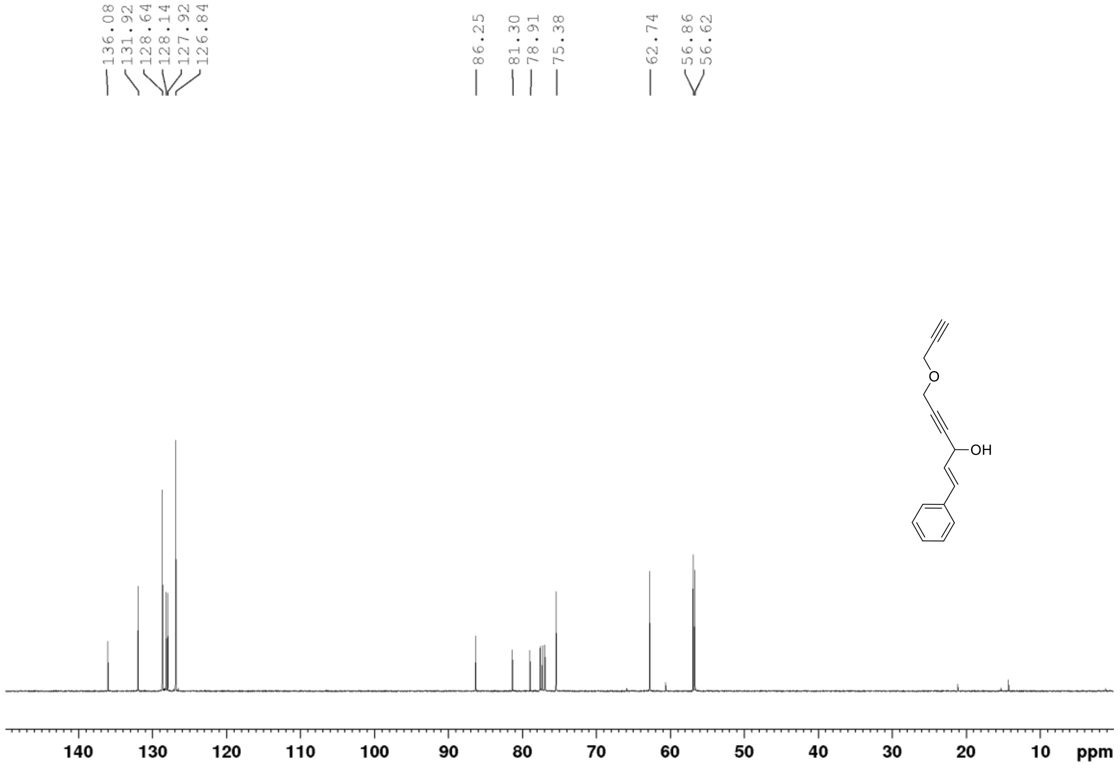

Supplementary Figure 17. The ^{13}C NMR (100.6 MHz, $CDCl_3$) spectrum of complex **S1-1** at room temperature.

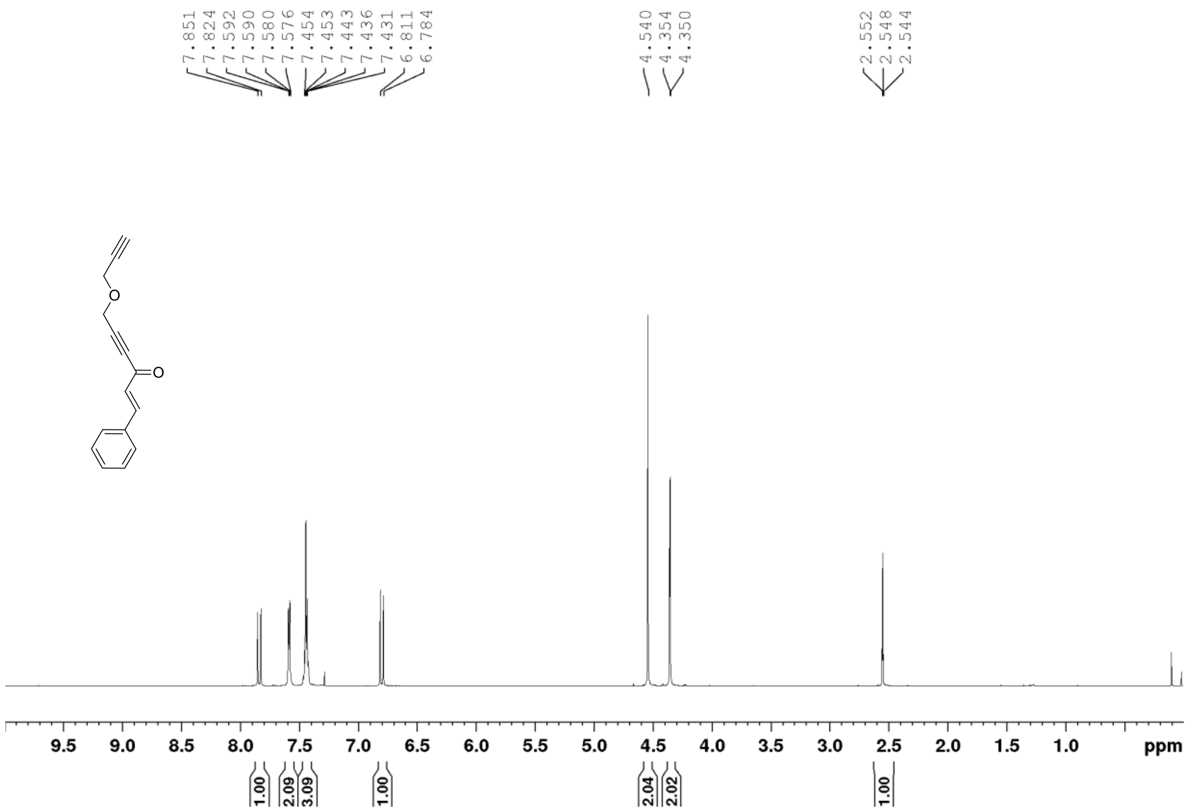

Supplementary Figure 18. The ^{1}H NMR (600.1 MHz, $CDCl_3$) spectrum of complex **S1-2** at room temperature.

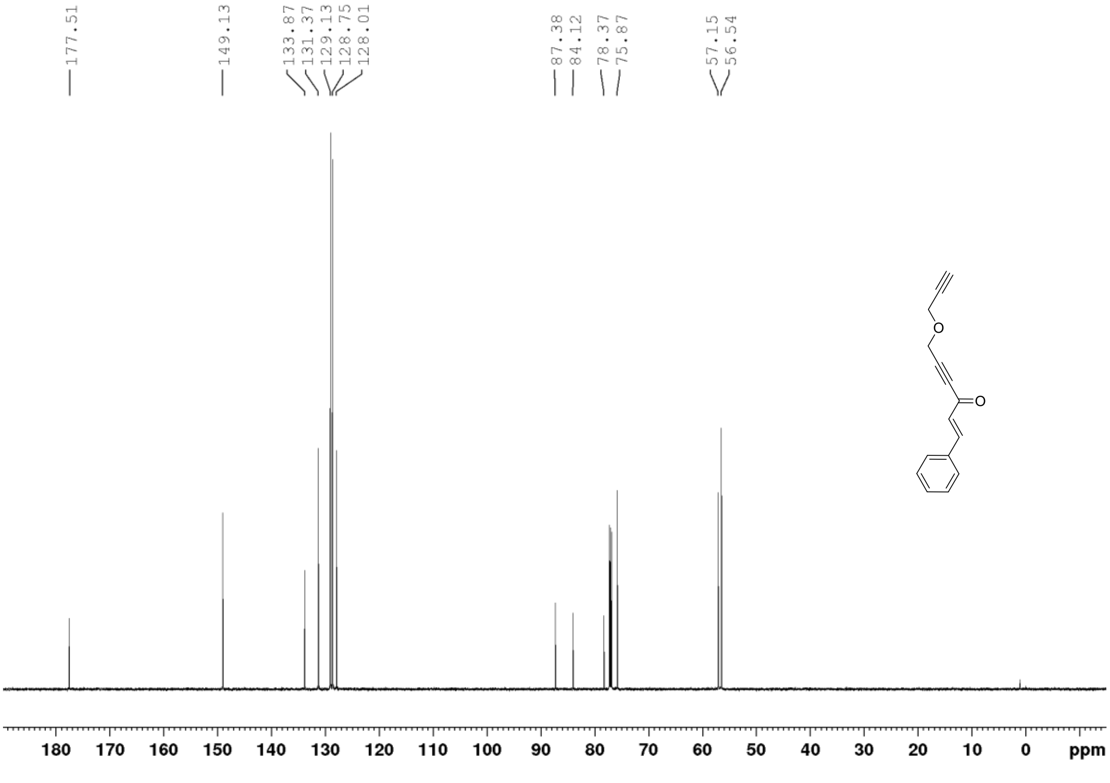

Supplementary Figure 19. The ^{13}C NMR (150.9 MHz, $CDCl_3$) spectrum of complex **S1-2** at room temperature.

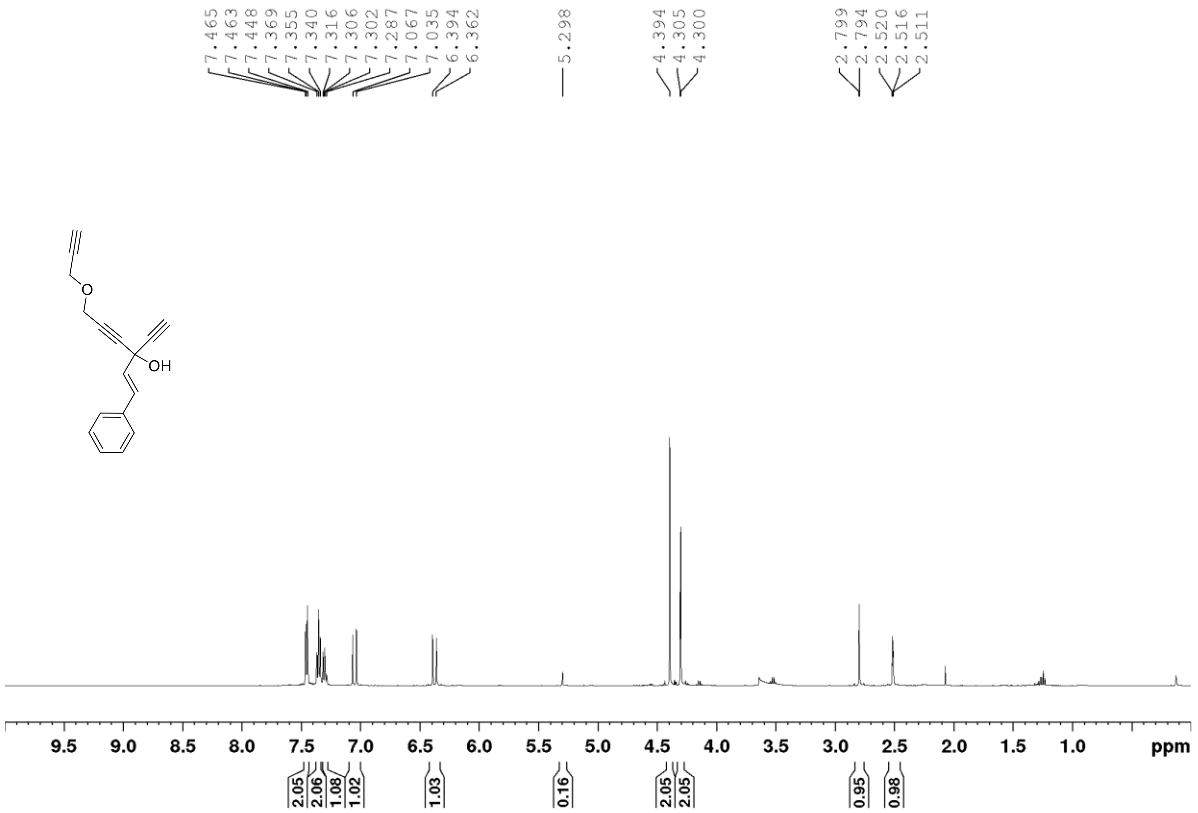

Supplementary Figure 20. The ^{1}H NMR (500.2 MHz, $CDCl_3$) spectrum of complex **L1** at room temperature.

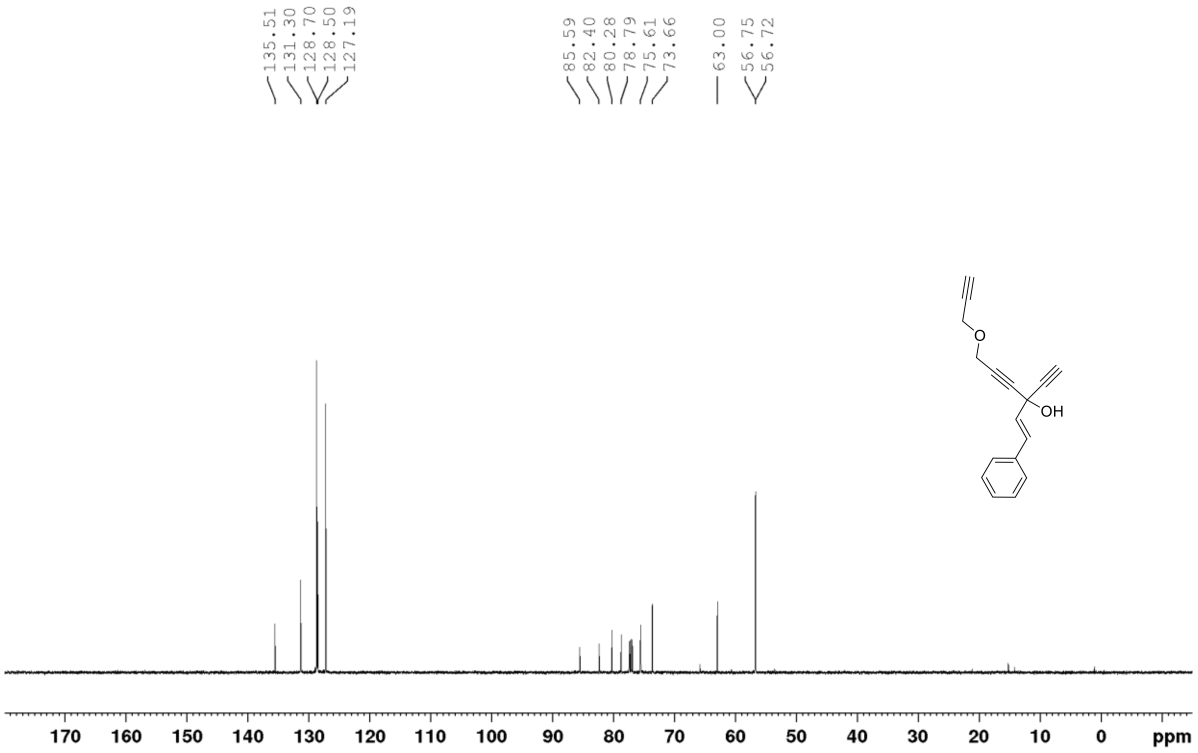

Supplementary Figure 21. The ^{13}C NMR (125.8 MHz, $CDCl_3$) spectrum of complex **L1** at room temperature.

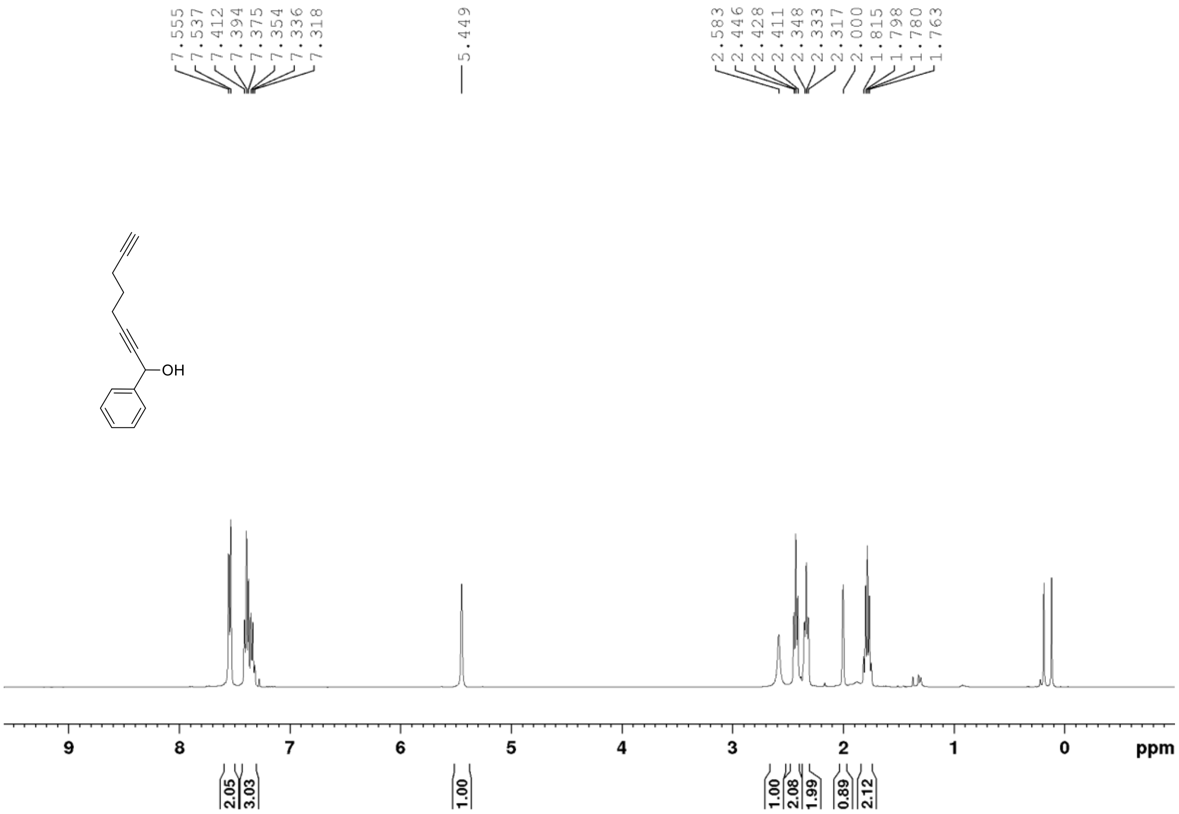

Supplementary Figure 22. The ^{1}H NMR (400.1 MHz, $CDCl_3$) spectrum of complex **S2-1** at room temperature.

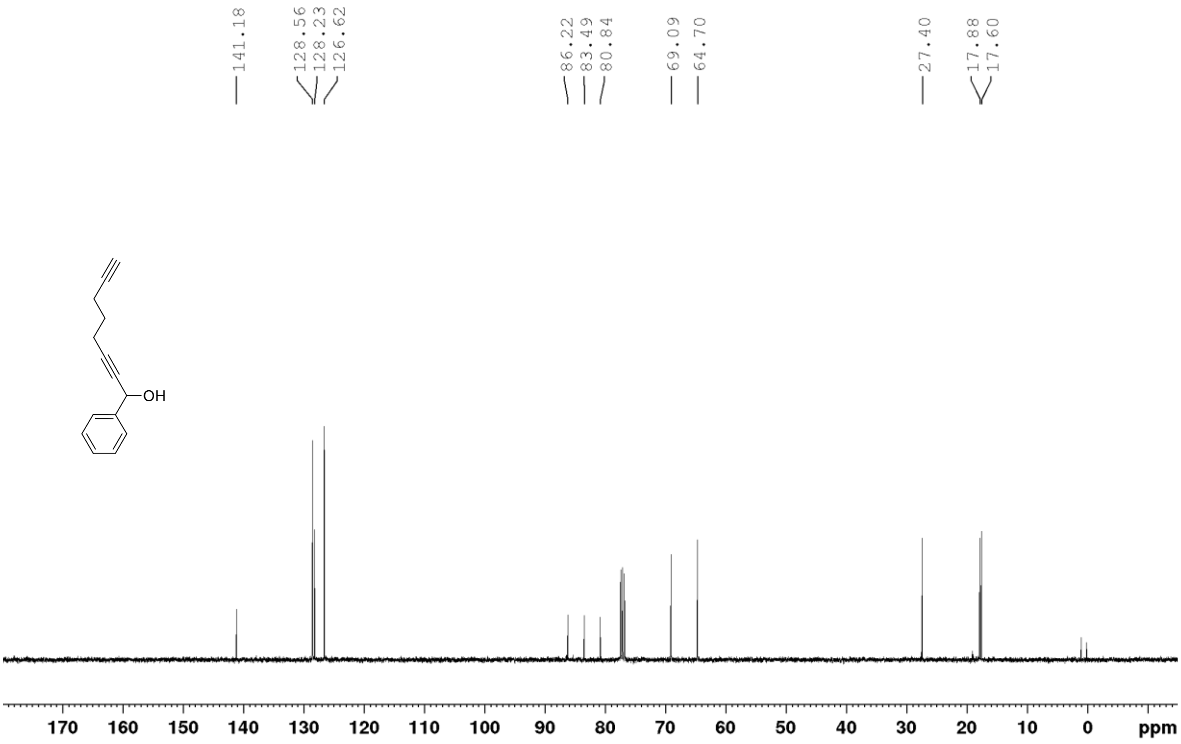

Supplementary Figure 23. The ^{13}C NMR (100.6 MHz, $CDCl_3$) spectrum of complex **S2-1** at room temperature.

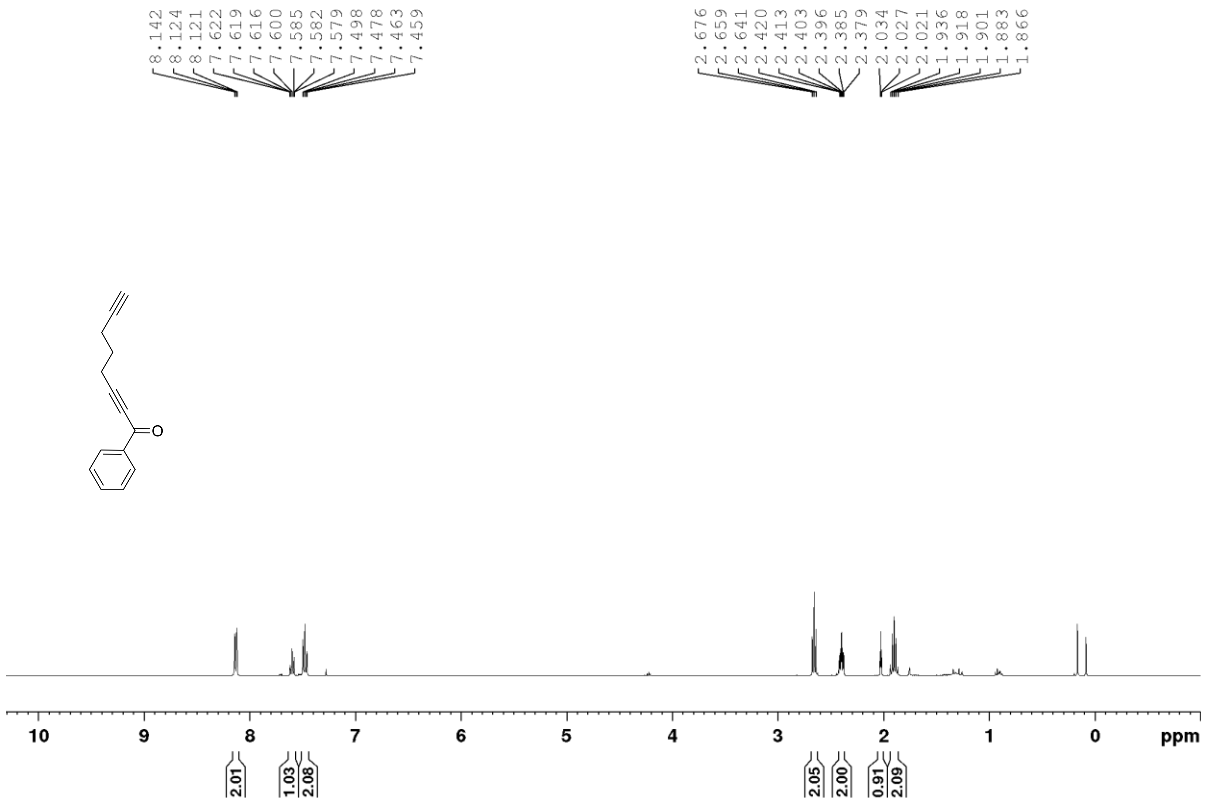

Supplementary Figure 24. The ^{1}H NMR (400.1 MHz, $CDCl_3$) spectrum of complex **S2-2** at room temperature.

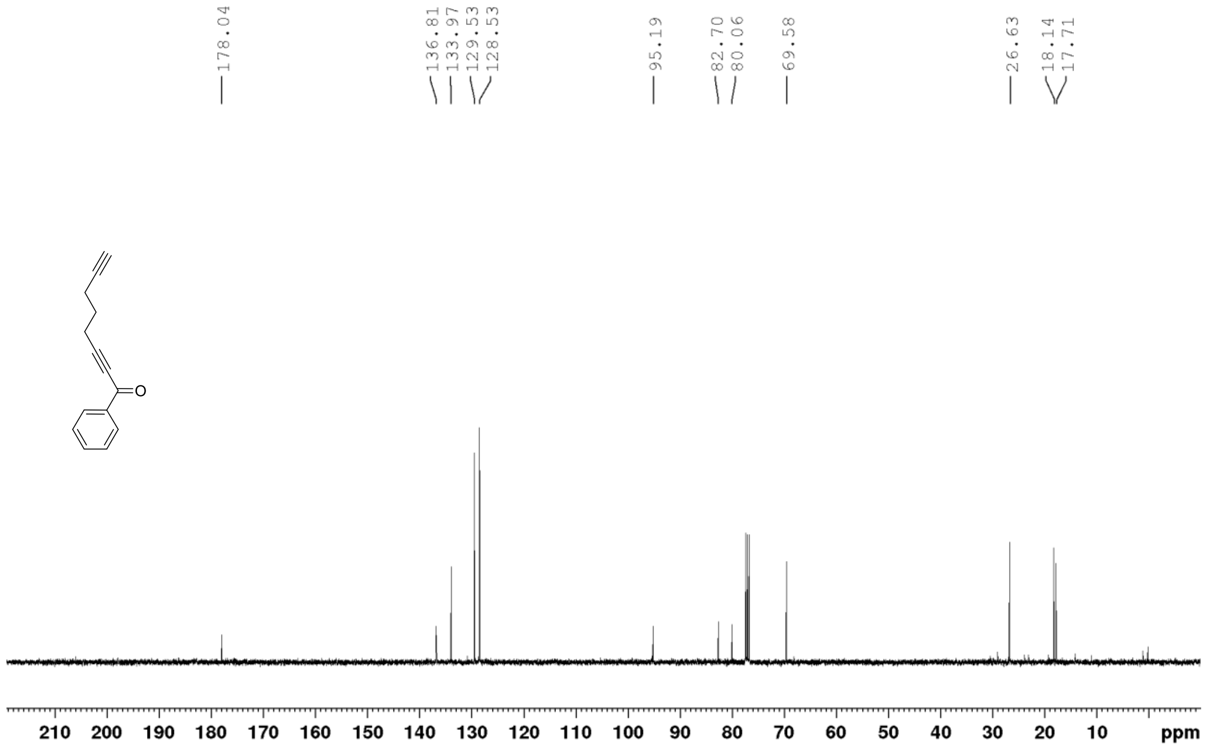

Supplementary Figure 25. The ^{13}C NMR (100.6 MHz, $CDCl_3$) spectrum of complex **S2-2** at room temperature.

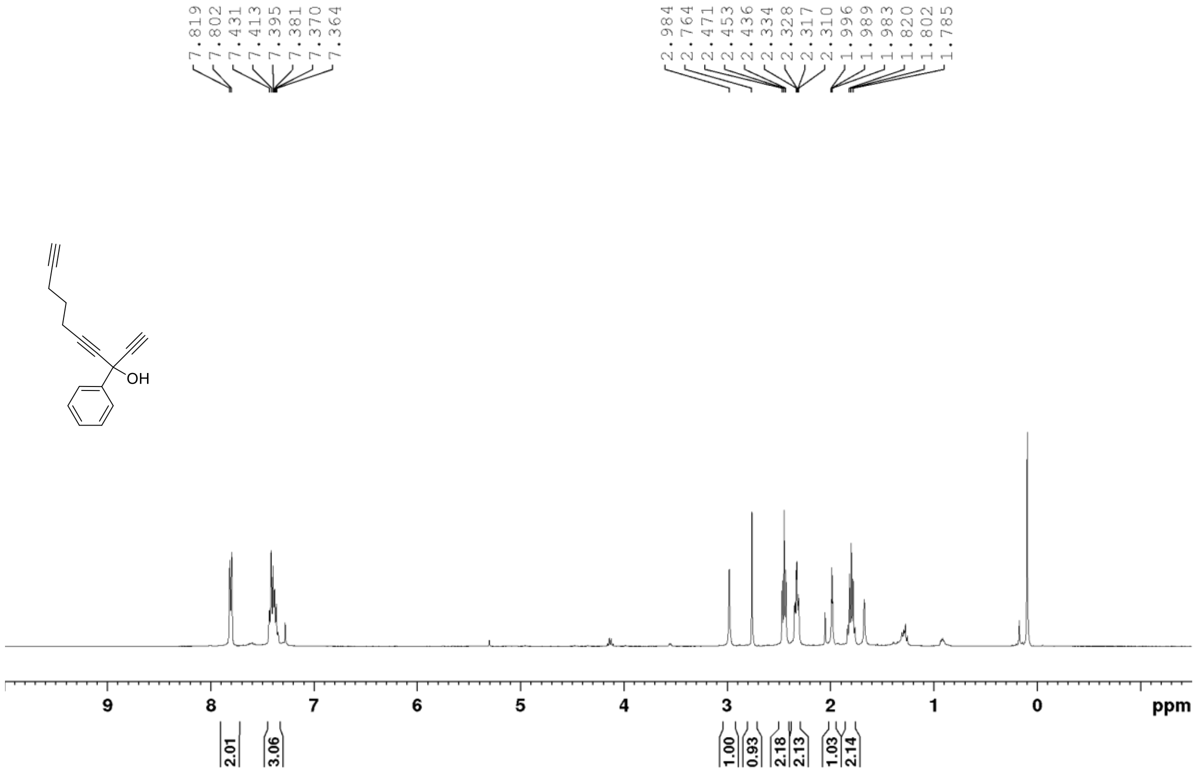

Supplementary Figure 26. The ^{1}H NMR (400.1 MHz, $CDCl_3$) spectrum of complex **L2** at room temperature.

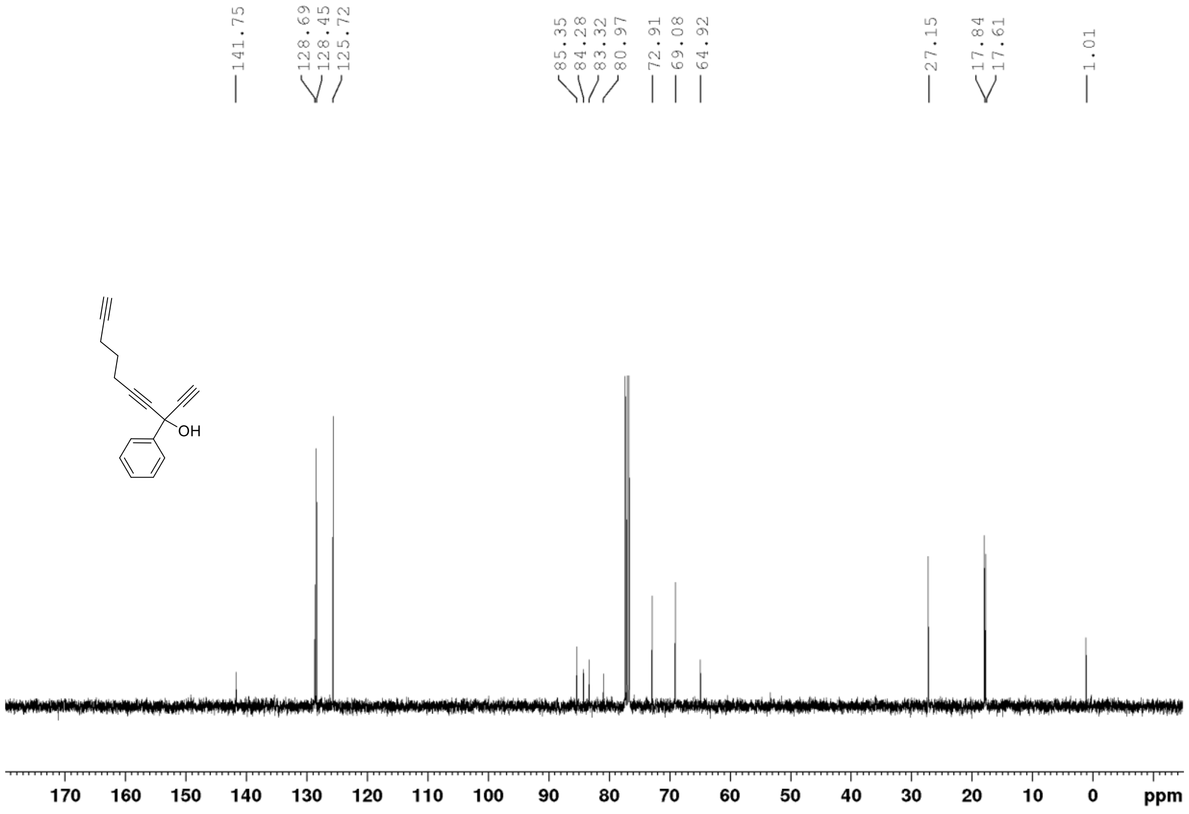

Supplementary Figure 27. The ^{13}C NMR (100.6 MHz, $CDCl_3$) spectrum of complex **L2** at room temperature.

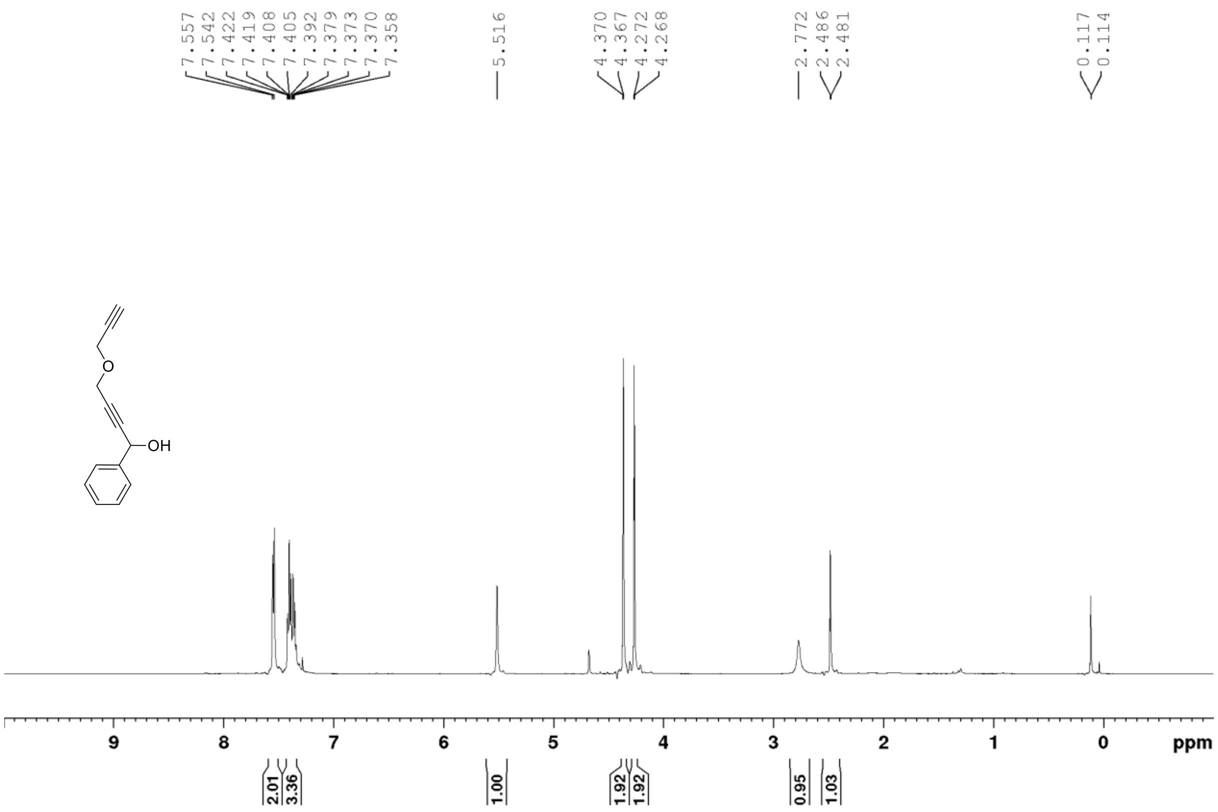

Supplementary Figure 28. The ^{1}H NMR (500.2 MHz, $CDCl_3$) spectrum of complex **S3-1** at room temperature.

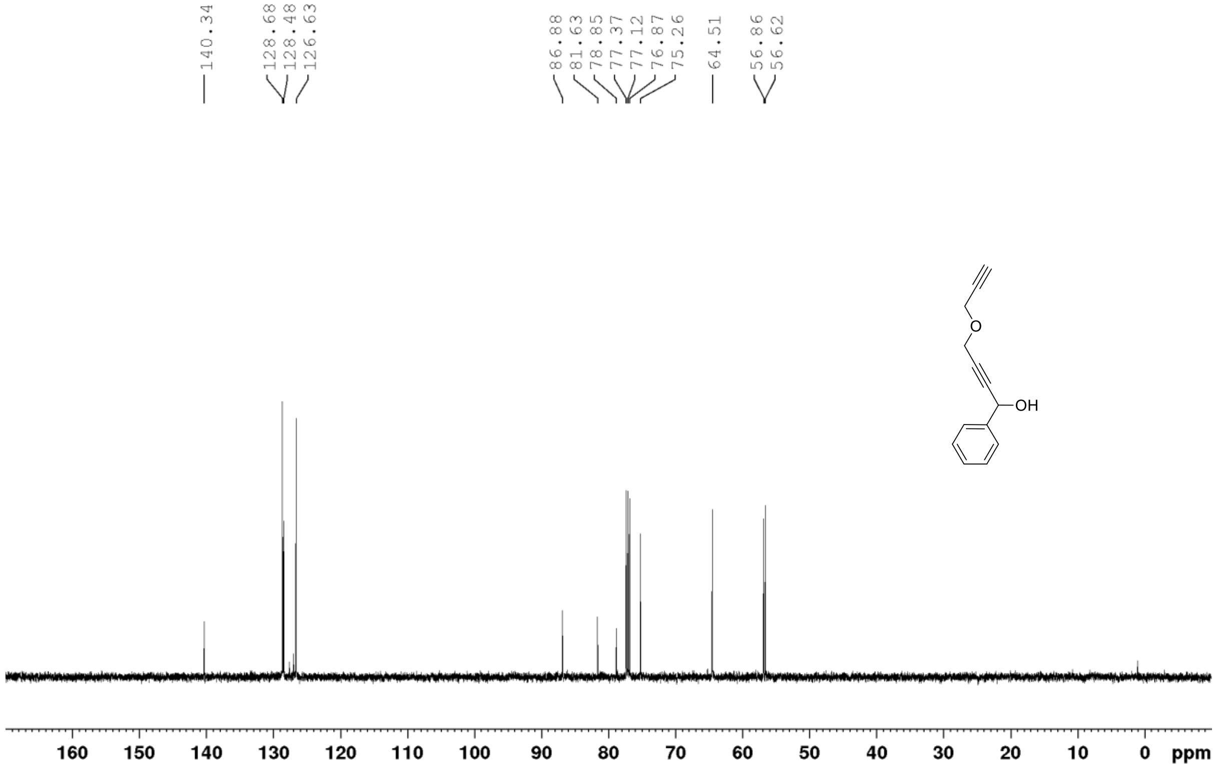

Supplementary Figure 29. The ^{13}C NMR (125.8 MHz, $CDCl_3$) spectrum of complex **S3-1** at room temperature.

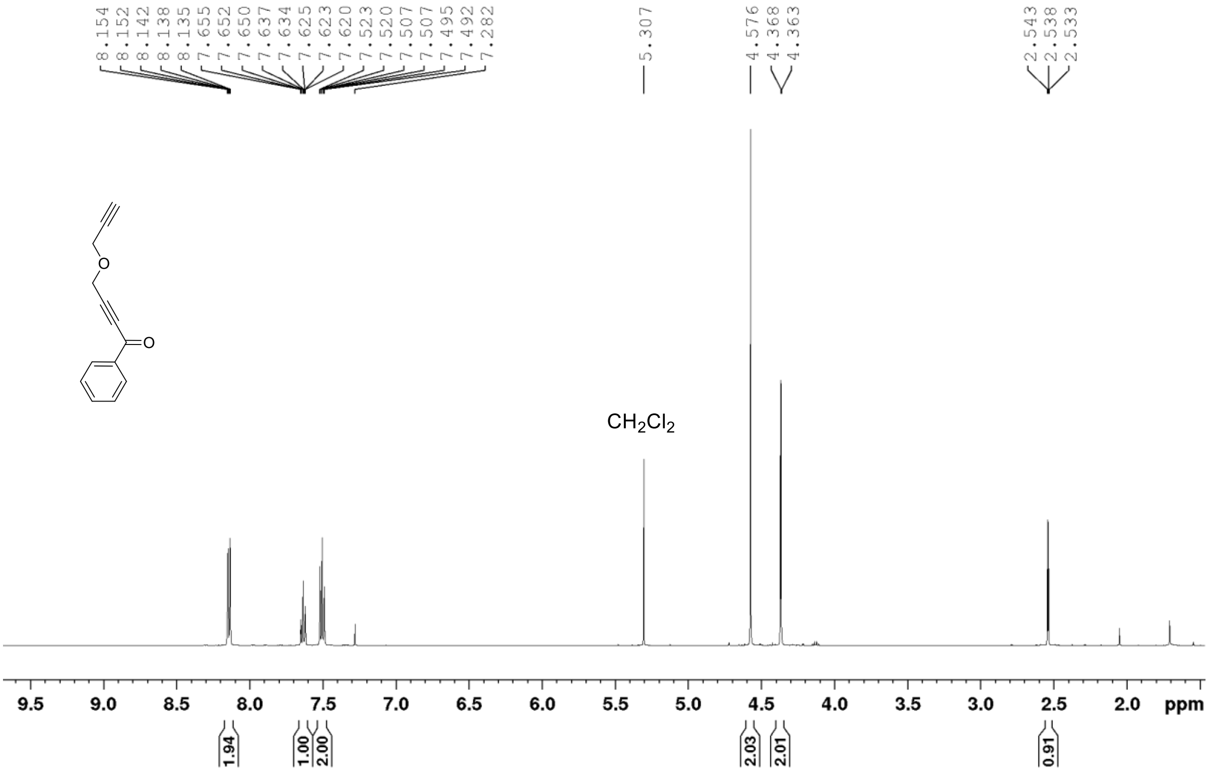

Supplementary Figure 30. The ^{1}H NMR (500.2 MHz, $CDCl_3$) spectrum of complex **S3-2** at room temperature.

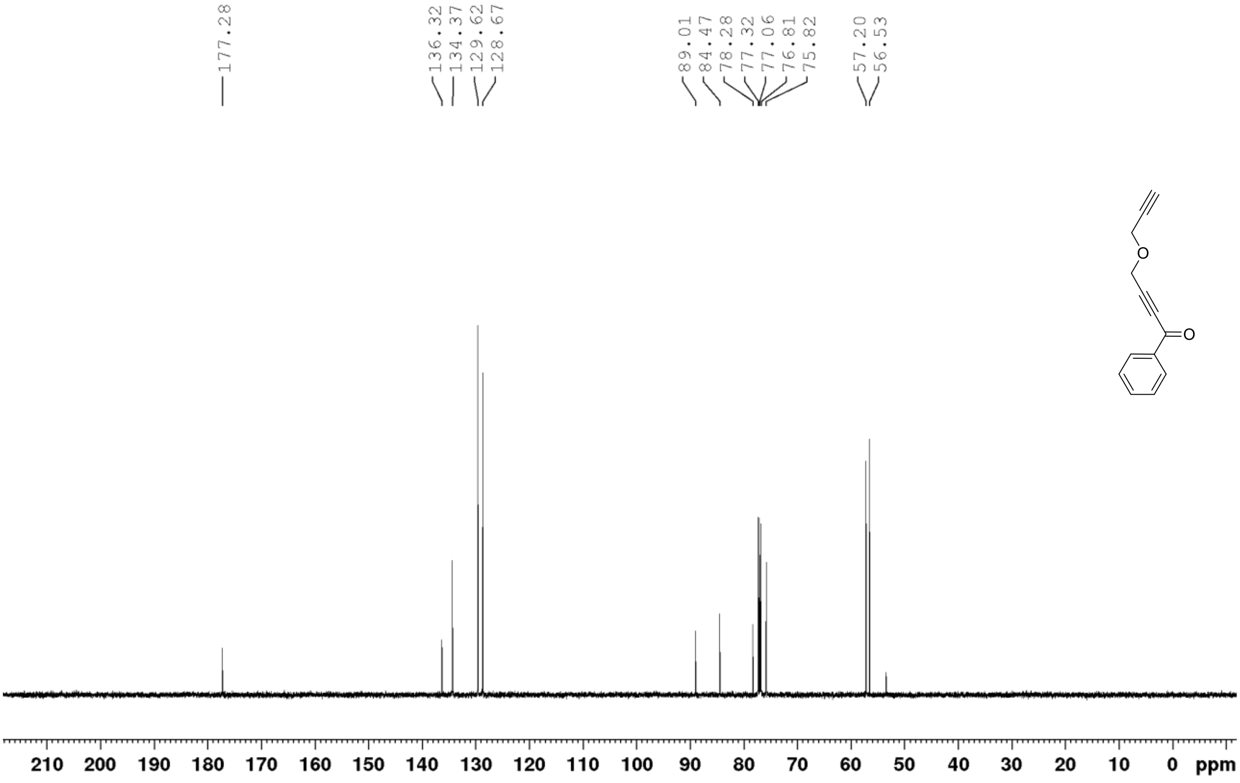

Supplementary Figure 31. The ^{13}C NMR (125.8 MHz, $CDCl_3$) spectrum of complex **S3-2** at room temperature.

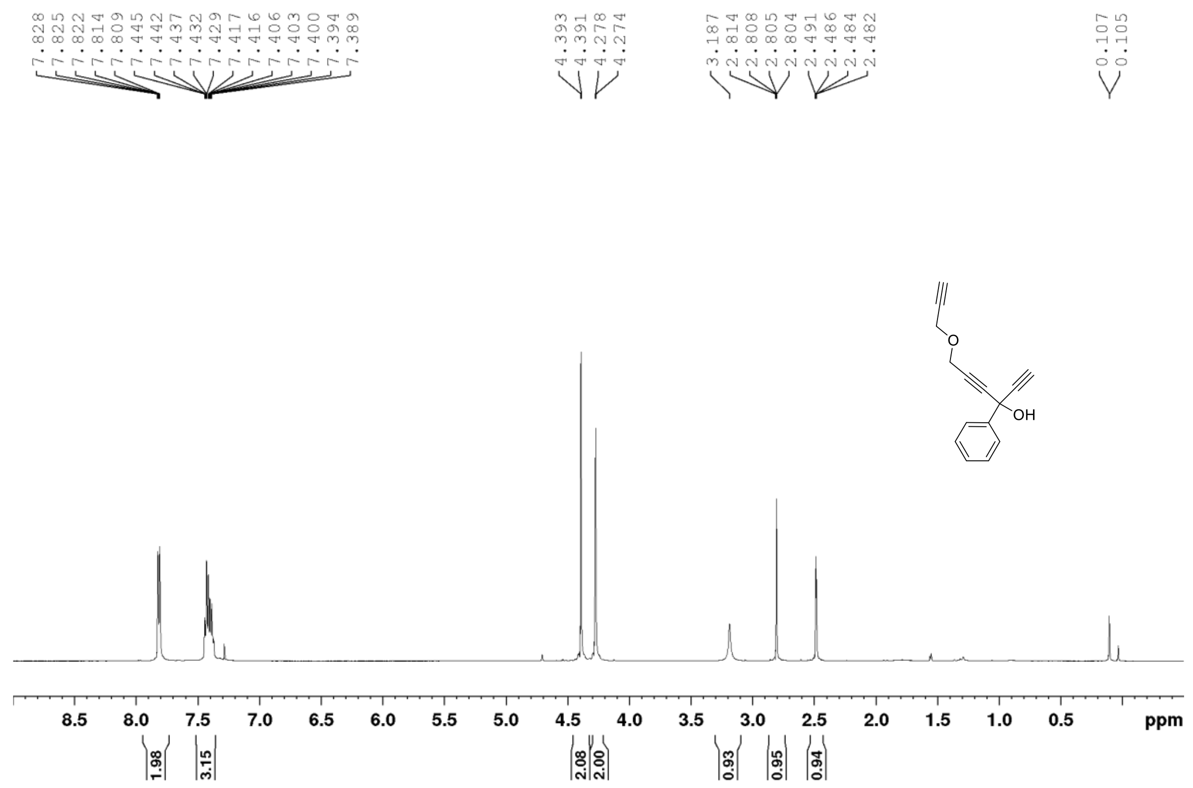

Supplementary Figure 32. The ^{1}H NMR (500.2 MHz, $CDCl_3$) spectrum of complex **L3** at room temperature.

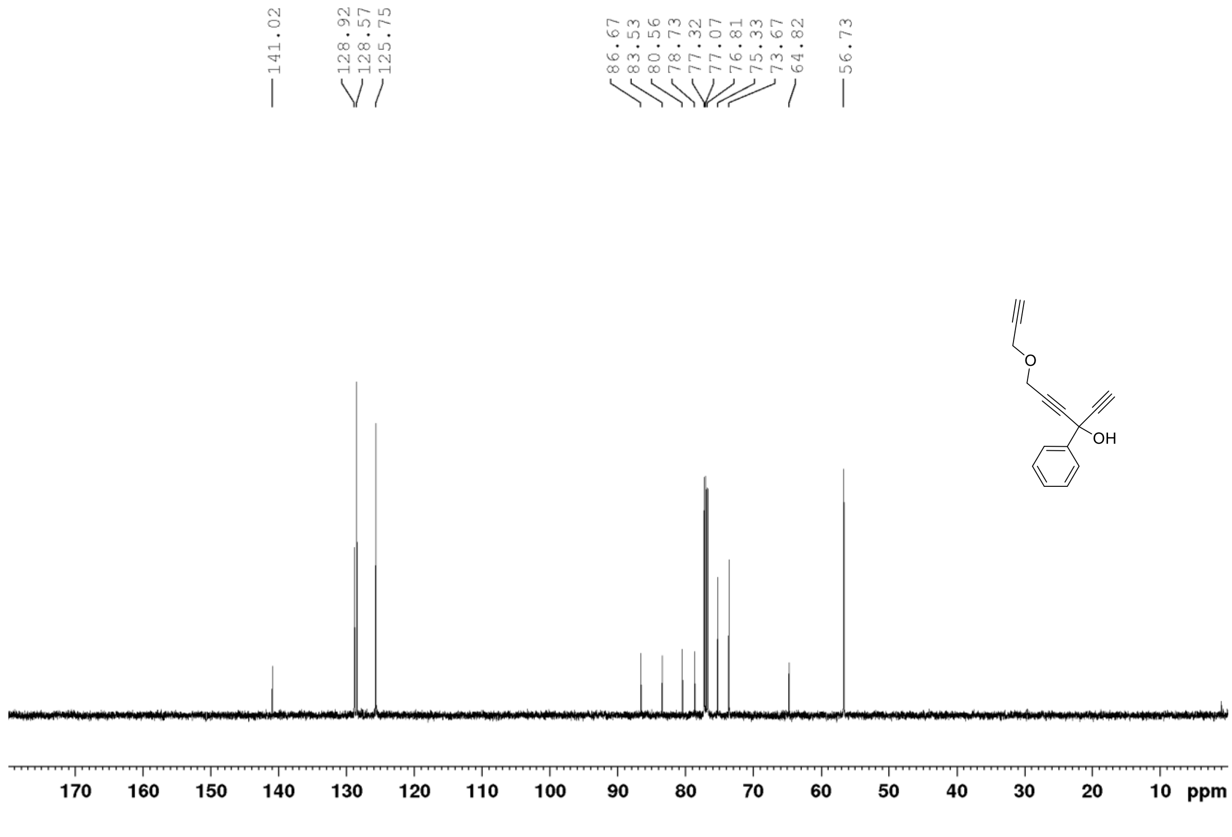

Supplementary Figure 33. The ^{13}C NMR (125.8 MHz, $CDCl_3$) spectrum of complex **L3** at room temperature.

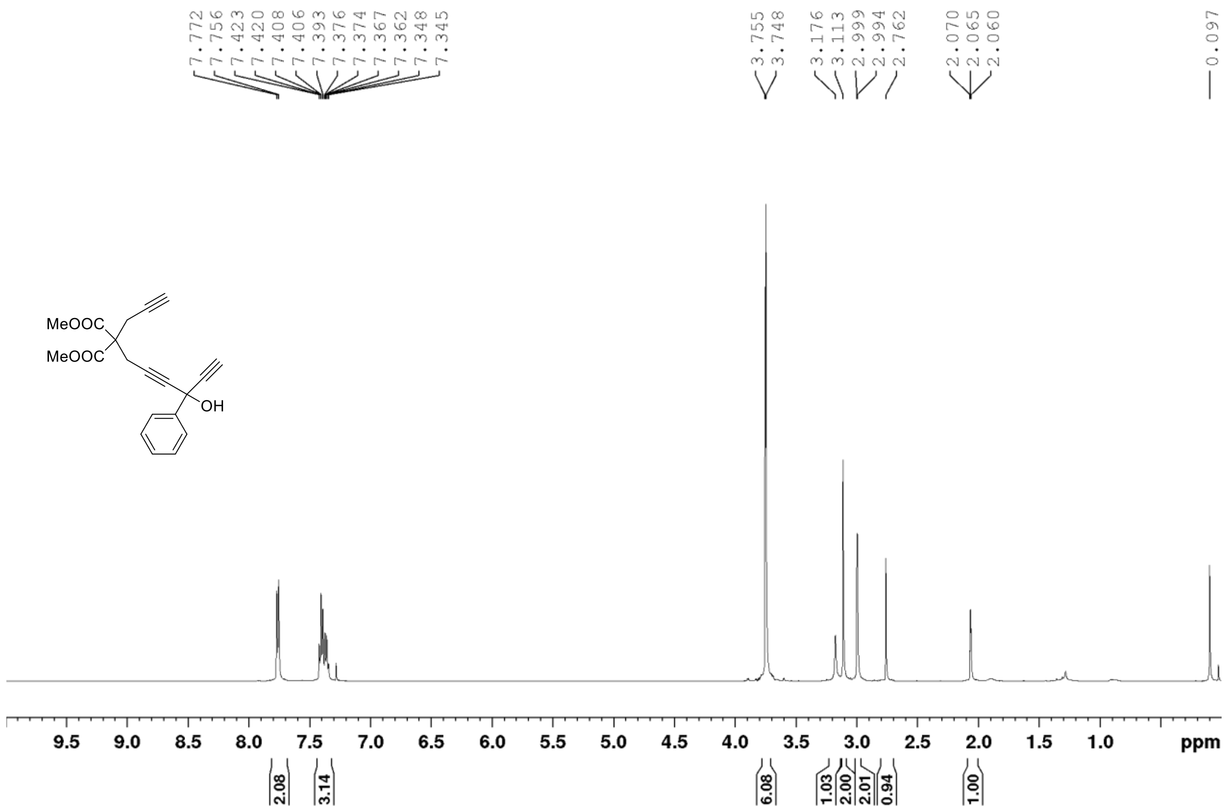

Supplementary Figure 34. The ^{1}H NMR (500.2 MHz, $CDCl_3$) spectrum of complex **L4** at room temperature.

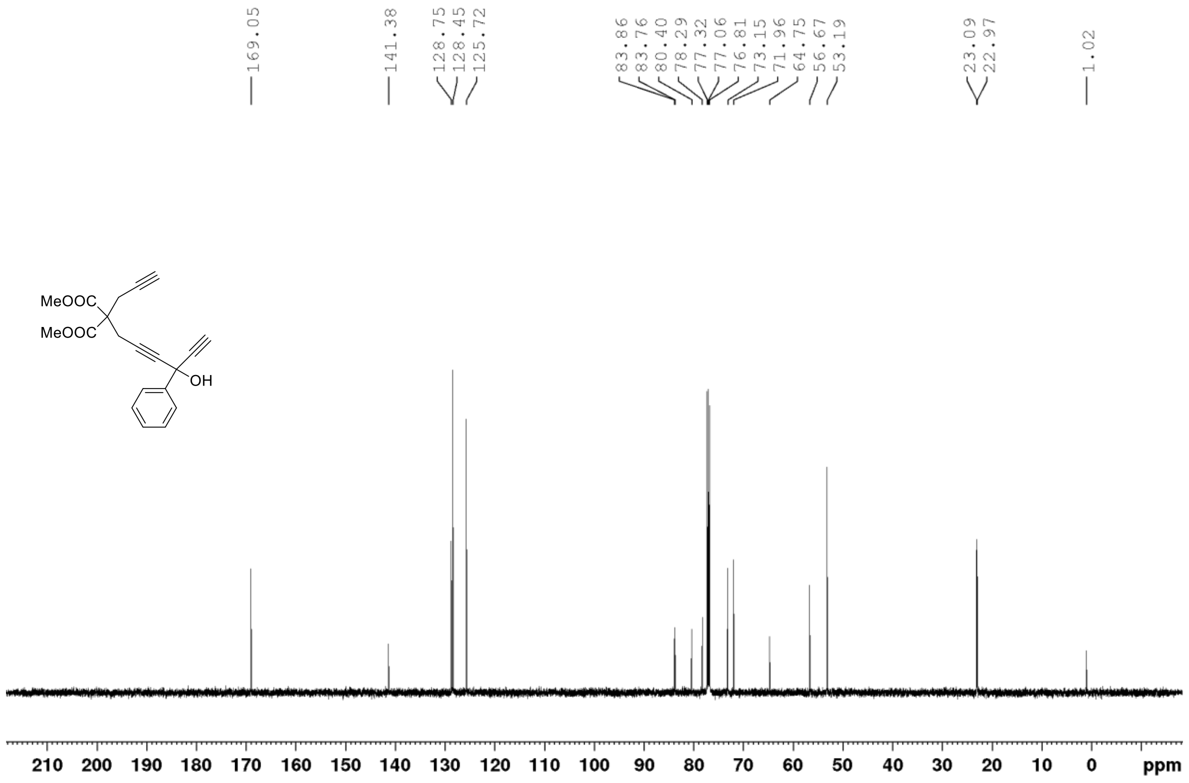

Supplementary Figure 35. The ^{13}C NMR (125.8 MHz, $CDCl_3$) spectrum of complex **L4** at room temperature.

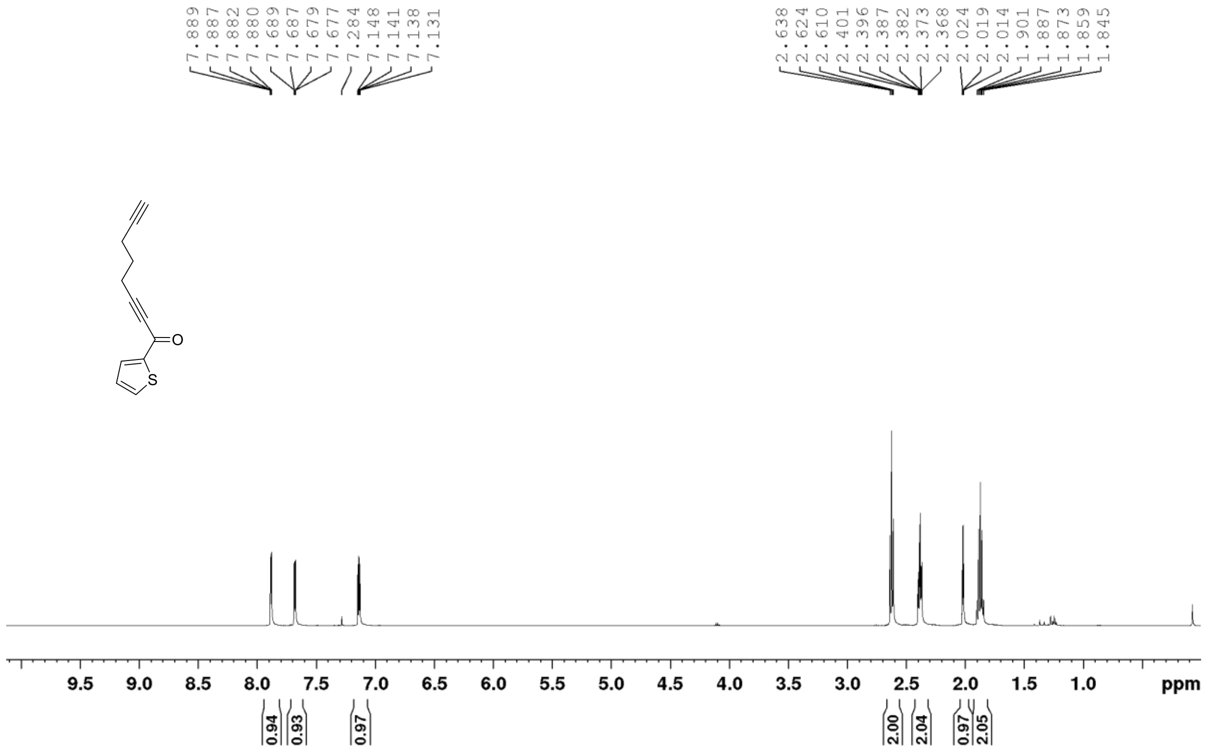

Supplementary Figure 36. The ^{1}H NMR (400.1 MHz, $CDCl_3$) spectrum of complex **S5-2** at room temperature.

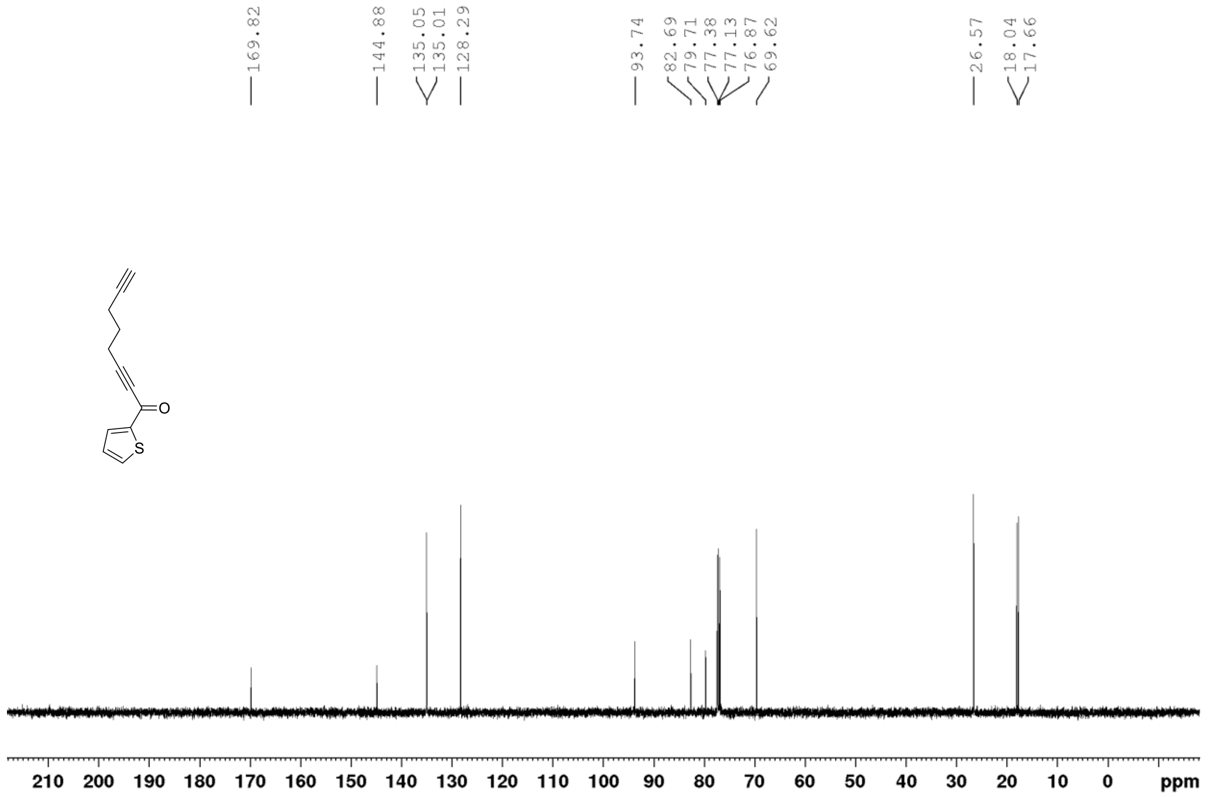

Supplementary Figure 37. The ^{13}C NMR (100.6 MHz, $CDCl_3$) spectrum of complex **S5-2** at room temperature.

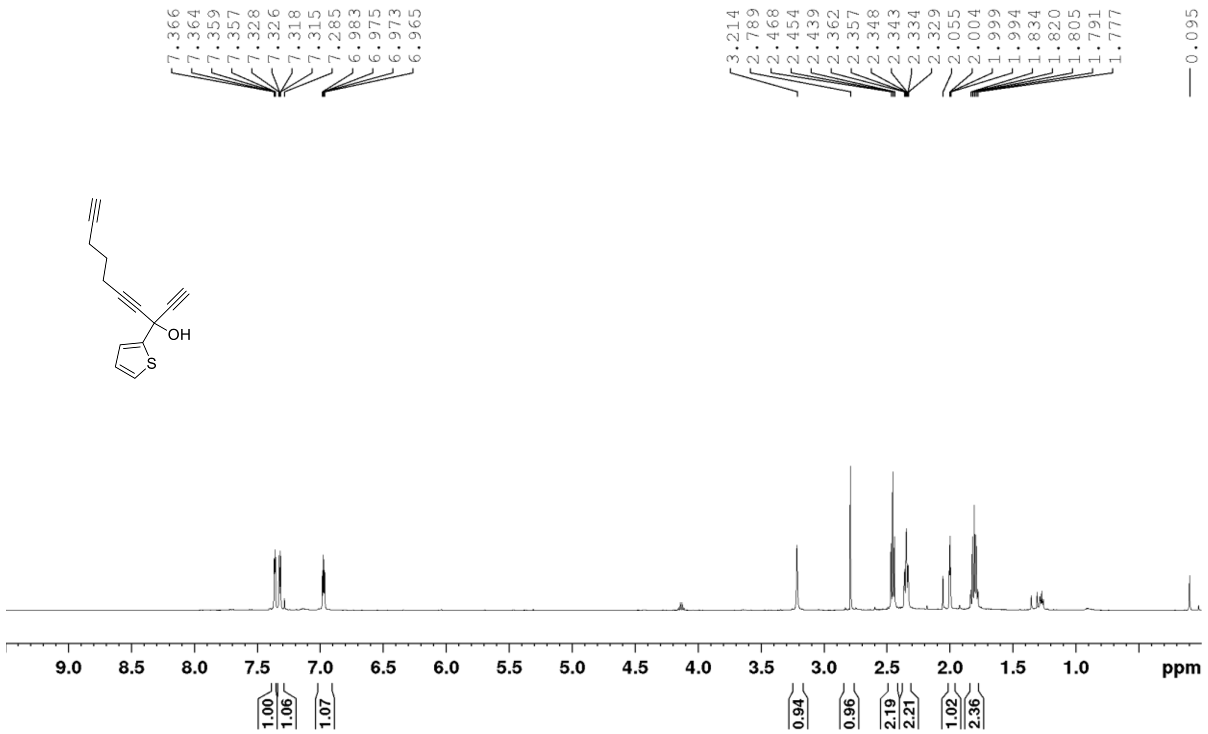

Supplementary Figure 38. The ^{1}H NMR (400.1 MHz, $CDCl_3$) spectrum of complex **L5** at room temperature.

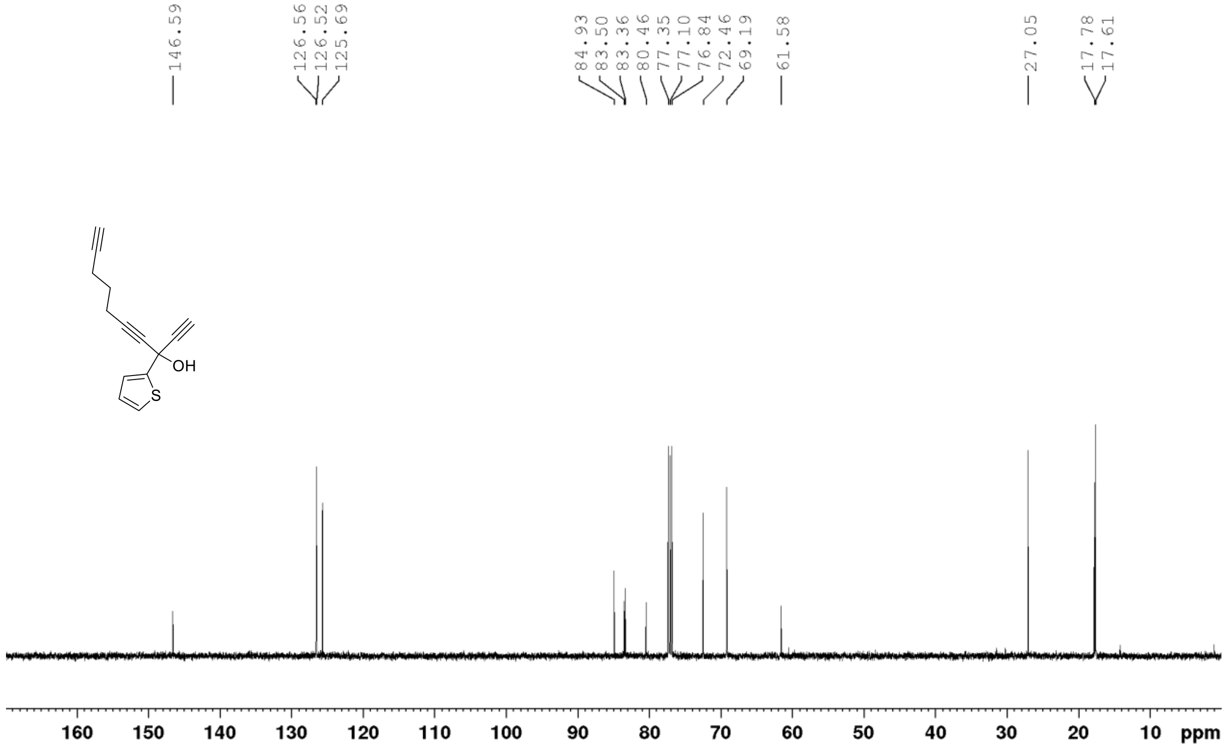

Supplementary Figure 39. The ^{13}C NMR (100.6 MHz, $CDCl_3$) spectrum of complex **L5** at room temperature.

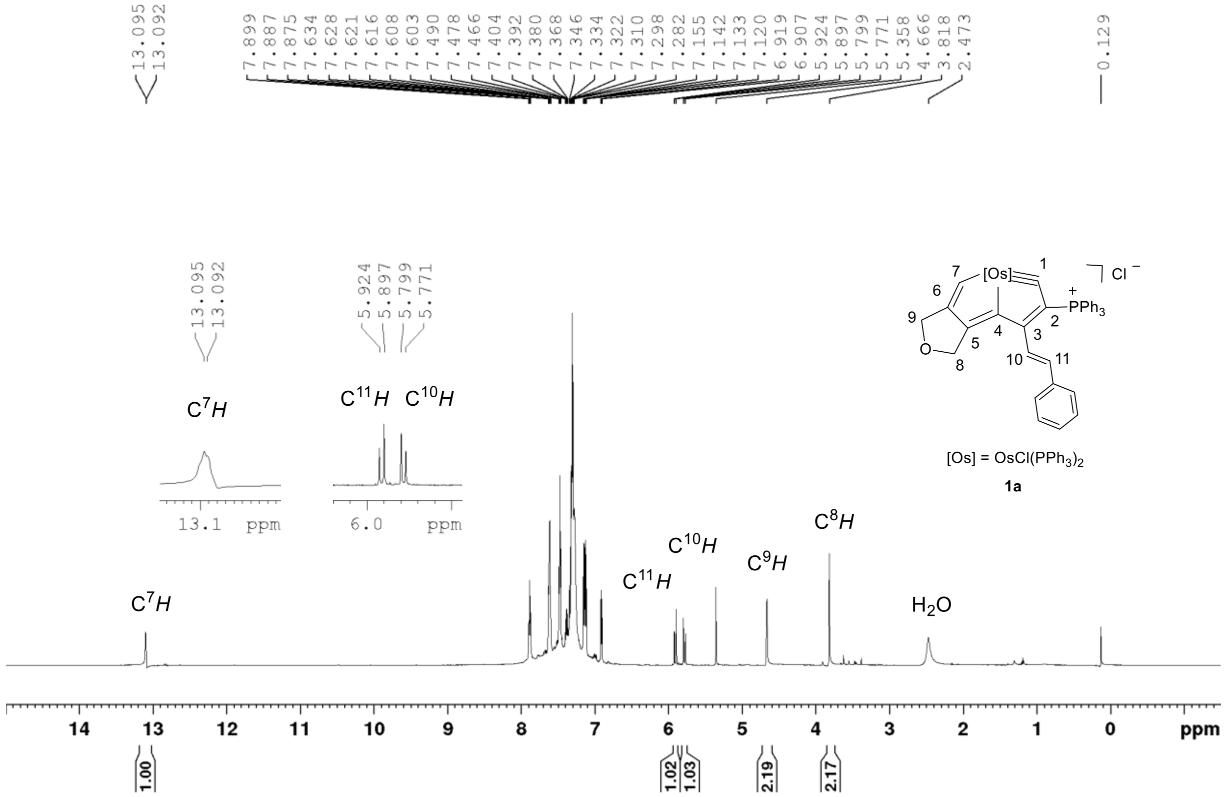

Supplementary Figure 40. The ^{1}H NMR (600.1 MHz, CD_2Cl_2) spectrum for complex **1a**.

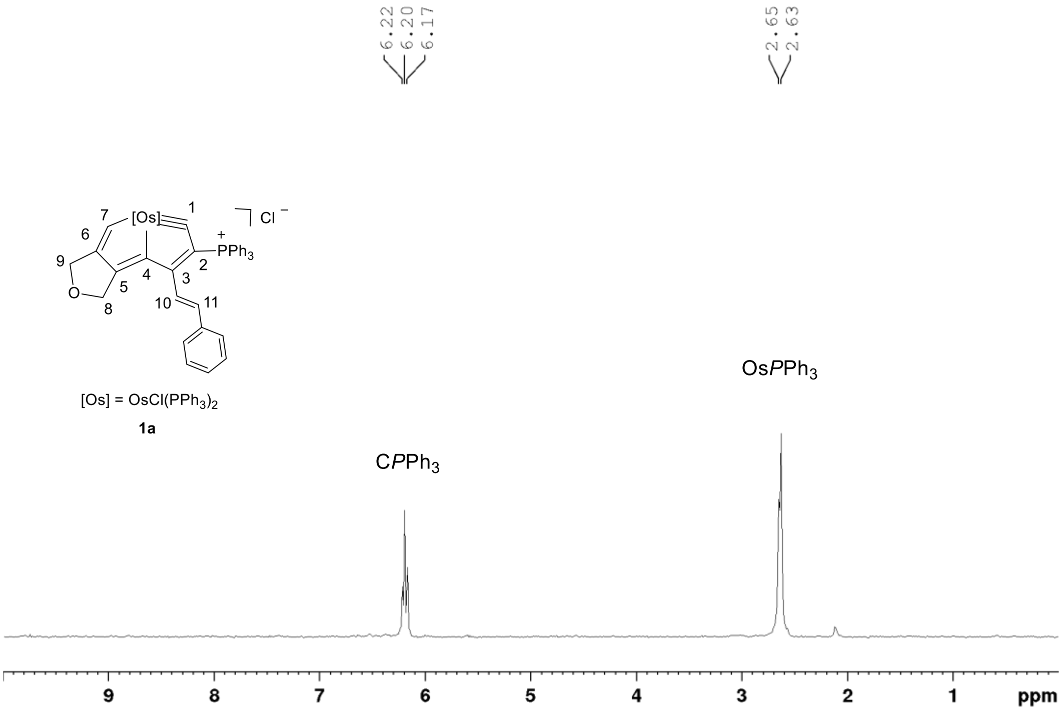

Supplementary Figure 41. The ^{31}P{^{1}H} NMR (242.9 MHz, CD_2Cl_2) spectrum for complex **1a**.

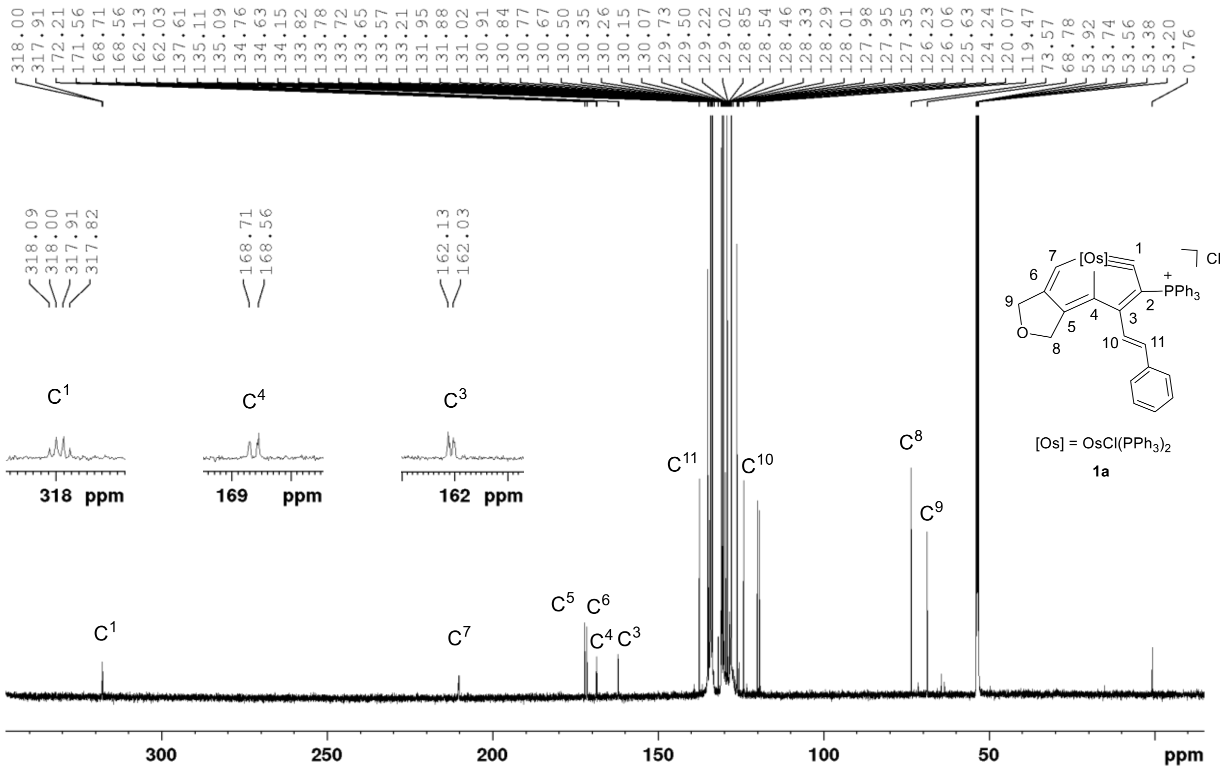

Supplementary Figure 42. The $^{13}C\{^{1}H\}$ NMR (150.9 MHz, CD_2Cl_2) spectrum for complex **1a**.

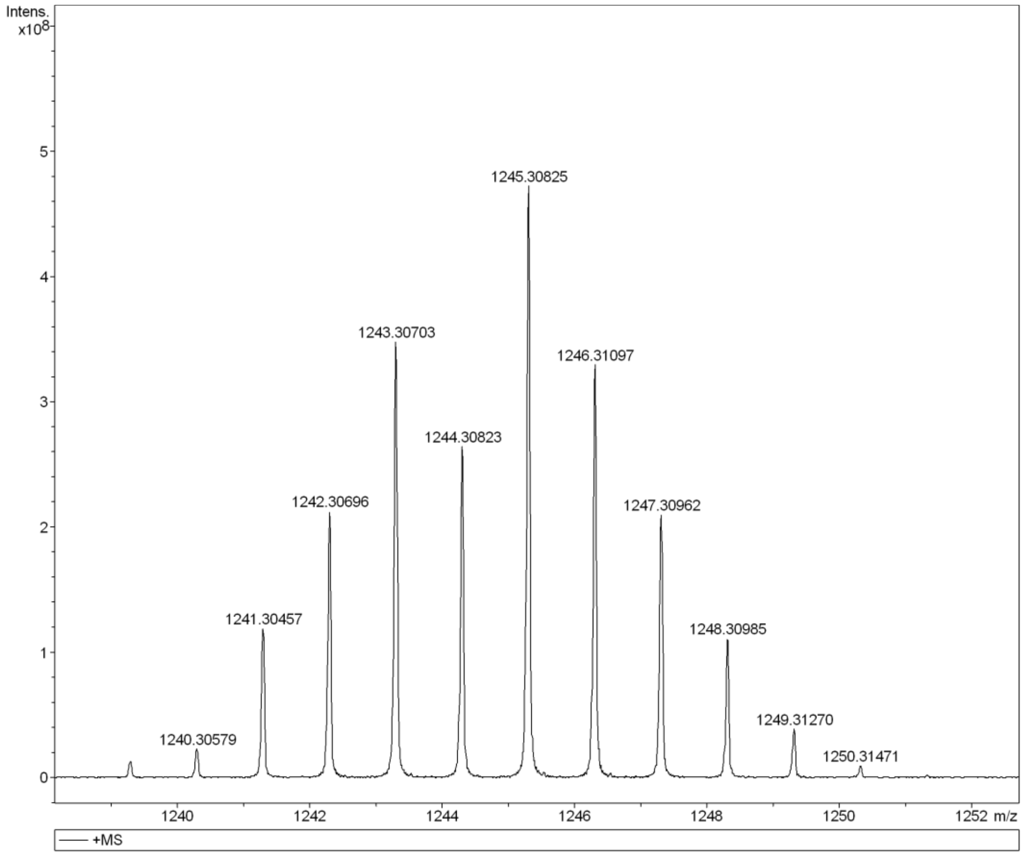

Supplementary Figure 43. Positive-ion ESI-MS spectrum of $[\mathbf{1a}]^{+}$ measured in methanol.

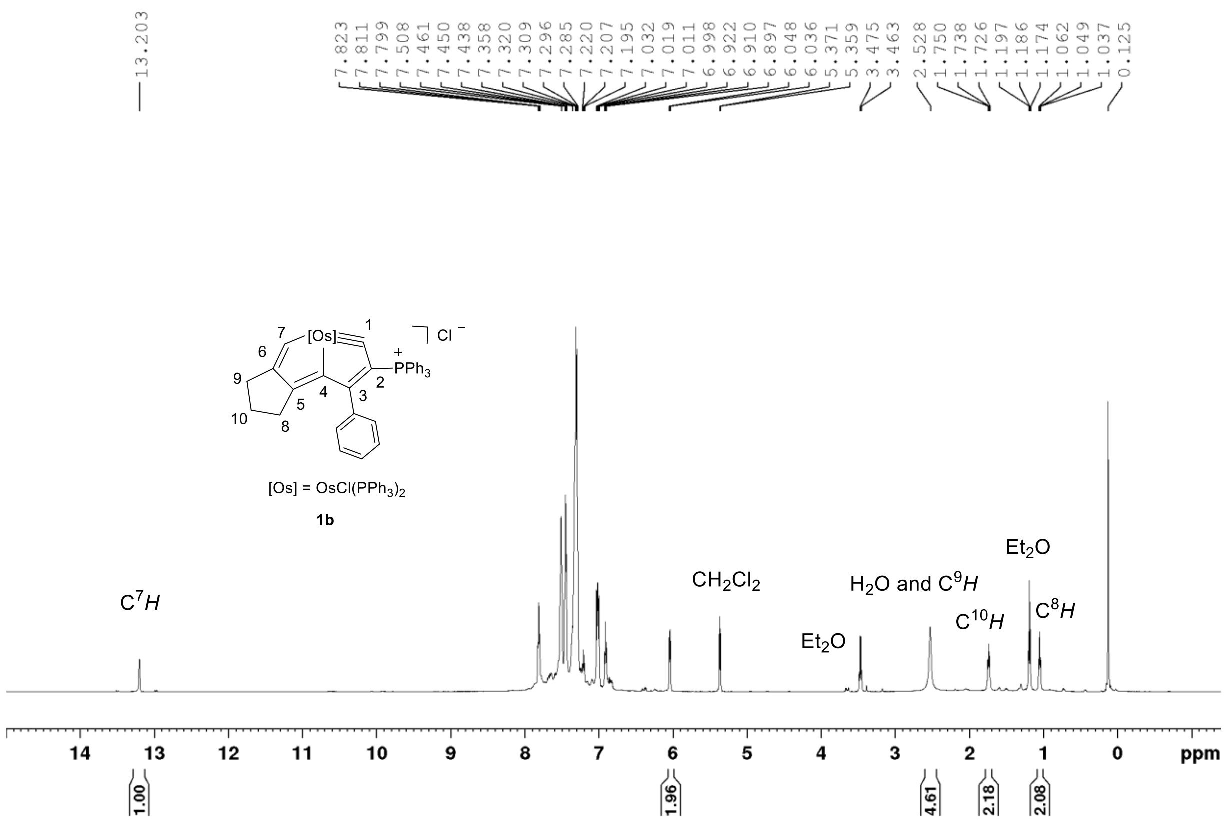

Supplementary Figure 44. The ^{1}H NMR (600.1 MHz, CD_2Cl_2) spectrum for complex **1b**.

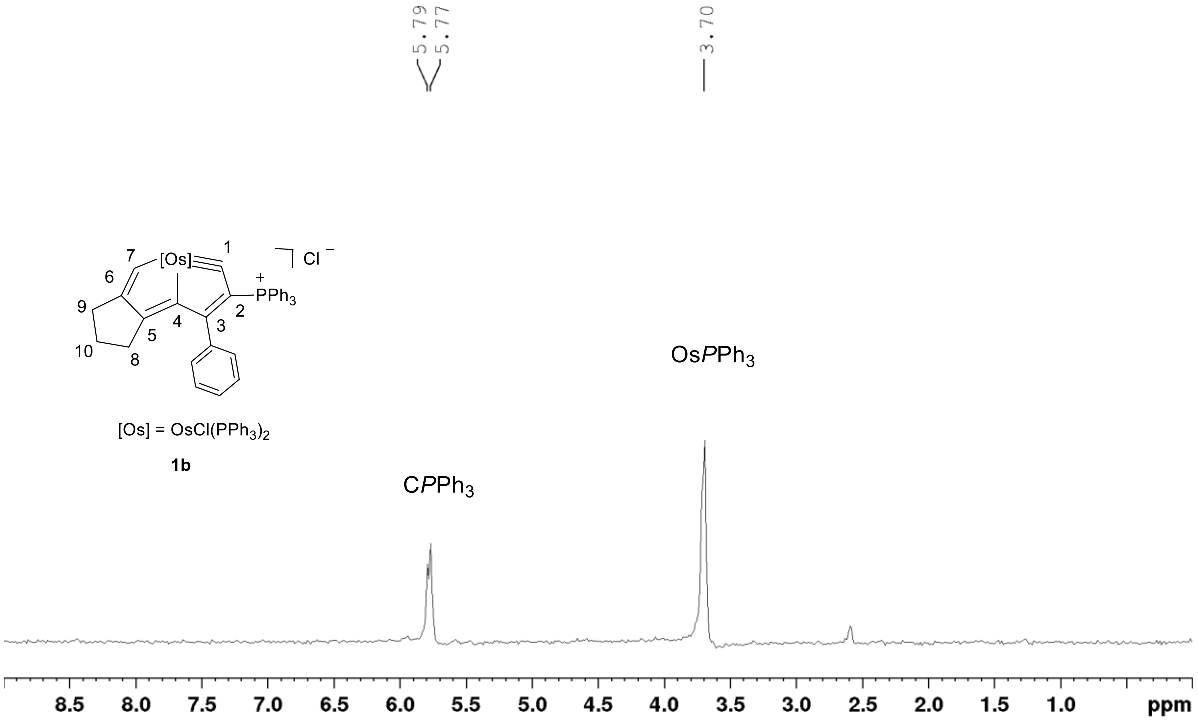

Supplementary Figure 45. The ^{31}P{^{1}H} NMR (242.9 MHz, CD_2Cl_2) spectrum for complex **1b**.

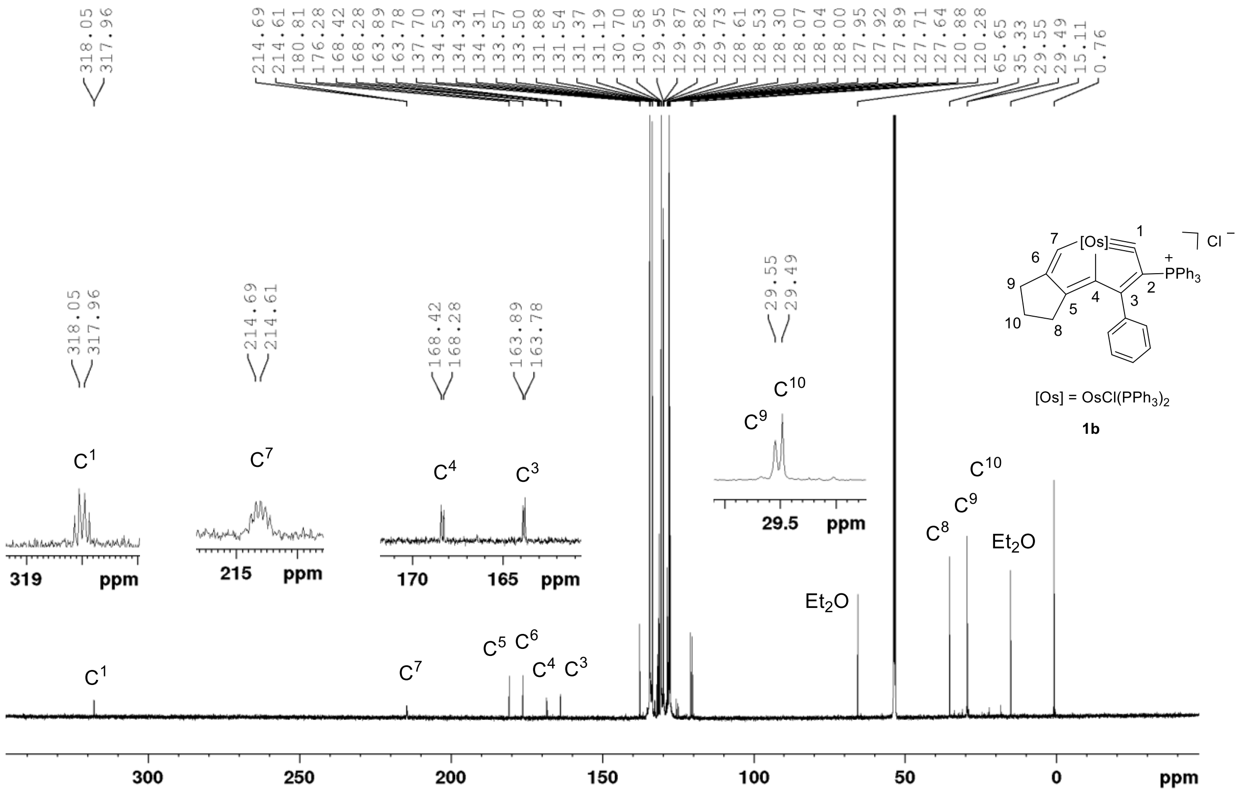

Supplementary Figure 46. The $^{13}C\{^{1}H\}$ NMR (150.9 MHz, CD_2Cl_2) spectrum for complex **1b**.

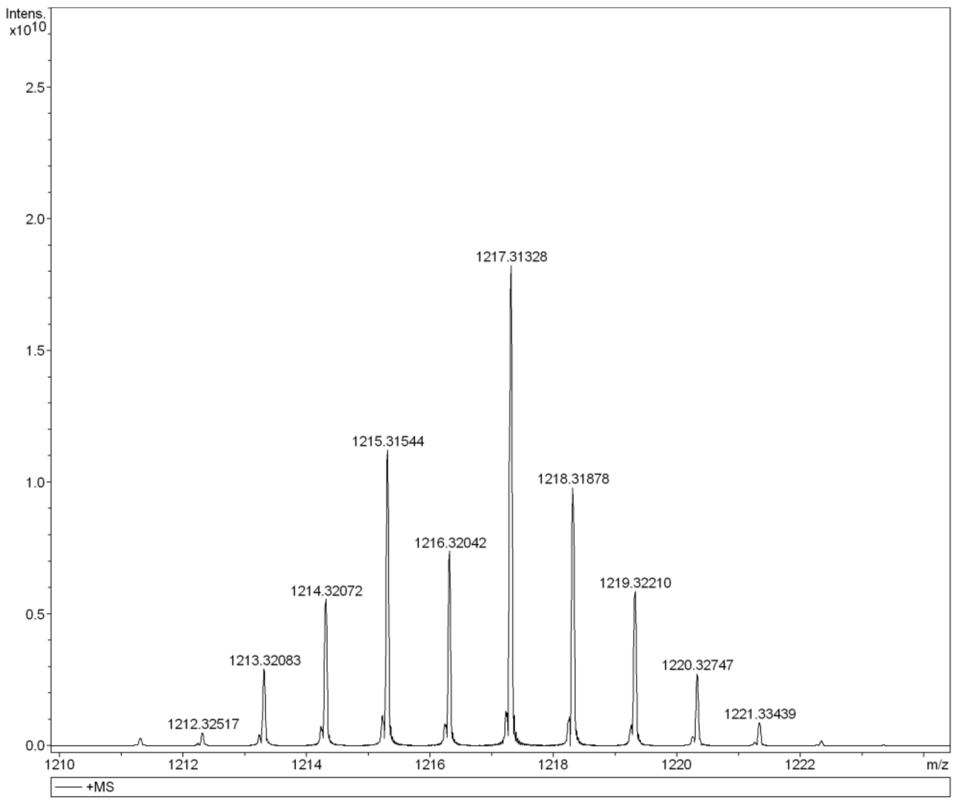

Supplementary Figure 47. Positive-ion ESI-MS spectrum of $[\mathbf{1b}]^+$ measured in methanol.

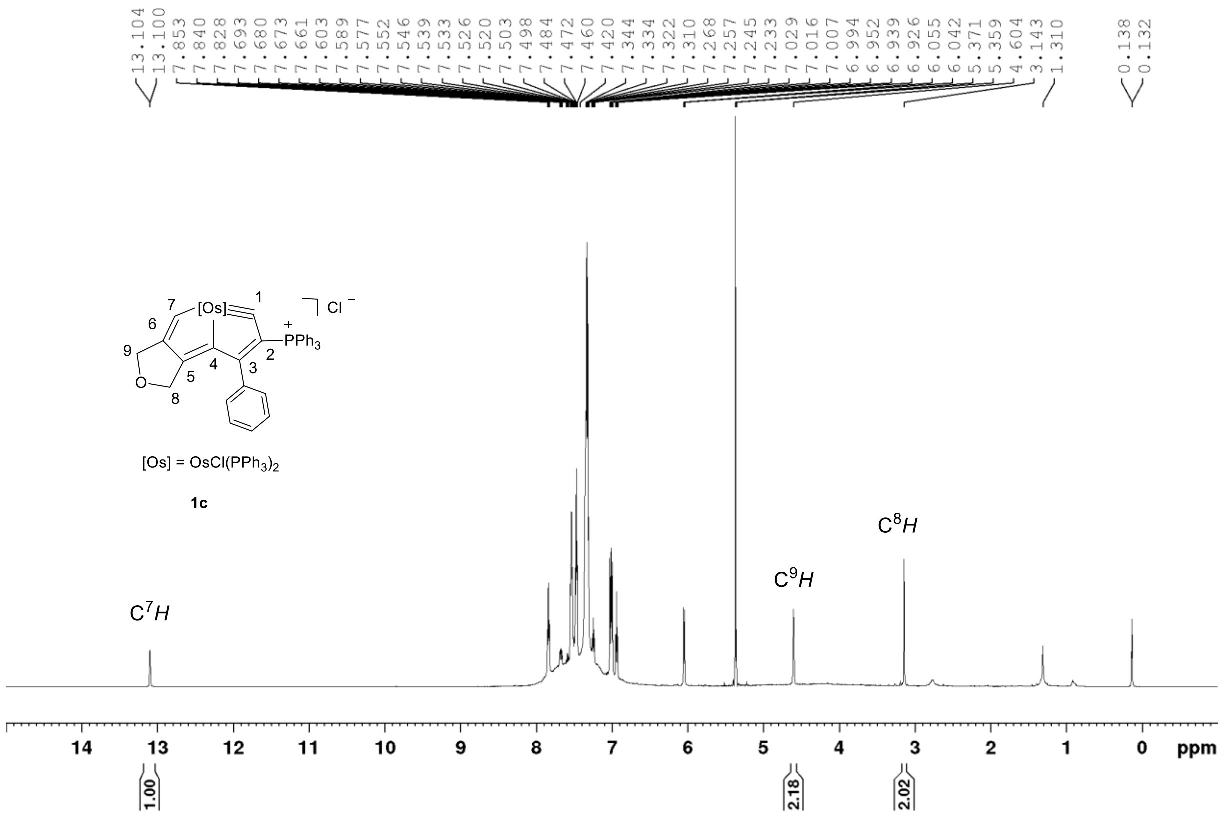

Supplementary Figure 48. The ^{1}H NMR (600.1 MHz, CD_2Cl_2) spectrum for complex **1c**.

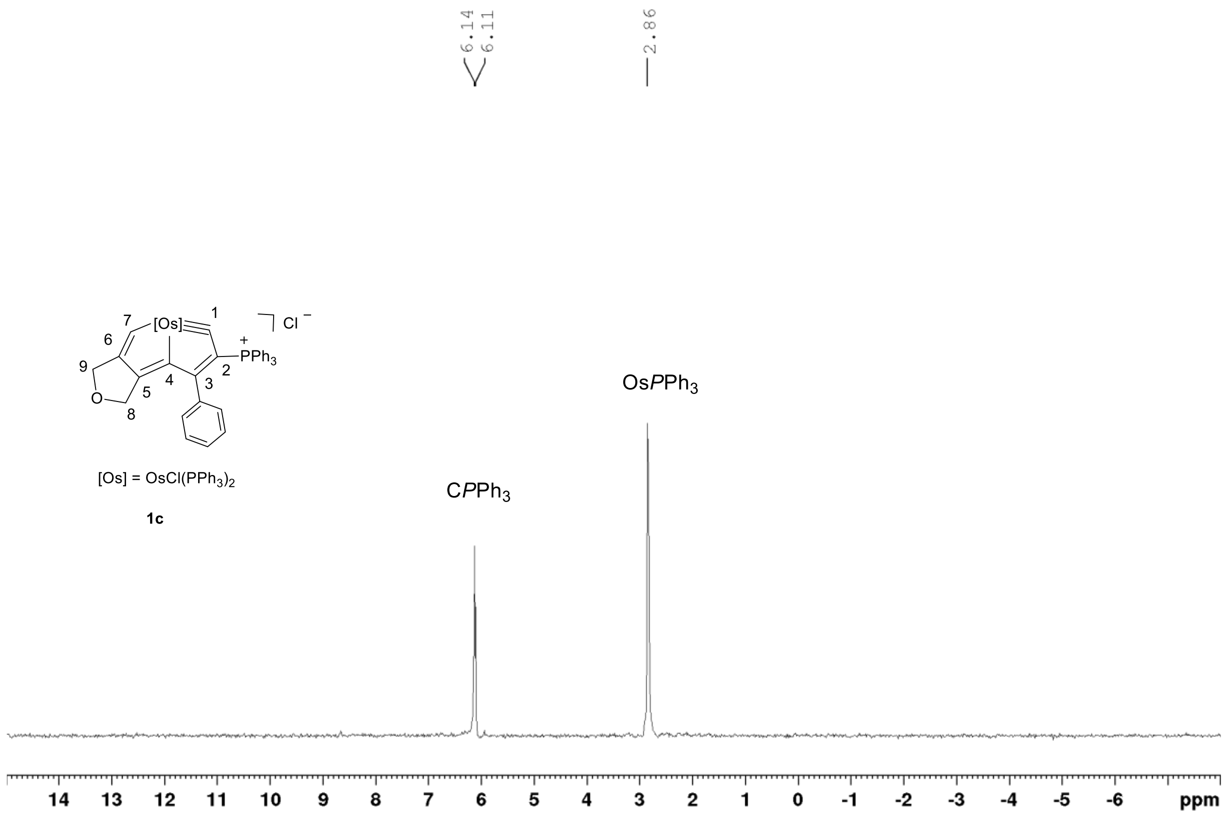

Supplementary Figure 49. The $^{31}P\{^1H\}$ NMR (242.9 MHz, CD_2Cl_2) spectrum for complex **1c**.

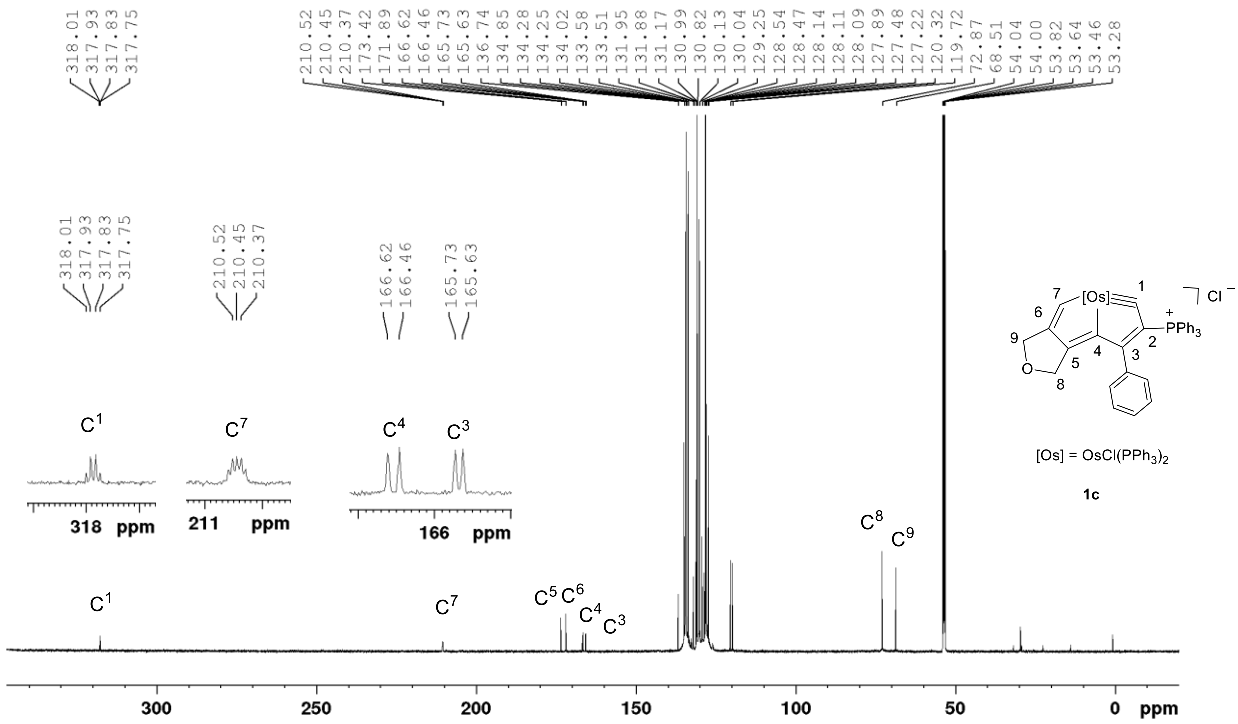

Supplementary Figure 50. The $^{13}C\{^{1}H\}$ NMR (150.9 MHz, CD_2Cl_2) spectrum for complex **1c**.

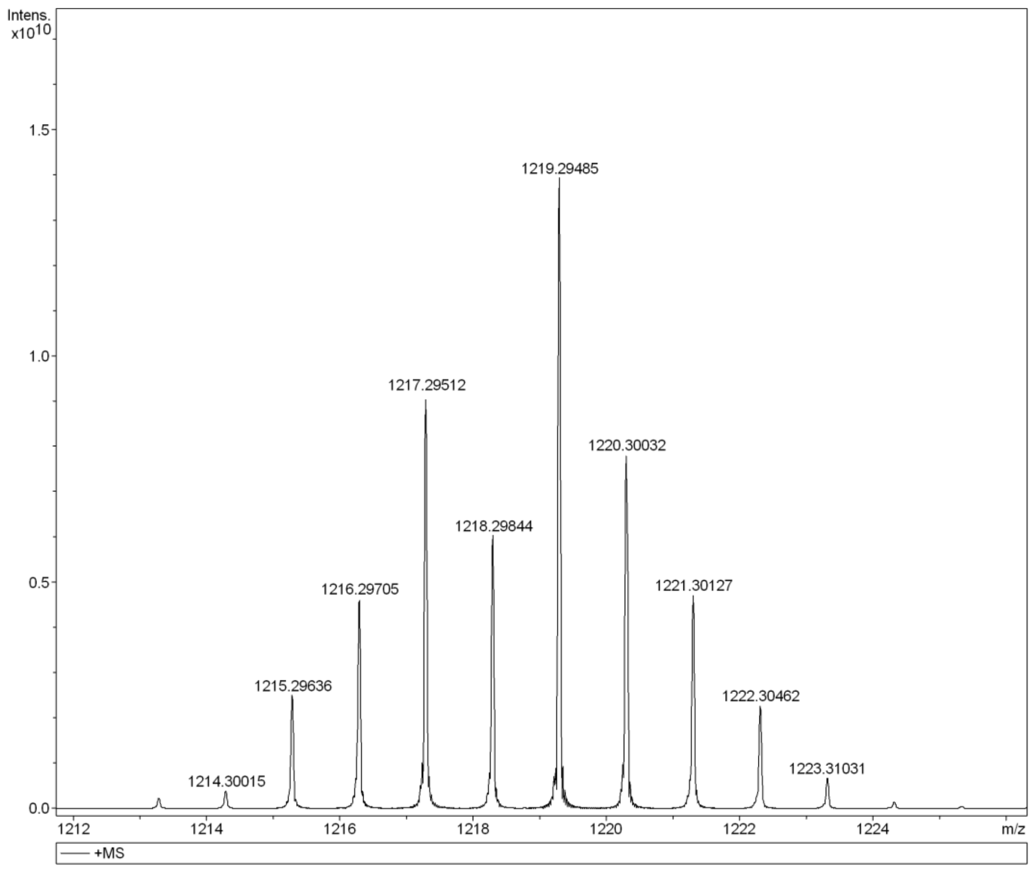

Supplementary Figure 51. Positive-ion ESI-MS spectrum of $[\mathbf{1c}]^+$ measured in methanol.

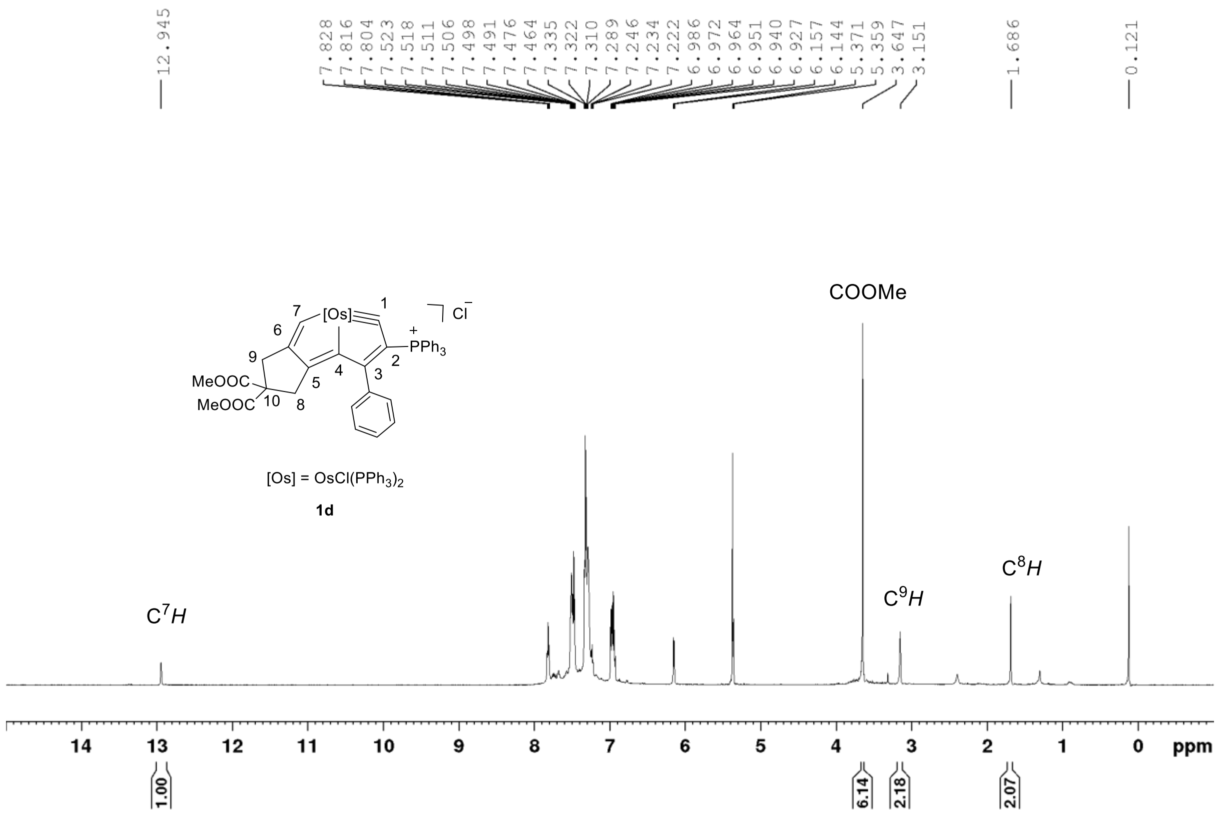

Supplementary Figure 52. The ^{1}H NMR (600.1 MHz, CD_2Cl_2) spectrum for complex **1d**.

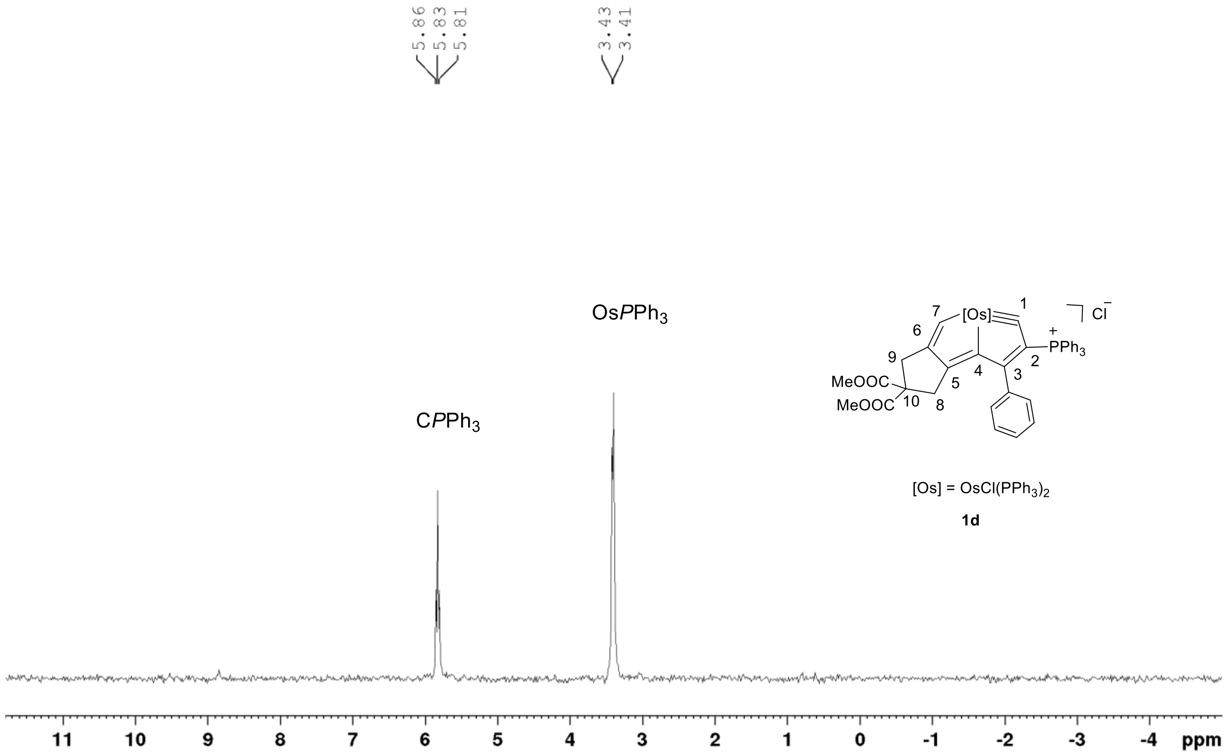

Supplementary Figure 53. The ^{31}P{^{1}H} NMR (242.9 MHz, CD_2Cl_2) spectrum for complex **1d**.

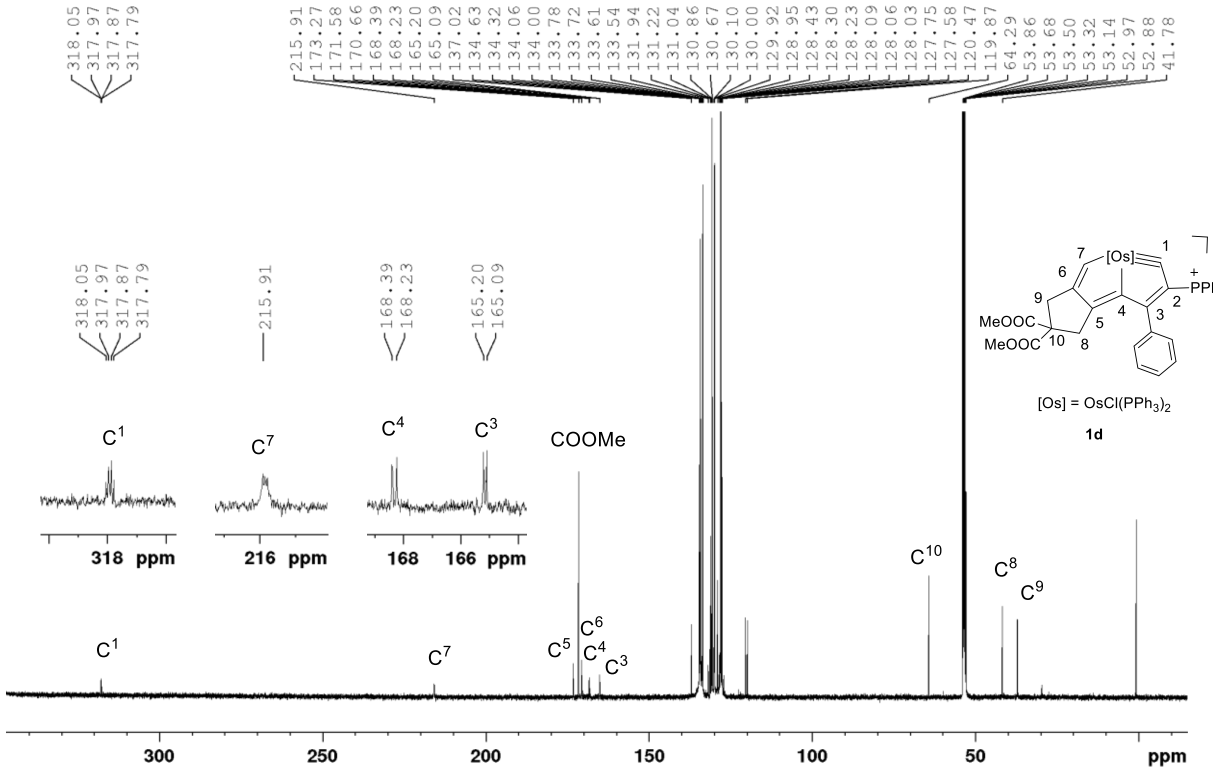

Supplementary Figure 54. The $^{13}C\{^{1}H\}$ NMR (150.9 MHz, CD_2Cl_2) spectrum for complex **1d**.

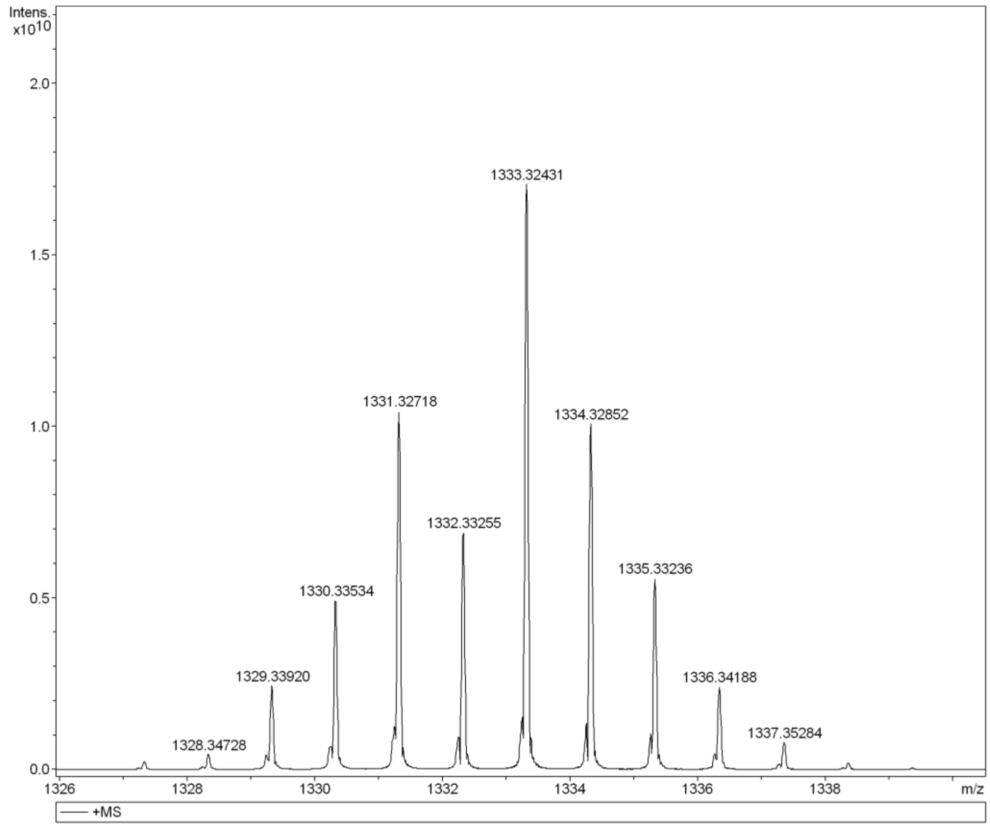

Supplementary Figure 55. Positive-ion ESI-MS spectrum of [**1d**]$^+$ measured in methanol.

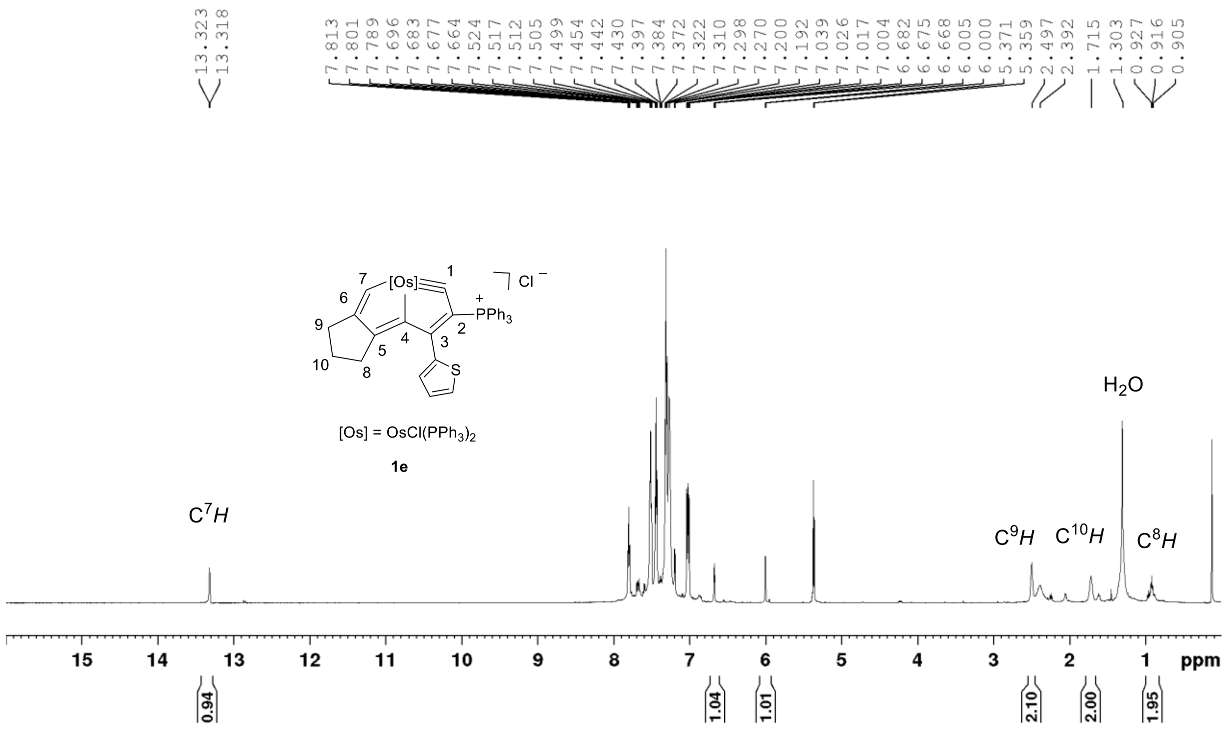

Supplementary Figure 56. The ^{1}H NMR (600.1 MHz, CD_2Cl_2) spectrum for complex **1e**.

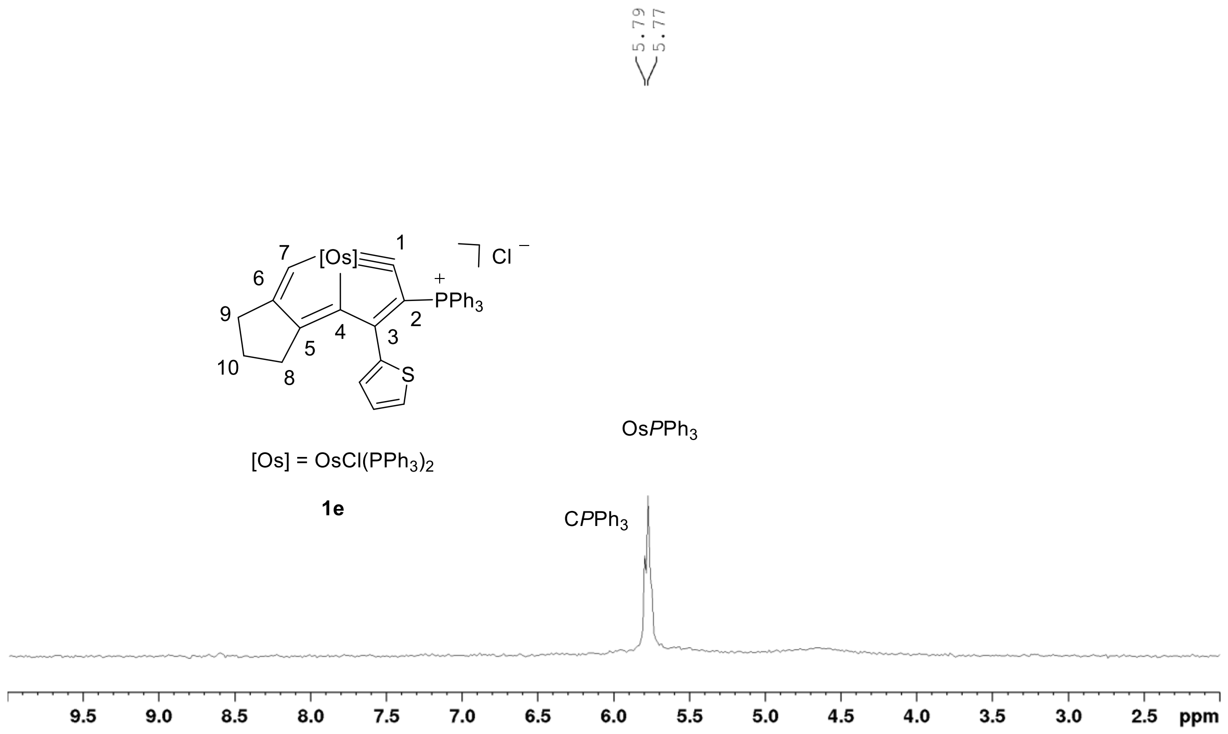

Supplementary Figure 57. The $^{31}P\{^{1}H\}$ NMR (242.9 MHz, CD_2Cl_2) spectrum for complex **1e**.

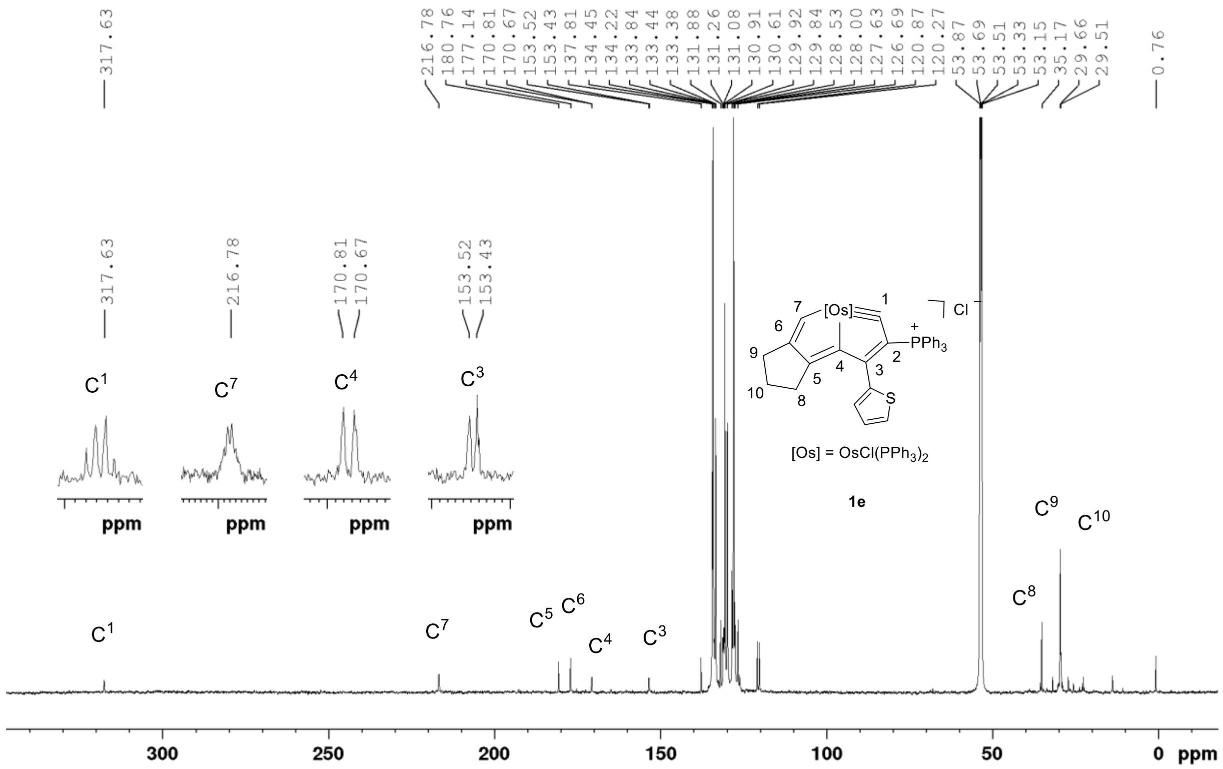

Supplementary Figure 58. The $^{13}C\{^1H\}$ NMR (150.9 MHz, CD_2Cl_2) spectrum for complex **1e**.

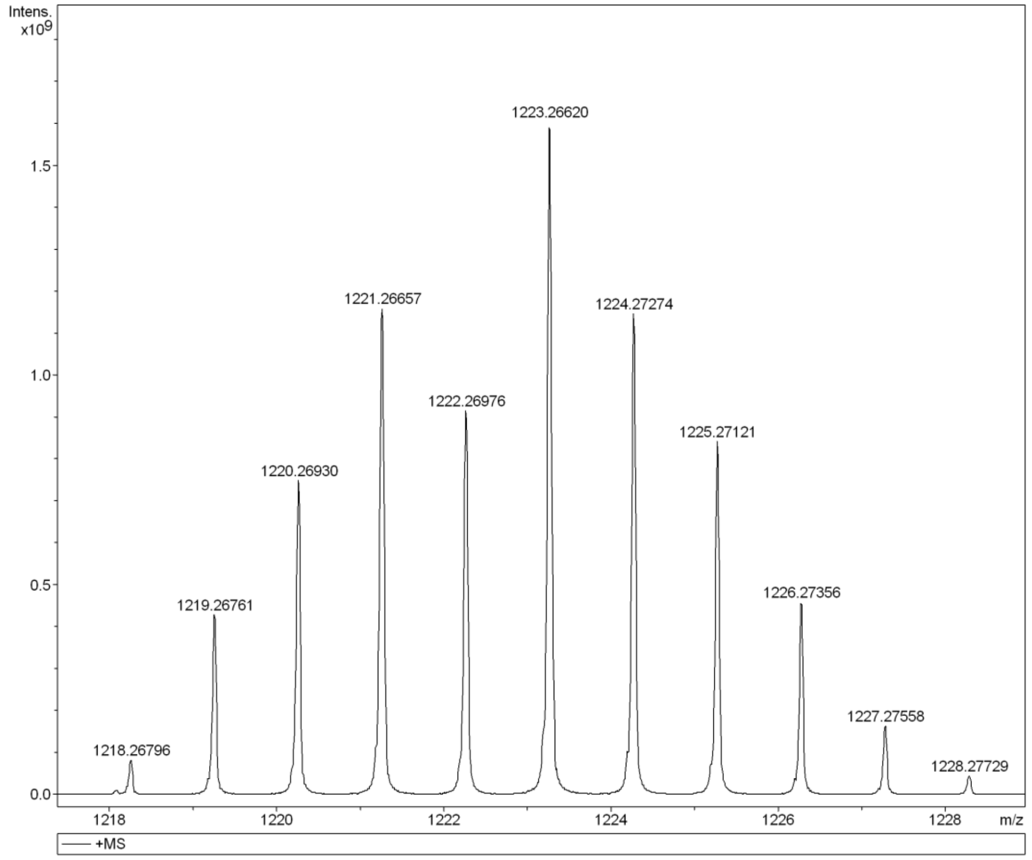

Supplementary Figure 59. Positive-ion ESI-MS spectrum of [**1e**]$^+$ measured in methanol.

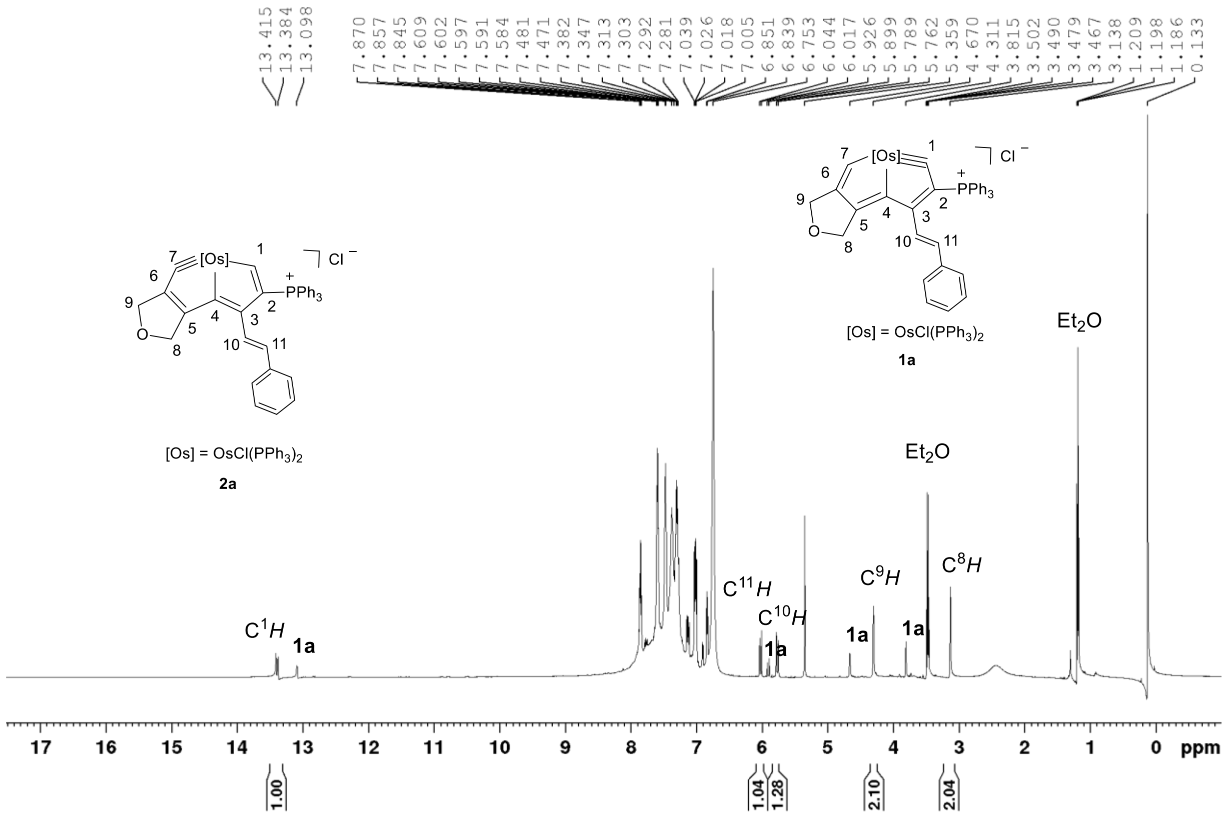

Supplementary Figure 60. The ^{1}H NMR (600.1 MHz, CD_2Cl_2) spectrum for complex **2a** and **1a**.

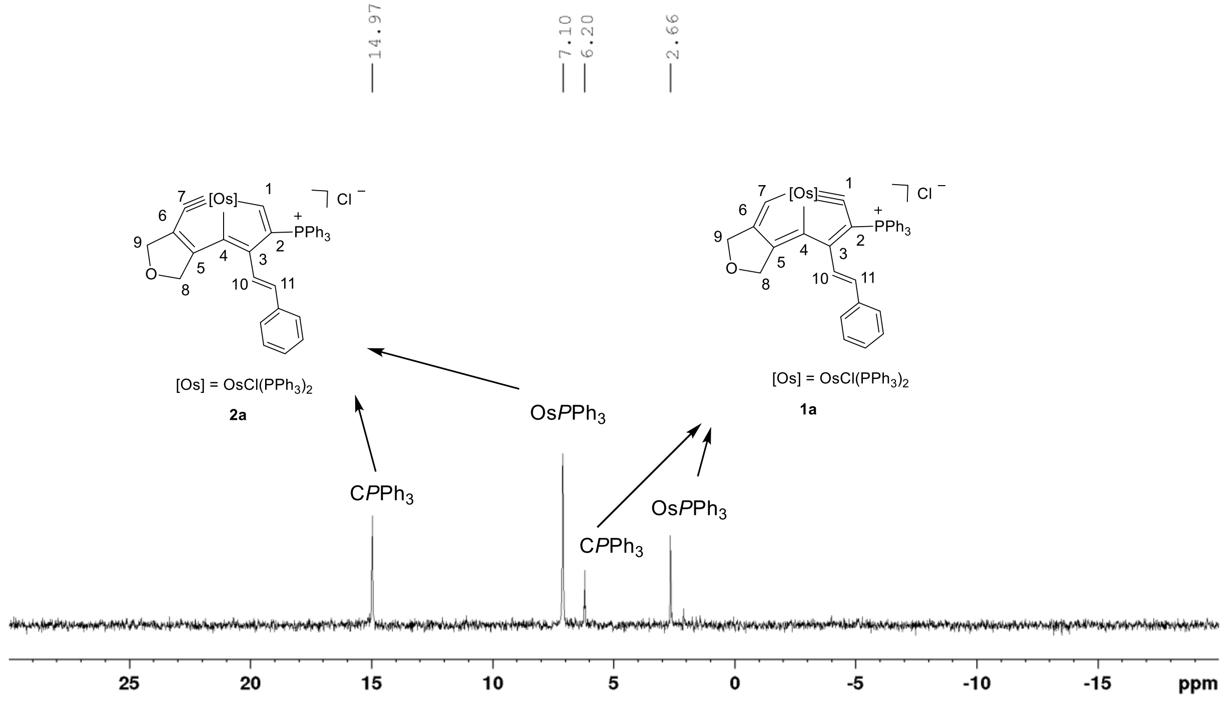

Supplementary Figure 61. The ^{31}P{^{1}H} NMR (242.9 MHz, CD_2Cl_2) spectrum for complex **2a** and **1a**.

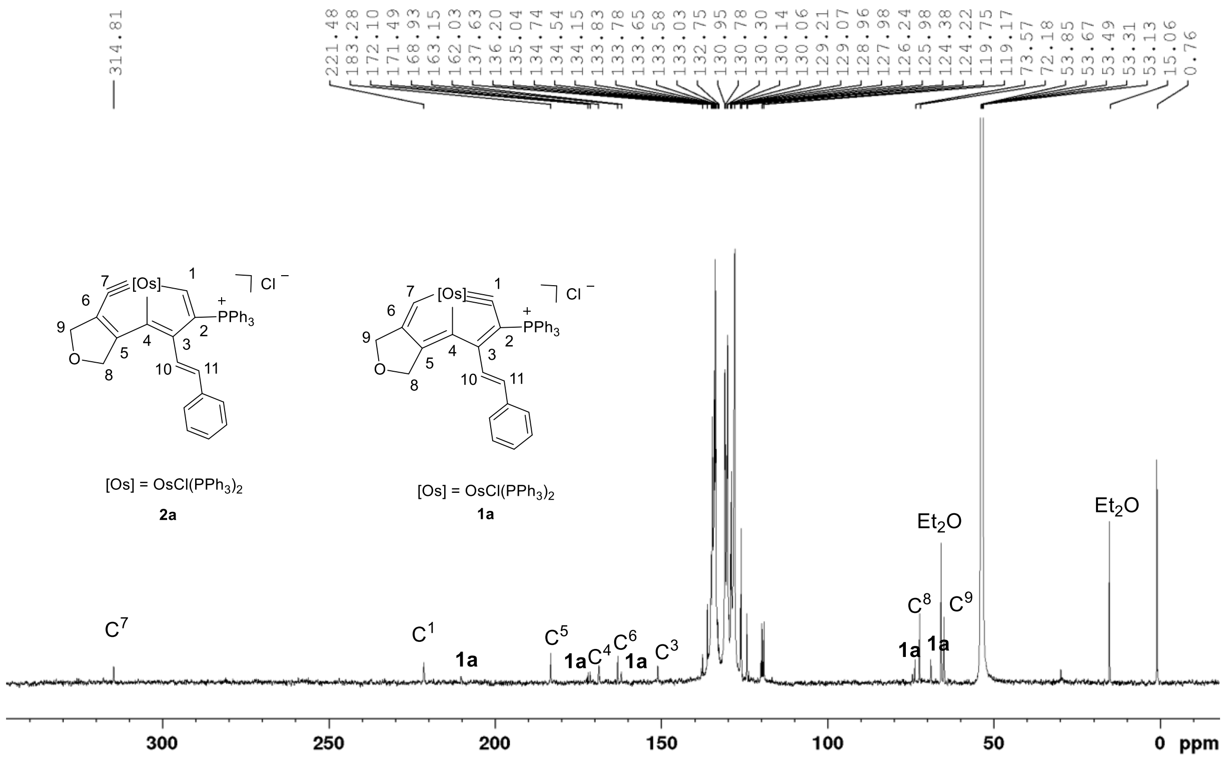

Supplementary Figure 62. The ${}^{13}C\{{}^{1}H\}$ NMR (150.9 MHz, CD_2Cl_2) spectrum for complex **2a** and **1a**.

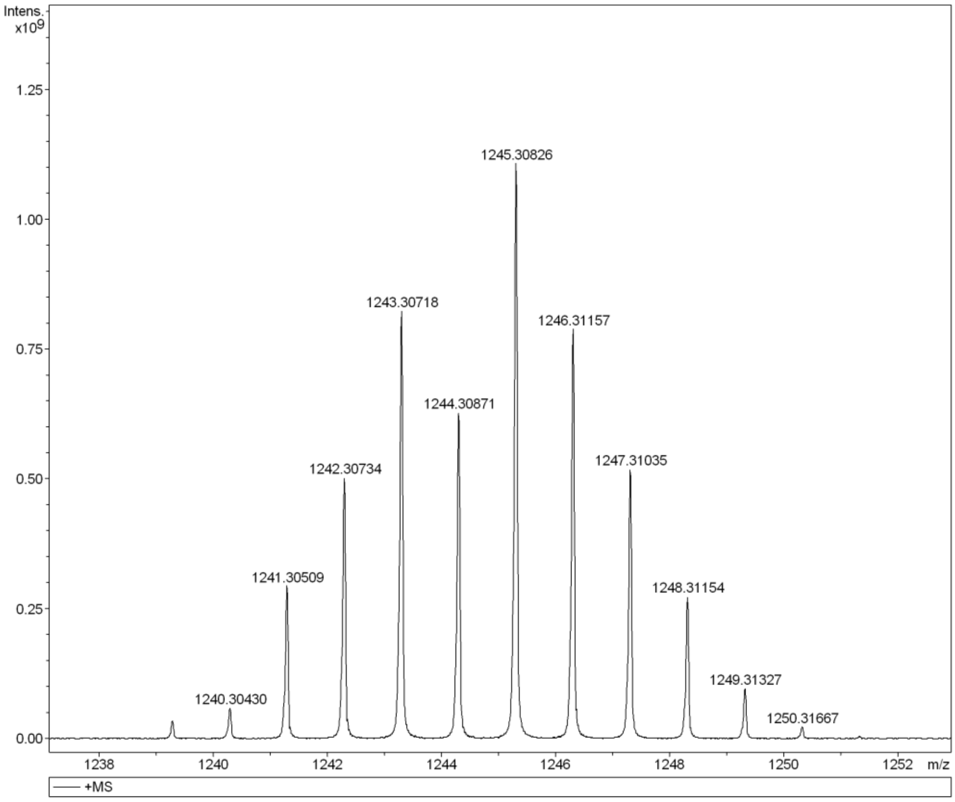

Supplementary Figure 63. Positive-ion ESI-MS spectrum of [**2a**]$^+$ and [**1a**]$^+$ measured in methanol.

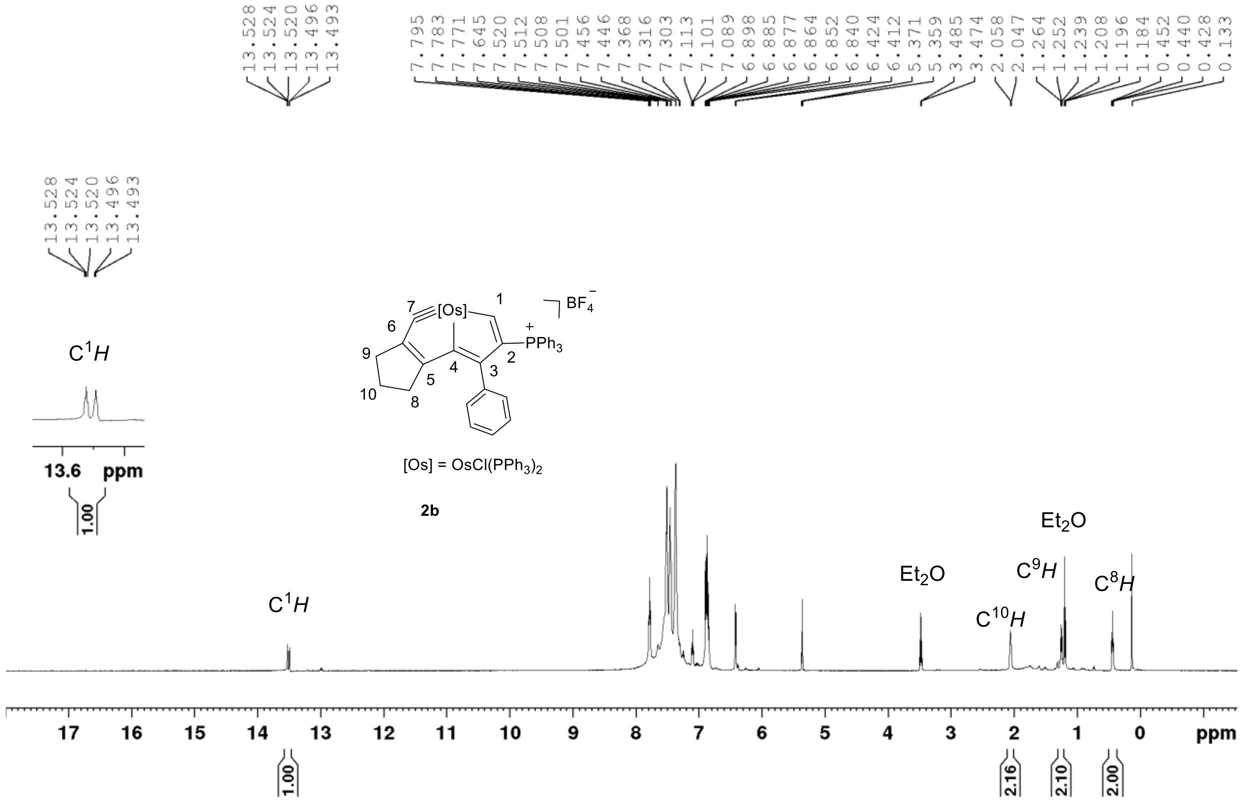

Supplementary Figure 64. The ^{1}H NMR (600.1 MHz, CD_2Cl_2) spectrum for complex **2b**.

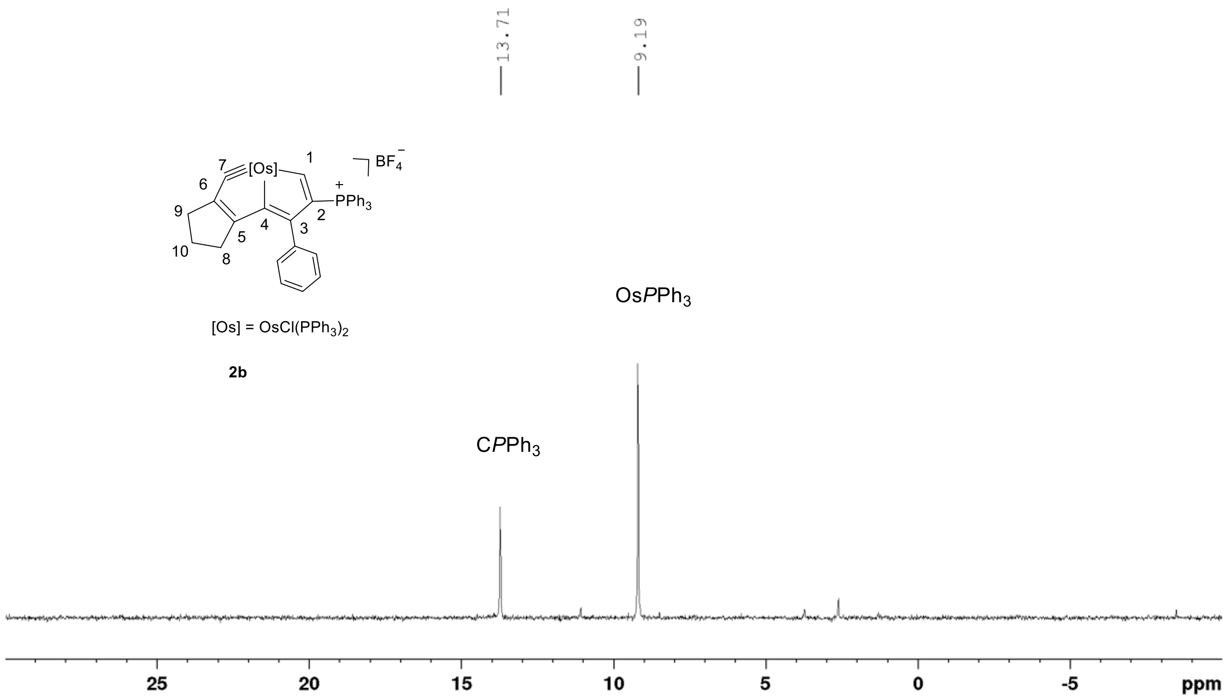

Supplementary Figure 65. The $^{31}P\{^{1}H\}$ NMR (242.9 MHz, CD_2Cl_2) spectrum for complex **2b**.

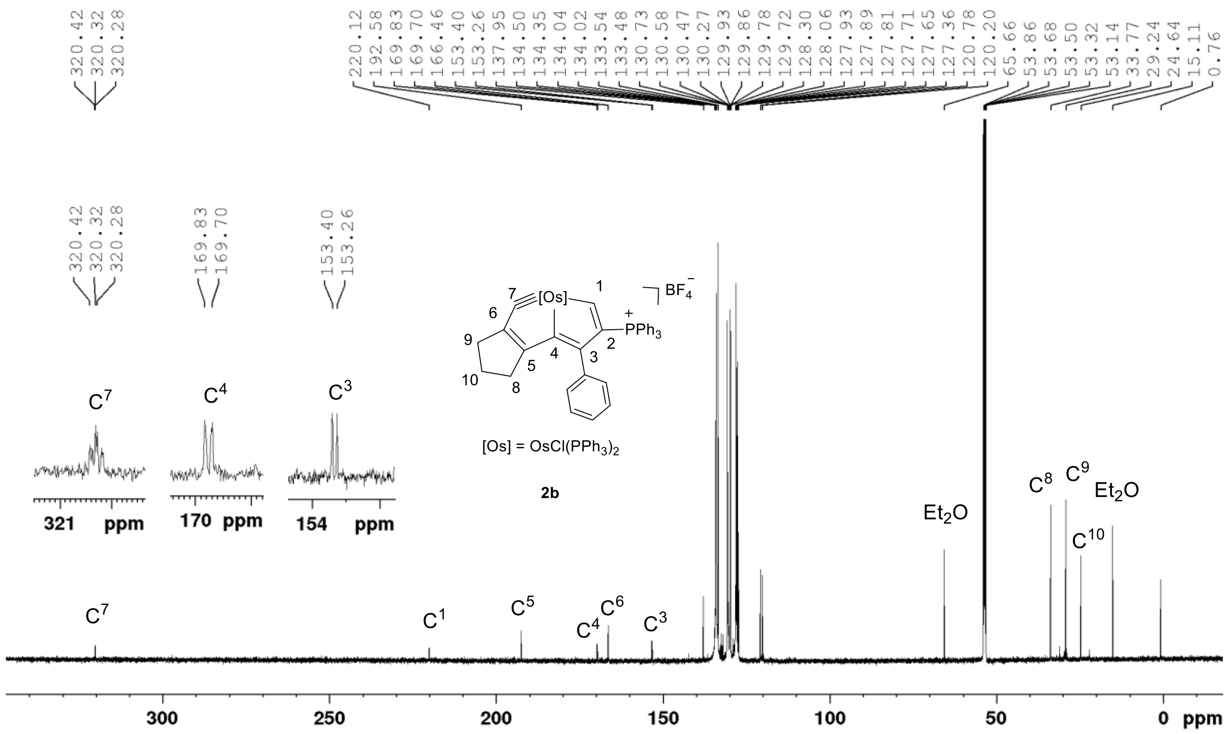

Supplementary Figure 66. The $^{13}C\{^{1}H\}$ NMR (150.9 MHz, CD_2Cl_2) spectrum for complex **2b**.

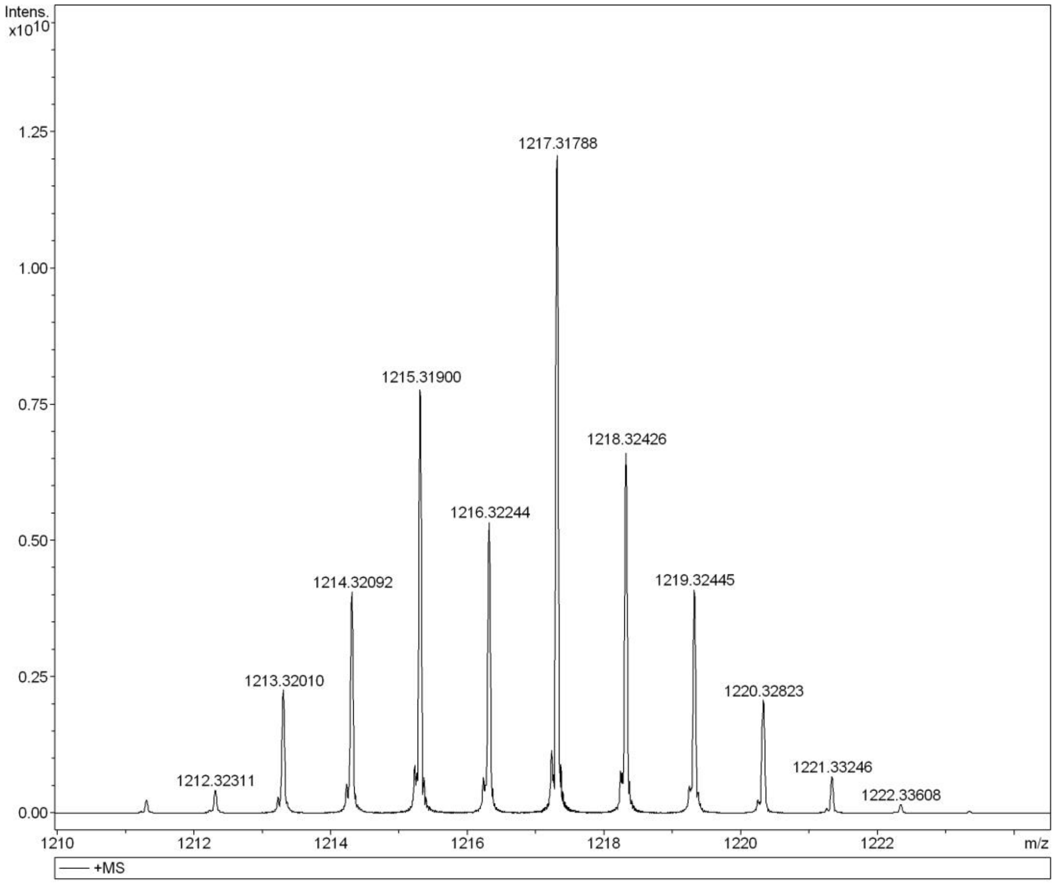

Supplementary Figure 67. Positive-ion ESI-MS spectrum of [**2b**]$^+$ measured in methanol.

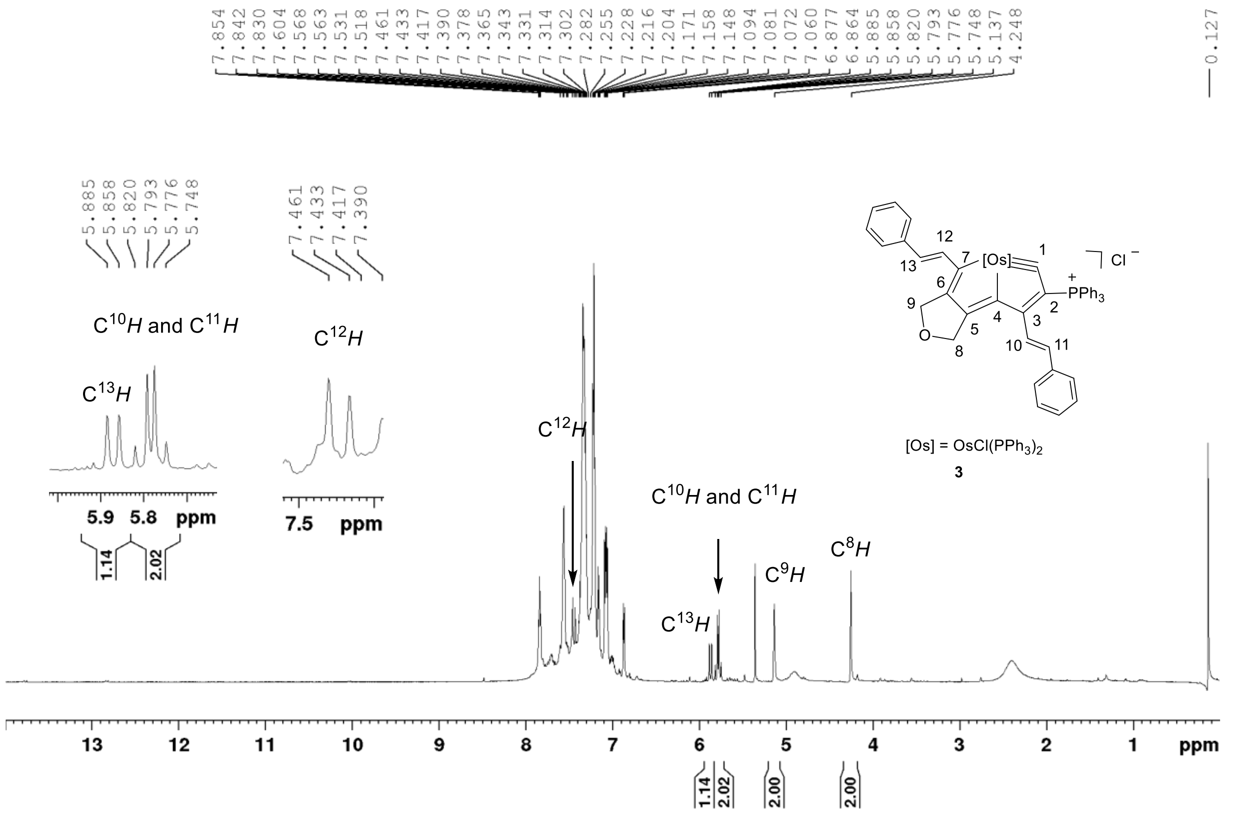

Supplementary Figure 68. The ^{1}H NMR (600.1 MHz, CD_2Cl_2) spectrum for complex **3**.

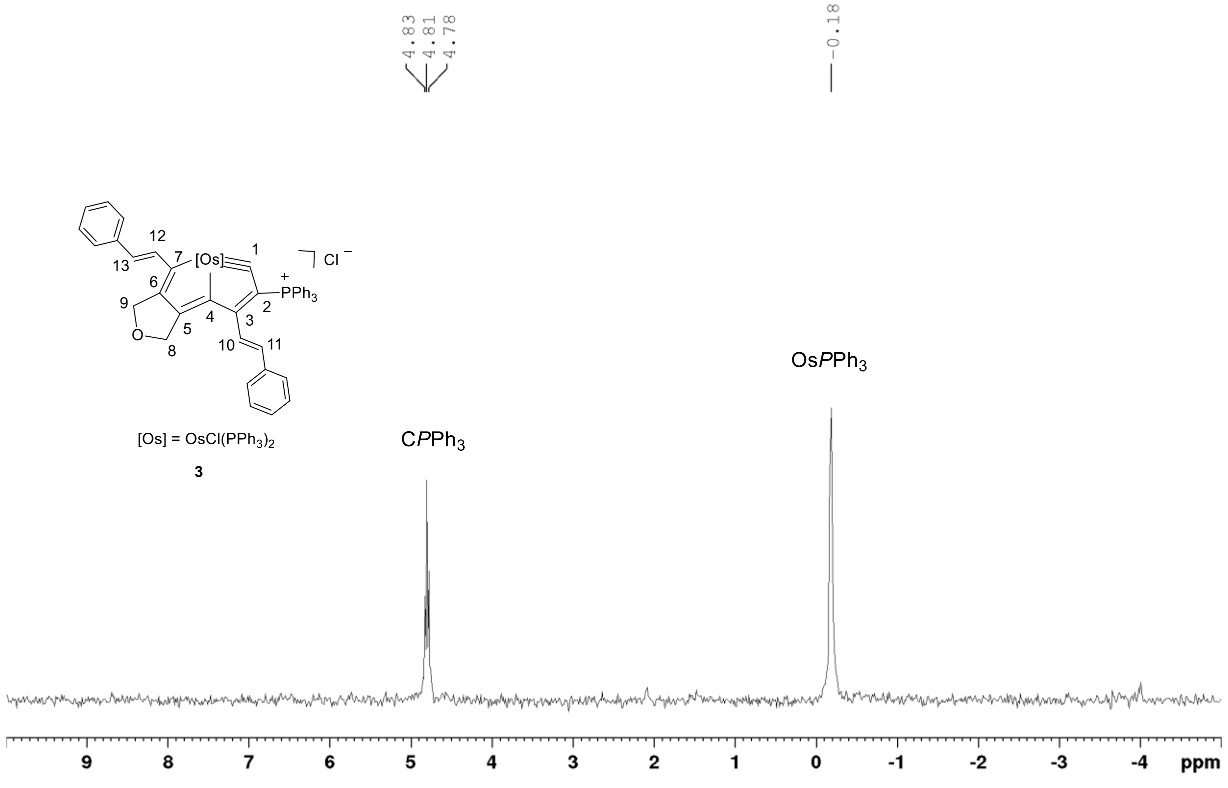

Supplementary Figure 69. The ^{31}P{^{1}H} NMR (242.9 MHz, CD_2Cl_2) spectrum for complex **3**.

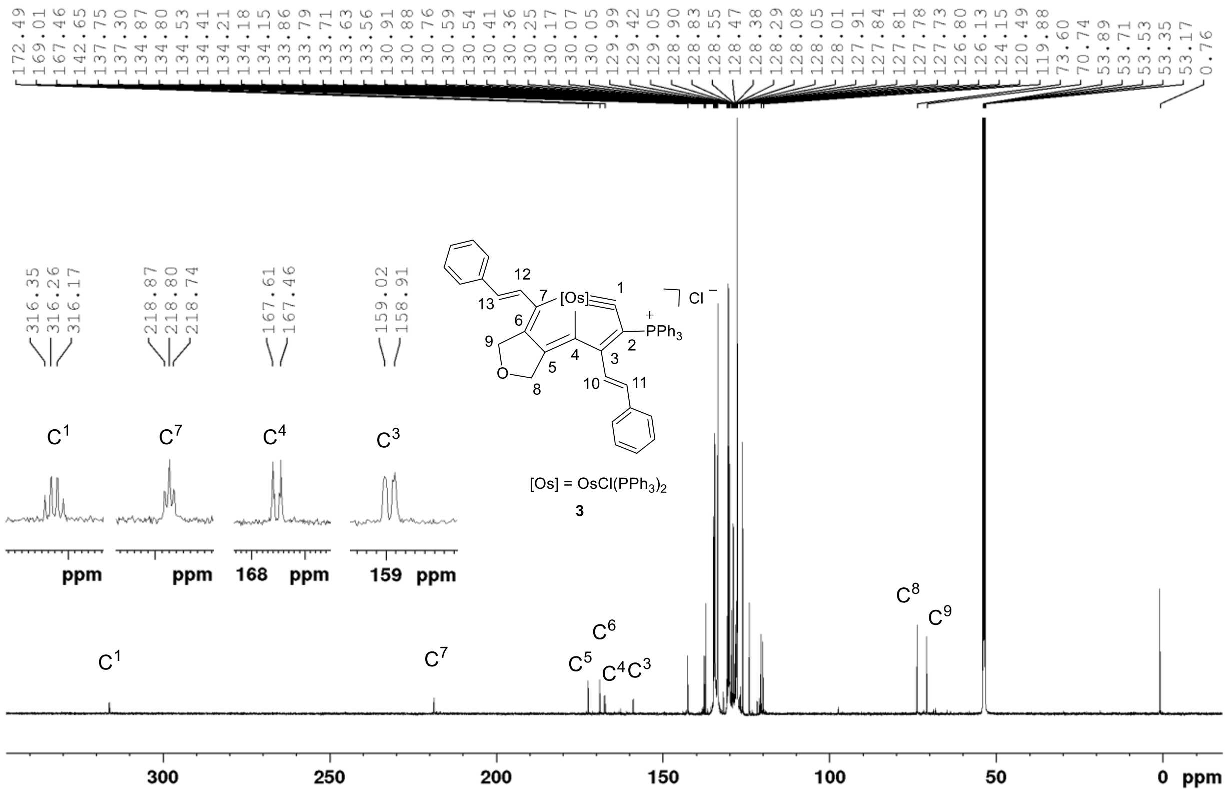

Supplementary Figure 70. The $^{13}C\{^{1}H\}$ NMR (150.9 MHz, CD_2Cl_2) spectrum for complex **3**.

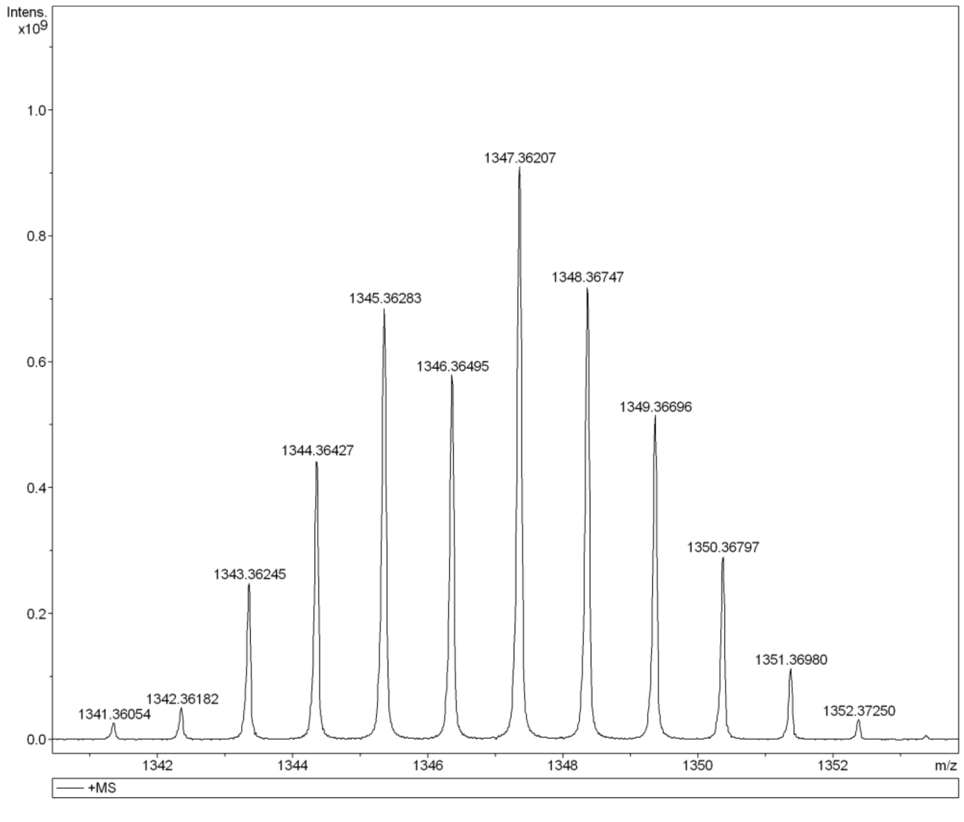

Supplementary Figure 71. Positive-ion ESI-MS spectrum of $[\mathbf{3}]^+$ measured in methanol.

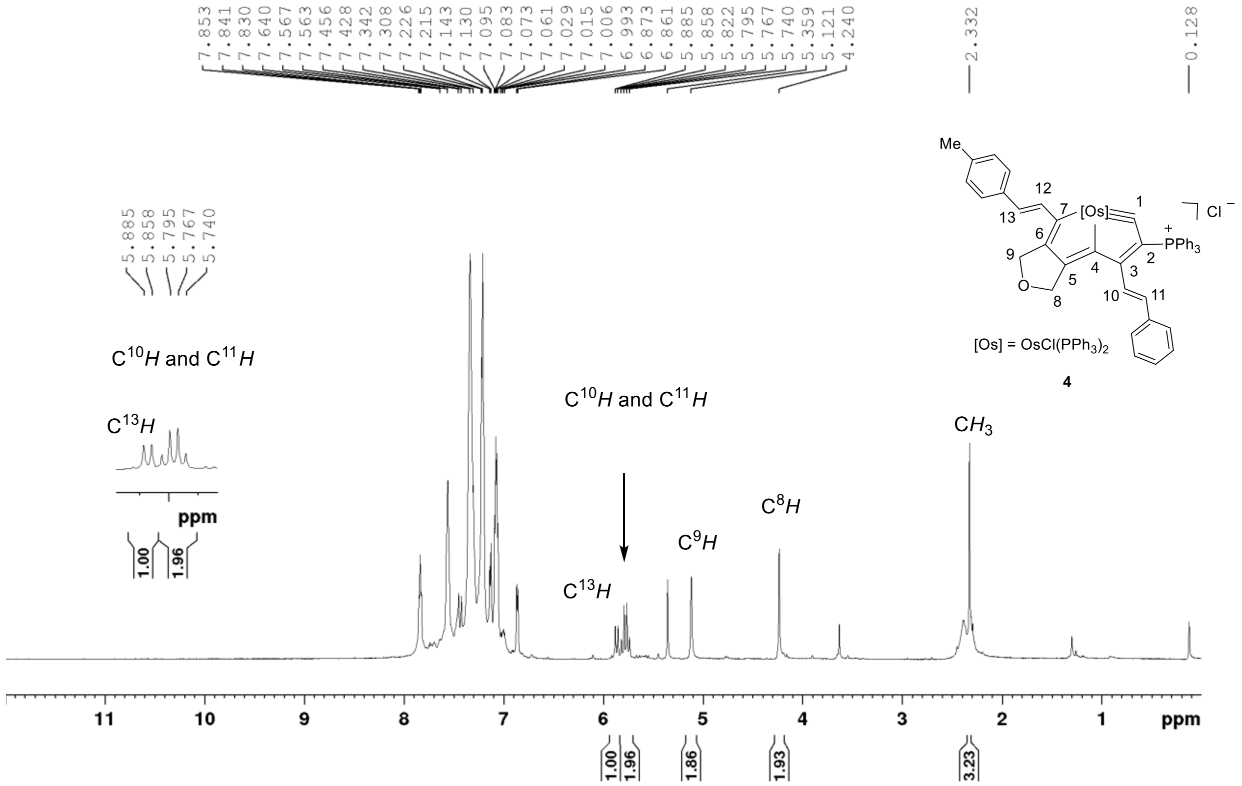

Supplementary Figure 72. The ^{1}H NMR (600.1 MHz, CD_2Cl_2) spectrum for complex **4**.

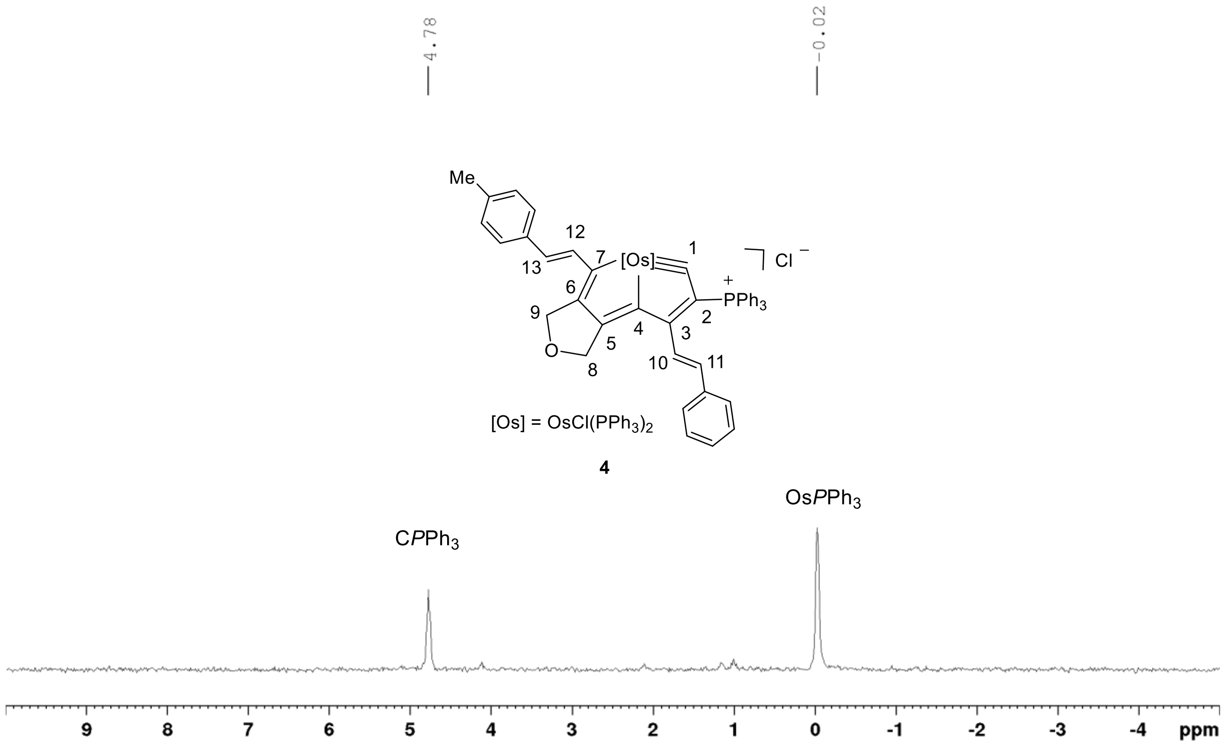

Supplementary Figure 73. The ^{31}P{^{1}H} NMR (242.9 MHz, CD_2Cl_2) spectrum for complex **4**.

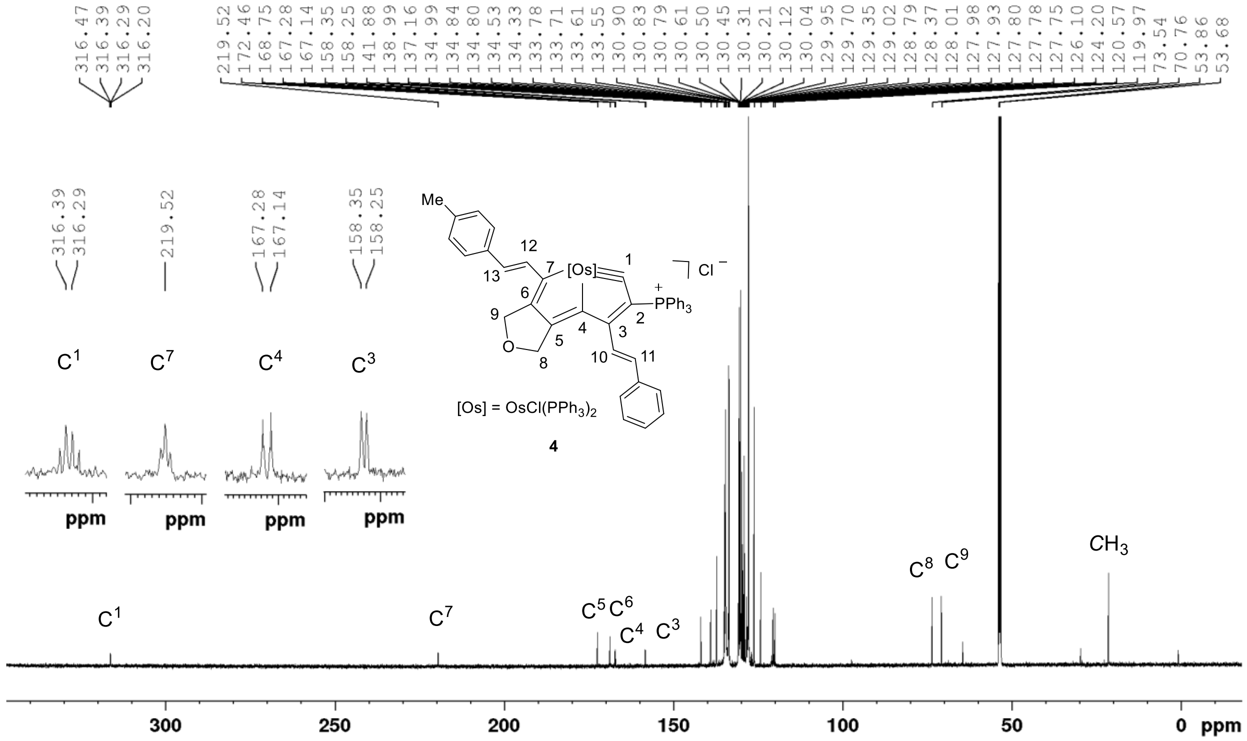

Supplementary Figure 74. The $^{13}C\{^{1}H\}$ NMR (150.9 MHz, CD_2Cl_2) spectrum for complex **4**.

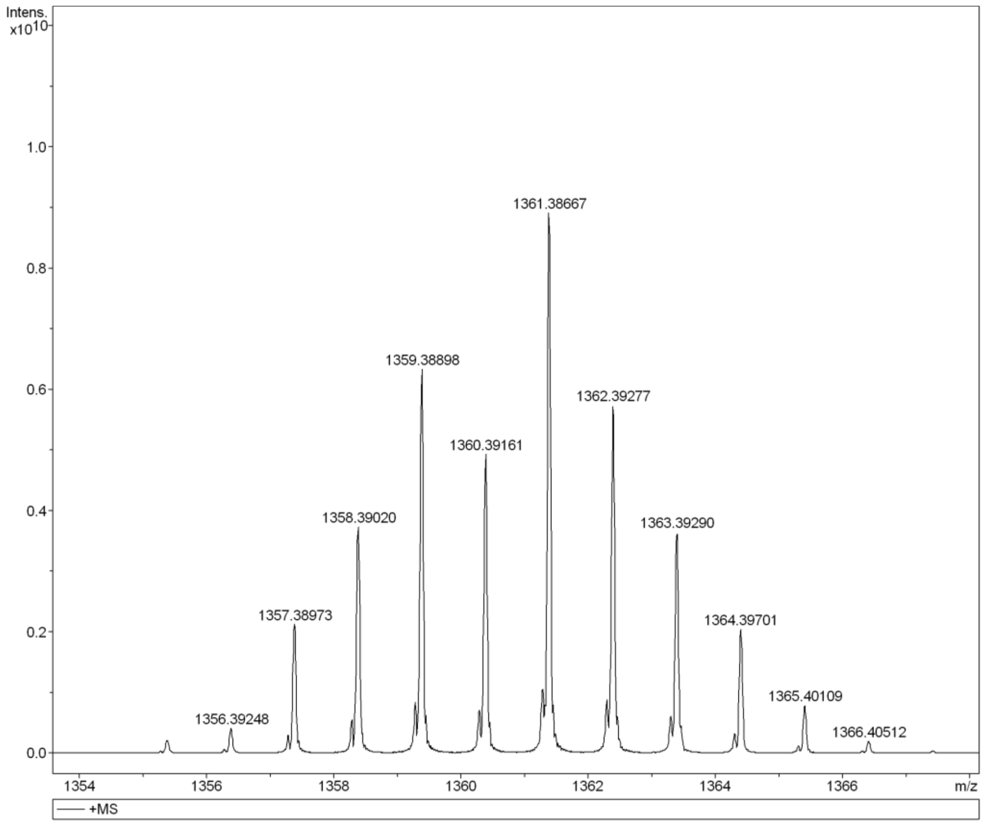

Supplementary Figure 75. Positive-ion ESI-MS spectrum of [**4**]$^{+}$ measured in methanol.

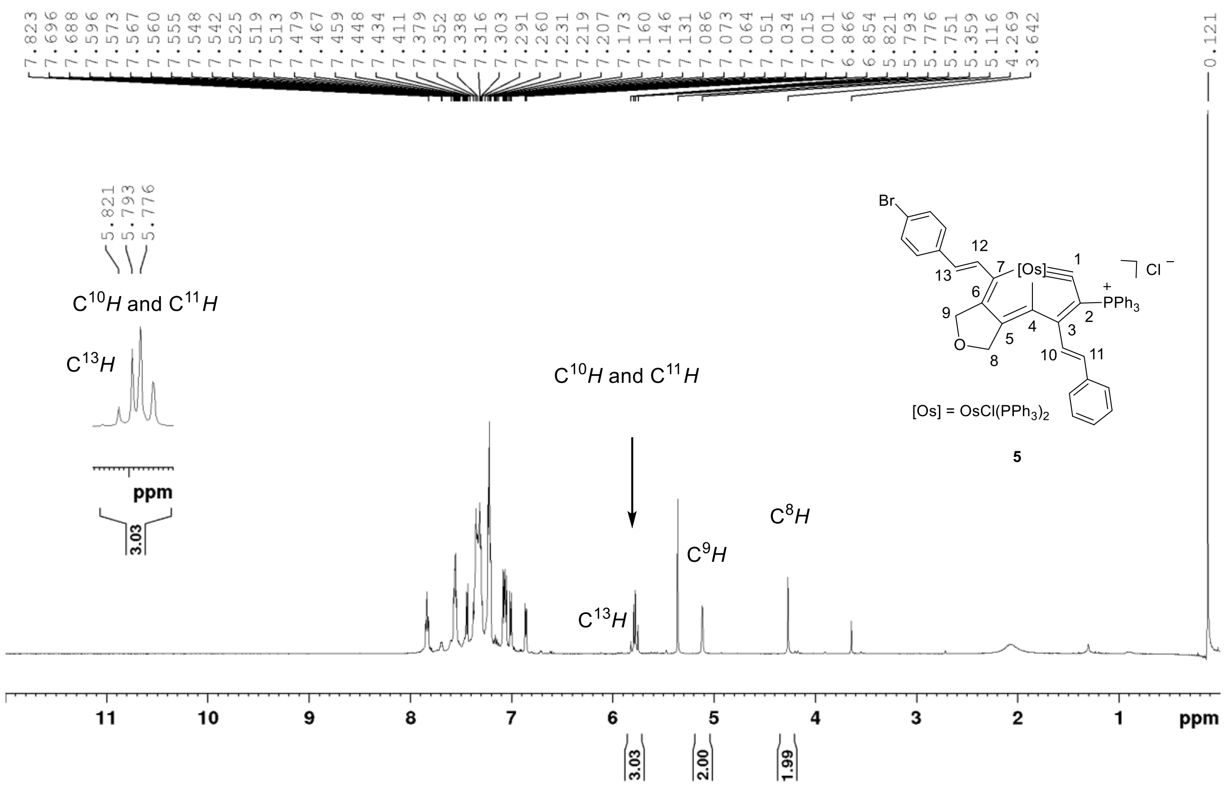

Supplementary Figure 76. The ^{1}H NMR (600.1 MHz, CD_2Cl_2) spectrum for complex **5**.

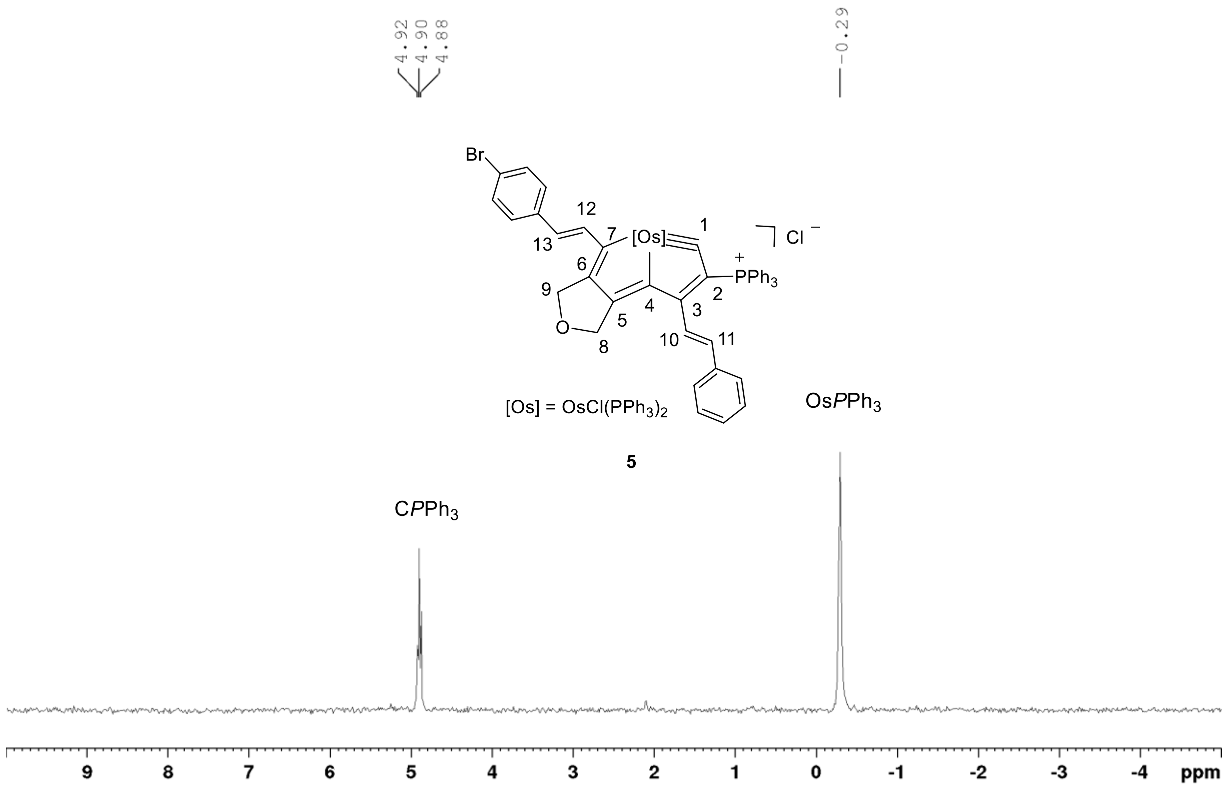

Supplementary Figure 77. The ^{31}P{^{1}H} NMR (242.9 MHz, CD_2Cl_2) spectrum for complex **5**.

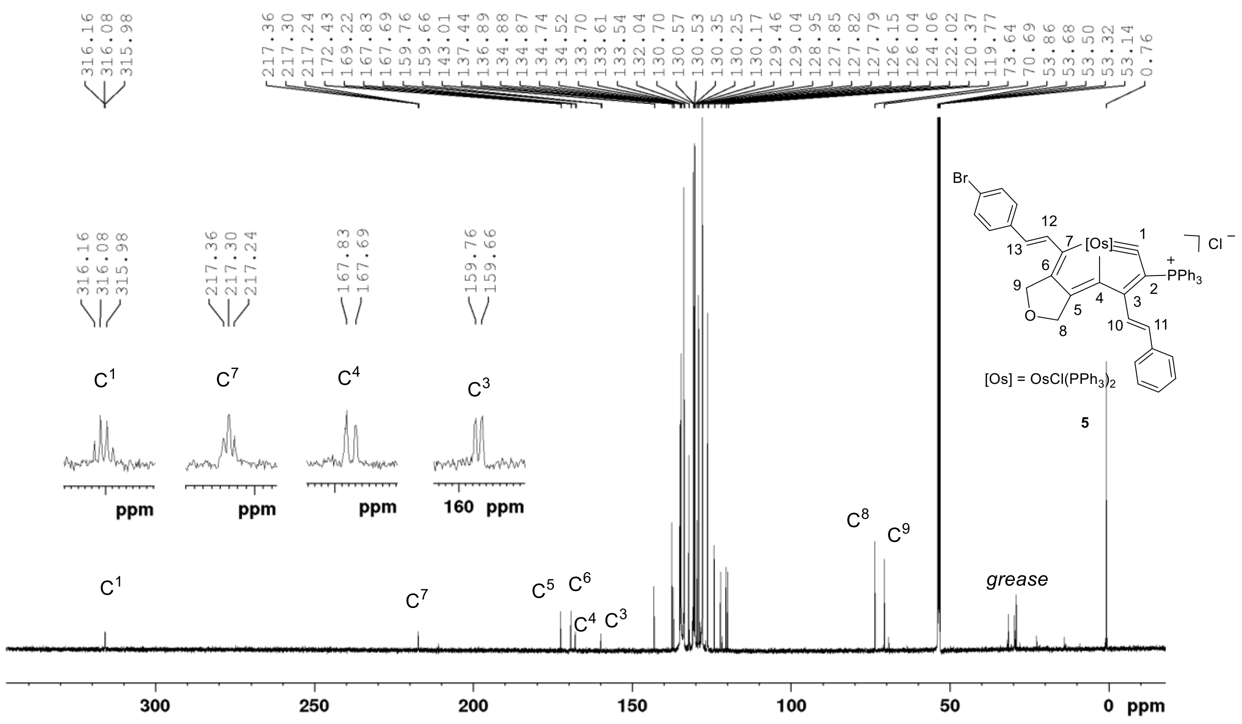

Supplementary Figure 78. The $^{13}C\{^1H\}$ NMR (150.9 MHz, CD_2Cl_2) spectrum for complex **5**.

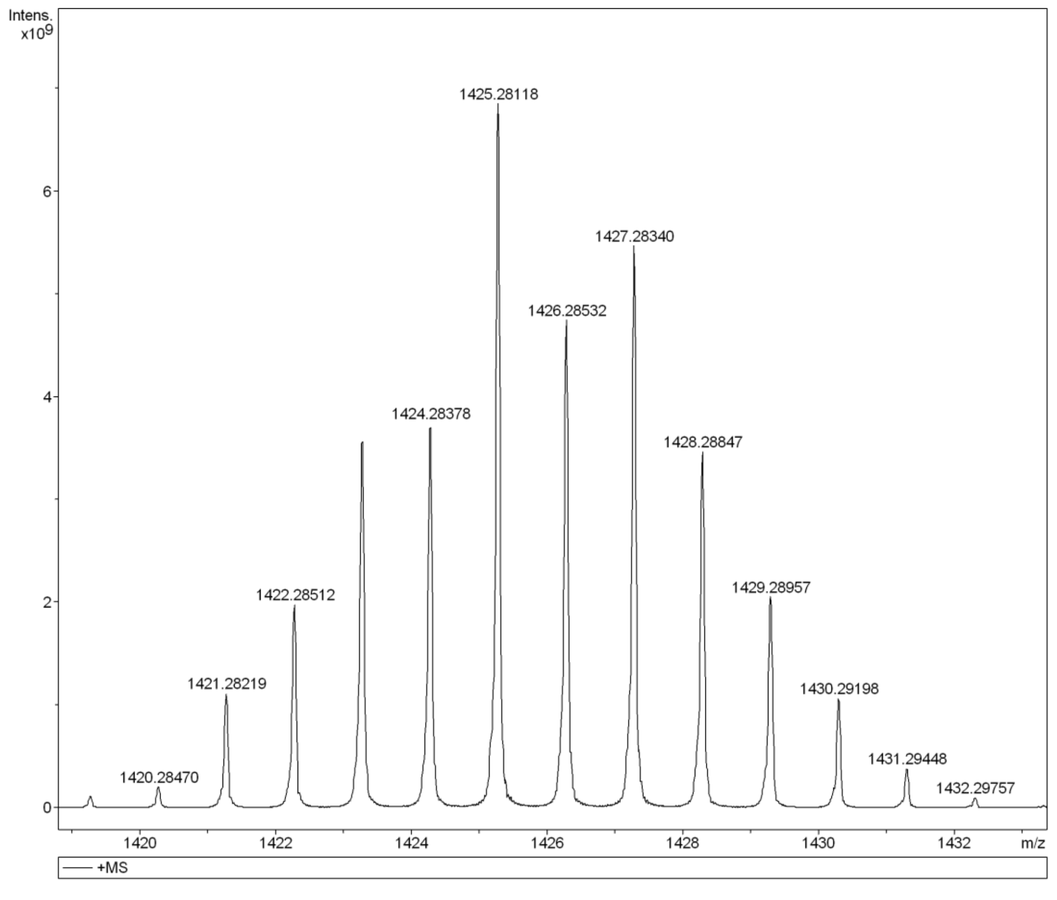

Supplementary Figure 79. Positive-ion ESI-MS spectrum of [**5**]$^+$ measured in methanol.

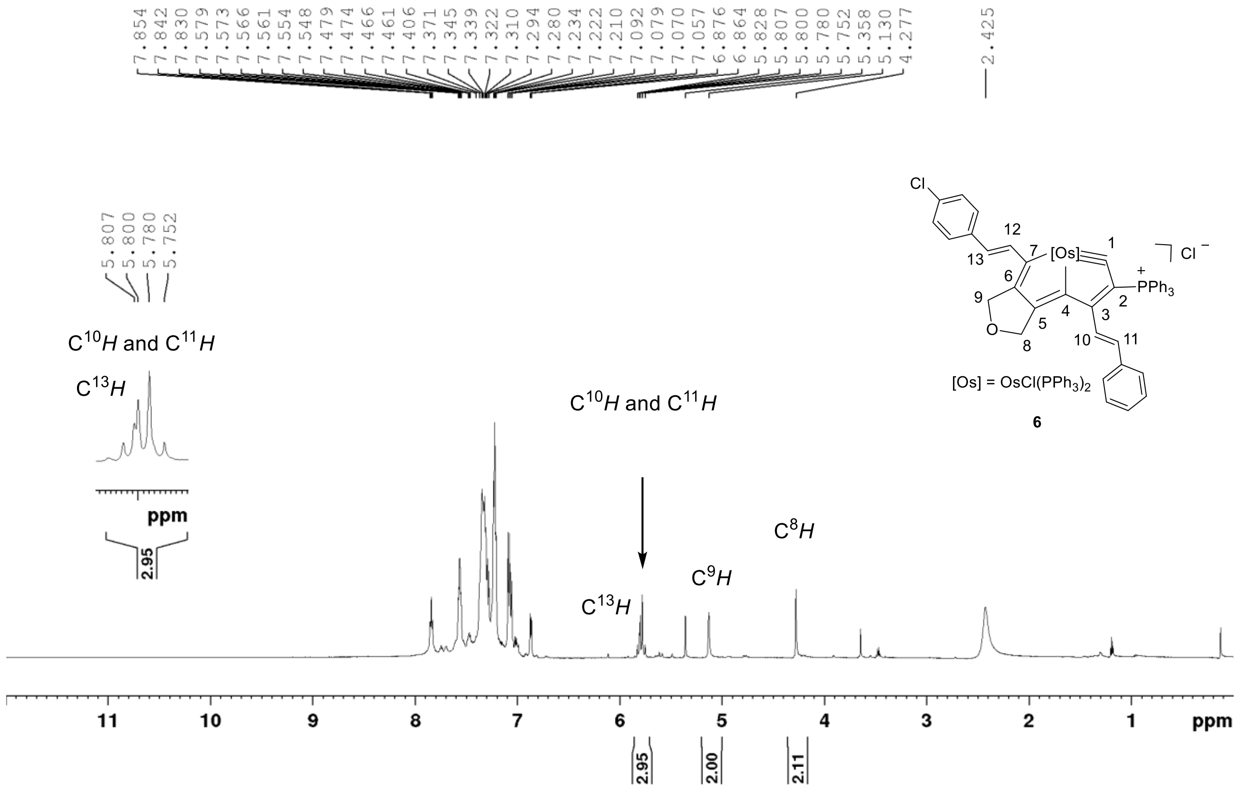

Supplementary Figure 80. The ^{1}H NMR (600.1 MHz, CD_2Cl_2) spectrum for complex **6**.

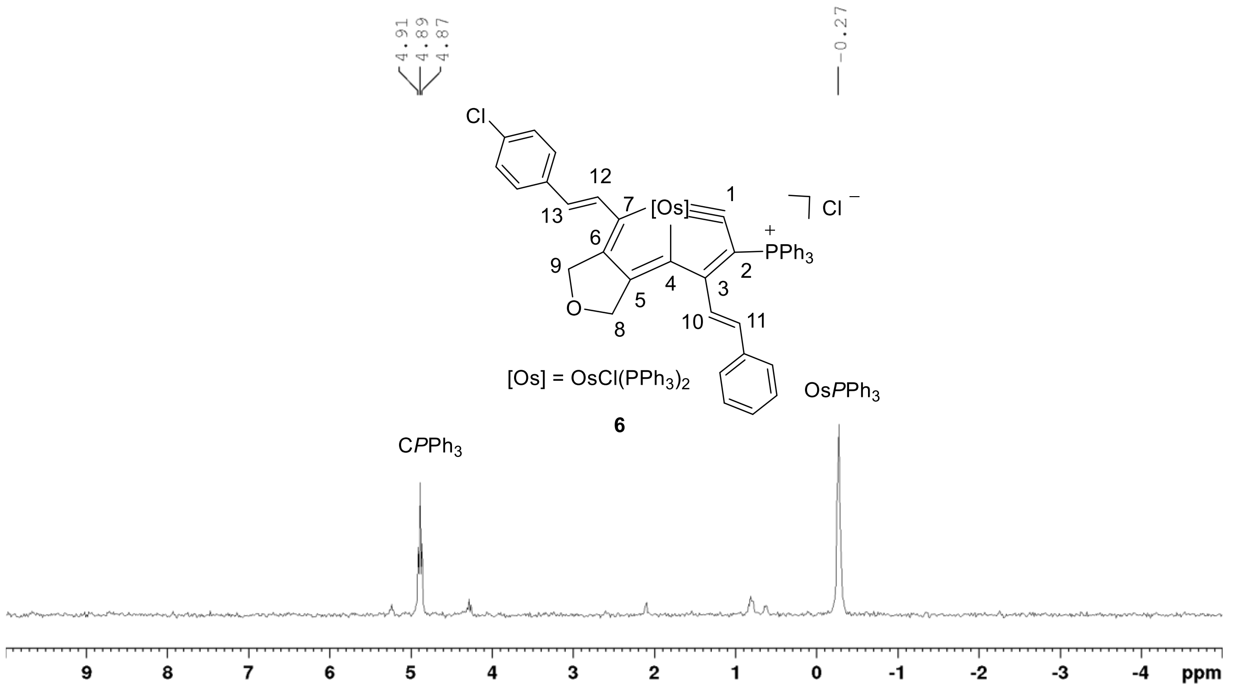

Supplementary Figure 81. The ^{31}P{^{1}H} NMR (242.9 MHz, CD_2Cl_2) spectrum for complex **6**.

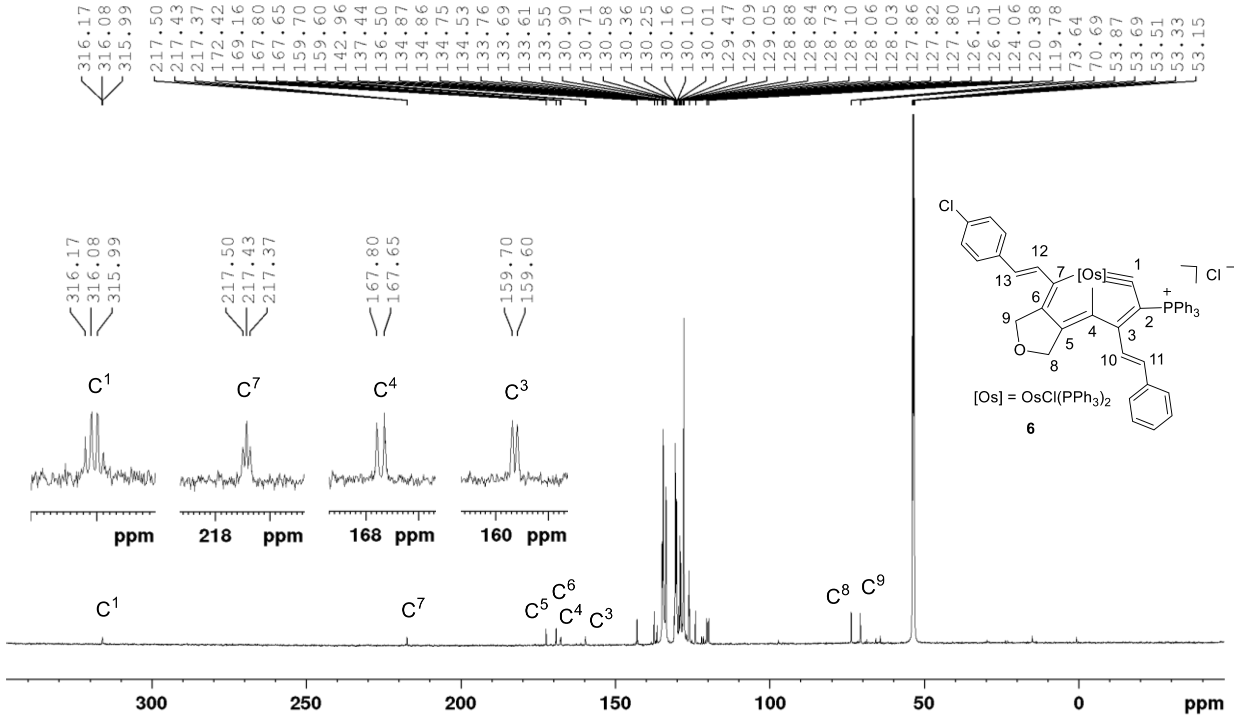

Supplementary Figure 82. The $^{13}C\{^1H\}$ NMR (150.9 MHz, CD_2Cl_2) spectrum for complex **6**.

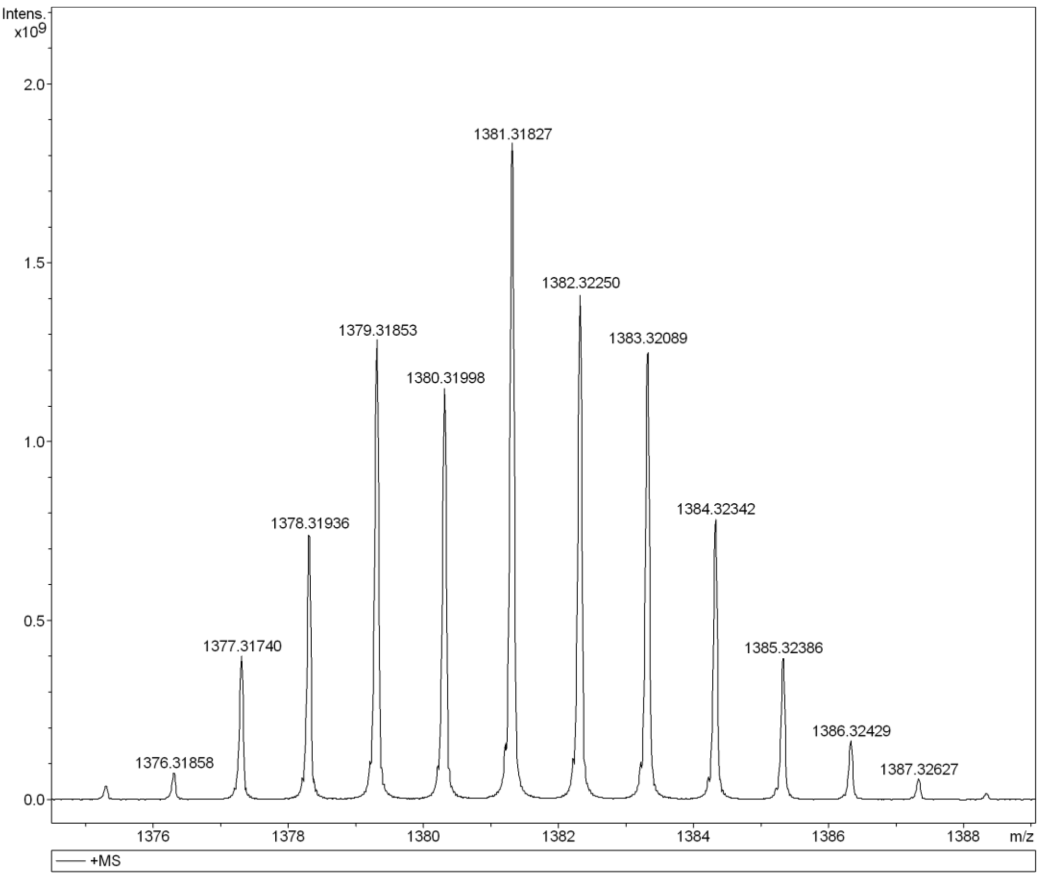

Supplementary Figure 83. Positive-ion ESI-MS spectrum of $[\mathbf{6}]^+$ measured in methanol.

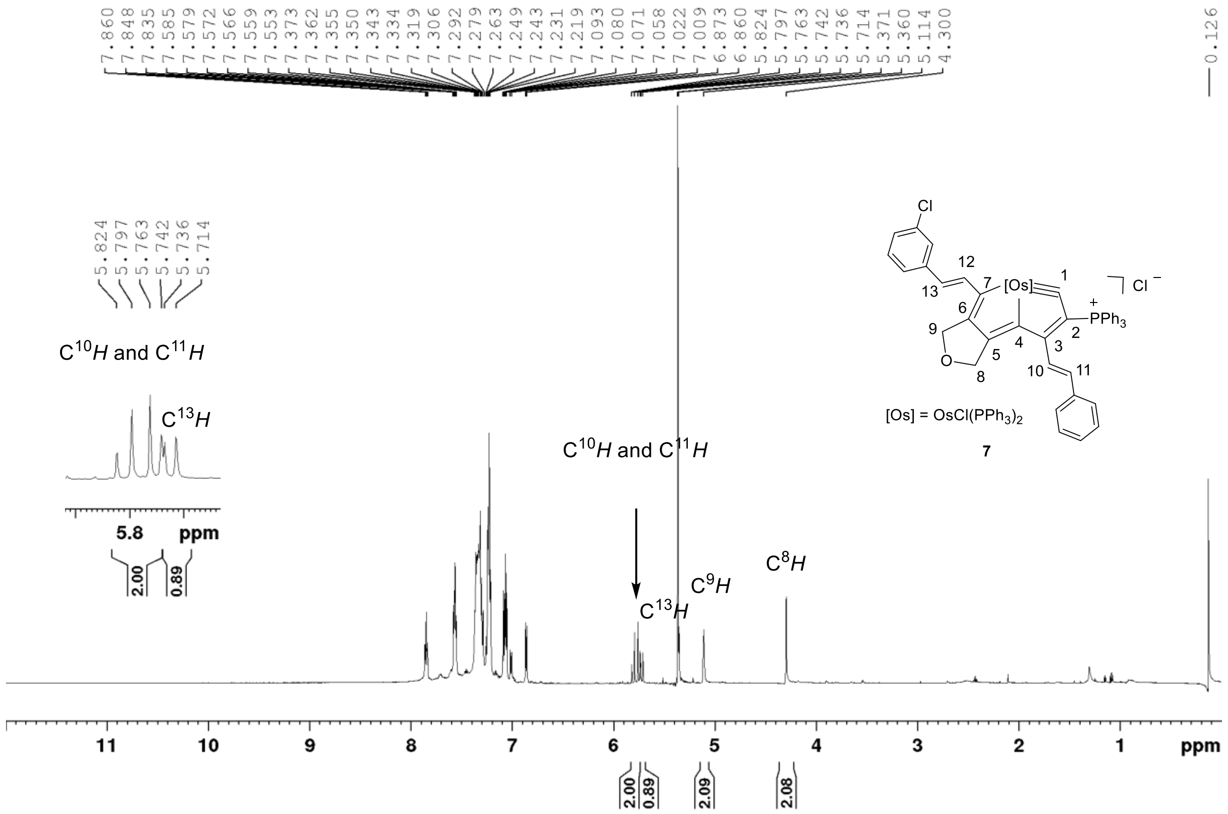

Supplementary Figure 84. The ^{1}H NMR (600.1 MHz, CD_2Cl_2) spectrum for complex **7**.

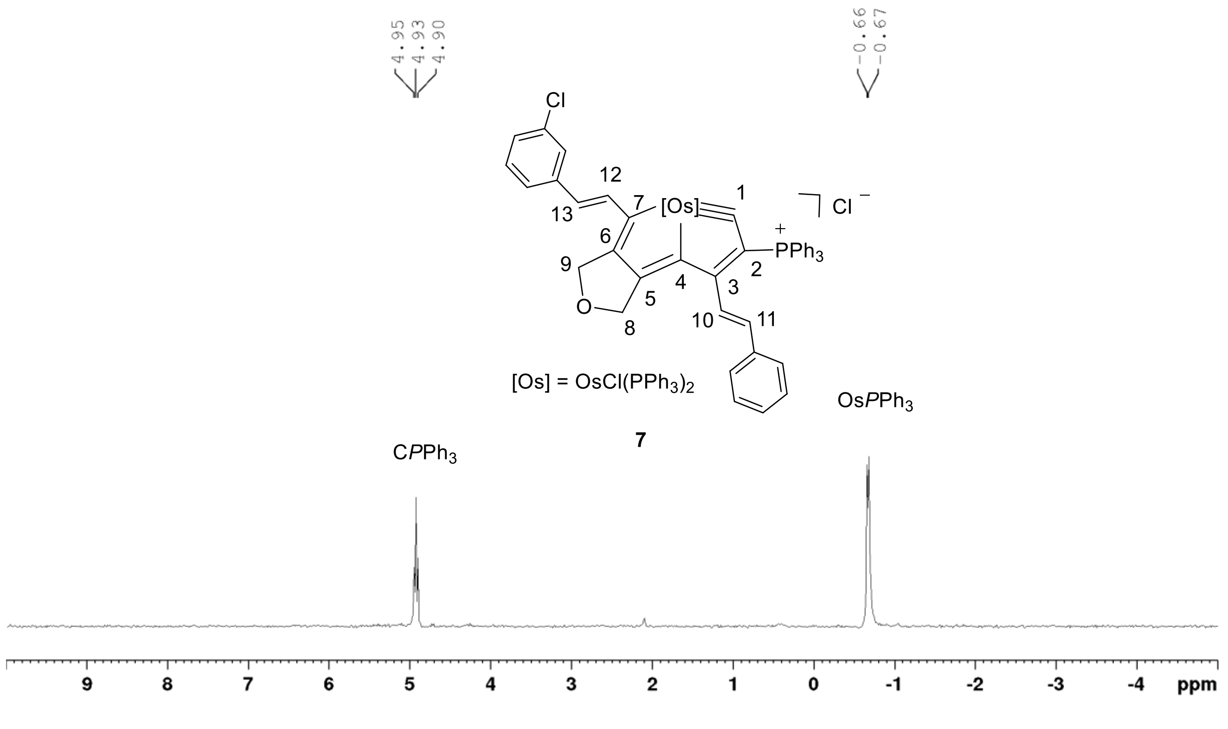

Supplementary Figure 85. The ^{31}P{^{1}H} NMR (242.9 MHz, CD_2Cl_2) spectrum for complex **7**.

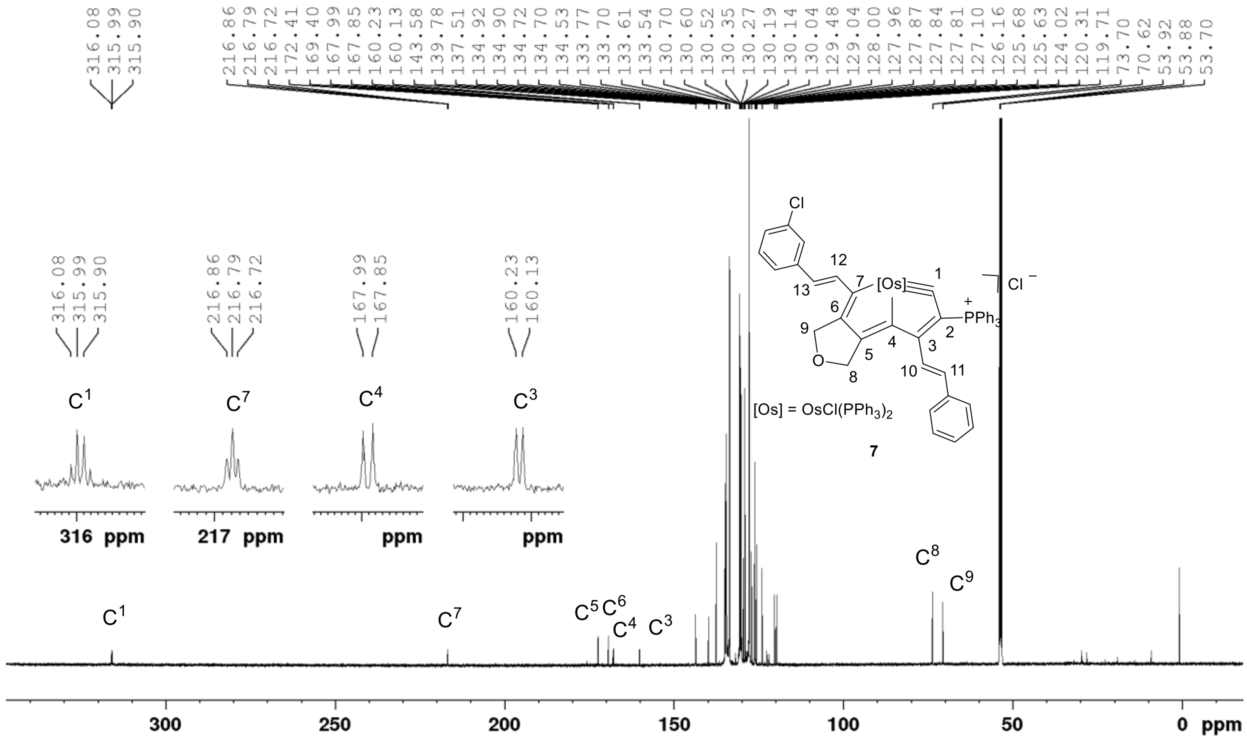

Supplementary Figure 86. The $^{13}C\{^{1}H\}$ NMR (150.9 MHz, CD_2Cl_2) spectrum for complex **7**.

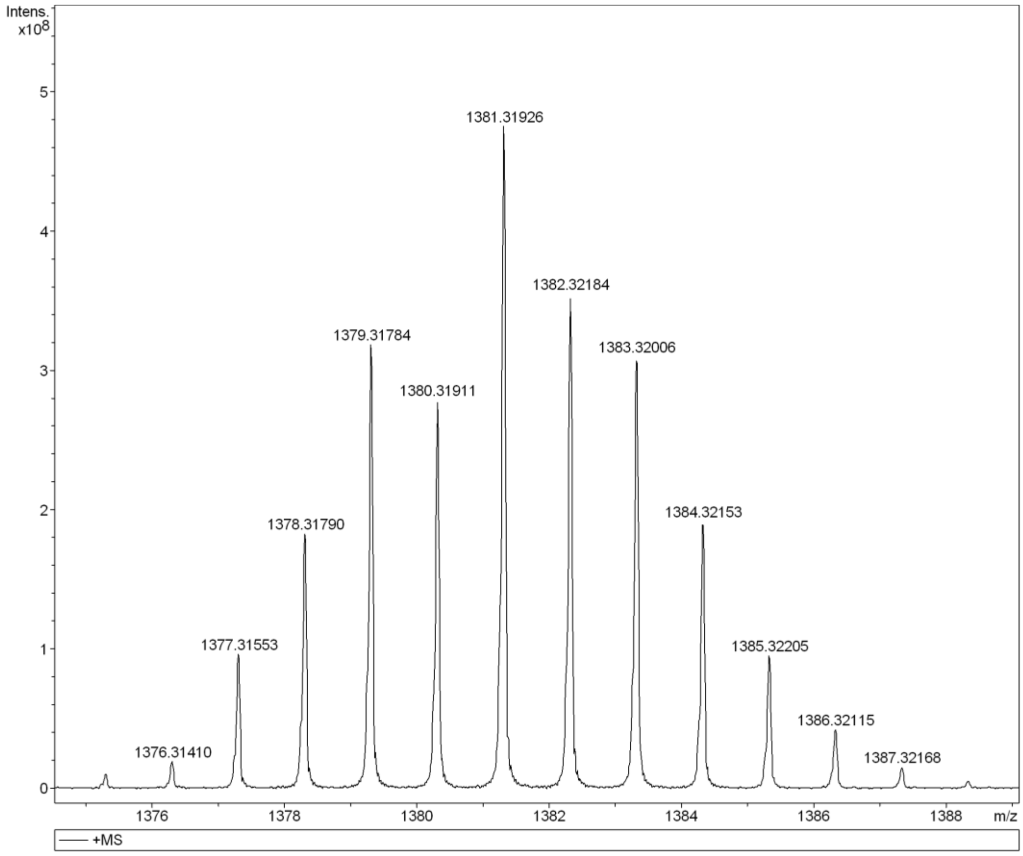

Supplementary Figure 87. Positive-ion ESI-MS spectrum of $[\mathbf{7}]^+$ measured in methanol.

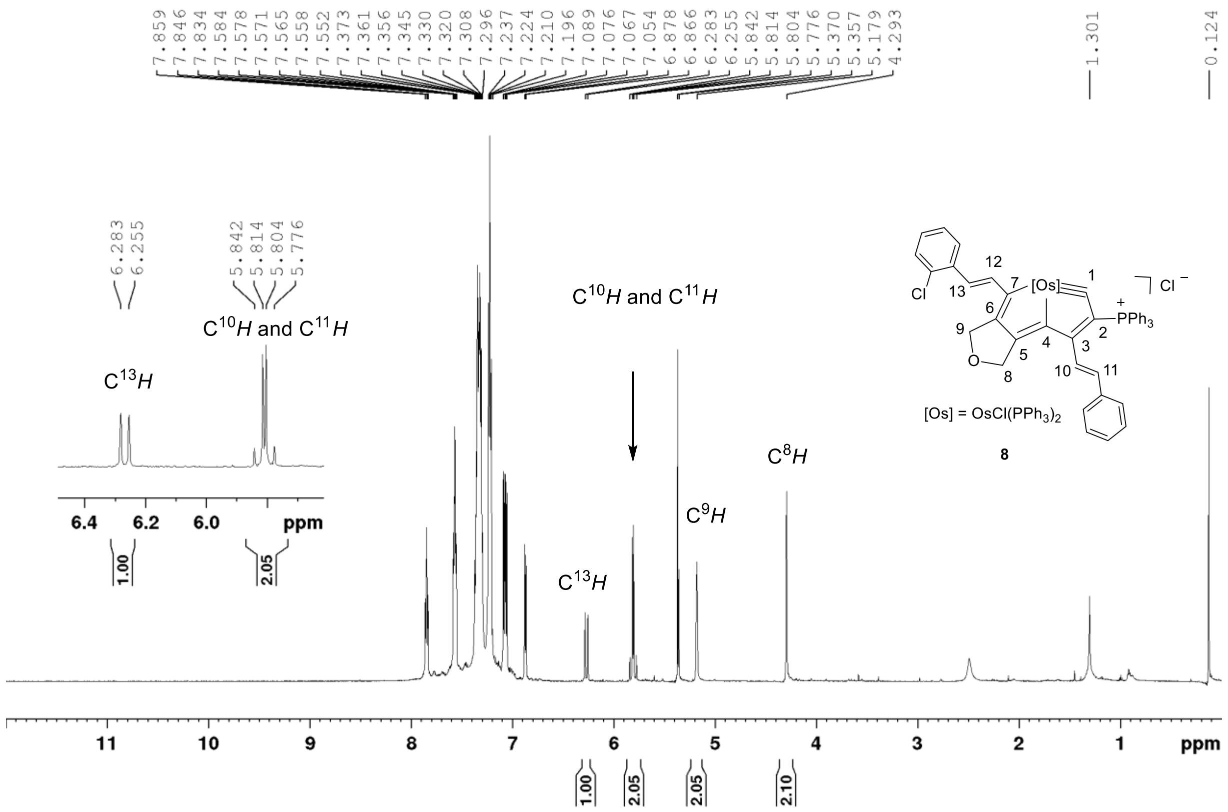

Supplementary Figure 88. The ^{1}H NMR (600.1 MHz, CD_2Cl_2) spectrum for complex **8**.

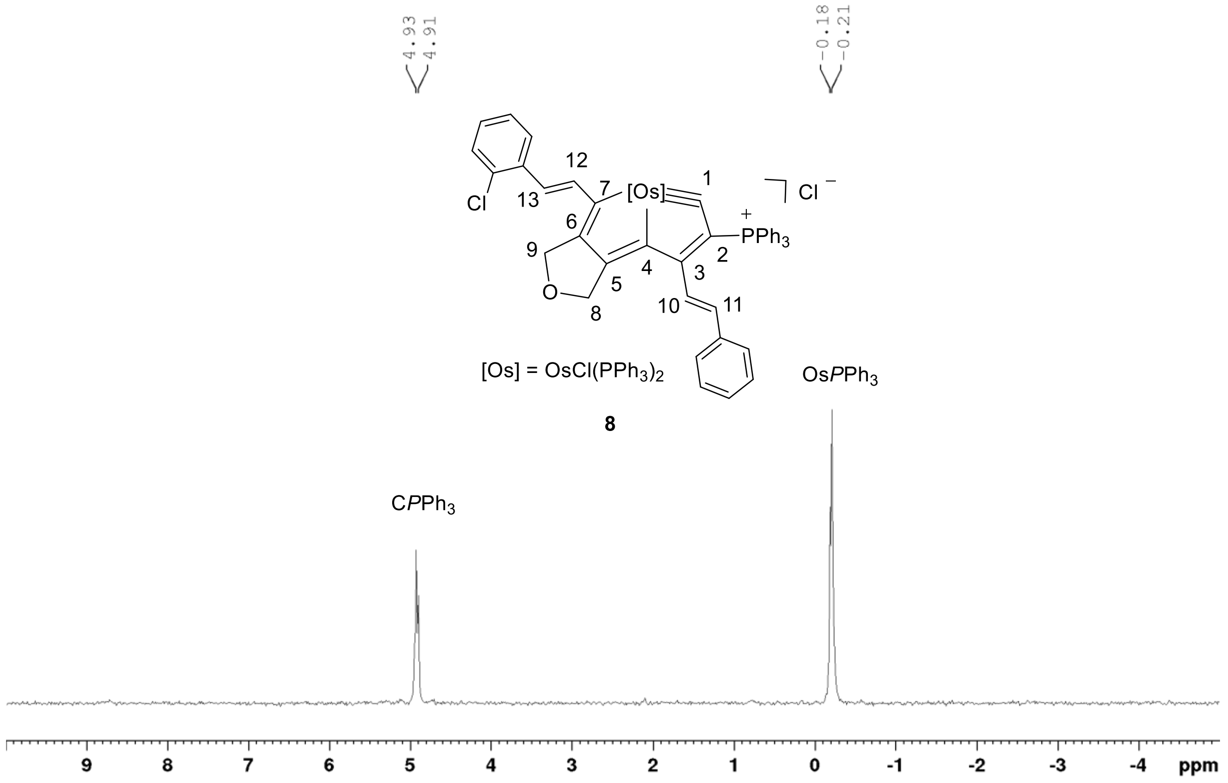

Supplementary Figure 89. The $^{31}P\{^1H\}$ NMR (242.9 MHz, CD_2Cl_2) spectrum for complex **8**.

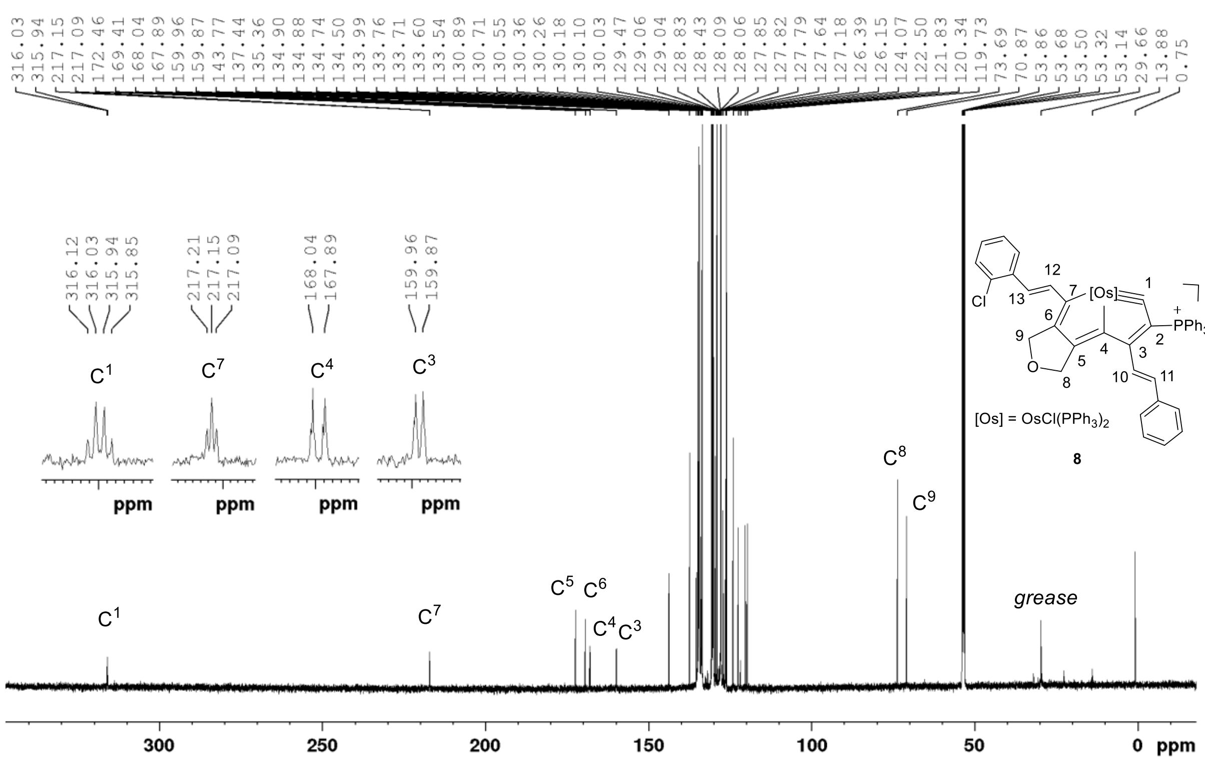

Supplementary Figure 90. The $^{13}C\{^1H\}$ NMR (150.9 MHz, CD_2Cl_2) spectrum for complex **8**.

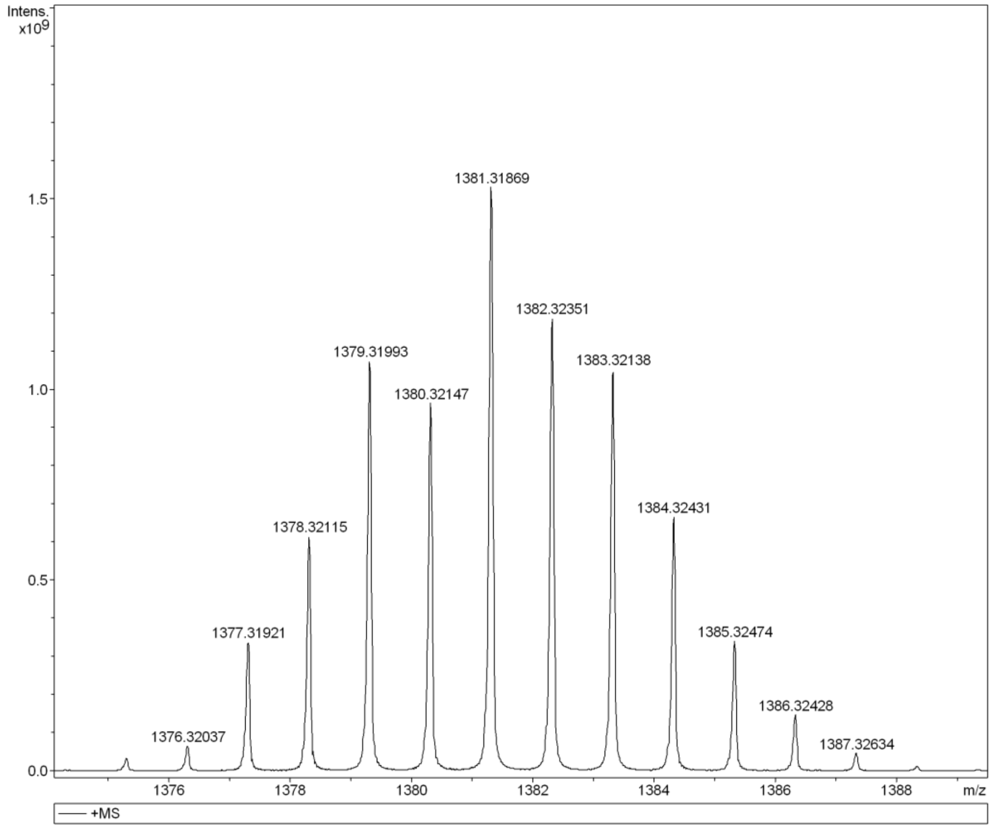

Supplementary Figure 91. Positive-ion ESI-MS spectrum of [**8**]$^+$ measured in methanol.

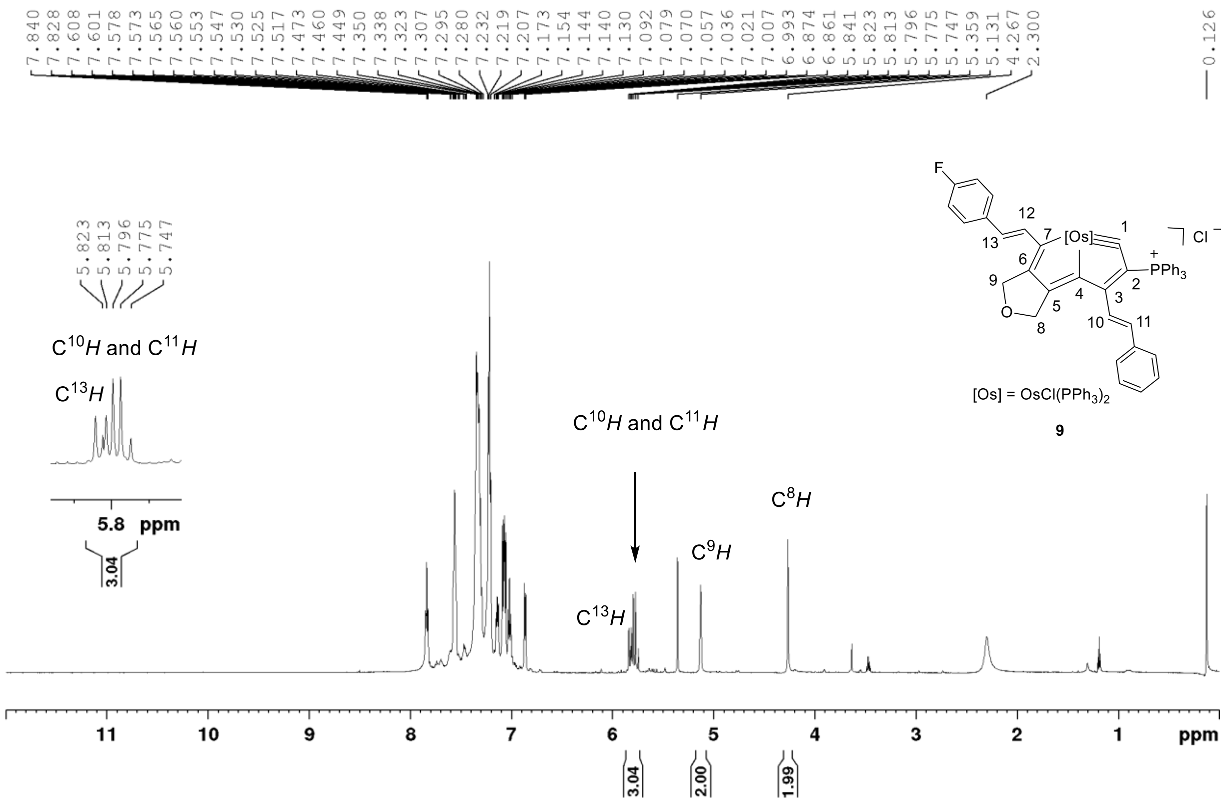

Supplementary Figure 92. The ^{1}H NMR (600.1 MHz, CD_2Cl_2) spectrum for complex **9**.

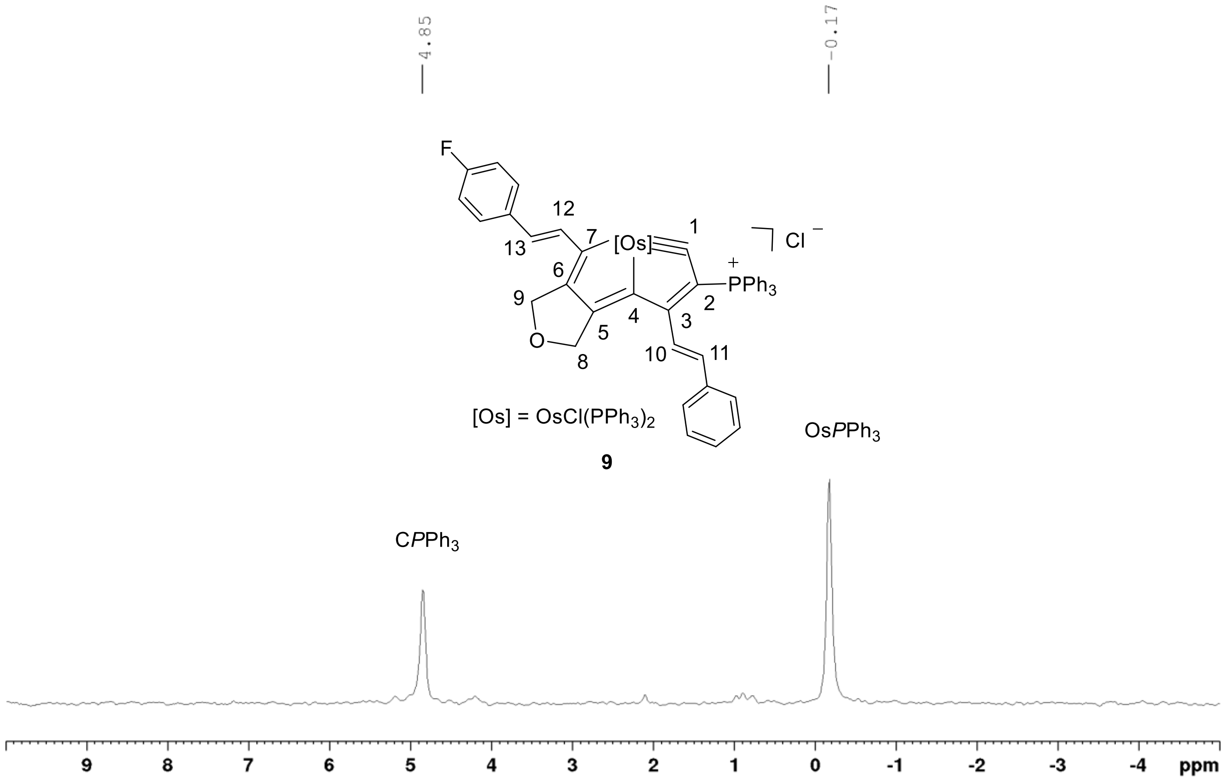

Supplementary Figure 93. The $^{31}P\{^{1}H\}$ NMR (242.9 MHz, CD_2Cl_2) spectrum for complex **9**.

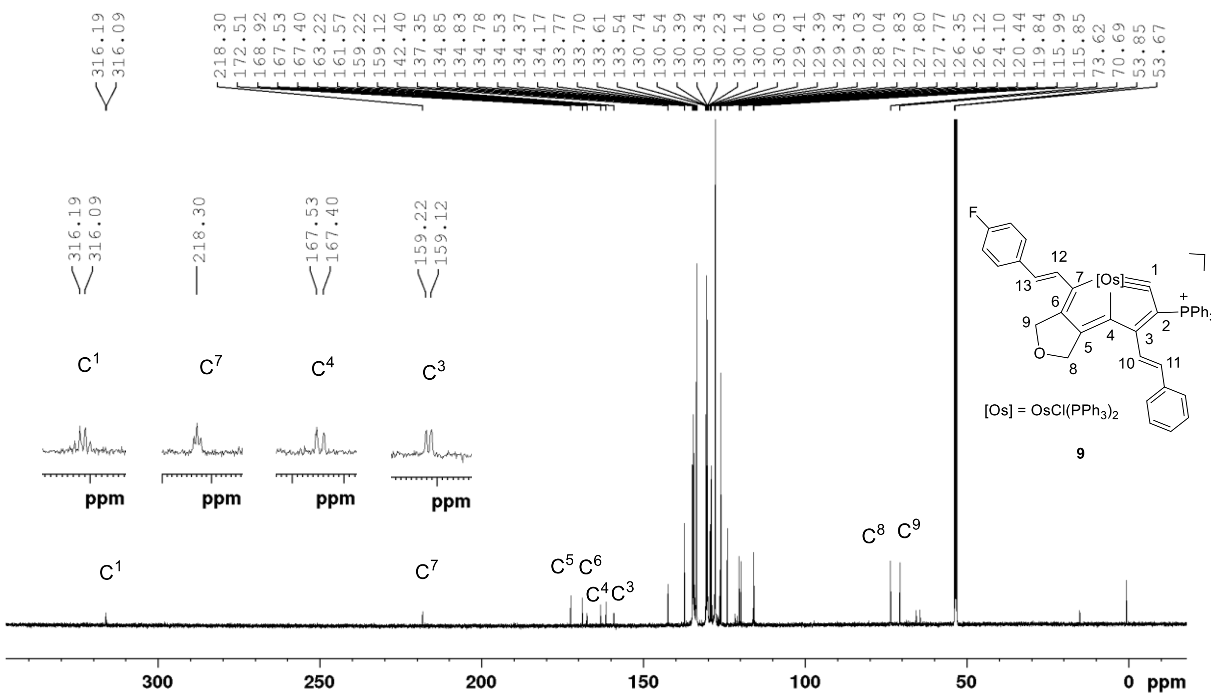

Supplementary Figure 94. The $^{13}C\{^{1}H\}$ NMR (150.9 MHz, CD_2Cl_2) spectrum for complex **9**.

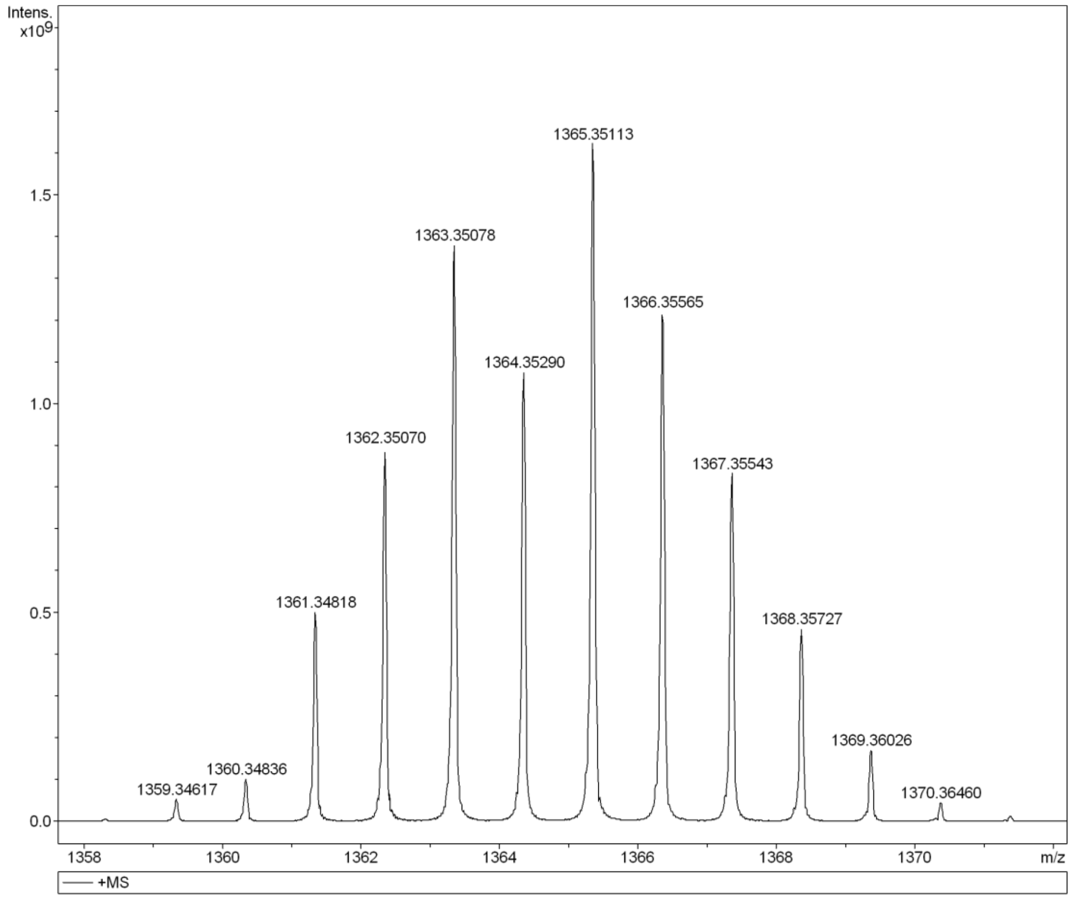

Supplementary Figure 95. Positive-ion ESI-MS spectrum of [**9**]$^{+}$ measured in methanol.

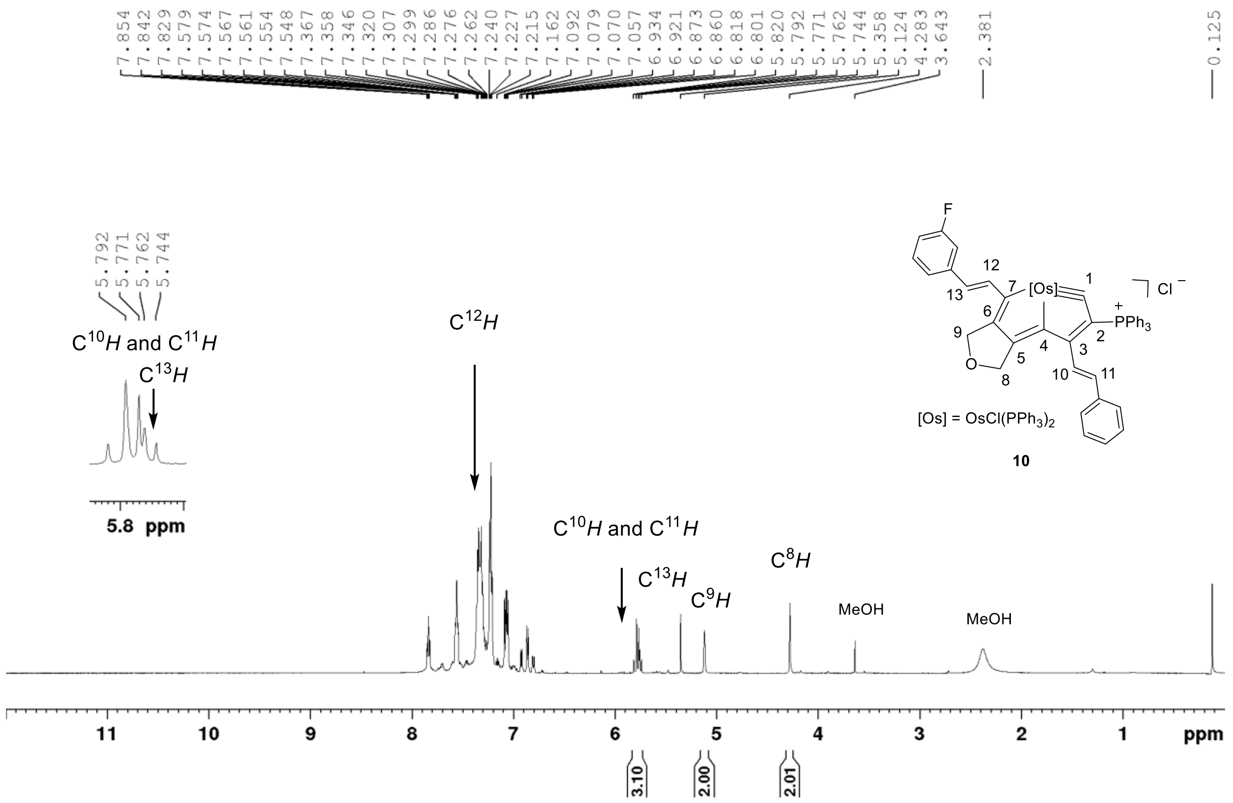

Supplementary Figure 96. The ^{1}H NMR (600.1 MHz, CD_2Cl_2) spectrum for complex **10**.

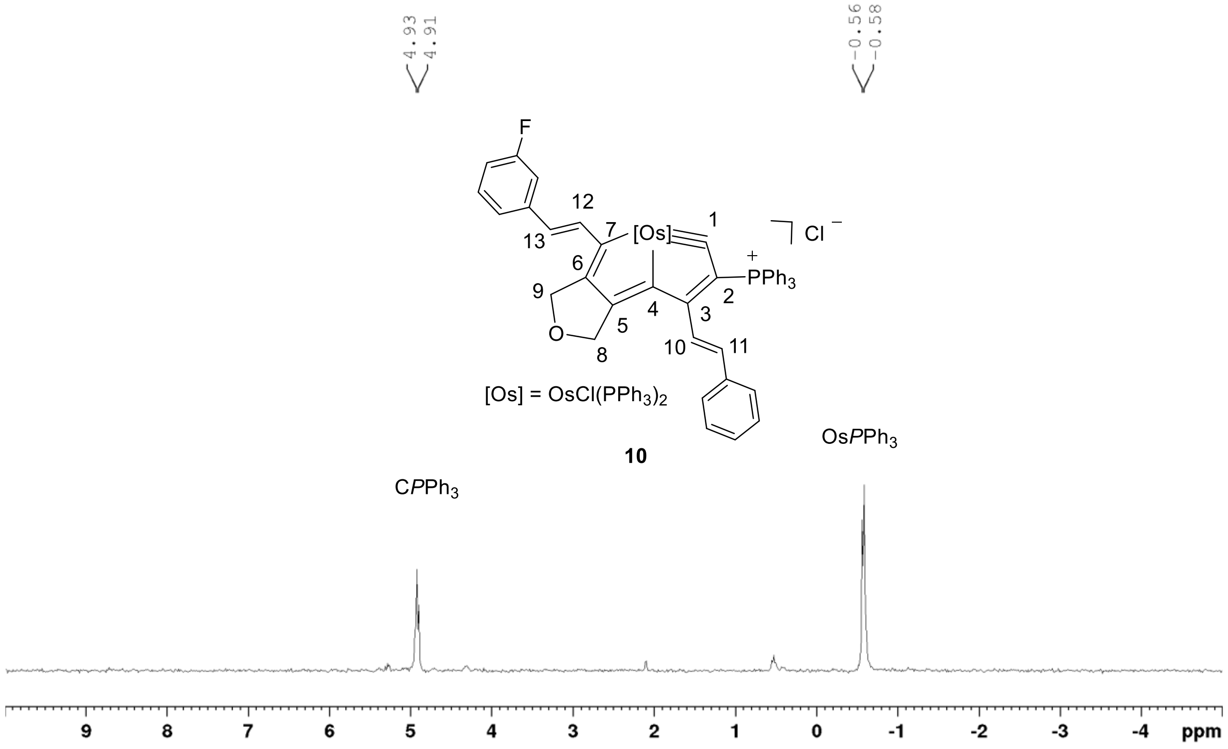

Supplementary Figure 97. The $^{31}P\{^{1}H\}$ NMR (242.9 MHz, CD_2Cl_2) spectrum for complex **10**.

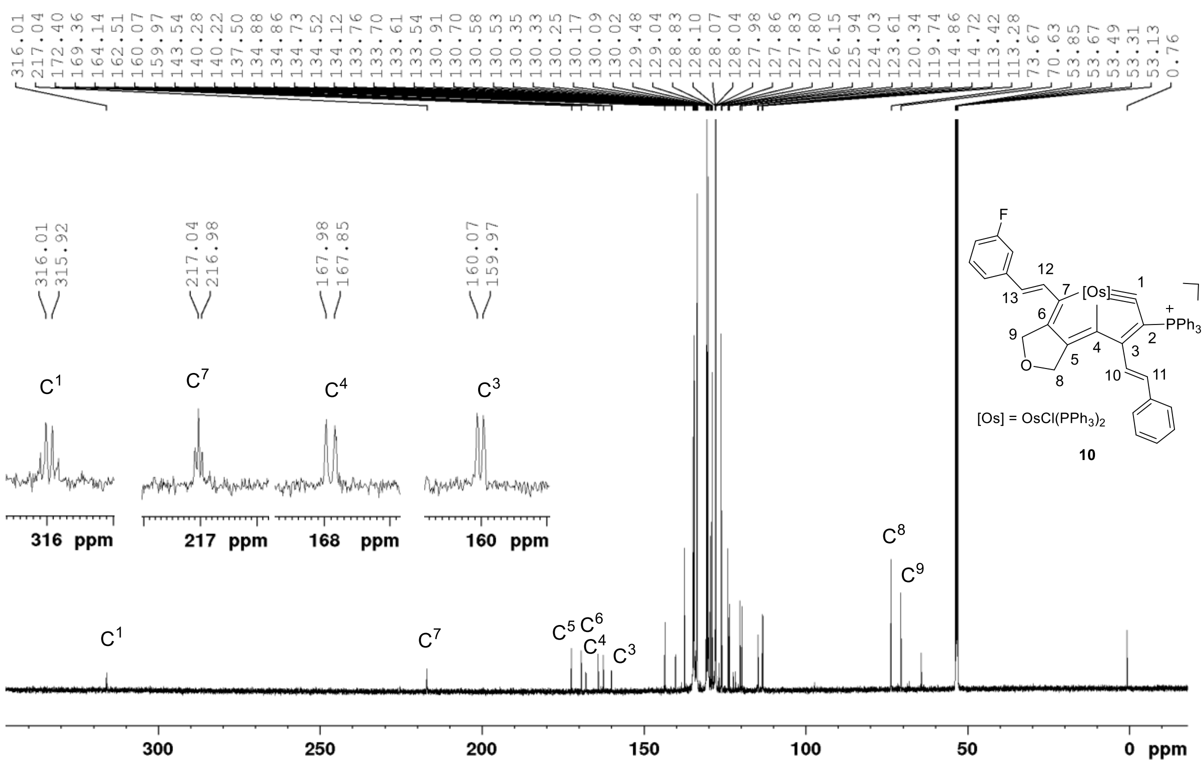

Supplementary Figure 98. The $^{13}C\{^{1}H\}$ NMR (150.9 MHz, CD_2Cl_2) spectrum for complex **10**.

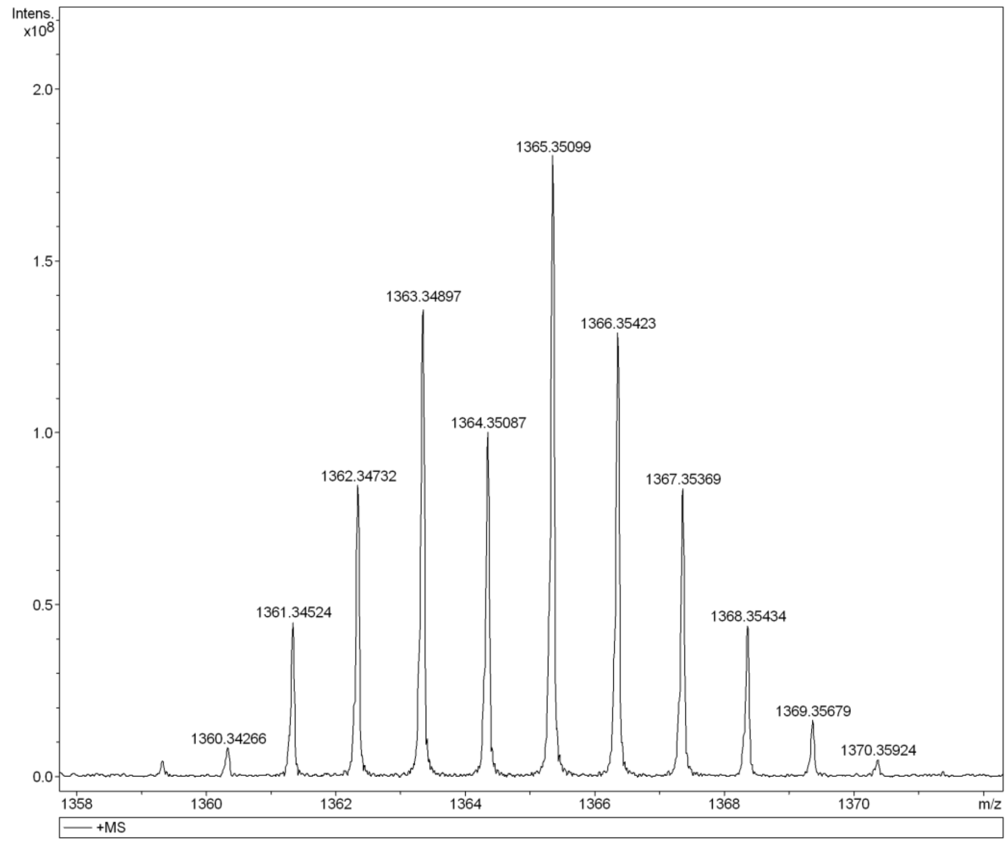

Supplementary Figure 99. Positive-ion ESI-MS spectrum of [**10**]$^+$ measured in methanol.

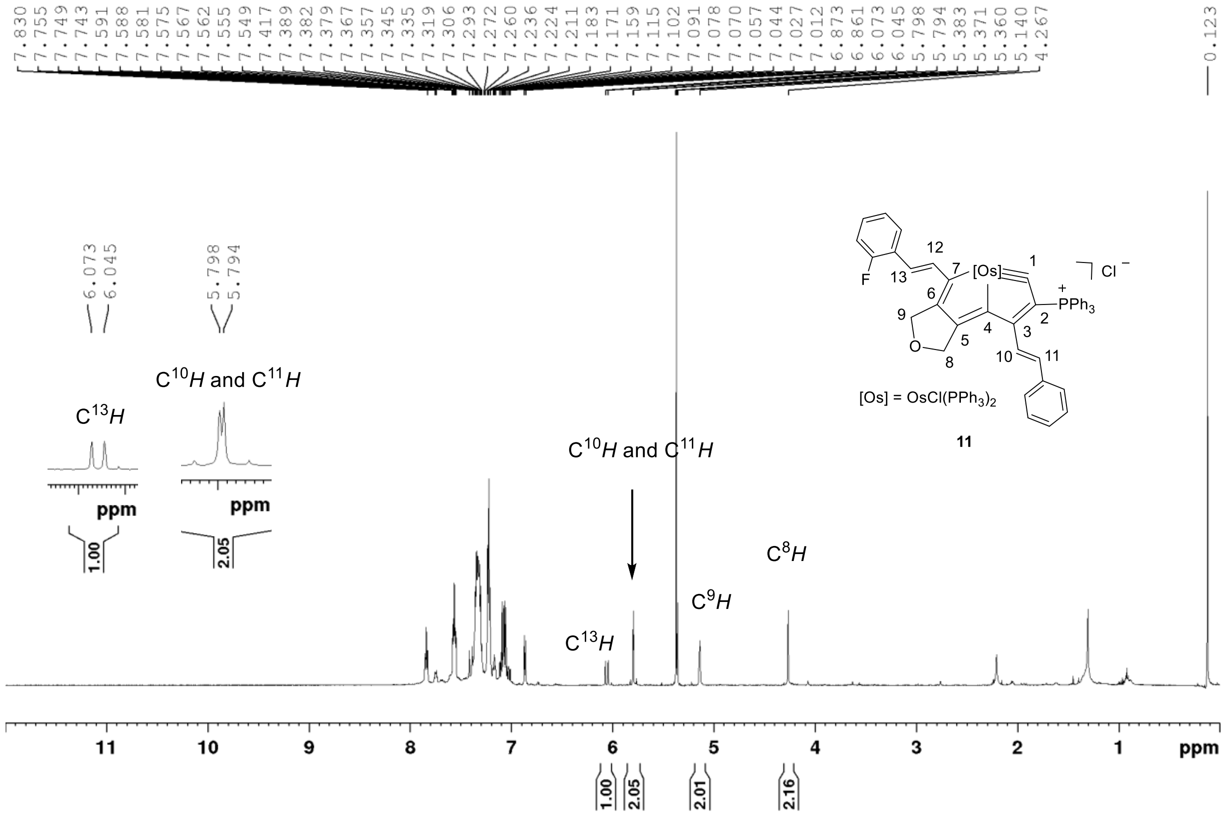

Supplementary Figure 100. The ^{1}H NMR (600.1 MHz, CD_2Cl_2) spectrum for complex **11**.

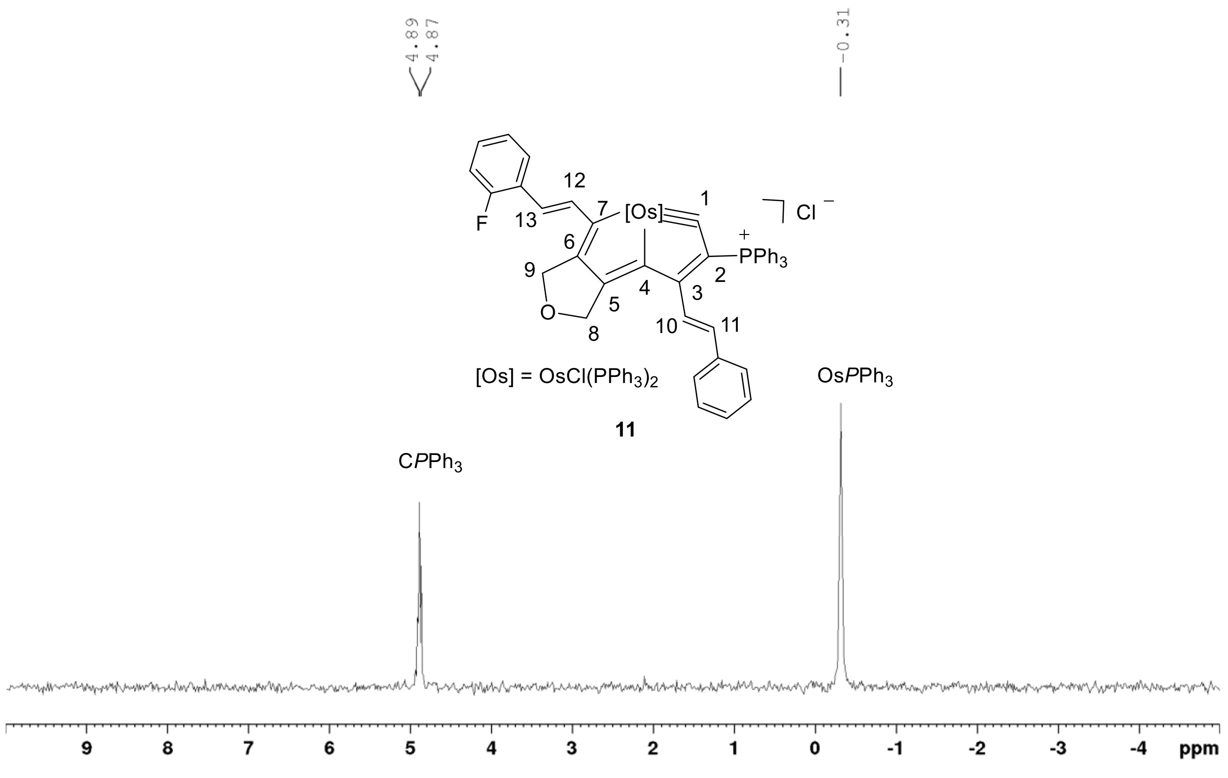

Supplementary Figure 101. The ^{31}P{^{1}H} NMR (242.9 MHz, CD_2Cl_2) spectrum for complex **11**.

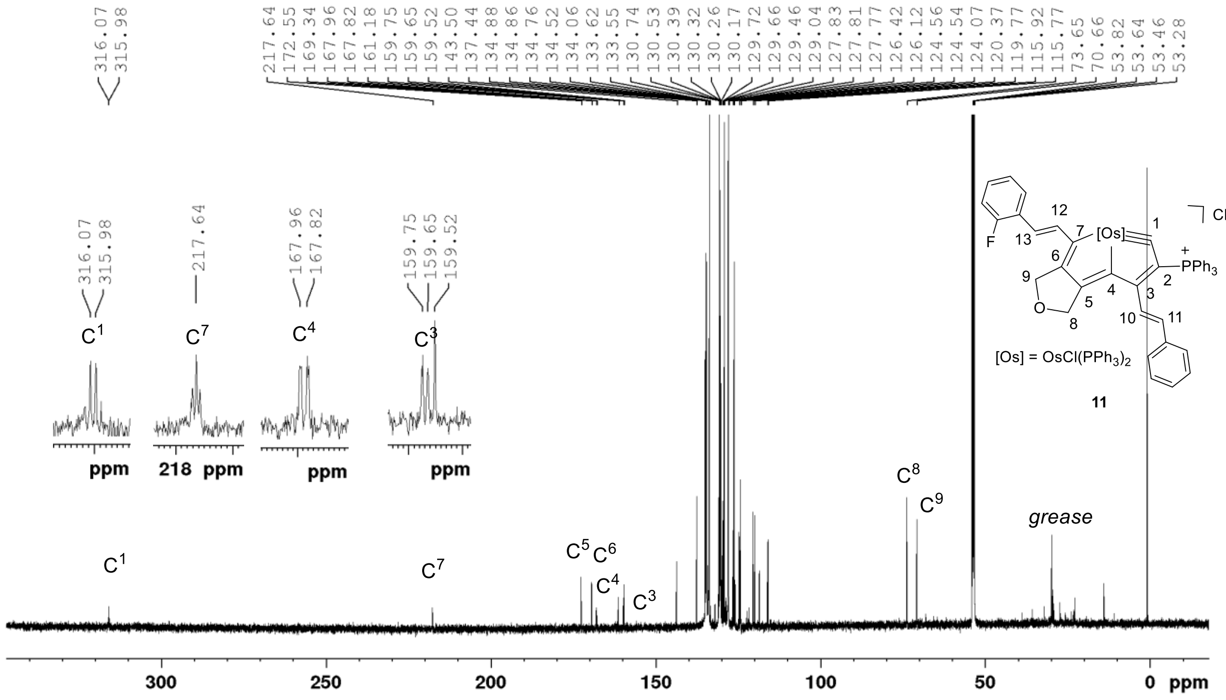

Supplementary Figure 102. The $^{13}C\{^{1}H\}$ NMR (150.9 MHz, CD_2Cl_2) spectrum for complex **11**.

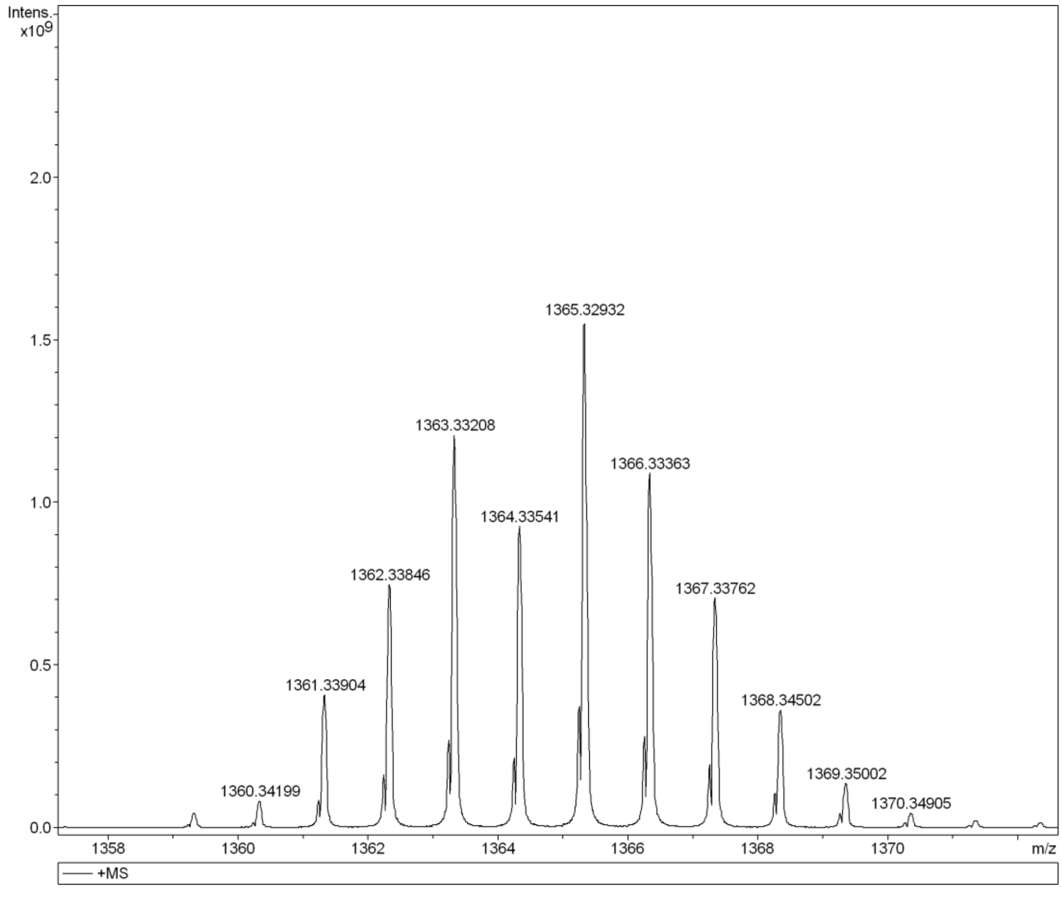

Supplementary Figure 103. Positive-ion ESI-MS spectrum of [**11**]$^+$ measured in methanol.

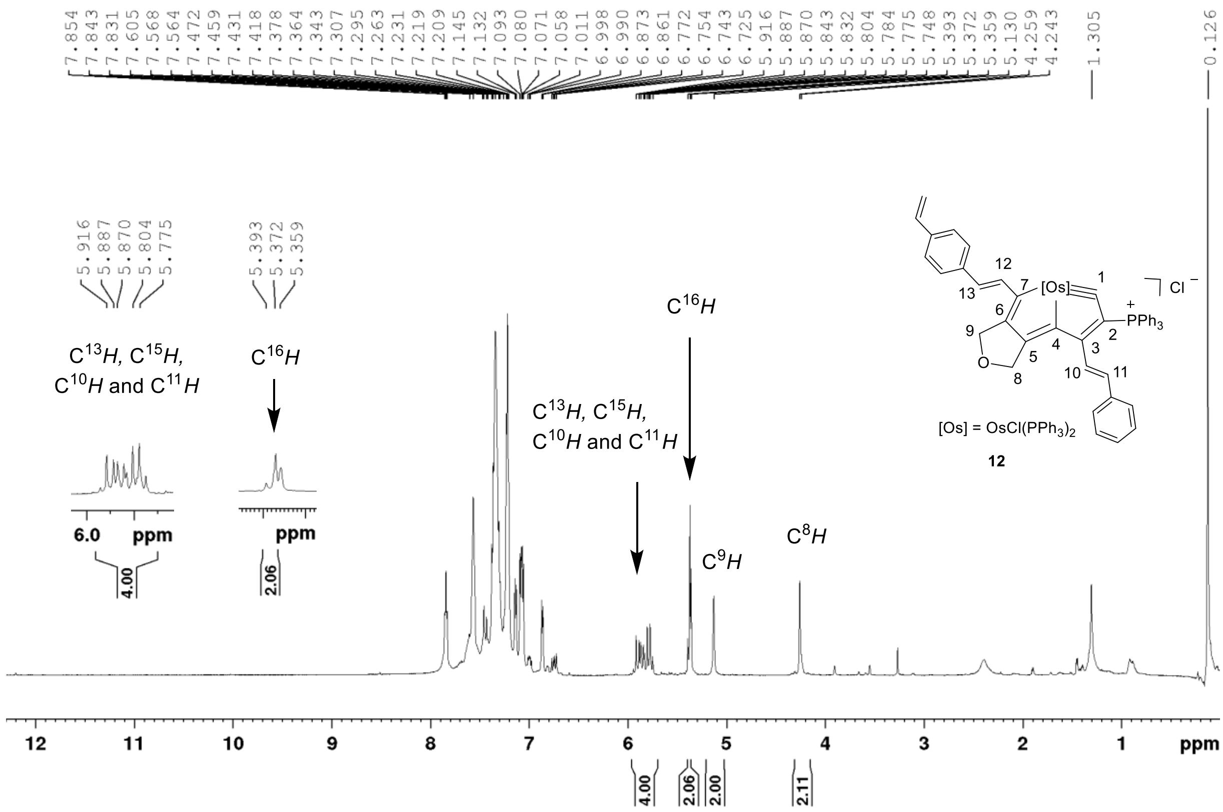

Supplementary Figure 104. The ^{1}H NMR (600.1 MHz, CD_2Cl_2) spectrum for complex **12**.

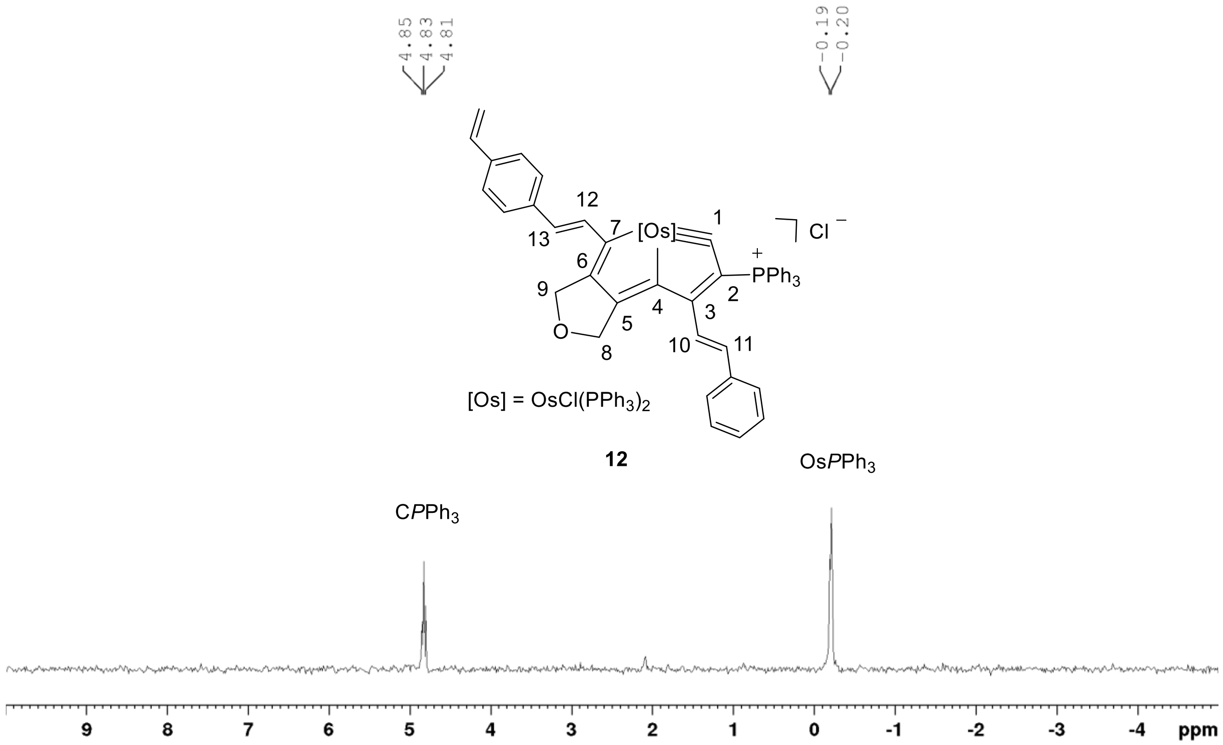

Supplementary Figure 105. The ^{31}P{^{1}H} NMR (242.9 MHz, CD_2Cl_2) spectrum for complex **12**.

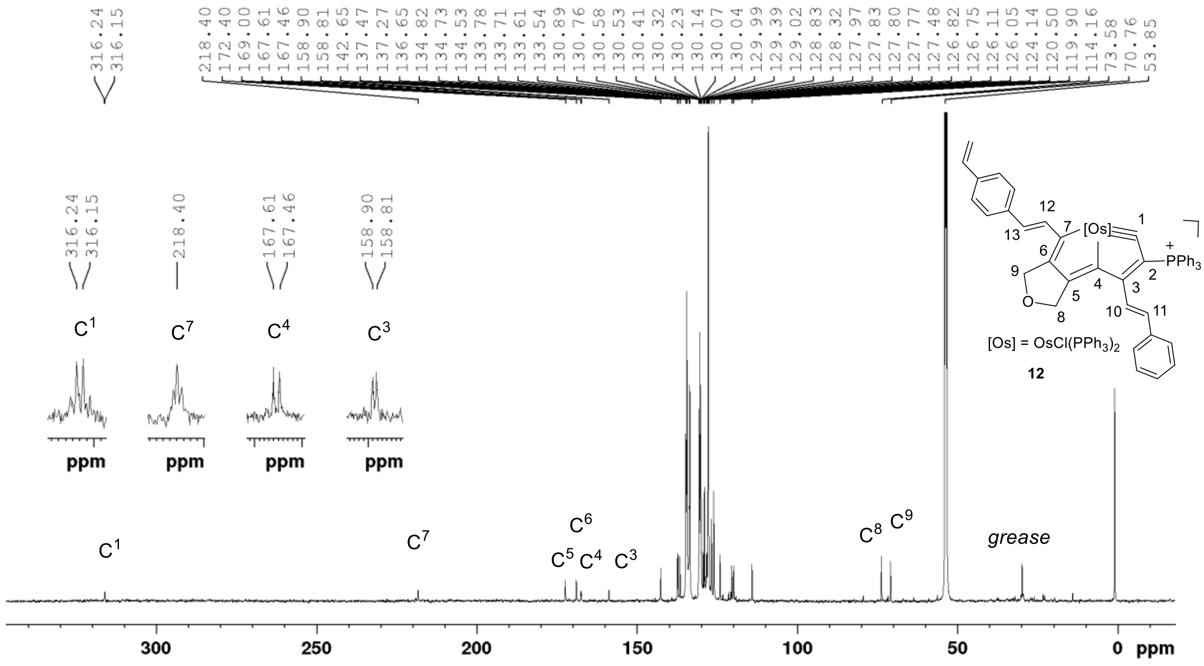

Supplementary Figure 106. The $^{13}C\{^1H\}$ NMR (150.9 MHz, CD_2Cl_2) spectrum for complex **12**.

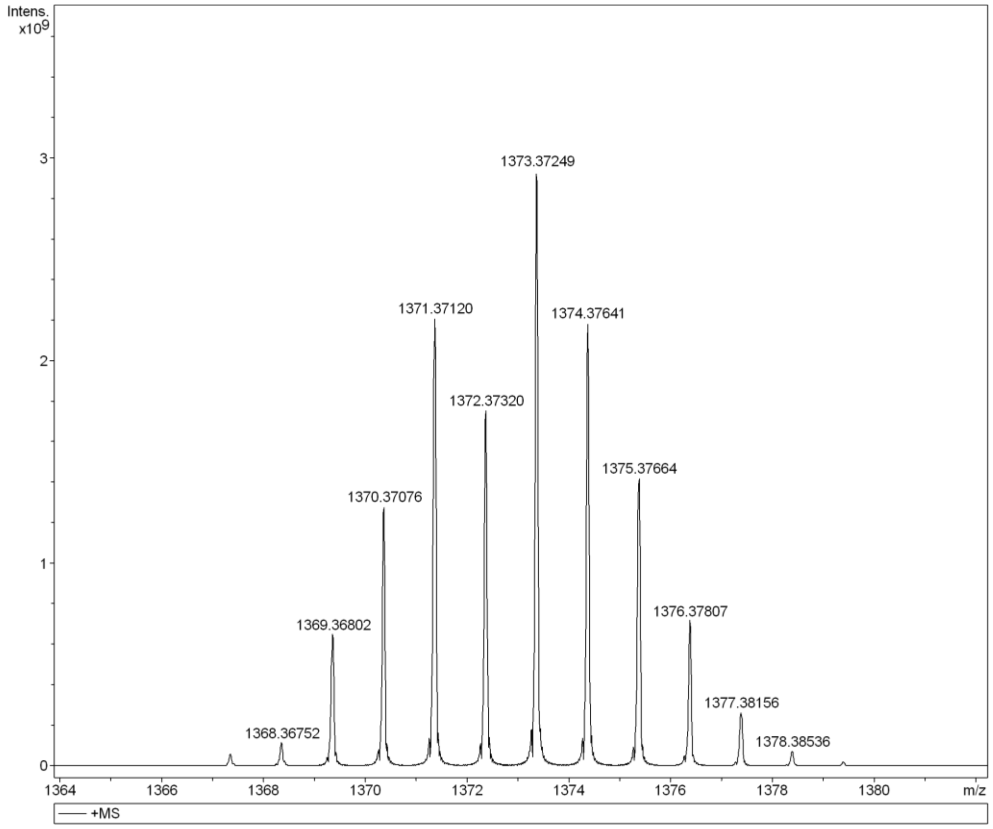

Supplementary Figure 107. Positive-ion ESI-MS spectrum of [**12**]$^+$ measured in methanol.

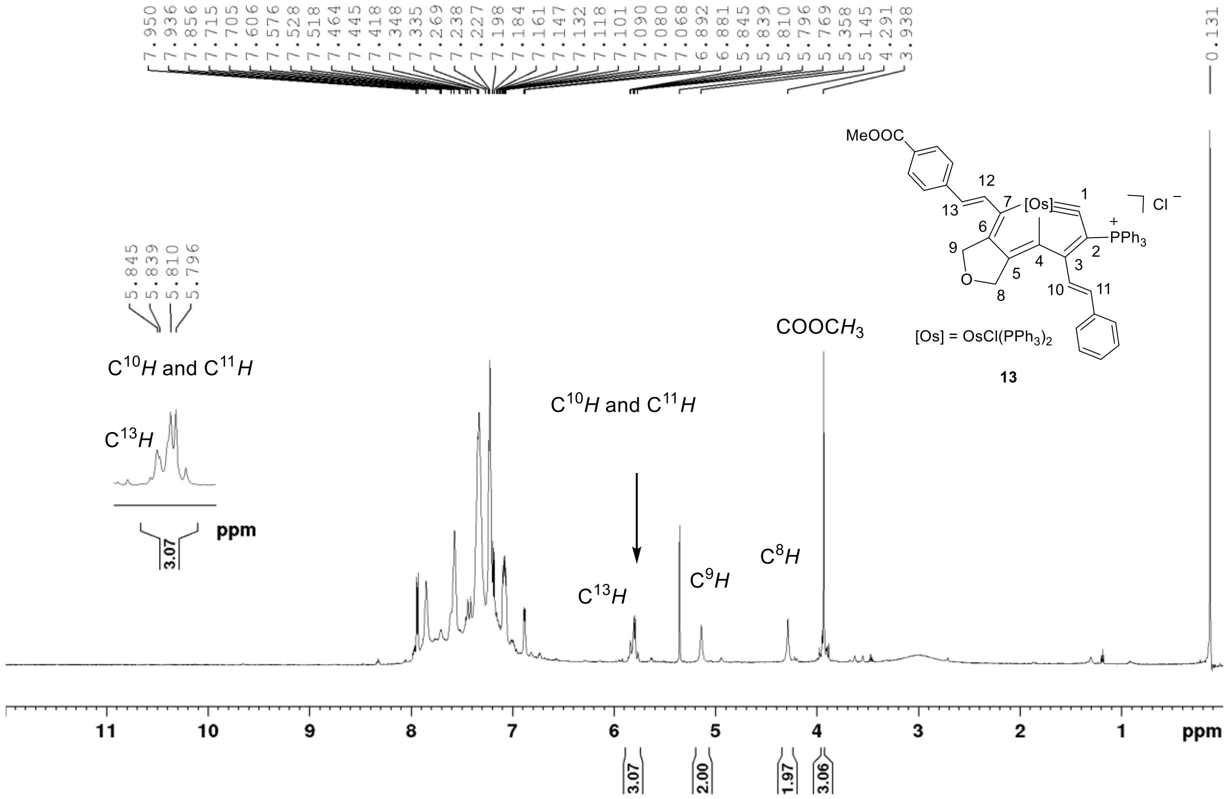

Supplementary Figure 108. The ^{1}H NMR (600.1 MHz, CD_2Cl_2) spectrum for complex **13**.

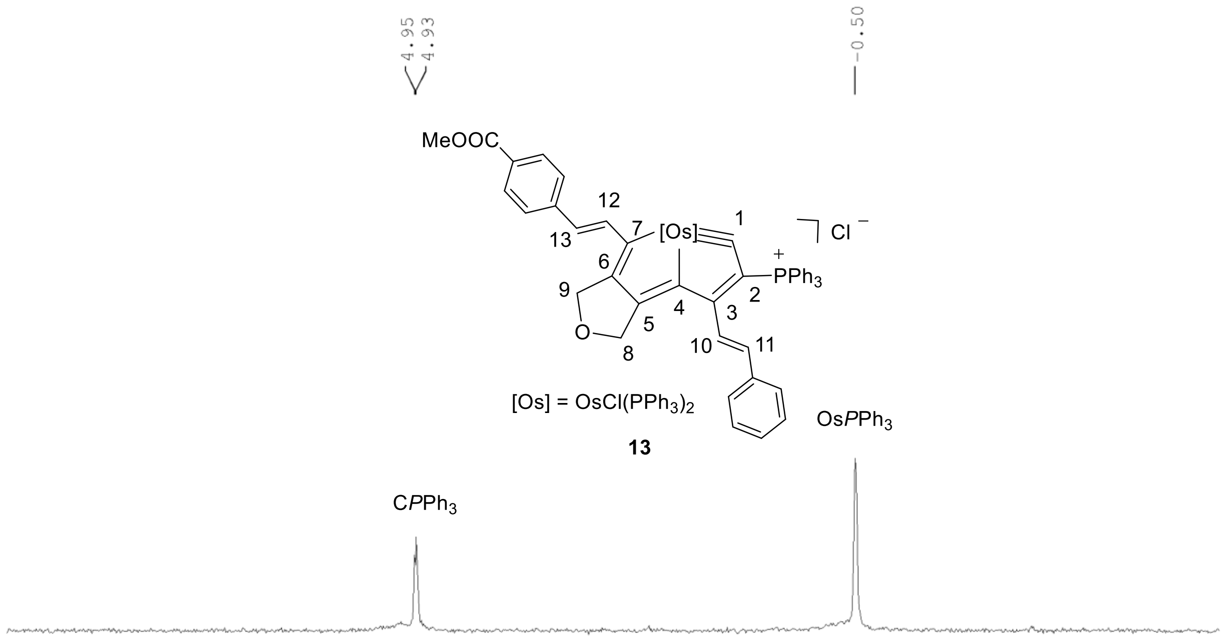

Supplementary Figure 109. The $^{31}P\{^{1}H\}$ NMR (242.9 MHz, CD_2Cl_2) spectrum for complex **13**.

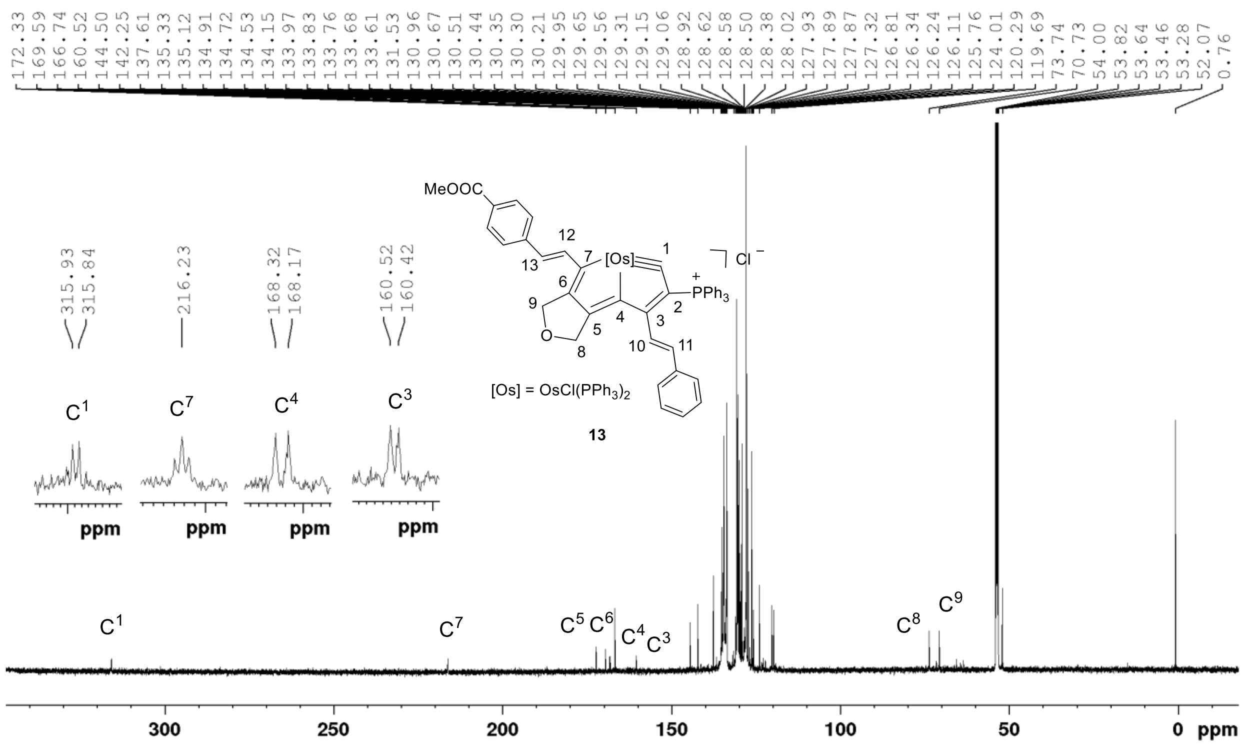

Supplementary Figure 110. The $^{13}C\{^1H\}$ NMR (150.9 MHz, CD_2Cl_2) spectrum for complex **13**.

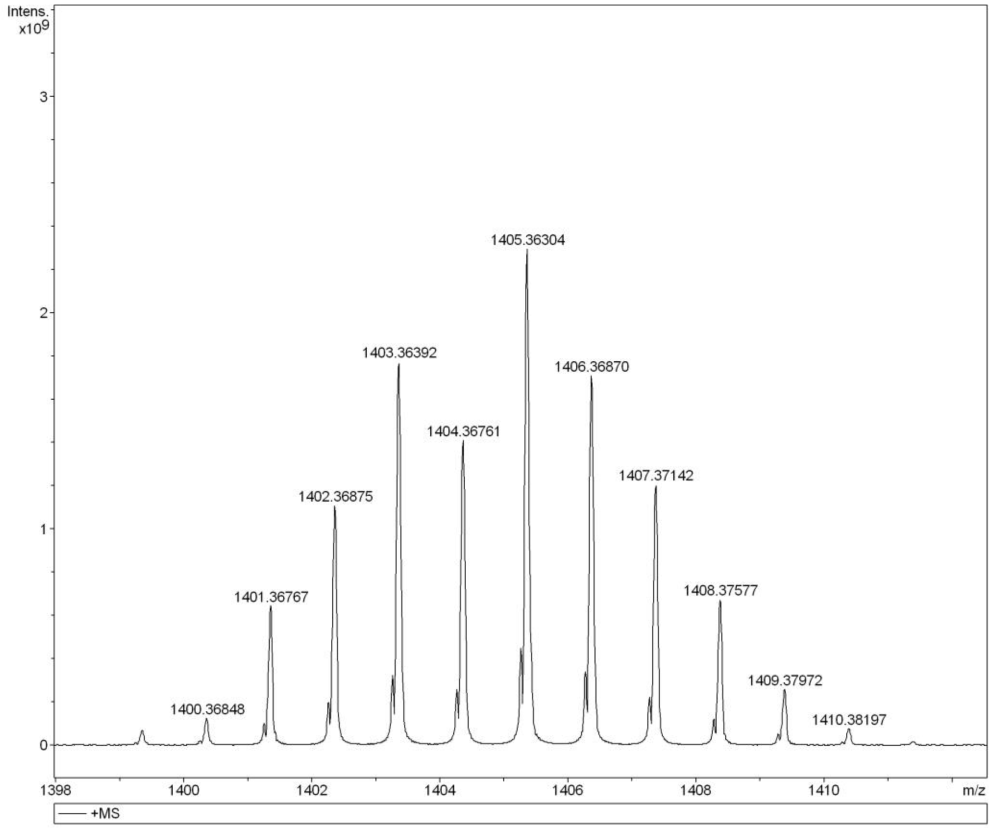

Supplementary Figure 111. Positive-ion ESI-MS spectrum of [**13**]$^+$ measured in methanol.

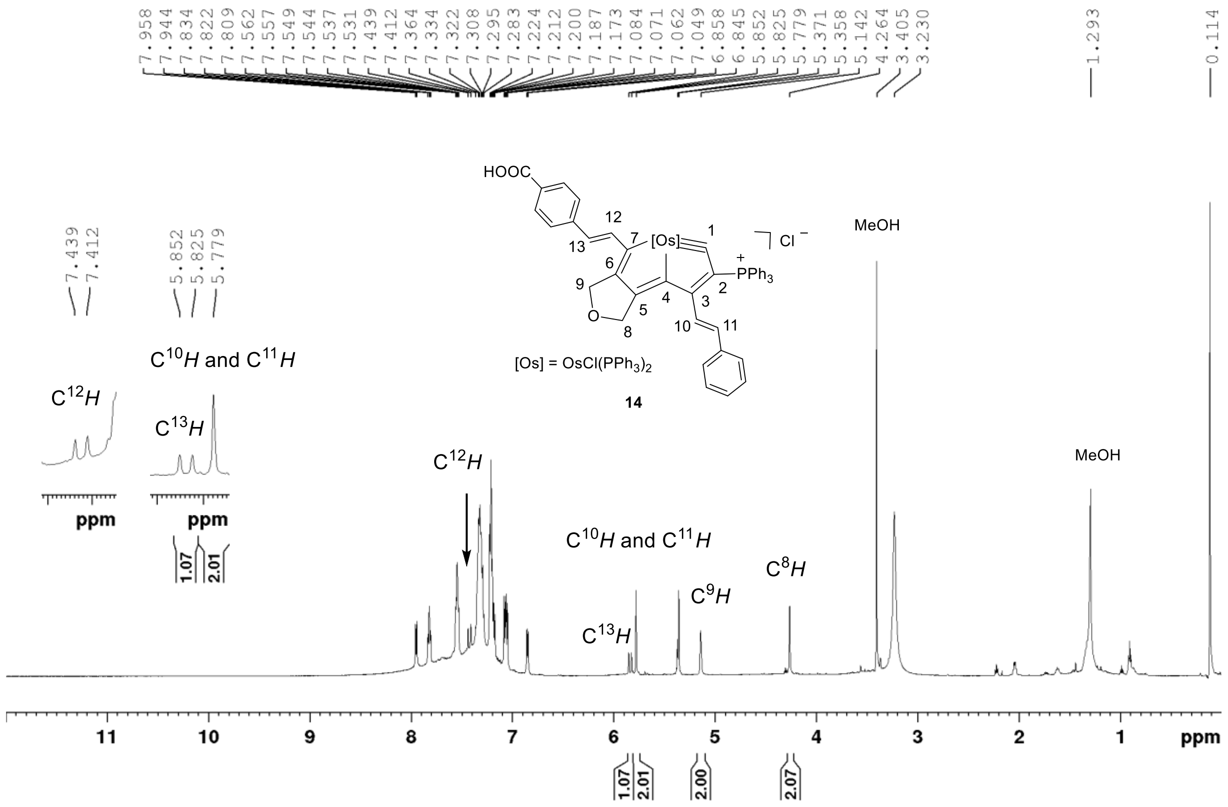

Supplementary Figure 112. The ^{1}H NMR (600.1 MHz, CD_2Cl_2) spectrum for complex **14**.

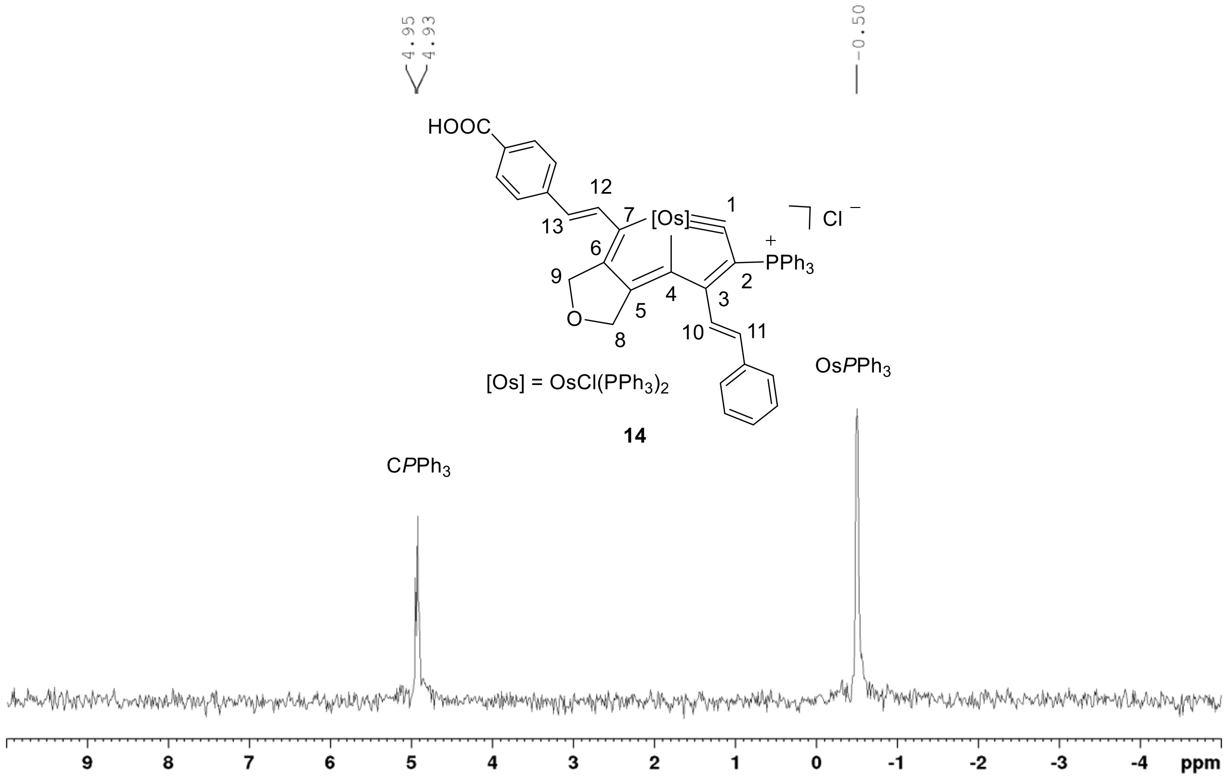

Supplementary Figure 113. The $^{31}P\{^{1}H\}$ NMR (242.9 MHz, CD_2Cl_2) spectrum for complex **14**.

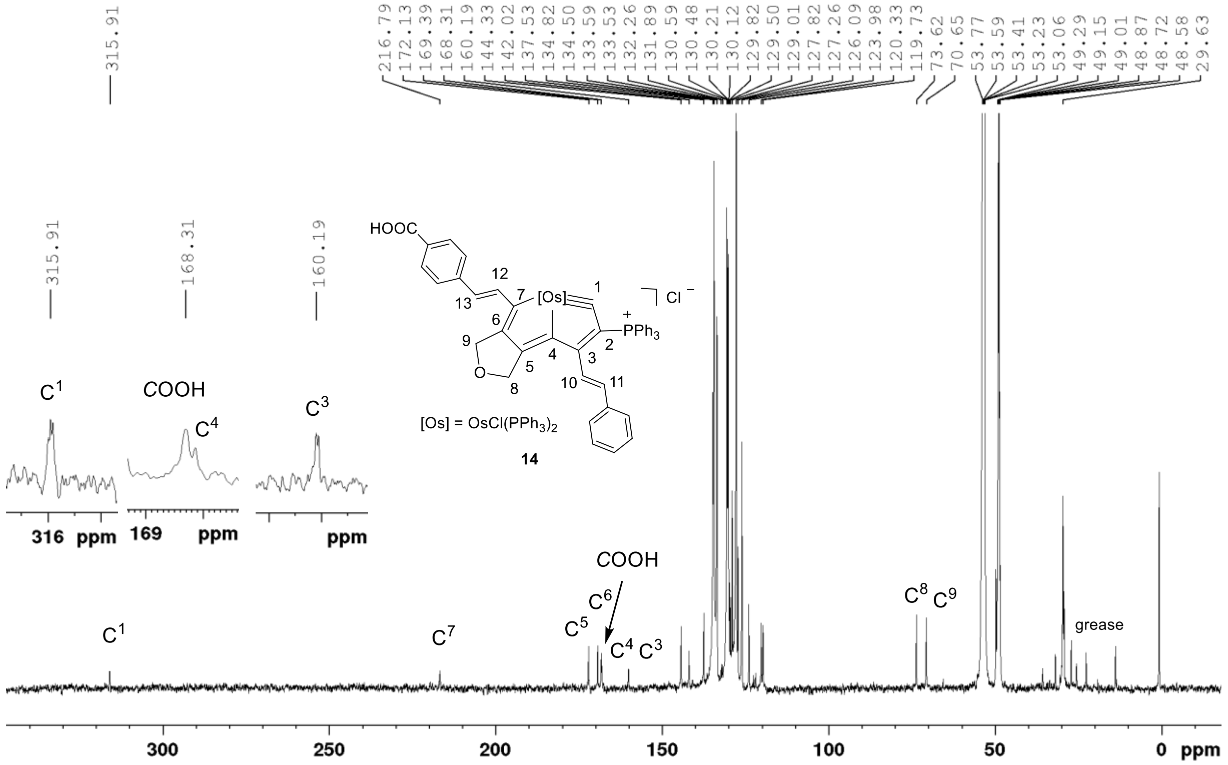

Supplementary Figure 114. The $^{13}C\{^{1}H\}$ NMR (150.9 MHz, CD_2Cl_2) spectrum for complex **14**.

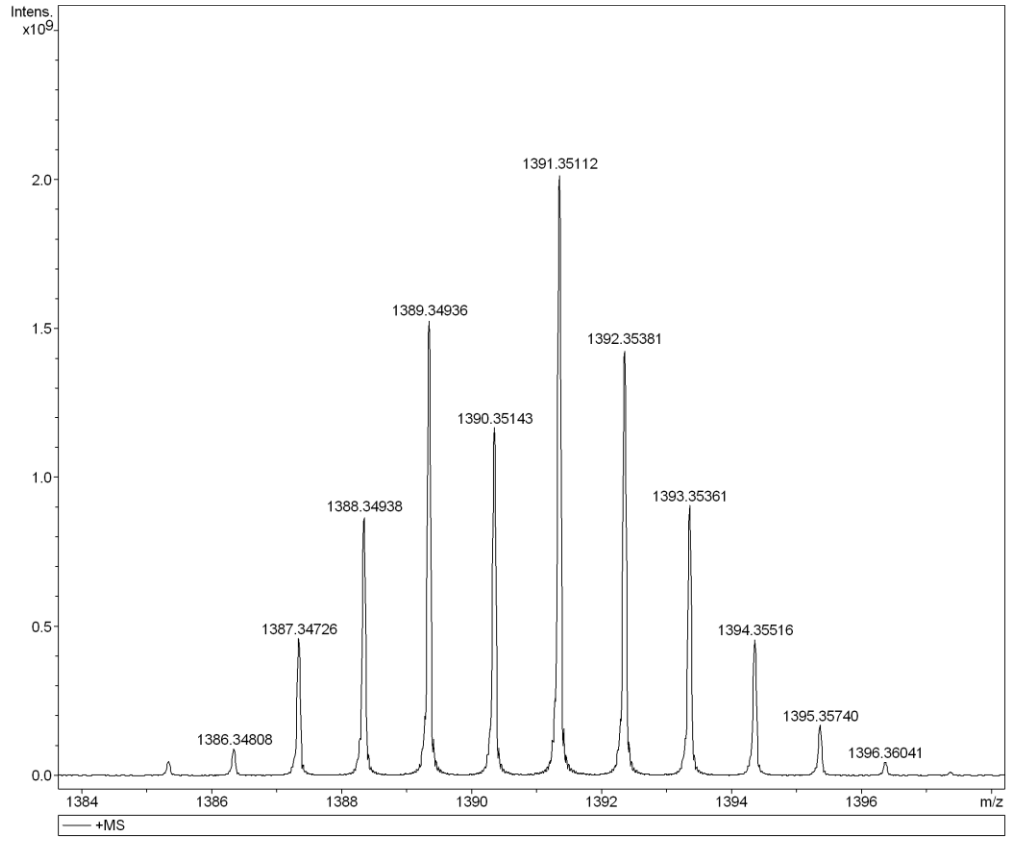

Supplementary Figure 115. Positive-ion ESI-MS spectrum of [**14**]$^{+}$ measured in methanol.

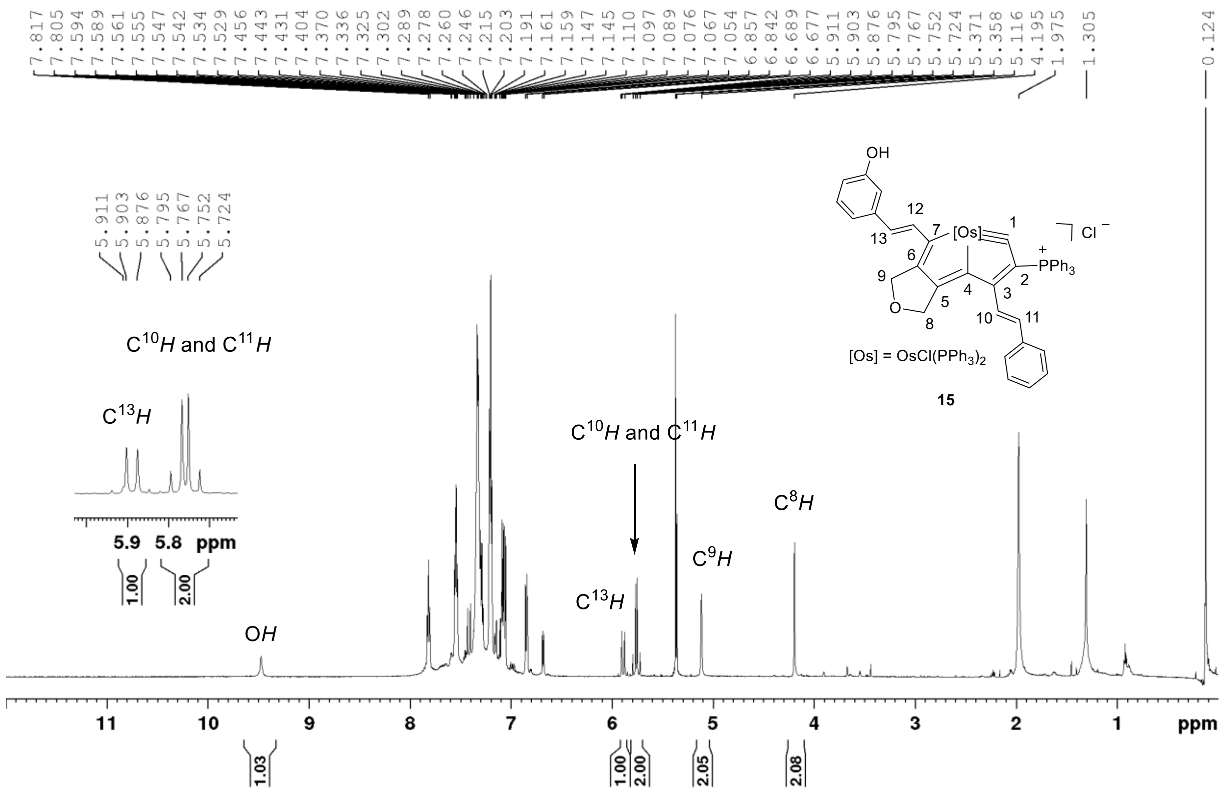

Supplementary Figure 116. The ^{1}H NMR (600.1 MHz, CD_2Cl_2) spectrum for complex **15**.

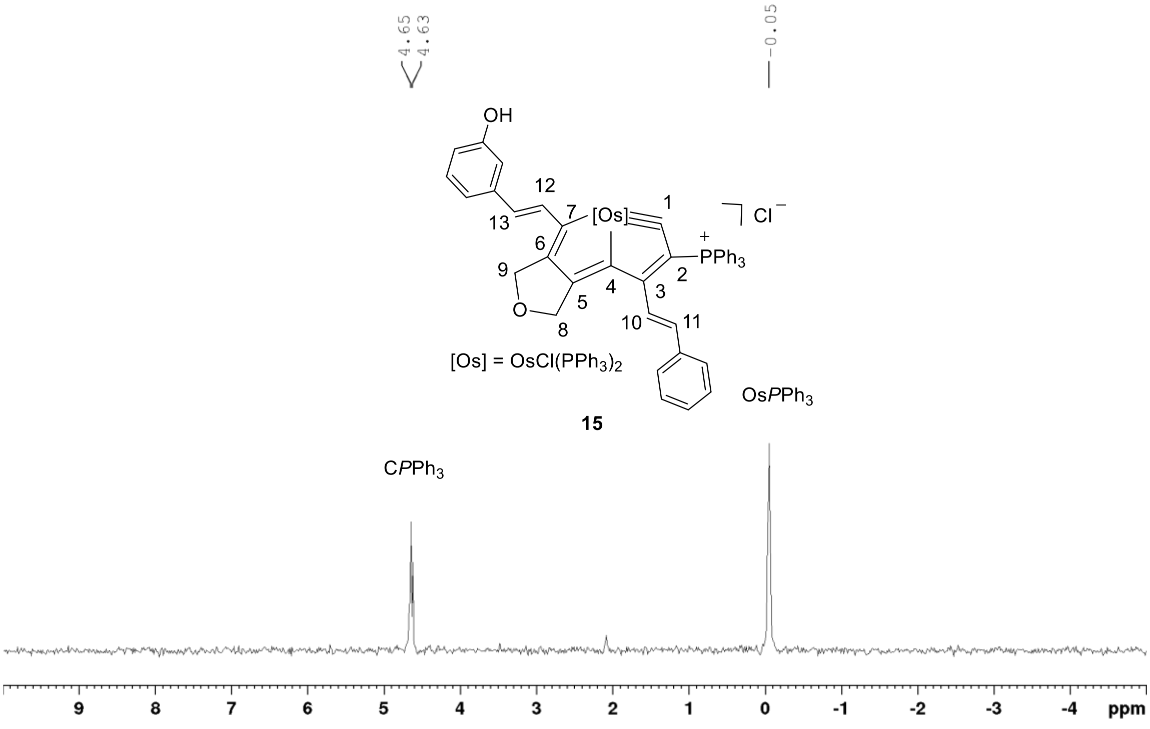

Supplementary Figure 117. The ^{31}P{^{1}H} NMR (242.9 MHz, CD_2Cl_2) spectrum for complex **15**.

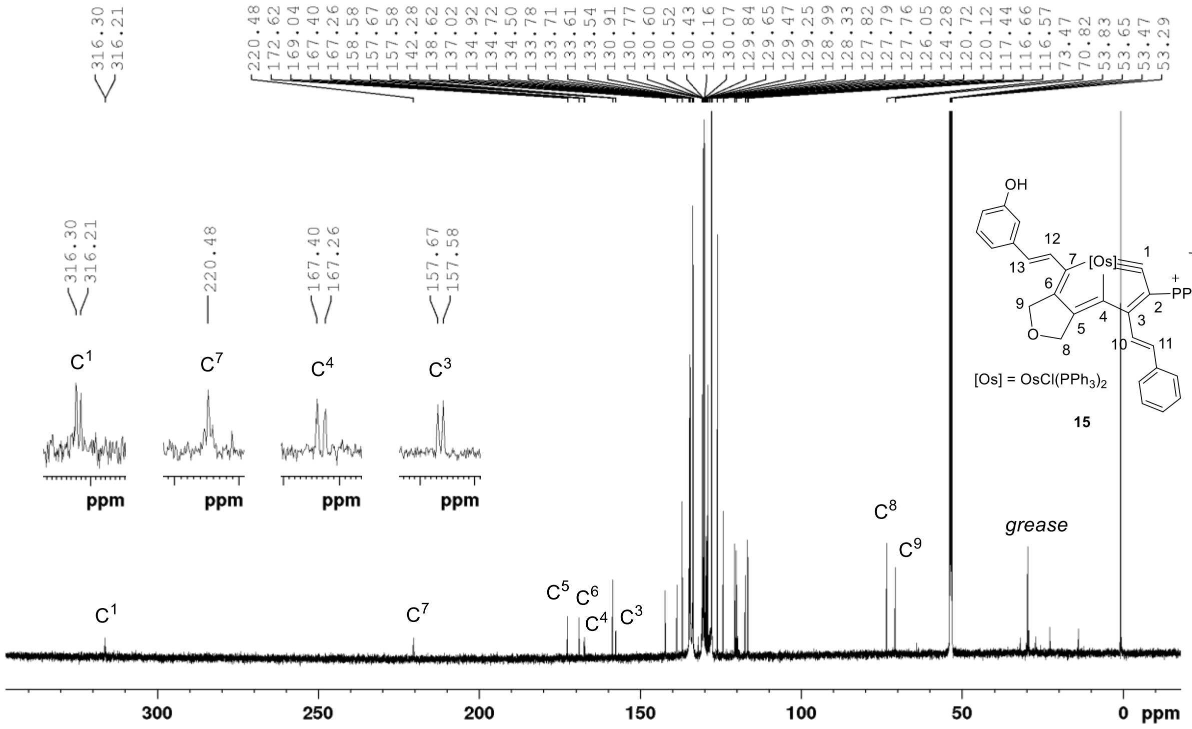

Supplementary Figure 118. The $^{13}C\{^1H\}$ NMR (150.9 MHz, CD_2Cl_2) spectrum for complex **15**.

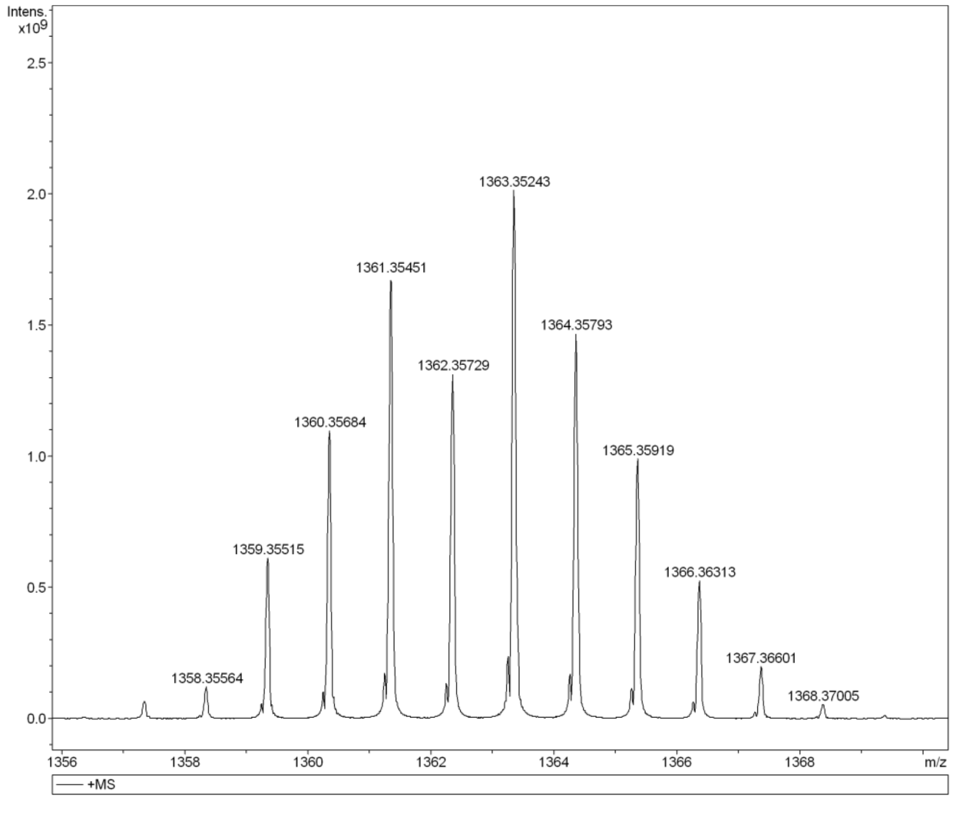

Supplementary Figure 119. Positive-ion ESI-MS spectrum of $[\mathbf{15}]^+$ measured in methanol.

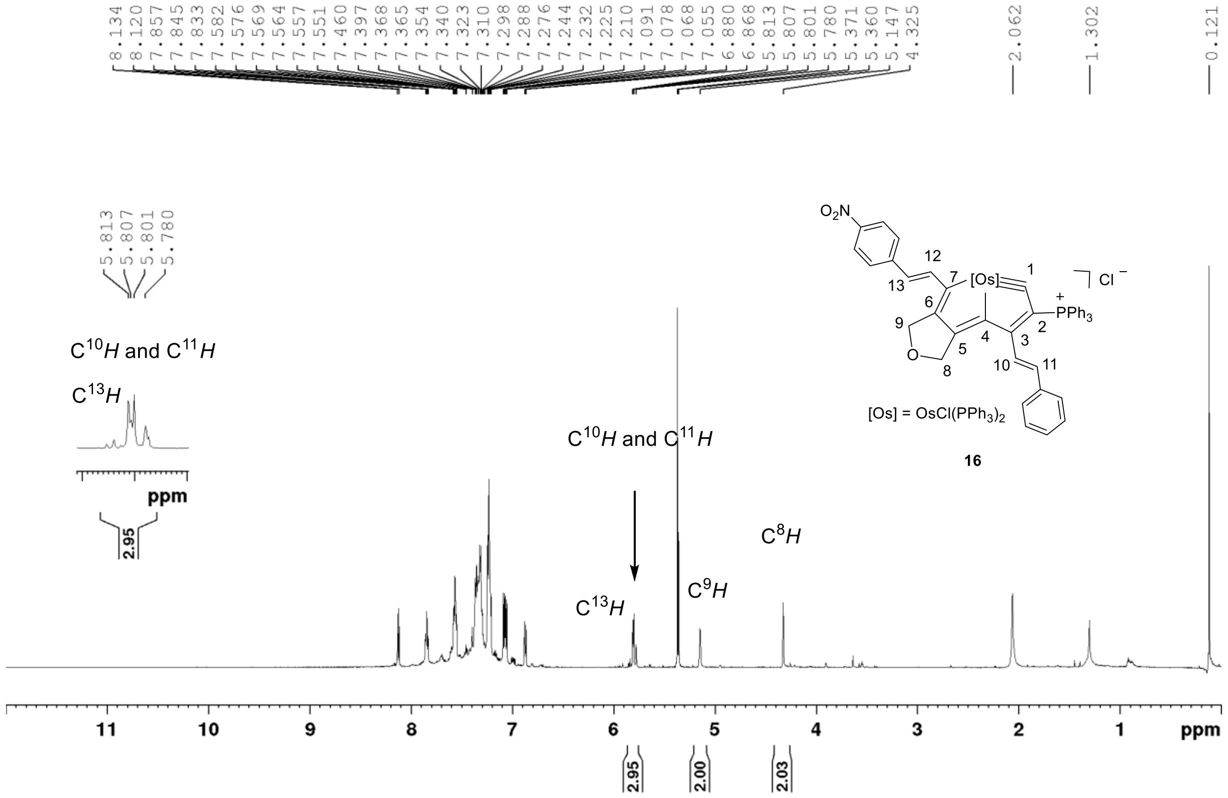

Supplementary Figure 120. The ^{1}H NMR (600.1 MHz, CD_2Cl_2) spectrum for complex **16**.

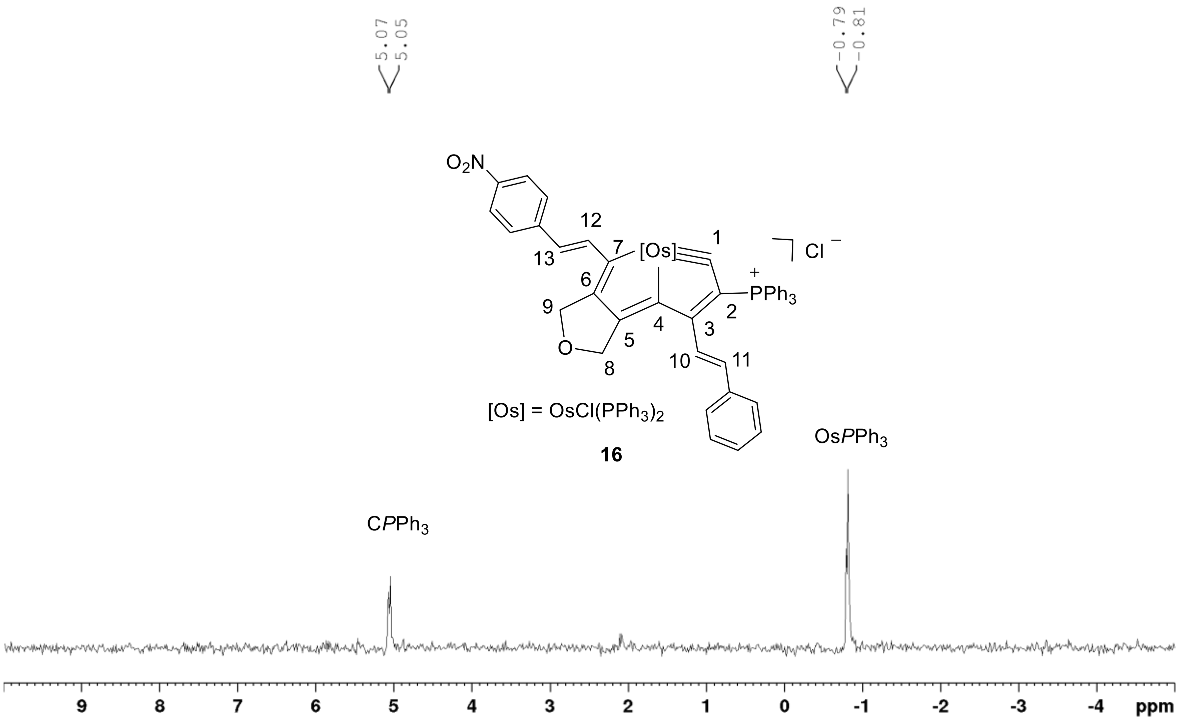

Supplementary Figure 121. The $^{31}P\{^1H\}$ NMR (242.9 MHz, CD_2Cl_2) spectrum for complex **16**.

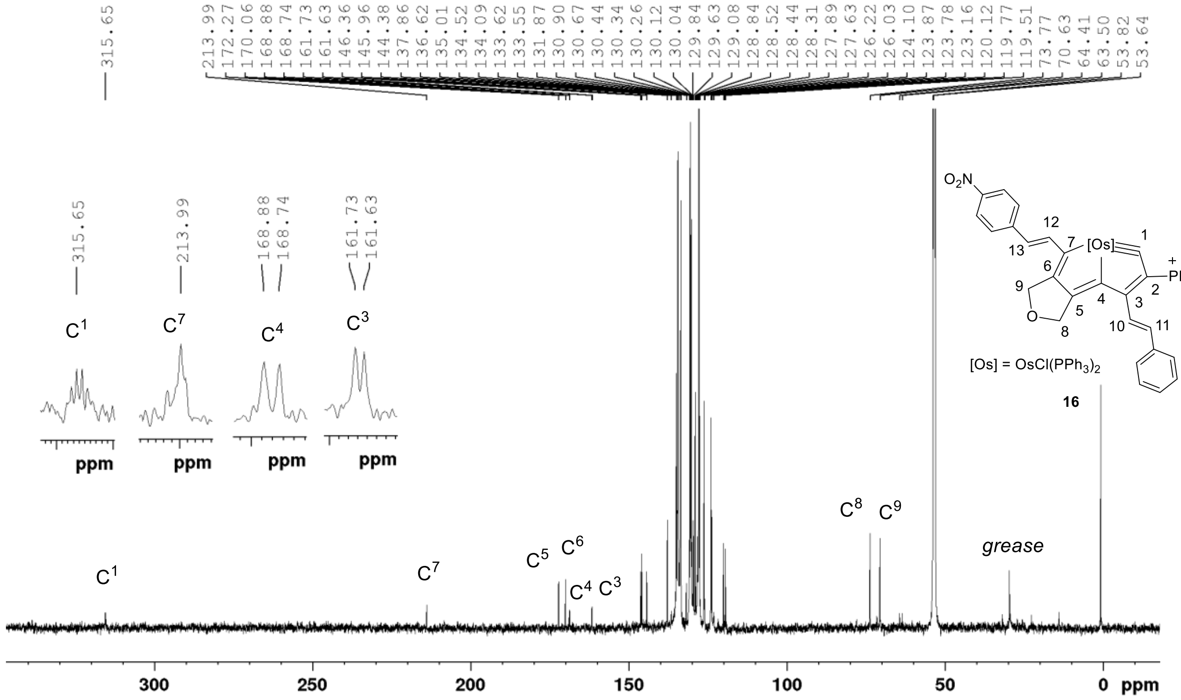

Supplementary Figure 122. The $^{13}C\{^{1}H\}$ NMR (150.9 MHz, CD_2Cl_2) spectrum for complex **16**.

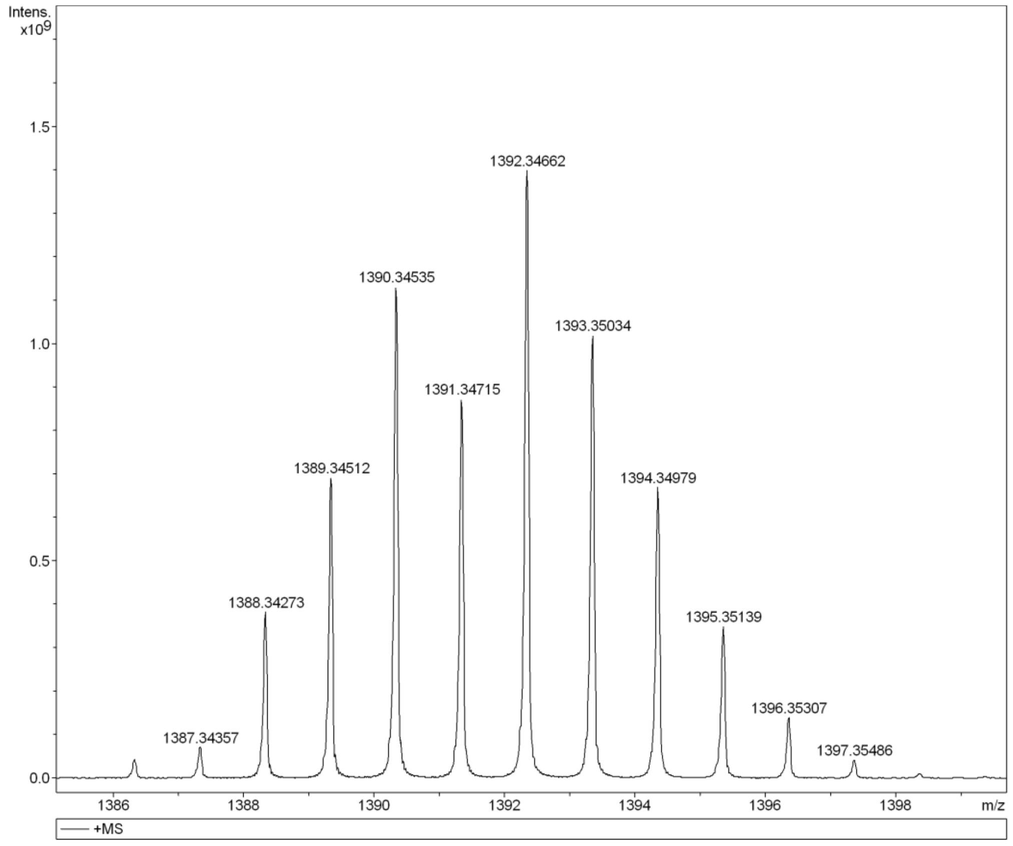

Supplementary Figure 123. Positive-ion ESI-MS spectrum of [**16**]$^+$ measured in methanol.

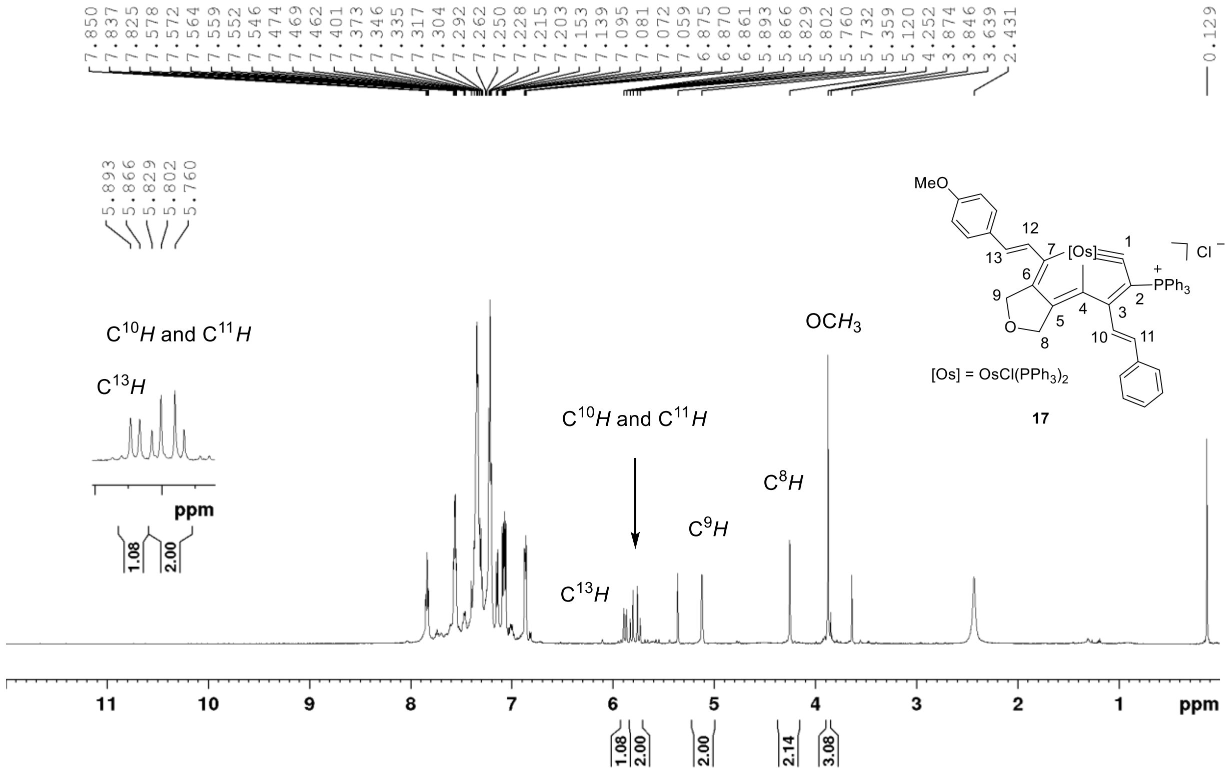

Supplementary Figure 124. The ^{1}H NMR (600.1 MHz, CD_2Cl_2) spectrum for complex **17**.

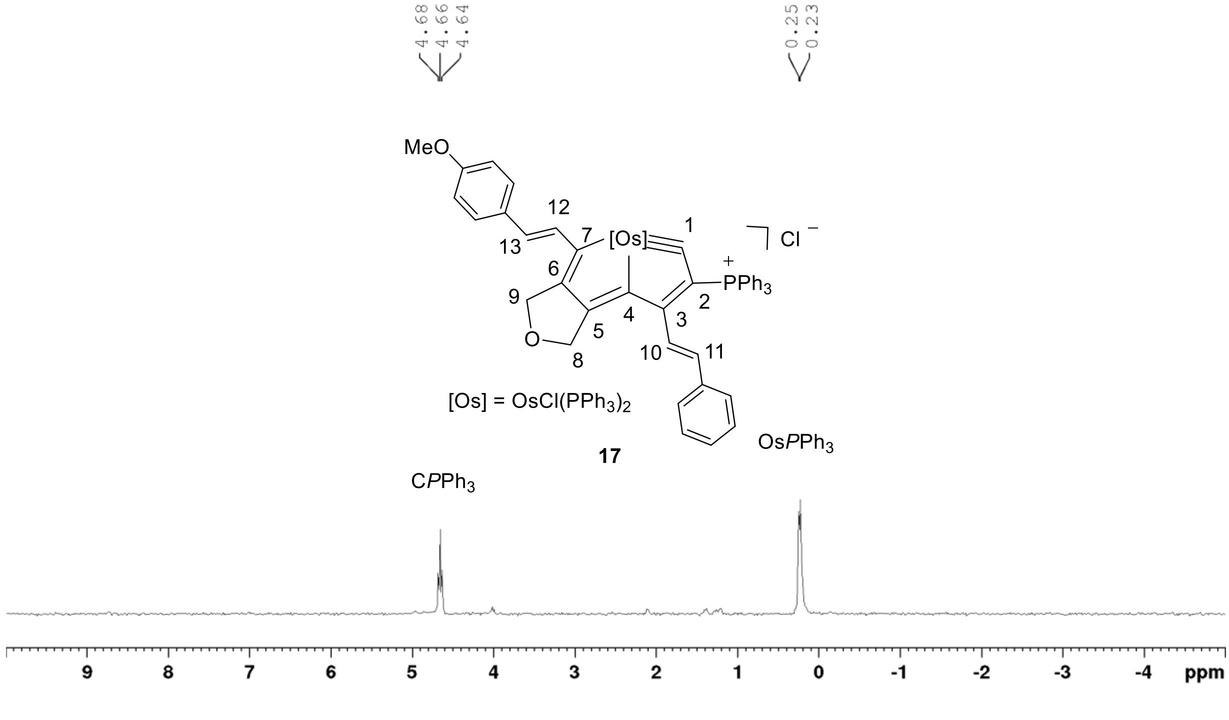

Supplementary Figure 125. The ^{31}P{^{1}H} NMR (242.9 MHz, CD_2Cl_2) spectrum for complex **17**.

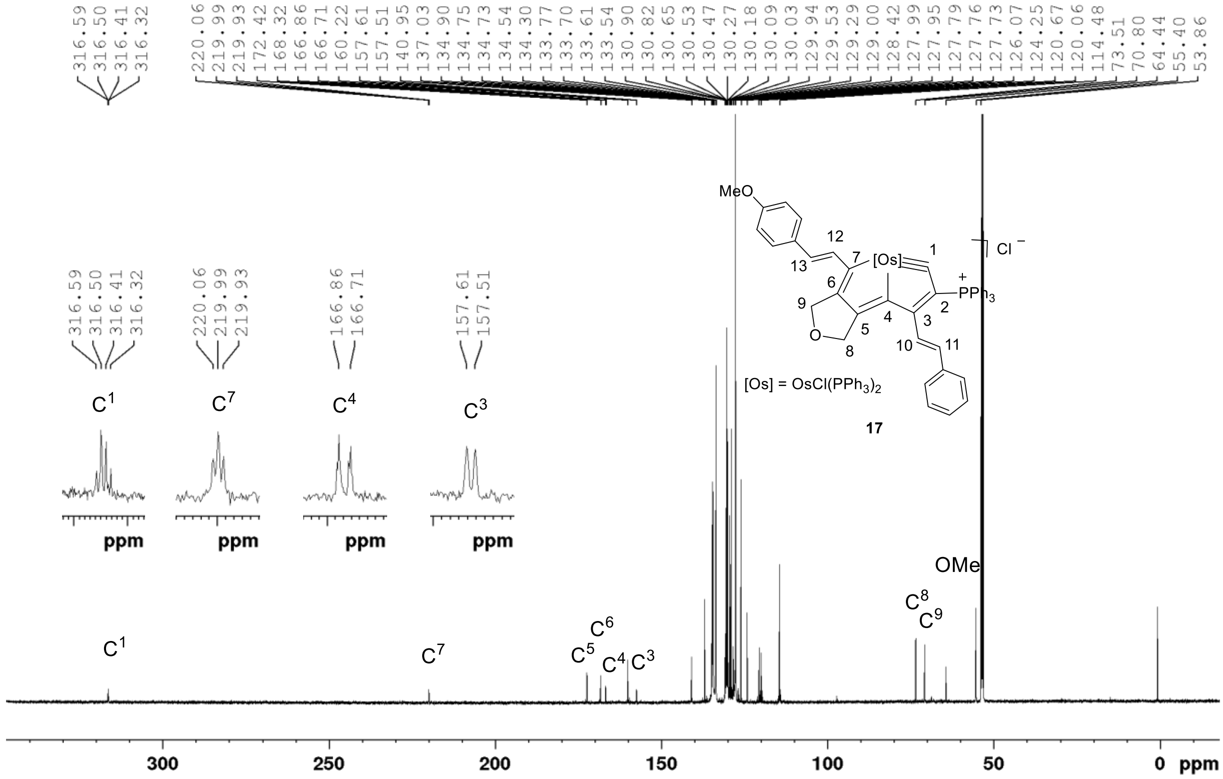

Supplementary Figure 126. The $^{13}C\{^1H\}$ NMR (150.9 MHz, CD_2Cl_2) spectrum for complex **17**.

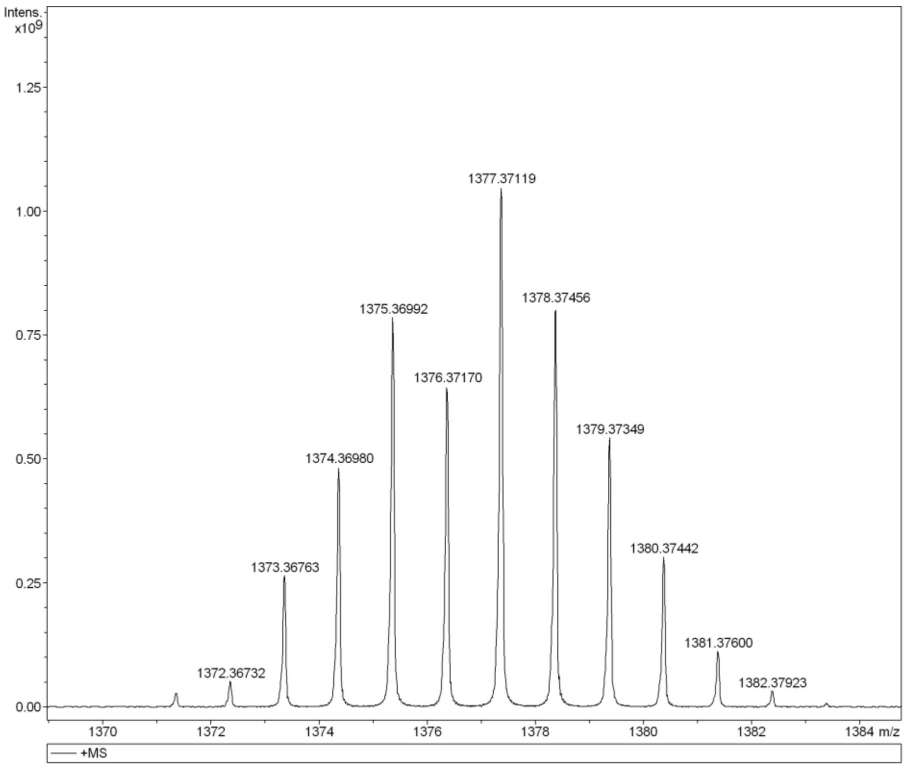

Supplementary Figure 127. Positive-ion ESI-MS spectrum of [**17**]$^+$ measured in methanol.

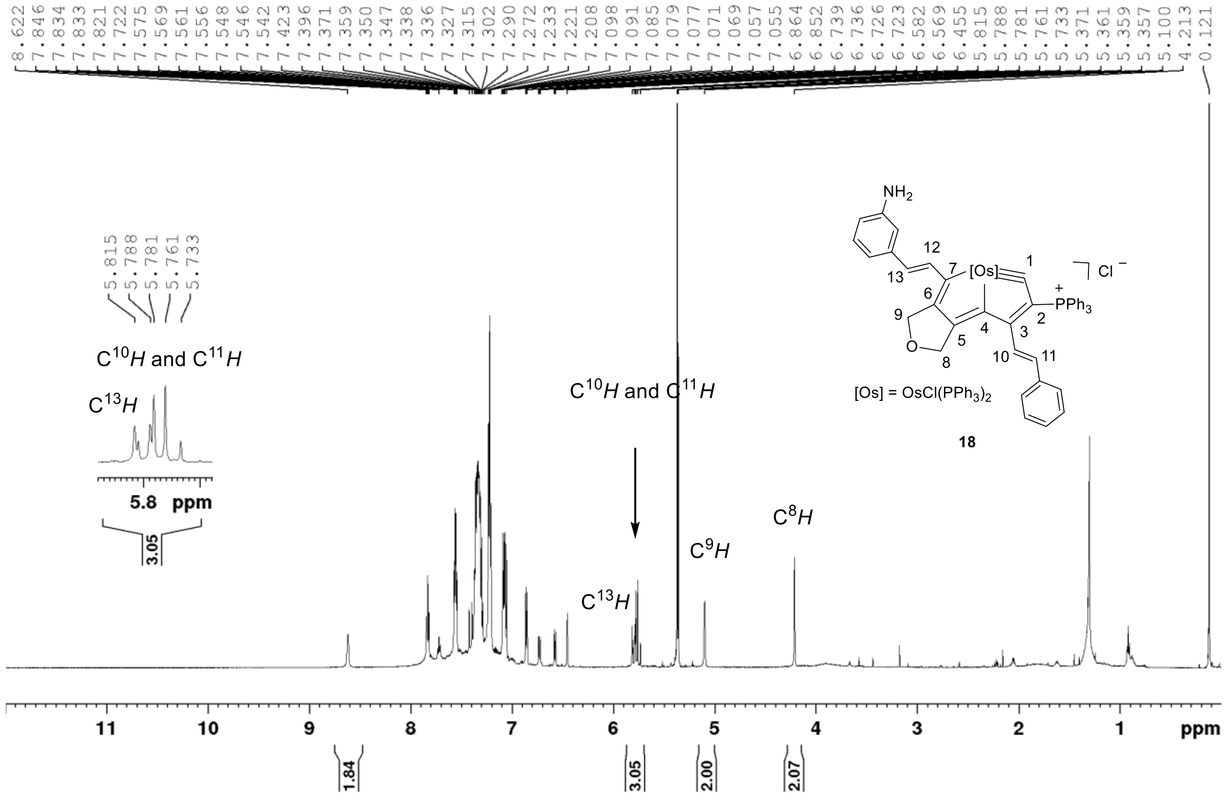

Supplementary Figure 128. The ^{1}H NMR (600.1 MHz, CD_2Cl_2) spectrum for complex **18**.

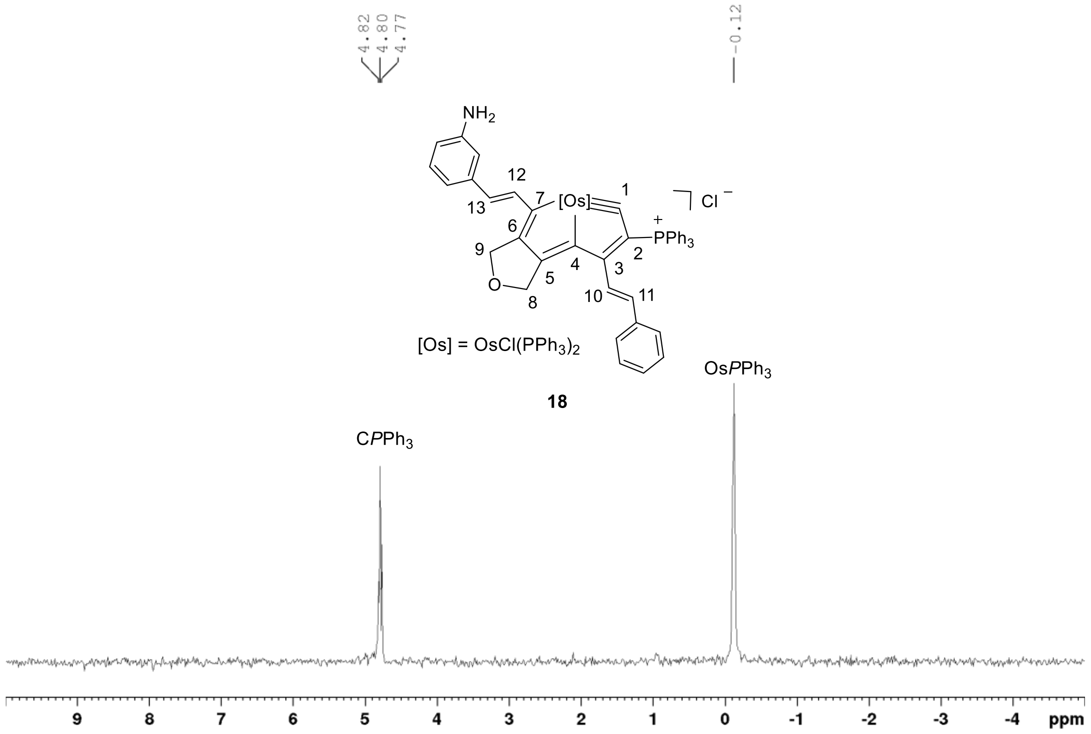

Supplementary Figure 129. The ^{31}P{^{1}H} NMR (242.9 MHz, CD_2Cl_2) spectrum for complex **18**.

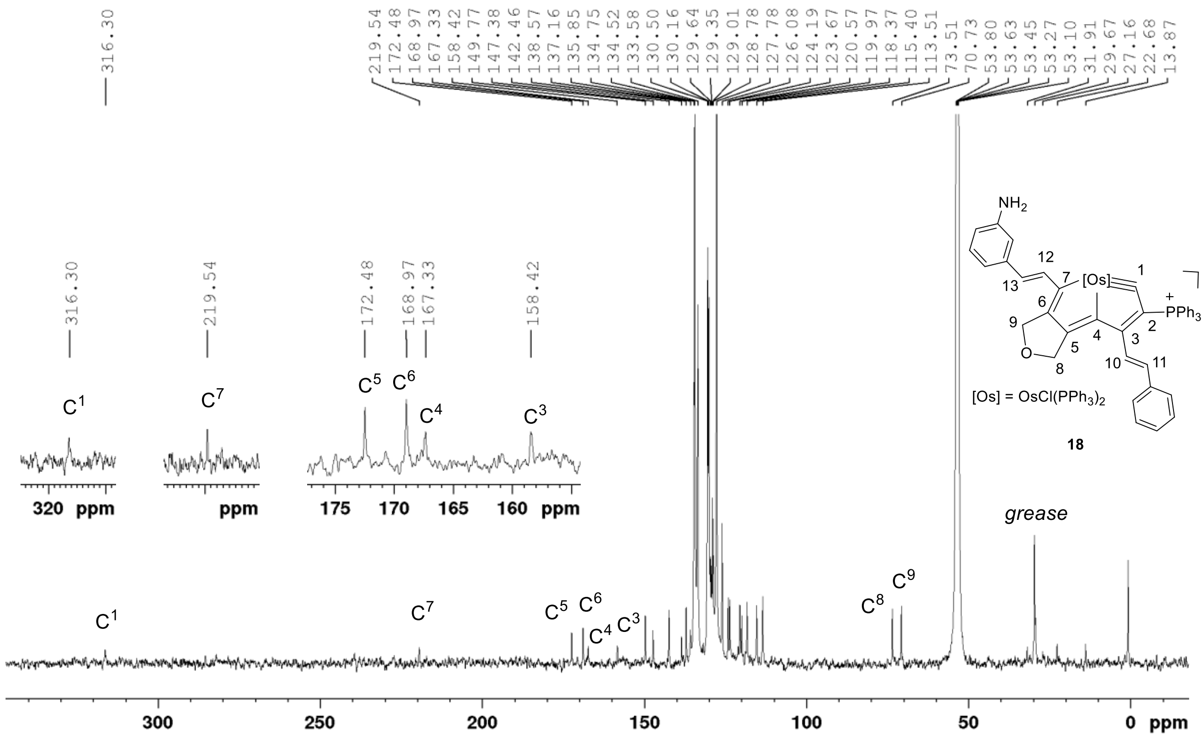

Supplementary Figure 130. The $^{13}C\{^{1}H\}$ NMR (150.9 MHz, CD_2Cl_2) spectrum for complex **18**.

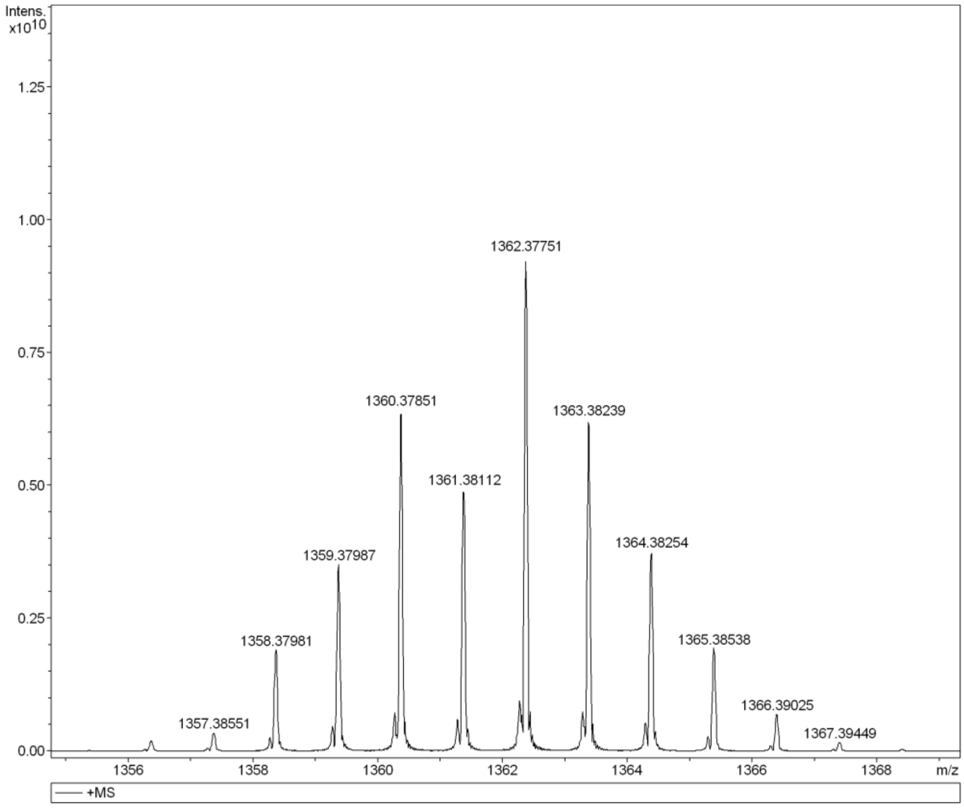

Supplementary Figure 131. Positive-ion ESI-MS spectrum of [**18**]$^{+}$ measured in methanol.

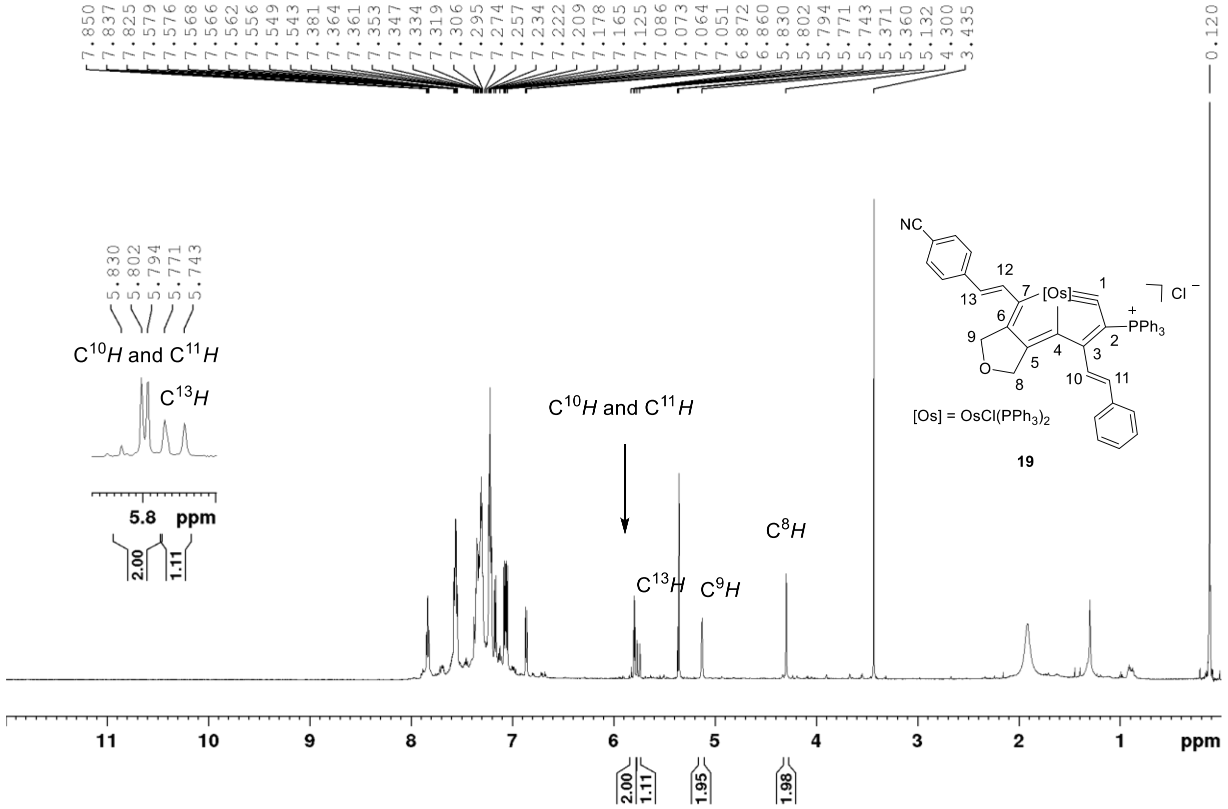

Supplementary Figure 132. The ^{1}H NMR (600.1 MHz, CD_2Cl_2) spectrum for complex **19**.

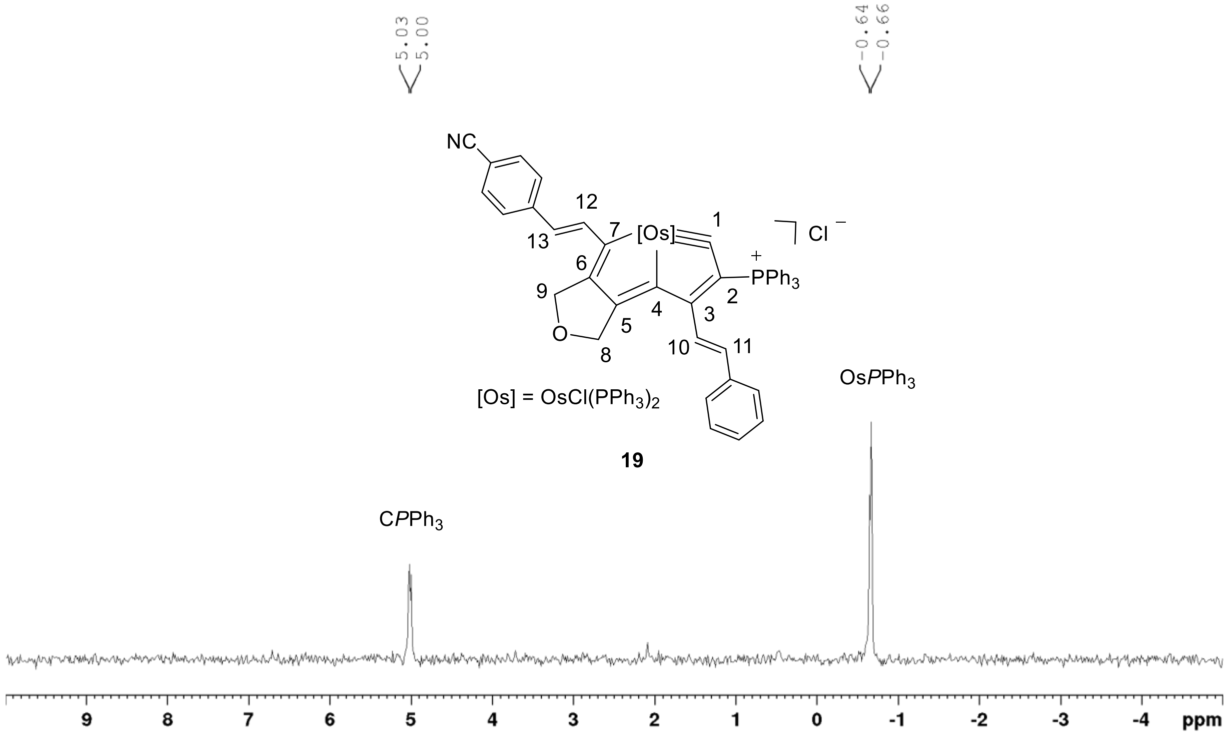

Supplementary Figure 133. The $^{31}P\{^{1}H\}$ NMR (242.9 MHz, CD_2Cl_2) spectrum for complex **19**.

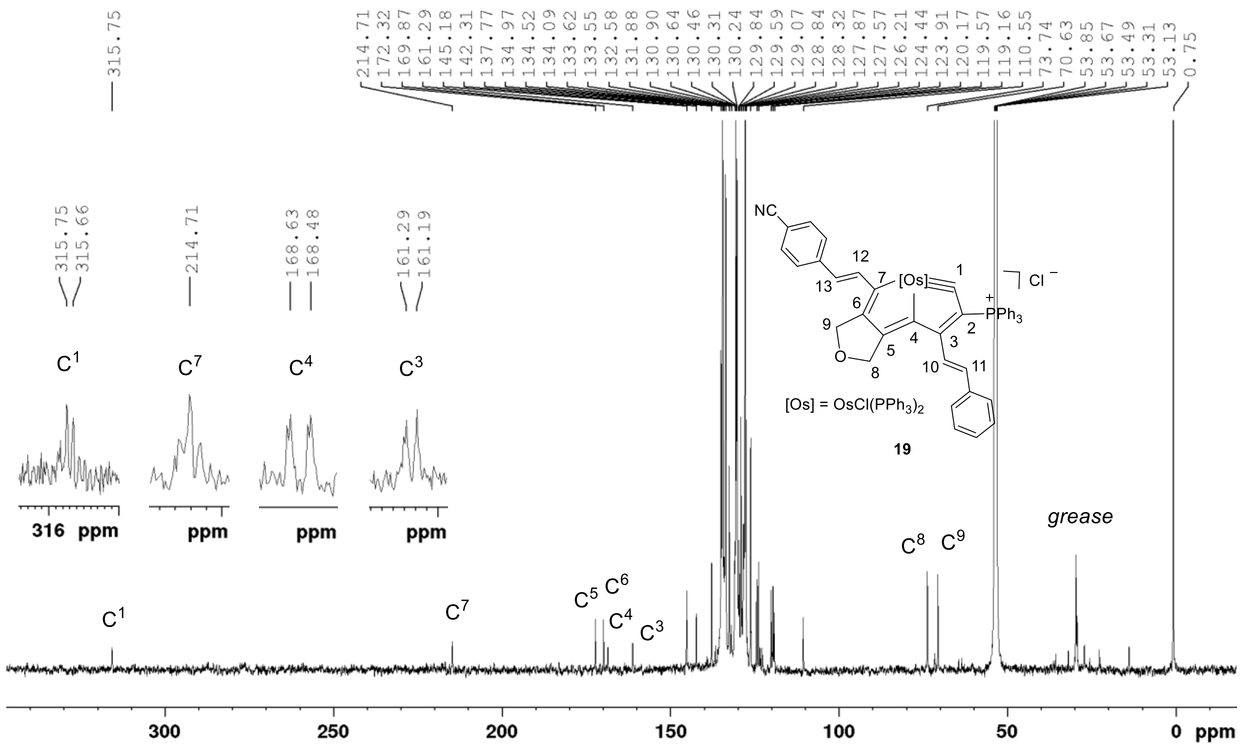

Supplementary Figure 134. The $^{13}C\{^1H\}$ NMR (150.9 MHz, CD_2Cl_2) spectrum for complex **19**.

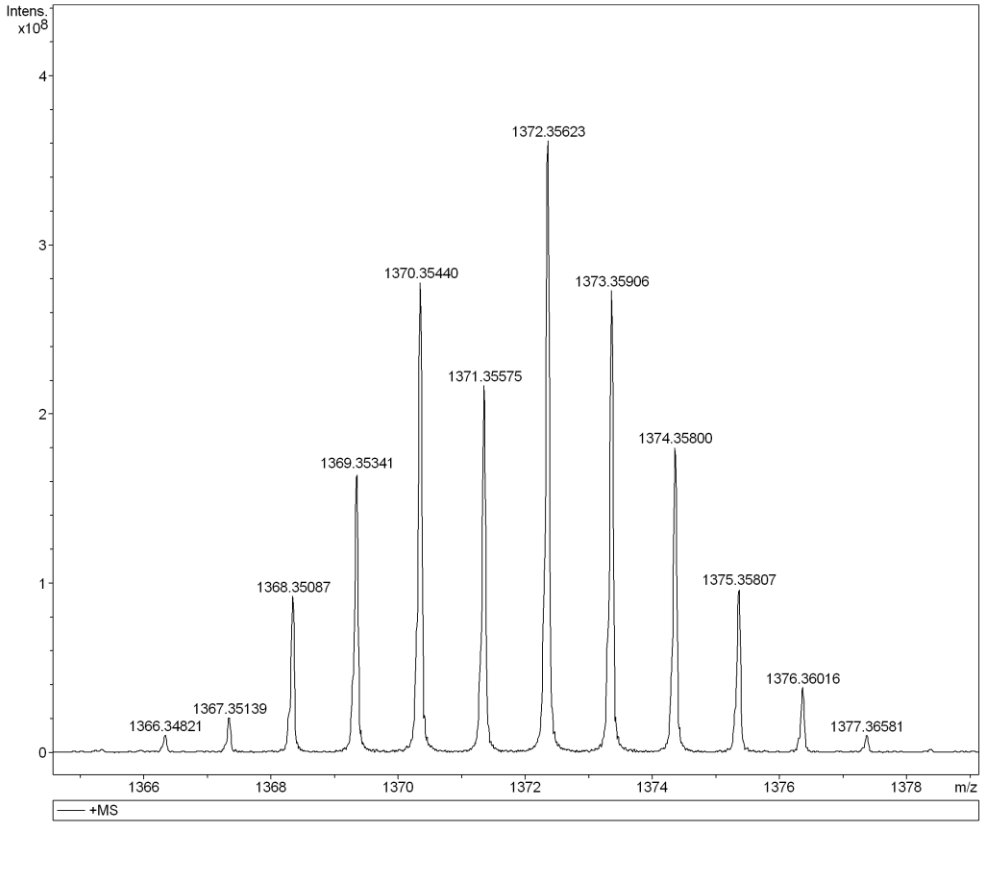

Supplementary Figure 135. Positive-ion ESI-MS spectrum of $[\mathbf{19}]^+$ measured in methanol.

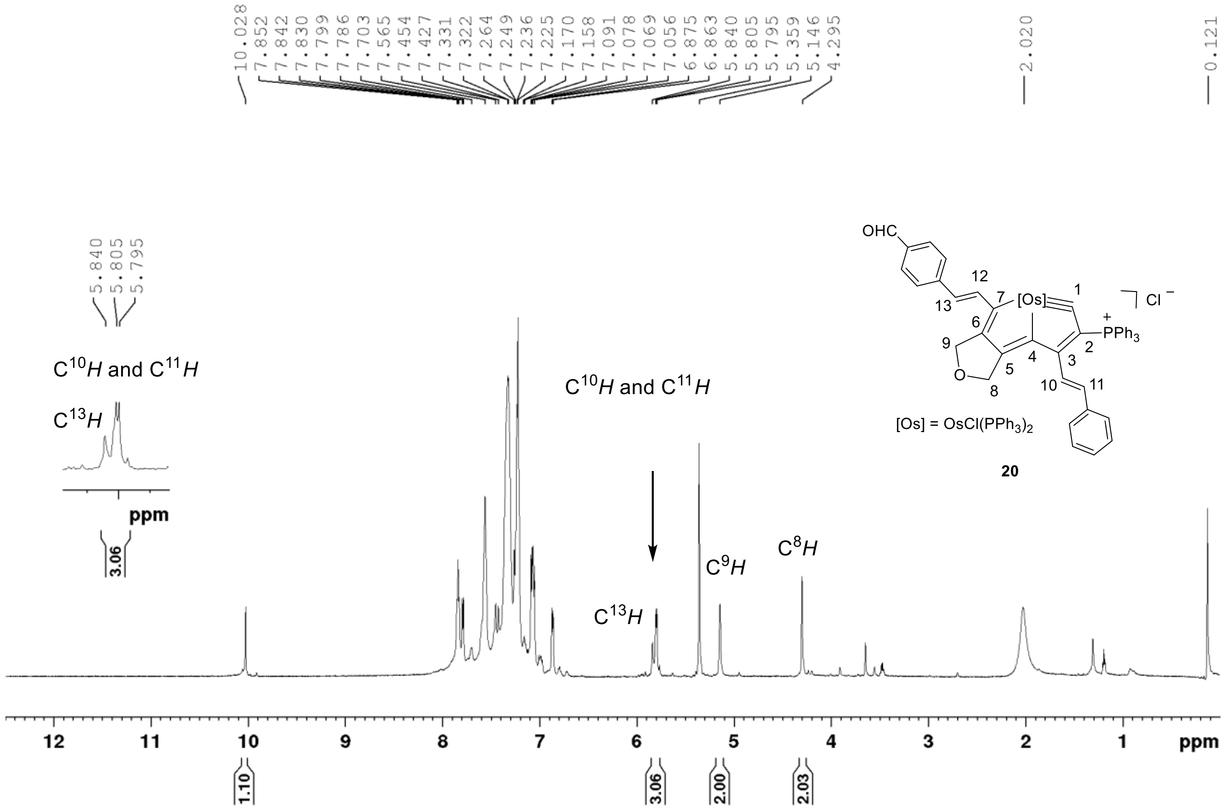

Supplementary Figure 136. The ^{1}H NMR (600.1 MHz, CD_2Cl_2) spectrum for complex **20**.

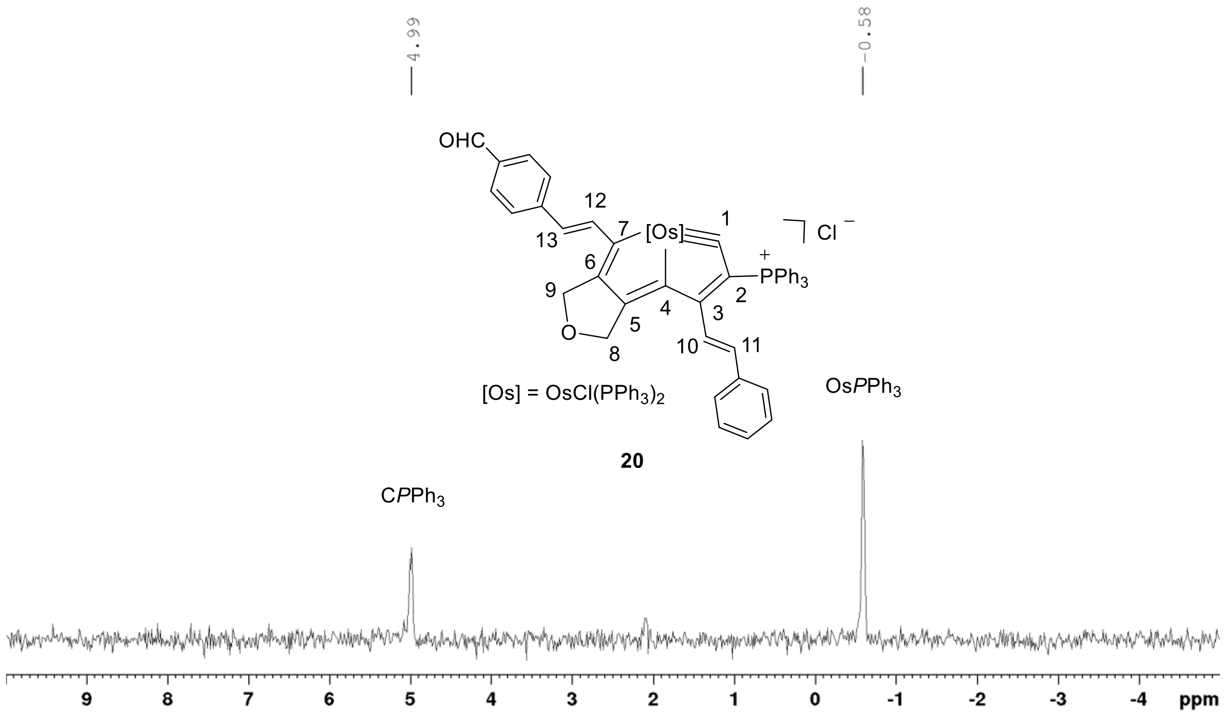

Supplementary Figure 137. The $^{31}P\{^{1}H\}$ NMR (242.9 MHz, CD_2Cl_2) spectrum for complex **20**.

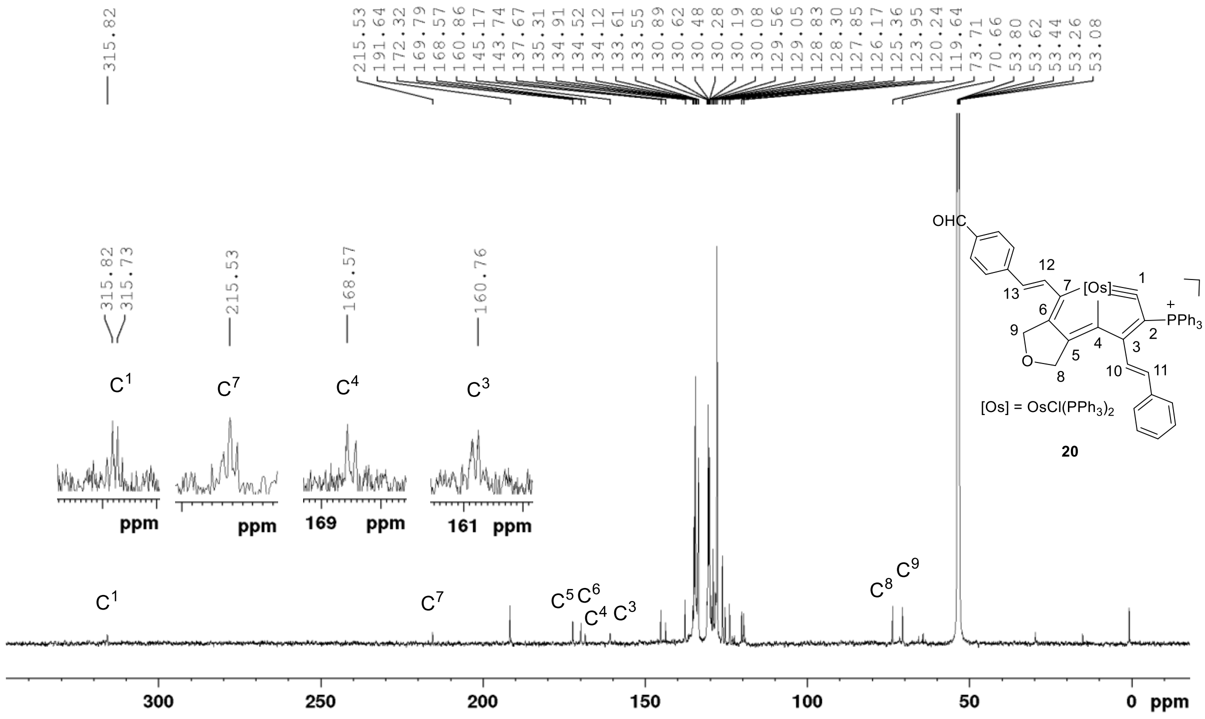

Supplementary Figure 138. The $^{13}C\{^{1}H\}$ NMR (150.9 MHz, CD_2Cl_2) spectrum for complex **20**.

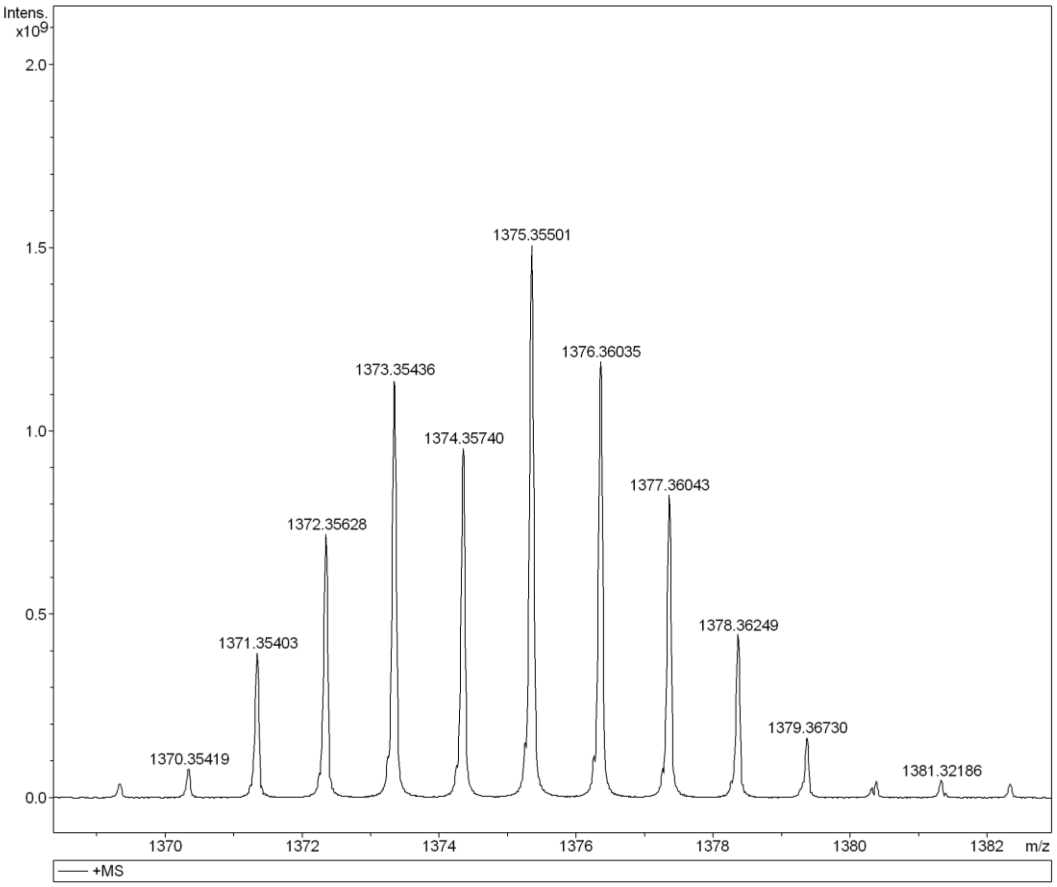

Supplementary Figure 139. Positive-ion ESI-MS spectrum of [**20**]$^+$ measured in methanol.

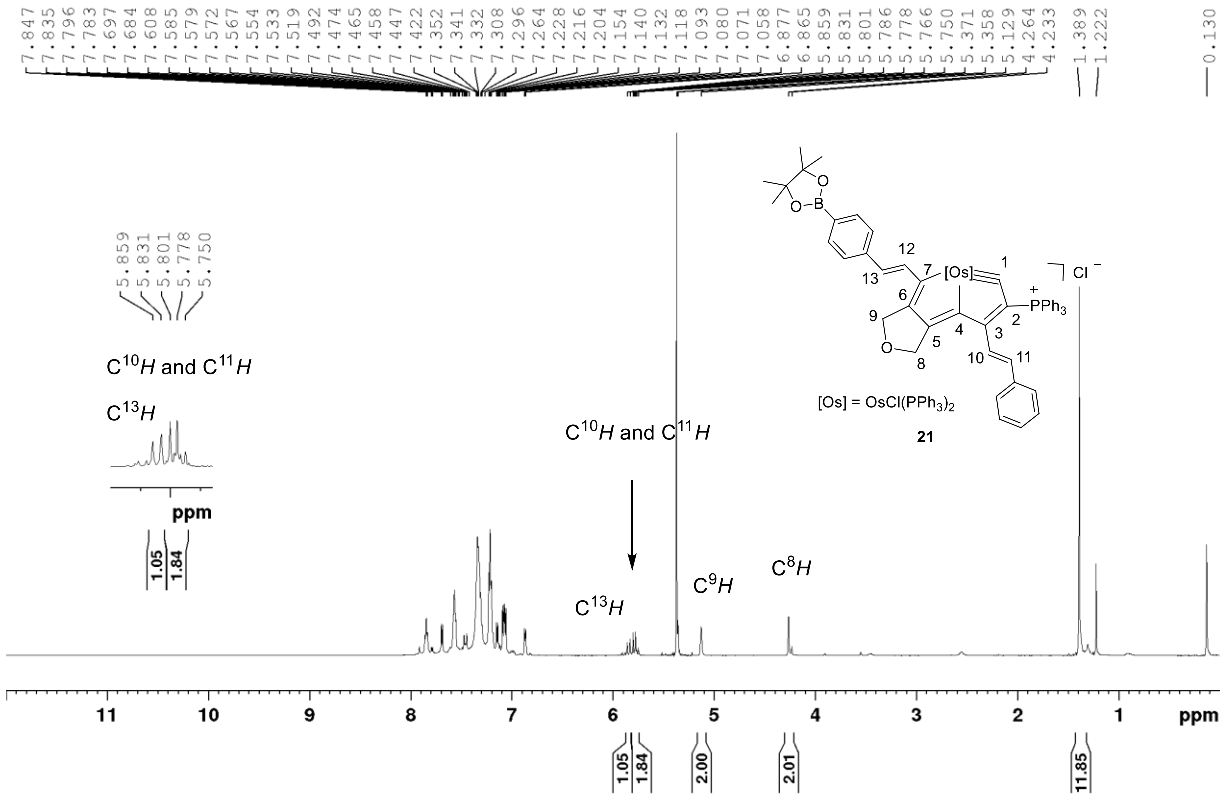

Supplementary Figure 140. The ^{1}H NMR (600.1 MHz, CD_2Cl_2) spectrum for complex **21**.

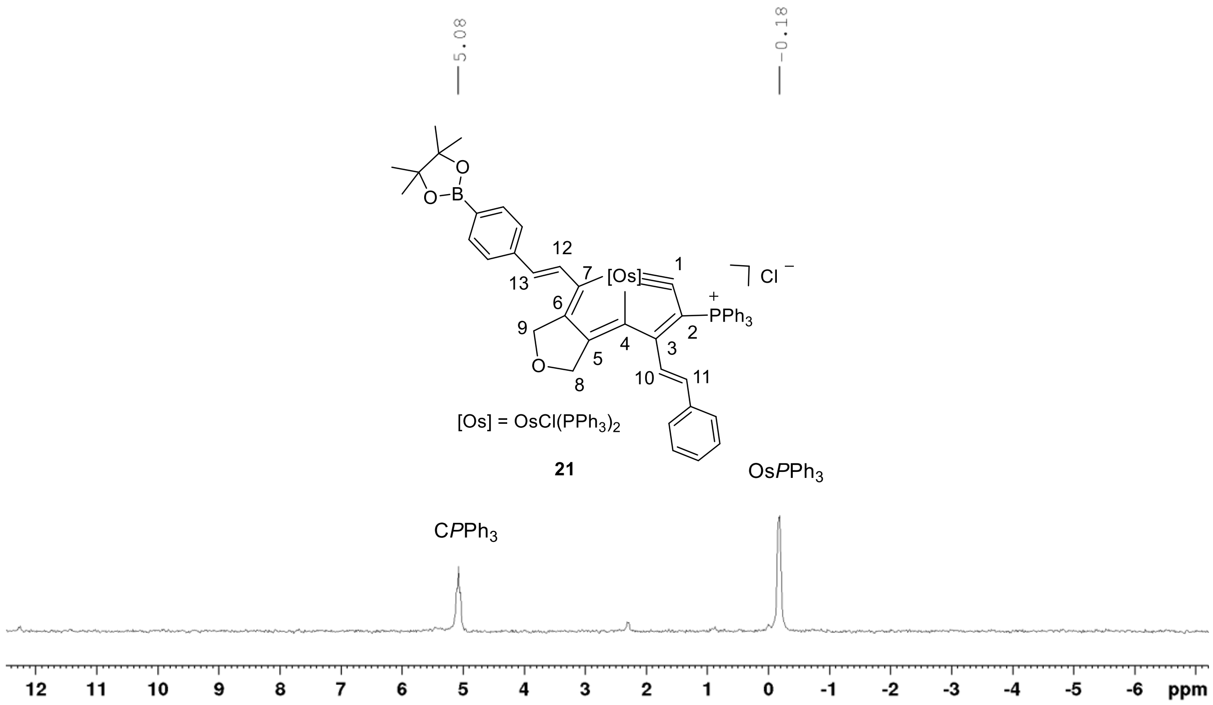

Supplementary Figure 141. The $^{31}P\{^{1}H\}$ NMR (242.9 MHz, CD_2Cl_2) spectrum for complex **21**.

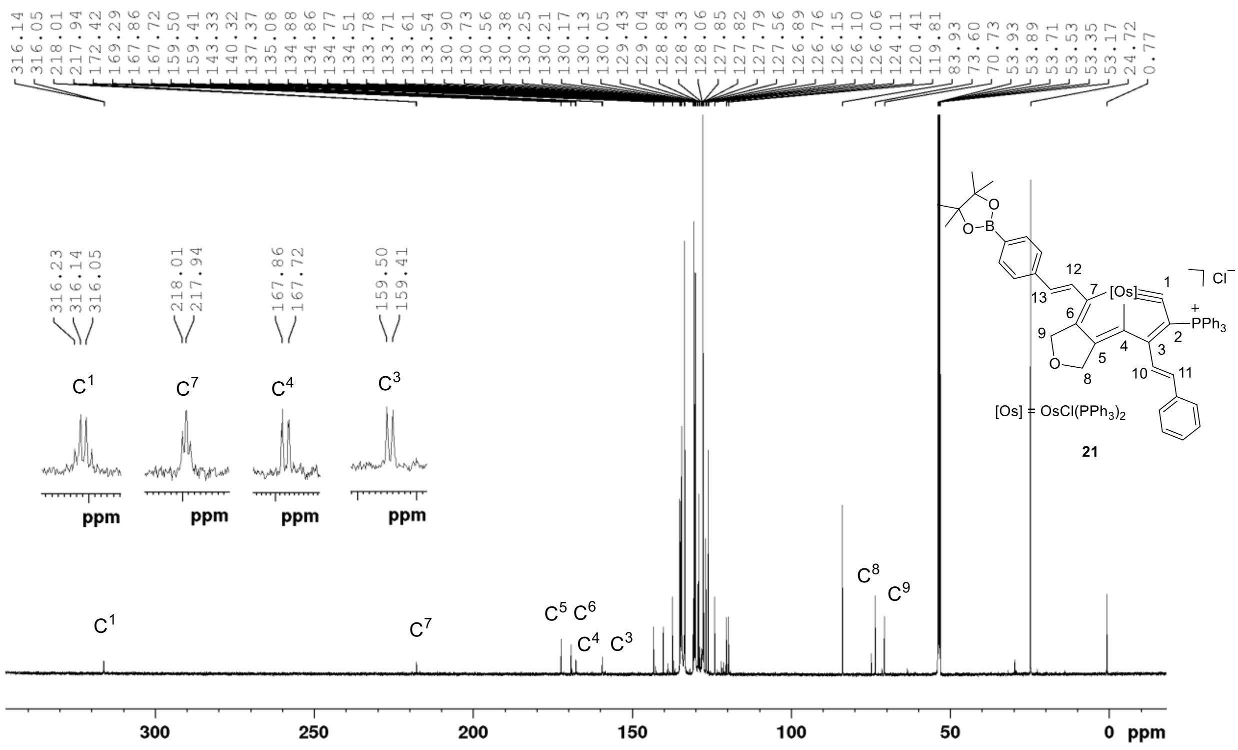

Supplementary Figure 142. The $^{13}C\{^1H\}$ NMR (150.9 MHz, CD_2Cl_2) spectrum for complex **21**.

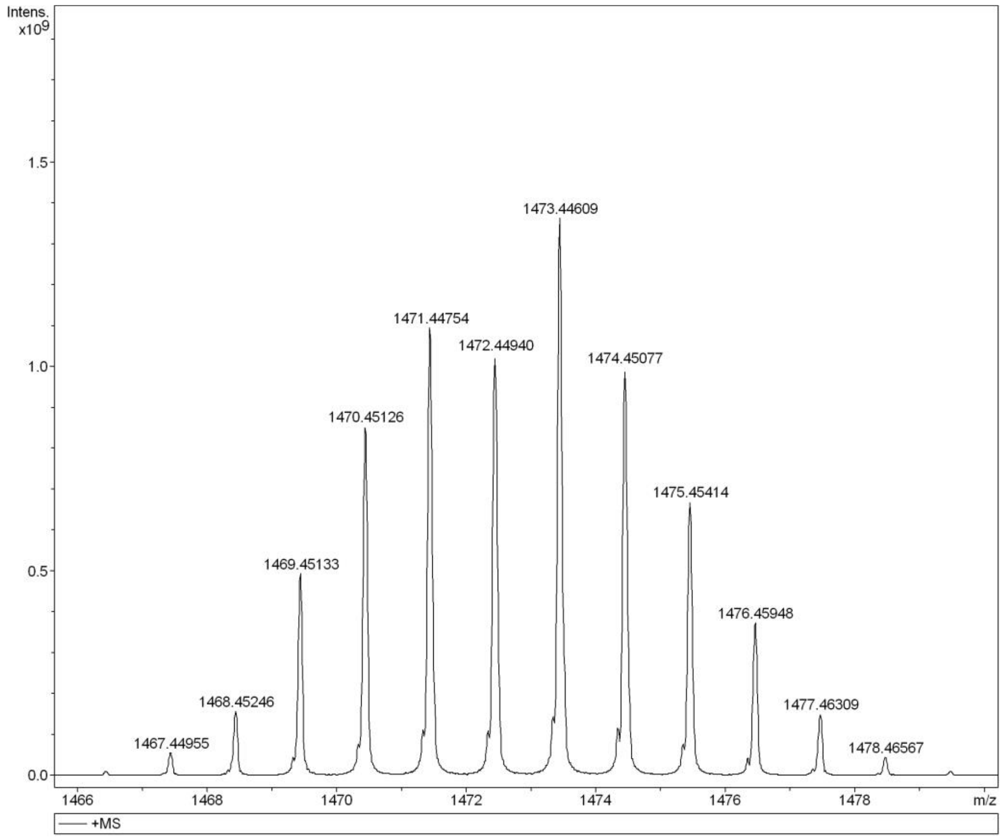

Supplementary Figure 143. Positive-ion ESI-MS spectrum of [**21**]$^+$ measured in methanol.

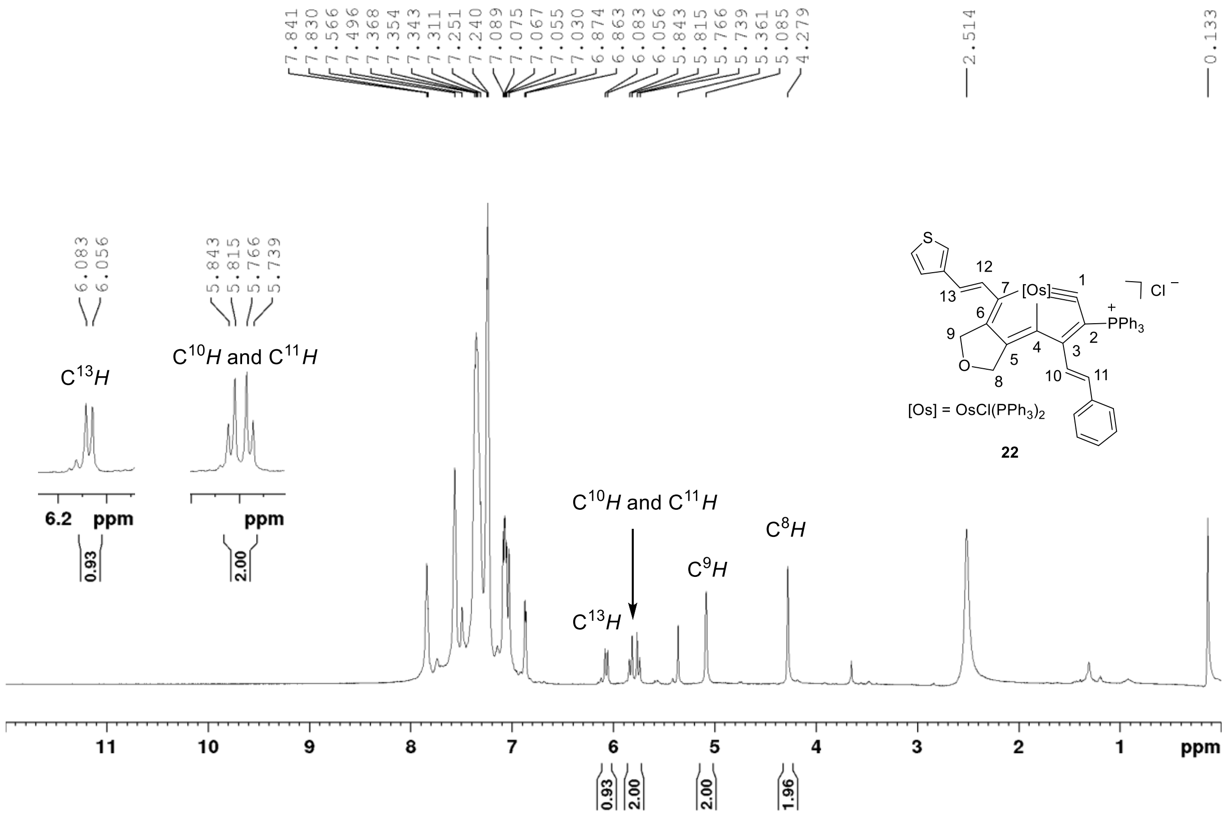

Supplementary Figure 144. The ^{1}H NMR (600.1 MHz, CD_2Cl_2) spectrum for complex **22**.

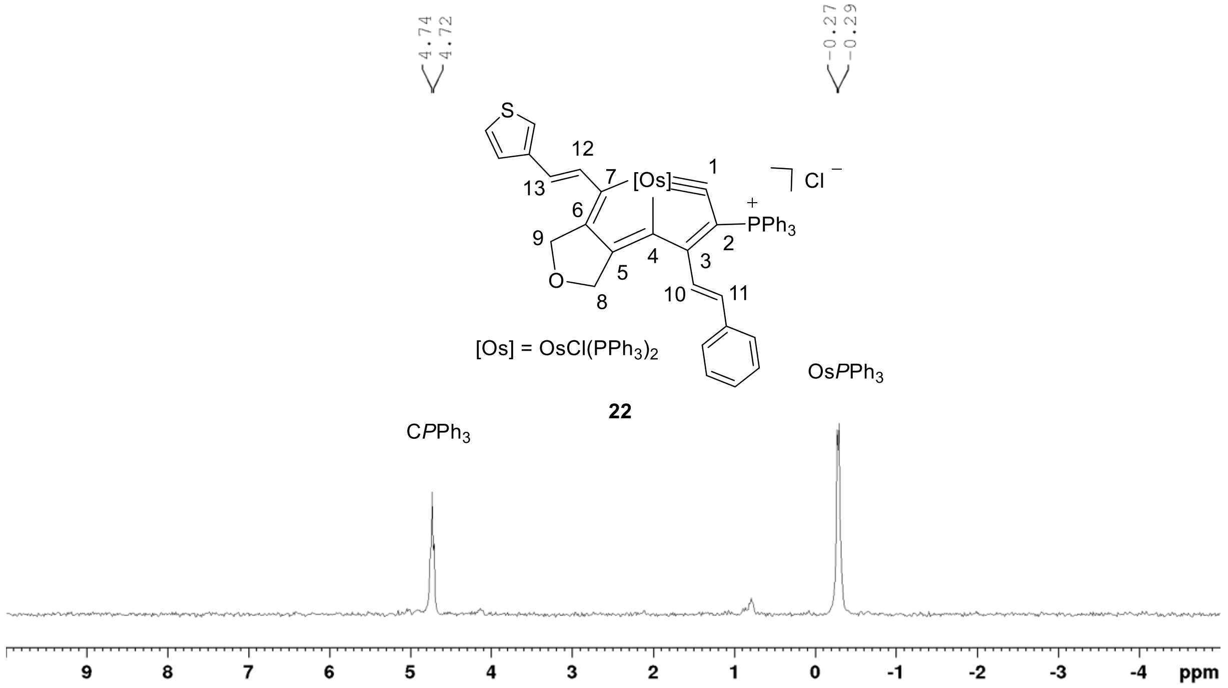

Supplementary Figure 145. The ^{31}P{^{1}H} NMR (242.9 MHz, CD_2Cl_2) spectrum for complex **22**.

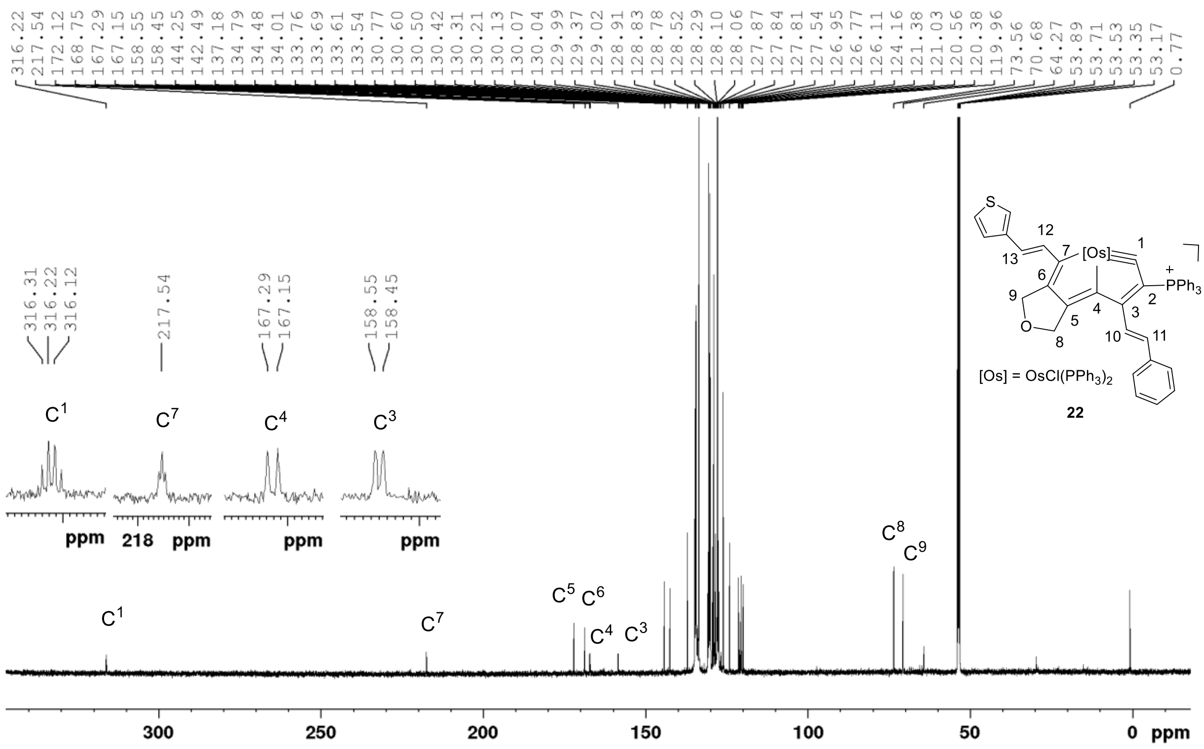

Supplementary Figure 146. The $^{13}C\{^{1}H\}$ NMR (150.9 MHz, CD_2Cl_2) spectrum for complex **22**.

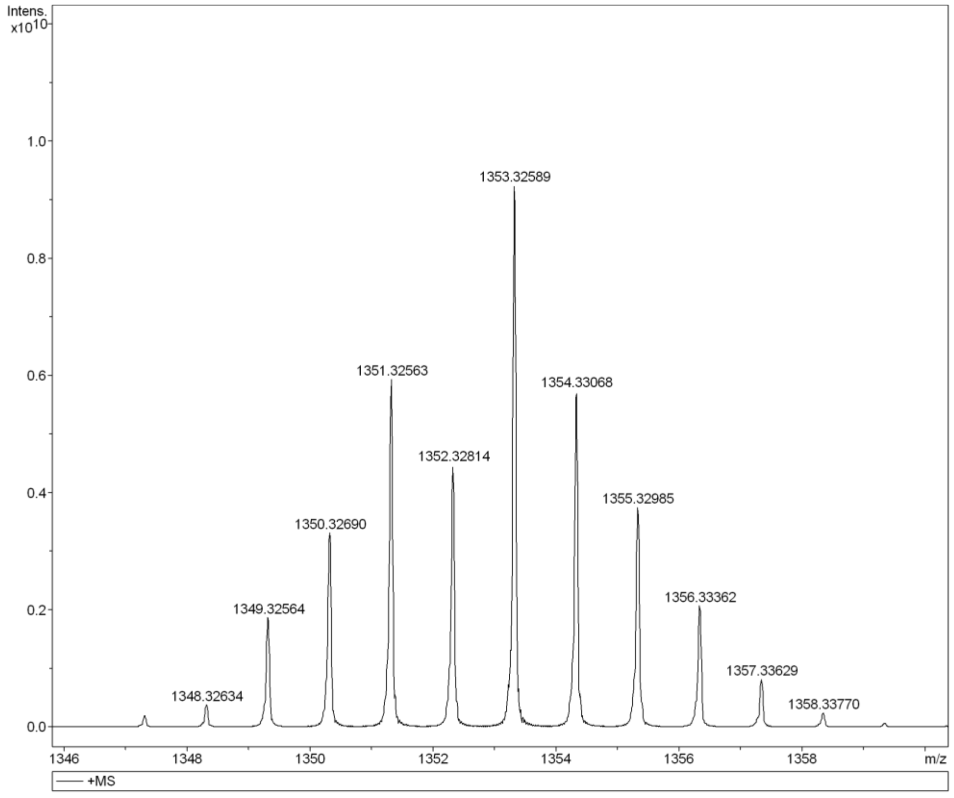

Supplementary Figure 147. Positive-ion ESI-MS spectrum of [**22**]$^+$ measured in methanol.

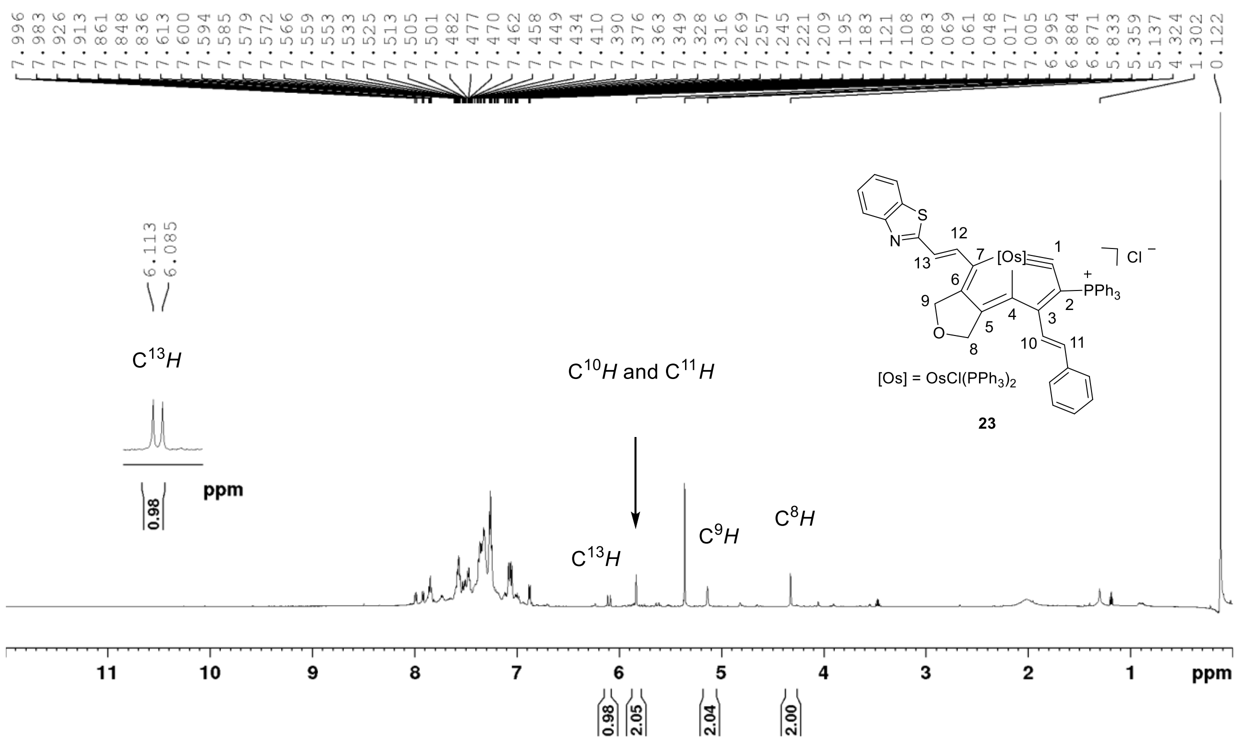

Supplementary Figure 148. The ^{1}H NMR (600.1 MHz, CD_2Cl_2) spectrum for complex **23**.

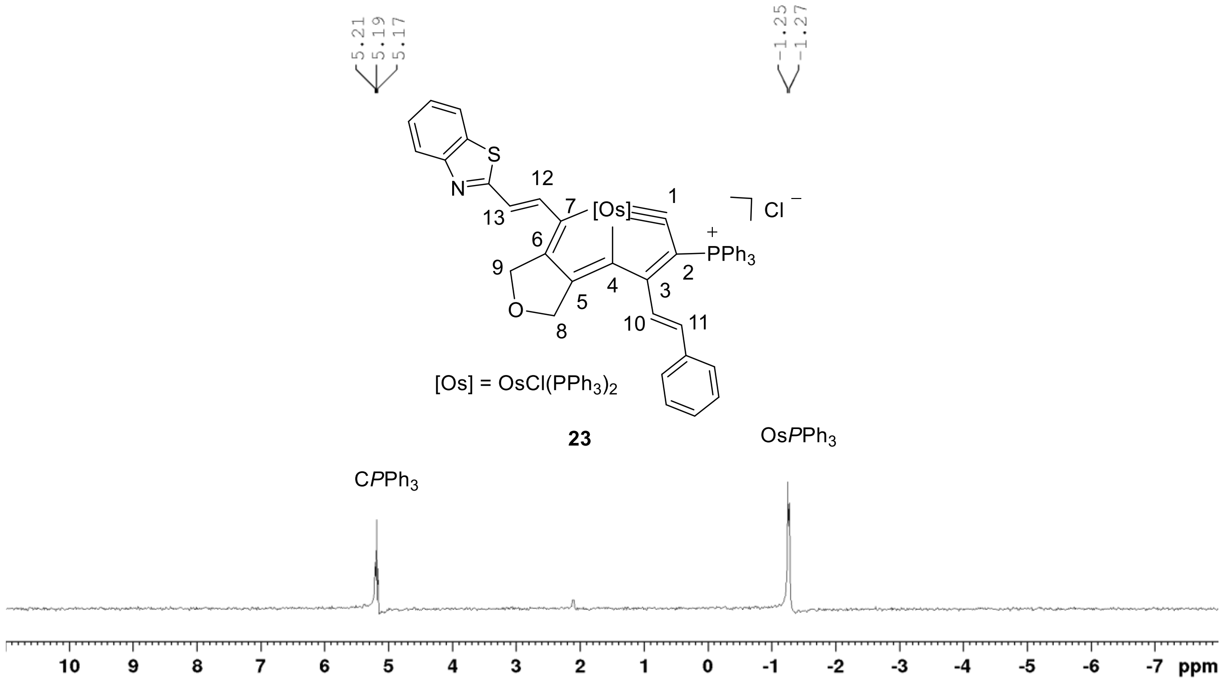

Supplementary Figure 149. The $^{31}P\{^{1}H\}$ NMR (242.9 MHz, CD_2Cl_2) spectrum for complex **23**.

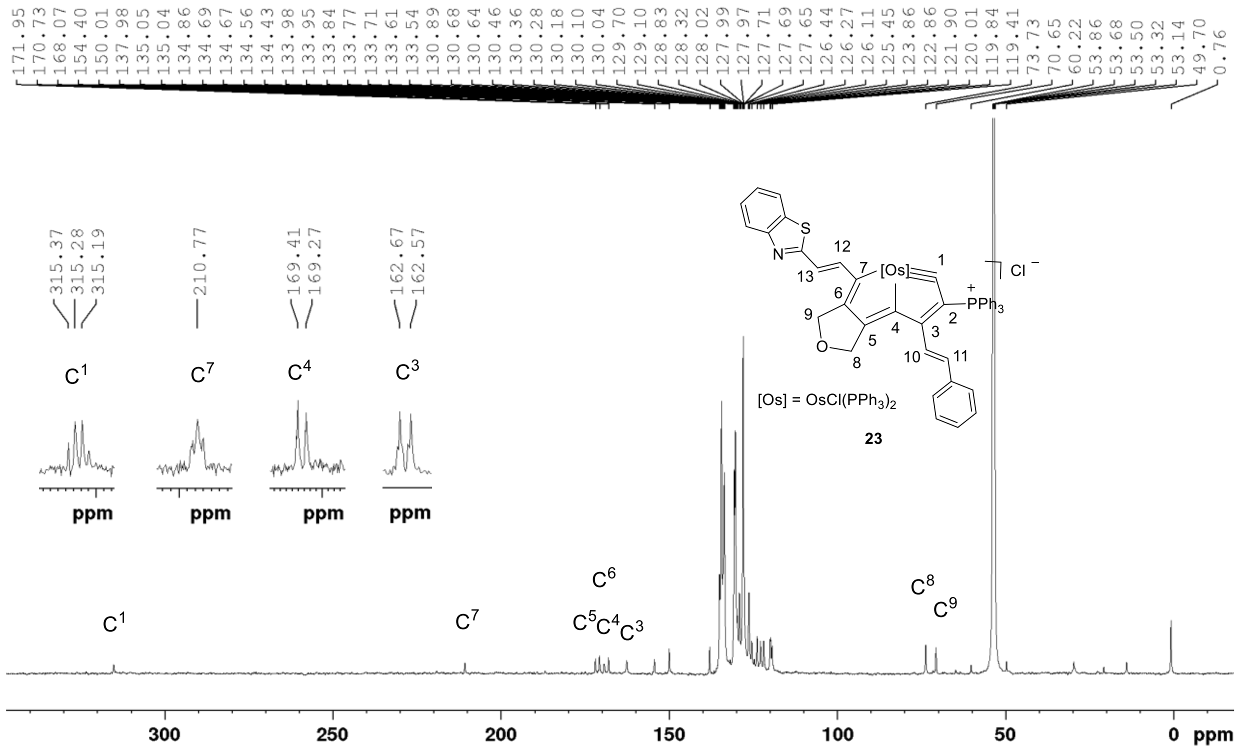

Supplementary Figure 150. The $^{13}C\{^{1}H\}$ NMR (150.9 MHz, CD_2Cl_2) spectrum for complex **23**.

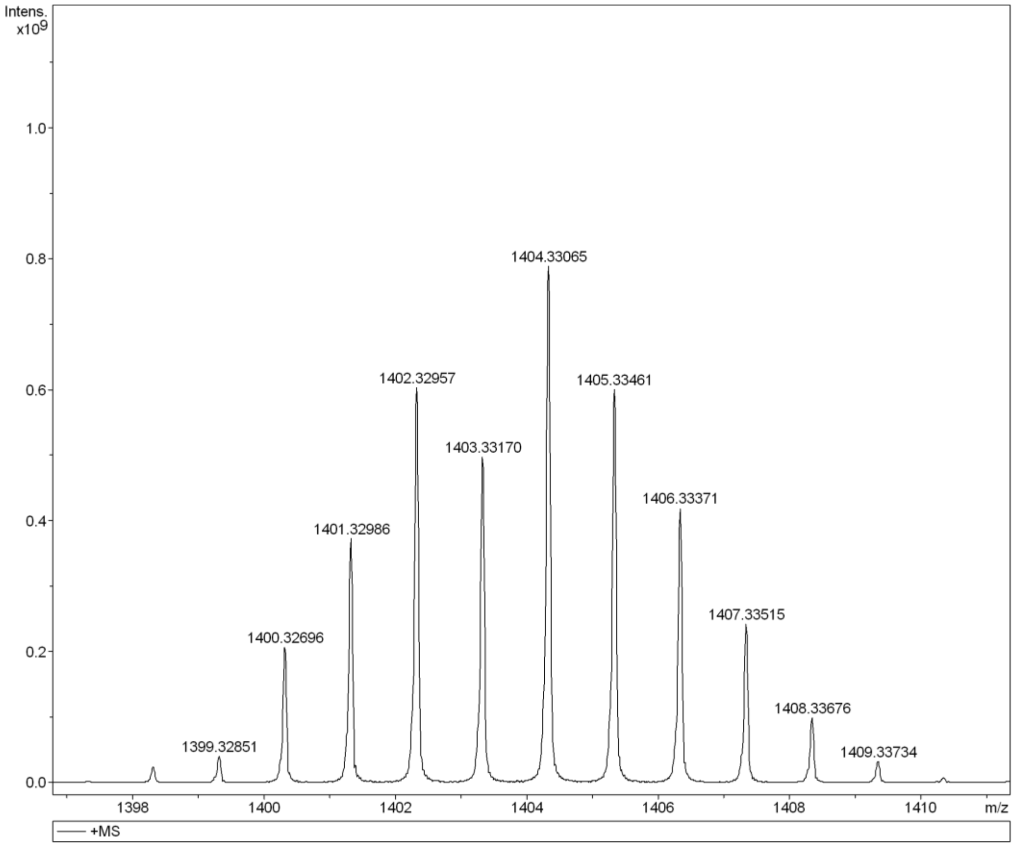

Supplementary Figure 151. Positive-ion ESI-MS spectrum of [**23**]$^{+}$ measured in methanol.

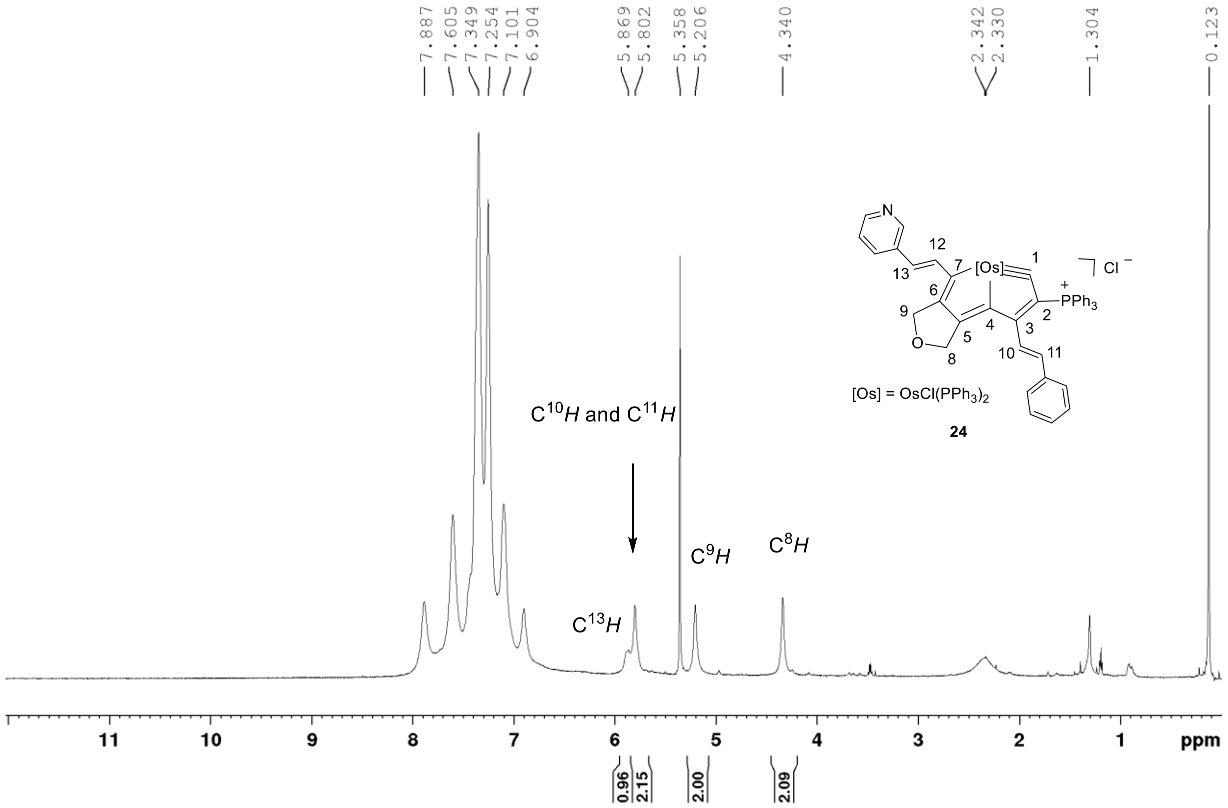

Supplementary Figure 152. The ^{1}H NMR (600.1 MHz, CD_2Cl_2) spectrum for complex **24**.

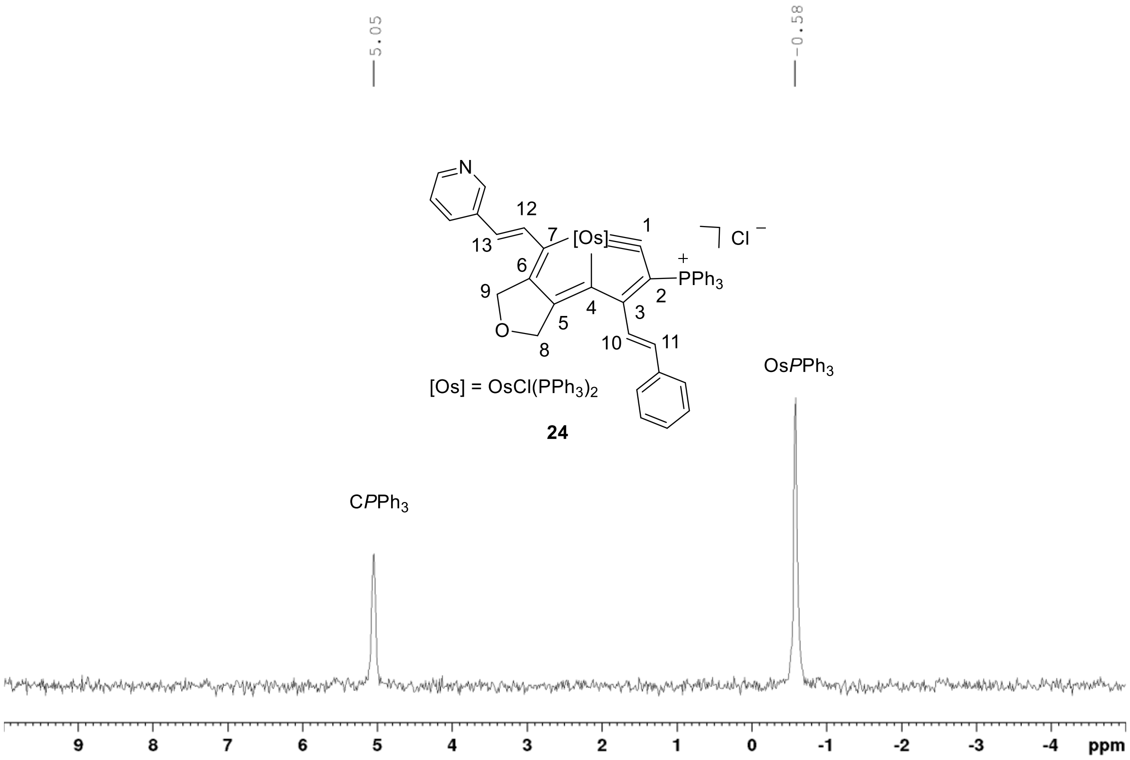

Supplementary Figure 153. The $^{31}P\{^{1}H\}$ NMR (242.9 MHz, CD_2Cl_2) spectrum for complex **24**.

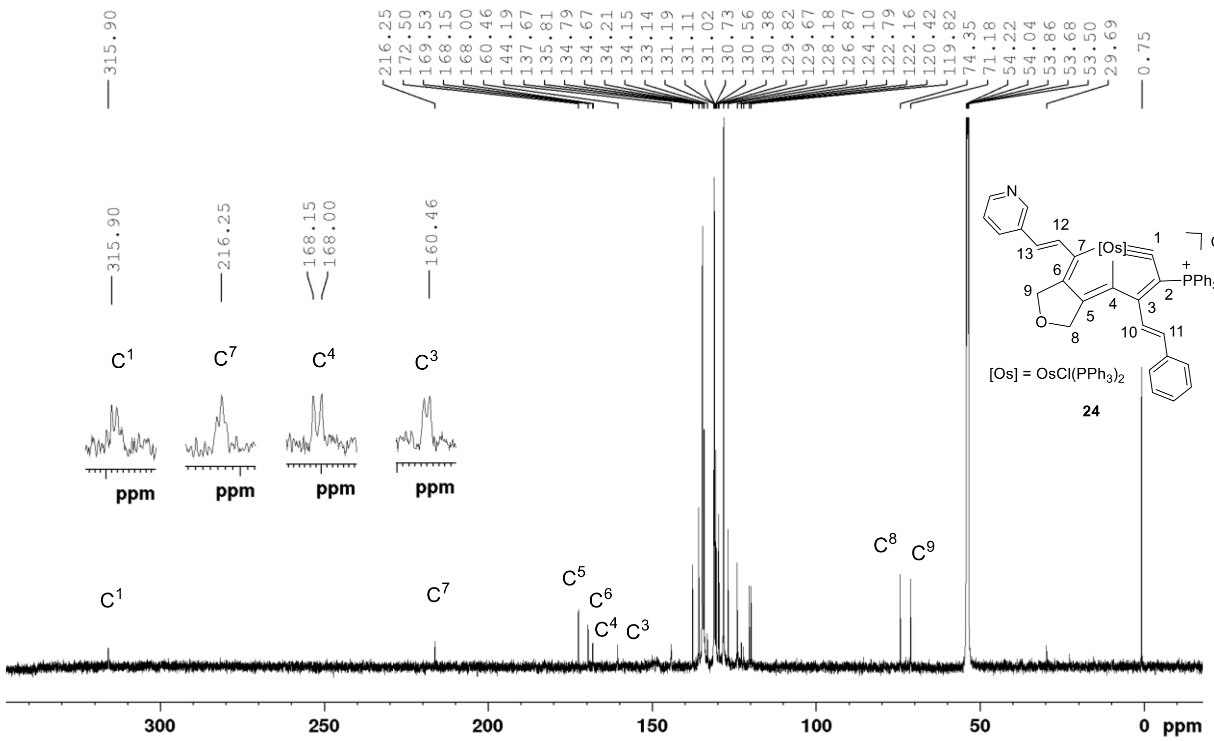

Supplementary Figure 154. The $^{13}C\{^{1}H\}$ NMR (150.9 MHz, CD_2Cl_2) spectrum for complex **24**.

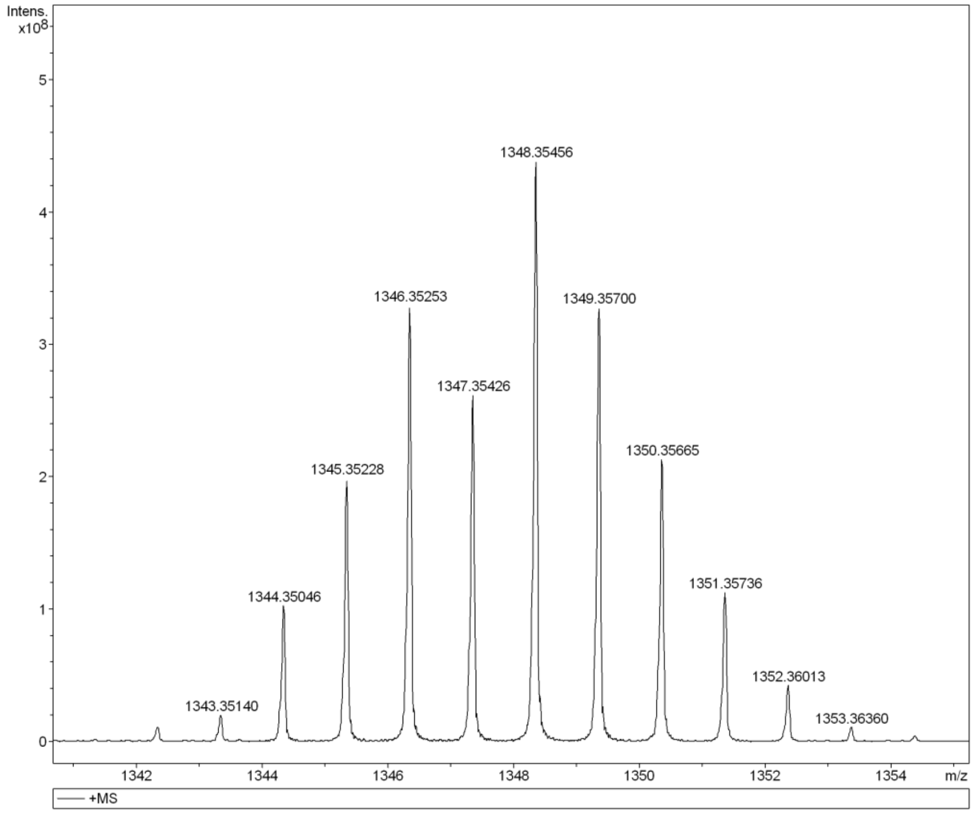

Supplementary Figure 155. Positive-ion ESI-MS spectrum of [**24**]$^{+}$ measured in methanol.

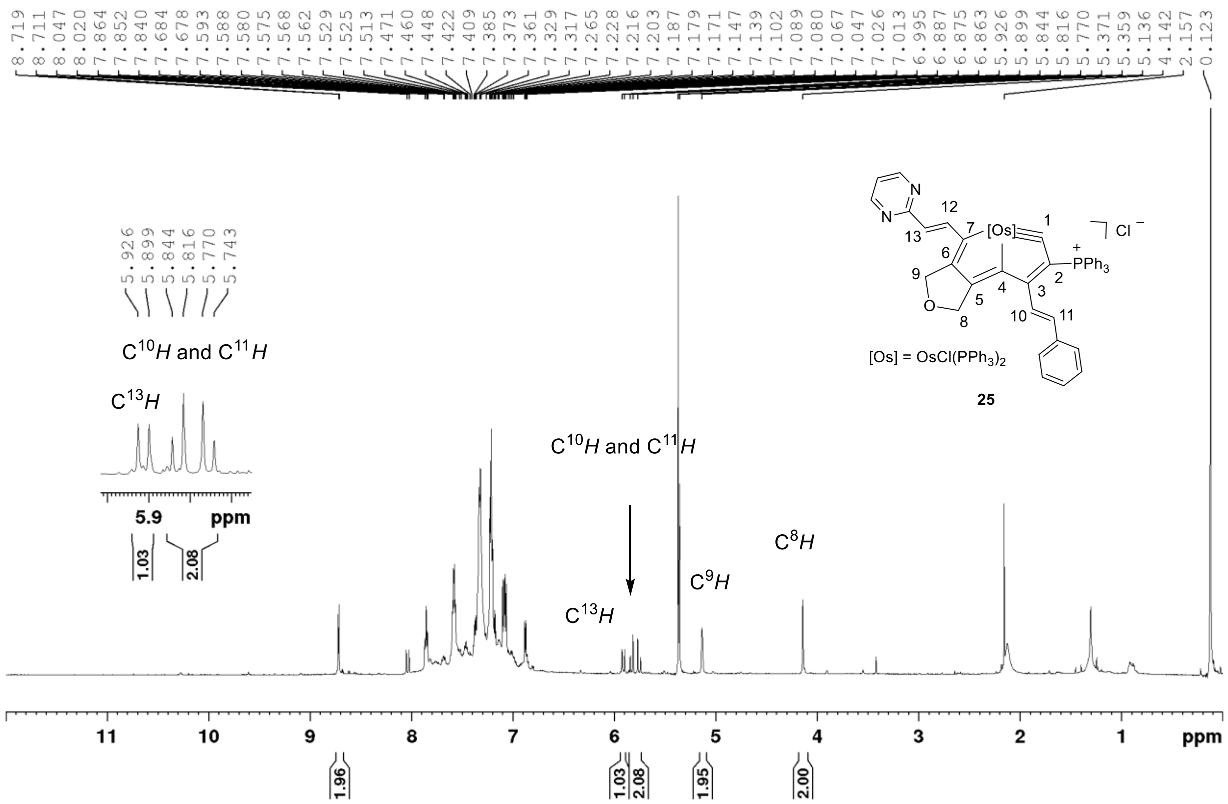

Supplementary Figure 156. The ^{1}H NMR (600.1 MHz, CD_2Cl_2) spectrum for complex **25**.

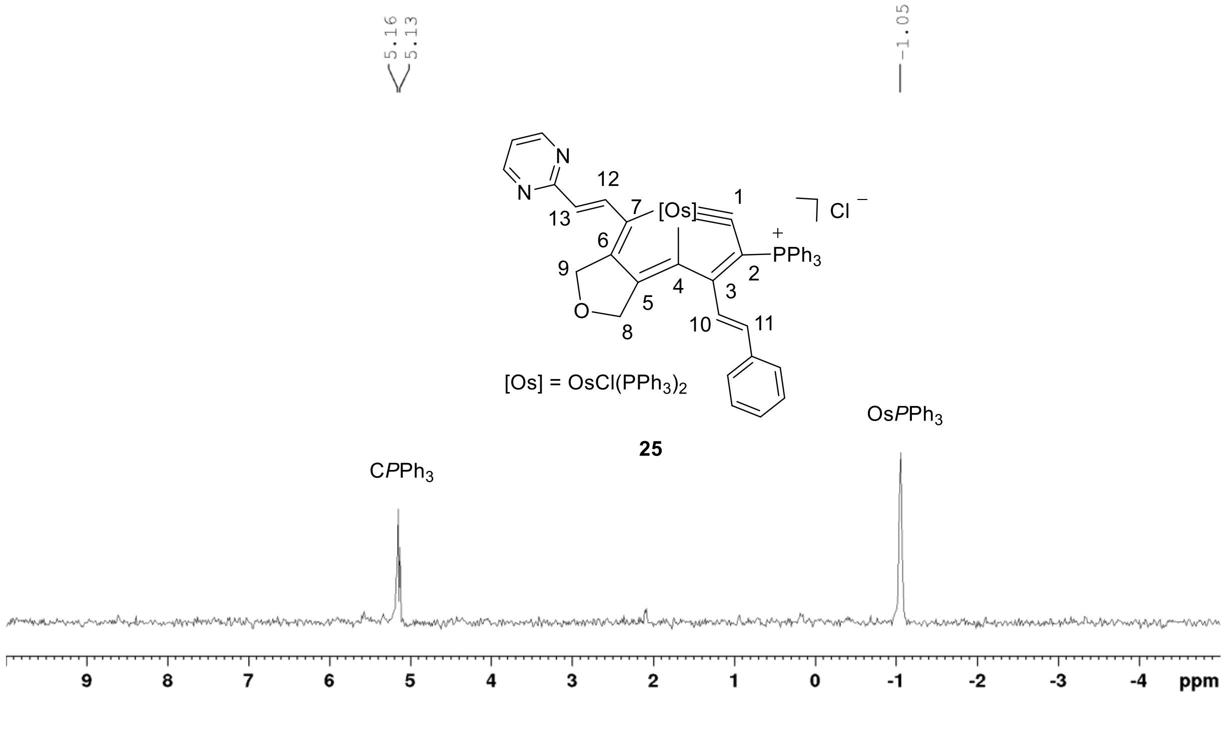

Supplementary Figure 157. The ^{31}P{^{1}H} NMR (242.9 MHz, CD_2Cl_2) spectrum for complex **25**.

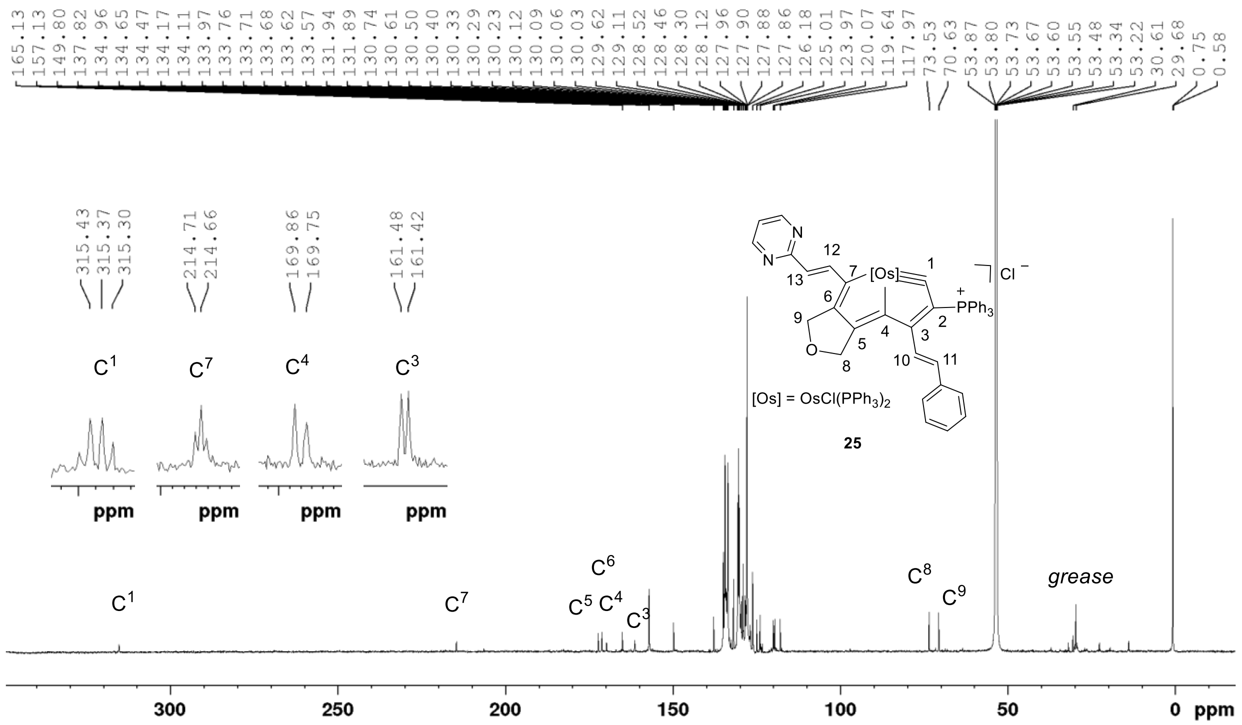

Supplementary Figure 158. The $^{13}C\{^{1}H\}$ NMR (150.9 MHz, CD_2Cl_2) spectrum for complex **25**.

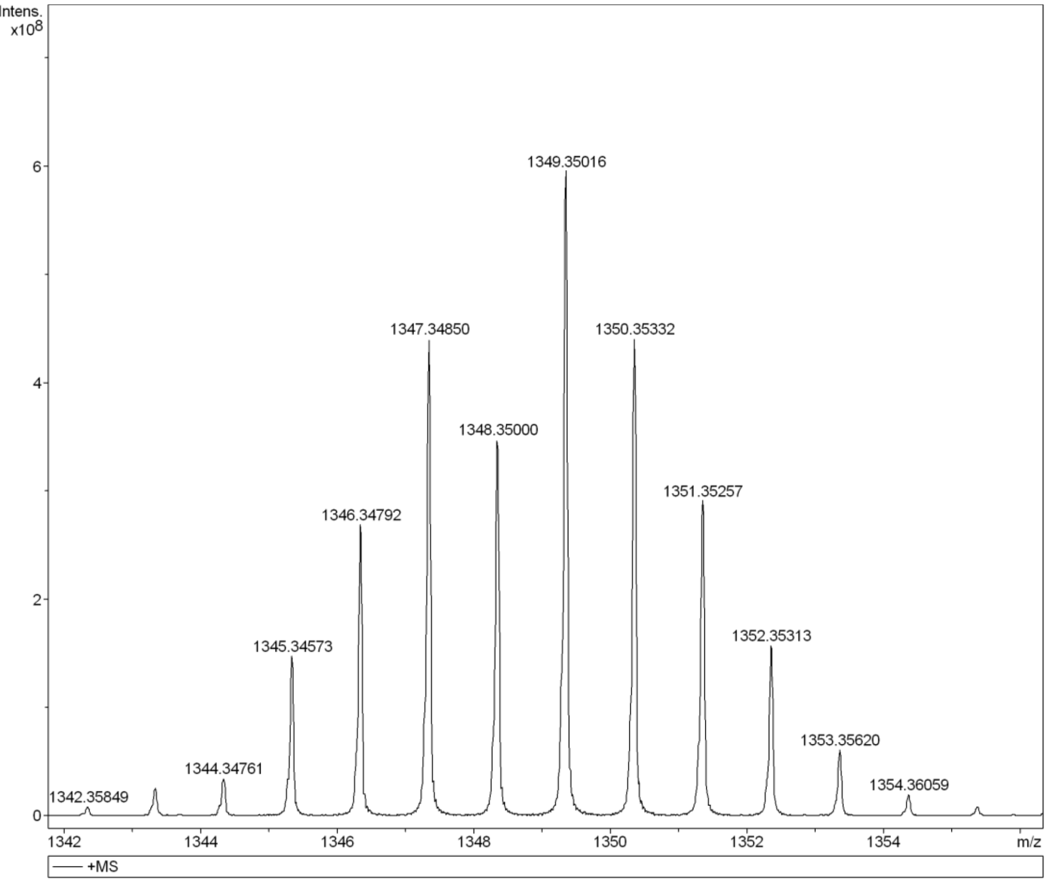

Supplementary Figure 159. Positive-ion ESI-MS spectrum of [**25**]$^{+}$ measured in methanol.

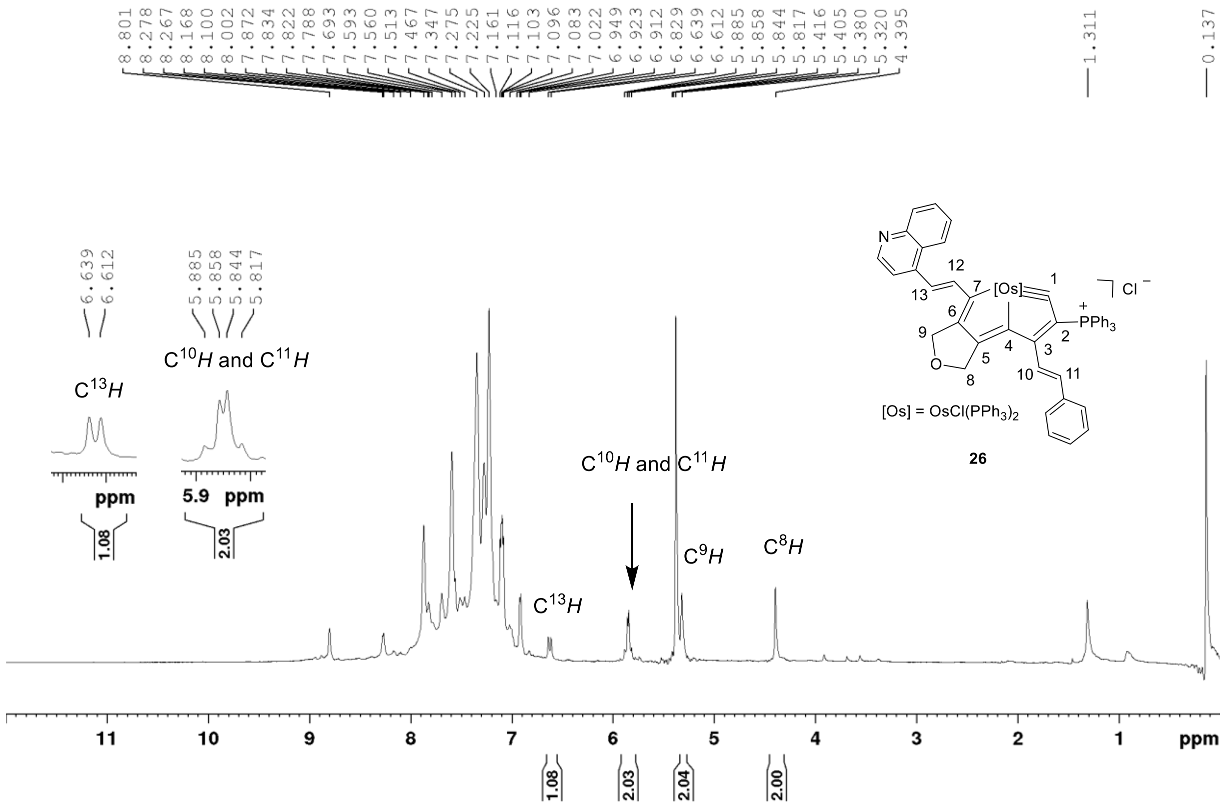

Supplementary Figure 160. The ^{1}H NMR (600.1 MHz, CD_2Cl_2) spectrum for complex **26**.

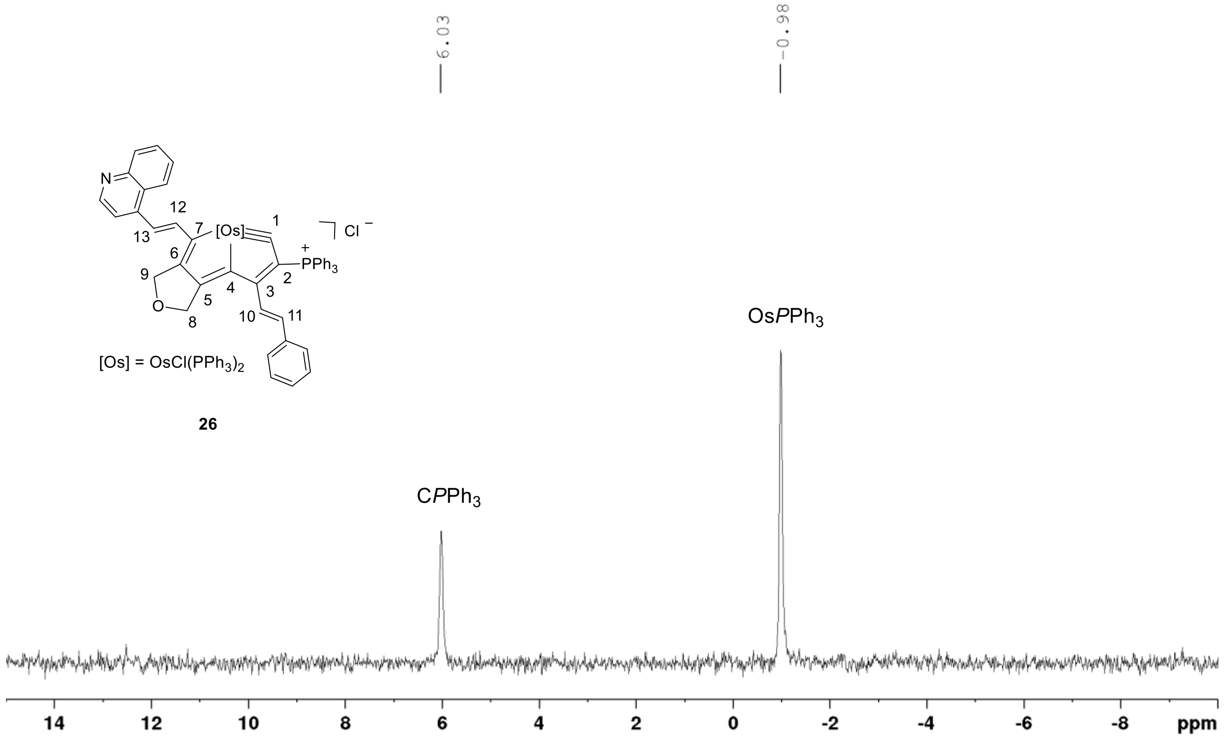

Supplementary Figure 161. The $^{31}P\{^{1}H\}$ NMR (242.9 MHz, CD_2Cl_2) spectrum for complex **26**.

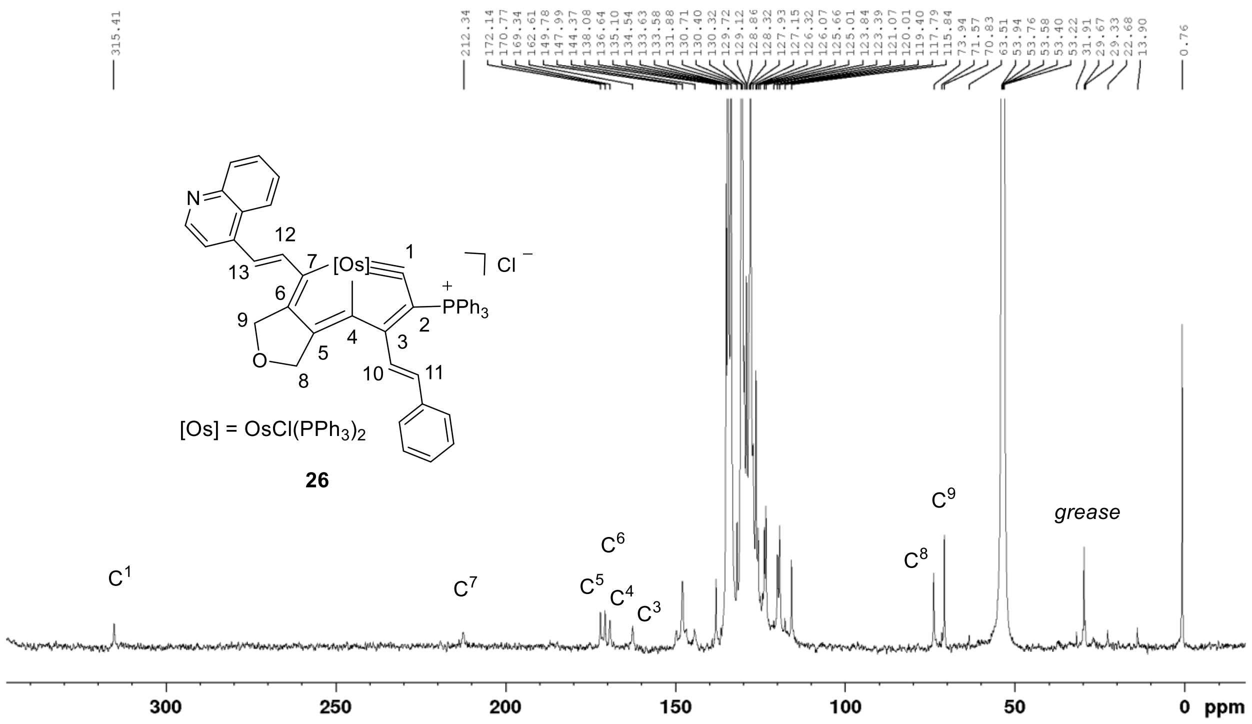

Supplementary Figure 162. The $^{13}C\{^{1}H\}$ NMR (150.9 MHz, CD_2Cl_2) spectrum for complex **26**.

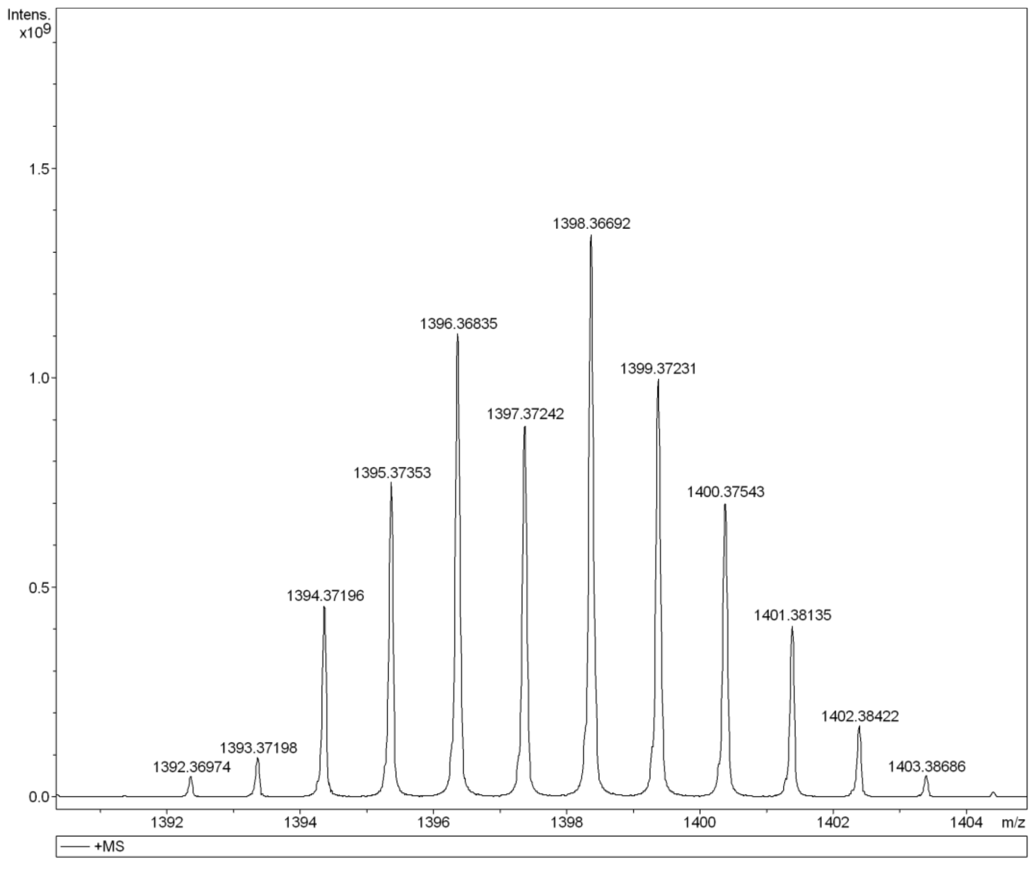

Supplementary Figure 163. Positive-ion ESI-MS spectrum of [**26**]$^+$ measured in methanol.

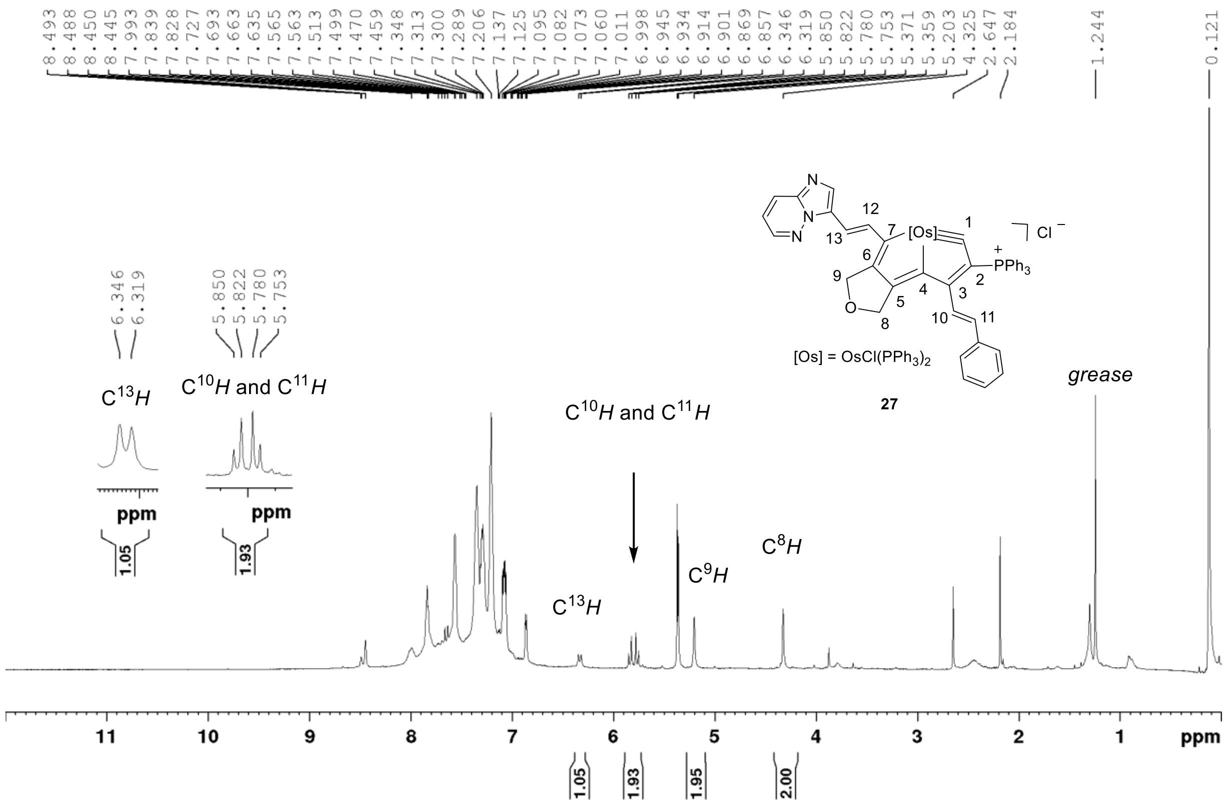

Supplementary Figure 164. The ^{1}H NMR (600.1 MHz, CD_2Cl_2) spectrum for complex **27**.

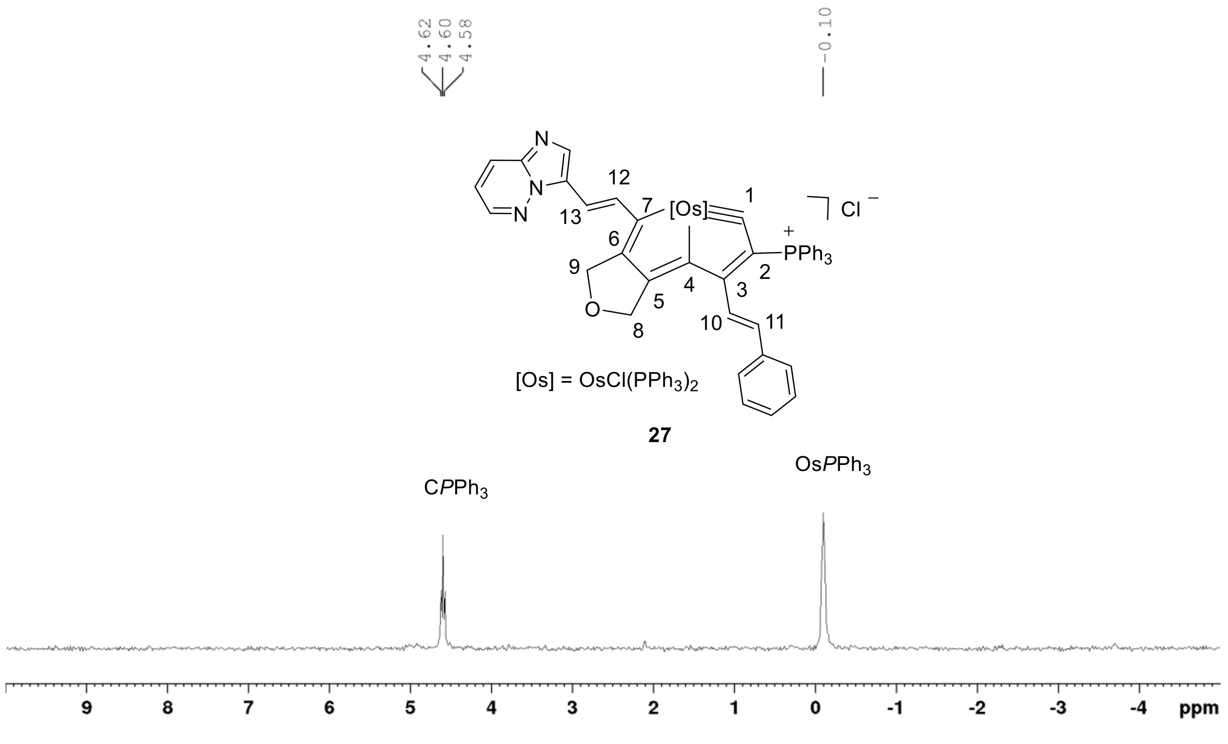

Supplementary Figure 165. The $^{31}P\{^1H\}$ NMR (242.9 MHz, CD_2Cl_2) spectrum for complex **27**.

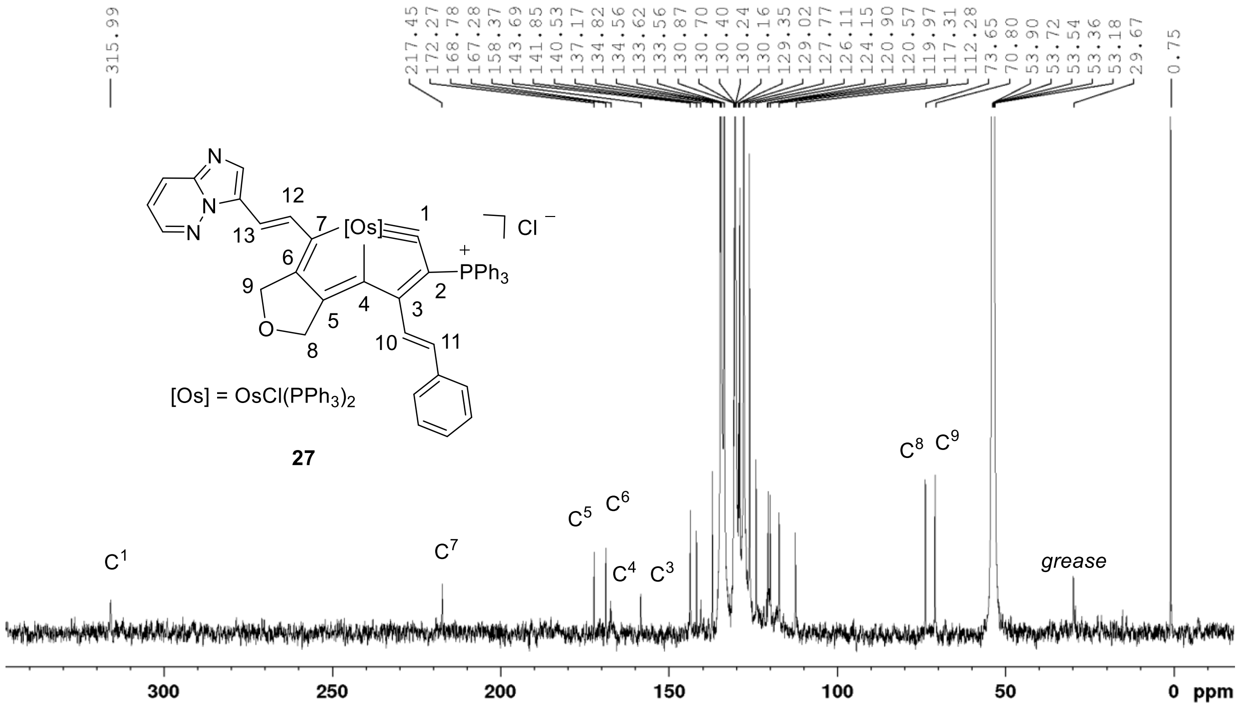

Supplementary Figure 166. The $^{13}C\{^{1}H\}$ NMR (150.9 MHz, CD_2Cl_2) spectrum for complex **27**.

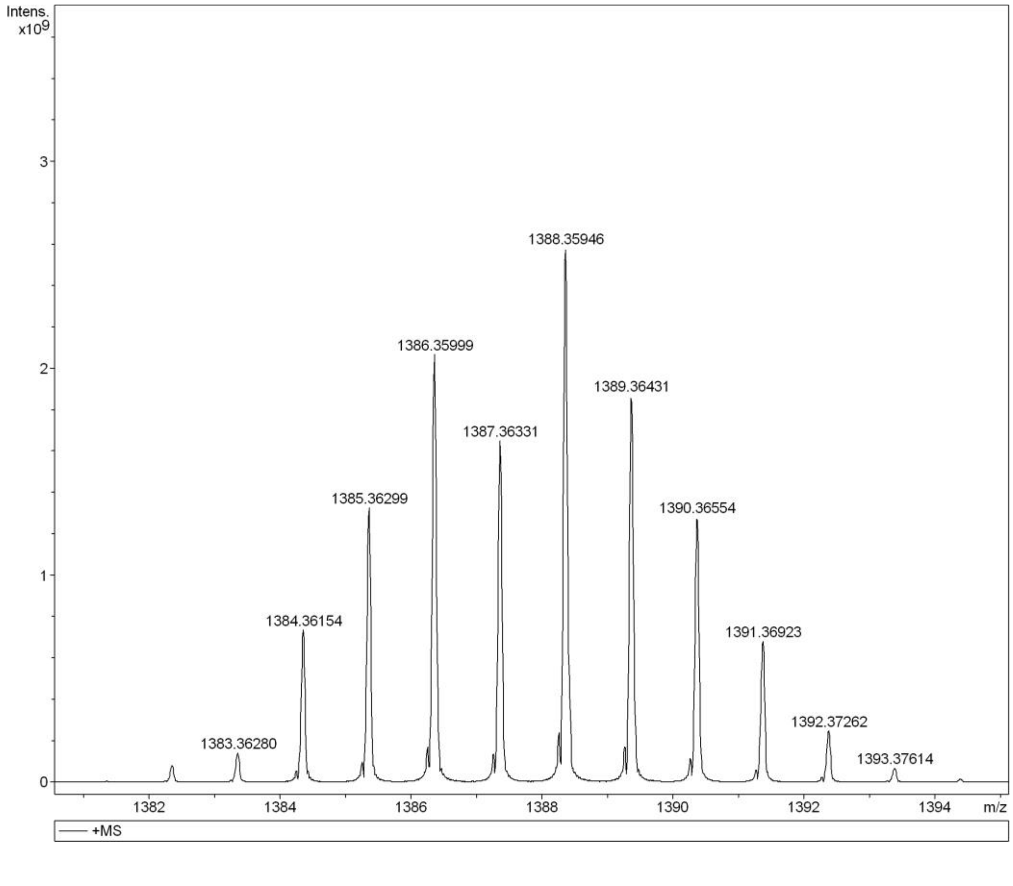

Supplementary Figure 167. Positive-ion ESI-MS spectrum of $[\mathbf{27}]^+$ measured in methanol.

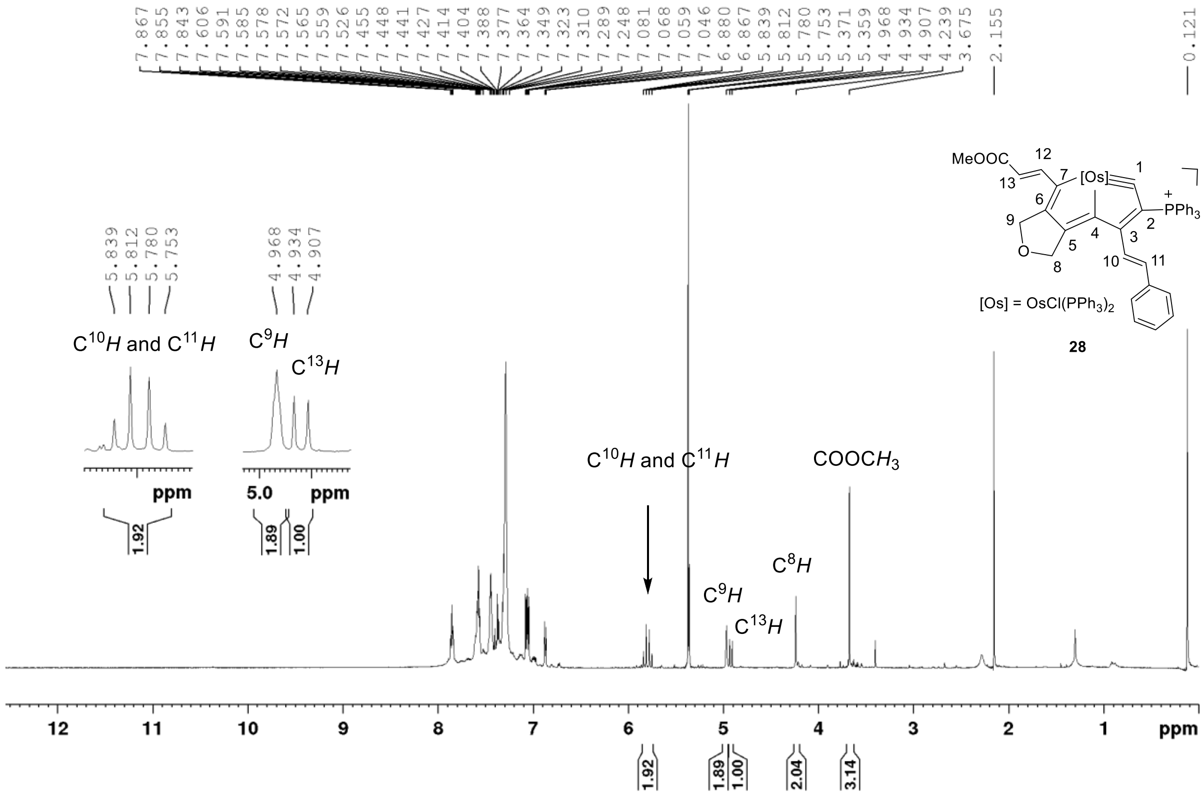

Supplementary Figure 168. The ^{1}H NMR (600.1 MHz, CD_2Cl_2) spectrum for complex **28**.

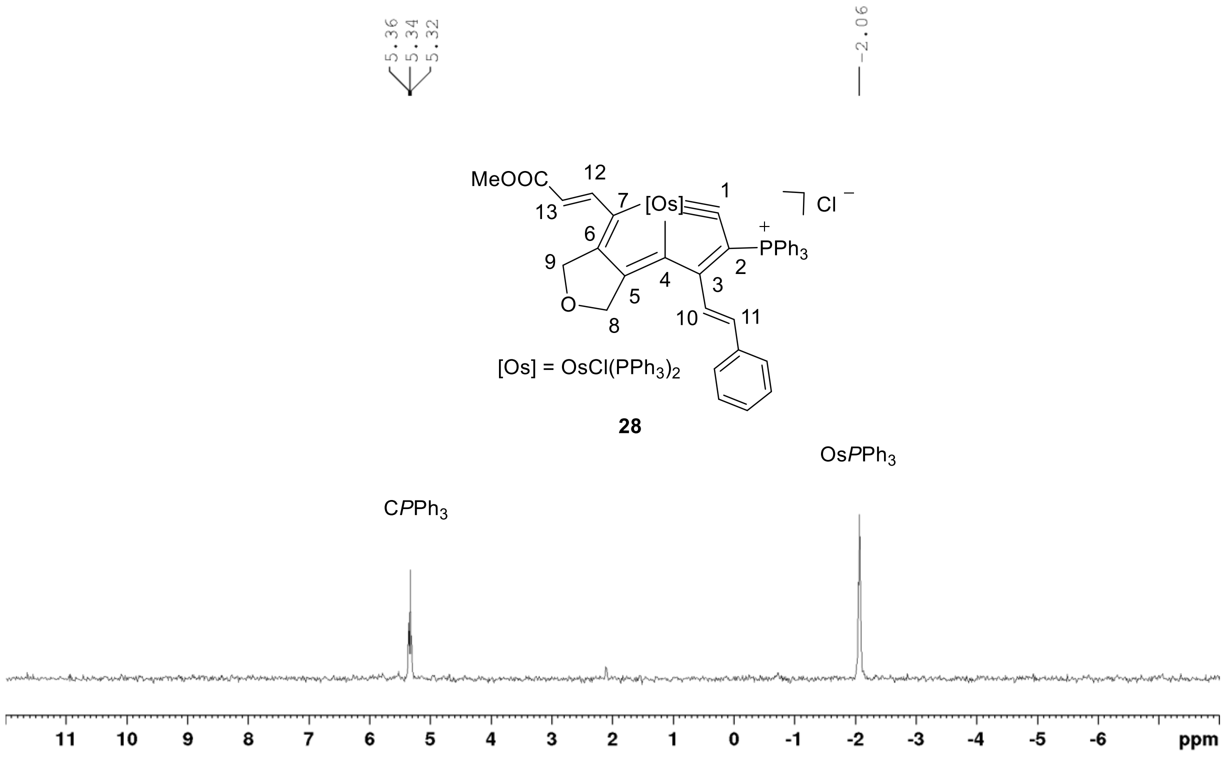

Supplementary Figure 169. The $^{31}P\{^{1}H\}$ NMR (242.9 MHz, CD_2Cl_2) spectrum for complex **28**.

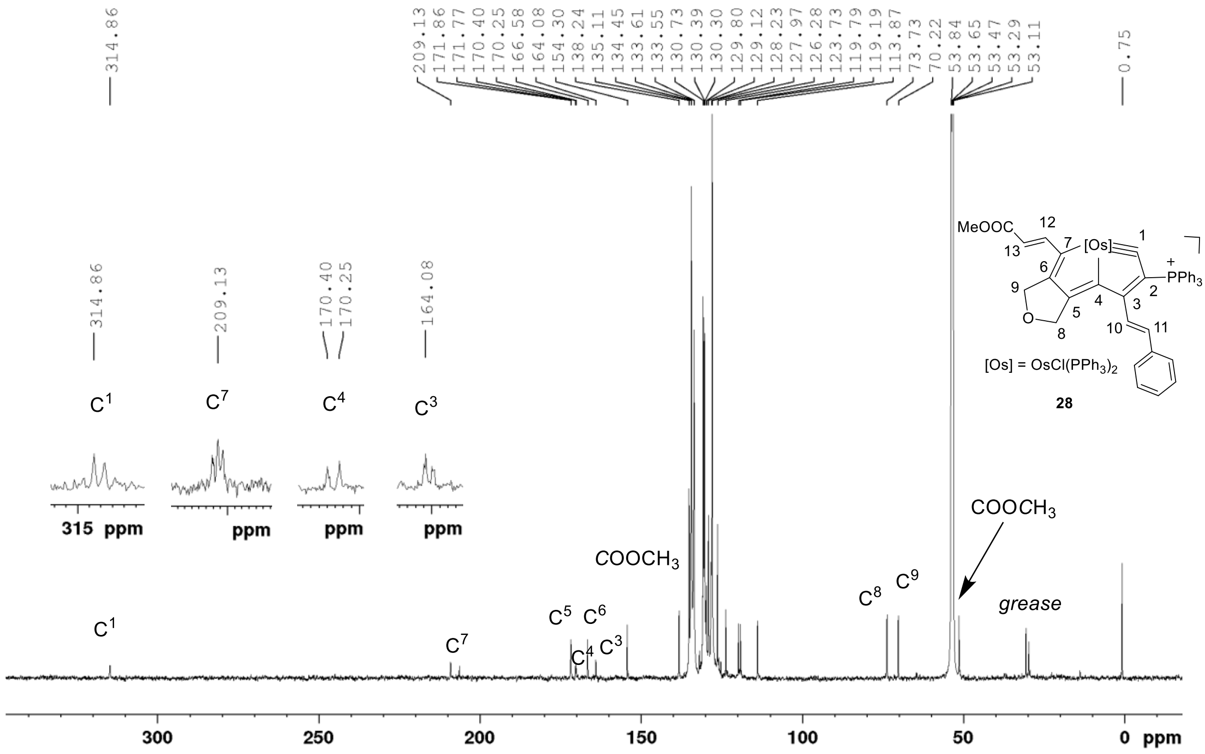

Supplementary Figure 170. The $^{13}C\{^1H\}$ NMR (150.9 MHz, CD_2Cl_2) spectrum for complex **28**.

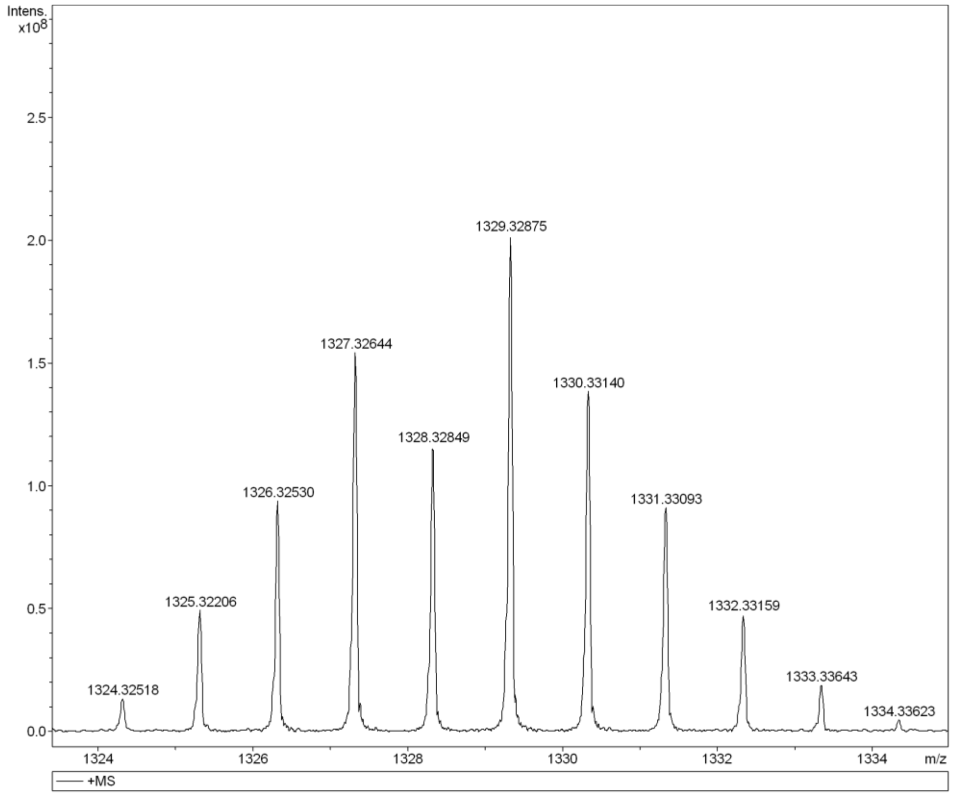

Supplementary Figure 171. Positive-ion ESI-MS spectrum of [**28**]$^+$ measured in methanol.

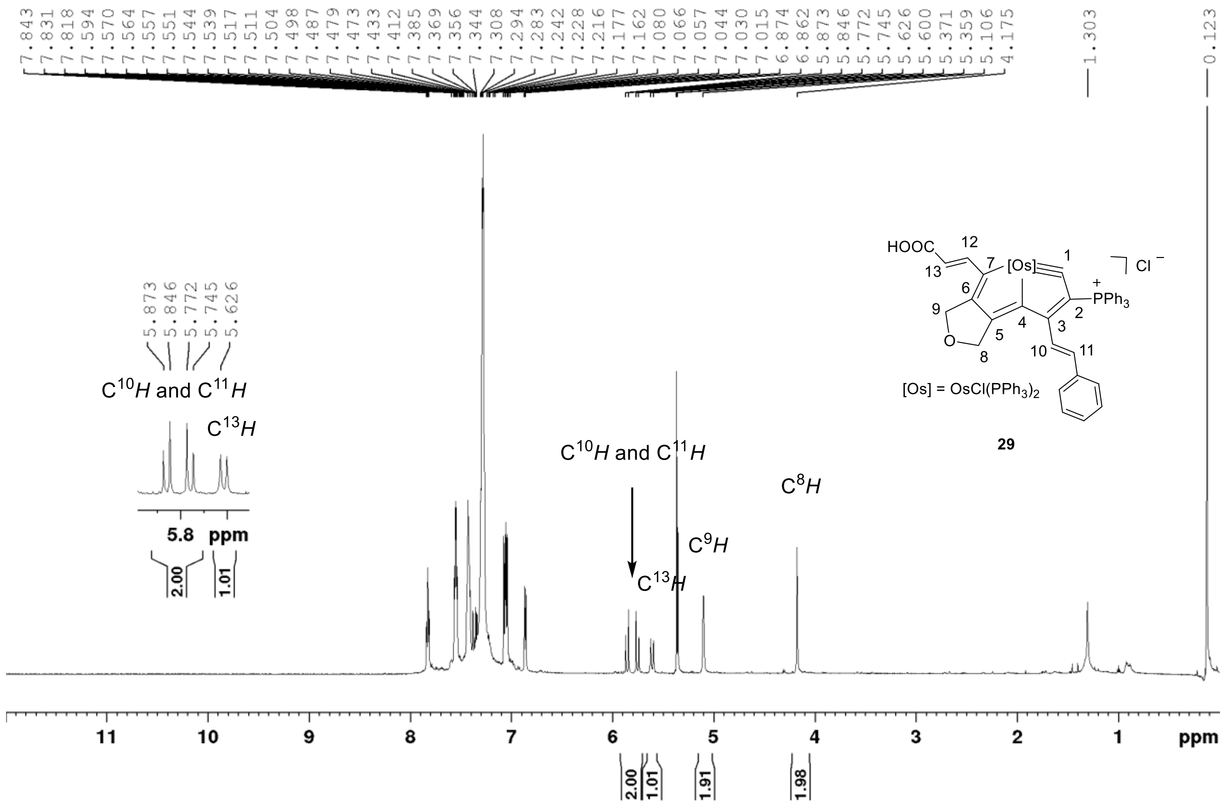

Supplementary Figure 172. The ^{1}H NMR (600.1 MHz, CD_2Cl_2) spectrum for complex **29**.

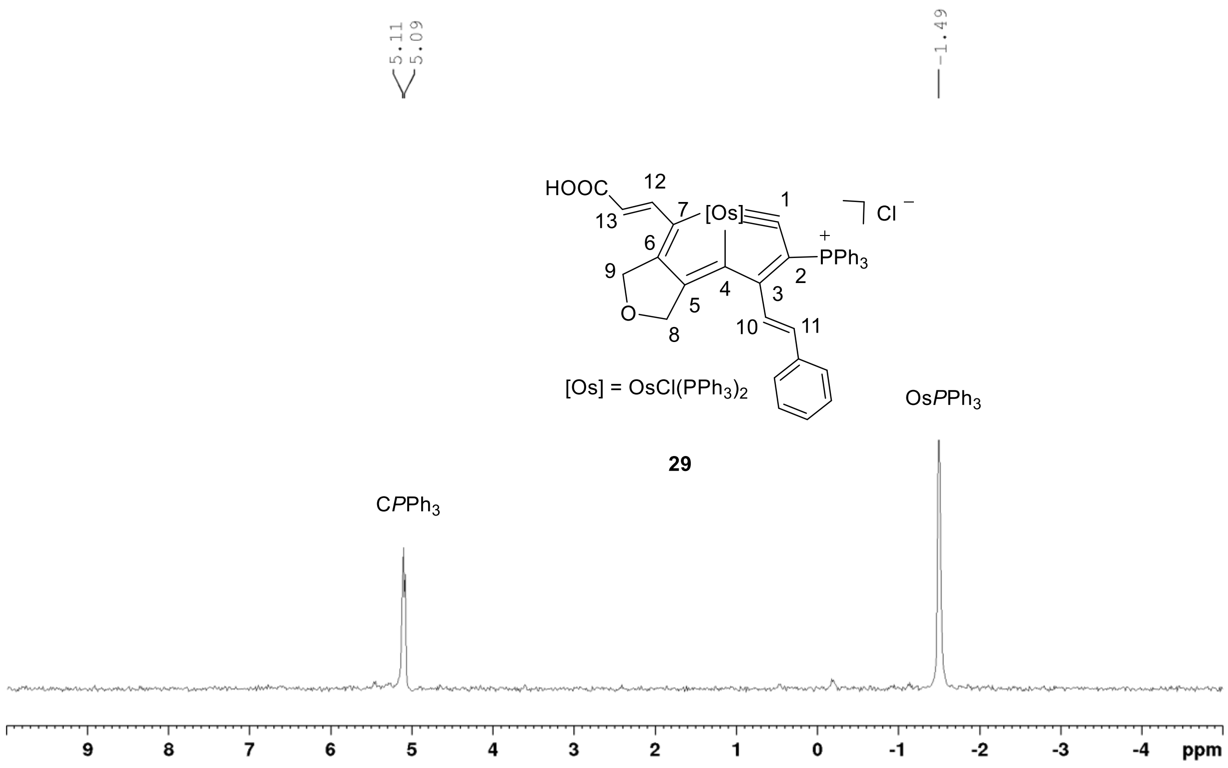

Supplementary Figure 173. The ^{31}P{^{1}H} NMR (242.9 MHz, CD_2Cl_2) spectrum for complex **29**.

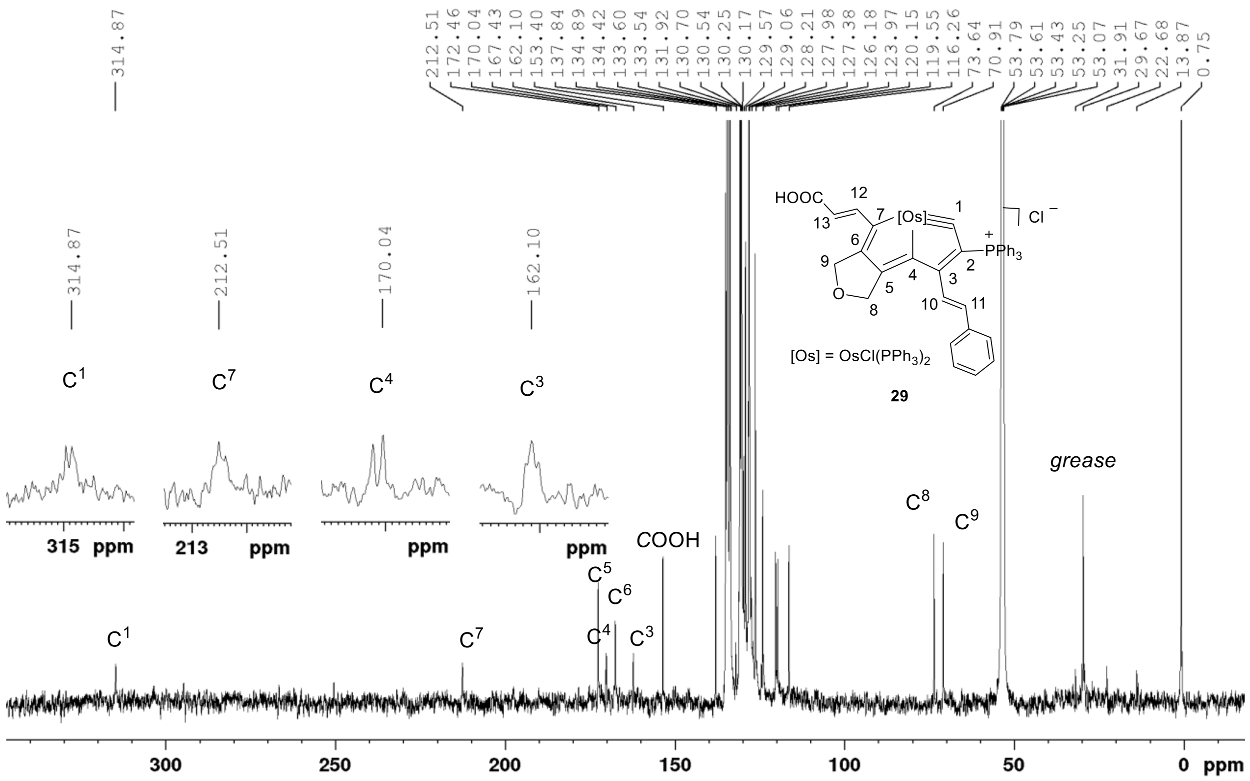

Supplementary Figure 174. The $^{13}C\{^{1}H\}$ NMR (150.9 MHz, CD_2Cl_2) spectrum for complex **29**.

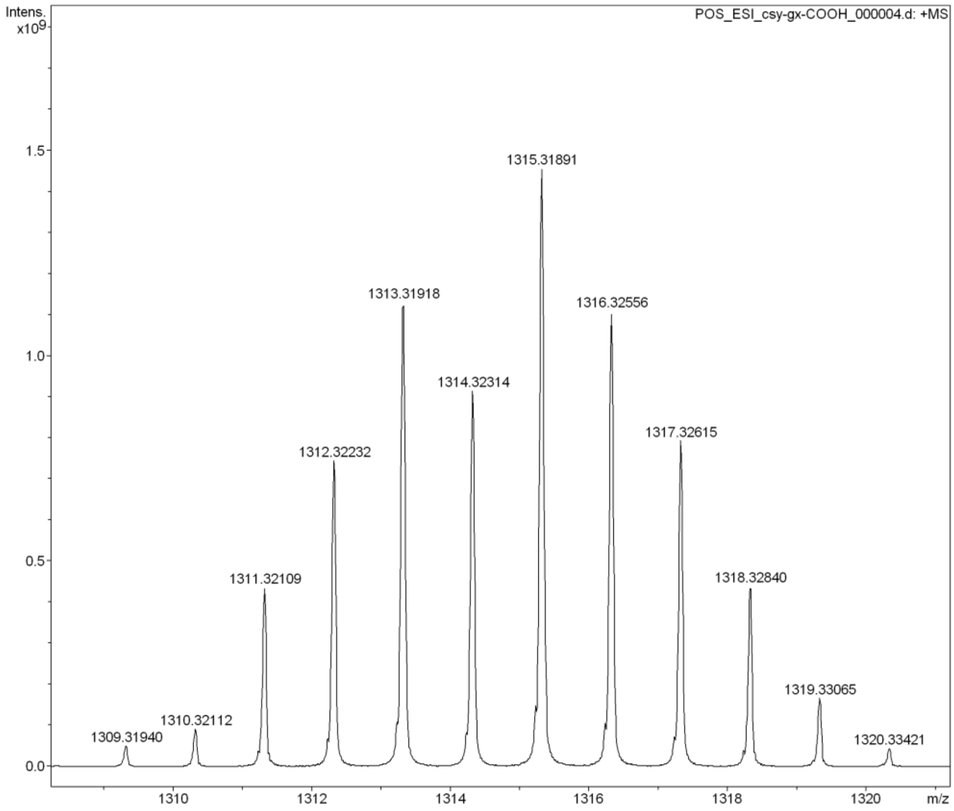

Supplementary Figure 175. Positive-ion ESI-MS spectrum of $[\mathbf{29}]^+$ measured in methanol.

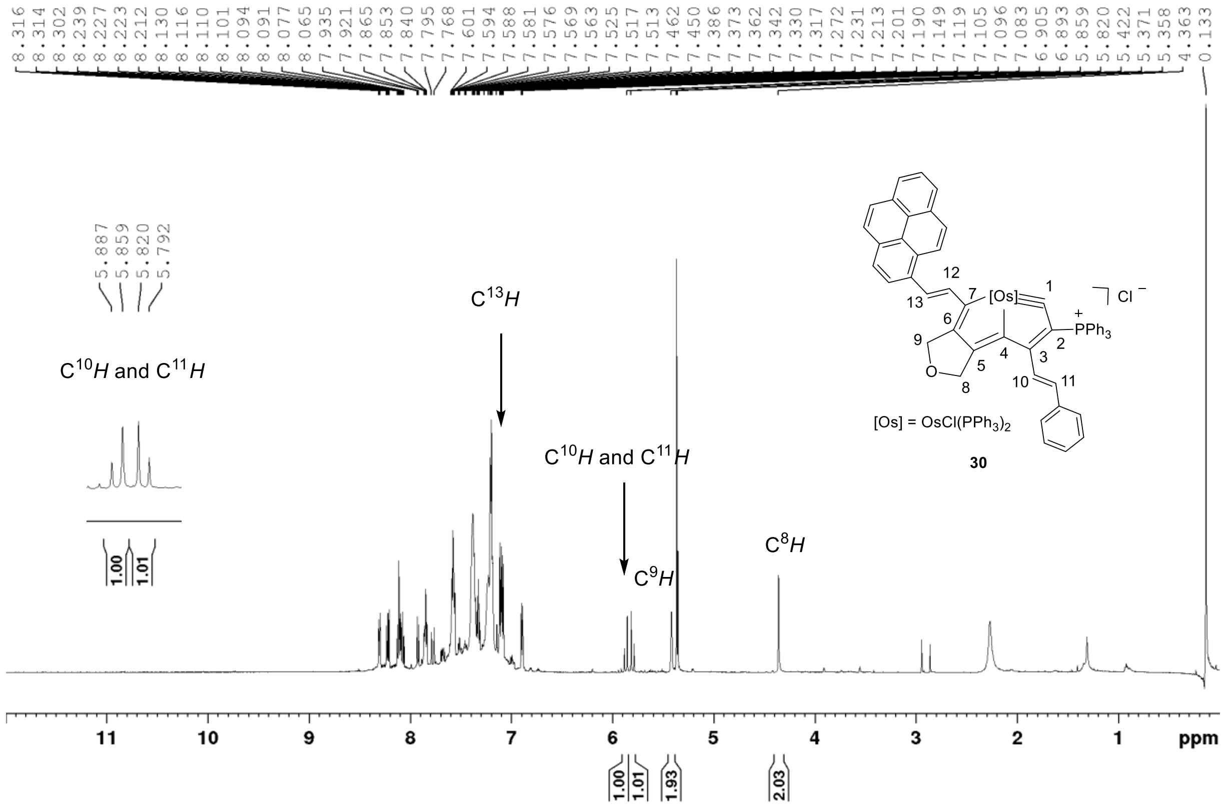

Supplementary Figure 176. The ^{1}H NMR (600.1 MHz, CD_2Cl_2) spectrum for complex **30**.

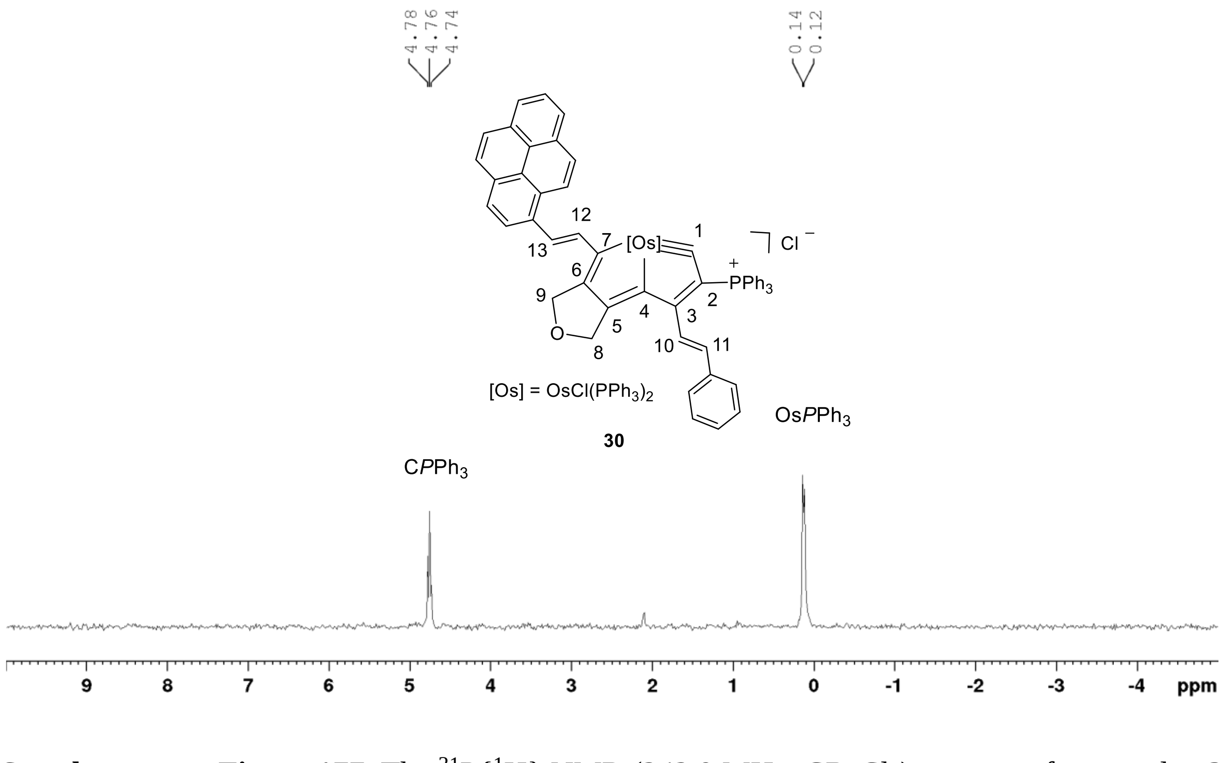

Supplementary Figure 177. The ^{31}P{^{1}H} NMR (242.9 MHz, CD_2Cl_2) spectrum for complex **30**.

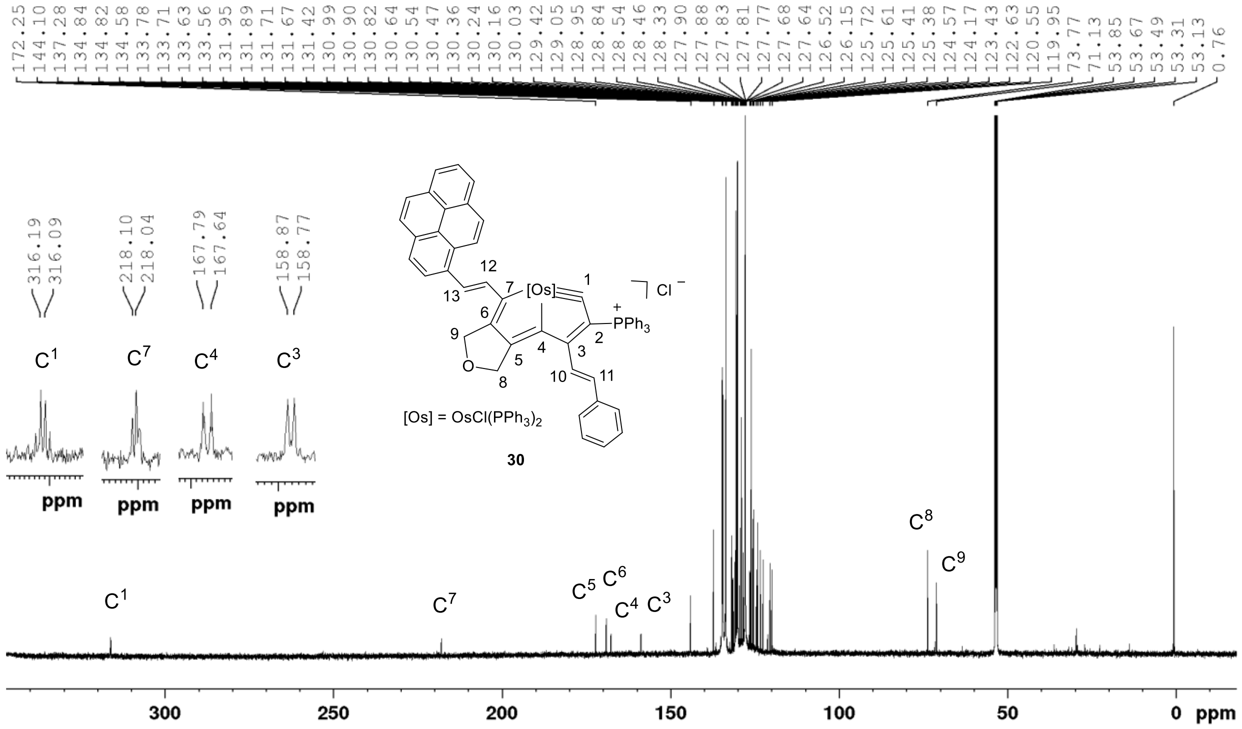

Supplementary Figure 178. The $^{13}C\{^{1}H\}$ NMR (150.9 MHz, CD_2Cl_2) spectrum for complex **30**.

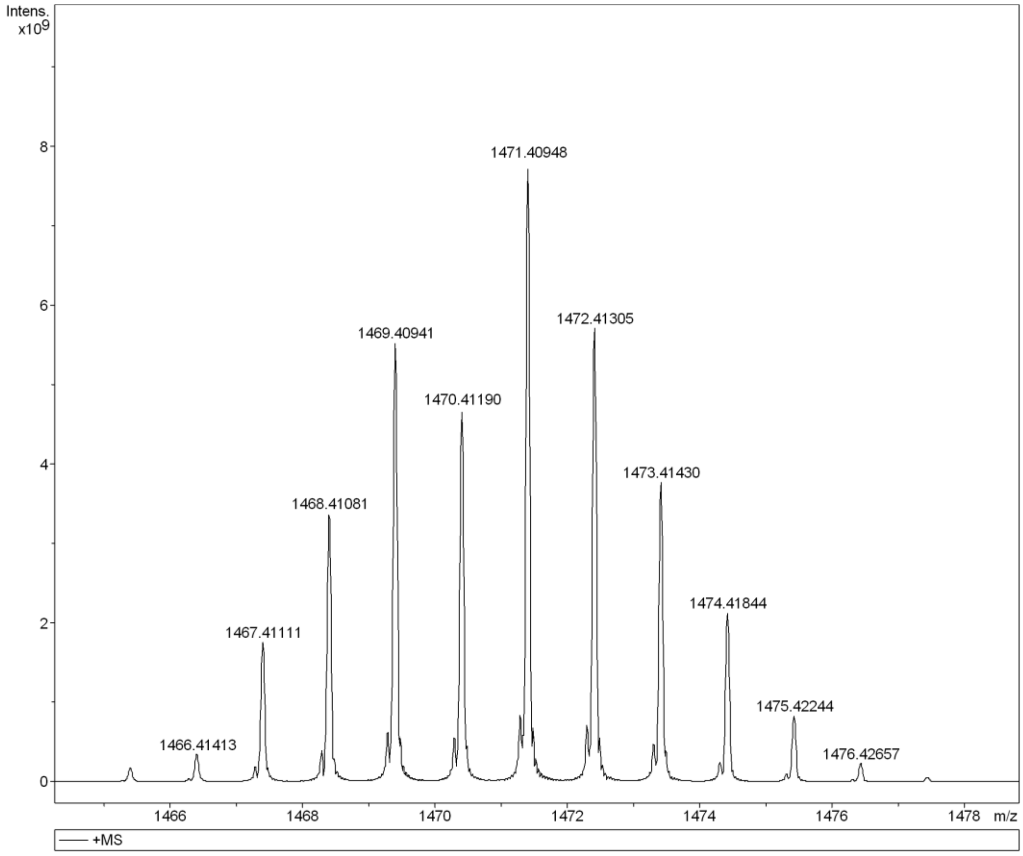

Supplementary Figure 179. Positive-ion ESI-MS spectrum of [**30**]$^{+}$ measured in methanol.

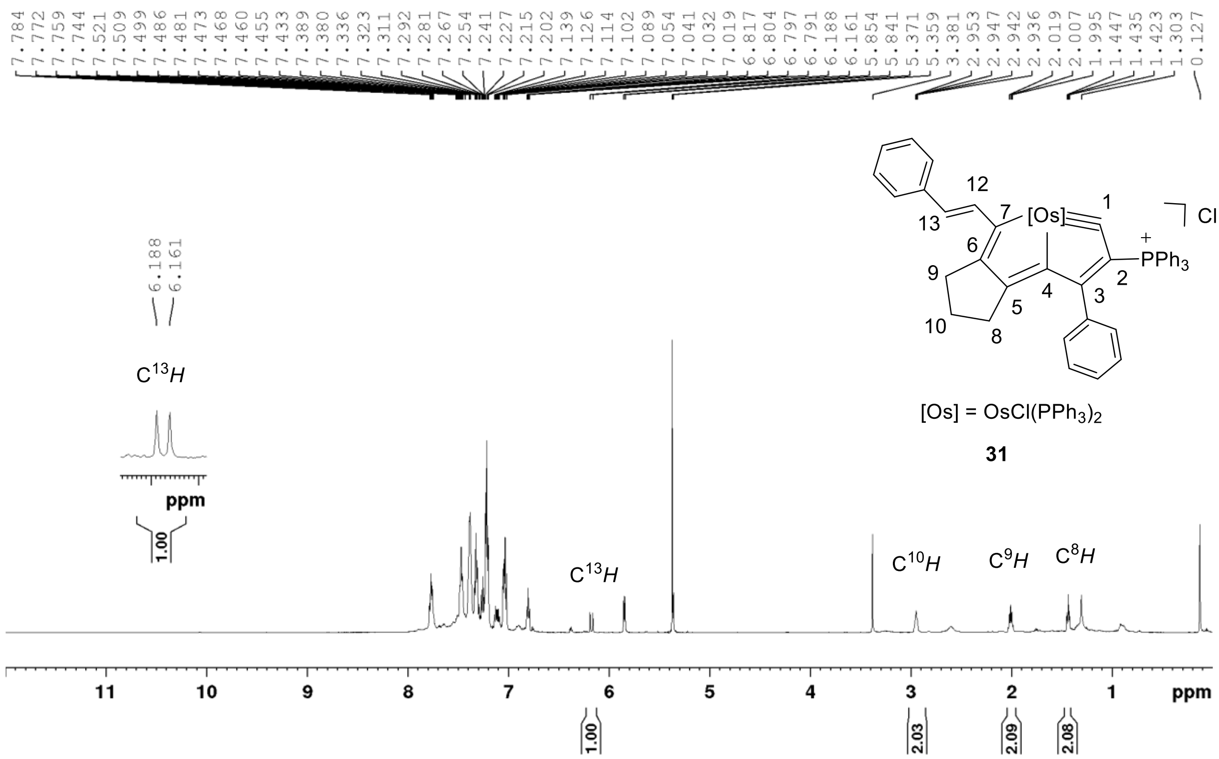

Supplementary Figure 180. The ^{1}H NMR (600.1 MHz, CD_2Cl_2) spectrum for complex **31**.

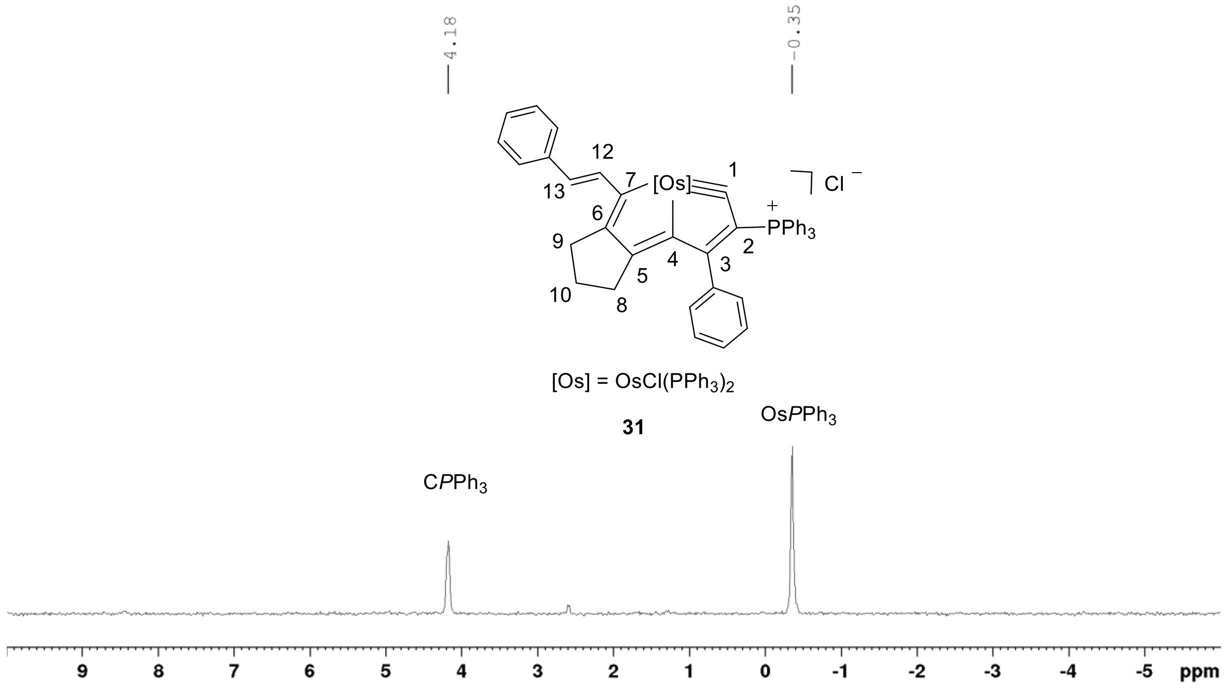

Supplementary Figure 181. The $^{31}P\{^{1}H\}$ NMR (242.9 MHz, CD_2Cl_2) spectrum for complex **31**.

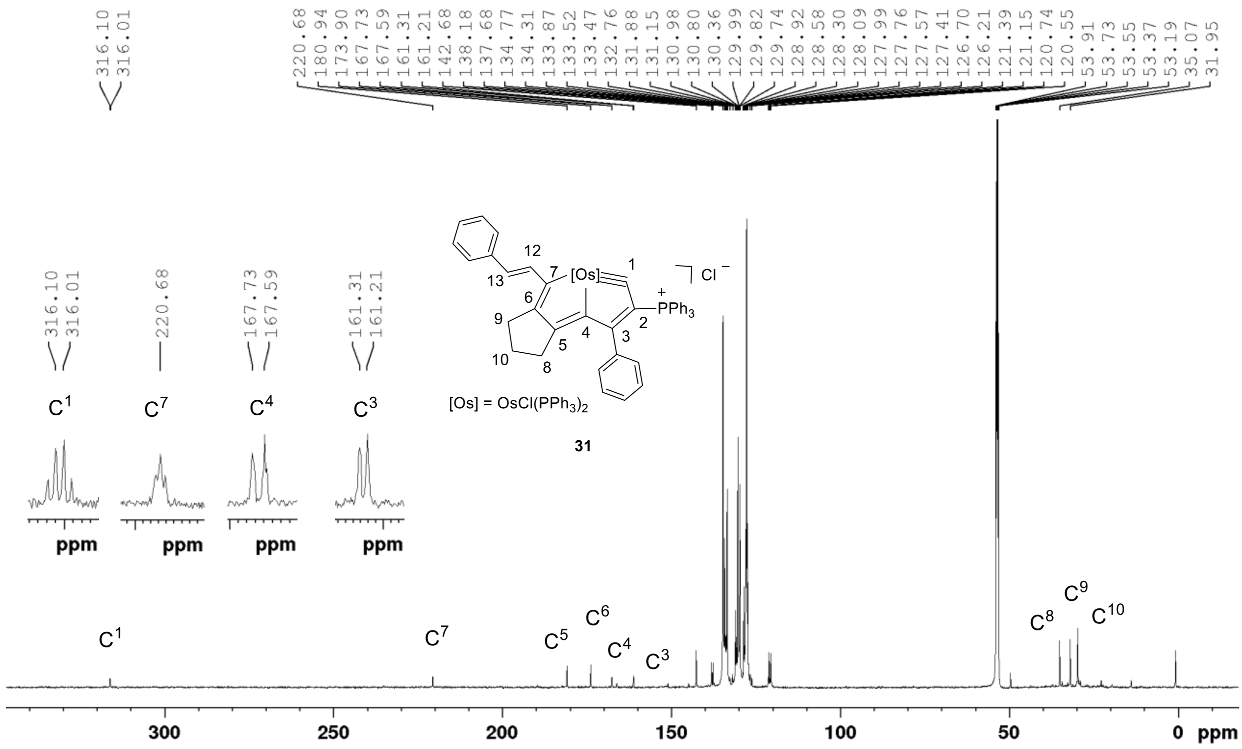

Supplementary Figure 182. The $^{13}C\{^1H\}$ NMR (150.9 MHz, CD_2Cl_2) spectrum for complex **31**.

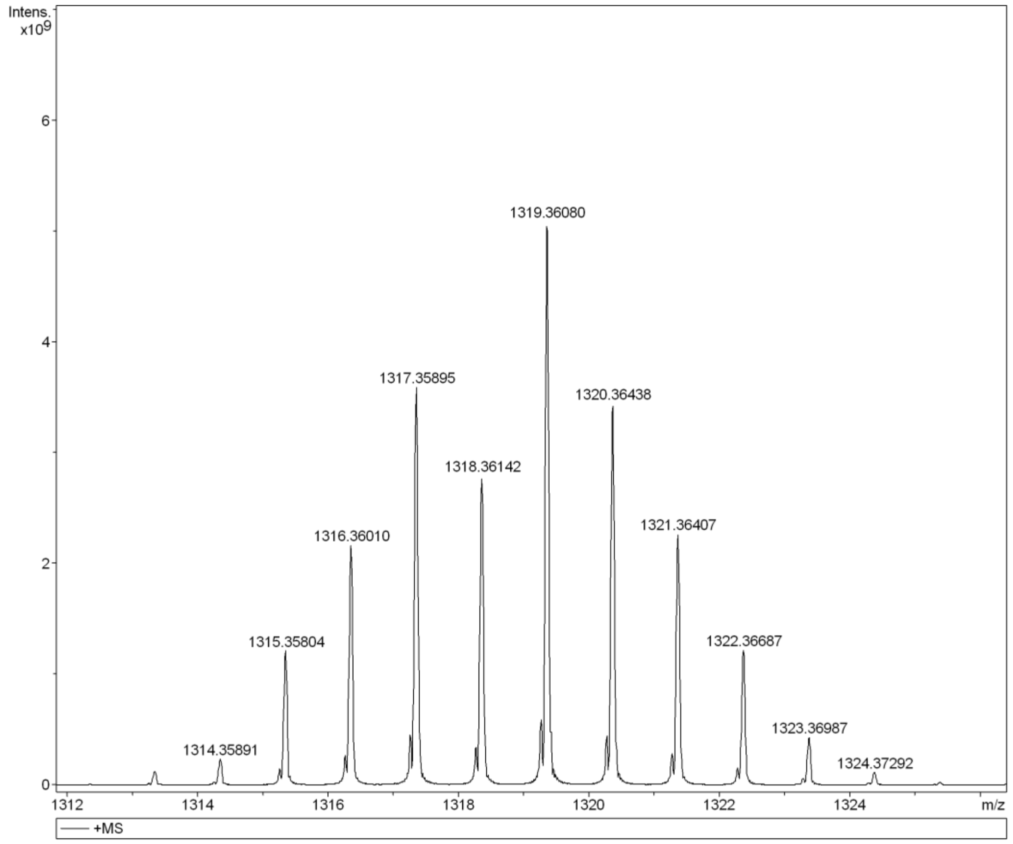

Supplementary Figure 183. Positive-ion ESI-MS spectrum of [**31**]$^+$ measured in methanol.

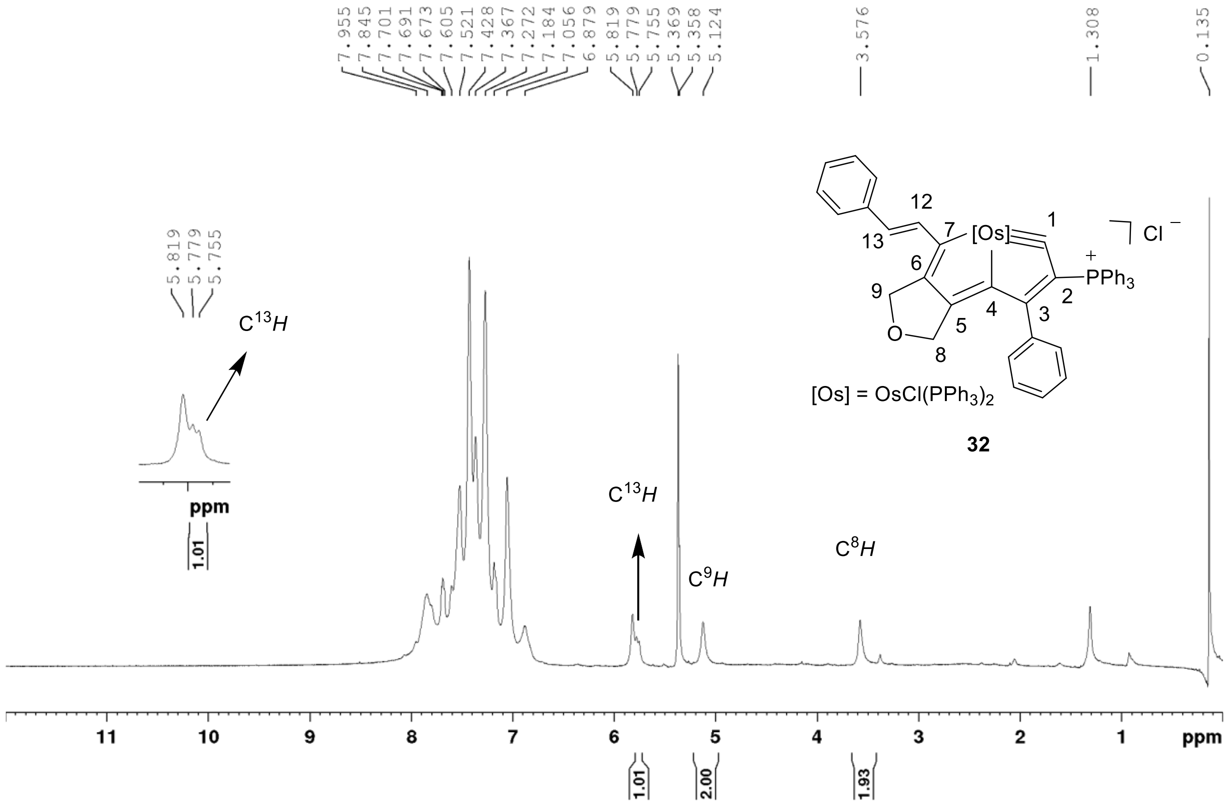

Supplementary Figure 184. The ^{1}H NMR (600.1 MHz, CD_2Cl_2) spectrum for complex **32**.

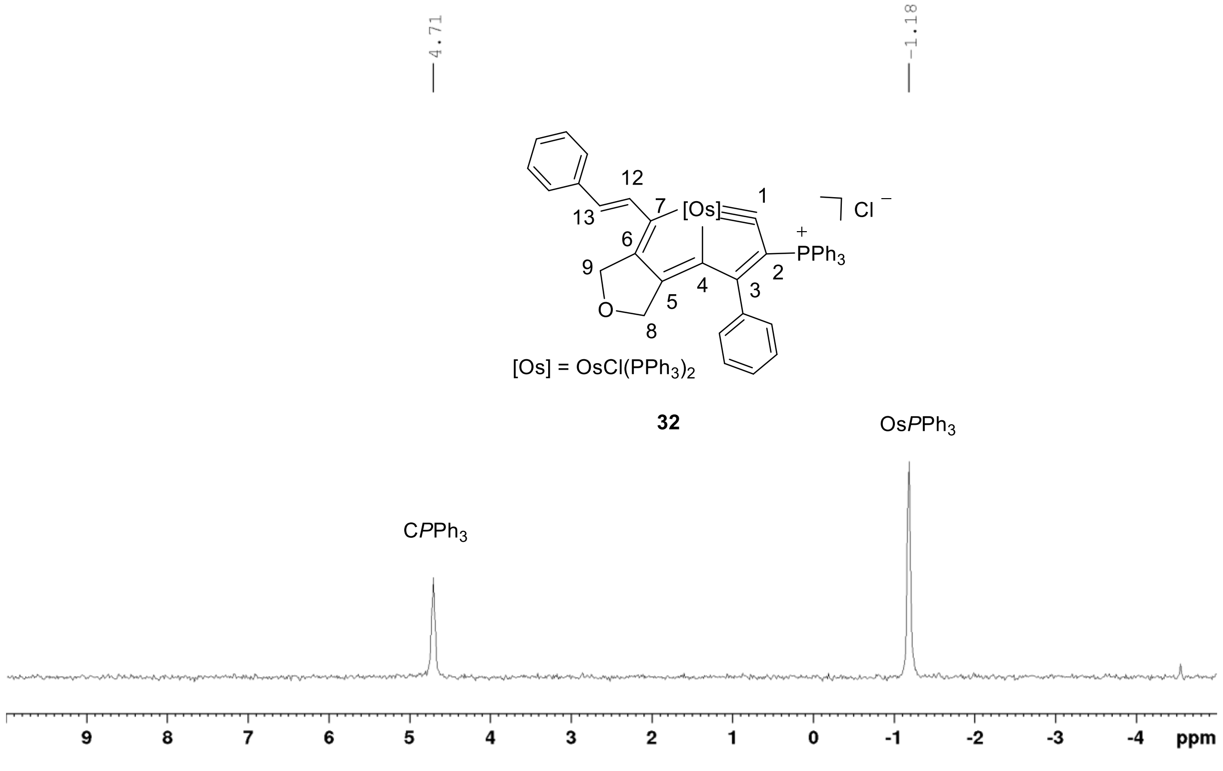

Supplementary Figure 185. The ^{31}P{^{1}H} NMR (242.9 MHz, CD_2Cl_2) spectrum for complex **32**.

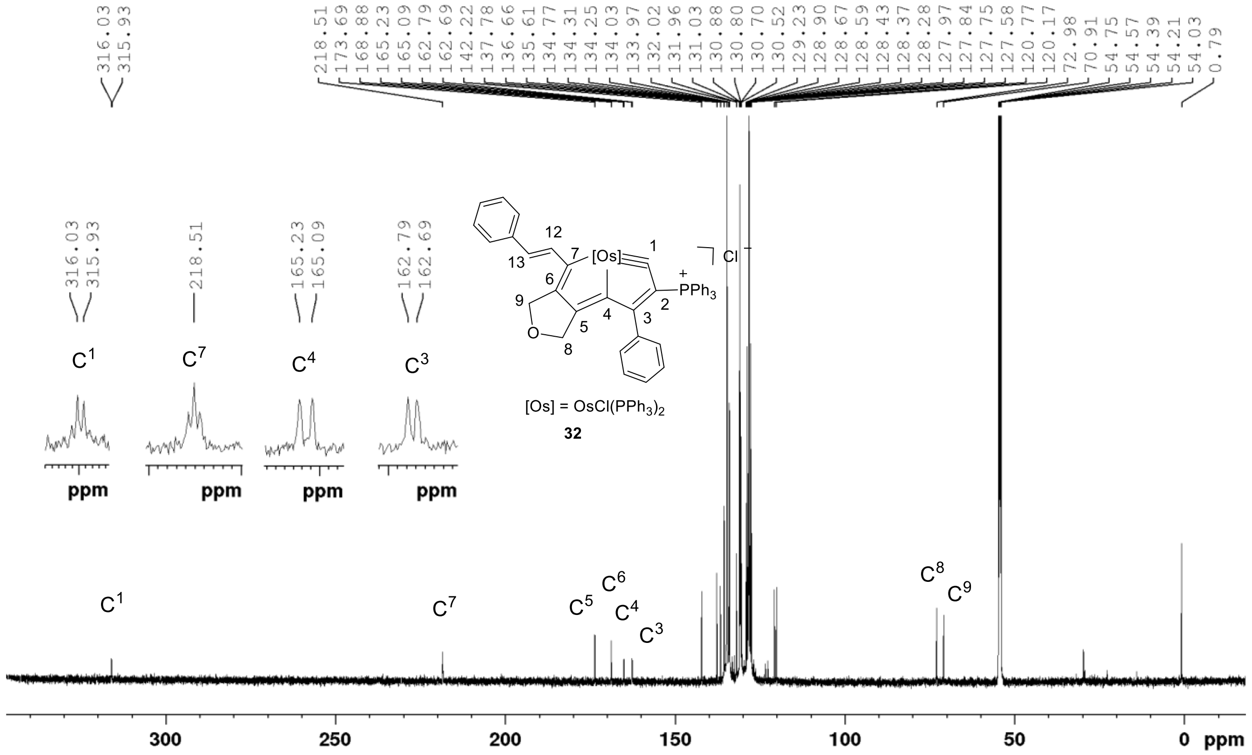

Supplementary Figure 186. The $^{13}C\{^{1}H\}$ NMR (150.9 MHz, CD_2Cl_2) spectrum for complex **32**.

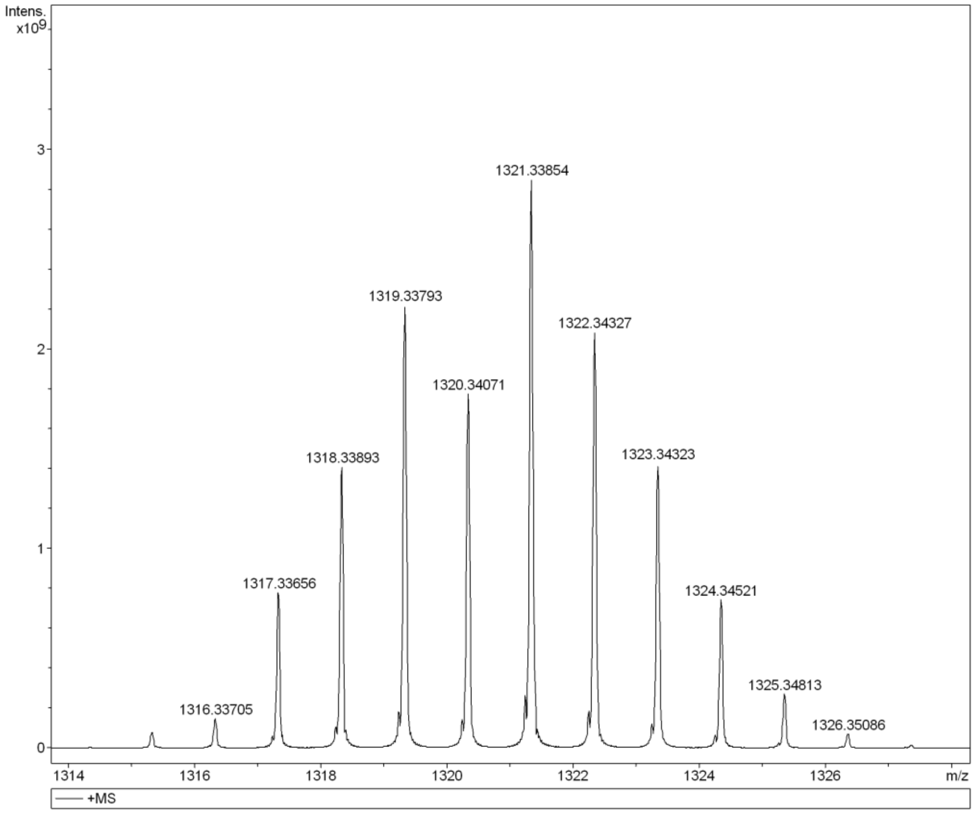

Supplementary Figure 187. Positive-ion ESI-MS spectrum of [**32**]$^{+}$ measured in methanol.

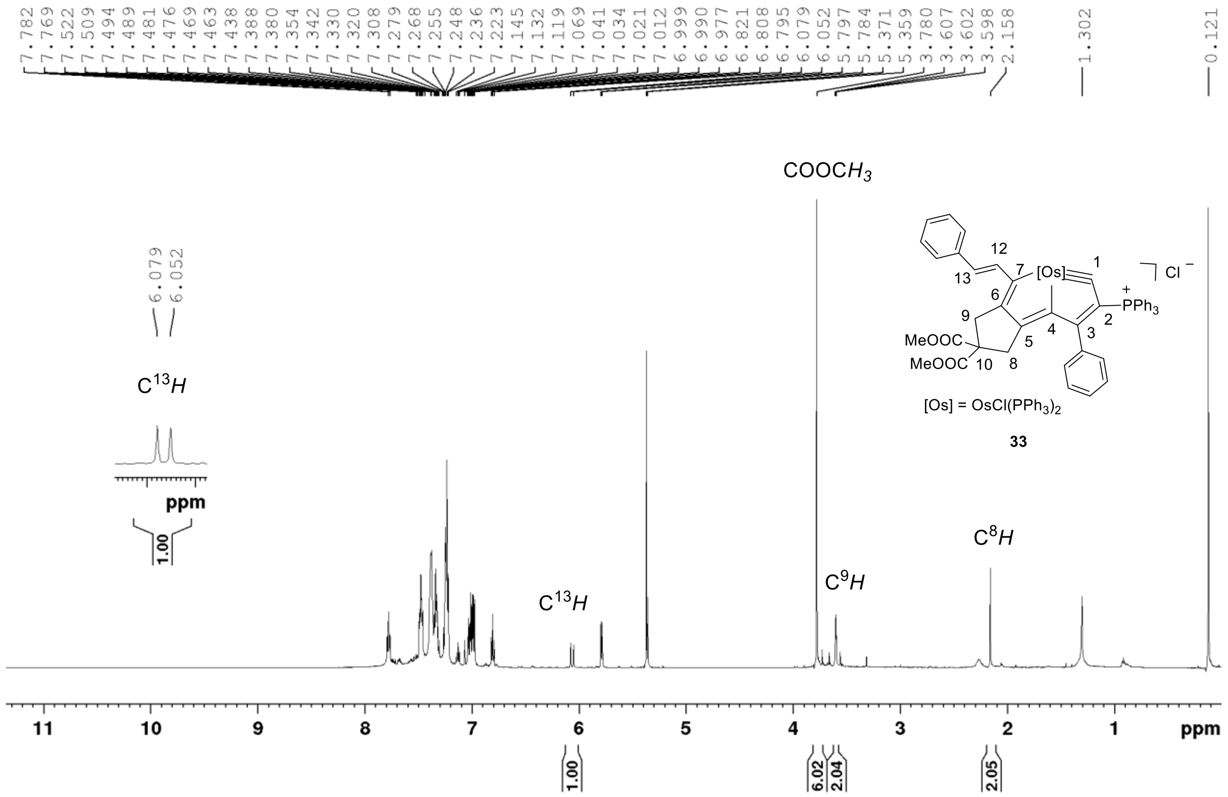

Supplementary Figure 188. The ^{1}H NMR (600.1 MHz, CD_2Cl_2) spectrum for complex **33**.

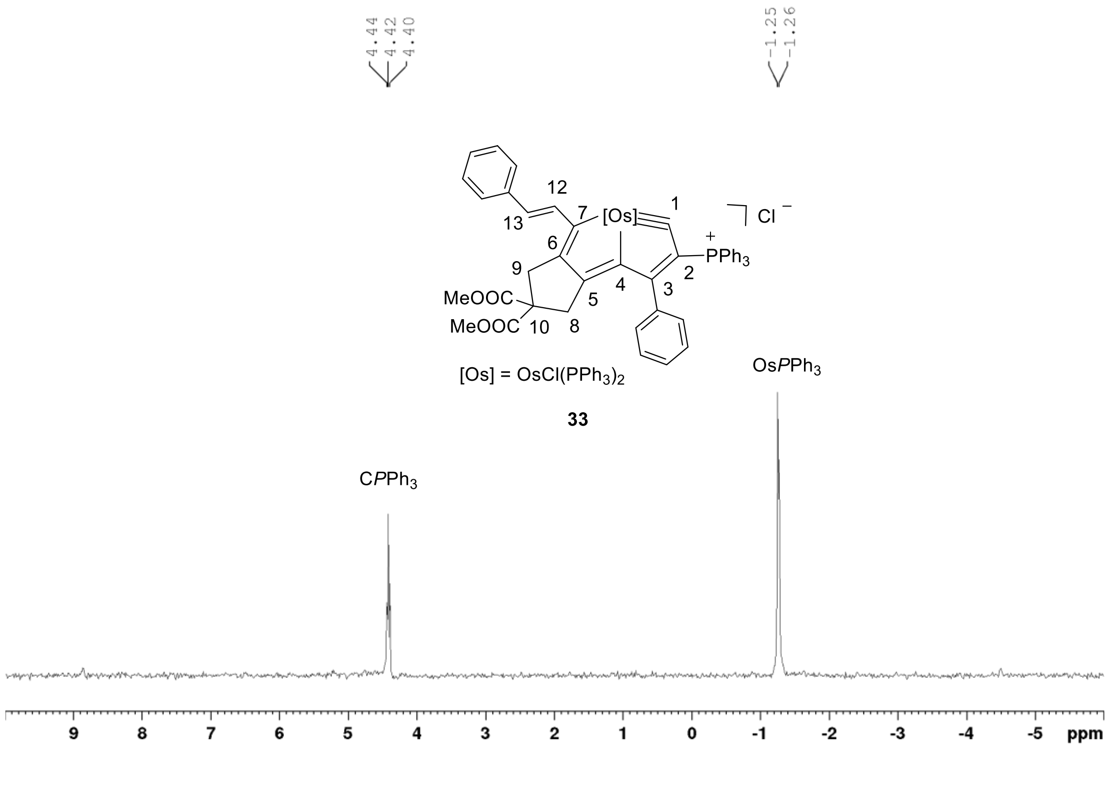

Supplementary Figure 189. The ^{31}P{^{1}H} NMR (242.9 MHz, CD_2Cl_2) spectrum for complex **33**.

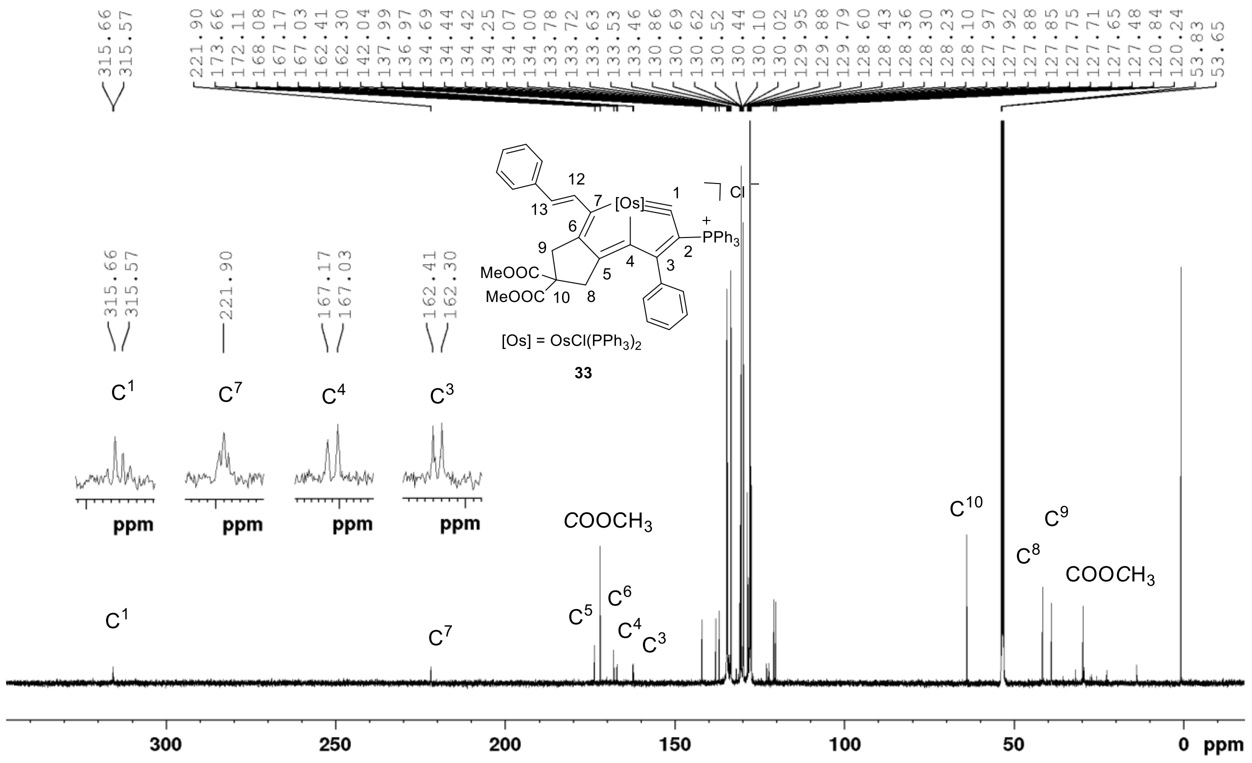

Supplementary Figure 190. The $^{13}C\{^1H\}$ NMR (150.9 MHz, CD_2Cl_2) spectrum for complex **33**.

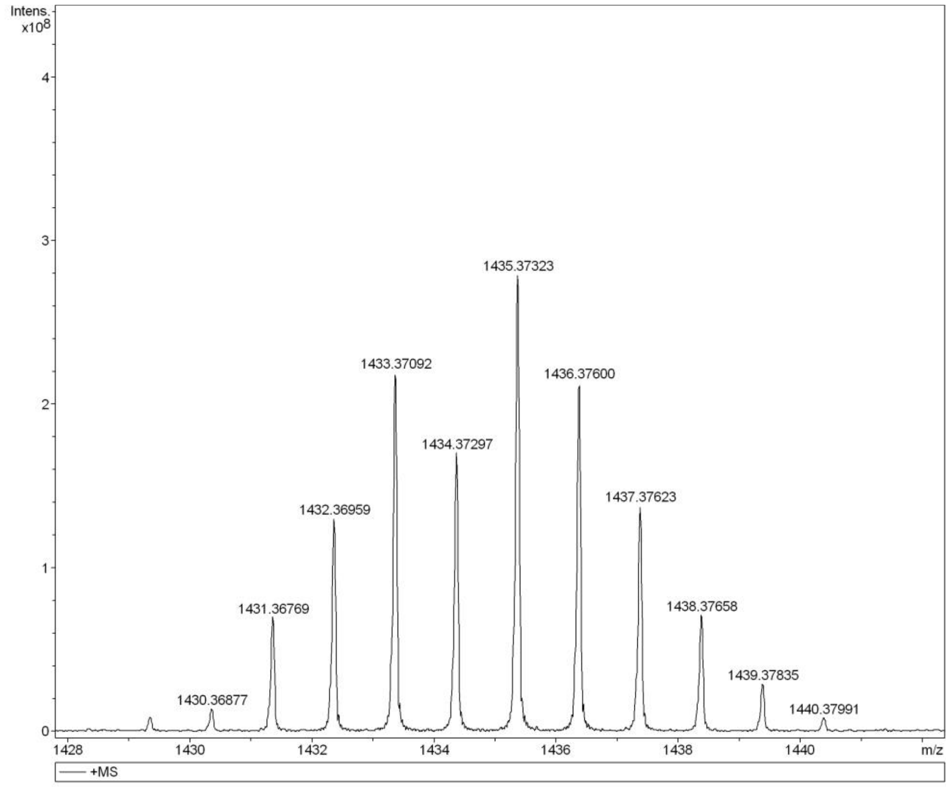

Supplementary Figure 191. Positive-ion ESI-MS spectrum of $[\mathbf{33}]^+$ measured in methanol.

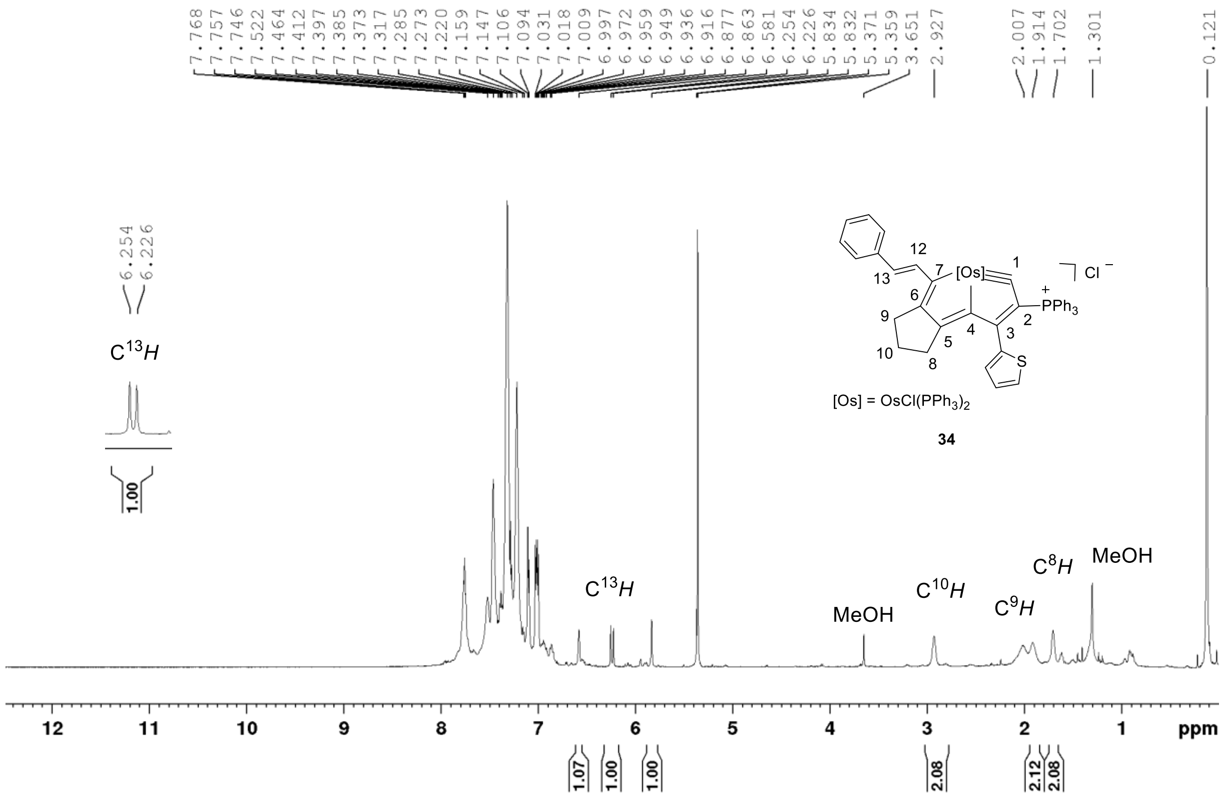

Supplementary Figure 192. The ^{1}H NMR (600.1 MHz, CD_2Cl_2) spectrum for complex **34**.

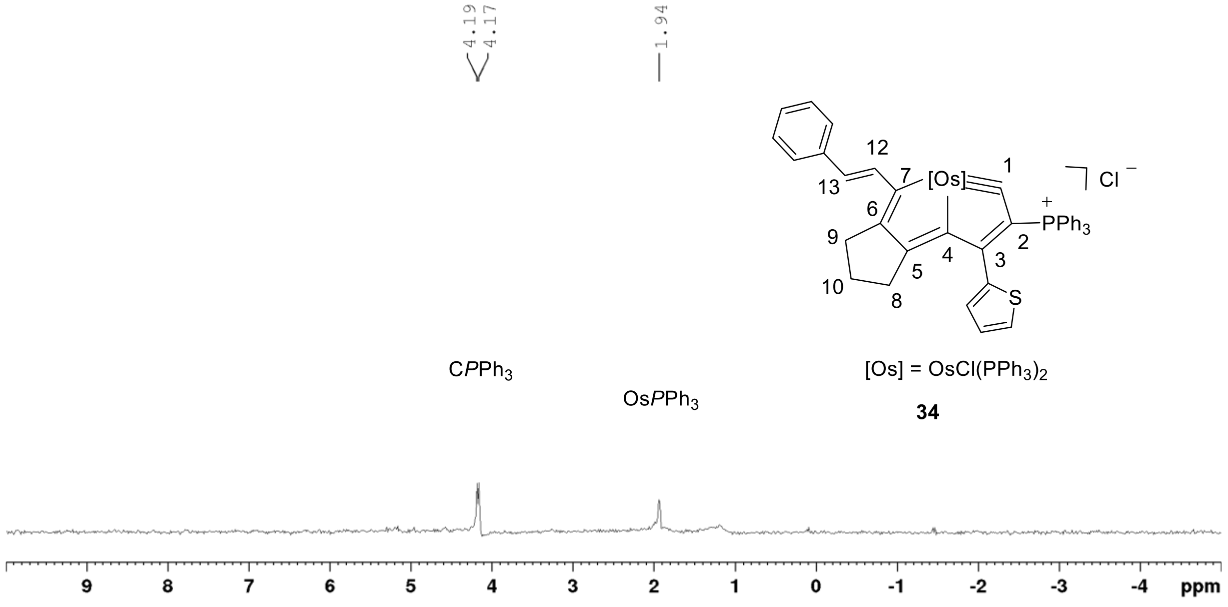

Supplementary Figure 193. The ^{31}P{^{1}H} NMR (242.9 MHz, CD_2Cl_2) spectrum for complex **34**.

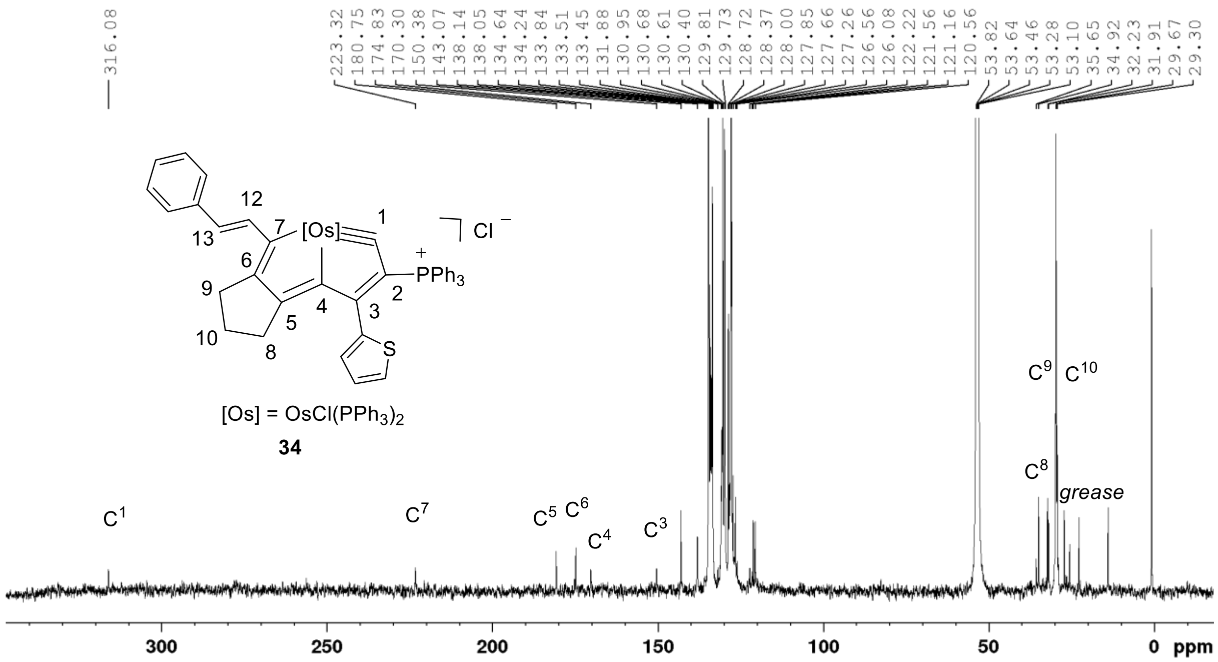

Supplementary Figure 194. The $^{13}C\{^1H\}$ NMR (150.9 MHz, CD_2Cl_2) spectrum for complex **34**.

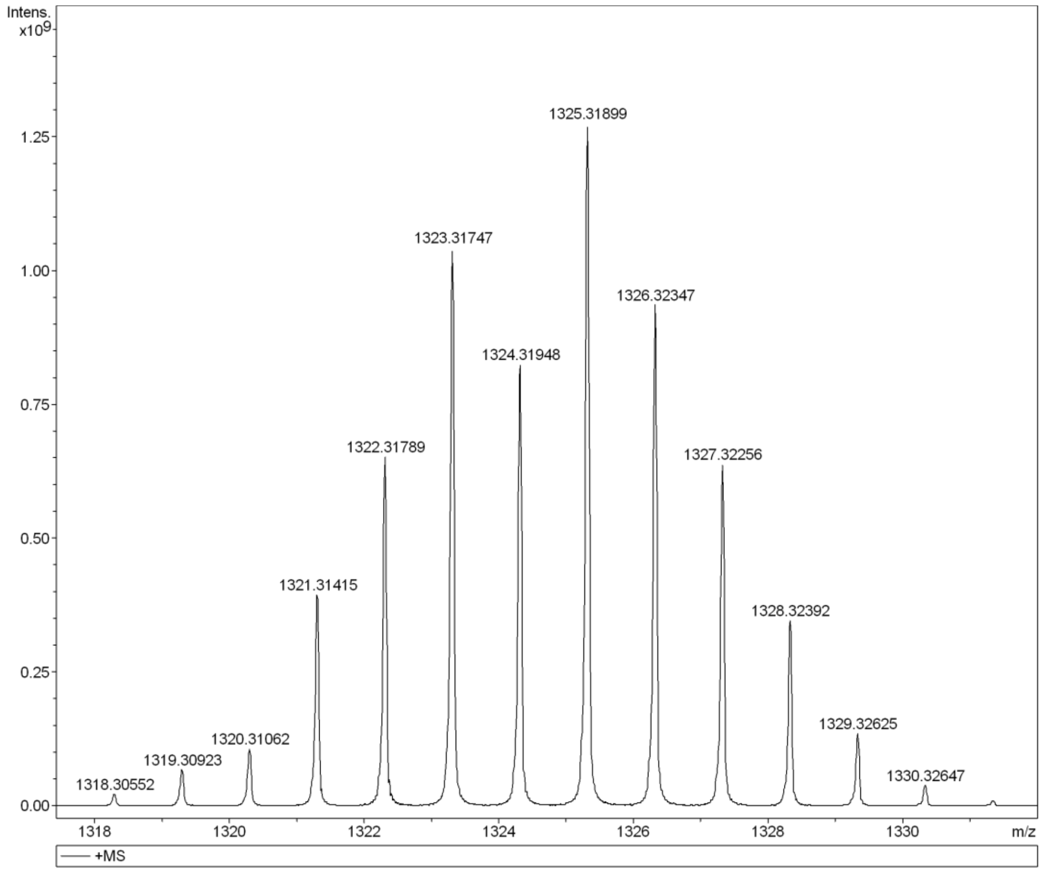

Supplementary Figure 195. Positive-ion ESI-MS spectrum of $[\mathbf{34}]^+$ measured in methanol.

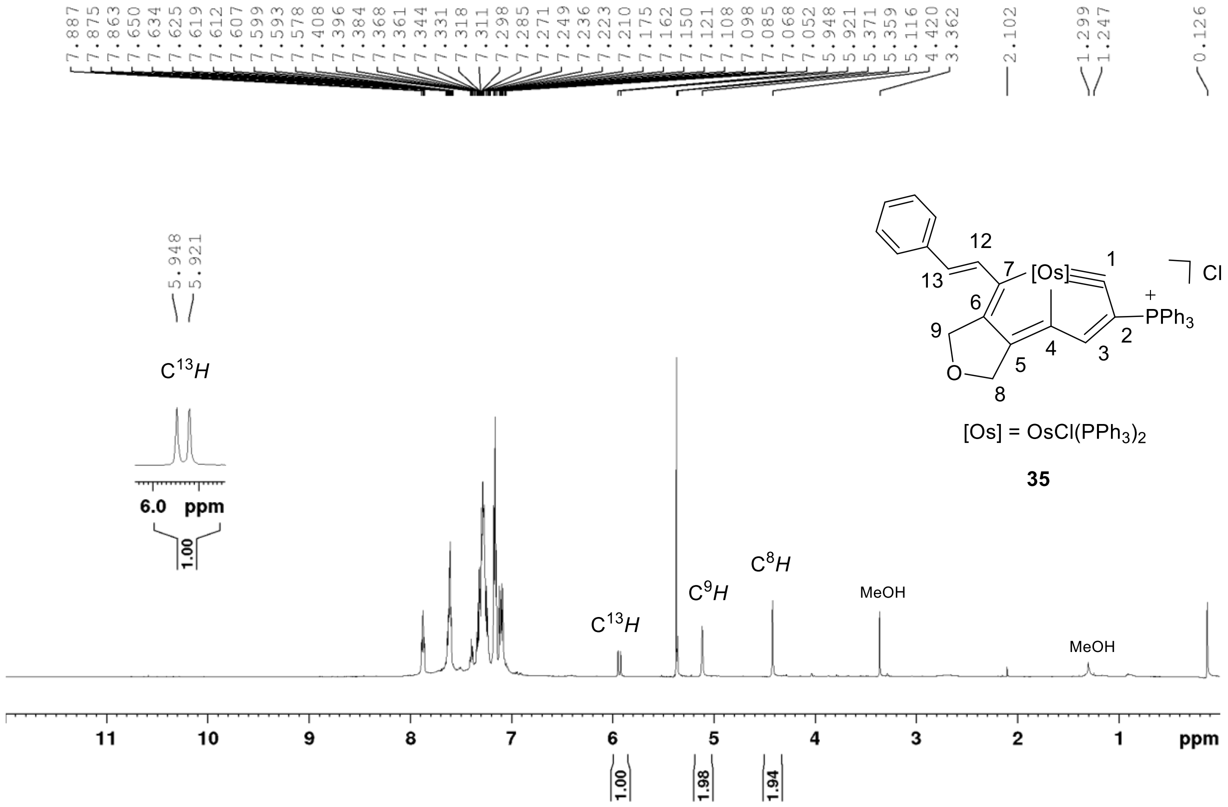

Supplementary Figure 196. The ^{1}H NMR (600.1 MHz, CD_2Cl_2) spectrum for complex **35**.

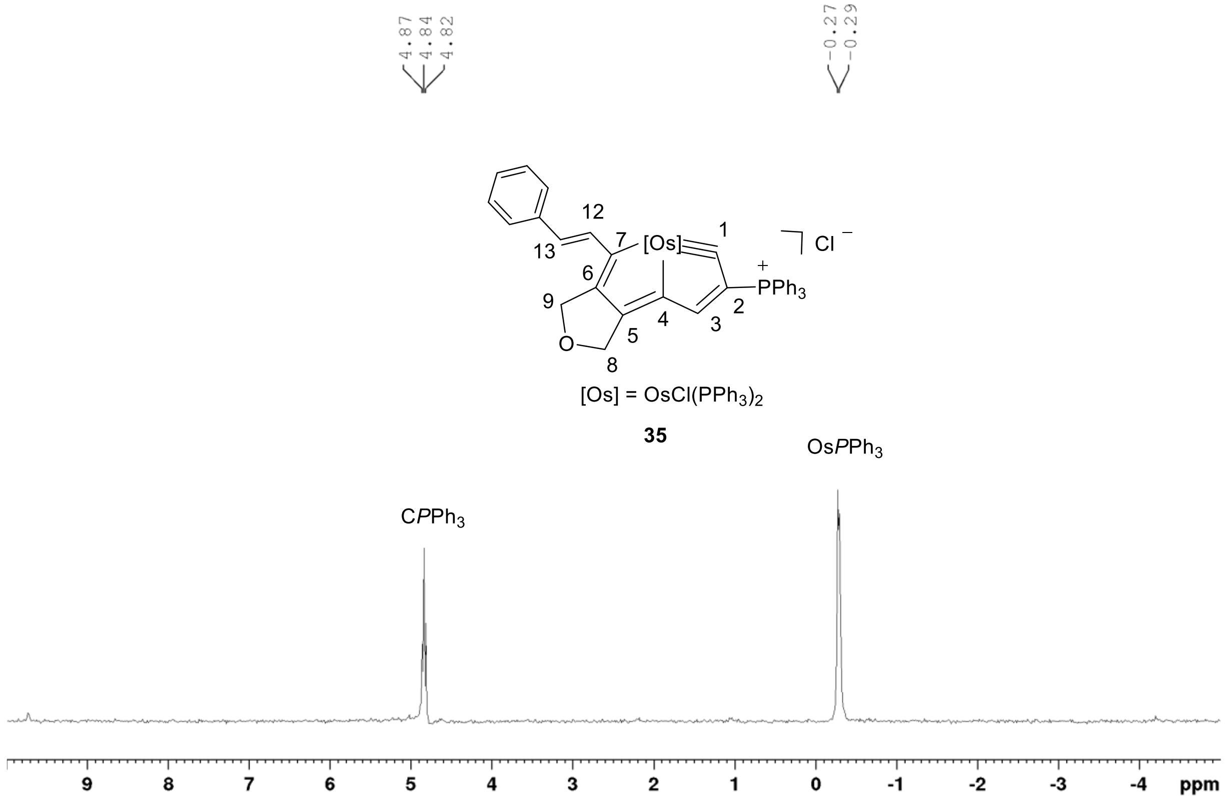

Supplementary Figure 197. The ^{31}P{^{1}H} NMR (242.9 MHz, CD_2Cl_2) spectrum for complex **35**.

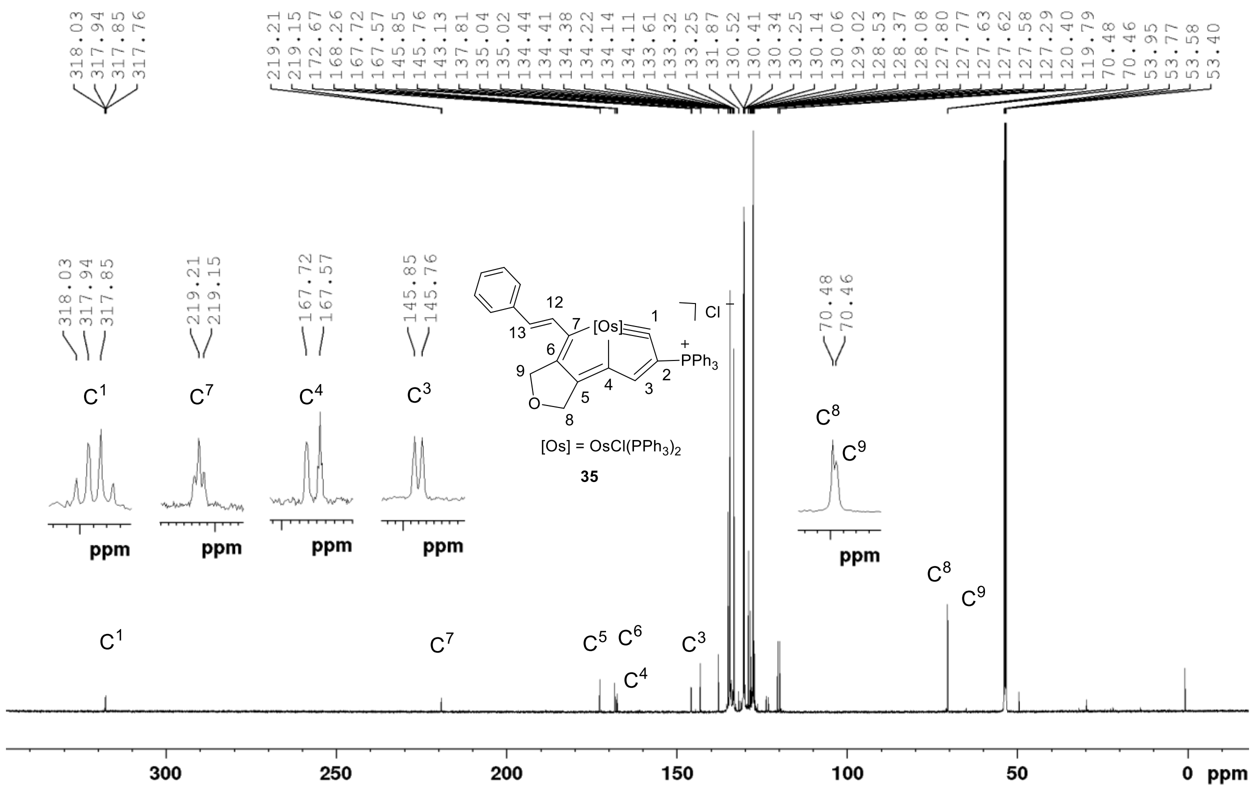

Supplementary Figure 198. The $^{13}C\{^{1}H\}$ NMR (150.9 MHz, CD_2Cl_2) spectrum for complex **35**.

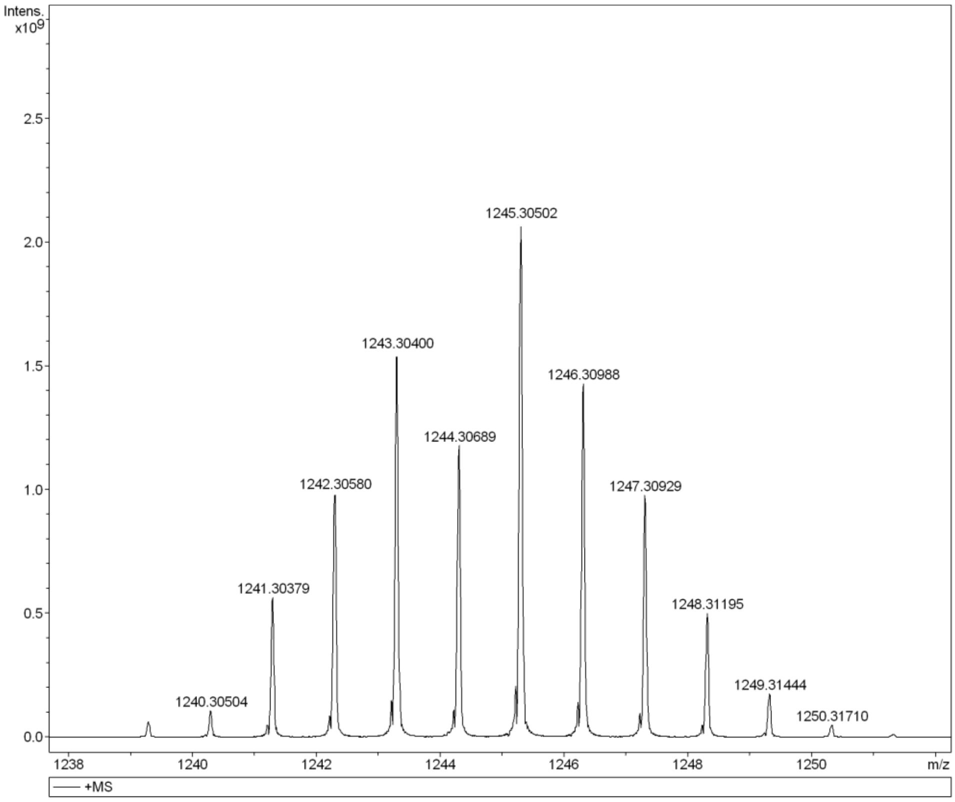

Supplementary Figure 199. Positive-ion ESI-MS spectrum of [**35**]$^{+}$ measured in methanol.

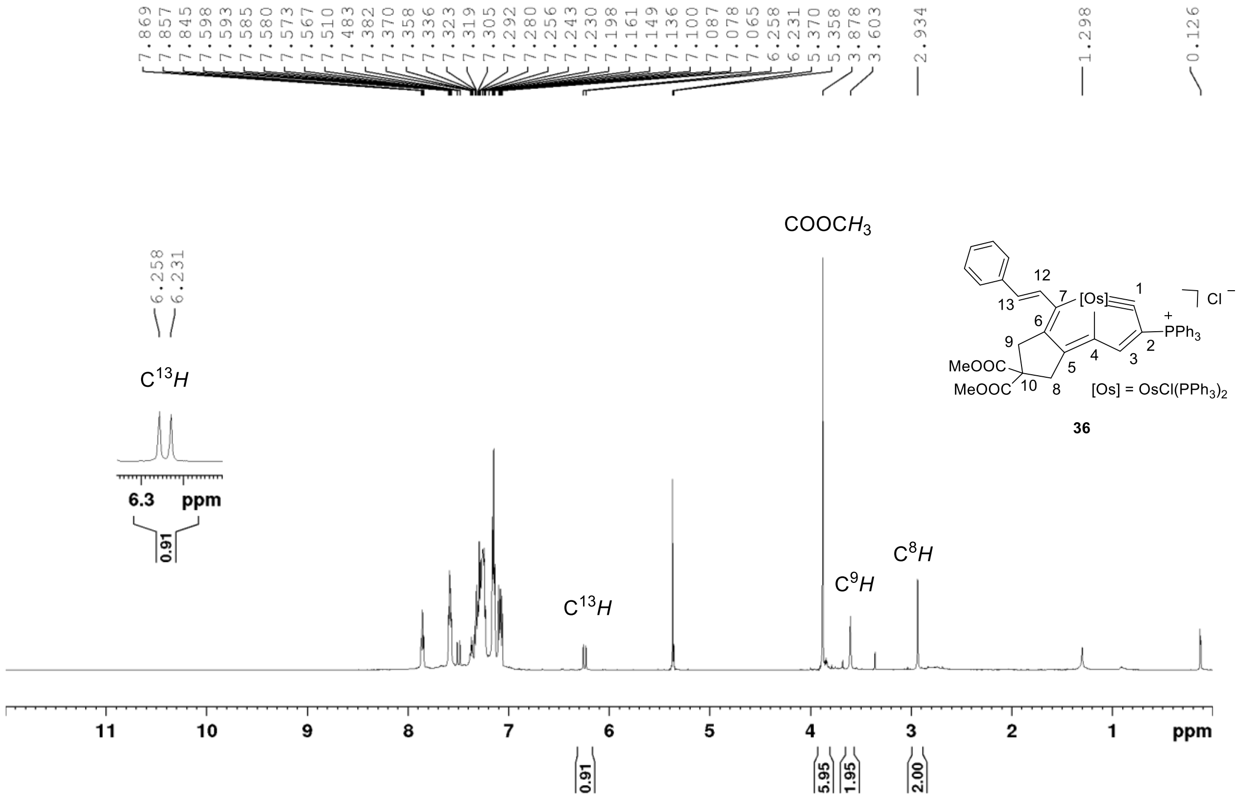

Supplementary Figure 200. The ^{1}H NMR (600.1 MHz, CD_2Cl_2) spectrum for complex **36**.

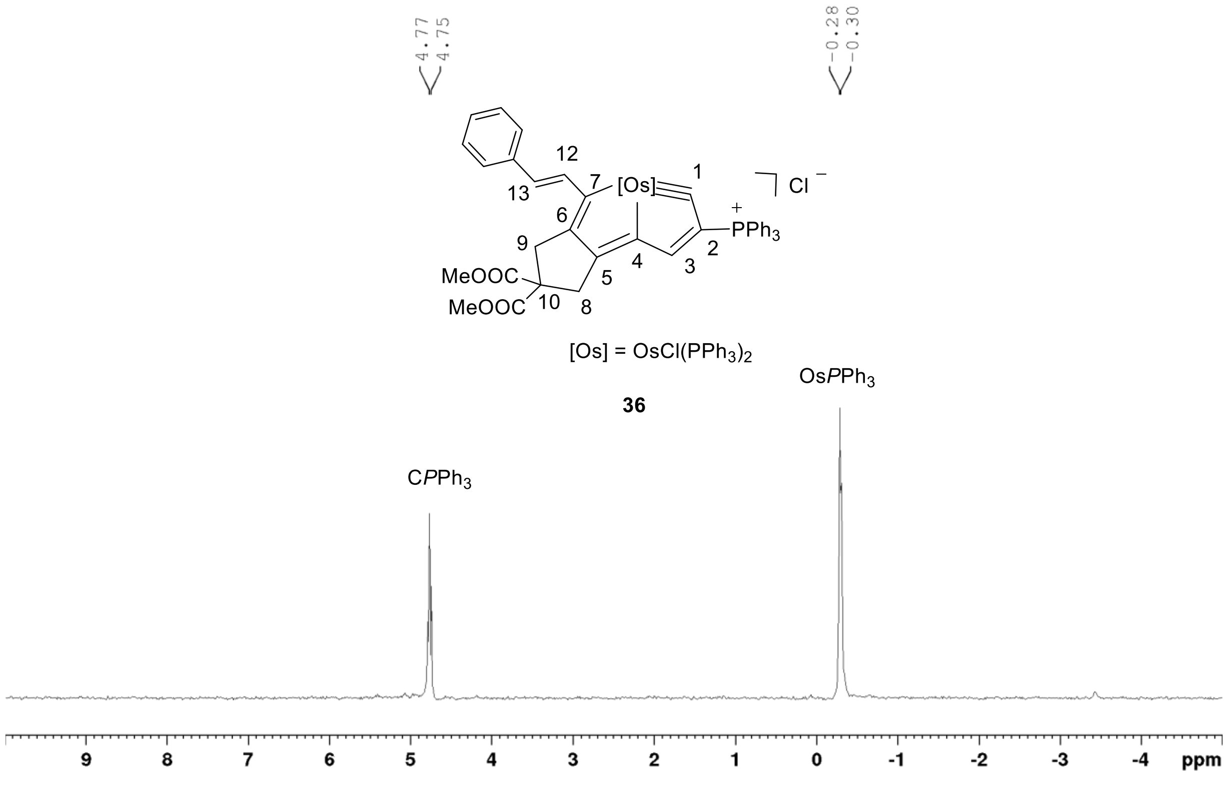

Supplementary Figure 201. The ^{31}P{^{1}H} NMR (242.9 MHz, CD_2Cl_2) spectrum for complex **36**.

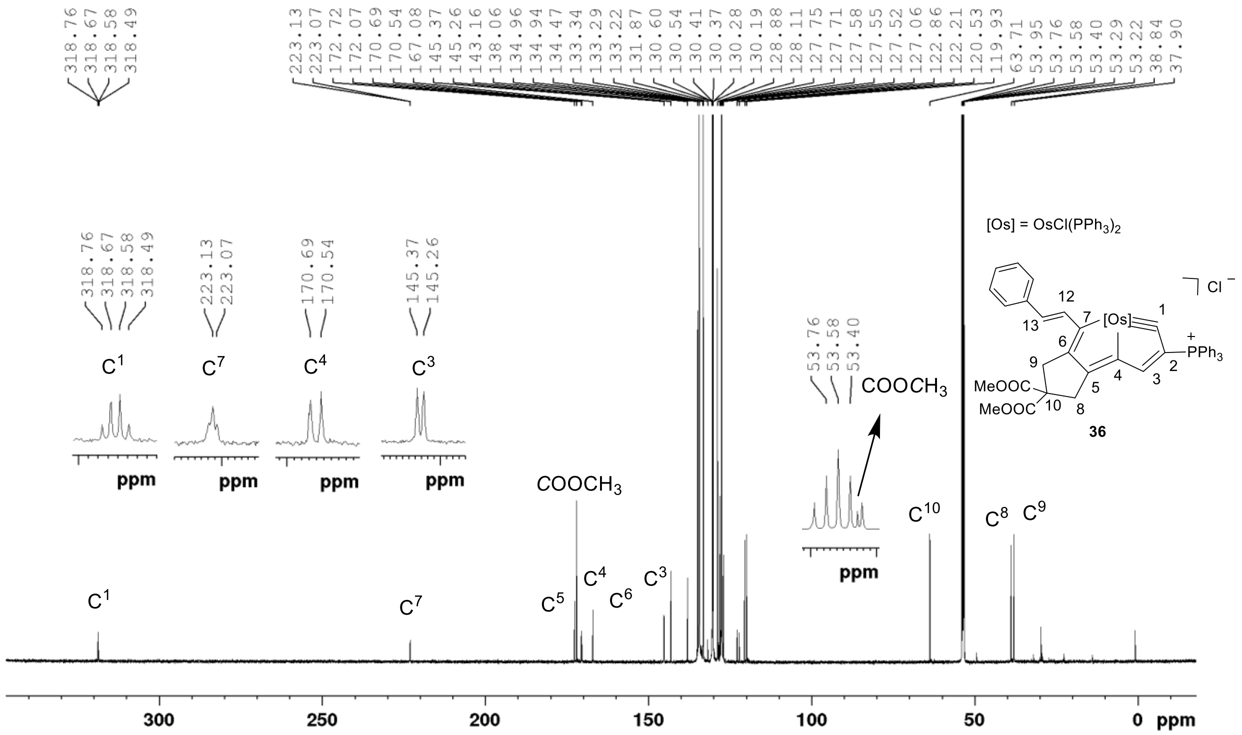

Supplementary Figure 202. The $^{13}C\{^1H\}$ NMR (150.9 MHz, CD_2Cl_2) spectrum for complex **36**.

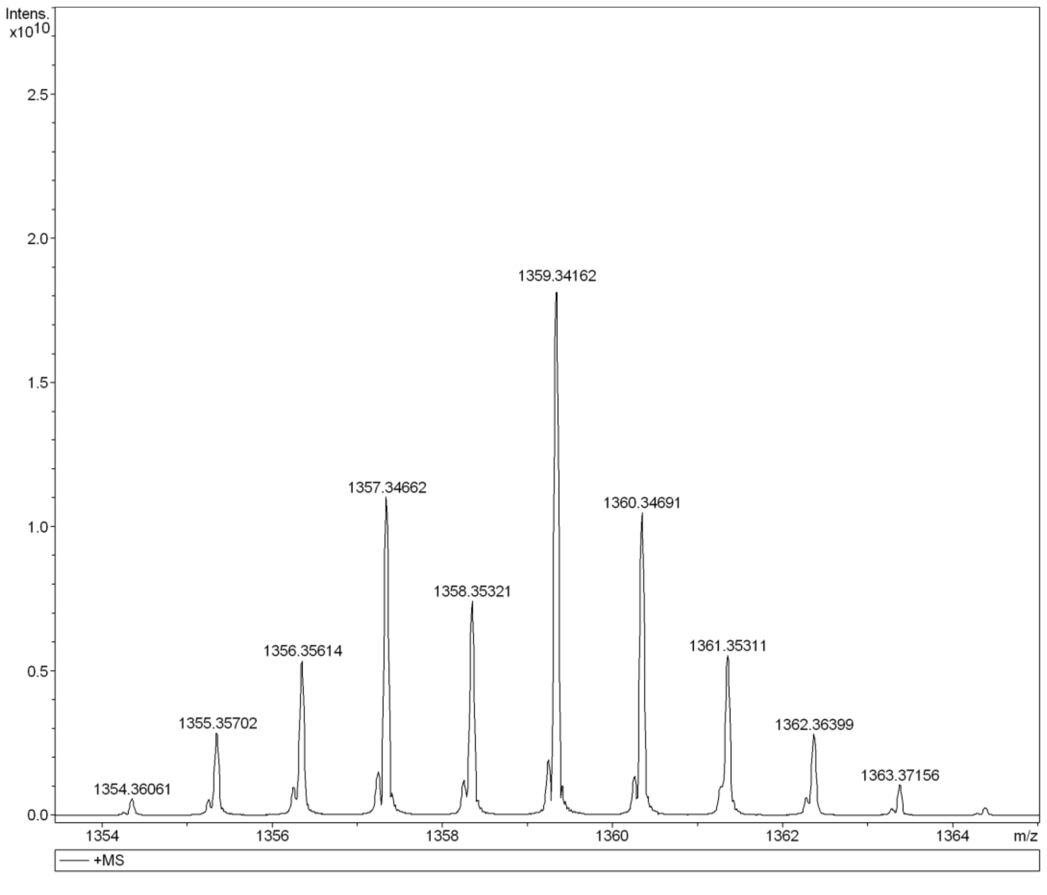

Supplementary Figure 203. Positive-ion ESI-MS spectrum of [**36**]$^+$ measured in methanol.

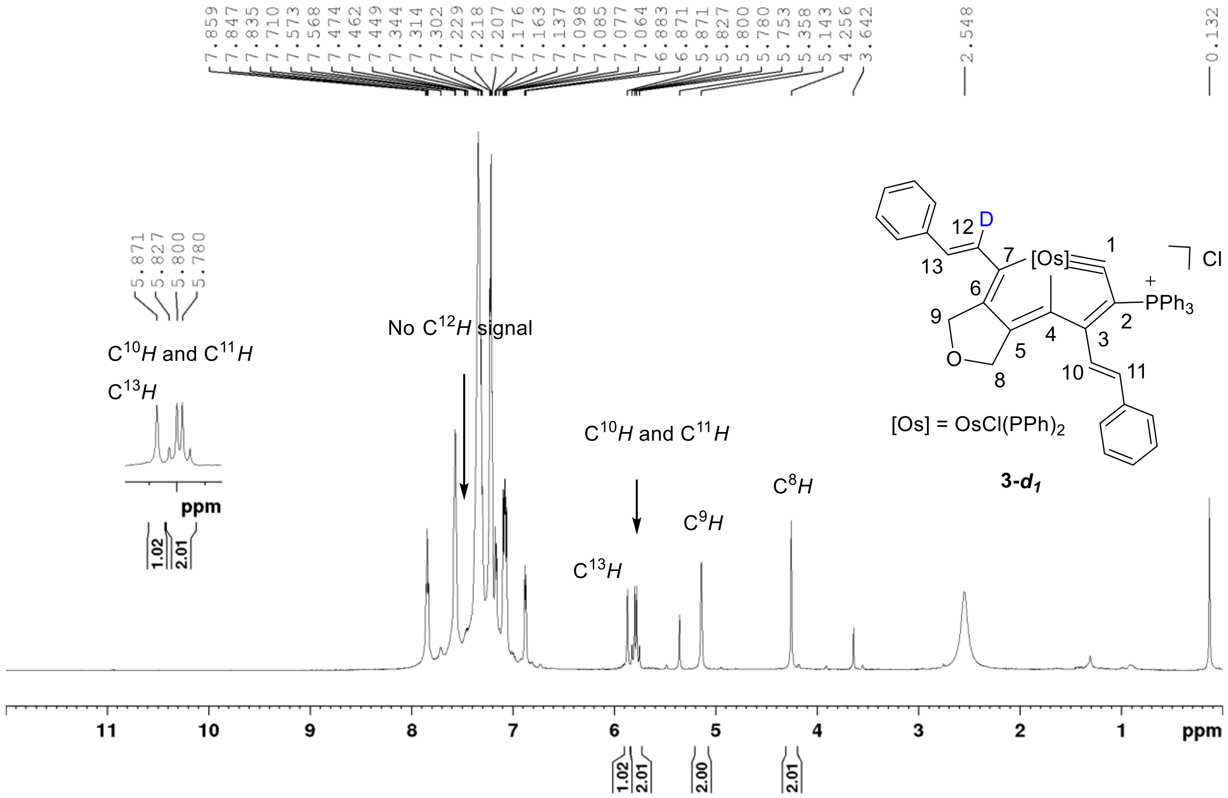

Supplementary Figure 204. The ^{1}H NMR (600.1 MHz, CD_2Cl_2) spectrum for complex **3-*d₁***.

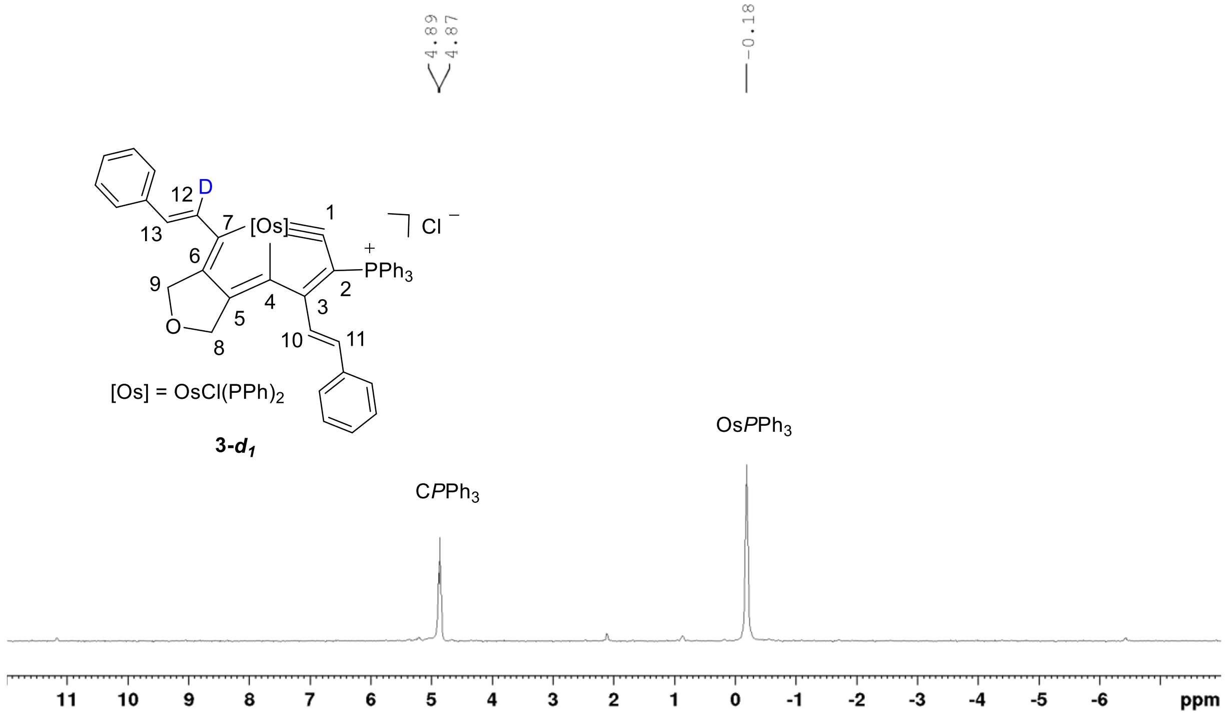

Supplementary Figure 205. The $^{31}P\{^{1}H\}$ NMR (242.9 MHz, CD_2Cl_2) spectrum for complex **3-d_1**.

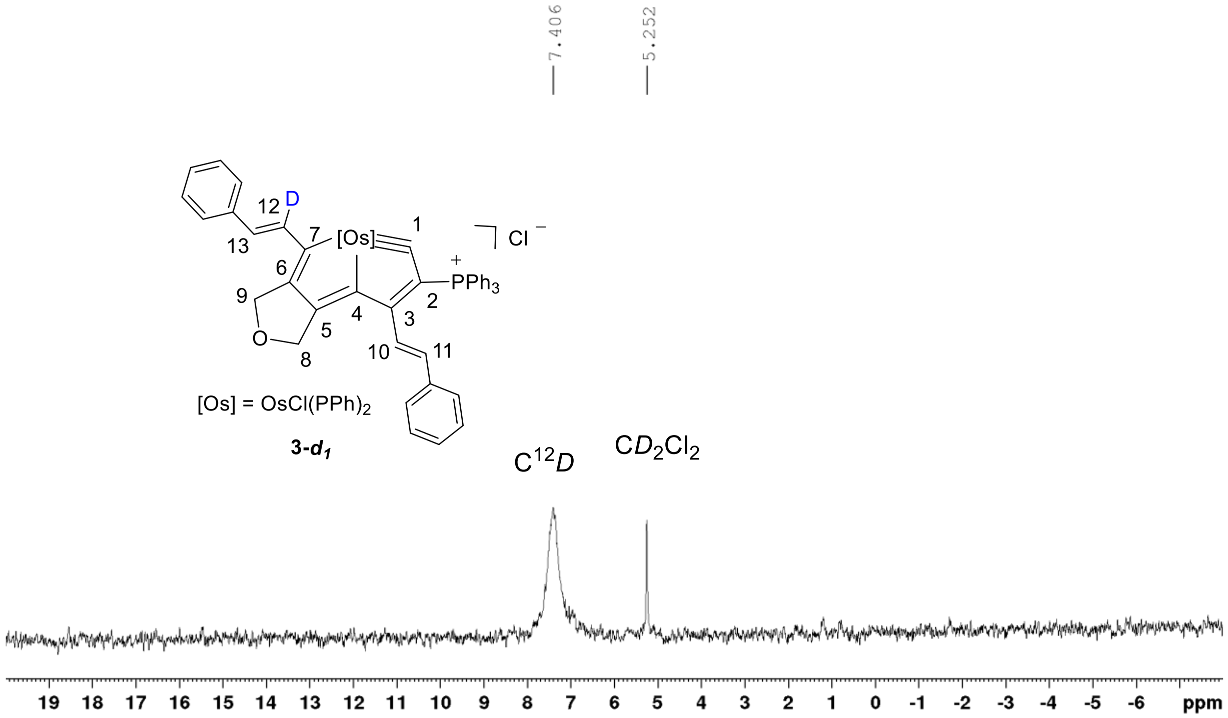

Supplementary Figure 206. The ^{2}D NMR (92.1 MHz, CH_2Cl_2) spectrum for complex **3-*d*$_1$**.

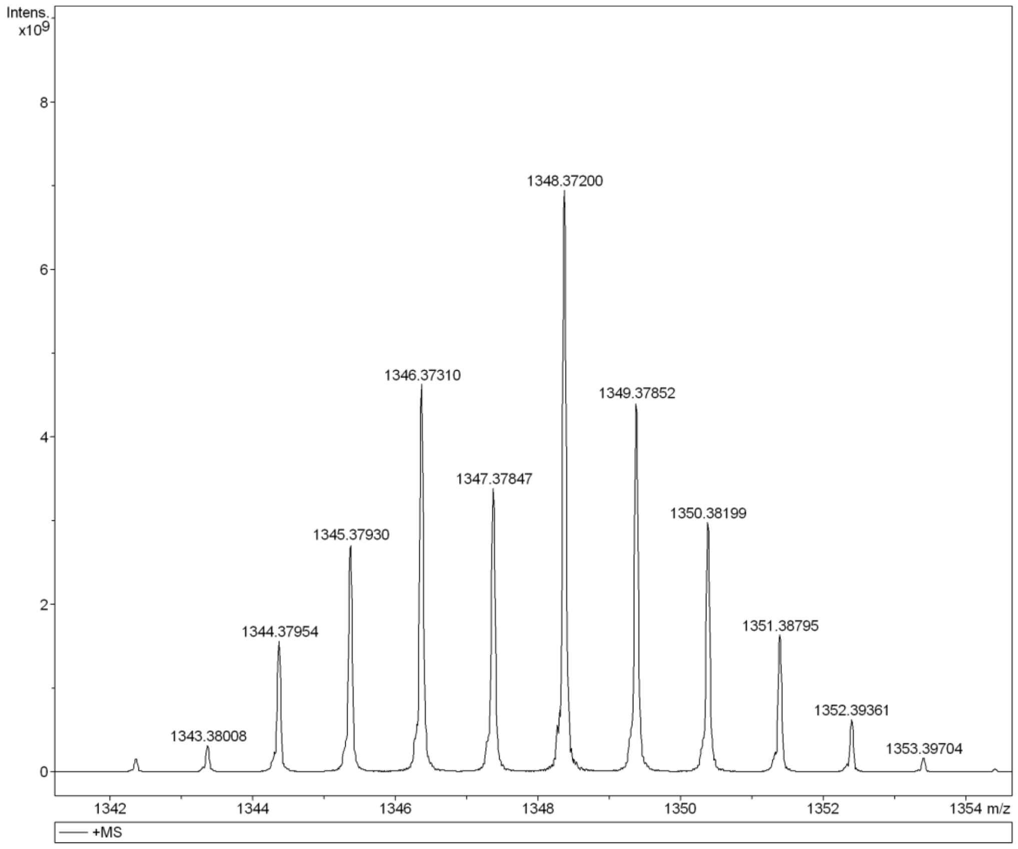

Supplementary Figure 207. Positive-ion ESI-MS spectrum of [**3-*d*$_1$**]$^+$ measured in methanol.

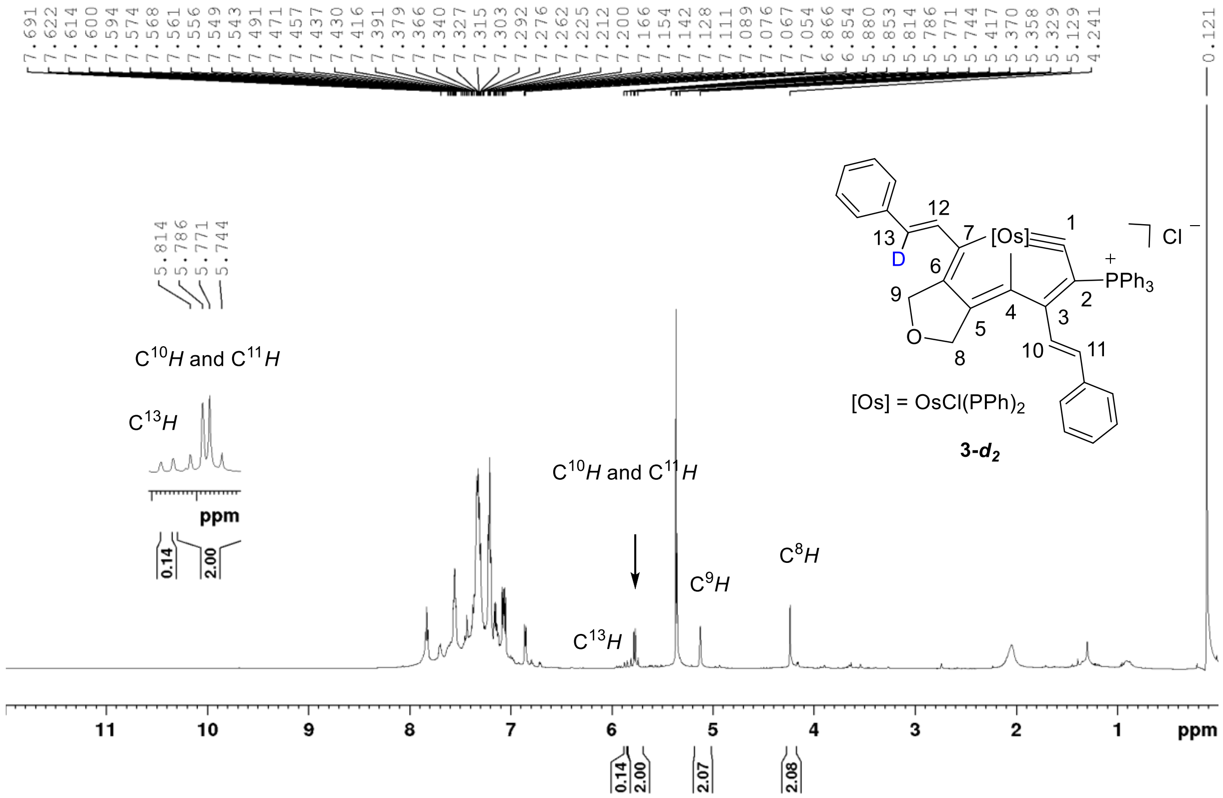

Supplementary Figure 208. The ^{1}H NMR (600.1 MHz, CD_2Cl_2) spectrum for complex **3-*d*₂**.

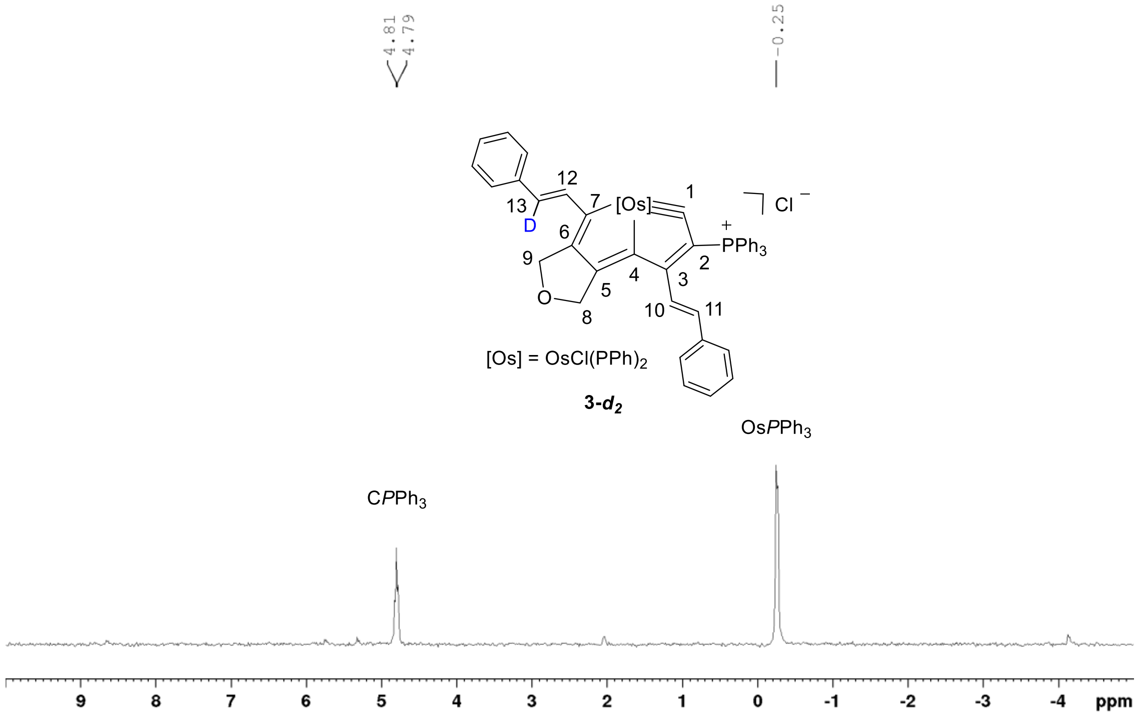

Supplementary Figure 209. The ^{31}P{^{1}H} NMR (242.9 MHz, CD_2Cl_2) spectrum for complex **3-*d*₂**.

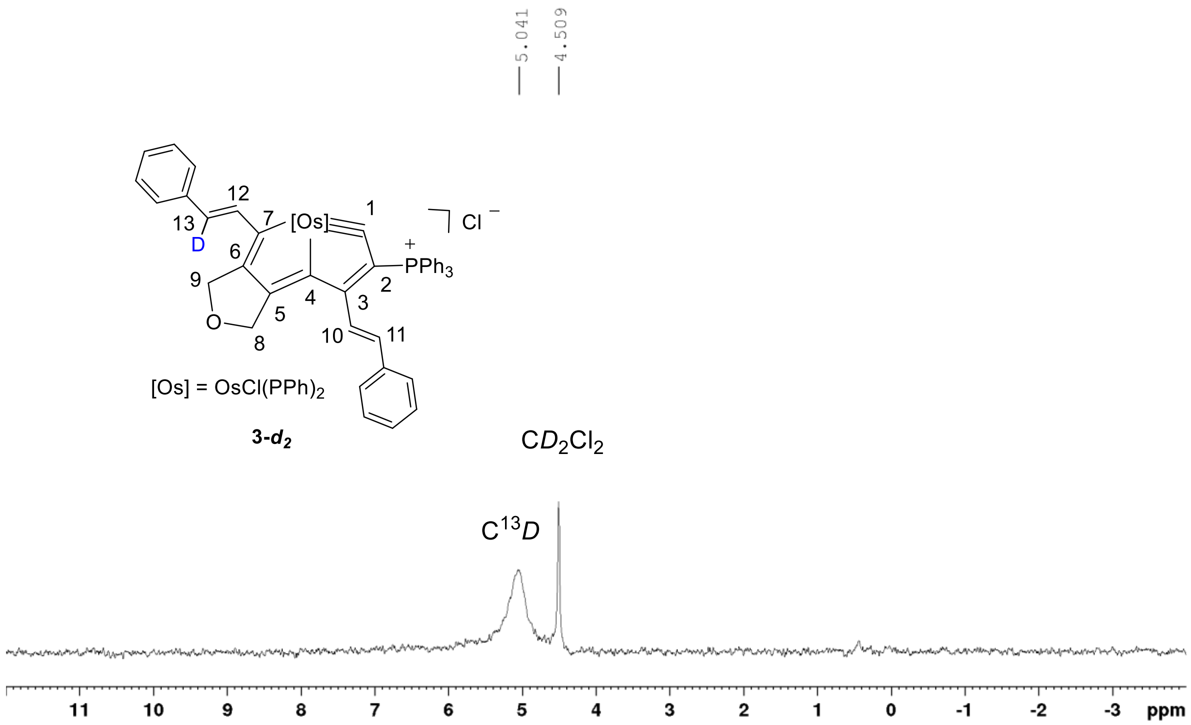

Supplementary Figure 210. The ^{2}D NMR (92.1 MHz, CH_2Cl_2) spectrum for complex **3-*d*₂**.

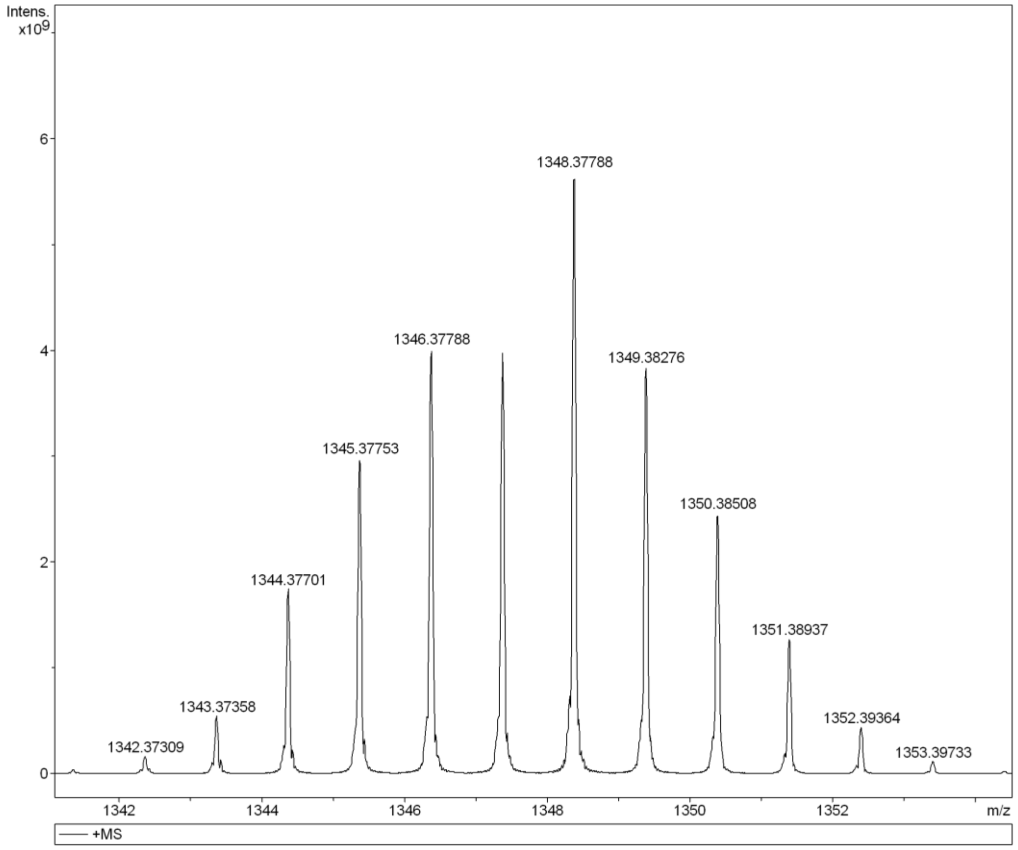

Supplementary Figure 211. Positive-ion ESI-MS spectrum of [**3-*d*₂**]$^{+}$ measured in methanol.

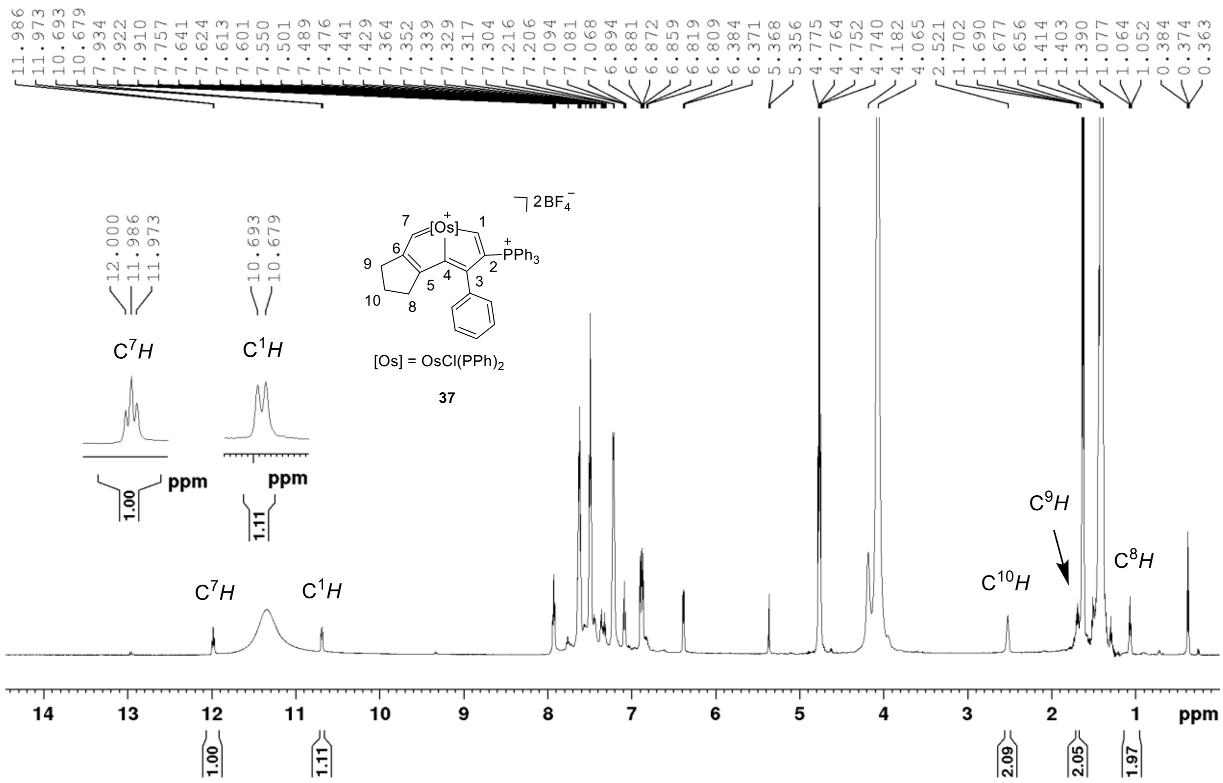

Supplementary Figure 212. The ^{1}H NMR (600.1 MHz, CD_2Cl_2) spectrum for complex **37**.

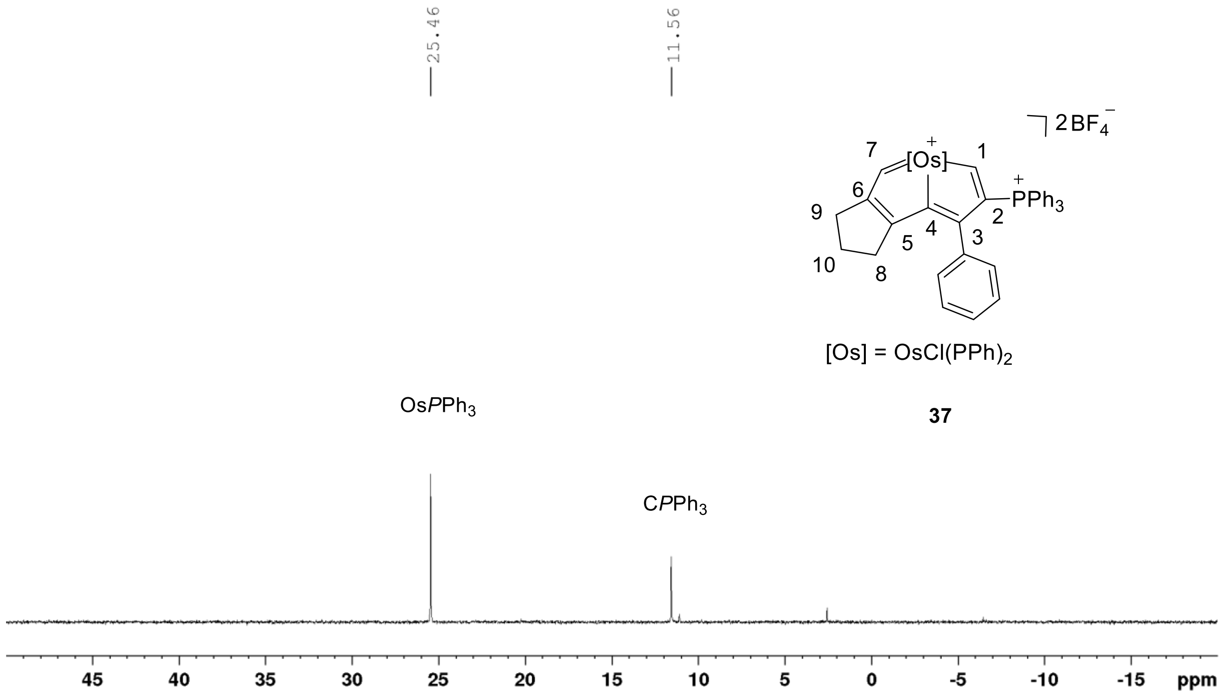

Supplementary Figure 213. The $^{31}P\{^1H\}$ NMR (242.9 MHz, CD_2Cl_2) spectrum for complex **37**.

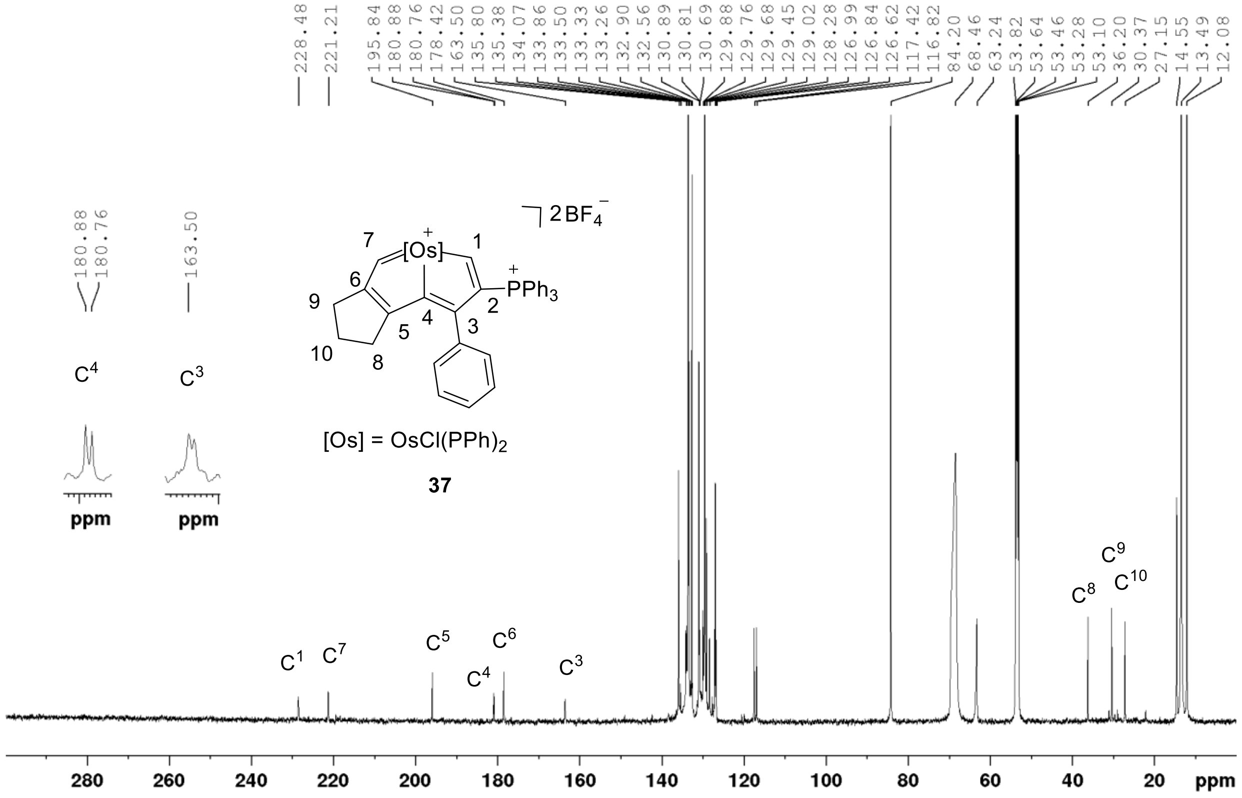

Supplementary Figure 214. The $^{13}C\{^1H\}$ NMR (150.9 MHz, CD_2Cl_2) spectrum for complex **37**.

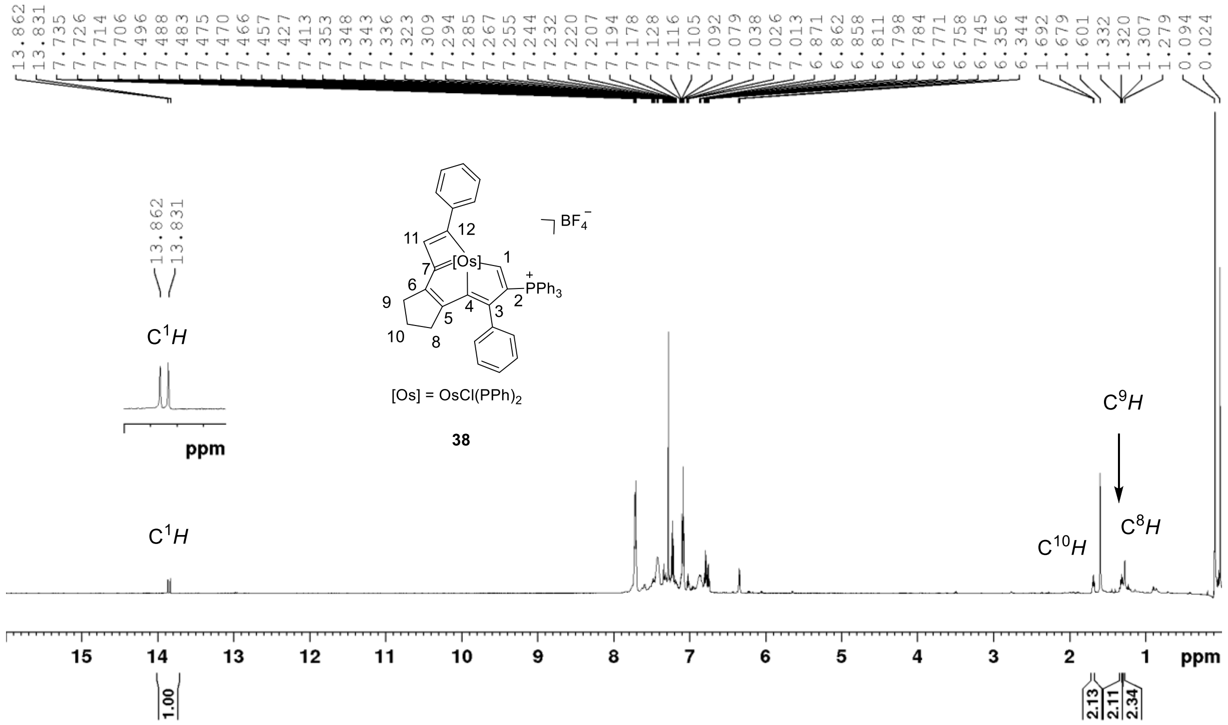

Supplementary Figure 215. The ^1H NMR (600.1 MHz, $CDCl_3$) spectrum for complex **38**.

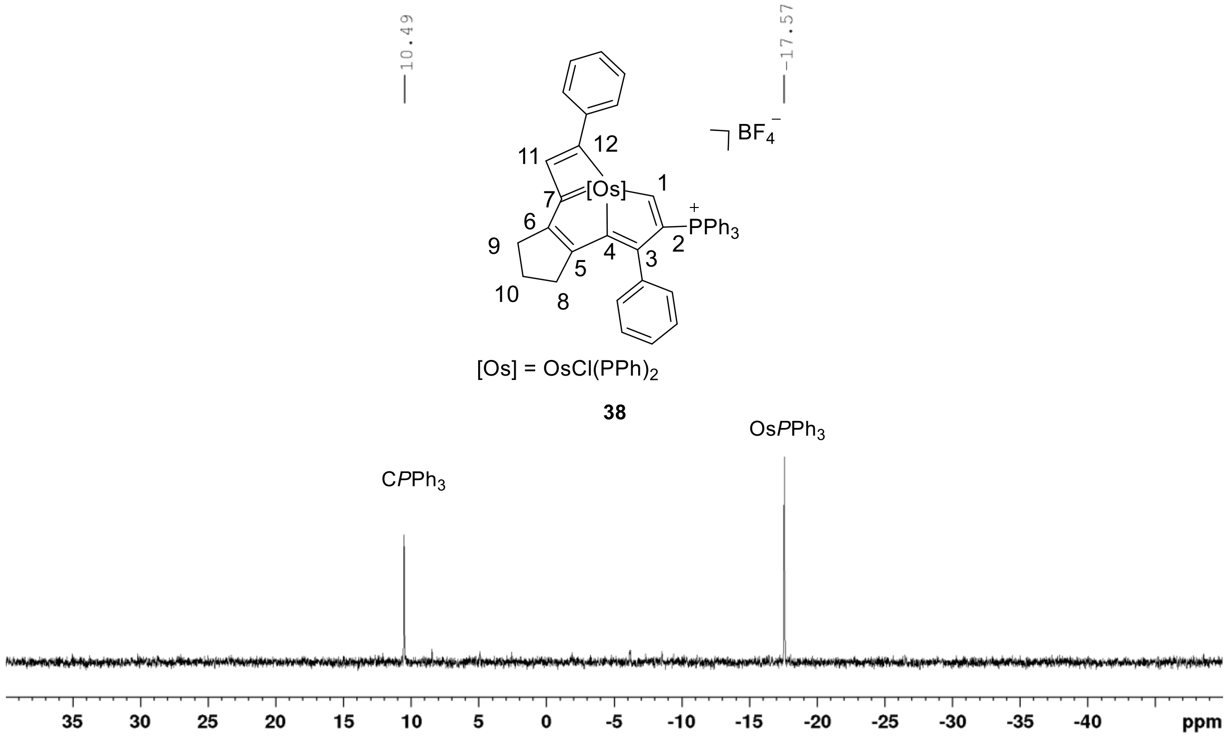

Supplementary Figure 216. The $^{31}P\{^1H\}$ NMR (242.9 MHz, CD_2Cl_2) spectrum for complex **38**.

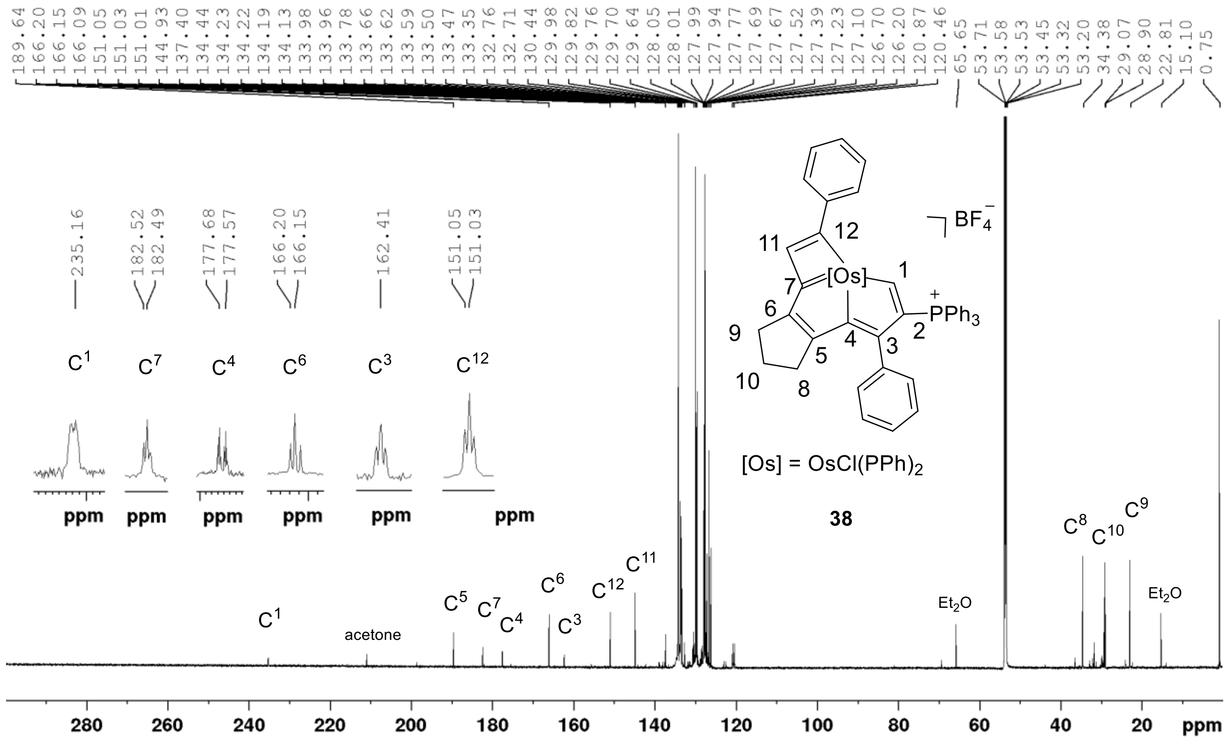

Supplementary Figure 217. The $^{13}C\{^1H\}$ NMR (150.9 MHz, CD_2Cl_2) spectrum for complex **38**.

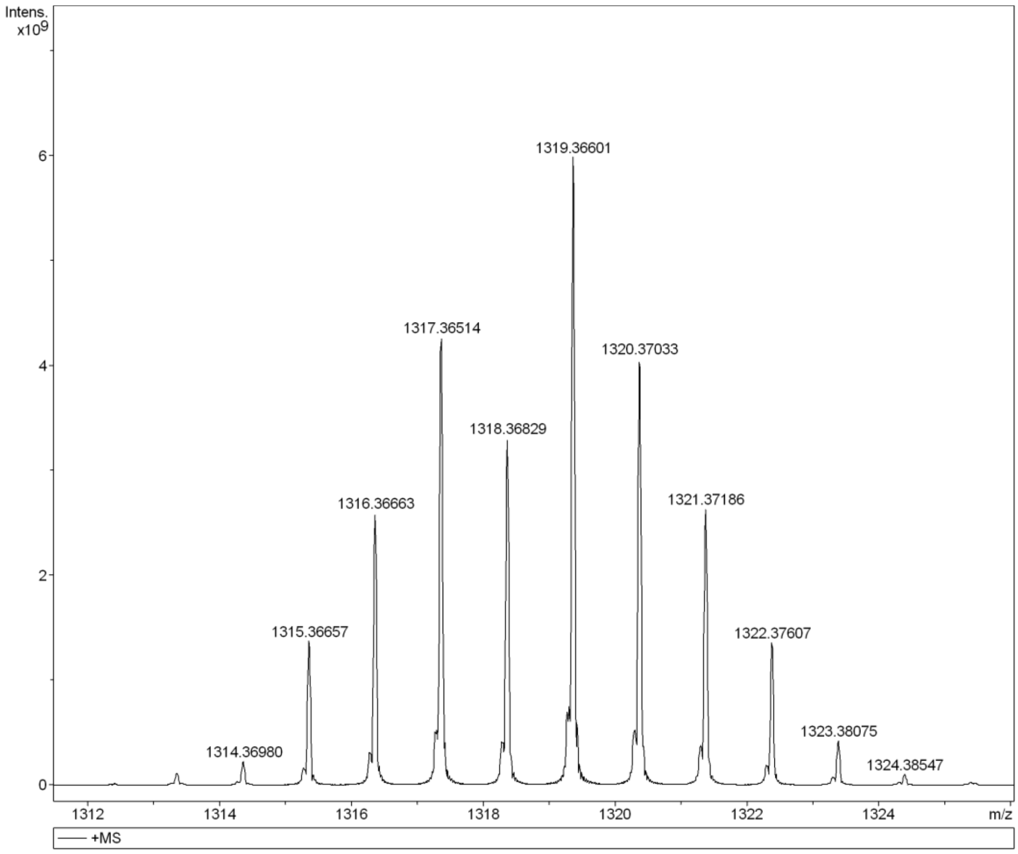

Supplementary Figure 218. Positive-ion ESI-MS spectrum of [**38**]$^+$ measured in methanol.

Supplementary Methods

Synthesis of Multiyne Chain:

Synthesis pathway for triyne L1.

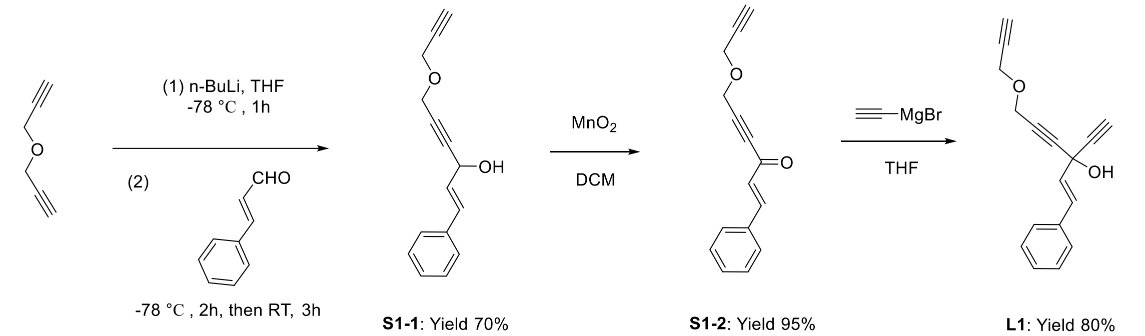

S1-1: Yield 70% **S1-2**: Yield 95% **L1**: Yield 80%

Preparation of compound S1-1: To an oven-dried flask containing propargyl ether (5.47 mL, 5.00 g, 53.1 mmol) and anhydrous tetrahydrofuran (200 mL), *n*-butyllithium (25.0 mL, 2.4 M in tetrahydrofuran, 60.0 mmol) was added dropwise over 45 min under N_2 at –78 ℃. After the reaction mixture was stirred for an additional 15 min, cinnamaldehyde (7.01 mL, 53.1 mmol) was added dropwise at the same temperature. After 2 h, the reaction was warmed to room temperature and stirred for 3 h. The reaction was quenched with saturated aqueous NH_4Cl (70 mL). The resulting mixture was extracted with Et_2O (3 × 250 mL), and the extract was washed with saturated brine (2 × 150 mL), dried over $MgSO_4$, and concentrated to dryness. The residue was chromatographed with hexane/EtOAc (5:1) to afford **S1-1** as a yellow oil. Yield: 8.41 g, 70%. ^{1}H NMR (400.1 MHz, $CDCl_3$): δ = 7.42 (d, *J* = 7.28 Hz, 2H), 7.31 (m, 3H), 6.78 (d, *J* = 15.96 Hz, 1H), 6.31 (dd, *J* = 16.11 Hz, *J* = 5.99 Hz, 1H), 5.12 (td, *J* = 5.92 Hz, *J* = 1.46 Hz, 1H), 4.37 (d, *J* = 1.79 Hz, 2H), 4.29 (d, *J* = 2.38 Hz, 2H), 2.48 (t, *J* = 2.44 Hz, 1H), 2.18 (d, *J* = 5.97 Hz, 1H). ^{13}C NMR (100.6 MHz, $CDCl_3$): δ = 136.1, 131.9, 128.6, 128.1, 127.9, 126.8, 86.25, 81.30, 78.91, 75.38, 62.74, 56.86, 56.62. HRMS (ESI): *m/z* calcd for $[C_{15}H_{14}O_2Na]^+$, 249.0886; found 249.0886.

Preparation of compound S1-2: To a solution of **S1-1** (8.41 g, 37.2 mmol) in dichloromethane (150 mL), then the solid MnO_2 (32.1 g, 372 mmol) powder was added. The reaction mixture was stirred for 2 h at room temperature. The reaction solution was chromatographed with dichloromethane to afford **S1-2** as a yellow solid. Yield: 7.99 g, 95%. ^{1}H NMR (600.1 MHz, $CDCl_3$): δ = 7.83 (d, *J* = 16.17 Hz, 1H), 7.58 (dd, *J* = 7.87 Hz, *J* = 1.29 Hz, 2H), 7.43 (m, 3H), 6.79 (d, *J* = 16.19 Hz, 1H), 4.54 (s,

2H), 4.35 (d, *J* = 2.35 Hz, 2H), 2.54 (t, *J* = 2.36 Hz, 1H). ^{13}C NMR (150.9 MHz, $CDCl_3$): δ = 177.5, 149.1, 133.9, 131.4, 129.1, 128.8, 128.0, 87.38, 84.12, 78.37, 75.87, 57.15, 56.54. HRMS (ESI): *m/z* calcd for $[C_{15}H_{12}O_2Na]^+$, 247.0730; found 247.0720.

Preparation of compound L1: To an oven-dried flask containing ethynylmagnesium bromide (120 mL, 0.5 M in tetrahydrofuran, 60.0 mmol), then the anhydrous tetrahydrofuran (10 mL) solution of **S1-2** (7.99 g, 32.3 mmol) was added dropwise over 45 min under N_2 at 0 ℃. The reaction was warmed to room temperature and stirred for 12 h. The reaction mixture was quenched with saturated aqueous NH_4Cl (70 mL). The resulting mixture was extracted with Et_2O (3 × 250 mL), and the extract was washed with saturated brine (2 × 150 mL), dried over $MgSO_4$, and concentrated to dryness. The residue was chromatographed with hexane/EtOAc (5:1) to afford **L1** as a yellow oil. Yield: 6.46 g, 80%. ^{1}H NMR (500.2 MHz, $CDCl_3$): δ = 7.46 (d, *J* = 7.32 Hz, 2H), 7.35 (t, *J* = 7.17 Hz, 2H), 7.30 (m, 1H), 7.04 (d, *J* = 15.67 Hz, 1H), 6.38 (d, *J* = 15.63 Hz, 1H), 5.30 (br, 1H), 4.39 (s, 2H), 4.30 (d, *J* = 2.38 Hz, 2H), 2.79 (s, 1H), 2.51 (s, 1H). ^{13}C NMR (125.8 MHz, $CDCl_3$): δ = 135.5, 131.3, 128.7, 128.5, 127.2, 85.59, 82.40, 80.28, 78.79, 75.61, 73.66, 63.00, 56.75, 56.72. HRMS (ESI): *m/z* calcd for $[C_{17}H_{14}O_2Na]^+$, 273.0886; found 273.0890.

Synthesis pathway for triyne L2.

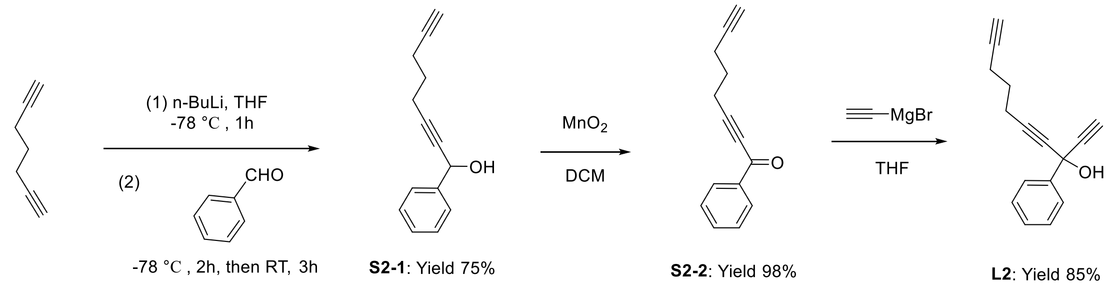

Preparation of compound S2-1: To an oven-dried flask containing 1,6-heptadiyne (5.00 mL, 4.02 g, 43.6 mmol) and anhydrous tetrahydrofuran (200 mL), *n*-butyllithium (18.7 mL, 2.4 M in tetrahydrofuran, 45.0 mmol) was added dropwise over 45 min under N_2 at –78 ℃. After the reaction mixture was stirred for an additional 15 min, benzaldehyde (4.65 mL,43.6 mmol) was added dropwise at the same temperature. After 2 h, the reaction was warmed to room temperature and stirred for 3 h. The reaction was quenched with saturated aqueous NH_4Cl (70 mL). The resulting mixture was extracted with Et_2O (3 × 250 mL), and the extract was washed with saturated brine (2 × 150 mL), dried

over $MgSO_4$, and concentrated to dryness. The residue was chromatographed with hexane/EtOAc (5:1) to afford **S2-1** as a yellow oil. Yield: 6.48 g, 75%. ^{1}H NMR (400.1 MHz, $CDCl_3$): δ = 7.54 (d, *J* = 7.63 Hz, 2H), 7.39 (t, *J* = 7.42 Hz, 2H), 7.34 (m, 1H), 5.45 (s, 1H), 2.58 (s, 1H), 2.43 (t, *J* = 6.97 Hz, 2H), 2.33 (t, *J* = 6.64 Hz, 2H), 2.00 (s, 1H), 1.78 (m, 2H). ^{13}C NMR (100.6 MHz, $CDCl_3$): δ = 141.2, 128.6, 128.2, 126.6, 86.22, 83.49, 80.84, 69.09, 64.70, 27.40, 17.88, 17.60. HRMS (ESI): *m/z* calcd for $[C_{14}H_{14}ONa]^+$, 221.0937; found 221.0938.

Preparation of compound S2-2: To a solution of **S2-1** (6.48 g, 32.7 mmol) in dichloromethane (150 mL), then the solid MnO_2 (28.4 g, 327 mmol) powder was added. The reaction mixture was stirred for 2 h at room temperature. The reaction solution was chromatographed with dichloromethane to afford **S2-2** as a yellow solid. Yield: 6.28 g, 98%. ^{1}H NMR (400.1 MHz, $CDCl_3$): δ = 8.13 (d, *J* = 8.42 Hz, 2H), 7.60 (t, *J* = 7.35 Hz, 1H), 7.47 (t, *J* = 8.05 Hz, 2H), 2.65 (t, *J* = 7.14 Hz, 2H), 2.40 (td, *J* = 7.08 Hz, *J* = 2.66 Hz, 2H), 2.03 (t, *J* = 2.63 Hz, 1H), 1.90 (m, 2H). ^{13}C NMR (100.6 MHz, $CDCl_3$): δ = 178.0, 136.8, 134.0, 129.5, 128.5, 95.19, 82.70, 80.06, 69.58, 26.63, 18.14, 17.71. HRMS (ESI): *m/z* calcd for $[C_{14}H_{12}ONa]^+$, 219.0780; found 219.0782.

Preparation of compound L2: To an oven-dried flask containing ethynylmagnesium bromide (120 mL, 0.5 M in tetrahydrofuran, 60.0 mmol), then the anhydrous tetrahydrofuran (10 mL) solution of **S2-2** (6.28 g, 32.0 mmol) was added dropwise over 45 min under N_2 at 0 ℃. The reaction was warmed to room temperature and stirred for 12 h. The reaction mixture was quenched with saturated aqueous NH_4Cl (70 mL). The resulting mixture was extracted with Et_2O (3 × 250 mL), and the extract was washed with saturated brine (2 ×150 mL), dried over $MgSO_4$, and concentrated to dryness. The residue was chromatographed with hexane/EtOAc (5:1) to afford **L2** as a yellow oil. Yield: 6.04 g, 85%. ^{1}H NMR (400.1 MHz, $CDCl_3$): δ = 7.80 (d, *J* = 7.96Hz, 2H), 7.38 (m, 3H), 2.98 (s, 1H), 2.76 (s, 1H), 2.45 (t, *J* = 7.31 Hz, 2H), 2.32 (td, *J* = 6.91 Hz, *J* = 2.64 Hz, 2H), 1.99 (t, *J* = 2.43 Hz, 1H), 1.80 (m, 2H). ^{13}C NMR (100.6 MHz, $CDCl_3$): δ = 141.8, 128.7, 128.5, 125.7, 85.35, 84.28, 83.32, 80.97, 72.91, 69.08, 64.92, 27.15, 17.84, 17.61. HRMS (ESI): *m/z* calcd for $[C_{16}H_{14}ONa]^+$, 245.0937; found 245.0933.

Synthesis pathway for triyne L3.

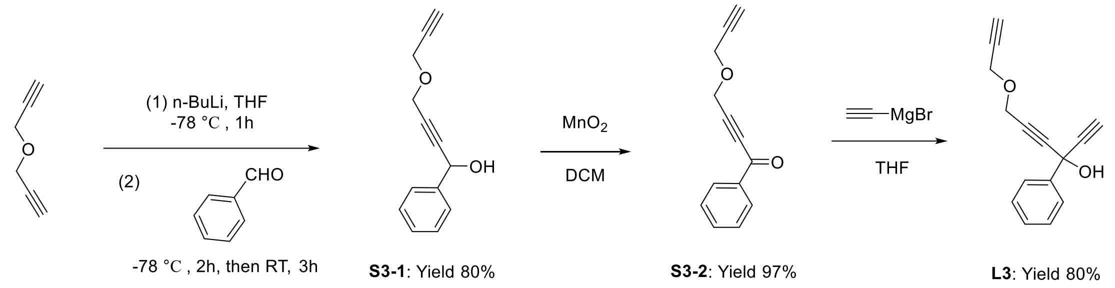

Preparation of compound S3-1: To an oven-dried flask containing propargyl ether (5.00 mL, 4.57 g, 47.6 mmol) and anhydrous tetrahydrofuran (200 mL), *n*-butyllithium (20 mL, 2.4 M in tetrahydrofuran, 48.0 mmol) was added dropwise over 45 min under N_2 at –78 ℃. After the reaction mixture was stirred for an additional 15 min, benzaldehyde (5.00 mL,47.6 mmol) was added dropwise at the same temperature. After 2 h, the reaction was warmed to room temperature and stirred for 3 h. The reaction was quenched with saturated aqueous NH_4Cl (70 mL). The resulting mixture was extracted with Et_2O (3 × 250 mL), and the extract was washed with saturated brine (2 × 150 mL), dried over $MgSO_4$, and concentrated to dryness. The residue was chromatographed with hexane/EtOAc (5:1) to afford **S3-1** as a yellow oil. Yield: 7.62 g, 80%. ^{1}H NMR (500.2 MHz, $CDCl_3$): δ = 7.54 (d, *J* = 7.94 Hz, 2H), 7.40 (t, *J* = 7.46 Hz, 2H), 7.37 (m, 1H), 5.52 (s, 1H), 4.37 (s, 2H), 4.27 (s, 2H), 2.77 (br, 1H), 2.48 (m, 1H). ^{13}C NMR (125.8 MHz, $CDCl_3$): δ = 140.3, 128.7, 128.5, 126.6, 86.88, 81.63, 78.85, 75.26, 64.51, 56.86, 56.62. HRMS (ESI): *m/z* calcd for $[C_{13}H_{12}O_2Na]^+$, 223.0730; found 223.0729.

Preparation of compound S3-2: To a solution of **S3-1** (7.62 g, 38.0 mmol) in dichloromethane (150 mL), then the solid MnO_2 (33.0 g, 380 mmol) powder was added. The reaction mixture was stirred for 2 h at room temperature. The reaction solution was chromatographed with dichloromethane to afford **S3-2** as a yellow solid. Yield: 7.30 g, 97%. ^{1}H NMR (500.2 MHz, $CDCl_3$): δ = 8.14 (dd, *J* = 8.52 Hz, *J* = 1.33 Hz, 2H), 7.63 (t, *J* = 7.51 Hz, 1H), 7.50 (t, *J* = 8.05 Hz, 2H), 4.58 (s, 2H), 4.36 (d, *J* = 2.30 Hz, 2H), 2.54 (t, *J* = 2.41 Hz, 1H). ^{13}C NMR (125.8 MHz, $CDCl_3$): δ = 177.3, 136.3, 134.4, 129.6, 128.7, 89.01, 84.47, 78.28, 75.82, 57.20, 56.53. HRMS (ESI): *m/z* calcd for $[C_{13}H_{10}O_2Na]^+$, 221.0573; found 221.0574.

Preparation of compound L3: To an oven-dried flask containing ethynylmagnesium bromide (120 mL, 0.5 M in tetrahydrofuran, 60.0 mmol), then the anhydrous tetrahydrofuran (10 mL) solution of

S3-2 (7.28 g, 36.8 mmol) was added dropwise over 45 min under N_2 at 0 ℃. The reaction was warmed to room temperature and stirred for 12 h. The reaction mixture was quenched with saturated aqueous NH_4Cl (70 mL). The resulting mixture was extracted with Et_2O (3 × 250 mL), and the extract was washed with saturated brine (2 × 150 mL), dried over $MgSO_4$, and concentrated to dryness. The residue was chromatographed with hexane/EtOAc (5:1) to afford **L3** as a yellow oil. Yield: 6.60 g, 80%. ^{1}H NMR (500.2 MHz, $CDCl_3$): δ = 7.81 (d, *J* = 8.07 Hz, 2H), 7.41 (m, 3H), 4.39 (s, 2H), 4.27 (s, 2H), 3.18 (br, 1H), 2.80 (s, 1H), 2.48 (m, 1H). ^{13}C NMR (125.8 MHz, $CDCl_3$): δ = 141.0, 128.9, 128.6, 125.8, 86.67, 83.53, 80.56, 78.73, 75.33, 73.67, 64.82, 56.73. HRMS (ESI): *m/z* calcd for $[C_{15}H_{12}O_2Na]^+$, 247.0729; found 247.0727.

Synthesis pathway for triyne L4.

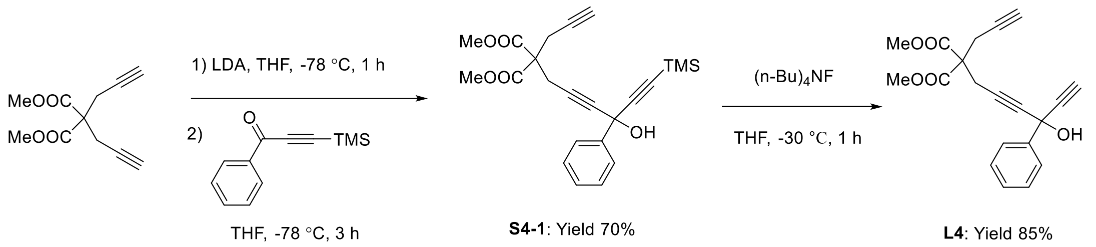

Preparation of compound S4-1: To an oven-dried flask containing dimethyl dipropargyl malonate (5.00 g, 24.0 mmol) and anhydrous tetrahydrofuran (200 mL), lithium diisopropylamide (20 mL, 2.0 M in tetrahydrofuran, 24.0 mmol) was added dropwise over 45 min under N_2 at –78 ℃. After the reaction mixture was stirred for an additional 15 min, Phenyl trimethylsilylethynyl ketone (4.85 g, 24.0 mmol) was added dropwise at the same temperature. After 3h, the reaction was quenched with saturated aqueous NH_4Cl (70 mL). The resulting mixture was extracted with Et_2O (3 × 250 mL), and the extract was washed with saturated brine (2 × 150 mL), dried over $MgSO_4$, and concentrated to dryness. The residue was chromatographed with hexane/EtOAc (5:1) to afford **S4-1** as a yellow oil. Yield: 6.90 g, 70%.

Preparation of compound L4: To a solution of **S4-1** (6.00 g, 14.6 mmol) in tetrahydrofuran (150 mL), then the tetrabutylammonium fluoride (30 mL, 1.0 M in tetrahydrofuran, 30.0 mmol) was added dropwise at -30 ℃. After 1 h, the reaction mixture was quenched with saturated aqueous NH_4Cl (70 mL). The resulting mixture was extracted with Et_2O (3 × 250 mL), and the extract was washed with saturated brine (2 × 150 mL), dried over $MgSO_4$, and concentrated to dryness. The residue was

chromatographed with hexane/EtOAc (5:1) to afford **L4** as a yellow solid. Yield: 4.19 g, 85%. ^{1}H NMR (500.2 MHz, $CDCl_3$): δ = 7.76 (d, *J* = 8.28 Hz, 2H), 7.40 (t, *J* = 7.77 Hz, 2H), 7.36 (m, 1H), 3.75 (d, *J* = 3.56 Hz, 6H), 3.18 (br, 1H), 3.11 (s, 2H), 2.99 (s, 2H), 2.76 (s, 1H), 2.06 (s, 1H). ^{13}C NMR (125.8 MHz, $CDCl_3$): δ = 169.1, 141.4, 128.8, 128.5, 125.7, 83.86, 83.76, 80.40, 78.29, 73.15, 71.96, 64.75, 56.67, 53.19, 23.09, 22.97. HRMS (ESI): *m/z* calcd for $[C_{20}H_{18}O_5Na]^+$, 361.1046; found 361.1041.

Synthesis pathway for triyne L5.

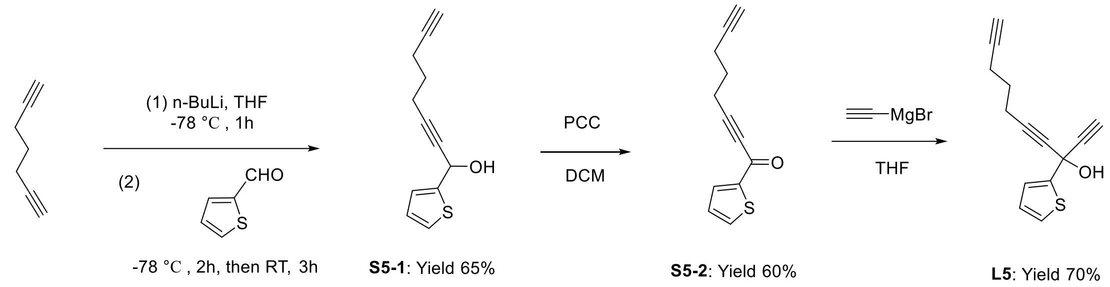

Preparation of compound S5-1: To an oven-dried flask containing 1,6-heptadiyne (5.00 mL, 4.02 g, 43.6 mmol) and anhydrous tetrahydrofuran (200 mL), *n*-butyllithium (18.7 mL, 2.4 M in tetrahydrofuran, 45.0 mmol) was added dropwise over 45 min under N_2 at –78 ℃. After the reaction mixture was stirred for an additional 15 min, 2-Thenaldehyde (4.07 mL,43.6 mmol) was added dropwise at the same temperature. After 2 h, the reaction was warmed to room temperature and stirred for 3 h. The reaction was quenched with saturated aqueous NH_4Cl (70 mL). The resulting mixture was extracted with Et_2O (3 × 250 mL), and the extract was washed with saturated brine (2 × 150 mL), dried over $MgSO_4$, and concentrated to dryness. The residue was chromatographed with hexane/EtOAc (5:1) to afford **S5-1** as a yellow oil. Yield: 5.78 g, 65%.

Preparation of compound S5-2: The **S5-1** (5.78 g, 28.3 mmol) was dissolved in dichloromethane (150 mL), then the pyridinium chlorochromate (12.2 g, 56.6 mmol) powder was added. The reaction mixture was stirred for 2 h at room temperature. The reaction solution was chromatographed with dichloromethane to afford **S5-2** as a yellow solid. Yield: 3.43 g, 60%. ^{1}H NMR (400.1 MHz, $CDCl_3$): δ = 7.88 (dd, *J* = 3.90 Hz, *J* = 1.27 Hz, 1H), 7.68 (dd, *J* = 4.94 Hz, *J* = 1.29 Hz, 1H), 7.14 (dd, *J* = 5.20 Hz, *J* = 3.80 Hz, 1H), 2.62 (t, *J* = 7.36 Hz, 2H), 2.38 (td, *J* = 6.98 Hz, *J* = 2.54 Hz, 2H), 2.02 (t, *J* = 2.58 Hz, 1H), 1.87 (m, *J* = 7.31 Hz, 2H). ^{13}C NMR (100.6 MHz, $CDCl_3$): δ = 169.8, 144.9,

135.1, 135.0, 128.3, 93.74, 82.69, 79.71, 69.62, 26.57, 18.04, 17.66. HRMS (ESI): *m/z* calcd for $[C_{12}H_{10}OSNa]^+$, 225.0345; found 225.0347.

Preparation of compound L5: To an oven-dried flask containing ethynylmagnesium bromide (120 mL, 0.5 M in tetrahydrofuran, 60.0 mmol), then the anhydrous tetrahydrofuran (10 mL) solution of **S5-2** (3.03 g, 15.0 mmol) was added dropwise over 45 min under N_2 at 0 ℃. The reaction was warmed to room temperature and stirred for 12 h. The reaction mixture was quenched with saturated aqueous NH_4Cl (70 mL). The resulting mixture was extracted with Et_2O (3 × 250 mL), and the extract was washed with saturated brine (2 ×150 mL), dried over $MgSO_4$, and concentrated to dryness. The residue was chromatographed with hexane/EtOAc (5:1) to afford **L5** as a yellow oil. Yield: 2.39 g, 70%. ^{1}H NMR (400.1 MHz, $CDCl_3$): δ = 7.36 (dd, *J* = 3.77 Hz, *J* = 1.33 Hz, 1H), 7.32 (dd, *J* = 5.24 Hz, *J* = 1.12 Hz, 1H), 6.97 (dd, *J* = 5.47 Hz, *J* = 3.83 Hz, 1H), 3.21 (s, 1H), 2.79 (s,1H), 2.45 (t, *J* = 7.06 Hz, 2H), 2.34 (td, *J* = 7.58 Hz, *J* = 2.58 Hz, 2H), 2.00 (t, *J* = 2.93 Hz, 1H), 1.81 (m, *J* = 6.76 Hz, 2H). ^{13}C NMR (100.6 MHz, $CDCl_3$): δ = 146.6, 126.6, 126.5, 125.7, 84.93, 83.50, 83.36, 80.46, 72.46, 69.19, 61.58, 27.05, 17.78, 17.61. HRMS (ESI): *m/z* calcd for $[C_{14}H_{12}OSNa]^+$, 251.0501; found 251.0508.

Synthesis of Osmapentalynes:

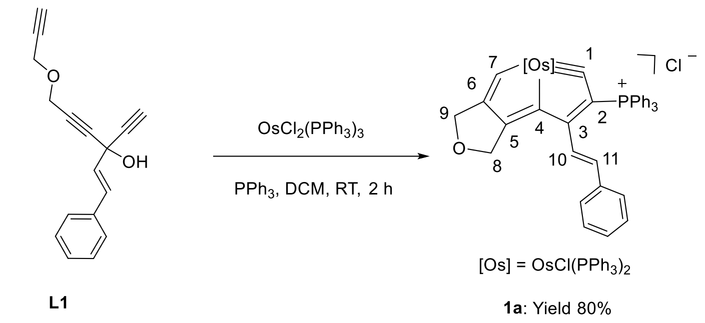

Preparation of complex 1a: A dichloromethane solution (5 mL) of **L1** (1.50 g, 6.00 mmol) was added slowly to a green solution of $OsCl_2(PPh_3)_3$ (6.30 g, 6.00 mmol) and PPh_3 (7.88 g, 30.0 mmol; the excess PPh_3 may assist in increasing the stability of $OsCl_2(PPh_3)_3$) in dichloromethane (150 mL) under an N_2 atmosphere. The reaction mixture was stirred at room temperature for 2 h to yield a brown solution. The solution was evaporated under vacuum to a volume of approximately 15 mL and then washed with Et_2O (3 × 200 mL) to afford a brown solid. The solid was purified by flash chromatography on silica gel (eluent: 20:1 dichloromethane/methanol) to yield complex **1a** as a brown solid. Yield: 6.33 g, 80%. ^1H NMR plus ^1H-^{13}C HSQC (600.1 MHz, CD_2Cl_2): δ = 13.09 (s, 1H, C^7H), 7.89–6.90 (50H, other aromatic protons), 5.92-5.77 (q, J = 16.95 Hz, 2H, $C^{11}H$ and $C^{10}H$), 4.67 (s, 2H, C^9H), 3.82 ppm (s, 2H, C^8H). ^{31}P NMR (242.9 MHz, CD_2Cl_2): δ = 6.20 (t, $J_{P\text{-}P}$ = 5.87 Hz, C*P*Ph3), 2.63 ppm (s, Os*P*Ph3). ^{13}C NMR plus DEPT-135, ^1H-^{13}C HSQC and ^1H-^{13}C HMBC (150.9 MHz, CD_2Cl_2): δ = 318.0 (dt, apparent q, $J_{P\text{-}C}$ = 12.54 Hz, $J_{P\text{-}C}$ = 12.54 Hz, C^1), 210.3 (m, C^7), 172.2 (s, C^5), 171.6 (s, C^6), 168.7 (d, $J_{P\text{-}C}$ = 22.27 Hz, C^4), 162.0 (d, $J_{P\text{-}C}$ = 15.26 Hz, C^3), 137.6 (s, C^{11}), 124.2 (s, C^{10}), 73.57 (s, C^8), 68.78 (s, C^9), 135.1–119.5 ppm (other aromatic carbons). Elemental analysis calcd (%) for $C_{71}H_{57}Cl_2OOsP_3$: C 66.61, H 4.49; found: C 66.57, H 4.22. HRMS (ESI): *m/z* calcd for $[C_{71}H_{57}ClOOsP_3]^+$, 1245.2916; found, 1245.3083.

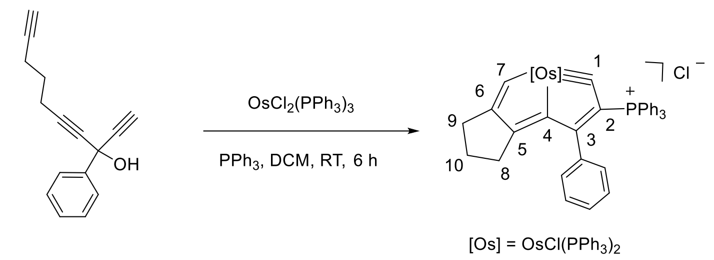

L2

1b: Yield 85%

Preparation of complex 1b: A dichloromethane solution (5 mL) of **L2** (1.50 g, 6.57 mmol) was added slowly to a green solution of $OsCl_2(PPh_3)_3$ (6.90 g, 6.00 mmol) and PPh_3 (7.88 g, 30.0 mmol; the excess PPh_3 may assist in increasing the stability of $OsCl_2(PPh_3)_3$) in dichloromethane (150 mL) under an N_2 atmosphere. The reaction mixture was stirred at room temperature for 6 h to yield a brown solution. The solution was evaporated under vacuum to a volume of approximately 15 mL and then washed with Et_2O (3 × 200 mL) to afford a brown solid. The solid was purified by flash chromatography on silica gel (eluent: 20:1 dichloromethane/methanol) to yield complex **1b** as a brown solid. Yield: 7.00 g, 85%. ^1H NMR plus ^1H-^{13}C HSQC (600.1 MHz, CD_2Cl_2): δ = 13.20 (s, 1H, C^7H), 7.82–6.03 (50H, other aromatic protons), 2.53 (m, 2H, C^9H), 1.74 (t, 2H, $C^{10}H$), 1.03 ppm (s, J = 7.54 Hz, 2H, C^8H). ^{31}P NMR (242.9 MHz, CD_2Cl_2): δ = 5.78 (d, $J_{P\text{-}P}$ = 5.80 Hz, CPPh3), 3.70 ppm (s, OsPPh3). ^{13}C NMR plus DEPT-135, ^1H-^{13}C HSQC and ^1H-^{13}C HMBC (150.9 MHz, CD_2Cl_2): δ = 318.0 (dt, apparent q, $J_{P\text{-}C}$ = 13.02 Hz, $J_{P\text{-}C}$ = 13.02 Hz, C^1), 214.6 (m, C^7), 180.8 (s, C^5), 176.3 (s, C^6), 168.3 (d, $J_{P\text{-}C}$ = 21.94 Hz, C^4), 163.8 (d, $J_{P\text{-}C}$ = 16.86 Hz, C^3), 35.33 (s, C^8), 29.55 (s, C^9), 29.49 (s, C^{10}), 137.7–120.3 ppm (other aromatic carbons). Elemental analysis calcd (%) for $C_{70}H_{57}Cl_2OsP_3$: C 67.14, H 4.59; found: C 67.01, H 4.88. HRMS (ESI): m/z calcd for $[C_{70}H_{57}ClOsP_3]^+$, 1217.2967; found, 1217.3133.

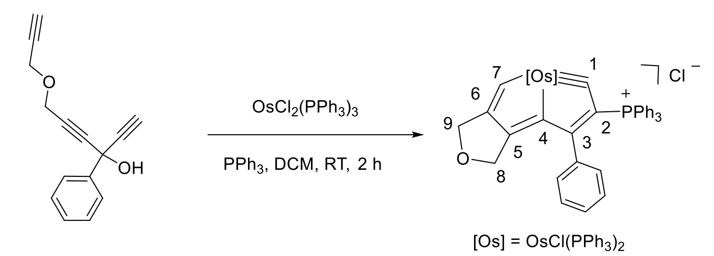

L3 **1c**: Yield 80%

Preparation of complex 1c: A dichloromethane solution (5 mL) of **L3** (1.50 g, 6.70 mmol) was added slowly to a green solution of $OsCl_2(PPh_3)_3$ (7.70 g, 6.00 mmol) and PPh_3 (8.80 g, 33.5 mmol; the excess PPh_3 may assist in increasing the stability of $OsCl_2(PPh_3)_3$) in dichloromethane (150 mL) under an N_2 atmosphere. The reaction mixture was stirred at room temperature for 2 h to yield a brown solution. The solution was evaporated under vacuum to a volume of approximately 15 mL and then washed with Et_2O (3 × 200 mL) to afford a brown solid. The solid was purified by flash chromatography on silica gel (eluent: 20:1 dichloromethane/methanol) to yield complex **1c** as a brown solid. Yield: 6.54 g, 80%. ^1H NMR plus ^1H-^{13}C HSQC (600.1 MHz, CD_2Cl_2): δ = 13.10 (s, 1H, C^7*H*), 7.85–6.04 (50H, other aromatic protons), 4.60 (s, 2H, C^9*H*), 3.14 ppm (s, 2H, C^8*H*). ^{31}P NMR (242.9 MHz, CD_2Cl_2): δ = 6.12 (d, $J_{P\text{-}P}$ = 6.04 Hz, C*P*Ph₃), 2.86 ppm (s, Os*P*Ph₃). ^{13}C NMR plus DEPT-135, ^1H-^{13}C HSQC and ^1H-^{13}C HMBC (150.9 MHz, CD_2Cl_2): δ = 317.8 (dt, apparent q, $J_{P\text{-}C}$ = 12.68 Hz, $J_{P\text{-}C}$ = 12.68 Hz, C^1), 210.4 (m, C^7), 173.4 (s, C^5), 171.9 (s, C^6), 166.5 (d, $J_{P\text{-}C}$ = 23.84 Hz, C^4), 165.6 (d, $J_{P\text{-}C}$ = 15.55 Hz, C^3), 72.87 (s, C^8), 68.51 (s, C^9), 136.7–119.7 ppm (other aromatic carbons). Elemental analysis calcd (%) for $C_{69}H_{55}Cl_2OOsP_3$: C 66.08, H 4.42; found: C 65.89, H 4.65. HRMS (ESI): *m/z* calcd for $[C_{69}H_{55}ClOOsP_3]^+$, 1219.2759; found, 1219.2949.

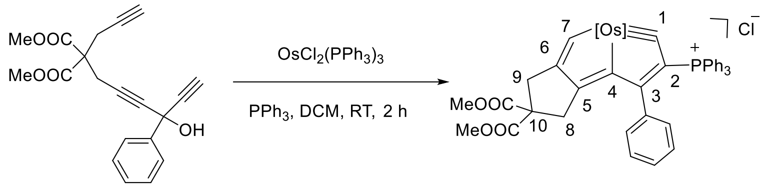

$[Os] = OsCl(PPh_3)_2$

L4 **1d**: Yield 80%

Preparation of complex 1d: A dichloromethane solution (5 mL) of **L4** (1.50 g, 4.44 mmol) was added slowly to a green solution of $OsCl_2(PPh_3)_3$ (5.70 g, 4.44 mmol) and PPh_3 (7.88 g, 30.0 mmol; the excess PPh_3 may assist in increasing the stability of $OsCl_2(PPh_3)_3$) in dichloromethane (150 mL) under an N_2 atmosphere. The reaction mixture was stirred at room temperature for 2 h to yield a brown solution. The solution was evaporated under vacuum to a volume of approximately 15 mL and then washed with Et_2O (3 × 200 mL) to afford a brown solid. The solid was purified by flash chromatography on silica gel (eluent: 20:1 dichloromethane/methanol) to yield complex **1d** as a brown solid. Yield: 4.74 g, 80%. ^1H NMR plus ^1H-^{13}C HSQC (600.1 MHz, CD_2Cl_2): δ = 12.95 (s, 1H, C^7*H*), 7.83–6.14 (50H, other aromatic protons), 3.65 (s, 6H, COOCH_3), 3.15 (s, 2H, C^9*H*), 1.69 ppm (s, 2H, C^8*H*). ^{31}P NMR (242.9 MHz, CD_2Cl_2): δ = 5.83 (t, $J_{P\text{-}P}$ = 5.94 Hz, C*P*Ph_3), 3.42 ppm (d, $J_{P\text{-}P}$ = 5.69 Hz, Os*P*Ph_3). ^{13}C NMR plus DEPT-135, ^1H-^{13}C HSQC and ^1H-^{13}C HMBC (150.9 MHz, CD_2Cl_2): δ = 317.9 (dt, apparent q, $J_{P\text{-}C}$ = 13.10 Hz, $J_{P\text{-}C}$ = 13.10 Hz, C^1), 215.9 (m, C^7), 173.3 (s, C^5), 171.6 (s, *C*OOMe), 170.7 (s, C^6), 168.3 (d, $J_{P\text{-}C}$ = 22.39 Hz, C^4), 165.1 (d, $J_{P\text{-}C}$ = 16.29 Hz, C^3), 64.29 (s, C^{10}), 52.88 (s, COO*C*H_3), 41.78 (s, C^8), 37.12 (s, C^9), 137.0–119.9 ppm (other aromatic carbons). Elemental analysis calcd (%) for $C_{74}H_{61}Cl_2O_4OsP_3$: C 64.96, H 4.49; found: C 64.76, H 4.31. HRMS (ESI): *m/z* calcd for $[C_{74}H_{61}ClO_4OsP_3]^+$, 1333.3077; found, 1333.3243.

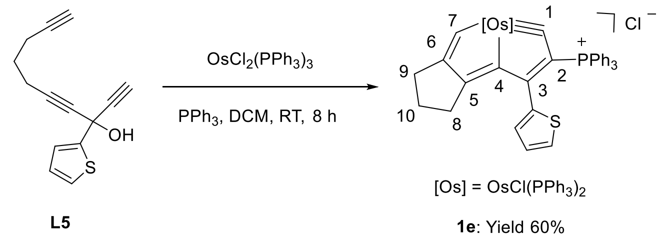

Preparation of complex 1e: A dichloromethane solution (5 mL) of **L5** (0.15 g, 0.66 mmol) was added slowly to a green solution of $OsCl_2(PPh_3)_3$ (0.85 g, 0.45 mmol) and PPh_3 (0.79 g, 3.00 mmol; the excess PPh_3 may assist in increasing the stability of $OsCl_2(PPh_3)_3$) in dichloromethane (15 mL) under an N_2 atmosphere. The reaction mixture was stirred at room temperature for 8 h to yield a brown solution. The solution was evaporated under vacuum to a volume of approximately 5 mL and then washed with Et_2O (3 × 50 mL) to afford a brown solid. The solid was purified by flash chromatography on silica gel (eluent: 20:1 dichloromethane/methanol) to yield complex **1e** as a brown solid. Yield: 4.74 g, 60%. ^{1}H NMR plus ^{1}H-^{13}C HSQC (600.1 MHz, CD_2Cl_2): δ = 13.32 (m, 1H, C^7H), 7.81–6.00 (48H, other aromatic protons), 2.50 (s, 2H, C^9H), 1.72 ppm (s, 2H, $C^{10}H$), 0.92 ppm (s, 2H, C^8H). ^{31}P NMR (242.9 MHz, CD_2Cl_2): δ = 5.79 (s, C*P*Ph$_3$), 5.77 ppm (s, Os*P*Ph$_3$). ^{13}C NMR plus DEPT-135, ^{1}H-^{13}C HSQC and ^{1}H-^{13}C HMBC (150.9 MHz, CD_2Cl_2): δ = 317.6 (dt, apparent q, $J_{P\text{-}C}$ = 13.83 Hz, $J_{P\text{-}C}$ = 13.83 Hz, C^1), 216.8 (m, C^7), 180.8 (s, C^5), 177.1 (s, C^6), 170.7 (d, $J_{P\text{-}C}$ = 21.22 Hz, C^4), 153.4 (d, $J_{P\text{-}C}$ = 15.08 Hz, C^3), 35.17 (s, C^8), 29.66 (s, C^9), 29.51 (s, C^{10}), 137.8–120.3 ppm (other aromatic carbons). Elemental analysis calcd (%) for $C_{68}H_{55}Cl_2OsP_3S$: C 64.91, H 4.41; found: C 64.74, H 4.25. HRMS (ESI): *m/z* calcd for $[C_{68}H_{55}ClOsP_3S]^+$, 1223.2529; found, 1223.2662.

Metal Carbon Triple Bond Shifted:

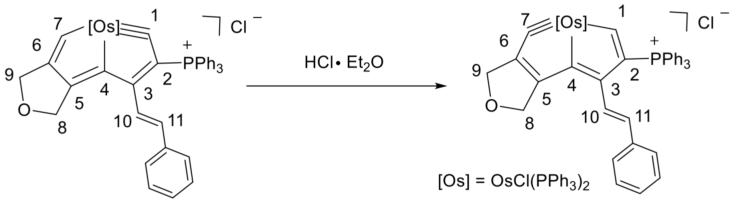

Preparation of complex 2a: The proton acid HCl • Et_2O was added to the dichloromethane solution (5 mL) of **1a** (1.50 g, 1.14 mmol) under an N_2 atmosphere. The reaction mixture was stirred at room temperature for 5 min to yield a brown solution. The solution was evaporated under vacuum to a volume of approximately 15 mL and then washed with Et_2O (3 × 200 mL) to yield complex **2a** as a brown solid (in ca. 75% yield based on ^{1}H- and ^{31}P-NMR). In our previous work[1], density functional theory computations have been employed to explain clearly the mechanism for the formation of **2a** form **1a**. Due to the small free energy reaction barriers between **2a** and **1a**, complex **1a** cannot be convert completely to **2a**. And they coexist in the system in a specific proportion. ^{1}H NMR plus ^{1}H-^{13}C HSQC (600.1 MHz, CD_2Cl_2): δ = 13.39 (d, *J* = 18.42 Hz, 1H, $C^{1}H$), 7.87–6.75 (50H, other aromatic protons), 6.03 (d, *J* = 16.77 Hz, 1H, $C^{11}H$), 5.77 (d, *J* = 16.77 Hz, 1H, $C^{10}H$), 4.31 (s, 2H, $C^{9}H$), 3.14 ppm (s, 2H, $C^{8}H$). ^{31}P NMR (242.9 MHz, CD_2Cl_2): δ = 14.97 (s, C*P*Ph_3), 7.10 ppm (s, Os*P*Ph_3). ^{13}C NMR plus DEPT-135, ^{1}H-^{13}C HSQC and ^{1}H-^{13}C HMBC (150.9 MHz, CD_2Cl_2): δ = 314.8 (s, C^{7}), 221.5 (s, C^{1}), 183.3 (s, C^{5}), 168.9 (d, $J_{P\text{-}C}$ = 19.02 Hz, C^{4}), 163.2 (s, C^{6}), 151.1 (d, $J_{P\text{-}C}$ = 21.90 Hz, C^{3}), 72.18 (s, C^{8}), 64.80 (s, C^{9}), 137.6–119.2 ppm (other aromatic carbons). Elemental analysis calcd (%) for $C_{71}H_{57}Cl_2OOsP_3$: C 66.61, H 4.49; found: C 66.99, H 4.88. HRMS (ESI): *m/z* calcd for $[C_{71}H_{57}ClOOsP_3]^+$, 1245.2916; found, 1245.3083.

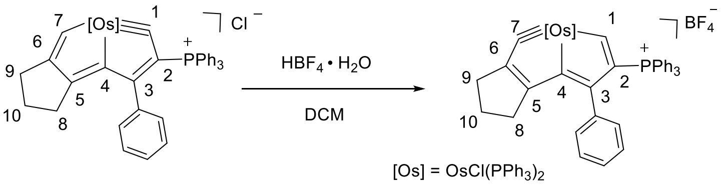

Preparation of complex 2b: The proton acid $HBF_4 \cdot H_2O$ was added to the dichloromethane solution (5 mL) of **1** (1.50 g, 1.23 mmol) under an N_2 atmosphere. The reaction mixture was stirred at room temperature for 5 min to yield a brown solution. The solution was evaporated under vacuum to a volume of approximately 15 mL and then washed with Et_2O (3 × 200 mL) to yield complex **2b** as a brown solid (in ca. 98% yield based on ^{1}H- and ^{31}P-NMR). ^{1}H NMR plus ^{1}H-^{13}C HSQC (600.1 MHz, CD_2Cl_2): δ = 13.50 (d, J = 18.47 Hz, 1H, C^1H), 7.80–6.41 (50H, other aromatic protons), 2.05 (s, 2H, $C^{10}H$), 1.25 (m, 2H, C^9H), 0.44 ppm (t, J = 18.47 Hz, 2H, C^8H). ^{31}P NMR (242.9 MHz, CD_2Cl_2): δ = 13.71 (s, C*P*Ph3), 9.19 ppm (s, Os*P*Ph3). ^{13}C NMR plus DEPT-135, ^{1}H-^{13}C HSQC and ^{1}H-^{13}C HMBC (150.9 MHz, CD_2Cl_2): δ = 320.3 (td, J = 15.92 Hz, J = 6.12 Hz, C^7), 220.1 (s, C^1), 192.6 (s, C^5), 169.8 (d, $J_{P\text{-}C}$ = 19.32 Hz, C^4), 166.5 (s, C^6), 153.3 (d, $J_{P\text{-}C}$ = 22.43 Hz, C^3), 33.77 (s, C^8), 29.24 (s, C^9), 24.64 (s, C^{10}), 138.0–120.2 ppm (other aromatic carbons). Elemental analysis calcd (%) for $C_{70}H_{57}BClFOsP_3$: C 67.44, H 4.61; found: C 67.70, H 4.89. HRMS (ESI): *m/z* calcd for $[C_{70}H_{57}ClOsP_3]^+$, 1217.2967; found, 1217.3179.

Addition of Alkynes and Osmium Carbynes:

Preparation of complex 3:

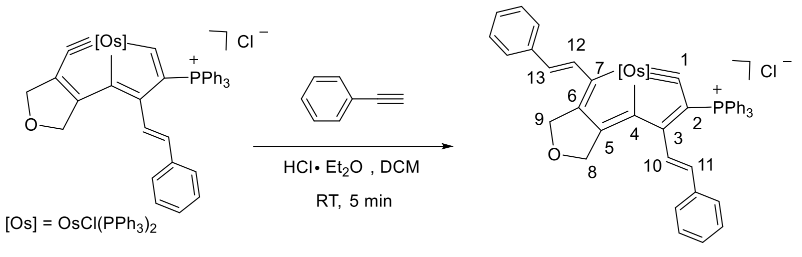

2a **3**: Yield 99%

Method A: The excessive proton acid HCl • Et2O (2 M in ether) solution (2.5 mL) and phenylacetylene (0.50 mmol) was added to the dichloromethane solution (25 mL) of **3** (0.50 g, 0.39 mmol) under an N_2 atmosphere (It is noteworthy that complex **2a** can be slowly oxidized in air and should be reacted under an inert atmosphere in contrast to complex **1a**). The reaction mixture was stirred at room temperature for 5 min to yield a magenta solution. The solution was evaporated under vacuum to a volume of approximately 5 mL and then washed with Et2O (1 × 100 mL) to afford a magenta solid. The solid was purified by flash chromatography on silica gel (eluent: 20:1 dichloromethane/methanol) to yield complex **3** as a magenta solid. Yield: 0.53 g, 99%.

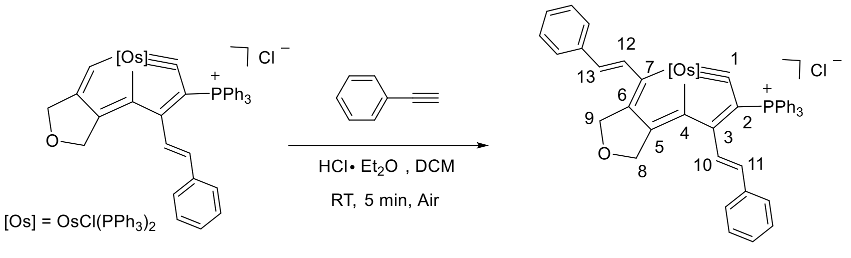

1a **3**: Yield 99%

Method B: The excessive proton acid HCl • Et2O (2 M in ether) solution (5.0 mL) was added to the dichloromethane solution (25 mL) of **1a** (1.00 g, 0.78 mmol) and phenylacetylene (1.00 mmol) under air without using standard Schlenk techniques. The reaction mixture was stirred at room temperature for 5 min to yield a magenta solution. The solution was evaporated under vacuum to a volume of approximately 8 mL and then washed with Et2O (1 × 150 mL) to afford a magenta solid. The solid was purified by flash chromatography on silica gel (eluent: 20:1 dichloromethane/methanol) to yield complex **3** as a magenta solid. Yield: 1.06 g, 99%. ^{1}H NMR plus ^{1}H-^{13}C HSQC (600.1 MHz, CD_2Cl_2): δ = 7.44 (d, *J* = 16.90 Hz, 1H, $C^{12}H$), 5.87 (d, *J* = 16.90 Hz, 1H, $C^{13}H$), 5.78 (d, *J* =

16.45 Hz, 2H, $C^{10}H$ and $C^{11}H$), 5.14 (s, 2H, $C^{9}H$), 4.25 (s, 2H, $C^{8}H$), 7.85–6.86 ppm (55H, other aromatic protons). ^{31}P NMR (242.9 MHz, CD_2Cl_2): δ = 4.81 (t, J = 5.96 Hz, C*P*Ph_3), -0.18 ppm (s, Os*P*Ph_3). ^{13}C NMR plus DEPT-135, ^{1}H-^{13}C HSQC and ^{1}H-^{13}C HMBC (150.9 MHz, CD_2Cl_2): δ = 316.2 (dt, apparent q, $J_{P\text{-}C}$ = 14.02 Hz, $J_{P\text{-}C}$ = 14.02 Hz, C^1), 218.8 (t, $J_{P\text{-}C}$ = 10.29 Hz, C^7), 172.5 (s, C^5), 169.0 (s, C^6), 167.5 (d, $J_{P\text{-}C}$ = 21.96 Hz, C^4), 159.0 (d, $J_{P\text{-}C}$ = 15.91 Hz, C^3), 73.60 (s, C^8), 70.74 (s, C^9), 142.7–119.9 ppm (other aromatic carbons). Elemental analysis calcd (%) for $C_{79}H_{63}Cl_2OOsP_3$: C 68.64, H 4.59; found: C 68.79, H 4.76. HRMS (ESI): *m/z* calcd for $[C_{79}H_{63}ClOOsP_3]^+$, 1347.3387; found, 1347.3621.

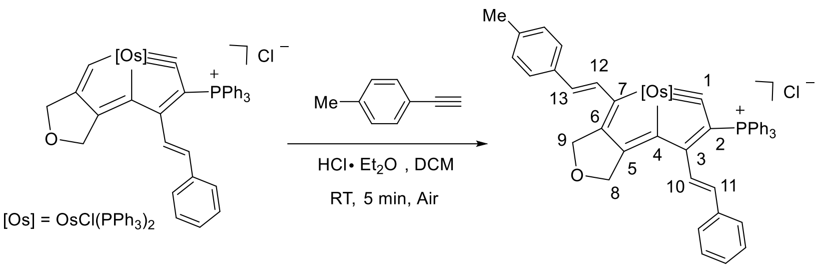

1a **4**: Yield 95%

Preparation of complex 4: The excessive proton acid HCl • Et_2O (2 M in ether) solution (2.5 mL) was added to the dichloromethane solution (25 mL) of **1a** (0.50 g, 0.39 mmol) and 4-Ethynyltoluene (0.50 mmol) under air without using standard Schlenk techniques. The reaction mixture was stirred at room temperature for 5 min to yield a dark purple solution. The solution was evaporated under vacuum to a volume of approximately 5 mL and then washed with Et_2O (1 × 100 mL) to afford a dark purple solid. The solid was purified by flash chromatography on silica gel (eluent: 20:1 dichloromethane/methanol) to yield complex **4** as a dark purple solid. Yield: 0.52 g, 95%. ^{1}H NMR plus ^{1}H-^{13}C HSQC (600.1 MHz, CD_2Cl_2): δ = 5.87 (d, J = 16.33 Hz, 1H, $C^{13}H$), 5.78 (q, J = 16.11 Hz, J = 16.11 Hz, 2H, $C^{10}H$ and $C^{11}H$), 5.12 (s, 2H, $C^{9}H$), 4.24 (s, 2H, $C^{8}H$), 2.33 (s, 3H, CH_3), 7.85–6.86 ppm (55H, other aromatic protons and $C^{12}H$). ^{31}P NMR (242.9 MHz, CD_2Cl_2): δ = 4.78 (s, C*P*Ph_3), -0.02 ppm (s, Os*P*Ph_3). ^{13}C NMR plus DEPT-135, ^{1}H-^{13}C HSQC and ^{1}H-^{13}C HMBC (150.9 MHz, CD_2Cl_2): δ = 316.3 (dt, apparent q, $J_{P\text{-}C}$ = 13.22 Hz, $J_{P\text{-}C}$ = 13.22 Hz, C^1), 219.5 (t, $J_{P\text{-}C}$ = 10.97 Hz, C^7), 172.5 (s, C^5), 168.8 (s, C^6), 167.2 (d, $J_{P\text{-}C}$ = 22.31 Hz, C^4), 158.3 (d, $J_{P\text{-}C}$ = 14.68 Hz, C^3), 73.54 (s, C^8), 70.76 (s, C^9), 21.39 (s, CH_3), 141.9–120.0 ppm (other aromatic carbons). Elemental analysis calcd (%) for $C_{80}H_{65}Cl_2OOsP_3$: C 68.81, H 4.69; found: C 68.45, H 4.41. HRMS (ESI): *m/z* calcd for $[C_{80}H_{65}ClOOsP_3]^+$, 1361.3544; found, 1361.3867.

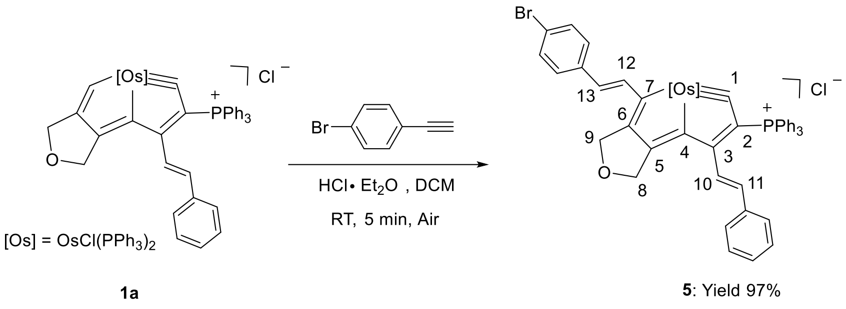

Preparation of complex 5: The excessive proton acid HCl • Et2O (2 M in ether) solution (2.5 mL) was added to the dichloromethane solution (25 mL) of **1a** (0.50 g, 0.39 mmol) and 4-Bromophenylacetylene (0.50 mmol) under air without using standard Schlenk techniques. The reaction mixture was stirred at room temperature for 5 min to yield a magenta solution. The solution was evaporated under vacuum to a volume of approximately 5 mL and then washed with Et2O (1 × 100 mL) to afford a magenta solid. The solid was purified by flash chromatography on silica gel (eluent: 20:1 dichloromethane/methanol) to yield complex **5** as a magenta solid. Yield: 0.55 g, 97%. ^{1}H NMR plus ^{1}H-^{13}C HSQC (600.1 MHz, CD_2Cl_2): δ = 5.79 (m, *3*H, C^{13}*H*, C^{10}*H* and C^{11}*H*), 5.12 (s, 2H, C^{9}*H*), 4.27 (s, 2H, C^{8}*H*), 7.82–6.85 ppm (55H, other aromatic protons and C^{12}*H*). ^{31}P NMR (242.9 MHz, CD_2Cl_2): δ = 4.90 (t, *J* = 5.79 Hz, C*P*Ph3), -0.29 ppm (s, Os*P*Ph3). ^{13}C NMR plus DEPT-135, ^{1}H-^{13}C HSQC and ^{1}H-^{13}C HMBC (150.9 MHz, CD_2Cl_2): δ = 316.0 (dt, apparent q, $J_{P\text{-}C}$ = 12.94 Hz, $J_{P\text{-}C}$ = 12.94 Hz, C^{1}), 217.4 (t, $J_{P\text{-}C}$ = 9.84 Hz, C^{7}), 172.4 (s, C^{5}), 169.2 (s, C^{6}), 167.7 (d, $J_{P\text{-}C}$ = 22.43 Hz, C^{4}), 159.7 (d, $J_{P\text{-}C}$ = 14.85 Hz, C^{3}), 73.64 (s, C^{8}), 70.69 (s, C^{9}), 143.0–119.8 ppm (other aromatic carbons). Elemental analysis calcd (%) for $C_{79}H_{62}BrCl_2OOsP_3$: C 64.93, H 4.28; found: C 64.59, H 4.66. HRMS (ESI): *m/z* calcd for $[C_{79}H_{62}BrClOOsP_3]^+$, 1425.2475; found, 1425.2812.

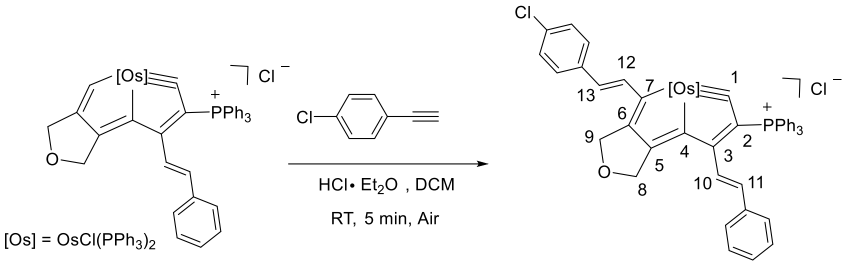

Preparation of complex 6: The excessive proton acid HCl • Et_2O (2 M in ether) solution (2.5 mL) was added to the dichloromethane solution (25 mL) of **1a** (0.50 g, 0.39 mmol) and 4-Chlorophenylacetylene (0.50 mmol) under air without using standard Schlenk techniques. The reaction mixture was stirred at room temperature for 5 min to yield a magenta solution. The solution was evaporated under vacuum to a volume of approximately 5 mL and then washed with Et_2O (1 × 100 mL) to afford a magenta solid. The solid was purified by flash chromatography on silica gel (eluent: 20:1 dichloromethane/methanol) to yield complex **6** as a magenta solid. Yield: 0.54 g, 98%. ^{1}H NMR plus ^{1}H-^{13}C HSQC (600.1 MHz, CD_2Cl_2): δ = 5.79 (m, 3H, $C^{13}H$, $C^{10}H$ and $C^{11}H$), 5.13 (s, 2H, C^9H), 4.28 (s, 2H, C^8H), 7.85–6.86 ppm (55H, other aromatic protons and $C^{12}H$). ^{31}P NMR (242.9 MHz, CD_2Cl_2): δ = 4.90 (t, J = 5.89 Hz, C*P*Ph3), -0.27 ppm (s, Os*P*Ph3). ^{13}C NMR plus DEPT-135, ^{1}H-^{13}C HSQC and ^{1}H-^{13}C HMBC (150.9 MHz, CD_2Cl_2): δ = 316.0 (dt, apparent q, $J_{P\text{-}C}$ = 14.73 Hz, $J_{P\text{-}C}$ = 14.73 Hz, C^1), 217.4 (t, $J_{P\text{-}C}$ = 10.40 Hz, C^7), 172.4 (s, C^5), 169.2 (s, C^6), 167.7 (d, $J_{P\text{-}C}$ = 22.72 Hz, C^4), 159.6 (d, $J_{P\text{-}C}$ = 15.15 Hz, C^3), 73.64 (s, C^8), 70.69 (s, C^9), 143.0–119.8 ppm (other aromatic carbons). Elemental analysis calcd (%) for $C_{79}H_{62}Cl_3OOsP_3$: C 66.97, H 4.41; found: C 66.69, H 4.08. HRMS (ESI): *m/z* calcd for $[C_{79}H_{62}Cl_2OOsP_3]^+$, 1381.2988; found, 1381.3183.

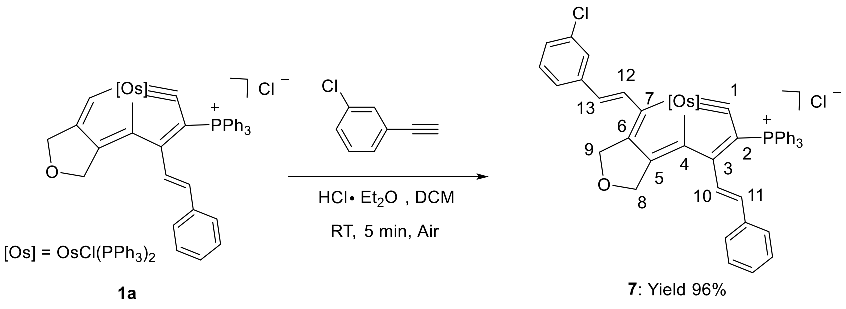

Preparation of complex 7: The excessive proton acid HCl • Et_2O (2 M in ether) solution (2.5 mL) was added to the dichloromethane solution (25 mL) of **1a** (0.50 g, 0.39 mmol) and 3-Chlorophenylacetylene (0.50 mmol) under air without using standard Schlenk techniques. The reaction mixture was stirred at room temperature for 5 min to yield a magenta solution. The solution was evaporated under vacuum to a volume of approximately 5 mL and then washed with Et_2O (1×100 mL) to afford a magenta solid. The solid was purified by flash chromatography on silica gel (eluent: 20:1 dichloromethane/methanol) to yield complex **7** as a magenta solid. Yield: 0.53 g, 96%. ^{1}H NMR plus ^{1}H-^{13}C HSQC (600.1 MHz, CD_2Cl_2): δ = 5.78 (q, J = 16.14 Hz, J = 16.14 Hz, 2H, $C^{10}H$ and $C^{11}H$), 5.72 (d, J = 16.58 Hz, 1H, $C^{13}H$), 5.11 (s, 2H, $C^{9}H$), 4.30 (s, 2H, $C^{8}H$), 7.86–6.86 ppm (55H, other aromatic protons and $C^{12}H$). ^{31}P NMR (242.9 MHz, CD_2Cl_2): δ = 4.93 (t, J = 5.71 Hz, C*P*Ph_3), -0.66 ppm (d, J = 6.03 Hz, Os*P*Ph_3). ^{13}C NMR plus DEPT-135, ^{1}H-^{13}C HSQC and ^{1}H-^{13}C HMBC (150.9 MHz, CD_2Cl_2): δ = 316.0 (dt, apparent q, $J_{P\text{-}C}$ = 12.78 Hz, $J_{P\text{-}C}$ = 12.78 Hz, C^{1}), 216.8 (t, $J_{P\text{-}C}$ = 10.38 Hz, C^{7}), 172.4 (s, C^{5}), 169.4 (s, C^{6}), 167.9 (d, $J_{P\text{-}C}$ = 20.83 Hz, C^{4}), 160.1 (d, $J_{P\text{-}C}$ = 14.81 Hz, C^{3}), 73.70 (s, C^{8}), 70.62 (s, C^{9}), 143.6–119.7 ppm (other aromatic carbons). Elemental analysis calcd (%) for $C_{79}H_{62}Cl_3OOsP_3$: C 66.97, H 4.41; found: C 67.12, H 4.72. HRMS (ESI): *m/z* calcd for $[C_{79}H_{62}Cl_2OOsP_3]^+$, 1381.2988; found, 1381.3193.

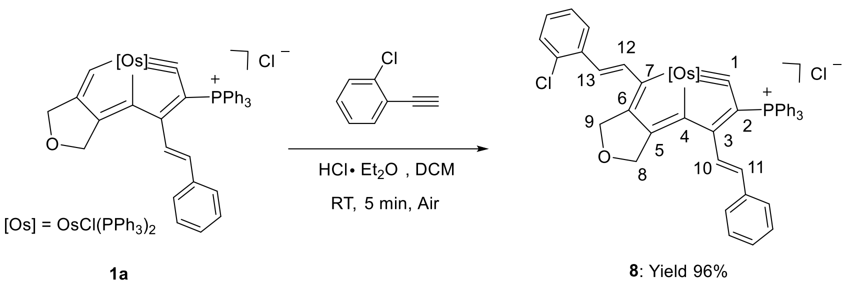

Preparation of complex 8: The excessive proton acid HCl • Et_2O (2 M in ether) solution (2.5 mL) was added to the dichloromethane solution (25 mL) of **1a** (0.50 g, 0.39 mmol) and 2-Chlorophenylacetylene (0.50 mmol) under air without using standard Schlenk techniques. The reaction mixture was stirred at room temperature for 5 min to yield a magenta solution. The solution was evaporated under vacuum to a volume of approximately 5 mL and then washed with Et_2O (1 × 100 mL) to afford a magenta solid. The solid was purified by flash chromatography on silica gel (eluent: 20:1 dichloromethane/methanol) to yield complex **8** as a magenta solid. Yield: 0.53 g, 96%. ^{1}H NMR plus ^{1}H-^{13}C HSQC (600.1 MHz, CD_2Cl_2): δ = 6.27 (d, J = 16.24 Hz, 1H, $C^{13}H$), 5.80 (q, J = 16.51 Hz, J = 16.51 Hz, 2H, $C^{10}H$ and $C^{11}H$), 5.18 (s, 2H, C^9H), 4.29 (s, 2H, C^8H), 7.86–6.87 ppm (55H, other aromatic protons and $C^{12}H$). ^{31}P NMR (242.9 MHz, CD_2Cl_2): δ = 4.92 (t, J = 5.94 Hz, C*P*Ph$_3$), -0.20 ppm (d, J = 5.82 Hz, Os*P*Ph$_3$). ^{13}C NMR plus DEPT-135, ^{1}H-^{13}C HSQC and ^{1}H-^{13}C HMBC (150.9 MHz, CD_2Cl_2): δ = 316.0 (dt, apparent q, $J_{P\text{-}C}$ = 14.20 Hz, $J_{P\text{-}C}$ = 14.20 Hz, C^1), 217.2 (t, $J_{P\text{-}C}$ = 9.80 Hz, C^7), 172.5 (s, C^5), 169.4 (s, C^6), 167.9 (d, $J_{P\text{-}C}$ = 21.55 Hz, C^4), 159.9 (d, $J_{P\text{-}C}$ = 14.54 Hz, C^3), 73.69 (s, C^8), 70.87 (s, C^9), 143.8–119.7 ppm (other aromatic carbons). Elemental analysis calcd (%) for $C_{79}H_{62}Cl_3OOsP_3$: C 66.97, H 4.41; found: C 67.22, H 4.89. HRMS (ESI): *m/z* calcd for $[C_{79}H_{62}Cl_2OOsP_3]^+$, 1381.2988; found, 1381.3187.

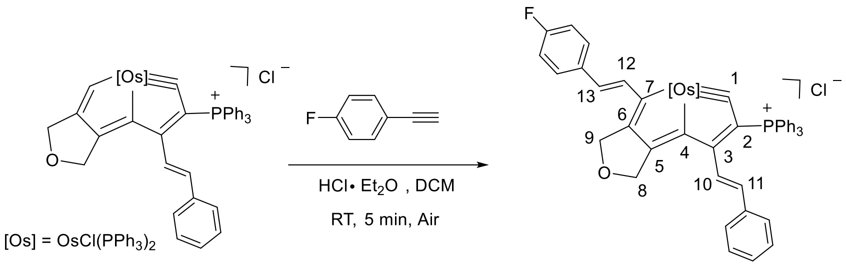

Preparation of complex 9: The excessive proton acid HCl • Et_2O (2 M in ether) solution (2.5 mL) was added to the dichloromethane solution (25 mL) of **1a** (0.50 g, 0.39 mmol) and 4-Fluorophenylacetylene (0.50 mmol) under air without using standard Schlenk techniques. The reaction mixture was stirred at room temperature for 5 min to yield a purple solution. The solution was evaporated under vacuum to a volume of approximately 5 mL and then washed with Et_2O (1 × 100 mL) to afford a purple solid. The solid was purified by flash chromatography on silica gel (eluent: 20:1 dichloromethane/methanol) to yield complex **9** as a purple solid. Yield: 0.53 g, 96%. ^{1}H NMR plus ^{1}H-^{13}C HSQC (600.1 MHz, CD_2Cl_2): δ = 5.82 (d, J = 16.38 Hz, 1H, $C^{13}H$), 5.79 (q, J = 16.49 Hz, J = 16.49 Hz, 2H, $C^{10}H$ and $C^{11}H$), 5.13 (s, 2H, C^9H), 4.27 (s, 2H, C^8H), 7.84–6.84 ppm (55H, other aromatic protons and $C^{12}H$). ^{31}P NMR (242.9 MHz, CD_2Cl_2): δ = 4.85 (s, C*P*Ph$_3$), -0.17 ppm (s, Os*P*Ph$_3$). ^{13}C NMR plus DEPT-135, ^{1}H-^{13}C HSQC and ^{1}H-^{13}C HMBC (150.9 MHz, CD_2Cl_2): δ = 316.1 (dt, apparent q, $J_{P\text{-}C}$ = 14.25 Hz, $J_{P\text{-}C}$ = 14.25 Hz, C^1), 218.3 (t, $J_{P\text{-}C}$ = 10.66 Hz, C^7), 172.5 (s, C^5), 168.9 (s, C^6), 167.4 (d, $J_{P\text{-}C}$ = 22.24 Hz, C^4), 159.2 (d, $J_{P\text{-}C}$ = 14.37 Hz, C^3), 73.62 (s, C^8), 70.69 (s, C^9), 163.2–115.9 ppm (other aromatic carbons). Elemental analysis calcd (%) for $C_{79}H_{62}Cl_2FOOsP_3$: C 67.76, H 4.46; found: C 67.99, H 4.89. HRMS (ESI): *m/z* calcd for $[C_{79}H_{62}ClFOOsP_3]^+$, 1365.3293; found, 1365.3511.

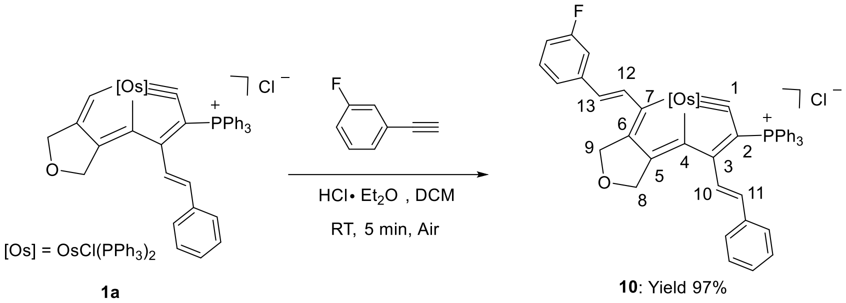

Preparation of complex 10: The excessive proton acid HCl • Et_2O (2 M in ether) solution (2.5 mL) was added to the dichloromethane solution (25 mL) of **1a** (0.50 g, 0.39 mmol) and 3-Fluorophenylacetylene (0.50 mmol) under air without using standard Schlenk techniques. The reaction mixture was stirred at room temperature for 5 min to yield a magenta solution. The solution was evaporated under vacuum to a volume of approximately 5 mL and then washed with Et_2O (1 × 100 mL) to afford a magenta solid. The solid was purified by flash chromatography on silica gel (eluent: 20:1 dichloromethane/methanol) to yield complex **10** as a magenta solid. Yield: 0.53 g, 97%. ^{1}H NMR plus ^{1}H-^{13}C HSQC (600.1 MHz, CD_2Cl_2): δ = 5.78 (d, J = 17.03 Hz, 2H, $C^{10}H$ and $C^{11}H$), 5.75 (d, J = 15.21 Hz, 1H, $C^{13}H$), 5.12 (s, 2H, $C^{9}H$), 4.28 (s, 2H, $C^{8}H$), 7.85–6.80 ppm (55H, other aromatic protons and $C^{12}H$). ^{31}P NMR (242.9 MHz, CD_2Cl_2): δ = 4.92 (t, J = 6.08 Hz, C*P*Ph3), -0.57 ppm (d, J = 5.80 Hz, Os*P*Ph3). ^{13}C NMR plus DEPT-135, ^{1}H-^{13}C HSQC and ^{1}H-^{13}C HMBC (150.9 MHz, CD_2Cl_2): δ = 316.0 (dt, apparent q, J_{P-C} = 14.05 Hz, J_{P-C} = 14.05 Hz, C^1), 217.0 (t, J_{P-C} = 9.51 Hz, C^7), 172.4 (s, C^5), 169.4 (s, C^6), 167.9 (d, J_{P-C} = 21.85 Hz, C^4), 160.0 (d, J_{P-C} = 15.37 Hz, C^3), 73.67 (s, C^8), 70.63 (s, C^9), 164.1–113.3 ppm (other aromatic carbons). Elemental analysis calcd (%) for $C_{79}H_{62}Cl_2FOOsP_3$: C 67.76, H 4.46; found: C 67.47, H 4.17. HRMS (ESI): *m/z* calcd for $[C_{79}H_{62}ClFOOsP_3]^+$, 1365.3293; found, 1365.3510.

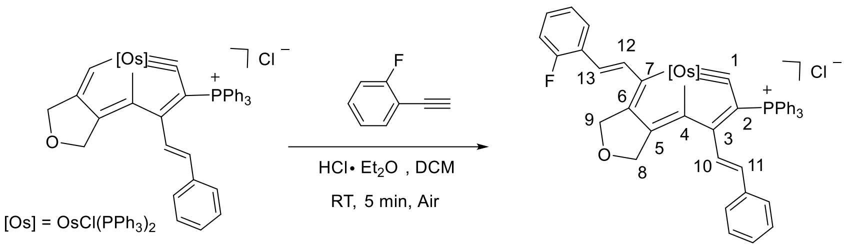

1a **11**: Yield 98%

Preparation of complex 11: The excessive proton acid HCl • Et_2O (2 M in ether) solution (2.5 mL) was added to the dichloromethane solution (25 mL) of **1a** (0.50 g, 0.39 mmol) and 2-Fluorophenylacetylene (0.50 mmol) under air without using standard Schlenk techniques. The reaction mixture was stirred at room temperature for 5 min to yield a magenta solution. The solution was evaporated under vacuum to a volume of approximately 5 mL and then washed with Et_2O (1×100 mL) to afford a magenta solid. The solid was purified by flash chromatography on silica gel (eluent: 20:1 dichloromethane/methanol) to yield complex **11** as a magenta solid. Yield: 0.54 g, 98%. ^{1}H NMR plus ^{1}H-^{13}C HSQC (600.1 MHz, CD_2Cl_2): δ = 6.06 (d, J = 16.62 Hz, 1H, $C^{13}H$), 5.79 (q, J = 17.13Hz, J = 17.13 Hz, 2H, $C^{10}H$ and $C^{11}H$), 5.14 (s, 2H, C^9H), 4.27 (s, 2H, C^8H), 7.85–6.86 ppm (55H, other aromatic protons and $C^{12}H$). ^{31}P NMR (242.9 MHz, CD_2Cl_2): δ = 4.89 (t, J = 5.92 Hz, C*P*Ph$_3$), -0.31 ppm (s, Os*P*Ph$_3$). ^{13}C NMR plus DEPT-135, ^{1}H-^{13}C HSQC and ^{1}H-^{13}C HMBC (150.9 MHz, CD_2Cl_2): δ = 316.0 (dt, apparent q, $J_{P\text{-}C}$ = 14.75 Hz, $J_{P\text{-}C}$ = 14.75 Hz, C^1), 217.6 (t, $J_{P\text{-}C}$ = 9.26 Hz, C^7), 172.6 (s, C^5), 169.3 (s, C^6), 167.8 (d, $J_{P\text{-}C}$ = 22.26 Hz, C^4), 159.7 (d, $J_{P\text{-}C}$ = 16.06 Hz, C^3), 73.65 (s, C^8), 70.66 (s, C^9), 161.2–115.8 ppm (other aromatic carbons). Elemental analysis calcd (%) for $C_{79}H_{62}Cl_2FOOsP_3$: C 67.76, H 4.46; found: C 67.88, H 4.85. HRMS (ESI): *m/z* calcd for $[C_{79}H_{62}ClFOOsP_3]^+$, 1365.3293; found, 1365.3293.

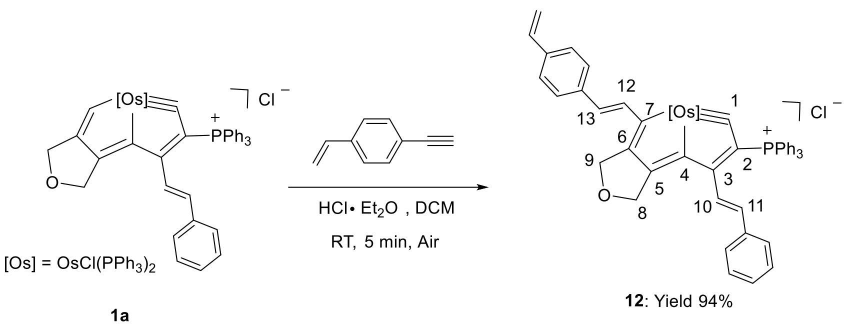

Preparation of complex 12: The excessive proton acid HCl • Et_2O (2 M in ether) solution (2.5 mL) was added to the dichloromethane solution (25 mL) of **1a** (0.50 g, 0.39 mmol) and 1-ethenyl-4-ethynylbenzene (0.50 mmol) under air without using standard Schlenk techniques. The reaction mixture was stirred at room temperature for 5 min to yield a dark purple solution. The solution was evaporated under vacuum to a volume of approximately 5 mL and then washed with Et_2O (1 × 100 mL) to afford a dark purple solid. The solid was purified by flash chromatography on silica gel (eluent: 20:1 dichloromethane/methanol) to yield complex **12** as a dark purple solid. Yield: 0.51 g, 94%. ^{1}H NMR plus ^{1}H-^{13}C HSQC (600.1 MHz, CD_2Cl_2): δ = 5.85 (m, 4H, $C^{13}H$, $C^{15}H$, $C^{10}H$ and $C^{11}H$), 5.37 (s, 2H, $C^{16}H$), 5.13 (s, 2H, C^9H), 4.26 (s, 2H, C^8H), 7.85–6.86 ppm (55H, other aromatic protons and $C^{12}H$). ^{31}P NMR (242.9 MHz, CD_2Cl_2): δ = 4.83 (t, J = 5.92 Hz, C*P*Ph_3), -0.19 ppm (d, J = 5.79 Hz, Os*P*Ph_3). ^{13}C NMR plus DEPT-135, ^{1}H-^{13}C HSQC and ^{1}H-^{13}C HMBC (150.9 MHz, CD_2Cl_2): δ = 316.2 (dt, apparent q, J_{P-C} = 14.63 Hz, J_{P-C} = 14.63 Hz, C^1), 218.4 (t, J_{P-C} = 10.62 Hz, C^7), 172.4 (s, C^5), 169.0 (s, C^6), 167.5 (d, J_{P-C} = 22.37 Hz, C^4), 158.9 (d, J_{P-C} = 14.62 Hz, C^3), 73.58 (s, C^8), 70.76 (s, C^9), 142.7–114.2 ppm (other aromatic carbons). Elemental analysis calcd (%) for $C_{81}H_{65}Cl_2OOsP_3$: C 69.07, H 4.65; found: C 69.37, H 4.81. HRMS (ESI): *m/z* calcd for $[C_{81}H_{65}ClOOsP_3]^+$, 1373.3544; found, 1373.3725.

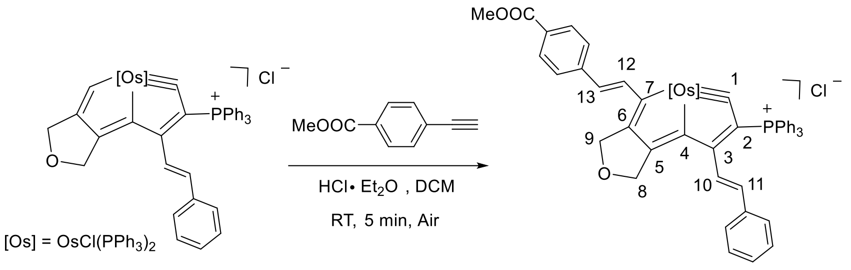

1a

13: Yield 96%

Preparation of complex 13: The excessive proton acid HCl • Et_2O (2 M in ether) solution (2.5 mL) was added to the dichloromethane solution (25 mL) of **1a** (0.50 g, 0.39 mmol) and 4-Ethynylbenzoic acid methyl ester (0.50 mmol) under air without using standard Schlenk techniques. The reaction mixture was stirred at room temperature for 5 min to yield a magenta solution. The solution was evaporated under vacuum to a volume of approximately 5 mL and then washed with Et_2O (1 × 100 mL) to afford a magenta solid. The solid was purified by flash chromatography on silica gel (eluent: 20:1 dichloromethane/methanol) to yield complex **13** as a magenta solid. Yield: 0.54 g, 96%. ^{1}H NMR plus ^{1}H-^{13}C HSQC (600.1 MHz, CD_2Cl_2): δ = 5.81 (m, 3H, C^{13}*H*, C^{10}*H* and C^{11}*H*), 5.15 (s, 2H, C^9*H*), 4.29 (s, 2H, C^8*H*), 3.94 (s, 3H, COOCH_3), 7.95–6.88 ppm (55H, other aromatic protons and C^{12}*H*). ^{31}P NMR (242.9 MHz, CD_2Cl_2): δ = 4.94 (d, *J* = 6.09 Hz, C*P*Ph$_3$), -0.50 ppm (s, Os*P*Ph$_3$). ^{13}C NMR plus DEPT-135, ^{1}H-^{13}C HSQC and ^{1}H-^{13}C HMBC (150.9 MHz, CD_2Cl_2): δ = 315.9 (dt, apparent q, $J_{P\text{-}C}$ = 12.31 Hz, $J_{P\text{-}C}$ = 12.31 Hz, C^1), 216.2 (t, $J_{P\text{-}C}$ = 10.81 Hz, C^7), 172.3 (s, C^5), 169.6 (s, C^6), 168.2 (d, $J_{P\text{-}C}$ = 23.10 Hz, C^4), 160.5 (d, $J_{P\text{-}C}$ = 14.59 Hz, C^3), 73.74 (s, C^8), 70.73 (s, C^9), 166.7–119.7 ppm (other aromatic carbons). Elemental analysis calcd (%) for $C_{81}H_{65}Cl_2O_3OsP_3$: C 67.54, H 4.55; found: C 67.25, H 4.84. HRMS (ESI): *m/z* calcd for $[C_{81}H_{65}ClO_3OsP_3]^+$, 1405.3442; found, 1405.3630.

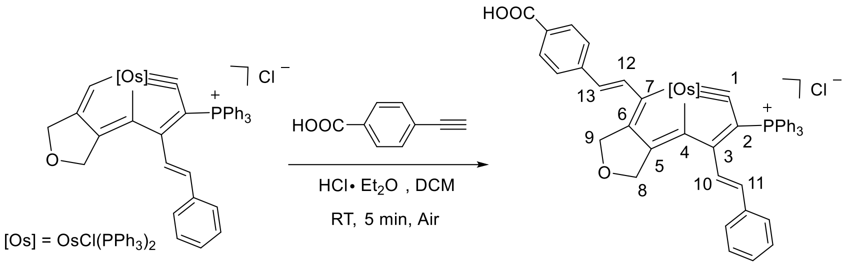

1a

14: Yield 94%

Preparation of complex 14: The excessive proton acid HCl • Et_2O (2 M in ether) solution (2.5 mL) was added to the dichloromethane solution (25 mL) of **1a** (0.50 g, 0.39 mmol) and 4-Eethynylbenzoic acid (0.50 mmol) under air without using standard Schlenk techniques. The reaction mixture was stirred at room temperature for 5 min to yield a magenta solution. The solution was evaporated under vacuum to a volume of approximately 5 mL and then washed with Et_2O (1× 100 mL) to afford a magenta solid. The solid was purified by flash chromatography on silica gel (eluent: 20:1 dichloromethane/methanol) to yield complex **14** as a magenta solid. Yield: 0.52 g, 94%. ^{1}H NMR plus ^{1}H-^{13}C HSQC (600.1 MHz, CD_2Cl_2): δ = 7.42 (d, *J* = 16.45 Hz, 1H, $C^{12}H$), 5.84 (d, *J* = 16.43 Hz, 1H, $C^{13}H$)， 5.78 (s, 2H, $C^{10}H$ and $C^{11}H$), 5.14 (s, 2H, $C^{9}H$), 4.26 (s, 2H, $C^{8}H$), 7.95–6.84 ppm (55H, other aromatic protons and -COO*H*). ^{31}P NMR (242.9 MHz, CD_2Cl_2): δ = 4.94 (t, *J* = 6.40 Hz, C*P*Ph3), -0.50 ppm (s, Os*P*Ph3). ^{13}C NMR plus DEPT-135, ^{1}H-^{13}C HSQC and ^{1}H-^{13}C HMBC (150.9 MHz, CD_2Cl_2): δ = 315.9 (m, C^1), 216.8 (s, C^7), 172.1 (s, C^5), 169.4 (s, C^6), 168.3 (s, *C*OOH), 168.2 (d, $J_{P\text{-}C}$ = 21.85 Hz, C^4), 160.2 (d, $J_{P\text{-}C}$ = 14.12 Hz, C^3), 73.62 (s, C^8), 70.65 (s, C^9), 144.3–119.7 ppm (other aromatic carbons). Elemental analysis calcd (%) for $C_{80}H_{63}Cl_2O_3OsP_3$: C 67.36, H 4.45; found: C 67.65, H 4.69. HRMS (ESI): *m/z* calcd for $[C_{80}H_{63}ClO_3OsP_3]^+$, 1391.3286; found, 1391.3511.

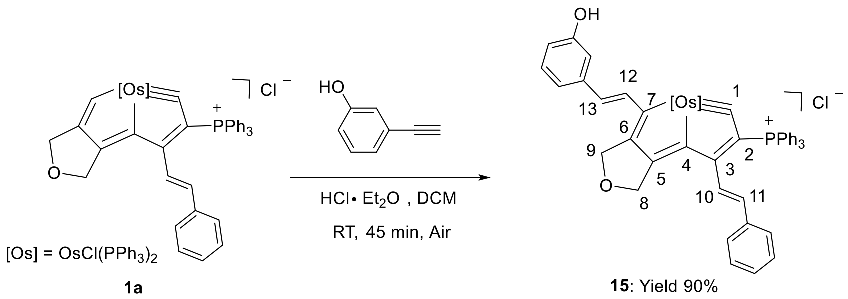

Preparation of complex 15: The excessive proton acid HCl • Et_2O (2 M in ether) solution (2.5 mL) was added to the dichloromethane solution (25 mL) of **1a** (0.50 g, 0.39 mmol) and 3-Hydroxyphenylacetylene (0.50 mmol) under air. The reaction mixture was stirred at room temperature for 5 min to yield a magenta solution. The solution was evaporated under vacuum to a volume of approximately 5 mL and then washed with Et_2O (1 × 100 mL) to afford a magenta solid. The solid was purified by flash chromatography on silica gel (eluent: 20:1 dichloromethane/methanol) to yield complex **15** as a magenta solid. Yield: 0.49 g, 90%. ^{1}H NMR plus ^{1}H-^{13}C HSQC (600.1 MHz, CD_2Cl_2): δ = 9.48 (br, 1H, O*H*), 5.89 (d, *J* = 16.31 Hz, 1H, $C^{13}H$), 5.76 (q, *J* = 16.87 Hz, *J* = 16.87 Hz, 2H, $C^{10}H$ and $C^{11}H$), 5.12 (s, 2H, C^9H), 4.20 (s, 2H, C^8H), 7.83–6.88 ppm (55H, other aromatic protons and $C^{12}H$). ^{31}P NMR (242.9 MHz, CD_2Cl_2): δ = 4.64 (t, *J* = 5.92 Hz, C*P*Ph_3), -0.05 ppm (s, Os*P*Ph_3). ^{13}C NMR plus DEPT-135, ^{1}H-^{13}C HSQC and ^{1}H-^{13}C HMBC (150.9 MHz, CD_2Cl_2): δ = 316.2 (d, $J_{P\text{-}C}$ = 14.56 Hz, C^1), 220.5 (t, $J_{P\text{-}C}$ = 10.56 Hz, C^7), 172.6 (s, C^5), 169.0 (s, C^6), 167.3 (d, $J_{P\text{-}C}$ = 21.43 Hz, C^4), 157.6 (d, $J_{P\text{-}C}$ = 14.40 Hz, C^3), 73.47 (s, C^8), 70.82 (s, C^9), 158.6–116.6 ppm (other aromatic carbons). Elemental analysis calcd (%) for $C_{79}H_{63}Cl_2O_2OsP_3$: C 67.85, H 4.54; found: C 67.49, H 4.25. HRMS (ESI): *m/z* calcd for $[C_{79}H_{63}ClO_2OsP_3]^+$, 1363.3336; found, 1363.3524.

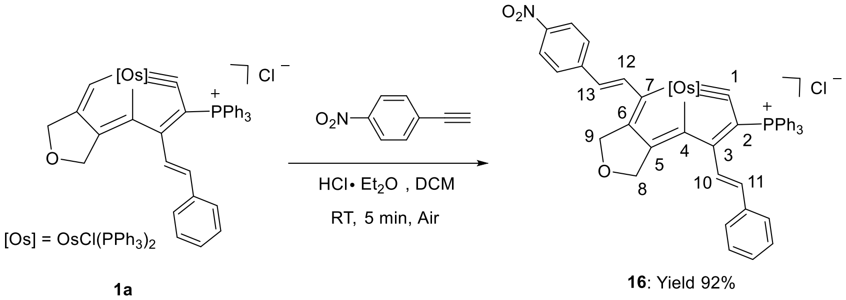

Preparation of complex 16: The excessive proton acid HCl • Et_2O (2 M in ether) solution (2.5 mL) was added to the dichloromethane solution (25 mL) of **1a** (0.50 g, 0.39 mmol) and 4-Nitroethynylbenzene (0.50 mmol) under air without using standard Schlenk techniques. The reaction mixture was stirred at room temperature for 5 min to yield a dark purple solution. The solution was evaporated under vacuum to a volume of approximately 5 mL and then washed with Et_2O (1 × 100 mL) to afford a dark purple solid. The solid was purified by flash chromatography on silica gel (eluent: 20:1 dichloromethane/methanol) to yield complex **16** as a dark purple solid. Yield: 0.51 g, 92%. ^{1}H NMR plus ^{1}H-^{13}C HSQC (600.1 MHz, CD_2Cl_2): δ = 5.80 (m, 3H, $C^{13}H$, $C^{10}H$ and $C^{11}H$), 5.15 (s, 2H, C^9H), 4.33 (s, 2H, C^8H), 8.13–6.87 ppm (55H, other aromatic protons and $C^{12}H$). ^{31}P NMR (242.9 MHz, CD_2Cl_2): δ = 5.06 (d, J = 6.80 Hz, C*P*Ph3), -0.80 ppm (d, J = 5.91 Hz, Os*P*Ph3). ^{13}C NMR plus DEPT-135, ^{1}H-^{13}C HSQC and ^{1}H-^{13}C HMBC (150.9 MHz, CD_2Cl_2): δ = 315.7 (dt, apparent q, $J_{P\text{-}C}$ = 14.19 Hz, $J_{P\text{-}C}$ = 14.19 Hz, C^1), 214.0 (s, C^7), 172.3 (s, C^5), 170.1 (s, C^6), 168.8 (d, $J_{P\text{-}C}$ = 21.84 Hz, C^4), 161.7 (d, $J_{P\text{-}C}$ = 14.95 Hz, C^3), 73.77 (s, C^8), 70.63 (s, C^9), 146.4–119.5 ppm (other aromatic carbons). Elemental analysis calcd (%) for $C_{79}H_{62}Cl_2NO_3OsP_3$: C 66.47, H 4.38, N 0.98; found: C 66.08, H 4.00, N 1.12. HRMS (ESI): *m/z* calcd for $[C_{79}H_{62}ClNO_3OsP_3]^+$, 1392.3238; found, 1392.3466.

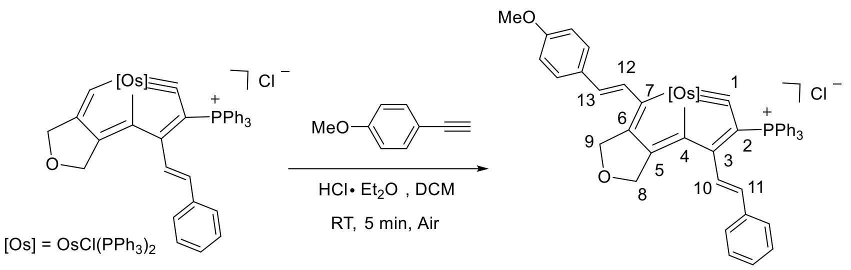

1a **17**: Yield 94%

Preparation of complex 17: The excessive proton acid HCl • Et_2O (2 M in ether) solution (2.5 mL) was added to the dichloromethane solution (25 mL) of **1a** (0.50 g, 0.39 mmol) and 4-Ethynylanisole (0.50 mmol) under air without using standard Schlenk techniques. The reaction mixture was stirred at room temperature for 5 min to yield a magenta solution. The solution was evaporated under vacuum to a volume of approximately 5 mL and then washed with Et_2O (1 × 100 mL) to afford a magenta solid. The solid was purified by flash chromatography on silica gel (eluent: 20:1 dichloromethane/methanol) to yield complex **17** as a magenta solid. Yield: 0.52 g, 94%. ^{1}H NMR plus ^{1}H-^{13}C HSQC (600.1 MHz, CD_2Cl_2): δ = 5.88 (d, J = 16.20 Hz, 1H, $C^{13}H$), 5.78 (q, J = 16.62 Hz, J = 16.62 Hz, 2H, $C^{10}H$ and $C^{11}H$), 5.12 (s, 2H, C^9H), 4.25 (s, 2H, C^8H), 3.87 (s, 3H, OCH_3), 7.85–6.86 ppm (55H, other aromatic protons and $C^{12}H$). ^{31}P NMR (242.9 MHz, CD_2Cl_2): δ = 4.66 (t, J = 5.93 Hz, $CPPh_3$), 0.24 ppm (d, J = 5.99 Hz, $OsPPh_3$). ^{13}C NMR plus DEPT-135, ^{1}H-^{13}C HSQC and ^{1}H-^{13}C HMBC (150.9 MHz, CD_2Cl_2): δ = 316.4 (dt, apparent q, $J_{P\text{-}C}$ = 12.70 Hz, $J_{P\text{-}C}$ = 12.70 Hz, C^1), 220.0 (t, $J_{P\text{-}C}$ = 9.04 Hz, C^7), 172.4 (s, C^5), 168.3 (s, C^6), 166.8 (d, $J_{P\text{-}C}$ = 21.94 Hz, C^4), 157.6 (d, $J_{P\text{-}C}$ = 15.25 Hz, C^3), 73.51 (s, C^8), 70.80 (s, C^9), 55.40 (s, OCH_3), 160.2–114.5 ppm (other aromatic carbons). Elemental analysis calcd (%) for $C_{80}H_{65}Cl_2O_2OsP_3$: C 68.03, H 4.64; found: C 68.41, H 4.98. HRMS (ESI): *m/z* calcd for $[C_{80}H_{65}ClO_2OsP_3]^+$, 1377.3493; found, 1377.3712.

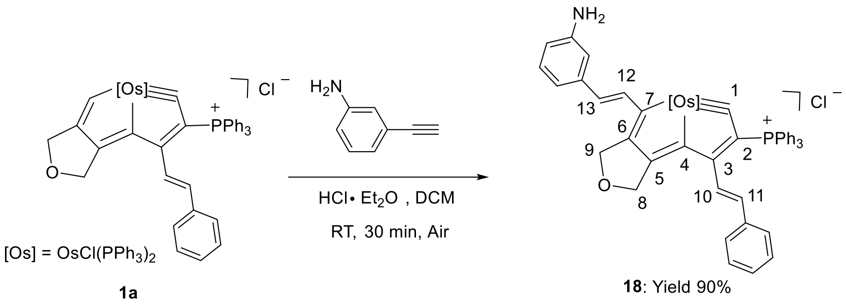

Preparation of complex 18: The excessive proton acid HCl • Et_2O (2 M in ether) solution (2.5 mL) was added to the dichloromethane solution (25 mL) of **1a** (0.50 g, 0.39 mmol) and 3-Chlorophenylacetylene (0.50 mmol) under air. The reaction mixture was stirred at room temperature for 30 min to yield a navy blue solution. The solution was evaporated under vacuum to a volume of approximately 5 mL and then washed with Et_2O (1 × 100 mL) to afford a navy blue solid. The solid was purified by flash chromatography on silica gel (eluent: 20:1 dichloromethane/methanol) to yield complex **18** as a navy blue solid. Yield: 0.49 g, 90%. ^{1}H NMR plus ^{1}H-^{13}C HSQC (600.1 MHz, CD_2Cl_2): δ = 8.62 (br, 2H, N*H*$_2$), 5.79 (d, *J* = 16.32 Hz, 1H, C^{13}*H*), 5.77 (q, *J* = 16.48 Hz, *J* = 16.48 Hz, 2H, C^{10}*H* and C^{11}*H*), 5.10 (s, 2H, C^9*H*), 4.21 (s, 2H, C^8*H*), 7.85–6.46 ppm (55H, other aromatic protons and C^{12}*H*). ^{31}P NMR (242.9 MHz, CD_2Cl_2): δ = 4.80 (t, *J* = 5.84 Hz, C*P*Ph$_3$), -0.12 ppm (s, Os*P*Ph$_3$). ^{13}C NMR plus DEPT-135, ^{1}H-^{13}C HSQC and ^{1}H-^{13}C HMBC (150.9 MHz, CD_2Cl_2): δ = 316.3 (s, C^1), 219.5 (s, C^7), 172.5 (s, C^5), 169.0 (s, C^6), 167.3 (s, C^4), 158.4 (s, C^3), 73.51 (s, C^8), 70.73 (s, C^9), 149.8–113.5 ppm (other aromatic carbons). Elemental analysis calcd (%) for $C_{79}H_{64}Cl_2NOOsP_3$: C 67.90, H 4.62, N 1.00; found: C 67.51, H 5.00, N 1.12. HRMS (ESI): *m/z* calcd for $[C_{79}H_{64}ClNOOsP_3]^+$, 1362.3496; found, 1362.3775.

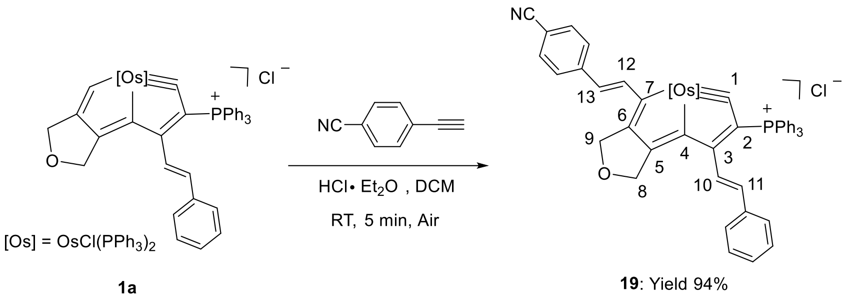

Preparation of complex 19: The excessive proton acid HCl • Et_2O (2 M in ether) solution (2.5 mL) was added to the dichloromethane solution (25 mL) of **1a** (0.50 g, 0.39 mmol) and 4-Ethynylbenzonitrile (0.50 mmol) under air without using standard Schlenk techniques. The reaction mixture was stirred at room temperature for 5 min to yield a dark purple solution. The solution was evaporated under vacuum to a volume of approximately 5 mL and then washed with Et_2O (1 × 100 mL) to afford a dark purple solid. The solid was purified by flash chromatography on silica gel (eluent: 20:1 dichloromethane/methanol) to yield complex **19** as a dark purple solid. Yield: 0.51 g, 94%. ^1H NMR plus ^1H-^{13}C HSQC (600.1 MHz, CD_2Cl_2): δ = 5.80 (q, *J* = 16.31 Hz, *J* = 16.31 Hz, 2H, $C^{10}H$ and $C^{11}H$), 5.76 (d, *J* = 16.50 Hz, 1H, $C^{13}H$), 5.13 (s, 2H, C^9H), 4.30 (s, 2H, C^8H), 7.85–6.86 ppm (55H, other aromatic protons and $C^{12}H$). ^{31}P NMR (242.9 MHz, CD_2Cl_2): δ = 5.01 (d, *J* = 5.95 Hz, C*P*Ph_3), -0.65 ppm (d, *J* = 5.84 Hz, Os*P*Ph_3). ^{13}C NMR plus DEPT-135, ^1H-^{13}C HSQC and ^1H-^{13}C HMBC (150.9 MHz, CD_2Cl_2): δ = 315.7 (dt, apparent q, $J_{P\text{-}C}$ = 16.09 Hz, $J_{P\text{-}C}$ = 16.09 Hz, C^1), 214.7 (t, $J_{P\text{-}C}$ = 10.42 Hz, C^7), 172.3 (s, C^5), 169.9 (s, C^6), 168.5 (d, $J_{P\text{-}C}$ = 22.37 Hz, C^4), 161.3 (d, $J_{P\text{-}C}$ = 16.83 Hz, C^3), 73.74 (s, C^8), 70.63 (s, C^9), 145.2–110.6 ppm (other aromatic carbons). Elemental analysis calcd (%) for $C_{80}H_{62}Cl_2NOOsP_3$: C 68.27, H 4.44, N 1.00; found: C 68.65, H 4.06, N 1.25. HRMS (ESI): *m/z* calcd for $[C_{80}H_{62}ClNOOsP_3]^+$, 1372.3340; found, 1372.3562.

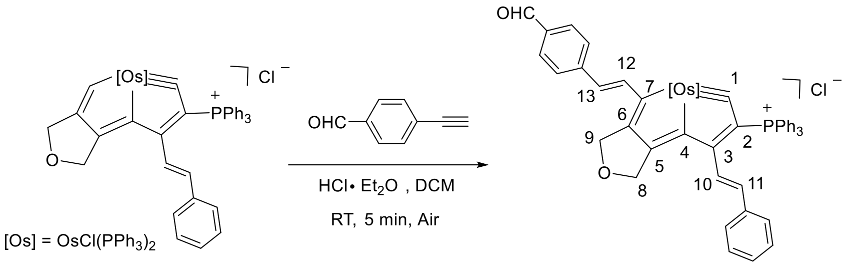

1a **20**: Yield 95%

Preparation of complex 20: The excessive proton acid HCl • Et_2O (2 M in ether) solution (2.5 mL) was added to the dichloromethane solution (25 mL) of **1a** (0.50 g, 0.39 mmol) and 4-Ethynylbenzaldehyde (0.50 mmol) under air without using standard Schlenk techniques. The reaction mixture was stirred at room temperature for 5 min to yield a dark purple solution. The solution was evaporated under vacuum to a volume of approximately 5 mL and then washed with Et_2O (1 × 100 mL) to afford a dark purples solid. The solid was purified by flash chromatography on silica gel (eluent: 20:1 dichloromethane/methanol) to yield complex **20** as a dark purple solid. Yield: 0.52 g, 95%. ^1H NMR plus ^1H-^{13}C HSQC (600.1 MHz, CD_2Cl_2): δ = 10.03 (s, 1H, CHO), 5.80 (m, 3H, $C^{13}H$, $C^{10}H$ and $C^{11}H$), 5.15 (s, 2H, C^9H), 4.30 (s, 2H, C^8H), 7.85–6.86 ppm (55H, other aromatic protons and $C^{12}H$). ^{31}P NMR (242.9 MHz, CD_2Cl_2): δ = 4.99 (d, J = 5.79 Hz, C*P*Ph_3), -0.58 ppm (s, Os*P*Ph_3). ^{13}C NMR plus DEPT-135, ^1H-^{13}C HSQC and ^1H-^{13}C HMBC (150.9 MHz, CD_2Cl_2): δ = 315.8 (dt, apparent q, $J_{P\text{-}C}$ = 14.50 Hz, $J_{P\text{-}C}$ = 14.50 Hz, C^1), 215.5 (t, $J_{P\text{-}C}$ = 10.53 Hz, C^7), 172.3 (s, C^5), 169.8 (s, C^6), 168.6 (d, $J_{P\text{-}C}$ = 21.59 Hz, C^4), 160.8 (d, $J_{P\text{-}C}$ = 15.33 Hz, C^3), 73.70 (s, C^8), 70.66 (s, C^9), 191.6–119.6 ppm (other aromatic carbons). Elemental analysis calcd (%) for $C_{80}H_{63}Cl_2O_2OsP_3$: C 68.13, H 4.50; found: C 68.01, H 4.25. HRMS (ESI): *m/z* calcd for $[C_{80}H_{63}ClO_2OsP_3]^+$, 1375.3337; found, 1375.3550.

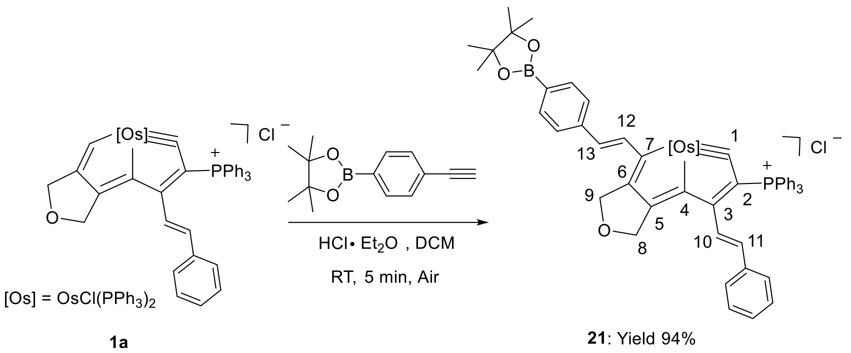

Preparation of complex 21: The excessive proton acid HCl • Et_2O (2 M in ether) solution (2.5 mL) was added to the dichloromethane solution (25 mL) of **1a** (0.50 g, 0.39 mmol) and 4-Ethynylbenzeneboronic acid pinacol ester (0.50 mmol) under air without using standard Schlenk techniques. The reaction mixture was stirred at room temperature for 5 min to yield a magenta solution. The solution was evaporated under vacuum to a volume of approximately 5 mL and then washed with Et_2O (1 × 100 mL) to afford a magenta solid. The solid was purified by flash chromatography on silica gel (eluent: 20:1 dichloromethane/methanol) to yield complex **21** as a magenta solid. Yield: 0.55 g, 94%. ^{1}H NMR plus ^{1}H-^{13}C HSQC (600.1 MHz, CD_2Cl_2): δ = 5.84 (d, J = 17.31 Hz, 1H, C^{13}*H*), 5.78 (m, 2H, C^{10}*H* and C^{11}*H*), 5.13 (s, 2H, C^9*H*), 4.26 (s, 2H, C^8*H*), 1.39 (s, 12H, CH_3), 7.91–6.86 ppm (55H, other aromatic protons and C^{12}*H*). ^{31}P NMR (242.9 MHz, CD_2Cl_2): δ = 5.08 (s, C*P*Ph$_3$), -0.18 ppm (s, Os*P*Ph$_3$). ^{13}C NMR plus DEPT-135, ^{1}H-^{13}C HSQC and ^{1}H-^{13}C HMBC (150.9 MHz, CD_2Cl_2): δ = 316.1 (dt, apparent q, $J_{P\text{-}C}$ = 13.54 Hz, $J_{P\text{-}C}$ = 13.54 Hz, C^1), 218.0 (t, $J_{P\text{-}C}$ = 10.77 Hz, C^7), 172.4 (s, C^5), 169.3 (s, C^6), 167.8 (d, $J_{P\text{-}C}$ = 21.24 Hz, C^4), 159.5 (d, $J_{P\text{-}C}$ = 14.53 Hz, C^3), 83.93, 73.60 (s, C^8), 70.73 (s, C^9), 24.72, 143.3–119.8 ppm (other aromatic carbons). Elemental analysis calcd (%) for $C_{85}H_{74}BCl_2O_3OsP_3$: C 67.68, H 4.95; found: C 67.35, H 4.60. HRMS (ESI): *m/z* calcd for $[C_{85}H_{74}BClO_3OsP_3]^+$, 1473.4251; found, 1473.4461.

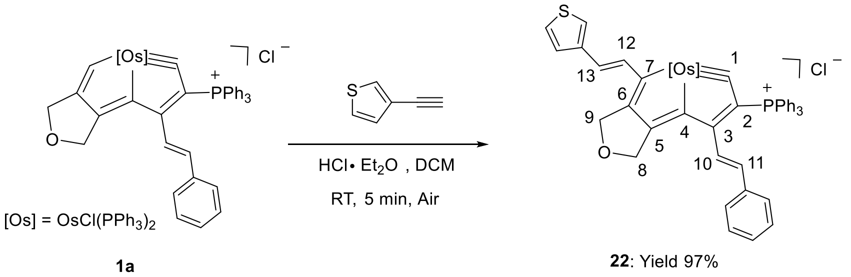

Preparation of complex 22: The excessive proton acid HCl • Et_2O (2 M in ether) solution (2.5 mL) was added to the dichloromethane solution (25 mL) of **1a** (0.50 g, 0.39 mmol) and 3-Ethynylthiophene (0.50 mmol) under air. The reaction mixture was stirred at room temperature for 5 min to yield a navy blue solution. The solution was evaporated under vacuum to a volume of approximately 5 mL and then washed with Et_2O (1× 100 mL) to afford a navy blue solid. The solid was purified by flash chromatography on silica gel (eluent: 20:1 dichloromethane/methanol) to yield complex **22** as a navy blue solid. Yield: 0.53 g, 97%. ^{1}H NMR plus ^{1}H-^{13}C HSQC (600.1 MHz, CD_2Cl_2): δ = 6.07 (d, *J* = 16.18 Hz, 1H, $C^{13}H$)，5.78 (q, *J* = 25.43 Hz, *J* = 16.69 Hz, 2H, $C^{10}H$ and $C^{11}H$), 5.09 (s, 2H, $C^{9}H$), 4.28 (s, 2H, $C^{8}H$), 7.84–6.88 ppm (54H, other aromatic protons and $C^{12}H$). ^{31}P NMR (242.9 MHz, CD_2Cl_2): δ = 4.73 (t, *J* = 5.99 Hz, C*P*Ph3), -0.28 ppm (d, *J* = 5.91 Hz, Os*P*Ph3). ^{13}C NMR plus DEPT-135, ^{1}H-^{13}C HSQC and ^{1}H-^{13}C HMBC (150.9 MHz, CD_2Cl_2): δ = 316.2 (dt, apparent q, $J_{P\text{-}C}$ = 14.25 Hz, $J_{P\text{-}C}$ = 14.25 Hz, C^1), 217.5 (t, $J_{P\text{-}C}$ = 9.09 Hz, C^7), 172.1 (s, C^5), 168.8 (s, C^6), 167.2 (d, $J_{P\text{-}C}$ = 22.17 Hz, C^4), 158.5 (d, $J_{P\text{-}C}$ = 16.37 Hz, C^3), 73.56 (s, C^8), 70.68 (s, C^9), 144.3–120.0 ppm (other aromatic carbons). Elemental analysis calcd (%) for $C_{77}H_{61}Cl_2OOsP_3S$: C 66.61, H 4.43; found: C 66.88, H 4.18. HRMS (ESI): *m/z* calcd for $[C_{77}H_{61}ClOOsP_3S]^+$, 1353.2949; found, 1353.3259.

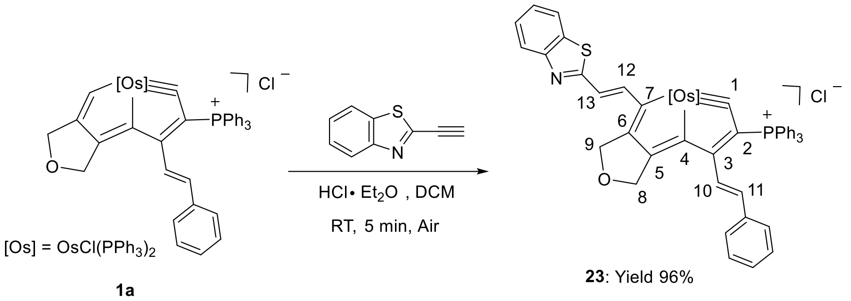

Preparation of complex 23: The excessive proton acid HCl • Et_2O (2 M in ether) solution (2.5 mL) was added to the dichloromethane solution (25 mL) of **1a** (0.50 g, 0.39 mmol) and 4-Ethynylbenzeneboronic acid pinacol ester (0.50 mmol) under air without using standard Schlenk techniques. The reaction mixture was stirred at room temperature for 5 min to yield a magenta solution. The solution was evaporated under vacuum to a volume of approximately 5 mL and then washed with Et_2O (1 × 100 mL) to afford a magenta solid. The solid was purified by flash chromatography on silica gel (eluent: 20:1 dichloromethane/methanol) to yield complex **23** as a magenta solid. Yield: 0.54 g, 96%. ^{1}H NMR plus ^{1}H-^{13}C HSQC (600.1 MHz, CD_2Cl_2): δ = 6.10 (d, J = 16.72 Hz, 1H, $C^{13}H$), 5.83 (s, 2H, $C^{10}H$ and $C^{11}H$), 5.14 (s, 2H, C^9H), 4.32 (s, 2H, C^8H), 8.00–6.87 ppm (55H, other aromatic protons and $C^{12}H$). ^{31}P NMR (242.9 MHz, CD_2Cl_2): δ = 5.19 (t, J = 5.96 Hz, CPPh$_3$), -1.26 ppm (d, J = 5.85 Hz, OsPPh$_3$). ^{13}C NMR plus DEPT-135, ^{1}H-^{13}C HSQC and ^{1}H-^{13}C HMBC (150.9 MHz, CD_2Cl_2): δ = 315.3 (dt, apparent q, $J_{P\text{-}C}$ = 13.54 Hz, $J_{P\text{-}C}$ = 13.54 Hz, C^1), 210.8 (t, $J_{P\text{-}C}$ = 10.52 Hz, C^7), 172.0 (s, C^5), 170.7 (s, C^6), 169.3 (d, $J_{P\text{-}C}$ = 22.39 Hz, C^4), 162.6 (d, $J_{P\text{-}C}$ = 14.89 Hz, C^3), 73.73 (s, C^8), 70.65 (s, C^9), 168.1–119.4 ppm (other aromatic carbons). Elemental analysis calcd (%) for $C_{80}H_{62}Cl_2NOOsP_3S$: C 66.75, H 4.34, N 0.97; found: C 66.60, H 4.01, N 1.33. HRMS (ESI): m/z calcd for $[C_{80}H_{62}ClNOOsP_3S]^+$, 1404.3058; found, 1404.3307.

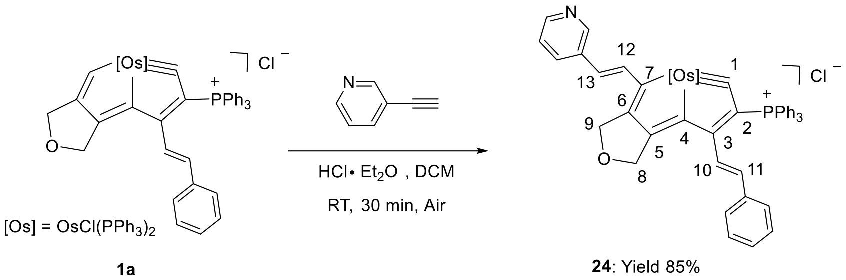

Preparation of complex 24: The excessive proton acid HCl • Et_2O (2 M in ether) solution (2.5 mL) was added to the dichloromethane solution (25 mL) of **1a** (0.50 g, 0.39 mmol) and 3-Ethynylpyridine (0.50 mmol) under air. The reaction mixture was stirred at room temperature for 30 min to yield a magenta solution. The solution was evaporated under vacuum to a volume of approximately 5 mL and then washed with Et_2O (1× 100 mL) to afford a magenta solid. The solid was purified by flash chromatography on silica gel (eluent: 20:1 dichloromethane/methanol) to yield complex **24** as a magenta solid. Yield: 0.46 g, 85%. ^{1}H NMR plus ^{1}H-^{13}C HSQC (600.1 MHz, CD_2Cl_2): δ = 5.83 (br, 3H, $C^{13}H$, $C^{10}H$ and $C^{11}H$), 5.21 (s, 2H, C^9H), 4.34 (s, 2H, C^8H), 7.89–6.90 ppm (55H, other aromatic protons and $C^{12}H$). ^{31}P NMR (242.9 MHz, CD_2Cl_2): δ = 5.05 (t, *J* = 5.84 Hz, C*P*Ph$_3$), -0.58 ppm (s, Os*P*Ph$_3$). ^{13}C NMR plus DEPT-135, ^{1}H-^{13}C HSQC and ^{1}H-^{13}C HMBC (150.9 MHz, CD_2Cl_2): δ = 315.9 (dt, apparent q, $J_{P\text{-}C}$ = 14.79 Hz, $J_{P\text{-}C}$ = 14.79 Hz, C^1), 216.3 (t, $J_{P\text{-}C}$ = 10.61 Hz, C^7), 172.5 (s, C^5), 169.5 (s, C^6), 168.0 (d, $J_{P\text{-}C}$ = 23.75 Hz, C^4), 160.5 (d, $J_{P\text{-}C}$ = 14.77 Hz, C^3), 74.35 (s, C^8), 71.18 (s, C^9), 144.2–119.8 ppm (other aromatic carbons). Elemental analysis calcd (%) for $C_{78}H_{62}Cl_2NOOsP_3$: C 67.72, H 4.52, N 1.01; found: C 67.35, H 4.88, N 1.29. HRMS (ESI): *m/z* calcd for $[C_{78}H_{62}ClNOOsP_3]^+$, 1348.3339; found, 1348.3546.

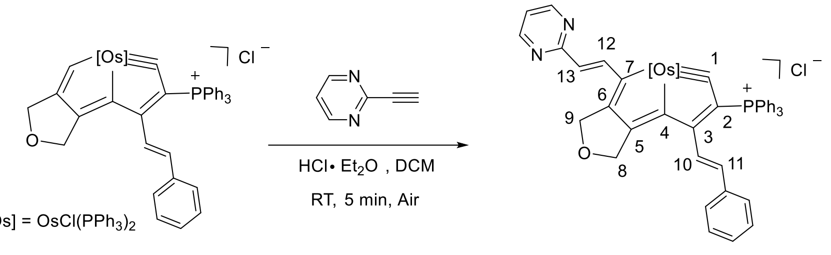

1a **25**: Yield 94%

Preparation of complex 25: The excessive proton acid HCl • Et_2O (2 M in ether) solution (2.5 mL) was added to the dichloromethane solution (25 mL) of **1a** (0.50 g, 0.39 mmol) and 2-Ethynylpyrimidine (0.50 mmol) under air without using standard Schlenk techniques. The reaction mixture was stirred at room temperature for 5 min to yield a magenta solution. The solution was evaporated under vacuum to a volume of approximately 5 mL and then washed with Et_2O (1 × 100 mL) to afford a magenta solid. The solid was purified by flash chromatography on silica gel (eluent: 20:1 dichloromethane/methanol) to yield complex **25** as a magenta solid. Yield: 0.51 g, 94%. ^{1}H NMR plus ^{1}H-^{13}C HSQC (600.1 MHz, CD_2Cl_2): δ = 8.71 (d, J = 5.03 Hz, 2H), 5.91 (q, J = 15.99 Hz, 1H, $C^{13}H$), 5.79 (q, J = 16.60 Hz, J = 16.60 Hz, 2H, $C^{10}H$ and $C^{11}H$), 5.14 (s, 2H, C^9H), 4.14 (s, 2H, C^8H), 8.05–6.86 ppm (52H, other aromatic protons and $C^{12}H$). ^{31}P NMR (242.9 MHz, CD_2Cl_2): δ = 5.16 (t, J = 5.89 Hz, C*P*Ph3), -1.05 ppm (s, Os*P*Ph3). ^{13}C NMR plus DEPT-135, ^{1}H-^{13}C HSQC and ^{1}H-^{13}C HMBC (150.9 MHz, CD_2Cl_2): δ = 315.4 (dt, apparent q, $J_{P\text{-}C}$ = 14.55 Hz, $J_{P\text{-}C}$ = 14.55 Hz, C^1), 214.7 (t, $J_{P\text{-}C}$ = 10.52 Hz, C^7), 172.3 (s, C^5), 171.2 (s, C^6), 169.9 (d, $J_{P\text{-}C}$ = 24.08 Hz, C^4), 161.4 (d, $J_{P\text{-}C}$ = 13.46 Hz, C^3), 73.53 (s, C^8), 70.63 (s, C^9), 165.1–118.0 ppm (other aromatic carbons). Elemental analysis calcd (%) for $C_{77}H_{61}Cl_2N_2OOsP_3$: C 66.80, H 4.44, N 2.02; found: C 66.51, H 4.11, N 1.88. HRMS (ESI): *m/z* calcd for $[C_{77}H_{61}ClN_2OOsP_3]^+$, 1349.3292; found, 1349.3502.

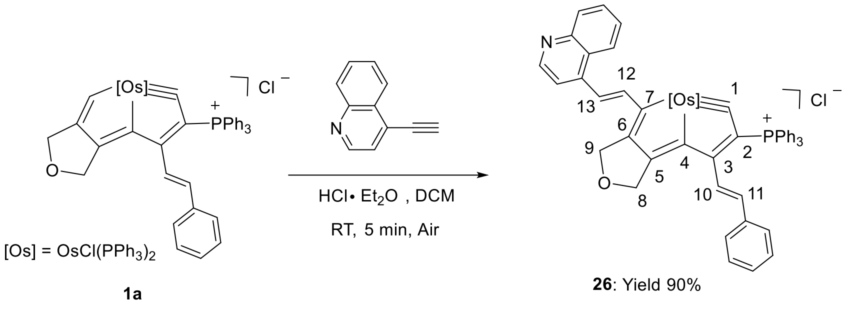

Preparation of complex 26: The excessive proton acid HCl • Et_2O (2 M in ether) solution (2.5 mL) was added to the dichloromethane solution (25 mL) of **1a** (0.50 g, 0.39 mmol) and 4-Ethynylquinoline (0.50 mmol) under air without using standard Schlenk techniques. The reaction mixture was stirred at room temperature for 5 min to yield a dark purple solution. The solution was evaporated under vacuum to a volume of approximately 5 mL and then washed with Et_2O (1 × 100 mL) to afford a dark purple solid. The solid was purified by flash chromatography on silica gel (eluent: 20:1 dichloromethane/methanol) to yield complex **26** as a dark purple solid. Yield: 0.50 g, 90%. ^{1}H NMR plus ^{1}H-^{13}C HSQC (600.1 MHz, CD_2Cl_2): δ = 6.62 (d, *J* = 17.50 Hz, 1H, $C^{13}H$), 5.84 (q, *J* = 17.02 Hz, *J* = 17.02 Hz, 2H, $C^{10}H$ and $C^{11}H$), 5.32 (s, 2H, $C^{9}H$), 4.40 (s, 2H, $C^{8}H$), 8.80–6.83 ppm (57H, other aromatic protons and $C^{12}H$). ^{31}P NMR (242.9 MHz, CD_2Cl_2): δ = 6.03 (s, C*P*Ph_3), -0.98 ppm (s, Os*P*Ph_3). ^{13}C NMR plus DEPT-135, ^{1}H-^{13}C HSQC and ^{1}H-^{13}C HMBC (150.9 MHz, CD_2Cl_2): δ = 315.4 (s, C^1), 212.3 (s, C^7), 172.1 (s, C^5), 170.8 (s, C^6), 169.3 (s, C^4), 162.6(s, C^3), 73.94 (s, C^8), 70.83 (s, C^9), 149.8–115.8 ppm (other aromatic carbons). Elemental analysis calcd (%) for $C_{82}H_{64}Cl_2NOOsP_3$: C 68.71, H 4.50, N 0.98; found: C 68.33, H 4.25, N 1.30. HRMS (ESI): *m/z* calcd for $[C_{82}H_{64}ClNOOsP_3]^+$, 1398.3497; found, 1398.3669.

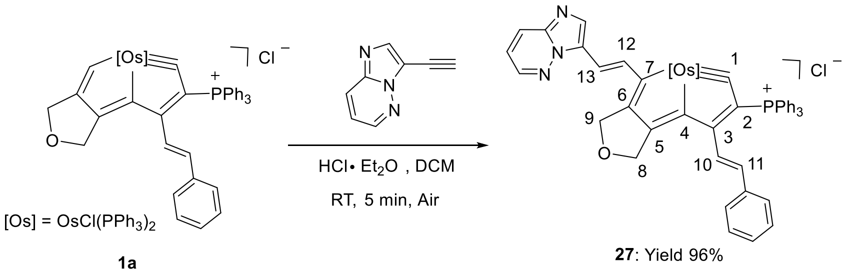

Preparation of complex 27: The excessive proton acid HCl • Et_2O (2 M in ether) solution (2.5 mL) was added to the dichloromethane solution (25 mL) of **1a** (0.50 g, 0.39 mmol) and 3-Ethynylimidazo[1,2-b]pyridazine (0.50 mmol) under air. The reaction mixture was stirred at room temperature for 5 min to yield a navy blue solution. The solution was evaporated under vacuum to a volume of approximately 5 mL and then washed with Et_2O (1 × 100 mL) to afford a navy blue solid. The solid was purified by flash chromatography on silica gel (eluent: 20:1 dichloromethane/methanol) to yield complex **27** as a navy blue solid. Yield: 0.53 g, 96%. ^{1}H NMR plus ^{1}H-^{13}C HSQC (600.1 MHz, CD_2Cl_2): δ = 6.33 (d, *J* = 16.32 Hz, 1H, $C^{13}H$), 5.80 (q, *J* = 16.90 Hz, *J* = 16.90 Hz, 2H, $C^{10}H$ and $C^{11}H$), 5.20 (s, 2H, C^9H), 4.33 (s, 2H, C^8H), 8.49–6.86 ppm (55H, other aromatic protons and $C^{12}H$). ^{31}P NMR (242.9 MHz, CD_2Cl_2): δ = 4.60 (t, *J* = 6.07 Hz, C*P*Ph$_3$), -0.10 ppm (s, Os*P*Ph$_3$). ^{13}C NMR plus DEPT-135, ^{1}H-^{13}C HSQC and ^{1}H-^{13}C HMBC (150.9 MHz, CD_2Cl_2): δ = 316.0 (s, C^1), 217.5 (s, C^7), 172.3 (s, C^5), 168.8 (s, C^6), 167.3 (s, C^4), 158.4 (s, C^3), 73.65 (s, C^9), 70.80 (s, C^8), 143.7–112.3 ppm (other aromatic carbons). Elemental analysis calcd (%) for $C_{79}H_{62}Cl_2N_3OOsP_3$: C 66.66, H 4.39, N 2.95; found: C 66.30, H 4.16, N, 3.08. HRMS (ESI): *m/z* calcd for $[C_{79}H_{62}ClN_3OOsP_3]^+$, 1388.3401; found, 1388.3595.

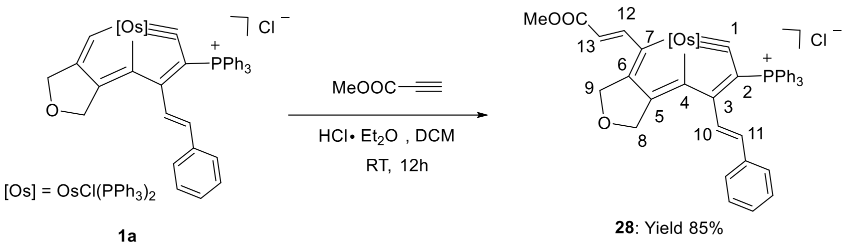

Preparation of complex 28: The excessive proton acid HCl • Et_2O (2 M in ether) solution (2.5 mL) was added to the dichloromethane solution (25 mL) of **1a** (0.50 g, 0.39 mmol) and propiolic acid (0.50 mmol) under air. The reaction mixture was stirred at room temperature for 12 h to yield a magenta solution. The solution was evaporated under vacuum to a volume of approximately 5 mL and then washed with Et_2O (1× 100 mL) to afford a magenta solid. The solid was purified by flash chromatography on silica gel (eluent: 20:1 dichloromethane/methanol) to yield complex **28** as a magenta solid. Yield: 0.44 g, 80%. ^{1}H NMR plus ^{1}H-^{13}C HSQC (600.1 MHz, CD_2Cl_2): δ = 5.79 (q, *J* = 16.57 Hz, *J* = 16.57 Hz, 2H, $C^{10}H$ and $C^{11}H$), 4.97 (s, 2H, C^9H), 4.91 (d, *J* = 16.27 Hz, 1H, $C^{13}H$), 4.24 (s, 2H, C^8H), 3.68 (s, 3H, $COOCH_3$), 7.87–6.87 ppm (51H, other aromatic protons and $C^{12}H$). ^{31}P NMR (242.9 MHz, CD_2Cl_2): δ = 5.34 (t, *J* = 5.93 Hz, C*P*Ph_3), -2.06 ppm (s, Os*P*Ph_3). ^{13}C NMR plus DEPT-135, ^{1}H-^{13}C HSQC and ^{1}H-^{13}C HMBC (150.9 MHz, CD_2Cl_2): δ = 314.9 (m, C^1), 209.1 (t, J_{P-C} = 10.51 Hz, C^7), 171.8 (d, J_{P-C} = 15.02 Hz, C^5), 170.3 (d, J_{P-C} = 23.25 Hz, C^4), 166.6 (s, C^6), 164.1 (d, J_{P-C} = 15.98 Hz, C^3), 154.3 (s, *C*$OOCH_3$), 73.73 (s, C^8), 70.22 (s, C^9), 51.37 (s, $COOCH_3$), 138.2–113.9 ppm (other aromatic carbons). Elemental analysis calcd (%) for $C_{75}H_{61}Cl_2O_3OsP_3$: C 66.03, H 4.51; found: C 66.33, H 4.28. HRMS (ESI): *m/z* calcd for $[C_{75}H_{61}ClO_3OsP_3]^+$, 1329.3128; found, 1329.3288.

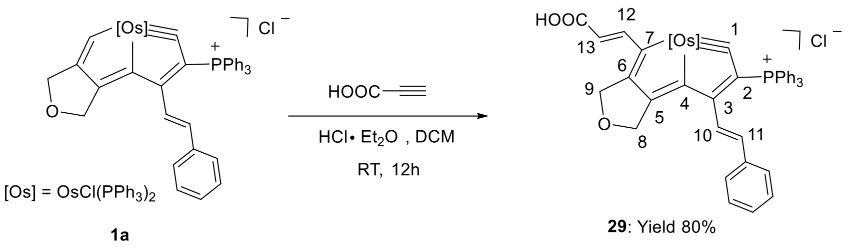

Preparation of complex 29: The excessive proton acid HCl • Et_2O (2 M in ether) solution (2.5 mL) was added to the dichloromethane solution (25 mL) of **1a** (0.50 g, 0.39 mmol) and propiolic acid (0.50 mmol) under air. The reaction mixture was stirred at room temperature for 12 h to yield a brown red solution. The solution was evaporated under vacuum to a volume of approximately 5 mL and then washed with Et_2O (1× 100 mL) to afford a brown red solid. The solid was purified by flash chromatography on silica gel (eluent: 20:1 dichloromethane/methanol) to yield complex **29** as a brown red solid. Yield: 0.44 g, 80%. ^{1}H NMR plus ^{1}H-^{13}C HSQC (600.1 MHz, CD_2Cl_2): δ = 5.79 (q, *J* = 16.60 Hz, *J* = 16.60 Hz, 2H, $C^{10}H$ and $C^{11}H$), 5.60 (d, *J* = 16.50 Hz, 1H, $C^{13}H$), 5.11 (s, 2H, C^9H), 4.18 (s, 2H, C^8H), 7.84–6.86 ppm (52H, other aromatic protons and $C^{12}H$). ^{31}P NMR (242.9 MHz, CD_2Cl_2): δ = 5.10 (d, *J* = 6.21 Hz, C*P*Ph3), -1.49 ppm (s, Os*P*Ph3). ^{13}C NMR plus DEPT-135, ^{1}H-^{13}C HSQC and ^{1}H-^{13}C HMBC (150.9 MHz, CD_2Cl_2): δ = 314.9 (m, C^1), 212.5 (m, C^7), 172.5 (s, C^5), 170.0 (d, $J_{P\text{-}C}$ = 21.28 Hz, C^4), 167.4 (s, C^6), 162.1 (m, C^3), 153.4 (s, *C*OOH), 73.64 (s, C^8), 70.91 (s, C^9), 137.8–116.3 ppm (other aromatic carbons). Elemental analysis calcd (%) for $C_{74}H_{59}Cl_2O_3OsP_3$: C 65.82, H 4.40; found: C 65.45, H 4.56. HRMS (ESI): *m/z* calcd for $[C_{74}H_{59}ClO_3OsP_3]^+$, 1315.2971; found, 1315.3189.

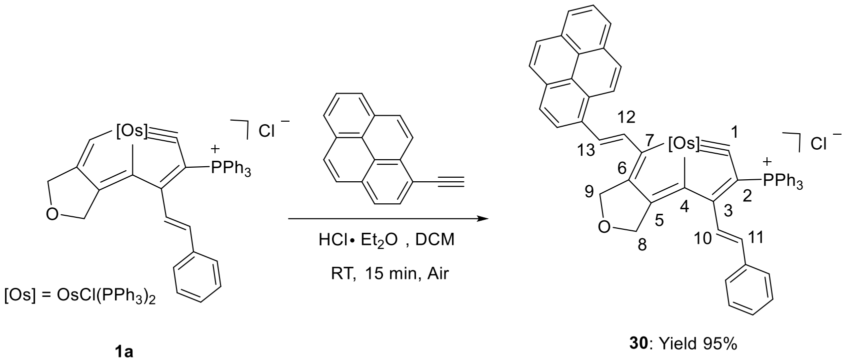

Preparation of complex 30: The excessive proton acid HCl • Et_2O (2 M in ether) solution (2.5 mL) was added to the dichloromethane solution (25 mL) of **1a** (0.50 g, 0.39 mmol) and 1-Ethynylpyrene (0.50 mmol) under air without using standard Schlenk techniques. The reaction mixture was stirred at room temperature for 15 min to yield a dark green solution. The solution was evaporated under vacuum to a volume of approximately 5 mL and then washed with Et_2O (1 × 100 mL) to afford a dark green solid. The solid was purified by flash chromatography on silica gel (eluent: 20:1 dichloromethane/methanol) to yield complex **30** as a dark green solid. Yield: 0.56 g, 95%. ^{1}H NMR plus ^{1}H-^{13}C HSQC (600.1 MHz, CD_2Cl_2): δ = 5.84 (q, *J* = 16.74 Hz, *J* = 16.74 Hz, 2H, C^{10}*H* and C^{11}*H*), 5.42 (s, 2H, C^9*H*), 4.36 (s, 2H, C^8*H*), 8.32–6.89 ppm (61H, other aromatic protons, C^{12}*H* and C^{13}*H*). ^{31}P NMR (242.9 MHz, CD_2Cl_2): δ = 4.76 (t, *J* = 5.86 Hz, C*P*Ph_3), -0.13 ppm (d, *J* = 6.01 Hz, Os*P*Ph_3). ^{13}C NMR plus DEPT-135, ^{1}H-^{13}C HSQC and ^{1}H-^{13}C HMBC (150.9 MHz, CD_2Cl_2): δ = 316.1 (dt, apparent q, $J_{P\text{-}C}$ = 14.35 Hz, $J_{P\text{-}C}$ = 14.35 Hz, C^1), 218.0 (t, $J_{P\text{-}C}$ = 9.41 Hz, C^7), 172.3 (s, C^5), 169.1 (s, C^6), 167.7 (d, $J_{P\text{-}C}$ = 21.41 Hz, C^4), 158.8 (d, $J_{P\text{-}C}$ = 14.88 Hz, C^3), 73.77 (s, C^8), 71.13 (s, C^9), 144.1–120.0 ppm (other aromatic carbons). Elemental analysis calcd (%) for $C_{89}H_{67}Cl_2OOsP_3$: C 70.95, H 4.48; found: C 70.78, H 4.31. HRMS (ESI): *m/z* calcd for $[C_{89}H_{67}ClOOsP_3]^+$, 1471.3702; found, 1471.4095.

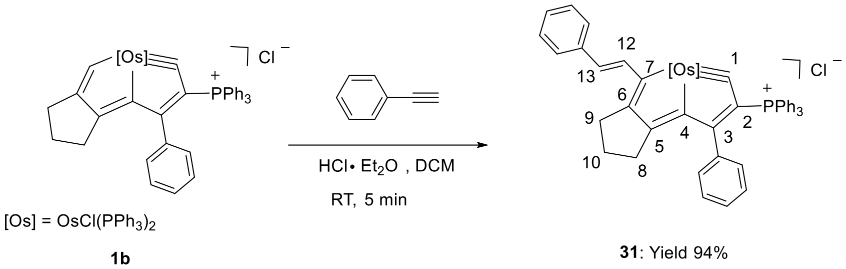

Preparation of complex 31: The excessive proton acid HCl • Et_2O (2 M in ether) solution (2.5 mL) were added to the dichloromethane solution (25 mL) of **1b** (0.49 g, 0.39 mmol) and phenylacetylene (0.50 mmol) under air. The reaction mixture was stirred at room temperature for 5 min to yield a magenta solution. The solution was evaporated under vacuum to a volume of approximately 5 mL and then washed with Et_2O (1× 100 mL) to afford a magenta solid. The solid was purified by flash chromatography on silica gel (eluent: 20:1 dichloromethane/methanol) to yield complex **31** as a magenta solid. Yield: 0.50 g, 94%. ^{1}H NMR plus ^{1}H-^{13}C HSQC (600.1 MHz, CD_2Cl_2): δ = 6.17 (d, J = 16.37 Hz, 1H, $C^{13}H$), 2.95 (s, 2H, $C^{10}H$), 2.01 (m, 2H, C^9H), 1.43 (t, J = 7.55 Hz, 2H, C^8H), 7.78–5.84 ppm (56H, other aromatic protons and $C^{12}H$). ^{31}P NMR (242.9 MHz, CD_2Cl_2): δ = 4.18 (s, C*P*Ph$_3$), -0.35 ppm (s, Os*P*Ph$_3$). ^{13}C NMR plus DEPT-135, ^{1}H-^{13}C HSQC and ^{1}H-^{13}C HMBC (150.9 MHz, CD_2Cl_2): δ = 316.0 (dt, apparent q, $J_{P\text{-}C}$ = 12.81 Hz, $J_{P\text{-}C}$ = 12.81 Hz, C^1), 220.7 (t, $J_{P\text{-}C}$ = 9.52 Hz, C^7), 180.9 (s, C^5), 173.9 (s, C^6), 167.6 (d, $J_{P\text{-}C}$ = 24.19 Hz, C^4), 161.2 (d, $J_{P\text{-}C}$ = 14.75 Hz, C^3), 35.07 (s, C^8), 31.95 (s, C^9), 29.73 (s, C^{10}), 142.7–120.6 ppm (other aromatic carbons). Elemental analysis calcd (%) for $C_{78}H_{63}Cl_2OsP_3$: C 69.17, H 4.69; found: C 69.45, H 4.39. HRMS (ESI): *m/z* calcd for $[C_{78}H_{63}ClOsP_3]^+$, 1319.3438; found, 1319.3608.

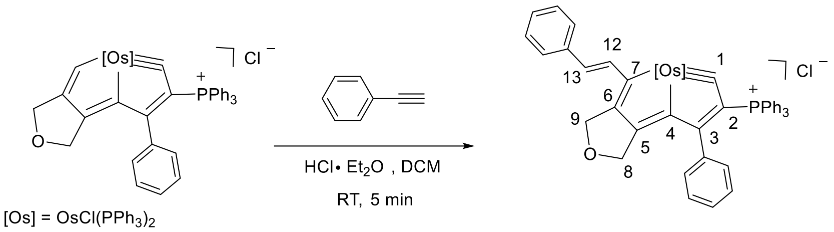

1c **32**: Yield 99%

Preparation of complex 32: The excessive proton acid HCl • Et_2O (2 M in ether) solution (2.5 mL) was added to the dichloromethane solution (25 mL) of **1c** (0.50 g, 0.39 mmol) and phenylacetylene (0.50 mmol) under air without using standard Schlenk techniques. The reaction mixture was stirred at room temperature for 5 min to yield a magenta solution. The solution was evaporated under vacuum to a volume of approximately 5 mL and then washed with Et_2O (1 × 100 mL) to afford a magenta solid. The solid was purified by flash chromatography on silica gel (eluent: 20:1 dichloromethane/methanol) to yield complex **32** as a magenta solid. Yield: 0.52 g, 99%. ^{1}H NMR plus ^{1}H-^{13}C HSQC (600.1 MHz, CD_2Cl_2): δ = 5.76 (d, J = 15.98 Hz, 1H, C^{13}*H*), 5.12 (s, 2H, C^9*H*), 3.58 (s, 2H, C^8*H*), 7.96–5.82 ppm (56H, other aromatic protons and C^{12}*H*). ^{31}P NMR (242.9 MHz, CD_2Cl_2): δ = 4.71 (s, C*P*Ph_3), -1.18 ppm (s, Os*P*Ph_3). ^{13}C NMR plus DEPT-135, ^{1}H-^{13}C HSQC and ^{1}H-^{13}C HMBC (150.9 MHz, CD_2Cl_2): δ = 316.0 (dt, apparent q, $J_{P\text{-}C}$ = 13.25 Hz, $J_{P\text{-}C}$ = 13.25 Hz, C^1), 218.5 (t, $J_{P\text{-}C}$ = 9.38 Hz, C^7), 173.7 (s, C^5), 168.9 (s, C^6), 165.1 (d, $J_{P\text{-}C}$ = 21.80 Hz, C^4), 162.7 (d, $J_{P\text{-}C}$ = 15.29 Hz, C^3), 72.98 (s, C^8), 70.91 (s, C^9), 142.2–120.2 ppm (other aromatic carbons). Elemental analysis calcd (%) for $C_{77}H_{61}Cl_2OOsP_3$: C 68.18, H 4.53; found: C 68.49, H 4.23. HRMS (ESI): *m/z* calcd for $[C_{77}H_{61}ClOOsP_3]^+$, 1321.3230; found, 1321.3385.

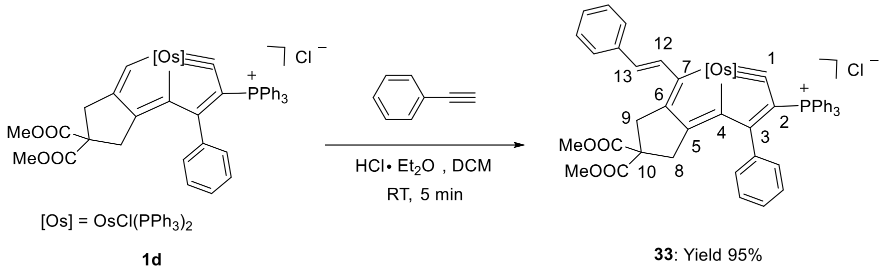

Preparation of complex 33: The excessive proton acid HCl • Et_2O (2 M in ether) solution (2.5 mL) was added to the dichloromethane solution (25 mL) of **1d** (0.50 g, 0.39 mmol) and phenylacetylene (0.50 mmol) under air without using standard Schlenk techniques. The reaction mixture was stirred at room temperature for 5 min to yield a magenta solution. The solution was evaporated under vacuum to a volume of approximately 5 mL and then washed with Et_2O (1 × 100 mL) to afford a magenta solid. The solid was purified by flash chromatography on silica gel (eluent: 20:1 dichloromethane/methanol) to yield complex **33** as a magenta solid. Yield: 0.54 g, 95%. ^{1}H NMR plus ^{1}H-^{13}C HSQC (600.1 MHz, CD_2Cl_2): δ = 6.06 (d, J = 16.60 Hz, 1H, $C^{13}H$), 3.78 (s, 6H, $COOCH_3$), 3.60 (s, 2H, $C^{9}H$), 2.16 (s, 2H, $C^{8}H$), 7.78–5.78 ppm (56H, other aromatic protons and $C^{12}H$). ^{31}P NMR (242.9 MHz, CD_2Cl_2): δ = 4.42 (t, J = 5.89 Hz, CPPh$_3$), -1.26 ppm (d, J = 5.95 Hz, OsPPh$_3$). ^{13}C NMR plus DEPT-135, ^{1}H-^{13}C HSQC and ^{1}H-^{13}C HMBC (150.9 MHz, CD_2Cl_2): δ = 315.6 (dt, apparent q, $J_{P\text{-}C}$ = 13.59 Hz, $J_{P\text{-}C}$ = 13.59 Hz, C^1), 221.9 (t, $J_{P\text{-}C}$ = 8.39 Hz, C^7), 173.7 (s, C^5), 172.1 (s, COOCH$_3$), 168.1 (s, C^6), 167.1 (d, $J_{P\text{-}C}$ = 24.78 Hz, C^4), 162.3 (d, $J_{P\text{-}C}$ = 16.27 Hz, C^3), 63.95 (s, C^{10}), 41.63 (s, C^8), 38.97 (s, C^9), 29.67 (s, COOCH$_3$), 142.0–120.2 ppm (other aromatic carbons). Elemental analysis calcd (%) for $C_{82}H_{67}Cl_2O_4OsP_3$: C 66.98, H 4.59; found: C 66.75, H 4.24. HRMS (ESI): m/z calcd for $[C_{82}H_{67}ClO_4OsP_3]^+$, 1435.3548; found, 1435.3732.

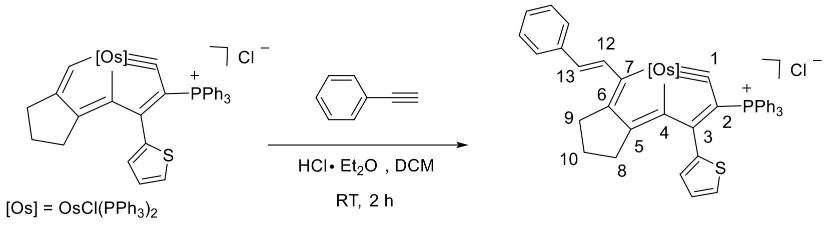

1e

34: Yield 75%

Preparation of complex 34: The excessive proton acid HCl • Et_2O (2 M in ether) solution (2.5 mL) was added to the dichloromethane solution (25 mL) of **1e** (0.50 g, 0.39 mmol) and phenylacetylene (0.50 mmol) under air. The reaction mixture was stirred at room temperature for 2 h to yield a magenta solution. The solution was evaporated under vacuum to a volume of approximately 5 mL and then washed with Et_2O (1× 100 mL) to afford a magenta solid. The solid was purified by flash chromatography on silica gel (eluent: 20:1 dichloromethane/methanol) to yield complex **34** as a magenta solid. Yield: 0.40 g, 75%. ^{1}H NMR plus ^{1}H-^{13}C HSQC (600.1 MHz, CD_2Cl_2): δ = 6.24 (d, *J* = 16.35 Hz, 1H, $C^{13}H$), 2.93 (s, 2H, $C^{10}H$), 1.91 (s, 2H, $C^{9}H$), 1.70 (s, 2H, $C^{8}H$), 7.77–5.83 ppm (54H, other aromatic protons and $C^{12}H$). ^{31}P NMR (242.9 MHz, CD_2Cl_2): δ = 4.18 (d, *J* = 5.67 Hz, C*P*Ph_3), 1.94 ppm (s, Os*P*Ph_3). ^{13}C NMR plus DEPT-135, ^{1}H-^{13}C HSQC and ^{1}H-^{13}C HMBC (150.9 MHz, CD_2Cl_2): δ = 316.1 (d, $J_{P\text{-}C}$ = 14.54 Hz, C^1), 223.3 (s, C^7), 180.8 (s, C^5), 174.8 (s, C^6), 170.3 (d, $J_{P\text{-}C}$ = 20.76 Hz, C^4), 150.4 (d, $J_{P\text{-}C}$ = 16.37 Hz, C^3), 34.92 (s, C^8), 32.20 (s, C^9), 29.30 (s, C^{10}), 143.1–120.6 ppm (other aromatic carbons). Elemental analysis calcd (%) for $C_{76}H_{61}Cl_2OsP_3S$: C 67.10, H 4.52; found: C 67.45, H 4.38. HRMS (ESI): *m/z* calcd for $[C_{76}H_{61}ClOsP_3S]^+$, 1325.3000; found, 1325.3190.

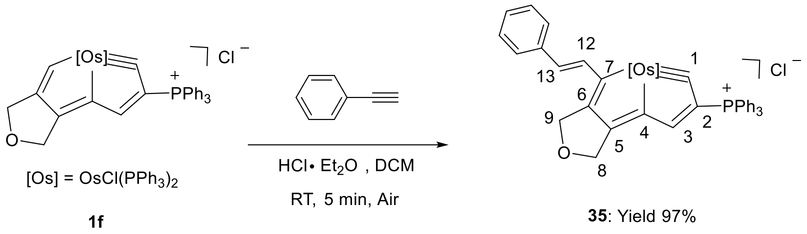

Preparation of complex 35: Compound **1f** was synthesized according to the literatures[2]. The excessive proton acid HCl • Et_2O (2 M in ether) solution (2.5 mL) was added to the dichloromethane solution (25 mL) of **1f** (0.46 g, 0.39 mmol) and phenylacetylene (0.50 mmol) under air without using standard Schlenk techniques. The reaction mixture was stirred at room temperature for 5 min to yield a magenta solution. The solution was evaporated under vacuum to a volume of approximately 5 mL and then washed with Et_2O (1× 100 mL) to afford a magenta solid. The solid was purified by flash chromatography on silica gel (eluent: 20:1 dichloromethane/methanol) to yield complex **35** as a magenta solid. Yield: 0.48 g, 97%. ^{1}H NMR plus ^{1}H-^{13}C HSQC (600.1 MHz, CD_2Cl_2): δ = 5.93 (d, *J* = 16.45 Hz, 1H, $C^{13}H$), 5.12 (s, 2H, C^9H), 4.42 (s, 2H, C^8H), 7.89–7.09 ppm (52H, other aromatic protons and $C^{12}H$). ^{31}P NMR (242.9 MHz, CD_2Cl_2): δ = 4.84 (t, *J* = 5.96 Hz, C*P*Ph3), -0.28 ppm (d, *J* = 5.82 Hz, Os*P*Ph3). ^{13}C NMR plus DEPT-135, ^{1}H-^{13}C HSQC and ^{1}H-^{13}C HMBC (150.9 MHz, CD_2Cl_2): δ = 317.9 (dt, apparent q, J_{P-C} = 14.22 Hz, J_{P-C} = 14.22 Hz, C^1), 219.2 (t, J_{P-C} = 10.06 Hz, C^7), 172.7 (s, C^5), 168.3 (s, C^6), 167.6 (d, J_{P-C} = 24.25 Hz, C^4), 145.8 (d, J_{P-C} = 15.14 Hz, C^3), 70.48 (s, C^8), 70.46 (s, C^9), 143.1–119.8 ppm (other aromatic carbons). Elemental analysis calcd (%) for $C_{71}H_{57}Cl_2OOsP_3$: C 66.61, H 4.49; found: C 66.35, H 4.68. HRMS (ESI): *m/z* calcd for $[C_{71}H_{57}ClOOsP_3]^+$, 1245.2916; found, 1245.3050.

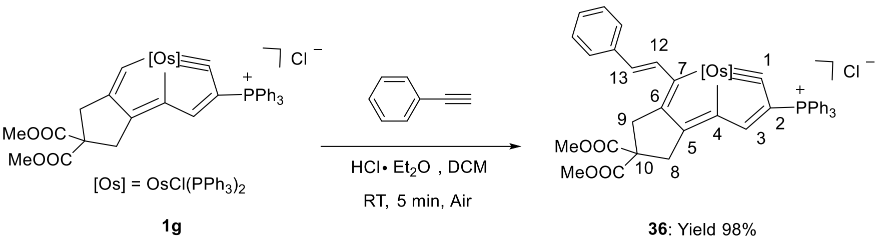

Preparation of complex 36: Compound **1g** was synthesized according to the literatures[2]. The excessive proton acid HCl • Et_2O (2 M in ether) solution (2.5 mL) was added to the dichloromethane solution (25 mL) of **7** (0.50 g, 0.39 mmol) and phenylacetylene (0.50 mmol) under air without using standard Schlenk techniques. The reaction mixture was stirred at room temperature for 5 min to yield a magenta solution. The solution was evaporated under vacuum to a volume of approximately 5 mL and then washed with Et_2O (1× 100 mL) to afford a magenta solid. The solid was purified by flash chromatography on silica gel (eluent: 20:1 dichloromethane/methanol) to yield complex **36** as a magenta solid. Yield: 0.5 g, 98%. ^1H NMR plus ^1H-^{13}C HSQC (600.1 MHz, CD_2Cl_2): δ = 6.24 (d, J = 16.42 Hz, 1H, $C^{13}H$), 3.88 (s, 6H, $COOCH_3$), 3.60 (s, 2H, C^9H), 2.93 (s, 2H, C^8H), 7.87–7.07 ppm (52H, other aromatic protons and $C^{12}H$). ^{31}P NMR (242.9 MHz, CD_2Cl_2): δ = 4.77 (t, J = 5.89 Hz, $CPPh_3$), -0.28 ppm (d, J = 5.73 Hz, $OsPPh_3$). ^{13}C NMR plus DEPT-135, ^1H-^{13}C HSQC and ^1H-^{13}C HMBC (150.9 MHz, CD_2Cl_2): δ = 318.6 (dt, apparent q, $J_{P\text{-}C}$ = 14.39 Hz, $J_{P\text{-}C}$ = 14.39 Hz, C^1), 223.1 (t, $J_{P\text{-}C}$ = 10.33 Hz, C^7), 172.7 (s, C^5), 172.1 (s, $COOCH_3$), 170.6 (d, $J_{P\text{-}C}$ = 21.39 Hz, C^4), 167.1 (s, C^6), 145.3 (d, $J_{P\text{-}C}$ = 16.44 Hz, C^3), 63.71 (s, C^{10}), 38.84 (s, C^8), 37.90 (s, C^9), 143.2–120.0 ppm (other aromatic carbons). Elemental analysis calcd (%) for $C_{76}H_{63}Cl_2O_4OsP_3$: C 65.47, H 4.55; found: C 65.22, H 4.81. HRMS (ESI): m/z calcd for $[C_{76}H_{63}ClO_4OsP_3]^+$, 1359.3234; found, 1359.3416.

Supplementary Discussion

The other aliphatic substituted alkynes, such as 1-hexyne, cyclopentylacetylene, 6-chloro-1-hexyne, 3-phenyl-1-propyne, propargyl alcohol, propargylamine, 4-pentynenitrile, trimethylsilylacetylene, etc., cannot be reacted in the same reaction. The low reactivity of non-aromatic substituted alkynes in these reactions mainly due to two reasons. Firstly, according to the DFT computations, the reaction mechanism undergoes carbenium ion intermediates. So, the carbenium ion intermediates (**Int2** and **Int3**) can be stabilized by the sp^2 hybrid aromatic groups. Secondly, compared with non-aromatic substituents, the distribution of electrons in π bonds can be activated by the conjugation of aromatic substituted groups, which make them more reactive. In addition, we also tried some internal alkynes, such as, diphenylacetylene, 1-phenyl-1-propyne, dimethyl acetylenedicarboxylate, 3-hexyne, etc., but all of them failed to react.

Mechanistic Studies:

Deuterium labeling experiments

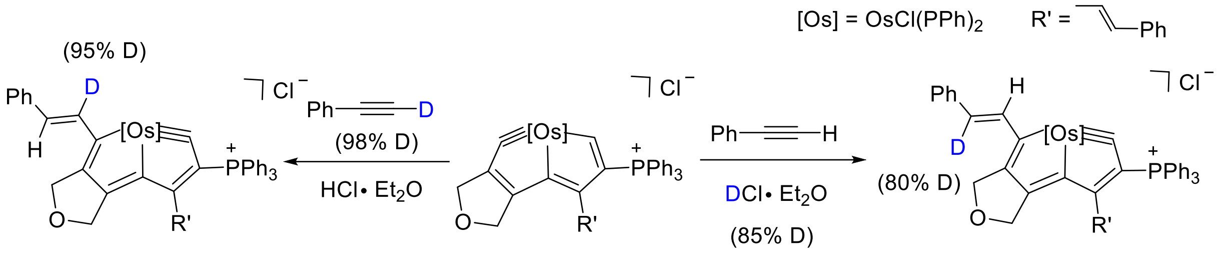

3-d_1 2a 3-d_2

Preparation of complex 3-*d₁*: The excessive proton acid HCl • Et_2O (2 M in ether) solution (0.5 mL) and phenylacetylene (0.05 mmol, 98% D) were added to the dichloromethane solution (5 mL) of **2a** (0.05 g, 0.04 mmol) under N_2 atmosphere. The reaction mixture was stirred at room temperature for 5 min to yield a magenta solution. The solution was evaporated under vacuum to a volume of approximately 1 mL and then washed with Et_2O (1 × 10 mL) to afford a magenta solid. The solid was purified by flash chromatography on silica gel (eluent: 20:1 dichloromethane/methanol) to yield complex **3-d_1** as a magenta solid. ^1H NMR (600.1 MHz, CD_2Cl_2): δ = 5.87 (s, 1H, $C^{13}H$), 5.79 (q, J = 17.03 Hz, J = 17.03 Hz, 2H, $C^{10}H$ and $C^{11}H$), 5.14 (s, 2H, C^9H), 4.26 (s, 2H, C^8H), 7.86–6.87 ppm (55H, other aromatic protons). ^{31}P NMR (242.9 MHz, CD_2Cl_2): δ = 4.81 (t, J = 5.96 Hz, CPPh_3), -0.18 ppm (s, OsPPh_3). ^2D NMR (92.1 MHz, CH_2Cl_2): δ = 7.41 ppm. HRMS (ESI): *m/z* calcd for $[C_{79}H_{62}DClOOsP_3]^+$, 1348.3450; found, 1348.3720.

Preparation of complex 3-*d₂*: The excessive proton acid DCl • Et_2O (2 M in ether, 85% D) solution (0.5 mL) and phenylacetylene (0.05 mmol) were added to the dichloromethane solution (5 mL) of **2a** (0.05 g, 0.04 mmol) under N_2 atmosphere. The reaction mixture was stirred at room temperature for 5 min to yield a magenta solution. The solution was evaporated under vacuum to a volume of approximately 1 mL and then washed with Et_2O (1 × 10 mL) to afford a magenta solid. The solid was purified by flash chromatography on silica gel (eluent: 20:1 dichloromethane/methanol) to yield complex **3-d_2** as a magenta solid. ^1H NMR (600.1 MHz, CD_2Cl_2): δ = 5.78 (q, J = 16.96 Hz, J = 16.96 Hz, 2H, $C^{10}H$ and $C^{11}H$), 5.13 (s, 2H, C^9H), 4.24 (s, 2H, C^8H), 7.69–6.85 ppm (56H, other aromatic protons and $C^{12}H$). ^{31}P NMR (242.9 MHz, CD_2Cl_2): δ = 4.88 (t, J = 5.96 Hz, CPPh_3), -0.25 ppm (d, J = 5.31 Hz, OsPPh_3). ^2D NMR (92.1 MHz, CH_2Cl_2): δ = 5.04 ppm. HRMS (ESI):

m/z calcd for $[C_{79}H_{62}DClOOsP_3]^+$, 1348.3450; found, 1348.3779.

Verification experiment

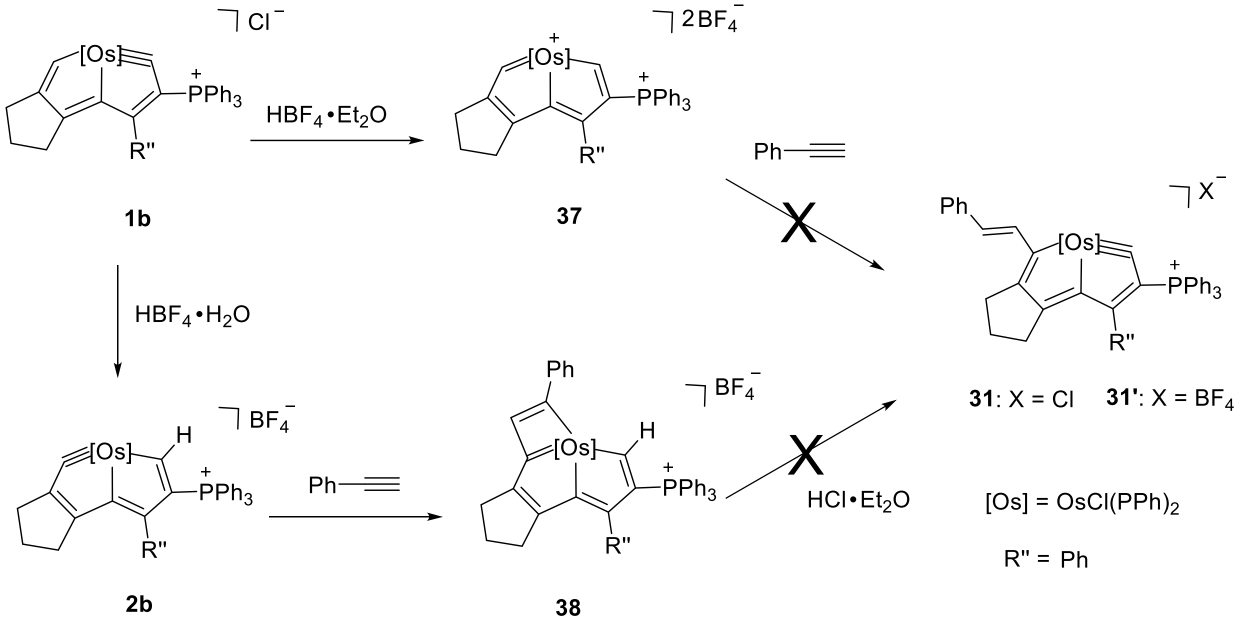

Preparation of complex 37: The excessive proton acid $HBF_4 \cdot Et_2O$ (50-55% w/w HBF_4) solution (0.05 mL) was added to the dichloromethane solution (0.5 mL) of **1b** (0.05 g, 0.04 mmol) under N_2 atmosphere at room temperature for 5 min to yield a brown solution of **37** (in ca. 99% yield based on ^{1}H- and ^{31}P-NMR), which was characterized by in situ ^{1}H NMR, ^{31}P NMR and ^{13}C NMR. The complex **37** only stable in a strong acid condition. ^{1}H NMR plus ^{1}H-^{13}C HSQC (600.1 MHz, CD_2Cl_2): δ = 11.98 (t, J = 8.28 Hz, 1H, C^7H), 10.68 (d, J = 9.08 Hz, 1H, C^1H), 2.52 (m, 2H, $C^{10}H$), 1.69 (t, J = 7.79 Hz, 2H, C^9H), 1.06 (t, J = 7.64 Hz, 2H, C^8H), 7.93–6.37 ppm (50H, other aromatic protons). ^{31}P NMR (242.9 MHz, CD_2Cl_2): δ = 25.46 (s, OsPPh$_3$), 11.56 ppm (s, CPPh$_3$). ^{13}C NMR plus DEPT-135, ^{1}H-^{13}C HSQC and ^{1}H-^{13}C HMBC (150.9 MHz, CD_2Cl_2): δ = 228.5 (s, C^1), 221.2 (s, C^7), 195.8 (s, C^5), 180.8 (d, $J_{P\text{-}C}$ = 18.15 Hz, C^4), 178.4 (s, C^6), 163.5 (d, $J_{P\text{-}C}$ = 13.61 Hz, C^3), 36.20 (s, C^8), 30.37 (s, C^9), 27.15 (s, C^{10}), 135.8–116.8 ppm (other aromatic carbons). Elemental analysis calcd (%) for $C_{70}H_{58}B_2ClF_8OsP_3$: C 60.42, H 4.20; found: C 62.63, H 4.53.

Preparation of complex 38: The phenylacetylene (0.05 mmol) was added to the dichloromethane solution (5 mL) of **2b** (0.05 g, 0.04 mmol) and phenylacetylene (0.50 mmol) under N_2 atmosphere. The reaction mixture was stirred at room temperature for 5 min to yield a dark green solution. The solution was evaporated under vacuum to a volume of approximately 5 mL and then washed with Et_2O (1 × 10

mL) to afford a dark green solid. The solid was purified by flash chromatography on silica gel (eluent: 30:1 dichloromethane/methanol) to yield complex **38** as a dark green solid. Yield: 0.04 g, 75%. ^{1}H NMR plus ^{1}H-^{13}C HSQC (600.1 MHz, $CDCl_3$): δ = 13.85 (d, *J* = 18.66 Hz, 1H, $C^{1}H$), 1.68 (t, *J* = 8.64 Hz, 2H, $C^{10}H$), 1.32 (m, 2H, $C^{9}H$), 1.28 (s, 2H, $C^{8}H$), 7.74–6.34 ppm (56H, other aromatic protons and $C^{11}H$). ^{31}P NMR (242.9 MHz, CD_2Cl_2): δ = 10.49 (s, Os*P*Ph_3), -17.57 ppm (s, C*P*Ph_3). ^{13}C NMR plus DEPT-135, ^{1}H-^{13}C HSQC and ^{1}H-^{13}C HMBC (150.9 MHz, CD_2Cl_2): δ = 235.2 (d, *J* = 14.45 Hz, C^{1}), 189.6 (s, C^{5}), 182.5 (t, *J* = 7.82 Hz, C^{7}), 177.6 (d, *J* = 23.23 Hz, C^{4}), 166.2 (t, *J* = 13.47 Hz, C^{6}), 162.4 (t, *J* = 9.99 Hz, C^{3}), 151.0 (t, *J* = 5.28 Hz, C^{12}), 144.9 (s, C^{11}), 34.38 (s, C^{8}), 28.90 (s, C^{10}), 22.81 (s, C^{9}), 137.4–120.5 ppm (other aromatic carbons). Elemental analysis calcd (%) for $C_{78}H_{63}BClF_4OsP_3$: C 66.64, H 4.52; found: C 66.35, H 4.32. HRMS (ESI): *m/z* calcd for $[C_{78}H_{63}ClOsP_3]^+$, 1319.3438; found, 1319.3660.

Supplementary References

1. Zhu, C.; Li, S.; Luo, M.; Zhou, X.; Niu, Y.; Lin, M.; Zhu, J.; Cao, Z.; Lu, X.; Wen, T. B.; Xie, Z.; Schleyer, P. v. R.; Xia, H. Stabilization of anti-aromatic and strained five-membered rings with a transition metal. *Nat. Chem.* **2013**, *5*, 698–703;

2. Zhuo, Q.; Lin, J.; Hua, Y.; Zhou, X.; Shao, Y.; Chen, S.; Chen, Z.; Zhu, J.; Zhang, H.; Xia, H. Multiyne chains chelating osmium via three metal-carbon sigma bonds. *Nat. Commun.*, **2017**, *8*, 1912.
