# Supplementary material for: Addition of alkynes and osmium carbynes towards functionalized dπ–pπ conjugated systems
Source: Nat Commun. 2020 Sep 16;11:4651. doi: 10.1038/s41467-020-18498-2 (PMC7495419; doi:10.1038/s41467-020-18498-2)
Supplement: Supplementary file 3 — Description of Additional Supplementary Files [file 41467_2020_18498_MOESM3_ESM.pdf]

Description of Additional Supplementary Files

File Name: Supplementary Data 1
Description: XYZ coordinates of the optimized structures in the DFT studies
