# Supplementary material for: Addition of alkynes and osmium carbynes towards functionalized dπ–pπ conjugated systems
Source: Nat Commun. 2020 Sep 16;11:4651. doi: 10.1038/s41467-020-18498-2 (PMC7495419; doi:10.1038/s41467-020-18498-2)
Supplement: Supplementary file 4 — Supplementary Data 1 [file 41467_2020_18498_MOESM4_ESM.pdf]

Cartesian Coordinates for DFT calculation

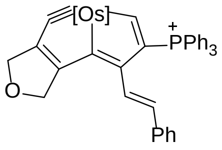

[Os] = OsCl(PPh$_3$)$_2$

2a

E = -4390.29965480

Os	1.71324700	-0.00514900	-0.11424700
Cl	3.25771500	-0.09255300	1.80261200
P	1.96116400	2.39032500	0.08145200
P	1.73210500	-2.42838400	-0.11143800
P	-2.66259700	-0.05848000	1.51870300
C	3.62047300	3.02979700	-0.33900200
C	1.62017400	3.03013800	1.76304200
C	3.10681000	-3.07150700	-1.13801600
O	0.92512200	0.78172400	-5.04960100
C	5.46397200	-2.95833200	-1.68542900
H	6.43878300	-2.50065600	-1.54511800
C	1.97744000	-3.27814600	1.49396900
C	2.49515600	0.04176000	-1.78998800
C	4.72919300	2.17901900	-0.42154100
H	4.60725500	1.12030900	-0.23608100
C	0.33881900	2.89051500	2.31745300
H	-0.44378200	2.41354900	1.74610300
C	0.88343700	3.32677300	-1.07277500
C	2.71769600	-4.46855100	1.54602000
H	3.17544100	-4.86360000	0.64631300
C	-0.28201900	3.99798500	-0.68400900
H	-0.51271200	4.12071300	0.36688400
C	2.63186500	3.61332400	2.53526500
H	3.63334500	3.71106300	2.13365500
C	0.10650600	0.12107600	-1.47239200
C	5.98635000	2.70254600	-0.72822800
H	6.84274700	2.03765000	-0.78937300
C	2.95860700	-4.14486200	-2.02423200
H	1.99317900	-4.62191100	-2.15115600
C	2.30317200	-4.64196400	3.92084300
H	2.43840100	-5.16349400	4.86392000
C	4.37033100	-2.48214700	-0.96696300
H	4.49101000	-1.66149800	-0.26744900
C	2.87756800	-5.14651100	2.75390100
H	3.45803600	-6.06380800	2.78190600
C	0.05991500	3.35981600	3.59888800
H	-0.94955900	3.27665500	3.99388800
C	1.39804000	-2.78067600	2.66850700
H	0.83570900	-1.85789000	2.64879200
C	1.56230400	-3.46045800	3.87377700
H	1.12363300	-3.05382600	4.77806100
C	0.49020500	0.23261300	-2.83441400
C	3.78314400	4.40699700	-0.56372700
H	2.92624100	5.07124200	-0.50588400
C	1.86807000	0.17337400	-3.02688900
C	-1.22306400	0.05442500	0.44905100
C	0.24052100	-3.25115800	-0.79896000
C	1.20297600	3.26036100	-2.43871600
H	2.12890100	2.79356400	-2.75134200
C	2.35783100	4.06038800	3.82935000
H	3.15369600	4.50316200	4.42048000
C	5.30877700	-4.01677800	-2.58520300

H	6.16217800	-4.38059400	-3.14974900
C	6.14387600	4.07023000	-0.95262200
H	7.12344400	4.47315400	-1.19263700
C	1.07381800	3.94432100	4.36172800
H	0.86452500	4.30630800	5.36399400
C	-1.20434900	0.13431200	-0.99811700
C	-0.83760300	4.42548800	-3.00294800
H	-1.50918500	4.84087100	-3.74824800
C	4.05749700	-4.60934600	-2.75140600
H	3.93243400	-5.43763800	-3.44261400
C	-1.13671300	4.54226700	-1.64523300
H	-2.03401300	5.06923100	-1.33141500
C	0.34602700	3.79677200	-3.39687700
H	0.60013000	3.70600600	-4.44838100
C	5.04009000	4.92338400	-0.86875000
H	5.15791600	5.98849800	-1.04437600
C	-0.11598700	-3.00831200	-2.13591300
H	0.48531100	-2.33526000	-2.73535100
C	-0.52628100	-4.14517700	-0.04148100
H	-0.25842500	-4.34750000	0.98947000
C	-2.91693600	2.70188500	1.21932100
H	-2.29938600	2.58768300	0.33561500
C	-0.15975700	0.46188800	-4.17134000
H	-0.69440000	-0.43764800	-4.52510000
H	-0.86832500	1.29681100	-4.16765600
C	-3.24295000	1.58761900	2.00406700
C	-3.54035400	0.73699300	-1.68680000
H	-3.64247000	1.41573500	-0.84618100
C	-4.02519600	-0.97135800	0.76267700
C	-3.36047300	3.96956900	1.59257400
H	-3.09584500	4.83103700	0.98753700
C	-1.20917300	-3.65616200	-2.70587800
H	-1.46148100	-3.47422200	-3.74677400
C	-2.18795700	-0.90354600	3.04964300
C	-2.35897100	0.11988000	-1.88843700
H	-2.23653600	-0.48713100	-2.78194500
C	-1.96043200	-4.56042000	-1.94858100
H	-2.79596600	-5.08647800	-2.40153700
C	-1.62055200	-4.79843900	-0.61685700
H	-2.19839500	-5.50138200	-0.02369000
C	-3.73501200	-2.11909400	0.01026100
H	-2.70996400	-2.44763100	-0.11827900
C	-4.12859900	4.13062500	2.74682300
H	-4.46850100	5.11938000	3.03890900
C	2.18054800	0.36236700	-4.48373600
H	2.92372600	1.14286300	-4.68030200
H	2.52908000	-0.56768900	-4.95877400
C	-1.49135600	-0.20067600	4.04687000
H	-1.25744600	0.85086300	3.92109900
C	-6.08926900	-2.36563100	-0.48656000
H	-6.89042400	-2.89866100	-0.98863700
C	-4.77032800	-2.81325100	-0.60716000
H	-4.54126200	-3.69368100	-1.19730000
C	-4.01254500	1.75117500	3.16762600
H	-4.25212200	0.89471000	3.78998600
C	-5.34563600	-0.52340100	0.88831400
H	-5.56941600	0.37818900	1.44766200
C	-7.19613900	0.22739000	-3.83523300
H	-8.13562200	0.09480600	-4.36322500
C	-4.45463100	3.02172000	3.53283900

H	-5.04721200	3.14705000	4.43357100
C	-1.09224100	-0.85908100	5.20772400
H	-0.55172000	-0.31350500	5.97458400
C	-1.38861500	-2.21333700	5.38097900
H	-1.08524800	-2.72171600	6.29116400
C	-2.47369800	-2.26511100	3.22072500
H	-3.01337200	-2.81125000	2.45516600
C	-6.37546900	-1.22493100	0.26177000
H	-7.39649700	-0.86692500	0.34338200
C	-2.07162000	-2.91512600	4.38656700
H	-2.29665300	-3.96851400	4.51921200
C	-6.12497500	-0.63740600	-4.08129200
H	-6.23478600	-1.44604100	-4.79809500
C	-7.05323000	1.25846900	-2.90468800
H	-7.88105100	1.93307400	-2.70705400
C	-5.84758400	1.42169800	-2.22801900
H	-5.73771600	2.22079300	-1.49871600
C	-4.92162000	-0.47494100	-3.40368200
H	-4.11107600	-1.17613200	-3.57585000
C	-4.75973300	0.56412800	-2.46884700
C	0.03557200	0.00686600	1.03013300
H	0.13221800	-0.04433900	2.11275700

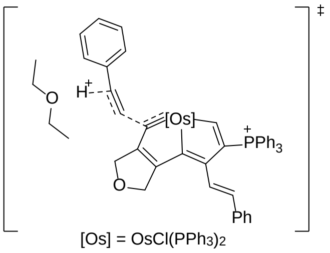

[Os] = OsCl(PPh3)2

TS1

E = -4932.73743823

Os	0.74220600	0.06473500	-0.70781700
Cl	1.40370900	-0.00325400	-3.09106100
P	0.92316200	-2.35698200	-0.83239700
P	0.56025500	2.49409600	-0.72851400
P	-3.94364700	-0.11745100	-0.75661300
C	2.64467900	-2.97570100	-0.93542700
C	0.11779800	-3.06459500	-2.31737300
C	2.03477100	3.44030500	-1.29671400
O	1.62862900	0.09321900	4.28894800
C	4.09487700	3.57755900	-2.57369200
H	4.82244900	3.10215900	-3.22641500
C	-0.79500200	3.19587000	-1.74604000
C	2.04941000	0.24493100	0.62996300
C	3.65268000	-2.17691000	-1.49041600
H	3.42128700	-1.17422100	-1.82490700
C	-1.28001300	-3.16251200	-2.38803800
H	-1.88941400	-2.84041500	-1.55310500
C	4.23309300	0.83598300	0.25870900
H	3.90305300	1.72049300	-0.24737900
C	0.23897400	-3.30954400	0.58416400
C	-1.47129600	4.37131700	-1.38399700
H	-1.22875600	4.88154500	-0.45863900
C	-0.61997800	-4.40815900	0.44187700
H	-0.93112300	-4.73731900	-0.54174800
C	0.88481100	-3.48607500	-3.41032100
H	1.96487300	-3.40934500	-3.37745600
C	-0.32098500	0.06937700	1.11632100
C	4.94616700	-2.68115200	-1.62530600
H	5.72229200	-2.05561800	-2.05685300
C	2.15843900	4.80508100	-0.98487700
H	1.40509900	5.29565300	-0.37769900
C	-2.78845600	4.25080600	-3.41011700

H	-3.56626200	4.65487100	-4.05095500
C	3.00645000	2.83439400	-2.10468600
H	2.88927600	1.79862100	-2.40017400
C	-2.46992800	4.88925700	-2.20988100
H	-2.99085000	5.79671000	-1.91884400
C	-1.89691800	-3.70303000	-3.51560500
H	-2.97844500	-3.80574200	-3.53952000
C	-1.10182200	2.57452000	-2.96628100
H	-0.55056700	1.69503000	-3.27898900
C	-2.09335500	3.10235300	-3.79118300
H	-2.32700000	2.60746100	-4.72752000
C	0.47550200	0.19756800	2.27484400
C	2.94630900	-4.28336800	-0.52450200
H	2.17229100	-4.91258100	-0.09812400
C	1.84183300	0.30890100	1.99949200
C	-2.20856900	-0.12415400	-0.26083300
C	0.26983900	3.15101700	0.96008200
C	5.07657700	0.04761700	0.71738700
C	0.67074800	-2.95503400	1.87250400
H	1.36080100	-2.13358800	1.99941500
C	0.26358600	-4.00171800	-4.54879600
H	0.87035800	-4.31968100	-5.39087000
C	4.22237200	4.92680100	-2.24117800
H	5.05982100	5.50754200	-2.61687400
C	5.24544100	-3.97735800	-1.20713400
H	6.25525000	-4.36341600	-1.30578200
C	-1.12522300	-4.11851200	-4.60334600
H	-1.60352100	-4.53838900	-5.48296200
C	-1.71864700	-0.01588100	1.09931500
C	-0.65773100	-4.72239700	2.84608000
H	-1.00719600	-5.26699300	3.71784200
C	3.24821700	5.53943700	-1.44701100
H	3.33107600	6.59316000	-1.19880700
C	-1.06598200	-5.10550800	1.56875000
H	-1.71794100	-5.96510900	1.44112300
C	0.22364400	-3.64827800	2.99411000
H	0.56897500	-3.35142700	3.98026800
C	4.24199900	-4.77822700	-0.65780200
H	4.46801900	-5.78919700	-0.33273500
C	1.34365100	3.49560700	1.79194200
H	2.35729800	3.47131000	1.40680200
C	-1.02685900	3.17360900	1.49372900
H	-1.86827100	2.87505900	0.88377200
C	-3.91790500	-2.78605600	0.07592700
H	-2.91137200	-2.60019600	0.43681700
C	0.29845800	0.21723900	3.76790100
H	-0.16404200	1.15758900	4.10463000
H	-0.30395900	-0.61701500	4.14336400
C	-4.66144900	-1.76032000	-0.52693400
C	-3.65410500	-0.50071200	2.63185900
H	-4.03211600	-1.26768600	1.96580000
C	-4.82482800	1.13941100	0.19205400
C	-4.47722000	-4.05549900	0.21426800
H	-3.89676700	-4.84391700	0.68144900
C	1.12192000	3.88449500	3.11456000
H	1.96397500	4.15706800	3.74353300
C	-4.15254100	0.27737000	-2.51283800
C	-2.50047800	0.12562900	2.31900000
H	-2.08326600	0.82503300	3.03676100
C	-0.17558200	3.93222300	3.62594500

H	-0.34620700	4.24635800	4.65103600
C	-1.25109900	3.57161100	2.81011300
H	-2.26808200	3.60262700	3.19138500
C	-4.32482000	2.45107200	0.13223800
H	-3.49411500	2.69458500	-0.52329700
C	-5.77218900	-4.30400700	-0.24470200
H	-6.20603700	-5.29298000	-0.13466200
C	2.59574800	0.45227600	3.29186200
H	3.45385800	-0.21830700	3.39207700
H	2.94517600	1.48644200	3.44036700
C	-3.58704200	-0.56820200	-3.48199900
H	-2.98977200	-1.42291700	-3.19251200
C	-5.99180300	3.13859500	1.74265300
H	-6.44089100	3.91399100	2.35533700
C	-4.91109300	3.44645400	0.90903400
H	-4.52616900	4.46050500	0.86066800
C	-5.95717600	-2.01558500	-1.00442700
H	-6.52373000	-1.23139100	-1.49802400
C	-5.92066600	0.83683300	1.00604400
H	-6.30275300	-0.17637100	1.05940000
C	-6.23615200	0.18269600	5.96004400
H	-6.90922900	0.35641900	6.79383700
C	-6.51094800	-3.28513500	-0.85395900
H	-7.51398600	-3.48226000	-1.21845600
C	-3.79813800	-0.31798100	-4.83439600
H	-3.34641300	-0.97139300	-5.57410300
C	-4.59983500	0.75645200	-5.23135200
H	-4.77646300	0.94119600	-6.28649600
C	-4.95802900	1.35282700	-2.91305400
H	-5.41263300	2.00508600	-2.17786800

C	-6.49862000	1.83948200	1.78467300
H	-7.33024400	1.59701100	2.43779600
C	-5.18252800	1.58313300	-4.27081800
H	-5.81405500	2.41210100	-4.57431400
C	-5.15078800	1.03986400	5.74531500
H	-4.98544200	1.88068800	6.41223000
C	-6.45147300	-0.89395900	5.09780700
H	-7.29093100	-1.56304200	5.26008400
C	-5.58600300	-1.10998600	4.02836400
H	-5.75199600	-1.94841300	3.35572600
C	-4.28805100	0.82486300	4.67715800
H	-3.46425200	1.51194700	4.50895600
C	-4.48951600	-0.25845500	3.80139900
C	-1.20810700	-0.11136800	-1.21314300
H	-1.45200000	-0.15857200	-2.27062900
H	6.11086000	1.39214300	0.79871400
O	6.80010300	2.20414500	0.84521800
C	7.84593500	2.01426900	1.88181700
H	8.38275200	2.96387800	1.90139500
H	8.51379400	1.21776800	1.54770900
C	7.22878900	2.58120900	-0.52779600
H	6.30829500	2.94416900	-0.98885300
H	7.90996500	3.42104400	-0.38229700
C	7.84781800	1.42371900	-1.28234300
H	7.14257200	0.59237900	-1.37536200
H	8.10419200	1.76514800	-2.29083400
H	8.76928200	1.06664700	-0.81342500
C	7.16477600	1.69925200	3.19451500
H	7.93008700	1.63553300	3.97457100
H	6.45978700	2.48847800	3.46955600

H	6.64391400	0.73859800	3.15656700
C	5.52180700	-1.19265400	1.29578900
C	4.56908100	-2.10624900	1.78337300
C	6.88558400	-1.52390400	1.35903600
C	4.97759400	-3.31596800	2.33109900
H	3.51813100	-1.86737400	1.69556300
C	7.28885100	-2.74022600	1.90471700
H	7.62627800	-0.84193600	0.95228200
C	6.33659900	-3.63498100	2.39724300
H	4.23187600	-4.01621200	2.69355000
H	8.34406300	-2.99153000	1.94393800
H	6.65277000	-4.58145600	2.82432800

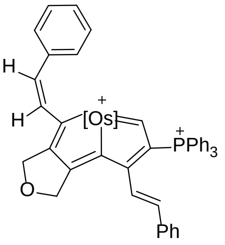

[Os] = $OsCl(PPh_3)_2$

Int1

E = -4699.14823337

Os	1.17793000	0.46262700	-0.45135300
Cl	1.84204700	1.01556600	-2.72190600
P	1.80441400	-1.90450500	-1.16402800
P	0.72474100	2.91096700	0.02834700
P	-3.43462000	-0.33251600	-1.01720200
C	3.63088900	-2.18270900	-1.30978900
C	1.17195100	-2.32622500	-2.85343900
C	2.04958700	4.15477600	-0.36177400
O	1.58269100	-0.59161200	4.57701400
C	3.98185800	4.90709600	-1.63219100
H	4.75574200	4.69739100	-2.36495700
C	-0.70118700	3.66669400	-0.88126600
C	2.40349500	0.49463900	1.16458400
C	4.45643400	-1.21939400	-1.91736800
H	4.03926100	-0.27982200	-2.25987300
C	-0.15663100	-2.75242600	-3.01822900
H	-0.81247500	-2.84071300	-2.15884500
C	3.66142900	0.99234100	0.65612900
H	3.58638400	1.87211800	0.02175900
C	1.24294200	-3.33655900	-0.13412400
C	-1.51215700	4.65587400	-0.29706900
H	-1.40078100	4.91529400	0.74950200
C	0.93670600	-4.58426000	-0.70871600
H	0.95880600	-4.71445400	-1.78413100
C	2.00059700	-2.26325300	-3.98318600
H	3.03332600	-1.95137400	-3.88998000
C	-0.02341200	-0.07114000	1.26506100
C	5.81397800	-1.47481100	-2.11536800
H	6.43333600	-0.72563000	-2.60060400
C	1.96083500	5.43736400	0.21288800
H	1.17434600	5.67122400	0.92217900
C	-2.59769700	5.05839700	-2.42447000
H	-3.32146300	5.60486900	-3.02223900
C	3.06134900	3.90821700	-1.30232400
H	3.11554000	2.95499600	-1.81276400
C	-2.45860100	5.33875500	-1.06404000
H	-3.07088600	6.10617400	-0.59871500
C	-0.63581600	-3.12664600	-4.27509300
H	-1.65596900	-3.48818500	-4.37441100
C	-0.84576700	3.39450100	-2.25160700
H	-0.19902300	2.67078600	-2.73573300
C	-1.78605800	4.08866100	-3.01490200
H	-1.87379300	3.87401600	-4.07541900
C	0.63947500	-0.14107300	2.50298100
C	4.20326900	-3.39706500	-0.89436400

H	3.59609600	-4.16134400	-0.42407100
C	1.99559100	0.17227300	2.44338900
C	-1.76934400	-0.20103600	-0.28663700
C	0.38086500	3.20109100	1.81972100
C	4.95836200	0.60756100	0.90571200
C	1.24948000	-3.23183500	1.26599500
H	1.51123400	-2.29417200	1.73565500
C	1.51328400	-2.61867800	-5.24296600
H	2.17352700	-2.56572900	-6.10347800
C	3.89984100	6.16556000	-1.03893700
H	4.61498500	6.94073600	-1.29763500
C	6.37254900	-2.68550100	-1.70097700
H	7.42896700	-2.88226600	-1.85818100
C	0.19756100	-3.05550400	-5.39418200
H	-0.17146700	-3.35303800	-6.37137800
C	-1.40363400	-0.31779800	1.10909700
C	0.61314300	-5.55559100	1.48862200
H	0.37283600	-6.41125500	2.11293500
C	2.88121900	6.42918700	-0.11962700
H	2.79814200	7.41086500	0.33743500
C	0.61931000	-5.67965700	0.09772200
H	0.39421900	-6.63584500	-0.36629800
C	0.93645100	-4.32892100	2.07070300
H	0.95308500	-4.22379100	3.15191000
C	5.56398000	-3.64227900	-1.08808500
H	5.98713000	-4.58795200	-0.76233400
C	1.41619800	3.57345500	2.69320400
H	2.41726400	3.74430800	2.31189500
C	-0.90083200	2.98769800	2.35486600
H	-1.71878100	2.68469500	1.71013300
C	-3.11504800	-3.12001000	-0.71553100
H	-2.20263400	-2.90331100	-0.16856400
C	0.31177700	-0.45879400	3.94230000
H	-0.26956600	0.35883000	4.40228300
H	-0.24535600	-1.39155600	4.07928400
C	-3.91065200	-2.07865700	-1.21998700
C	-3.33165200	-1.39204400	2.34581000
H	-3.59421300	-1.99868600	1.48467600
C	-4.62856800	0.56137000	0.01940900
C	-3.49224900	-4.44872000	-0.92092400
H	-2.86738100	-5.24665900	-0.53157900
C	1.16831200	3.76093600	4.05549300
H	1.97926800	4.06750400	4.70983200
C	-3.50363200	0.38812900	-2.69091300
C	-2.26797100	-0.55565600	2.25754000
H	-1.97273800	-0.02036300	3.15365300
C	-0.11547300	3.57452800	4.57002200
H	-0.31015500	3.73776400	5.62583400
C	-1.14868600	3.18267600	3.71506700
H	-2.15440200	3.04297400	4.10231900
C	-4.35708000	1.90548500	0.33430500
H	-3.44809400	2.38512800	-0.01889400
C	-4.66021100	-4.74491200	-1.62643200
H	-4.95222200	-5.77883700	-1.78417200
C	2.57421400	0.13113100	3.83511100
H	3.52197400	-0.40461800	3.92546700
H	2.70916700	1.14686600	4.23936200
C	-2.75377100	-0.19864600	-3.72706700
H	-2.09742100	-1.03971400	-3.53438800
C	-6.45751200	2.04204300	1.52866900
H	-7.16966500	2.61723500	2.11269400
C	-5.27353100	2.63946200	1.08512500
H	-5.06596500	3.67966800	1.31752100
C	-5.07606800	-2.38264700	-1.94789800
H	-5.68369800	-1.58926000	-2.37357300
C	-5.81712900	-0.03477800	0.46395900

H	-6.03584200	-1.07184500	0.23358600
C	-5.80921600	-2.22626900	5.72692600
H	-6.44145600	-2.45195300	6.58043400
C	-5.45021400	-3.71178200	-2.13976700
H	-6.35279700	-3.94047400	-2.69809100
C	-2.85837900	0.28763600	-5.02820600
H	-2.26876400	-0.16932700	-5.81708200
C	-3.72977900	1.34235800	-5.31565500
H	-3.82290900	1.70814100	-6.33396400
C	-4.37741000	1.44747000	-2.98384200
H	-4.97696500	1.90384600	-2.20554600
C	-6.72652000	0.70843200	1.21850300
H	-7.64383700	0.24170000	1.56353100
C	-4.48967800	1.91474000	-4.29502100
H	-5.17765300	2.72545700	-4.51488000
C	-4.88864200	-1.17339100	5.79622400
H	-4.81065500	-0.58118500	6.70318700
C	-5.91320800	-2.98293900	4.55780500
H	-6.62514600	-3.80076700	4.49885900
C	-5.09987600	-2.68868400	3.46648200
H	-5.17756800	-3.28458500	2.55986200
C	-4.07512900	-0.88039800	4.70795300
H	-3.37652800	-0.05173600	4.77799000
C	-4.16211100	-1.63719500	3.52061800
C	-0.68899900	0.16297500	-1.06048600
H	-0.79078500	0.35427500	-2.12664800
H	5.69414700	1.27647200	0.46046800
C	5.56197800	-0.46153400	1.67474400
C	4.88449300	-1.61627900	2.12684000
C	6.94112700	-0.33952700	1.96851400
C	5.55511700	-2.59055500	2.85718500
H	3.84023600	-1.75972600	1.87876300
C	7.60603900	-1.30832000	2.71076600
H	7.48262700	0.53430800	1.61552100
C	6.91332000	-2.43686300	3.15948100
H	5.02201100	-3.47573200	3.19123200
H	8.66115800	-1.18936200	2.93667800
H	7.43035800	-3.19825700	3.73598400

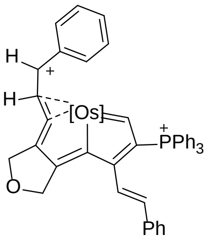

[Os] = $OsCl(PPh_3)_2$

Int2

E = -4699.14394641

Os	-1.38988400	0.47229900	-0.03448400
Cl	-2.46748800	1.12104400	2.06269800
P	-1.87342500	-1.94503700	0.47253500
P	-0.73219200	2.82593900	-0.37321900
P	3.03777500	-0.01011600	1.44513400
C	-0.41905300	-3.08960600	0.64132800
C	-2.76631600	-3.07521800	-0.67902600
C	-2.05495400	4.09727700	-0.52137700
O	-0.74451800	-0.85425400	-4.93521500
C	-4.12029200	5.05622800	0.31530700
H	-4.97102800	5.00555700	0.98827500
C	0.33883700	3.62216100	0.88751900
C	-2.24259300	0.44457900	-1.86167300
C	0.35699300	-3.33497300	-0.50447800
H	0.23355800	-2.71955900	-1.38521900
C	-2.56918000	-2.95968200	-2.06096100
H	-2.01697400	-2.12400300	-2.45987100
C	-3.35721700	1.24820800	-1.45391300
H	-3.19529900	2.31481900	-1.59817600
C	-2.74669300	-2.03964100	2.06855200

C	1.06311500	4.77252600	0.53090600
H	1.07668700	5.11300600	-0.49862000
C	-4.13566500	-2.20507000	2.11849700
H	-4.69895600	-2.37378700	1.21140600
C	-3.42883100	-4.20833200	-0.18277200
H	-3.54195900	-4.35638400	0.88416100
C	0.12594600	-0.13073600	-1.40811900
C	1.24332400	-4.40747400	-0.55169100
H	1.81848400	-4.58687100	-1.45513200
C	-1.90764600	5.19754200	-1.38097300
H	-1.04722300	5.27609700	-2.03492300
C	1.75015200	5.06957700	2.83171400
H	2.29234800	5.63387500	3.58435600
C	-3.16421500	4.04175800	0.33925900
H	-3.26586600	3.21604700	1.03368800
C	1.76452600	5.48886400	1.49766900
H	2.31184500	6.38258200	1.21269100
C	-3.06955400	-3.92360600	-2.93582000
H	-2.90467900	-3.81420400	-4.00370300
C	0.30522100	3.22243900	2.22891900
H	-0.31035400	2.38635100	2.53154200
C	1.01439900	3.94136000	3.19390600
H	0.97853900	3.61955500	4.22913800
C	-0.24708400	-0.26180400	-2.75137300
C	-0.26980300	-3.94128500	1.74492600
H	-0.88153500	-3.80928600	2.62810200
C	-1.58026600	0.08462400	-3.00528800
C	1.49162200	-0.18354500	0.49470900
C	0.21105600	2.94049300	-1.93746200
C	-4.51358200	0.97022500	-0.75265300
C	-2.04813400	-1.78513300	3.25923500
H	-0.98081600	-1.59326600	3.23308800
C	-3.93624500	-5.16650700	-1.05849800
H	-4.45400700	-6.03333900	-0.65930800
C	-3.98101500	6.13631200	-0.55860600
H	-4.72783800	6.92389500	-0.57619400
C	1.36476700	-5.26688700	0.54324700
H	2.02726200	-6.12550300	0.49177000
C	-3.76586100	-5.02561100	-2.43771600
H	-4.15519200	-5.77898300	-3.11558400
C	1.42589400	-0.36633500	-0.92818700
C	-4.10021200	-1.94222000	4.52330600
H	-4.62330800	-1.91257300	5.47394800
C	-2.87102800	6.20608000	-1.40128800
H	-2.74877000	7.04903800	-2.07438500
C	-4.80638900	-2.15723900	3.34053400
H	-5.88342900	-2.29130400	3.36275900
C	-2.71712800	-1.75339000	4.47885400
H	-2.16372000	-1.56414400	5.39371500
C	0.61824400	-5.02066300	1.69412500
H	0.69795700	-5.68328000	2.55080700
C	-0.45480400	3.03664000	-3.16901100
H	-1.53159000	3.16306100	-3.20009700
C	1.60280500	2.77703800	-1.93125000
H	2.13273300	2.68596700	-0.99333400
C	3.35114300	-2.75749500	1.72461000
H	2.28064700	-2.82765400	1.56663400
C	0.37211500	-0.68924600	-4.05254400
H	1.06511400	0.07661100	-4.43358600
H	0.91259000	-1.63995100	-3.99214700
C	4.00797000	-1.52335500	1.62649200
C	3.42997500	-1.68030100	-1.55802400
H	3.25727500	-2.31274200	-0.69593600
C	4.03954800	1.26820100	0.65098800
C	4.08696100	-3.90519500	2.01291400
H	3.57708000	-4.85824300	2.08704500

C	0.26225000	2.99923600	-4.36552200
H	-0.26354200	3.09428500	-5.31066900
C	2.61695400	0.43094800	3.15146400
C	2.54618300	-0.68671400	-1.79883900
H	2.63240400	-0.07797000	-2.69454500
C	1.65106000	2.85246700	-4.34866500
H	2.20686200	2.83670800	-5.28124400
C	2.31919700	2.73819500	-3.12727700
H	3.39965900	2.63296800	-3.09611400
C	3.74739600	2.62320600	0.87975400
H	2.96457500	2.91335400	1.56876700
C	5.46858700	-3.82838200	2.20379500
H	6.03606600	-4.72681800	2.42533300
C	-1.86292700	-0.08375900	-4.47283800
H	-2.77813400	-0.64224700	-4.69175100
H	-1.91673900	0.88619700	-4.99063500
C	1.70520400	-0.39532000	3.83441800
H	1.24172300	-1.23728800	3.32999500
C	5.44713400	3.25611500	-0.72304100
H	5.99877800	4.02875500	-1.24960400
C	4.46158300	3.60917800	0.20047700
H	4.23437100	4.65186600	0.39057300
C	5.39150300	-1.43879700	1.84814400
H	5.90097600	-0.48268500	1.81002800
C	5.02896500	0.91493000	-0.28333600
H	5.26069800	-0.12245800	-0.48319700
C	7.02111400	-2.66038300	-3.62967700
H	7.94409600	-2.91349000	-4.14185800
C	6.11870200	-2.59544300	2.12309500
H	7.19021800	-2.53014600	2.28187700
C	1.41583800	-0.15564900	5.17385200
H	0.70394000	-0.78995700	5.69247800
C	2.05653600	0.88534600	5.85297300
H	1.83534300	1.06680800	6.89993300
C	3.28492700	1.44812700	3.84409200
H	4.02643700	2.06183600	3.34806900
C	5.72517000	1.90892600	-0.96692000
H	6.48717900	1.62512900	-1.68590400
C	3.00004000	1.67144300	5.19217200
H	3.52080400	2.46137300	5.72394900
C	6.20458200	-1.63691600	-4.12669700
H	6.49576800	-1.10018700	-5.02429800
C	6.64710700	-3.35510100	-2.47795200
H	7.27955600	-4.14768300	-2.09052400
C	5.45936000	-3.03151100	-1.82738100
H	5.16755400	-3.56382300	-0.92570000
C	5.02095000	-1.30926400	-3.47715600
H	4.39615100	-0.51419800	-3.87325800
C	4.62775000	-2.00565400	-2.31679100
C	0.26810100	0.17157000	1.03489000
H	0.16133400	0.36073200	2.09730700
H	-4.95579000	1.84913300	-0.28536900
C	-5.29421700	-0.22825700	-0.61592100
C	-5.20925800	-1.28489600	-1.54454000
C	-6.28492500	-0.27416700	0.39140700
C	-6.07156400	-2.36899300	-1.45197500
H	-4.49850000	-1.22289400	-2.35831000
C	-7.13726000	-1.36573400	0.48809900
H	-6.36602600	0.55001700	1.09365800
C	-7.02826800	-2.41574800	-0.43188500
H	-6.00336700	-3.17389900	-2.17446200
H	-7.89515400	-1.39718200	1.26417000
H	-7.70630800	-3.26133300	-0.36787800

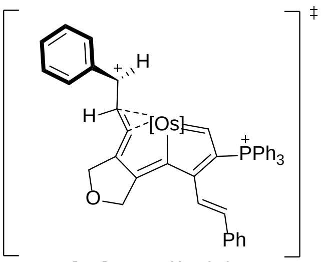

[Os] = $OsCl(PPh_3)_2$

TS2

E = -4699.12558746

Os	1.31022600	0.28850600	-0.29821700
Cl	2.28749200	0.70190300	-2.59327000
P	1.43580800	-2.14324100	-0.66727700
P	1.06297900	2.67040600	0.15875600
P	-3.31578500	0.29276000	-1.18234100
C	3.06715500	-2.87286700	-1.15022000
C	0.42361000	-2.84546200	-2.03366300
C	2.56548900	3.72475200	0.08273800
O	1.12978500	-0.87241200	4.67956000
C	4.66229900	4.29529900	-0.99625900
H	5.42878200	4.06921800	-1.73162300
C	-0.10055600	3.61219000	-0.90194500
C	2.39637900	0.05326400	1.35590000
C	3.57000100	-2.48793800	-2.41093500
H	3.03787500	-1.75183000	-3.00186300
C	0.16556300	-2.08184400	-3.18014000
H	0.51573800	-1.05854500	-3.23792000
C	3.42634200	0.45959100	0.48609300
H	3.79016200	1.48602100	0.64380000
C	0.88426900	-3.03794300	0.83476200
C	-0.79661500	4.72661800	-0.40756900
H	-0.70699900	5.01128000	0.63482700
C	-0.45521800	-3.43566700	0.96672700
H	-1.14347700	-3.30715400	0.14111500
C	0.05254700	-4.20054200	-2.01608300
H	0.29046000	-4.82099600	-1.15922300
C	-0.06930000	-0.09846100	1.24037900
C	4.75121800	-3.03813700	-2.90095500
H	5.11646300	-2.73769700	-3.87849200
C	2.66073000	4.89388900	0.85558900
H	1.88121400	5.14731300	1.56529800
C	-1.73100600	5.13018700	-2.60318000
H	-2.36732700	5.71498100	-3.26052400
C	3.56867100	3.43899000	-0.85734700
H	3.47464800	2.56822200	-1.49636200
C	-1.61365600	5.47568700	-1.25449000
H	-2.14850900	6.33614800	-0.86313900
C	-0.49142200	-2.65438500	-4.27157500
H	-0.66920600	-2.05888100	-5.16247300
C	-0.19252400	3.29360500	-2.26452000
H	0.39806700	2.48153800	-2.67213200
C	-1.00861400	4.04803000	-3.10700000
H	-1.07848100	3.78557800	-4.15716600
C	0.45796500	-0.31253400	2.52115100
C	3.76409100	-3.84270100	-0.41422600
H	3.37265900	-4.21350100	0.52291000
C	1.85792500	-0.20889400	2.58451100
C	-1.65610100	0.06069200	-0.48863500
C	0.43929800	2.89068600	1.86814500
C	4.49338900	-0.32487200	-0.12601800
C	1.73149700	-3.17288000	1.94625600
H	2.75278300	-2.81465800	1.90104000
C	-0.61697200	-4.76187700	-3.10261700
H	-0.89960900	-5.81011800	-3.07538100
C	4.76357400	5.44169800	-0.20770800
H	5.61440200	6.10697000	-0.31843700
C	5.45359100	-3.98336100	-2.14514300
H	6.36910300	-4.42101100	-2.53195900
C	-0.89977900	-3.98740000	-4.23050300

H	-1.41309600	-4.42817500	-5.07942000
C	-1.43137500	-0.18852600	0.90899500
C	-0.05767100	-4.15631900	3.24119800
H	-0.41832700	-4.59406500	4.16661100
C	3.75731600	5.74180800	0.71393400
H	3.82193900	6.64150900	1.31820800
C	-0.92098100	-3.99181100	2.15664200
H	-1.96157500	-4.29096500	2.23746400
C	1.26911500	-3.73907300	3.13427100
H	1.93982700	-3.83456000	3.98160900
C	4.95461300	-4.38301700	-0.90479700
H	5.47660100	-5.13761100	-0.32427900
C	1.32860000	2.93547700	2.95069600
H	2.39881700	2.95741700	2.77340500
C	-0.93768600	2.84449100	2.12831200
H	-1.64158400	2.77475500	1.30984100
C	-3.33284200	-2.43950200	-1.58966200
H	-2.25172300	-2.35816600	-1.55140000
C	-0.04768000	-0.63955600	3.89252200
H	-0.62842800	0.19269600	4.31498800
H	-0.66783500	-1.54176100	3.90975300
C	-4.13540200	-1.30211700	-1.40700100
C	-3.37231800	-1.46215900	1.77052400
H	-3.30414300	-2.09510500	0.89301000
C	-4.21915500	1.40755800	-0.08369100
C	-3.92869700	-3.67634900	-1.82657400
H	-3.30234100	-4.54995900	-1.97148100
C	0.84875800	2.97188100	4.26152900
H	1.54912400	3.02119600	5.08970300
C	-3.23041400	1.02264900	-2.83834000
C	-2.46590400	-0.47216500	1.90066800
H	-2.44652000	0.14312700	2.79760200
C	-0.52490400	2.95674900	4.50840900

H	-0.89473700	2.99829400	5.52827700
C	-1.41807500	2.88791200	3.43569800
H	-2.49052600	2.87724200	3.60952300
C	-3.76279800	2.73574300	-0.01320100
H	-2.95947700	3.08224600	-0.65460300
C	-5.32095700	-3.78275100	-1.88286300
H	-5.78471500	-4.74810600	-2.06012000
C	2.31084400	-0.43588100	3.99946000
H	3.07792200	-1.21416500	4.09571700
H	2.69952100	0.48705000	4.45600600
C	-2.53470200	0.31611700	-3.83447000
H	-2.04355400	-0.62173200	-3.60196500
C	-5.36011300	3.18408800	1.74591400
H	-5.80698200	3.87438000	2.45475000
C	-4.33832500	3.61907000	0.89719500
H	-3.98255800	4.64346100	0.94241700
C	-5.53035400	-1.40107800	-1.50710300
H	-6.15560800	-0.51957700	-1.41426300
C	-5.24070400	0.97254900	0.77096600
H	-5.57489900	-0.05638500	0.75625800
C	-6.69383100	-2.46144500	4.25157800
H	-7.54878800	-2.71941300	4.86869100
C	-6.11830800	-2.64568300	-1.73093500
H	-7.19878500	-2.72499000	-1.79442800
C	-2.49770700	0.80543300	-5.13644000
H	-1.95207400	0.26141600	-5.90121700
C	-3.18025400	1.98117700	-5.46322400
H	-3.16086300	2.35405300	-6.48254000
C	-3.93096400	2.18914700	-3.17195800
H	-4.50032900	2.72585300	-2.42369400
C	-5.80709100	1.86257300	1.68232000
H	-6.59017300	1.51477800	2.34810000
C	-3.90408100	2.66159200	-4.48442100

H	-4.45232100	3.56310700	-4.73871200
C	-5.86425400	-1.39438200	4.61650000
H	-6.07728700	-0.82677300	5.51723700
C	-6.42040800	-3.19099200	3.09343800
H	-7.06313800	-4.01714000	2.80548100
C	-5.32085100	-2.85717600	2.30610200
H	-5.11225500	-3.41609400	1.39687200
C	-4.76729600	-1.05940400	3.83124400
H	-4.13530600	-0.22567000	4.12195200
C	-4.47546600	-1.79025500	2.66359700
C	-0.49602400	0.31129800	-1.21337700
H	-0.58881900	0.58607800	-2.25926100
H	4.36153500	-0.62033700	-1.16388300
C	5.72908900	-0.61827900	0.46067700
C	6.01139700	-0.30707600	1.82567600
C	6.72023300	-1.27842800	-0.32754400
C	7.23415300	-0.64070400	2.36846300
H	5.25128600	0.18860200	2.41995800
C	7.94262300	-1.60989800	0.22971900
H	6.49345000	-1.51577800	-1.36064700
C	8.19731100	-1.28999100	1.57052700
H	7.46094200	-0.40854100	3.40353100
H	8.70103800	-2.10977100	-0.36296800
H	9.15835900	-1.54790500	2.00601200

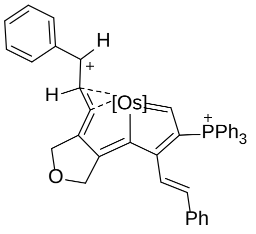

[Os] = $OsCl(PPh_3)_2$

Int3

E = -4699.17729700

Os	-1.34117200	0.11102900	0.09874700
Cl	-2.49098700	0.35844300	2.25137300
P	-1.33182700	-2.35731100	0.27164400
P	-1.21992600	2.55429900	-0.18115400
P	3.15289700	0.33569100	1.49370400
C	-2.90796700	-3.26170300	0.55658200
C	-0.34629000	-3.07669300	1.64944900
C	-2.78902000	3.50950500	-0.16361800
O	-0.50250000	-0.61661600	-4.92115400
C	-5.01243100	3.87339100	0.73449700
H	-5.83544800	3.53565200	1.35756500
C	-0.21258900	3.47975500	1.04446700
C	-2.17340800	0.02701100	-1.71550700
C	-3.81344900	-2.74902100	1.49817400
H	-3.63743000	-1.77871600	1.94657600
C	-0.35000400	-2.44551100	2.90150500
H	-0.86357200	-1.49956700	3.02993000
C	-3.53247100	0.26265600	-1.29629800
H	-3.86653400	1.29594800	-1.24521900
C	-0.62274400	-3.06837700	-1.25802900
C	0.41987800	4.68207700	0.68754100
H	0.38373000	5.03823700	-0.33565200
C	0.76086900	-3.26306800	-1.38430800
H	1.41327900	-3.09442400	-0.53619800
C	0.23246000	-4.35013400	1.52689300
H	0.19645000	-4.87903000	0.58144300
C	0.24964300	-0.07176500	-1.32401300
C	-4.93891100	-3.48752600	1.85992900
H	-5.63337600	-3.07996600	2.58790000
C	-2.86925400	4.74775300	-0.82332600
H	-2.03754700	5.10851500	-1.41810300
C	1.15571600	4.98918700	2.97258000
H	1.68818500	5.57214100	3.71785700
C	-3.86663200	3.08282900	0.62636900

H	-3.80284500	2.15099900	1.17449700
C	1.10503600	5.42727700	1.64660700
H	1.58875600	6.35639800	1.35985800
C	0.25592600	-3.05984500	3.99918800
H	0.23172000	-2.57033600	4.96858200
C	-0.18616400	3.06390500	2.38277200
H	-0.73017300	2.17849900	2.68740900
C	0.49872600	3.81433400	3.33857600
H	0.51471400	3.47656900	4.36897400
C	-0.10981500	-0.19572900	-2.67128700
C	-3.13992000	-4.52550500	-0.00817400
H	-2.43628900	-4.95181200	-0.71332700
C	-1.48945100	-0.13672200	-2.88897500
C	1.62195300	0.04914700	0.56497200
C	-0.45370100	2.94728700	-1.79808800
C	-4.41571200	-0.75512100	-1.02287300
C	-1.43471100	-3.26065600	-2.38622700
H	-2.50569300	-3.10046600	-2.32190100
C	0.84308200	-4.95474200	2.62560400
H	1.28188800	-5.94247400	2.51873400
C	-5.09582500	5.09062400	0.05796600
H	-5.98796600	5.70324800	0.14312400
C	-5.17697900	-4.73666600	1.28342900
H	-6.05425100	-5.31041700	1.56644000
C	0.86701300	-4.30673800	3.86231200
H	1.33499600	-4.78255800	4.71838100
C	1.57109300	-0.09599900	-0.86530300
C	0.49506400	-3.87241600	-3.70911100
H	0.92427800	-4.18610500	-4.65530200
C	-4.01991500	5.52668500	-0.71867600
H	-4.07239900	6.47836200	-1.23832300
C	1.31469500	-3.66953700	-2.59695200
H	2.38738500	-3.81932100	-2.67338700
C	-0.87992500	-3.66086400	-3.60183900
H	-1.52143700	-3.80528100	-4.46500000
C	-4.27817700	-5.25094600	0.34660100
H	-4.45217200	-6.22538400	-0.09950200
C	-1.23518500	3.06801100	-2.95539100
H	-2.31755600	3.03274600	-2.88896300
C	0.94310900	2.98844200	-1.91774700
H	1.56648700	2.87418600	-1.04087500
C	3.38490700	-2.42127700	1.63169200
H	2.31611900	-2.43976700	1.44617700
C	0.56372100	-0.36658800	-3.99944900
H	1.11458300	0.54278200	-4.28310000
H	1.25569100	-1.21410600	-4.02028100
C	4.07804900	-1.20209600	1.68383300
C	3.72399800	-1.08734900	-1.59681400
H	3.67064500	-1.76907400	-0.75528800
C	4.07884800	1.60667600	0.60623800
C	4.07677900	-3.61551100	1.82275100
H	3.53715500	-4.55514200	1.78165100
C	-0.63142000	3.25607100	-4.20059400
H	-1.24957900	3.36126400	-5.08678300
C	2.80307400	0.91097300	3.17273200
C	2.71143300	-0.21084900	-1.76504900
H	2.69032900	0.43962400	-2.63551500
C	0.75869700	3.32345000	-4.30541000
H	1.22364000	3.48388600	-5.27320000
C	1.54555100	3.18547000	-3.15854800
H	2.62882200	3.24127800	-3.22189000
C	3.51952100	2.89657000	0.58041100
H	2.62397700	3.12484900	1.14795300
C	5.45163500	-3.59767500	2.07143400
H	5.98906800	-4.53010600	2.21297300
C	-1.77240800	-0.25905900	-4.35877300

H	-2.49490300	-1.04450300	-4.60354100
H	-2.13341100	0.68856100	-4.78436200
C	2.00929900	0.10175200	4.00311100
H	1.58716500	-0.82438900	3.63011900
C	5.26531900	3.61222500	-0.93209900
H	5.72889000	4.39181500	-1.52869300
C	4.11762000	3.89429900	-0.18552100
H	3.68193600	4.88808500	-0.19770900
C	5.44974900	-1.18038800	1.97715900
H	5.98201300	-0.23913800	2.06517200
C	5.22914600	1.32548300	-0.14276100
H	5.65176000	0.32946900	-0.15803700
C	7.31378700	-1.55051400	-3.84817100
H	8.23610700	-1.67236900	-4.40738600
C	6.13408900	-2.38149200	2.15819800
H	7.19777900	-2.36656000	2.37266700
C	1.78845300	0.47223600	5.32629700
H	1.16636600	-0.14942500	5.96263700
C	2.38624000	1.62700700	5.84052300
H	2.22436000	1.90560100	6.87710100
C	3.41658200	2.05824400	3.69353400
H	4.05559700	2.67413700	3.07315800
C	5.81810000	2.32999900	-0.90920400
H	6.70218200	2.10227700	-1.49564800
C	3.20787400	2.40829500	5.02773700
H	3.68936600	3.29426600	5.42917800
C	6.38656100	-0.57438200	-4.23314500
H	6.59193200	0.05868000	-5.09087600
C	7.05045300	-2.36585200	-2.74612900
H	7.76775700	-3.12276000	-2.44463500
C	5.86407800	-2.20785600	-2.03400500
H	5.65930300	-2.83723100	-1.17106100
C	5.20322300	-0.41399300	-3.52252200
H	4.49495200	0.35008400	-3.82813800
C	4.92139900	-1.23256100	-2.41158000
C	0.36921000	0.15929800	1.13069700
H	0.25867300	0.31235900	2.19792900
H	-4.05310200	-1.77282400	-1.12540400
C	-5.79404300	-0.63029200	-0.63538600
C	-6.40930900	0.61549200	-0.37319600
C	-6.55966100	-1.81182700	-0.51577600
C	-7.74342700	0.66870700	0.00013100
H	-5.84022000	1.53424300	-0.45573400
C	-7.89708800	-1.75163300	-0.14746000
H	-6.08921300	-2.77028300	-0.70998600
C	-8.49000800	-0.51234000	0.11304500
H	-8.21172100	1.62672500	0.20213800
H	-8.47933400	-2.66321200	-0.06129400
H	-9.53506200	-0.46359900	0.40315800

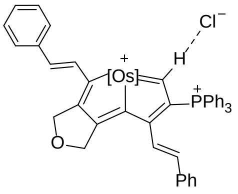

[Os] = $OsCl(PPh_3)_2$

Int4

E = -5159.63108255

Os	-1.07249200	0.11499900	0.38176400
Cl	-2.49527900	0.69158700	2.22715300
P	-0.92021300	-2.15917400	1.32609700
P	-1.53171800	2.32001500	-0.55067500
P	3.50368000	1.06295700	0.50581000
C	-2.49942000	-3.05822700	1.05670100
C	-0.68417700	-2.30040100	3.12733900
C	-0.43793100	2.87257300	-1.92527700
O	-1.17077900	-2.70681400	-3.88016200

C	0.61784100	2.43396900	-4.07222300
H	0.76566500	1.77731100	-4.92484000
C	-3.23052900	2.38705600	-1.23598200
C	-2.35150000	-0.71798400	-1.00826500
C	-3.58402700	-2.66745000	1.86197400
H	-3.43429000	-1.93795100	2.65025000
C	-0.09835100	-1.26181000	3.85319900
H	0.13062900	-0.30697400	3.40272600
C	-3.74745600	-0.68726400	-0.65602700
H	-3.98919000	-0.37183800	0.34709400
C	0.45043000	-3.20394600	0.67086200
C	-3.50039300	2.28907100	-2.60474000
H	-2.69144400	2.26985800	-3.32540700
C	1.53766200	-3.50127200	1.50655900
H	1.53578500	-3.16343200	2.53517200
C	-0.97902200	-3.51819100	3.76537200
H	-1.43459600	-4.33030100	3.20767300
C	0.18011500	-0.50543900	-1.23869500
C	-4.85089800	-3.20323700	1.64436100
H	-5.67993100	-2.89249200	2.27253900
C	0.19303100	4.12401200	-1.89587800
H	0.02107400	4.79577000	-1.06401100
C	-5.87729300	2.27619200	-2.14630700
H	-6.90295600	2.21731900	-2.49687300
C	-0.21829100	2.03548000	-3.03140100
H	-0.71284400	1.07598300	-3.08303900
C	-4.82112700	2.22647300	-3.05506900
H	-5.02080300	2.15113500	-4.12013400
C	-6.17669000	-0.99636300	-1.15405400
C	0.21025100	-1.43610900	5.20367000
H	0.67284100	-0.61299800	5.73900200
C	-4.29789400	2.44242400	-0.32335400
H	-4.09508300	2.51383500	0.73961100
C	-5.61186300	2.39562700	-0.78053100
H	-6.43054600	2.43322300	-0.06922300
C	-0.44795700	-1.19384800	-2.27966600
C	-2.70681800	-4.00904400	0.05126000
H	-1.88449400	-4.36468600	-0.55392700
C	-1.82850200	-1.30314400	-2.15733000
C	1.89080100	0.37566600	0.07558000
C	-1.50598300	3.70932000	0.63265400
C	-4.76573600	-1.00934900	-1.49773800
H	-4.54687100	-1.27460600	-2.52720500
C	0.47879700	-3.67709700	-0.65175700
H	-0.32856200	-3.45218800	-1.33280400
C	-0.68139000	-3.68352800	5.11509400
H	-0.91502800	-4.62378700	5.60582500
C	1.25716500	3.67493100	-4.02295600
H	1.90587700	3.98780400	-4.83593800
C	-5.05841200	-4.12525400	0.61665800
H	-6.05065100	-4.52766000	0.43770500
C	-0.08155500	-2.64404900	5.83554400
H	0.15063500	-2.77986500	6.88796700
C	1.56755400	-0.34557800	-1.12514900
C	2.62543200	-4.72804500	-0.27334700
H	3.46332600	-5.31116300	-0.64152900
C	1.04180900	4.51782100	-2.93278400
H	1.52624400	5.48854400	-2.88725700
C	2.61898200	-4.24816900	1.03497000
H	3.45072700	-4.45783900	1.70110200

C	-6.64952800	-0.68095100	0.13653200
H	-5.94754400	-0.44856300	0.92933000
C	1.54865900	-4.43979500	-1.11373800
H	1.54177000	-4.80533800	-2.13630600
C	-3.98239800	-4.53303400	-0.17021900
H	-4.12719100	-5.27125400	-0.95372000
C	-0.80453700	3.61631800	1.83819300
H	-0.28739400	2.71526300	2.13206000
C	-2.16696700	4.90818400	0.30617900
H	-2.72469500	4.98747900	-0.62180200
C	3.58958700	-0.89615200	2.44800700
H	2.52101300	-0.96164000	2.29944800
C	-0.02645200	-1.92561000	-3.52507500
H	0.24074300	-1.22322000	-4.33523600
H	0.81990900	-2.60098800	-3.37166900
C	-7.11819100	-1.29344500	-2.15705900
H	-6.76621300	-1.53656400	-3.15641000
C	4.35292300	0.00479900	1.69325800
C	3.53083400	-1.73782700	-1.54471500
H	3.59961700	-1.85593100	-0.46514100
C	4.46382300	1.22342600	-1.02856300
C	4.20686500	-1.68833500	3.41291100
H	3.60587100	-2.36972100	4.00720600
C	-8.93605300	-0.95397100	-0.60638500
H	-10.00038900	-0.93541200	-0.39254200
C	-8.48384600	-1.27159300	-1.88863100
H	-9.19453600	-1.50155800	-2.67664600
C	-0.75332200	4.70650200	2.70757700
H	-0.19465600	4.60531700	3.63236500
C	3.33193400	2.74210200	1.14644500
C	2.53049500	-0.97122200	-2.02179400
H	2.37539100	-0.85564800	-3.09041200
C	-1.40390100	5.89416400	2.37928500
H	-1.36842900	6.74189000	3.05752300
C	-2.11090200	5.99441600	1.17533500
H	-2.62411500	6.91627100	0.91800700
C	-8.01247500	-0.66072300	0.40391700
H	-8.36135500	-0.41466900	1.40252800
C	3.97009900	2.11055700	-1.99802300
H	3.09008000	2.71362300	-1.80410400
C	5.58108200	-1.58344700	3.63143200
H	6.05940400	-2.19960200	4.38672000
C	-2.35202700	-2.14061200	-3.29273100
H	-3.00244600	-2.95866100	-2.96407600
H	-2.90121200	-1.54147300	-4.03819500
C	4.24599200	3.22941500	2.08798800
H	4.99439800	2.57646100	2.52088700
C	5.73762300	1.43786800	-3.50600000
H	6.22893200	1.51642900	-4.47107300
C	4.60276100	2.20700300	-3.23401900
H	4.20804800	2.88695500	-3.98213800
C	5.73351000	0.12485800	1.92690500
H	6.32965600	0.83716200	1.36615700
C	5.59929900	0.45337400	-1.30365100
H	5.96709800	-0.26516200	-0.58252900
C	6.60739100	-3.81377100	-3.65336500
H	7.40101700	-4.33696500	-4.17795300
C	6.34289200	-0.67516400	2.89133000
H	7.40968200	-0.58389900	3.06954200
C	4.15409200	4.55216400	2.51065800

H	4.85073600	4.92603700	3.25423900
C	3.15719000	5.38655800	2.00078700
H	3.08234900	6.41384400	2.34402500
C	2.33352900	3.57558800	0.63408000
H	1.61012500	3.19007800	-0.07108800
C	6.23642400	0.56665500	-2.53842000
H	7.10358400	-0.05065000	-2.74946500
C	2.24764200	4.89674700	1.06387300
H	1.45434500	5.53460300	0.68860500
C	5.64070100	-3.10286200	-4.37353400
H	5.68564300	-3.07439800	-5.45822300
C	6.54583300	-3.84814000	-2.25927500
H	7.29242900	-4.39743700	-1.69348200
C	5.52373600	-3.17616500	-1.59237300
H	5.47353500	-3.19928500	-0.50664500
C	4.62278500	-2.43017700	-3.70724400
H	3.88759700	-1.87234400	-4.27890500
C	4.54807700	-2.45486300	-2.30323800
C	0.75115800	0.57398000	0.83268700
H	0.80078900	1.02643000	1.89586000
Cl	1.55894300	1.65849100	3.58825800

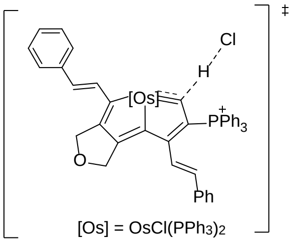

TS3

E = -5159.62485762

Os	1.00879700	-0.10085800	0.39921000
Cl	2.64373100	-0.60072600	2.15073700
P	0.95173400	2.20051600	1.25939200
P	1.47352400	-2.32477300	-0.46347200
P	-3.52456100	-1.05153400	0.51068900
C	2.50592500	3.09388700	0.84285500
C	0.88585900	2.44950300	3.06842700
C	0.35289500	-2.95635900	-1.78520900
O	1.16728400	2.59064800	-3.93307300
C	-0.72334300	-2.62191700	-3.94183700
H	-0.90438800	-1.99203300	-4.80803500
C	3.14807500	-2.43573000	-1.20605900
C	2.36279200	0.65773600	-1.01677100
C	3.63753500	2.75016900	1.60368100
H	3.54004500	2.05587600	2.43060500
C	0.60799400	1.40014800	3.94438900
H	0.47914800	0.39752100	3.57068000
C	3.77220600	0.62440200	-0.73137500
H	4.04548600	0.32254600	0.26825200
C	-0.46347200	3.22514800	0.66998600
C	3.36820400	-2.34738700	-2.58477800
H	2.53368200	-2.31341700	-3.27479700
C	-1.49643500	3.55033800	1.56257900
H	-1.42979600	3.24300800	2.59991300
C	1.08874600	3.74527000	3.57724100
H	1.31465300	4.56522400	2.90235500
C	-0.21522300	0.49642800	-1.22842600
C	4.88640600	3.27965400	1.28890600
H	5.75223700	3.00025300	1.88096700
C	-0.22481200	-4.23237300	-1.72208500
H	-0.02807100	-4.87482400	-0.87296700
C	5.76059000	-2.37668000	-2.21866000
H	6.77279000	-2.33643900	-2.60880400

C	0.08838400	-2.15679800	-2.90905600
H	0.53125600	-1.17392000	-2.98009000
C	4.67078200	-2.31125900	-3.08606700
H	4.83009400	-2.24227500	-4.15836800
C	6.18779000	0.90825600	-1.30009500
C	0.51280400	1.63866900	5.31729600
H	0.28927300	0.81164100	5.98379400
C	4.24909800	-2.50777600	-0.33590100
H	4.08771500	-2.56620600	0.73435000
C	5.54541800	-2.48552600	-0.84373100
H	6.38969100	-2.53191600	-0.16337800
C	0.43438300	1.13806500	-2.28443900
C	2.64995600	3.99544700	-0.21733600
H	1.79481700	4.31590700	-0.79540000
C	1.81904500	1.22161500	-2.17338100
C	-1.92631700	-0.34521500	0.11131900
C	1.50043100	-3.66096200	0.78447400
C	4.76797000	0.92222100	-1.60836200
H	4.52746300	1.16500500	-2.63832700
C	-0.57895500	3.64972800	-0.66429900
H	0.18257100	3.39779800	-1.38707300
C	0.99975600	3.97825000	4.94575500
H	1.15933200	4.98000800	5.33356700
C	-1.29474700	-3.89378500	-3.86674400
H	-1.91824900	-4.26154700	-4.67643000
C	5.02916100	4.15115900	0.20816900
H	6.00665400	4.54906100	-0.04638200
C	0.70830000	2.92363100	5.81876000
H	0.64019700	3.10843700	6.88692600
C	-1.60520800	0.35005300	-1.10797400
C	-2.69602000	4.71840800	-0.18641100
H	-3.55490100	5.29226500	-0.51963500
C	-1.04846000	-4.69397400	-2.75115900
H	-1.48579300	-5.68551900	-2.68161200
C	-2.60392800	4.28652200	1.13542400
H	-3.39016800	4.52818500	1.84430000
C	6.68990300	0.62391300	-0.01310400
H	6.00513500	0.41211800	0.80025000
C	-1.67731600	4.39582900	-1.08431000
H	-1.73945700	4.72018300	-2.11871800
C	3.90708600	4.51450700	-0.53517200
H	4.00120500	5.21432300	-1.36063200
C	0.92525300	-3.47581100	2.04444900
H	0.47720400	-2.53097100	2.30751900
C	2.09949700	-4.89514700	0.47459800
H	2.56908200	-5.04306800	-0.49297400
C	-3.81717800	1.07109600	2.25217300
H	-2.76050900	1.23200300	2.08074000
C	0.01710400	1.83293800	-3.55279400
H	-0.25754200	1.10678800	-4.33997800
H	-0.82457600	2.51996600	-3.42364200
C	7.10759200	1.17859000	-2.33042700
H	6.73335700	1.39860700	-3.32715800
C	-4.48295400	0.03203600	1.59024800
C	-3.59248100	1.70706900	-1.53851800
H	-3.64225800	1.86062300	-0.46322400
C	-4.43925900	-1.36109300	-1.02691700
C	-4.51884000	1.89567200	3.12954800
H	-3.99652500	2.69317100	3.64841900
C	8.96005400	0.87567100	-0.81308500

H	10.02894500	0.86154800	-0.62258800
C	8.47909700	1.16176600	-2.09222700
H	9.17204800	1.37162200	-2.90150800
C	0.93225300	-4.50907700	2.98170100
H	0.47716600	-4.34345600	3.95299100
C	-3.29580600	-2.65616100	1.31229300
C	-2.58237000	0.94812200	-2.00884900
H	-2.43891700	0.80941300	-3.07697700
C	1.51551100	-5.73511000	2.66634500
H	1.52530600	-6.53905300	3.39665000
C	2.09947200	-5.92769900	1.40947400
H	2.56341800	-6.87795200	1.16235200
C	8.05872800	0.60828000	0.22396100
H	8.42942100	0.38651900	1.22049600
C	-3.92485500	-2.32553700	-1.90655700
H	-3.04088400	-2.89844800	-1.65068700
C	-5.88128300	1.68825500	3.34808700
H	-6.42548400	2.33132000	4.03297700
C	2.34219500	2.01086600	-3.34533100
H	3.01901800	2.82146100	-3.05551100
H	2.86141900	1.37377400	-4.08126600
C	-4.17430900	-3.08766400	2.31436400
H	-4.95441100	-2.43323600	2.68405700
C	-5.68371900	-1.81089200	-3.48580400
H	-6.16512100	-1.98386300	-4.44359800
C	-4.54651900	-2.54193500	-3.13349800
H	-4.13972700	-3.28454900	-3.81152600
C	-5.85351700	-0.18210500	1.81578000
H	-6.37607700	-0.98756400	1.30993400
C	-5.57799400	-0.62769700	-1.38221800

H	-5.96098700	0.14687800	-0.73036800
C	-6.76814700	3.64015400	-3.63639700
H	-7.58600500	4.12870500	-4.15718900
C	-6.54782500	0.64994000	2.69132200
H	-7.60711200	0.48652200	2.86266900
C	-4.01239200	-4.35301700	2.87244300
H	-4.68426400	-4.68146100	3.65913500
C	-2.98109200	-5.18762400	2.43642600
H	-2.85323900	-6.16936400	2.88207200
C	-2.25925700	-3.49081100	0.87884700
H	-1.56304500	-3.14712100	0.12645700
C	-6.19873400	-0.85620000	-2.60865700
H	-7.06899300	-0.26930400	-2.88377700
C	-2.10566200	-4.75562000	1.44028000
H	-1.28726500	-5.39101700	1.11972300
C	-5.82737500	2.89227300	-4.35335800
H	-5.91697900	2.79967400	-5.43170900
C	-6.65087500	3.75419000	-2.25018300
H	-7.37828500	4.33129500	-1.68699700
C	-5.59839400	3.12619600	-1.58771000
H	-5.50465300	3.21078900	-0.50788700
C	-4.77889400	2.26300300	-3.69171000
H	-4.06587600	1.67286500	-4.25910700
C	-4.64538700	2.37174800	-2.29569800
C	-0.76366900	-0.51238200	0.86006200
H	-0.95572600	-0.92972000	2.35881900
Cl	-1.46839000	-1.28367400	3.66390500

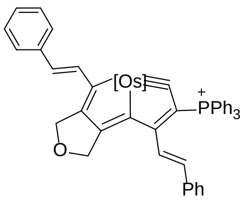

[Os] = OsCl(PPh3)2

3

E = -4698.82014630

Os	-0.99456800	-0.01634600	0.33990200
Cl	-2.70008200	0.14202400	2.12987800
P	-1.01558500	-2.38125700	0.87452800
P	-1.29446200	2.35064500	0.03934100
P	3.44994400	0.55611000	0.99001900
C	-2.53528800	-3.17560400	0.23124500
C	-1.00537200	-2.69951700	2.67630000
C	-2.90629000	2.74881000	-0.74335500
O	-1.16265300	-1.36737000	-4.60134000
C	-5.32149900	2.77213200	-0.50844400
H	-6.20290800	2.66081900	0.11490600
C	-1.34237000	3.31788700	1.59109400
C	-2.36162100	-0.38542500	-1.22257600
C	-3.75092200	-2.98935300	0.90935600
H	-3.76274800	-2.48324500	1.86792800
C	-0.29812900	-1.83272500	3.52088000
H	0.15951400	-0.94196800	3.10585400
C	-3.77566800	-0.39234300	-0.97018300
H	-4.06085000	-0.34040400	0.07081400
C	0.36481700	-3.45220900	0.27805400
C	-1.93148700	4.59308000	1.59284900
H	-2.35682900	4.99408500	0.67834200
C	0.95137500	-4.41104600	1.11908800
H	0.60926700	-4.52219300	2.14048600
C	-1.62419100	-3.83472900	3.21772300
H	-2.17271100	-4.51376400	2.57346300
C	0.73720800	0.18224700	0.91452300
C	0.23926500	-0.21961400	-1.36480600
C	-4.94520800	-3.42396200	0.33674100
H	-5.88085600	-3.27059300	0.86554000
C	-3.04795100	3.02999800	-2.10639500
H	-2.17702200	3.15826800	-2.73708500
C	-1.46366600	4.81461400	3.95189000
H	-1.52464100	5.38763800	4.87272800
C	-4.05717000	2.62297200	0.05460900
H	-3.95939300	2.38725800	1.10801500
C	-1.98492300	5.34053500	2.76687200
H	-2.44463900	6.32434500	2.76023300
C	-6.18856000	-0.39423900	-1.61227800
C	-0.20856600	-2.10239400	4.88474100
H	0.33279600	-1.41964600	5.53396300
C	-0.80263600	2.80467200	2.77507300
H	-0.34945500	1.82147600	2.77568900
C	-0.87464600	3.55080500	3.95180900
H	-0.48030400	3.13398800	4.87242700
C	-0.41785700	-0.53066600	-2.56311200
C	-2.53707500	-3.80212600	-1.02213400
H	-1.60726600	-3.96433200	-1.55552500
C	-1.80271300	-0.62049400	-2.48839100
C	1.92851500	0.12283700	0.20026200
C	-0.01746600	3.21569500	-0.96503500
C	-4.76510700	-0.41429000	-1.90350000
H	-4.51660900	-0.40855400	-2.95965900
C	0.83361500	-3.34177700	-1.03998700
H	0.38728500	-2.62039800	-1.70622100

C	-1.53905000	-4.09621900	4.58592600
H	-2.02722200	-4.97496900	4.99674700
C	-5.45811600	3.03012500	-1.87338600
H	-6.44626500	3.12119000	-2.31369300
C	-4.94450700	-4.02661100	-0.92172900
H	-5.87914100	-4.34577500	-1.37278500
C	-0.83050600	-3.23163700	5.42140600
H	-0.76839700	-3.43439900	6.48654100
C	1.63361300	-0.15479200	-1.18771900
C	2.44912300	-5.09905700	-0.65761600
H	3.25246800	-5.73505500	-1.01765900
C	-4.31995300	3.16234700	-2.66797800
H	-4.41623700	3.38030800	-3.72785100
C	1.97445900	-5.23537000	0.64786300
H	2.39822200	-5.98824600	1.30688800
C	-6.70140600	-0.40160600	-0.29804000
H	-6.02264000	-0.43277400	0.54695800
C	1.87419900	-4.14767900	-1.50119600
H	2.22922300	-4.03186600	-2.52066600
C	-3.73819300	-4.21807900	-1.59702600
H	-3.72725600	-4.70003000	-2.57044500
C	0.22777700	2.81551600	-2.28901200
H	-0.30816600	1.97070300	-2.70050200
C	0.71764300	4.28846300	-0.43916700
H	0.55929200	4.60180600	0.58574900
C	3.81795100	-2.16883400	1.46938300
H	2.95859100	-2.33089300	0.82689000
C	0.01004500	-0.85798800	-3.96863700
H	0.38364100	0.03719400	-4.49934600
H	0.79910400	-1.61819400	-4.01895600

C	-7.10079200	-0.34872500	-2.68334800
H	-6.71853500	-0.34034100	-3.70110400
C	4.26586800	-0.87009400	1.74300500
C	3.79768600	-0.98658800	-2.05091600
H	3.96676500	-1.48045900	-1.09729000
C	4.57588100	1.34562300	-0.18198600
C	4.47363000	-3.25593900	2.04959900
H	4.12307700	-4.25861000	1.83698800
C	-8.96509800	-0.31434700	-1.15030900
H	-10.03532400	-0.28240100	-0.96945600
C	-8.47389500	-0.30818900	-2.45719400
H	-9.16036800	-0.27185000	-3.29788200
C	1.14379800	3.50406000	-3.08309400
H	1.30210800	3.19470800	-4.11230300
C	3.06372100	1.68925900	2.34830100
C	2.62166100	-0.34823500	-2.23455300
H	2.38457800	0.07490900	-3.20743200
C	1.84361100	4.59452500	-2.56144200
H	2.53965700	5.14671000	-3.18628900
C	1.64167000	4.97179000	-1.23334700
H	2.19177300	5.80987300	-0.81514600
C	-8.07208400	-0.36228100	-0.07350900
H	-8.45038100	-0.36705900	0.94465100
C	4.05638100	2.31237300	-1.05688500
H	3.01113500	2.59509000	-1.00677900
C	5.55993700	-3.05111200	2.90011400
H	6.06414100	-3.90151100	3.34909000
C	-2.32903500	-1.01832500	-3.84211700
H	-2.99681500	-1.88721600	-3.80581700
H	-2.86867400	-0.19438400	-4.33892400

C	2.64753100	1.15626300	3.57832200
H	2.59283700	0.08152500	3.71769900
C	6.21666000	2.47708800	-2.13241100
H	6.85043600	2.90625700	-2.90214400
C	4.88127000	2.87626200	-2.02477700
H	4.47299900	3.61640500	-2.70411300
C	5.34720800	-0.66222100	2.61666600
H	5.67232100	0.34637600	2.85450000
C	5.91342100	0.94531900	-0.28930900
H	6.30985100	0.17292900	0.35917900
C	7.16691600	-1.22936100	-4.65937100
H	8.03708000	-1.28443600	-5.30649500
C	5.99432800	-1.75384200	3.18873900
H	6.83046600	-1.59414400	3.86232400
C	2.32088900	2.01343300	4.62644500
H	2.00575300	1.60155800	5.58032200
C	2.40209600	3.39659500	4.45044800
H	2.14936200	4.06255200	5.26937400
C	3.13662200	3.07694400	2.16988300
H	3.45925600	3.49231600	1.22193300
C	6.73126700	1.51597600	-1.26395900
H	7.76150500	1.18882600	-1.35852100
C	2.80332100	3.92625200	3.22344800
H	2.86277700	5.00151600	3.08775600
C	6.09627600	-0.39336600	-4.99339700
H	6.13746400	0.20404800	-5.89951700
C	7.11288800	-1.99019500	-3.48988500
H	7.94065000	-2.64110200	-3.22422200
C	5.99631400	-1.91303000	-2.66178700
H	5.95493700	-2.49902900	-1.74658500
C	4.98206600	-0.31531600	-4.16563700
H	4.17232000	0.36114600	-4.42021600
C	4.91154600	-1.07822200	-2.98529000

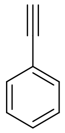

E = -308.411455474

C	0.59356800	-0.00001400	-0.00000600
C	-0.11924600	1.21315700	-0.00000500
C	-1.51145100	1.20864000	0.00000400
C	-2.21156400	0.00000600	0.00000500
C	-1.51147300	-1.20862800	0.00000100
C	-0.11925600	-1.21316100	-0.00000200
H	0.43067600	2.14871000	-0.00000700
H	-2.05202100	2.15082200	0.00000800
H	-3.29776300	0.00002200	0.00001000
H	-2.05203800	-2.15081300	0.00000400
H	0.43063600	-2.14873300	-0.00000400
C	2.02176100	-0.00000400	-0.00001300
C	3.23152000	0.00000900	-0.00000200
H	4.29735800	-0.00003200	0.00009200

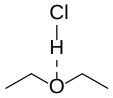

E = -694.483731763

O	-0.47053200	0.04979700	-0.27370200
C	-1.21778000	-1.06554100	0.23277100
H	-0.71151500	-1.95032400	-0.16377600
H	-1.13547600	-1.09458700	1.32883800
C	-0.65336900	1.26975900	0.45572100

C	0.16408900	2.35657000	-0.21913000
H	0.02881000	3.30708900	0.30757900
H	1.22906600	2.10564300	-0.21095600
H	-0.15485600	2.48506100	-1.25805500
C	-2.67371500	-1.03689400	-0.21358200
H	-2.73475600	-0.99185100	-1.30539500
H	-3.18472000	-1.94477200	0.12579000
H	-3.21230400	-0.17760300	0.19894600
H	-0.33404800	1.11924300	1.49799000
H	-1.71867600	1.53303900	0.46369600
H	1.14617300	-0.39223600	-0.20178900
Cl	2.40183600	-0.79649700	-0.00752500

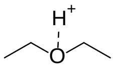

E = -234.002032488

H	-0.44874800	1.46783600	-0.22460100
O	-0.04719900	0.57497000	-0.26633400
C	1.38938800	0.61146500	0.24040200
H	1.78752600	1.50619000	-0.23941100
H	1.32547200	0.74762900	1.32257300
C	-1.00210300	-0.43686600	0.34045800
C	-2.39218800	-0.11691800	-0.14825300
H	-3.07439100	-0.87120700	0.25855500
H	-2.74129500	0.85914000	0.20814100
H	-2.45442300	-0.15905100	-1.23904100
C	2.09916300	-0.64748600	-0.18361600
H	2.04852900	-0.78726500	-1.26676100
H	3.15345900	-0.53772200	0.09391800
H	1.72035100	-1.53702400	0.32615400
H	-0.88025100	-0.36047600	1.42363700
H	-0.62420000	-1.38898600	-0.02644200

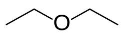

E = -233.672873141

O	0.07588100	-0.58448400	-0.30487600
C	-1.24759200	-0.62555800	0.20072600
H	-1.69508900	-1.52562900	-0.23425900
H	-1.22923700	-0.75995000	1.29590200
C	0.93069200	0.32728800	0.36214300
C	2.34167600	0.11973300	-0.16312700
H	3.03919100	0.80949400	0.32460900
H	2.67239700	-0.90647800	0.02609100
H	2.37540500	0.29611700	-1.24310800
C	-2.08535900	0.59907300	-0.16531300
H	-2.08219400	0.74913200	-1.25014600
H	-3.12213300	0.46043000	0.16277900
H	-1.70610900	1.51083400	0.30804100
H	0.89235600	0.15102600	1.45133000
H	0.61186500	1.36767900	0.19119000

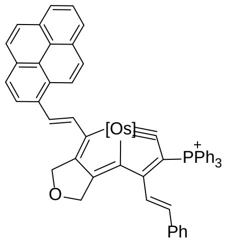

[Os] = OsCl(PPh_3)$_2$

30

E = -5082.38304096

Os	-0.05420900	0.76407000	0.24862000
Cl	1.20718900	2.50009700	1.48543000
P	0.59873200	-0.75668000	1.99459300
P	-0.43144300	2.52244200	-1.36810600
P	-4.50635100	0.06135500	0.83120500
O	0.80488600	-1.82585500	-4.00405700
C	-0.12795500	-1.29184100	-1.95090500
C	1.53066500	0.08022800	-0.94572700

C	5.34340100	0.31034800	-0.81170900
C	-1.71555100	2.25992300	-2.66509800
C	-2.03628100	5.37686800	1.11856100
H	-2.54893700	5.39500900	2.07432700
C	-2.86134400	-0.06027300	0.20348700
C	-2.76924300	3.17127500	-2.83377900
H	-2.85232400	4.03309400	-2.18314000
C	7.71214900	-0.21945400	-0.38829500
C	2.39570300	-1.13903800	2.06814300
C	-0.93185400	4.12069300	-0.63075000
C	-1.79220500	6.56526200	0.42959600
H	-2.11793600	7.51409600	0.84619800
C	2.93229900	-2.38790600	1.73308100
H	2.28395600	-3.21626300	1.47563000
C	1.19096600	-0.84482800	-1.94414400
C	5.60934200	1.63737800	-0.41255200
H	4.80314600	2.36058100	-0.43795800
C	6.23502900	-2.01462000	-1.16944600
H	5.25494500	-2.36909500	-1.46319800
C	-5.70310200	0.03948400	-0.52588000
C	6.41148300	-0.63478300	-0.81469300
C	8.78936000	-1.15490500	-0.33702900
C	7.94357700	1.13256700	0.00944300
C	3.26565700	-0.09720400	2.43383500
H	2.86360100	0.87710800	2.68533300
C	-0.95787200	-0.74499900	-0.95786200
C	-0.69435900	5.31761600	-1.32441200
H	-0.17238300	5.29824800	-2.27563600
C	4.64270400	-0.30440800	2.45192800
H	5.30387900	0.51390600	2.71939900
C	8.57748300	-2.51517100	-0.70744900
C	1.11313700	2.91368100	-2.28230000
C	-0.99933300	-2.87294000	3.00991500
H	-1.15088800	-2.25788500	3.88891700
C	2.86549600	0.56028400	-0.73614700
H	2.96801100	1.42689200	-0.09684500
C	10.08603700	-0.73804100	0.09254700
C	-4.40388300	-2.55577600	1.77867700
H	-3.64627800	-2.73115300	1.02391000
C	7.26438200	-2.90665200	-1.12284200
H	7.09830000	-3.94460800	-1.39925700
C	-3.70729200	2.99134300	-3.85312500
H	-4.50517900	3.71798900	-3.97833000
C	-3.97978800	1.74133400	2.96981400
H	-3.43582700	0.90246000	3.39113400
C	-0.26219800	0.71972900	6.30008700
H	-0.45948300	1.06187700	7.31185900
C	1.41114200	2.34252700	-3.52619600
H	0.67118000	1.75886000	-4.05820500
C	2.08999700	3.68808500	-1.63457800
H	1.87863900	4.12367000	-0.66549500
C	-0.04956800	-3.21447500	0.81556000
H	0.54012400	-2.85442800	-0.01878400
C	9.64793500	-3.42181400	-0.64718600
H	9.48029800	-4.45717000	-0.93127400
C	-5.33478100	2.70636800	1.19427600
H	-5.83984600	2.61249900	0.23913500
C	10.28189100	0.63118700	0.46949800
H	11.27157400	0.94232500	0.79248100
C	6.86984700	2.04195900	-0.01988100
H	7.04427100	3.07414700	0.27150100
C	5.17308100	-1.54760900	2.10292700
H	6.24731700	-1.70178400	2.09639400
C	-2.58422800	0.96945700	-4.53658200
H	-2.49978400	0.11205600	-5.19773700
C	3.33911000	3.87780800	-2.22323300

H	4.08178700	4.48706400	-1.71616400
C	0.59972600	-0.35797300	6.08255500
H	1.07231500	-0.85774600	6.92286300
C	-7.78020300	-0.73168700	-1.49460800
H	-8.69560900	-1.30985000	-1.41663600
C	11.12809500	-1.67841900	0.13622000
H	12.11375500	-1.35781600	0.46218800
C	-0.85463900	1.36469000	5.21419500
H	-1.49814900	2.22399600	5.37897000
C	-4.84786400	-3.60950000	2.57629400
H	-4.43228700	-4.59979600	2.42653100
C	-2.33605300	-0.97488000	-0.78776600
C	3.63926500	3.28390800	-3.45024800
H	4.61889200	3.42090100	-3.89806600
C	-5.81138600	-3.39084800	3.56195800
H	-6.15013900	-4.21581600	4.18140600
C	-5.38235300	3.91216900	1.89334100
H	-5.92514100	4.75446500	1.47647000
C	-2.84189300	-3.22813100	-1.72425200
H	-1.86228600	-3.57139100	-1.40908600
C	-4.03458800	2.94964600	3.65999100
H	-3.53584600	3.04335700	4.61900300
C	-6.30866900	0.71156700	-2.76595700
H	-6.07551100	1.25265700	-3.67545100
C	-4.73786200	4.03250600	3.12508300
H	-4.78440800	4.96989300	3.67124400
C	-7.49102000	-0.02635700	-2.66411800
H	-8.18446200	-0.05512400	-3.49920700
C	2.66964000	2.52209000	-4.10177200
H	2.88528900	2.07142500	-5.06624900
C	-6.33905800	-2.11181400	3.75817700
H	-7.08064700	-1.93903500	4.53185300
C	-0.61535400	-4.48664600	0.76707200
H	-0.44965800	-5.11715700	-0.10214200
C	-1.57193700	-4.14589800	2.95449800
H	-2.16432100	-4.49956300	3.79263200
C	-1.37974900	-4.95787600	1.83807500
H	-1.81389100	-5.95287000	1.80124400
C	-3.68856300	-4.23849000	-2.35034200
C	-3.95412100	-6.55528800	-3.06046700
H	-3.54787300	-7.55809400	-3.15289000
C	-4.98796300	-3.96980700	-2.82473700
H	-5.40218600	-2.96918500	-2.74350500
C	-5.23965800	-6.27367600	-3.52404600
H	-5.83982000	-7.05592300	-3.97879600
C	-1.83346200	0.72543200	0.70096700
C	-1.60994100	4.15706100	0.59405300
H	-1.78864000	3.23618500	1.13623600
C	3.99341300	-0.08570900	-1.15155200
H	3.87628500	-1.01187600	-1.70014600
C	-6.88891000	-0.70136700	-0.42260000
H	-7.10793700	-1.26048300	0.48019400
C	-1.12251600	6.53402700	-0.79554500
H	-0.92975400	7.45505100	-1.33765000
C	-1.64598800	1.14902300	-3.52214700
H	-0.84696600	0.43087600	-3.41315000
C	-0.22128200	-2.39900500	1.94727700
C	-4.94930100	-1.27749800	1.96163200
C	-4.63509400	1.61996500	1.73372100
C	0.26014300	-0.14850900	3.68905800
C	-0.29914100	-2.17703200	-3.16506500
H	-0.25253700	-3.24677000	-2.90047600
H	-1.22835300	-2.01151700	-3.71655600
C	0.86574000	-0.78602300	4.78340300
H	1.54892300	-1.61305900	4.61608700
C	1.88517000	-1.39485900	-3.16556400

H	2.47145400	-0.64549800	-3.70640700	H	-4.47683400	1.29399100	-1.79992000
H	2.54761600	-2.24747700	-2.93305600	C	-5.91502300	-1.05388800	2.95697500
C	4.31516600	-2.58625400	1.74654000	H	-6.32713500	-0.06073200	3.10440300
H	4.72035300	-3.55757400	1.47878400	C	-3.61411800	1.89573900	-4.71164800
C	9.25419900	1.52561500	0.43052600	H	-4.33215500	1.76698700	-5.51665900
H	9.41195700	2.55999600	0.72332500	C	-3.18292600	-1.94944000	-1.45779700
C	-0.59775700	0.93477500	3.91155800	H	-4.19301100	-1.62123600	-1.68191000
H	-1.03556900	1.44891500	3.06589900	C	-3.18914900	-5.54691400	-2.47938400
C	10.90998900	-3.00489800	-0.23021000	H	-2.18747400	-5.76720600	-2.11824400
H	11.72830700	-3.71733300	-0.18977500	C	-5.75159100	-4.97703300	-3.40274800
C	-5.40798000	0.74462700	-1.70402300	H	-6.75101800	-4.75371900	-3.76482800
